# Multi-spatial-scale dynamic interactions between functional sources reveal sex-specific changes in schizophrenia

**DOI:** 10.1162/netn_a_00196

**Published:** 2022-06-01

**Authors:** Armin Iraji, Ashkan Faghiri, Zening Fu, Srinivas Rachakonda, Peter Kochunov, Aysenil Belger, Judy M. Ford, Sarah McEwen, Daniel H. Mathalon, Bryon A. Mueller, Godfrey D. Pearlson, Steven G. Potkin, Adrian Preda, Jessica A. Turner, Theodorus G. M. van Erp, Vince D. Calhoun

**Affiliations:** Tri-Institutional Center for Translational Research in Neuroimaging and Data Science (TReNDS), Georgia State University, Georgia Institute of Technology, and Emory University, Atlanta, GA, USA; Maryland Psychiatric Research Center, Department of Psychiatry, School of Medicine, University of Maryland, Baltimore, MD, USA; Department of Psychiatry, University of North Carolina, Chapel Hill, NC, USA; Department of Psychiatry, University of California San Francisco, San Francisco, CA, USA; San Francisco VA Medical Center, San Francisco, CA, USA; Department of Psychiatry and Biobehavioral Sciences, University of California Los Angeles, Los Angeles, CA, USA; Department of Psychiatry, University of Minnesota, Minneapolis, MN, USA; Departments of Psychiatry and Neuroscience, Yale University, School of Medicine, New Haven, CT, USA; Department of Psychiatry and Human Behavior, University of California Irvine, Irvine, CA, USA; Department of Psychology, Georgia State University, Atlanta, GA, USA; Clinical Translational Neuroscience Laboratory, Department of Psychiatry and Human Behavior, University of California Irvine, Irvine, CA, USA

**Keywords:** Multi-spatial-scale dynamic functional connectivity, Multi-spatial-scale intrinsic connectivity networks, Multi-model-order independent component analysis (ICA), Multiscale ICA (msICA), Resting-state fMRI

## Abstract

We introduce an extension of independent component analysis (ICA), called multiscale ICA, and design an approach to capture dynamic functional source interactions within and between multiple spatial scales. Multiscale ICA estimates functional sources at multiple spatial scales without imposing direct constraints on the size of functional sources, overcomes the limitation of using fixed anatomical locations, and eliminates the need for model-order selection in ICA analysis. We leveraged this approach to study sex-specific and sex-common connectivity patterns in schizophrenia. Results show dynamic reconfiguration and interaction within and between multi-spatial scales. Sex-specific differences occur (a) within the subcortical domain, (b) between the somatomotor and cerebellum domains, and (c) between the temporal domain and several others, including the subcortical, visual, and default mode domains. Most of the sex-specific differences belong to between-spatial-scale functional interactions and are associated with a dynamic state with strong functional interactions between the visual, somatomotor, and temporal domains and their anticorrelation patterns with the rest of the brain. We observed significant correlations between multi-spatial-scale functional interactions and symptom scores, highlighting the importance of multiscale analyses to identify potential biomarkers for schizophrenia. As such, we recommend such analyses as an important option for future functional connectivity studies.

## Author Summary

Brain function can be modeled as the dynamic interactions between functional sources (e.g., intrinsic connectivity networks, ICNs) at different spatial scales. Each spatial scale contains its own functional sources with unique information. For example, the default mode (DM)-ICNs from lower order independent component analysis (ICA) are not a simple union of DM-ICNs from a higher order. Furthermore, dynamic functional interactions occur both within and between different spatial scales, which has been underrepresented. Here, we introduce multiscale ICA to capture functional sources and their interactions across multiple spatial scales. We leveraged this approach to study sex-specific changes in schizophrenia. Most sex-specific differences occur in between-model order, highlighting the benefit of multi-spatial-scale analysis. In sum, studying multi-spatial-scale functional sources provides us with a wealth of information to better characterize brain function.

## INTRODUCTION

### Multi-spatial-Scale Dynamic Interactions

**Figure 1.**
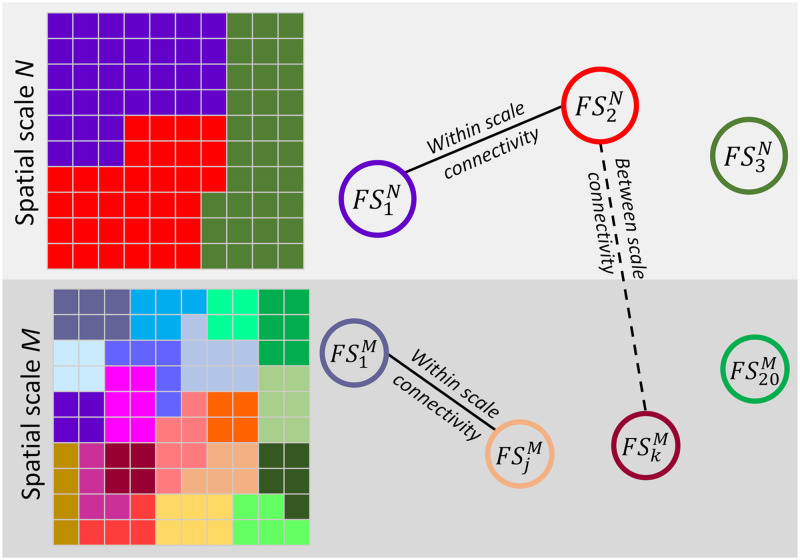
A toy example of multi-spatial-scale analysis. A system with two true spatial scales, *M* and *N*. Each spatial scale has its own set of functional source (FS). Functional sources of spatial scale *M* are not just a simple split of functional sources of scale *N*. Each functional source represents a segregation unit in a given scale, and functional connectivity between functional sources indicates functional integration. Functional interaction (functional connectivity) exist both within and between different spatial scales.

Brain function has been modeled as coordination and interaction between functional sources, which has been summarized via the principles of segregation and integration (Genon et al., 2018). In other words, the brain can be segregated into distinct functional sources (e.g., intrinsic connectivity networks, ICNs), which dynamically interact with each other (i.e., functional integration). Notably, functional sources exist at different spatial scales, and dynamic functional interactions occur both within and between different spatial scales. Previous work has highlighted the importance of analysis at multiple spatial scales (Li et al., 2018); however, most multi-spatial-scale studies have built upon a single set of nodes (e.g., predefined regions or single model-order independent component analysis, ICA) and identifying multiple levels of modularity (e.g., with different resolution parameters) or clusters (e.g., different number of clusters) (Doucet et al., 2011). In the cse of using functional sources as nodes, information at different spatial scales captures functional integration among those sources at multiple resolutions. However, each spatial scale also contains its own functional sources with unique functional information. For instance, larger functional sources are not a simple union of smaller functional sources (Figure 1). In addition, functional interactions occur among functional sources across (within and between) different spatial scales (e.g., large networks interact with small networks), which convey important information about the brain as shown in this study. This relationship is effectively ignored if using a single spatial scale to analyze the data.

Here, we present an approach that combines multiscale ICA (msICA) and functional network connectivity (FNC) to study multi-spatial-scale functional interactions (both within and between spatial scales). Multiscale ICA uses multi-model-order ICA to estimate functional sources at multiple spatial scales directly. Static and dynamic FNC (sFNC/dFNC) were applied to capture static and dynamic interactions between functional sources, both within and between multiple spatial scales. We leveraged this approach to study sex-specific and sex-common schizophrenia differences, which have been understudied but may play an important role in understanding the neural mechanisms as it is clear there are sex differences in schizophrenia, for example in disease onset (Nawka et al., 2013; Li et al., 2016).

### Intrinsic Connectivity Networks: Assessment of Functional Sources

A functional source can be defined as a temporally synchronized pattern (Iraji, Miller, et al., 2020), and studying brain function requires a proper estimation of functional sources to prevent incorrect functional connectivity inferences (Iraji, Miller, et al., 2020). Because of its emphasis on capturing spatially distinct and temporally coherent sources, ICA has proven itself to be a strong method to identify functional source estimates. ICA is a data-driven multivariate technique, which divides the brain into overlapping functionally distinct patterns (Calhoun & Adalı, 2012; Calhoun & de Lacy, 2017; Kaboodvand et al., 2018), called intrinsic connectivity networks. Each ICN is a temporally synchronized pattern of the brain, a good estimation of a functional source. The ICN time course describes its functional activity over time, while its spatial pattern indicates the contribution of spatial locations to ICN. The spatial scale of ICNs can be set effectively using the model orders of ICA. In other words, we can study brain segregation and estimate ICNs at different spatial scales by using ICA with different model orders. Low-model-order ICAs result in large-scale spatially distributed ICNs (Damoiseaux et al., 2008; Iraji et al., 2016), while higher model order results in more spatially granular ICNs (Allen et al., 2011; Iraji, Faghiri, et al., 2019; Iraji, Fu, et al., 2019). Therefore, we proposed to use msICA (running ICA with multiple orders) to estimate functional sources of multiple spatial scales. While there have been a few studies of the effect of model order on the spatial maps of ICNs (Abou-Elseoud et al., 2010), to our knowledge there is no work that has studied brain function across multiple model orders. Similarly, no work has yet evaluated dynamic functional interaction jointly at multiple model orders.

It is worth mentioning that ICA does not impose a direct constraint on the spatial extent of functional sources estimates; thus, msICA allows data itself determine the spatial extent of estimated functional sources without generating spurious sources for different spatial scales. In other words, msICA does not force the functional sources of a given spatial scale to have a similar spatial extent. This gives msICA a great advantage as we do not expect different brain areas or functional domains, such as “the primary cortex versus the frontal lobe” and “the visual domain versus the cognitive control domain across,” to be parceled at the same level of granularity (see the Results section, Multi-spatial-Scale Functional Segregation: Intrinsic Connectivity Networks).

Multiscale ICA also addresses the model-order selection problem because, in general, one remedy to parameter selections is finding a procedure to combine results from several parameters. Various information-theoretic criteria such as the minimum description-length criterion (MDL) and Akaike’s information criterion (AIC) have been used to estimate an optimal model order. However, the optimal number can vary across them; as such, the model-order selection problem still remains as selecting the estimation method. Instead of focusing on a single model order selected by these approaches, msICA includes information on all spatial scales (within the constraints of the number of model orders we use).

### Functional Network Connectivity: Assessment of Functional Interaction

While ICA effectively segregates the brain into ICNs, FNC provides a way to study functional interaction and integration. FNC is defined as the temporal dependency among ICNs and is commonly estimated using Pearson’s correlation coefficient between ICN time courses (Jafri et al., 2008). Thus, FNC characterizes the functionally integrated relationship across the brain by calculating the functional interaction between ICNs.

Traditionally, functional integration has been studied using sFNC, where the overall functional interactions are calculated using scan-length averaged FNC. However, the brain constantly integrates and processes the information in real time. Considering the brain’s rich, dynamic nature, a number of methods have moved beyond the “static” oversimplification and evaluate the temporal reconfiguration of functional interactions using dFNC (Allen et al., 2014; Calhoun et al., 2014; Iraji, Faghiri, et al., 2020). The dFNC approaches calculate time-resolved FNC, allowing us to study variations in functional integrations over time and identify different brain functional interaction patterns, also known as brain functional states (Iraji, Faghiri, et al., 2020).

### Schizophrenia

Schizophrenia is a psychotic disorder accompanied by various cognitive impairments and a decrease in social and occupational functioning. Schizophrenia is a heterogeneous syndromic diagnosis of exclusion, lacks unique symptoms, and is diagnosed clinically by both positive symptoms (such as delusions, hallucinations, disorganized speech, disorganized, or catatonic behavior), and negative symptoms (such as apathy, blunted affect, and anhedonia; American Psychiatric Association, 2013), plus a decline in social functioning. Schizophrenia overlaps considerably with both schizo-affective disorder and psychotic bipolar disorder, not only symptomatically, but also in terms of genetics and at the level of other biomarkers (Clementz et al., 2016). The diverse temporal trajectory across individuals with schizophrenia and the different types of clinical symptoms suggest alterations in various functional domains and brain capacity reductions to integrate information across the brain. Schizophrenia has been hypothesized as a developmental disorder of disrupted brain function, which can be characterized by functional dysconnectivity and/or changes in functional integration (Friston & Frith, 1995; Kahn et al., 2015; Stephan et al., 2006). Therefore, studying static and dynamic FNC can provide vital information about brain functional integration and its schizophrenia changes, potentially improving our understanding of the actual brain pathology underlying different schizophrenia subcategories.

In early work, Meda et al. show abnormal FNC, including those related to paralimbic circuits, which were correlated significantly with PANSS negative scores (Meda et al., 2012). Focusing on the default mode, hypoconnectivity was observed across all related networks (Meda et al., 2014). Dynamic studies also identify hypoconnectivity as the dominant dysconnectivity pattern, while identifying few consistent hyperconnectivity patterns. The strengths of dynamic functional connectivity (dFC) between subcortical and sensory networks are weaker in individuals with schizophrenia (Damaraju et al., 2014). The weaker dFC strengths have also been observed in several brain networks in spatial dynamic studies (Iraji, Deramus, et al., 2019; Iraji, Fu, et al., 2019). The decrease in the strengths of dFC (transient hypoconnectivity) seems to be accompanied by higher fluctuations of dFC between brain regions (Yue et al., 2018) and within and between several brain networks (Iraji, Deramus, et al., 2019; Ma et al., 2014). Sun et al. (2019) reported overall higher global efficiency across the schizophrenia brain. The alteration in the dFNC patterns in schizophrenia also seems to be related to cognitive performance (Fu et al., 2018; Iraji, Deramus, et al., 2019; Yue et al., 2018). For instance, the temporal variability of FNC between the amygdala-medial prefrontal cortex (mPFC) is positively correlated with total symptom severity and negatively correlated with information-processing efficiency (Yue et al., 2018). The correlation between the energy index (spatiotemporal uniformity) of the subcortical domain and the attention/vigilance domain of computerized multiphasic interactive neurocognitive dualdisplay system (CMINDS) was reported to be disrupted in schizophrenia (Iraji, Deramus, et al., 2019). Studies also show frequency-specific dFC alterations in schizophrenia patients (Faghiri et al., 2021; Yaesoubi et al., 2017; Zhang et al., 2018). However, previous studies have not studied functional interactions across multiple spatial scales and have underappreciated differences between male and female cohorts (Damaraju et al., 2014; Faghiri et al., 2021; Iraji, Deramus, et al., 2019; Miller et al., 2019; Miller et al., 2016).

Schizophrenia incidence is higher in men (Aleman et al., 2003; McGrath et al., 2004), but paradoxically there is equal overall prevalence (Saha et al., 2005). There is also evidence suggesting sex differences in onset, symptom expression, and outcome in schizophrenia (Li et al., 2016; Navarro et al., 1996; Nawka et al., 2013). For instance, males have more severe overall symptoms, worse outcomes, and more negative and fewer affective symptoms, and they experience symptoms earlier than females (Li et al., 2016). Furthermore, symptoms respond more quickly to treatments in females. However, sex differences in symptoms and outcomes also depend on the age of onset and treatment (Li et al., 2016; Seeman, 2019). Understanding sex-specific characteristics of functional connectivity, which is currently lacking in the field, can help provide an important insight to understand sex differences in schizophrenia and potentially the opportunity to deliver sex-specific treatments and care for individuals with schizophrenia.

Considering the previous static and dynamic FNC findings on sex differences in typical control cohorts (Allen et al., 2011; Yaesoubi et al., 2020) and previous reports on sex differences in schizophrenia (Li et al., 2016; Navarro et al., 1996; Nawka et al., 2013), we hypothesize that multiscale functional interactions capture sex-specific changes in schizophrenia, which are significantly correlated with schizophrenia’s symptoms score. We examined our hypothesis using the following pipeline: (a) We estimated ICNs at multiple spatial scales using ICA with model orders of 25, 50, 75, and 100. (b) We calculated the multi-spatial-scale static and dynamic functional integrations using within and between model orders sFNC and dFNC using a window-based approach (Allen et al., 2014; Iraji, Faghiri, et al., 2020). (c) We evaluated sex-specific differences between typical controls and individuals with schizophrenia.

## MATERIALS AND METHODS

### Participant Demographics and Data Inclusion Criteria

The data used in this study were selected from three projects: FBIRN (Functional Imaging Biomedical Informatics Research Network), MPRC (Maryland Psychiatric Research Center), and COBRE (Center for Biomedical Research Excellence). We selected a subset of data that satisfy the inclusion criteria, including (a) data of individuals with typical control or schizophrenia diagnosis; (b) data with high-quality registration to echo-planar imaging (EPI) template; and (c) head motion transition of less than 3° rotations and 3-mm translations in every direction (Fu et al., 2020). Mean framewise displacement among selected subjects is average ± standard deviation = 0.1778 ± 0.1228; min ∼ man = 0.0355 ∼ 0.9441. Thus, we report on resting-state fMRI (rsfMRI) data from 827 individuals, including 477 typical controls and 350 individuals with schizophrenia selected (Table 1).

**Table 1.** Demographic information of the data used in the study. FBIRN: Functional Imaging Biomedical Informatics Research Network. MPRC: Maryland Psychiatric Research Center. COBRE: Center for Biomedical Research Excellence.

**Project**	**Diagnostic**	***N***	**Sex**	***N***	**Age (years)**	
					**Mean ± *SD***	**Median/range**
**FBIRN**	Control group	160	Male	115	37.26 ± 10.71	39/(19–59)
			Female	45	36.47 ± 11.33	33/(19–58)
	Schizophrenia group	150	Male	114	38.74 ± 11.78	40/(18–62)
			Female	36	39.06 ± 11.40	36/(21–57)
**MPRC**	Control group	238	Male	94	38.72 ± 13.63	40/(12–68)
			Female	144	41.22 ± 16.06	44/(10–79)
	Schizophrenia group	150	Male	98	35.57 ± 13.18	32/(13–63)
			Female	52	44.60 ± 13.87	47/(13–63)
**COBRE**	Control group	79	Male	55	39.07 ± 12.43	38/(18–65)
			Female	24	34.92 ± 10.23	34/(18–58)
	Schizophrenia group	50	Male	42	37.43 ± 15.05	32.5/(19–64)
			Female	8	43.25 ± 12.78	40/(31–65)

### Data Acquisition

The FBIRN dataset was collected from seven sites. The same rsfMRI parameters were used across all sites: a standard gradient EPI sequence, repetition time (TR)/echo time (TE) = 2,000/30 ms, voxel spacing size = 3.4375 × 3.4375 × 4 mm, slice gap = 1 mm, flip angle (FA) = 77°, field of view (FOV) = 220 × 220 mm, and a total of 162 volume. Six of the seven sites used 3-Tesla Siemens Tim Trio scanners, and one site used a 3-Tesla General Electric Discovery MR750 scanner.

The MPRC dataset was collected in three sites using a standard EPI sequence, including Siemens 3-Tesla Siemens Allegra scanner (TR/TE = 2,000/27 ms, voxel spacing size = 3.44 × 3.44 × 4 mm, FOV = 220 × 220 mm, and 150 volumes); 3-Tesla Siemens Trio scanner (TR/TE = 2,210/30 ms, voxel spacing size = 3.44 × 3.44 × 4 mm, FOV = 220 × 220 mm, and 140 volumes); and 3-Tesla Siemens Tim Trio scanner (TR/TE = 2,000/30 ms, voxel spacing size = 1.72 × 1.72 × 4 mm, FOV = 220 × 220 mm, and 444 volumes).

The COBRE dataset was collected in one site using a standard EPI sequence with TR/TE = 2,000/29 ms, voxel spacing size = 3.75 × 3.75 × 4.5 mm, slice gap = 1.05 mm, FA = 75°, FOV = 240 × 240 mm, and a total of 149 volumes. Data were collected using a 3-Tesla Siemens Tim Trio scanner.

### Preprocessing/MRI Data Preprocessing

The preprocessing was performed primarily using the statistical parametric mapping (SPM12, https://www.fil.ion.ucl.ac.uk/spm/) toolbox. The rsfMRI data preprocessing used the following steps: (a) discarding the first five volumes for magnetization equilibrium purposes, (b) rigid motion correction to correct subject head motion during scan, and (c) slice-time correction to account for temporal misalignment in data acquisition. Next, the data of each subject were nonlinearly registered to a Montreal Neurological Institute (MNI) EPI template, resampled to 3 mm^3^ isotropic voxels, and spatially smoothed using a Gaussian kernel with a 6 mm full width at half-maximum (FWHM = 6 mm). The voxel time courses were then z-scored (variance normalized). We are interested in identifying functional sources, temporally synchronized regions. Therefore, temporal coupling and not amplitude information is the information of interest. Variance normalization was shown to enhance sensitivity to functional segregation and functional sources (Iraji, Fu, et al., 2019) and to be highly reproducible across different studies. Furthermore, prior to calculating static and dynamic FNC, an additional post hoc cleaning procedure was performed on the time courses of ICNs to reduce the effect of remaining noise, which may not be wholly removed using ICA, and to improve the detection of dynamic FNC patterns (Allen et al., 2014). ICN time courses were detrended by removing linear, quadratic, and cubic trends. The six motion realignment parameters and their derivatives were regressed out. Outliers were detected based on the median absolute deviation, similar to that implemented in AFNI 3Ddespike (https://afni.nimh.nih.gov/), and replaced with the best estimate using a third-order spline fit to the clean portions of the time courses. Bandpass filtering was applied using a fifth-order Butterworth filter with a cutoff frequency of 0.01–0.15 Hz.

### Intrinsic Connectivity Network Estimation

For the initial work in this paper, we utilized spatial ICA with several model orders (25, 50, 75, and 100) to identify ICNs at multiple spatial scales. Similar to most ICA-based studies of fMRI, we implemented group-level spatial ICA followed by a back-reconstruction technique to estimate subject-specific independent components time courses.

We used the GIFT toolbox (https://trendscenter.org/software/gift/; Calhoun & Adalı, 2012; Calhoun et al., 2001; Iraji, Faghiri, et al., 2020). First, subject-specific spatial principal components analysis (PCA) was applied to normalize the data and to allow subjects to contribute similarly in the common subspace. The subject-specific PCA also has denoising and computational benefits (Erhardt et al., 2011). We retain maximum subject-level variance (greater than 99.99%). While the subject-specific PCA privileges subject differences at the subject level, the group-level PCA favors subject commonalities (Erhardt et al., 2011). All subject-level principal components were concatenated together across the time dimension, and group-level spatial PCA was applied to concatenated subject-level principal components. *N* (25, 50, 75, and 100) group-level principal components that explained the maximum variance were selected as the input for spatial ICA to calculate *N* (25, 50, 75, and 100) group independent components.

Infomax was chosen as the ICA algorithm because it has been widely used and compares favorability with other algorithms (Correa et al., 2007; Correa et al., 2005). For each model order (N = 25, 50, 75, and 100), the Infomax ICA algorithm was run 100 times and clustered together within the ICASSO framework (Himberg et al., 2004). The run with the closest independent components to the centrotypes of stable clusters (ICASSO cluster quality index > 0.8) was selected as the best run and used for future analysis (Ma et al., 2011). This is an important point and facilitates replicable results. Next, the subject-specific independent components time courses were calculated using the spatial multiple regression technique (Calhoun et al., 2004). At each time point, the contribution of each independent component to the BOLD signal was calculated using linear regression (Calhoun et al., 2004).

We selected a subset of independent components as ICNs if they are stable (ICASSO stability index > 0.8) and depict common ICN properties including (a) dominant low-frequency fluctuations of their time courses evaluated using dynamic range and the ratio of low-frequency to high-frequency power; (b) peak activations in the gray matter; (c) low spatial overlap with vascular, ventricular; and (d) low spatial similarity with motion and other known artifacts. Finally, ICNs were grouped into functional domains based on prior knowledge of their anatomical and functional properties (Allen et al., 2011).

### Static and Dynamic FNC Calculation

We calculated static and dynamic functional network connectivity between every single pair of ICNs across all model orders to effectively capture functional integration and interaction across different spatial scales. For a subset of data (15%) with a sampling rate different from 2 s, ICN time courses were interpolated to 2 s. Minimum data length across all subjects was selected for further analysis. Static FNC (sFNC) was estimated by calculating the Pearson correlation between each pair of ICN time courses resulting in one sFNC matrix for each individual. Each element of the sFNC matrix is the functional connectivity between a pair of ICNs.

In contrast to sFNC, which uses the full length of scan, in dynamic FNC (dFNC), we calculate multiple FNC matrices for different time segments of scan (i.e., FNC matrices for durations smaller than the whole time series; Iraji, Faghiri, et al., 2020). As a result, we can study variations in FNC over time. Here, we used a window-based approach with the slide step size of 2 s (maximum overlap between consecutive windows). A recommended window size is between 30 s and 60 s (Iraji, Faghiri, et al., 2020); thus, we chose the middle value (44 s, time point increment is 2 s). A tapered window was created by convolving a rectangle window (width = 44 s) with a Gaussian (σ = 6 s) and was used to calculate windowed FNC. This results in a series of windowed-FNC matrices over time (FNC as a function of time) containing dFNC information.

Next, we identified dFNC states from windowed-FNC matrices using the k-means clustering, in which each cluster represents one dynamic state (Iraji, Faghiri, et al., 2020). We applied a two-stage k-means clustering. First, windows with local maxima with FNC variances were selected for each subject, and k-means clustering was applied to the set of all subject-specific local maxima (also known as exemplars). We used the city-block distance metric because it is suggested to be a more effective dissimilarity measure than Euclidean distance for high-dimensional data (Aggarwal et al., 2001). K-means clustering was repeated 100 times with different initializations using the k-means++ technique to increase the chance of escaping local minima. The resulting centroids were then used to initialize a clustering to all 93,451 (827 subjects × 113 windows) windowed-FNC matrices. The optimal number of dFNC states was selected based on the elbow criterion by calculating the ratio of within- to between-cluster variance and running the clustering procedure for 1 to 15 clusters. Subject-specific dFNC states were next estimated by averaging windowed FNC of time windows assigned to a given state.

We repeated the dFNC state identification procedures using two alternative ways to ensure that the dFNC states are not biased to the clustering algorithm. (a) We first applied k-means clustering at the subject level and then concatenated the subject-level centroids for group-level clustering and identifying dFNC states. 2) We directly applied k-mean clustering to all 93,451 (827 subjects × 113 windows) windowed-FNC matrices. We also evaluated the clustering results using Euclidean and correlation distances.

### Group Comparison Analysis

We evaluated sex-specific differences in multiscale sFNC and dFNC between the control group (CT) and the individuals with schizophrenia (SZ). For each sex cohort, male and female, we separately assessed diagnostic group differences, that is, male controls versus male individuals with schizophrenia (maleCT vs. maleSZ) and female controls versus female individuals with schizophrenia (femaleCT vs. femaleSZ). We used a general linear model (GLM) with age, data acquisition site, and mean framewise displacement as covariates. Framewise displacement is the sum of changes in the six rigid-body transform parameters (framewise displacement(t) = |Δdx(t)| + |Δdy(t)| + |Δdz(t)| + |Δα(t)| + |Δβ(t)| + |Δγ(t)|). Mean framewise displacement was added to the GLM to account for any residual motion effect that was not removed in the previous three motion-removal steps. The statistical analysis results were corrected for multiple comparisons using a 5% false discovery rate (FDR). It is worth mentioning that all statistical analysis results were combined (sFNC and dFNC; male and female; across all model orders) and corrected for multiple comparisons, which is more conservative than correcting for each statistical analysis separately.

Next, we evaluated sex-specific differences for the sFNC and dFNC features that showed a significant difference between the control group and individuals with schizophrenia in either of the sex cohorts (“maleCT vs. maleSZ” and/or “femaleCT vs. femalesSZ”). For each feature, we compared the difference of the *t* value of the GLM statistic between two sex cohorts (“*t* value of maleCT vs. maleSZ” − “*t* value of femaleSZ vs. femaleCT”) with a null distribution. The *p* value of the *t* value of difference was corrected for multiple comparisons using the same procedure explained in the previous paragraph.

The null distribution was created by randomly permuting sex labels within each diagnostic group. In other words, the diagnostic label remained intact; individuals with schizophrenia remained schizophrenia, and control subjects remained in the control group, and only the sex labels were randomly permuted. Furthermore, the number of females and males in each diagnostic group did not change. This permutation process was repeated 5,000 times. For each permutation, the GLM was applied to two null male and null female cohorts independently. For each feature, the difference of the *t* value of diagnosis for two null cohorts was calculated. This results in 5,000 samples of the null distribution for each feature.

We also studied sex-specific differences at the domain level across different spatial scales. For static FNC and each dynamic state, the average FNC was calculated within and between seven functional domains both within and between four model orders (e.g., “CC25-DM25 and “CR50-VS100”). For example, “CR50-VS100” is the average FNC between every pair of ICNs that belong to the cerebellum domain model order 50 and ICNs that belong to the visual domain model order 100. This results in a 28 × 28 domain-level functional integration matrix. The static and dynamic state domain-level functional integration matrices were then evaluated for sex-specific differences.

### Relationship With Symptom Scores

We further evaluate whether the multiscale functional network connectivity pairs showing sex-specific changes in schizophrenia are related to the symptoms of schizophrenia. The positive and negative syndrome scale (PANSS) scores are available for the FBIRN dataset, while the MPRC dataset includes the Brief Psychiatric Rating Scale (BPRS) scores. We transformed BPRS total scores to PANSS total scores using the matching obtained from 3,767 individuals (Leucht et al., 2013). Next, we evaluated the relationship between the PANSS total score and domain-level features with significant sex-specific differences. Correlation analyses were conducted after regressing out age, site, and mean functional domain and corrected for multiple comparisons.

## RESULTS

### Multi-spatial-Scale Functional Segregation: Intrinsic Connectivity Networks

**Figure 2.**
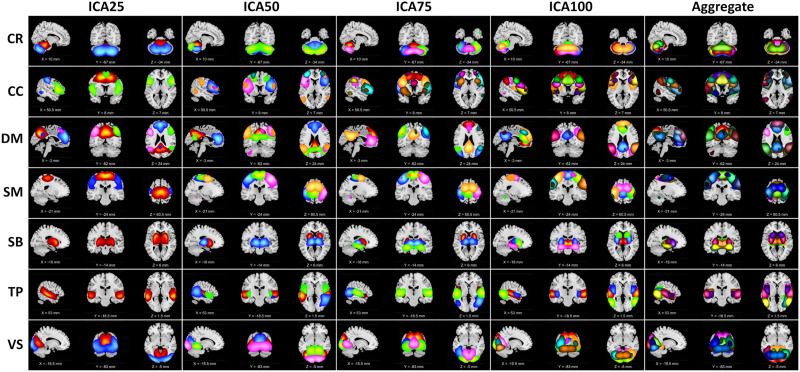
Visualization of the intrinsic connectivity networks (ICNs) identified from four ICA model orders of 25, 50, 75, and 100. ICNs were grouped into seven functional domains based on their anatomical and functional properties. The functional domains are cognitive control (CC), default mode (DM), visual (VS), subcortical (SB), cerebellum (CR), somatomotor (SM), and temporal (TP). Columns represent the composite maps of seven functional domains for four ICA model orders and aggregated. Each color represents the spatial map of one ICN thresholded at |Z| > 1.96 (*p* = 0.05).

We performed spatial ICA with 25, 50, 75, and 100 components on rsfMRI data from 827 subjects to functionally segregate the brain at different spatial scales. Based on the criteria explained in the Materials and Methods section (see Intrinsic Connectivity Network Estimation subsection), we identified 15, 28, 36, and 48 independent components as ICNs for model orders 25, 50, 75, and 100, respectively. Detailed information of the ICNs, including spatial maps, coordinates of peak activations, and temporal and frequency information, can be found in the Supporting Information, Supplementary 1. ICNs were grouped into seven functional domains, including cognitive control (CC), cerebellum (CR), default mode (DM), subcortical (SB), somatomotor (SM), temporal (TP), and visual (VS). Figure 2 illustrates the composite views of functional domains for each model order and aggregated. Each composite view is obtained by thresholding and overlaying associated ICNs. For example, the first image in subplot (CR, ICA25) was obtained by thresholding (|Z| > 1.96) and overlaying two ICNs associated with the cerebellum domain in model order 25. Table 2 shows the number of ICNs for each model order and functional domain. The results suggest that as the model order increases, the number of ICNs increases, and the brain and the functional domains segregate into more functional sources (ICNs). For instance, the subcortical domain consists of only one ICN in model order 25, enclosing the whole subcortical regions, while it parcels into spatially distinct ICNs as model order increases. However, the number of ICNs does not increase proportionally with model order. While some functional domains break into more ICNs as the model order increases, others demonstrate a smaller amount of changes in the number of ICNs and their spatial distributions across model orders studied in this work. For example, we observe significant changes in the ICNs associated with the cognitive control domain across model orders, particularly between model order 50 and 75, while the number of ICNs are the same for model order 50 and 75 for the somatomotor and visual domains. Interestingly, across different model orders, we observed ICNs with high spatial overlap (high spatial similarity) but clearly distinct features. The second row of Figure 5 shows two distinct ICNs with high spatial overlap associated with the primary motor cortex.

**Table 2.** The number of intrinsic connectivity networks (ICNs) for each model order and functional domains, cognitive control (CC), cerebellum (CR), default mode (DM), subcortical (SB), somatomotor (SM), and temporal (TP).

	*CR*	*CC*	*DM*	*SM*	*SB*	*TP*	*VS*	*Total*
*IC25*	2	3	4	2	1	1	2	*15*
*IC50*	3	6	5	5	2	3	4	*28*
*IC75*	4	11	6	5	3	3	4	*36*
*IC100*	5	14	8	7	4	3	7	*48*
*Total*	*14*	*34*	*23*	*19*	*10*	*10*	*17*	

### Dynamic Functional Integration: Static/Dynamic Functional Network Connectivity

**Figure 3.**
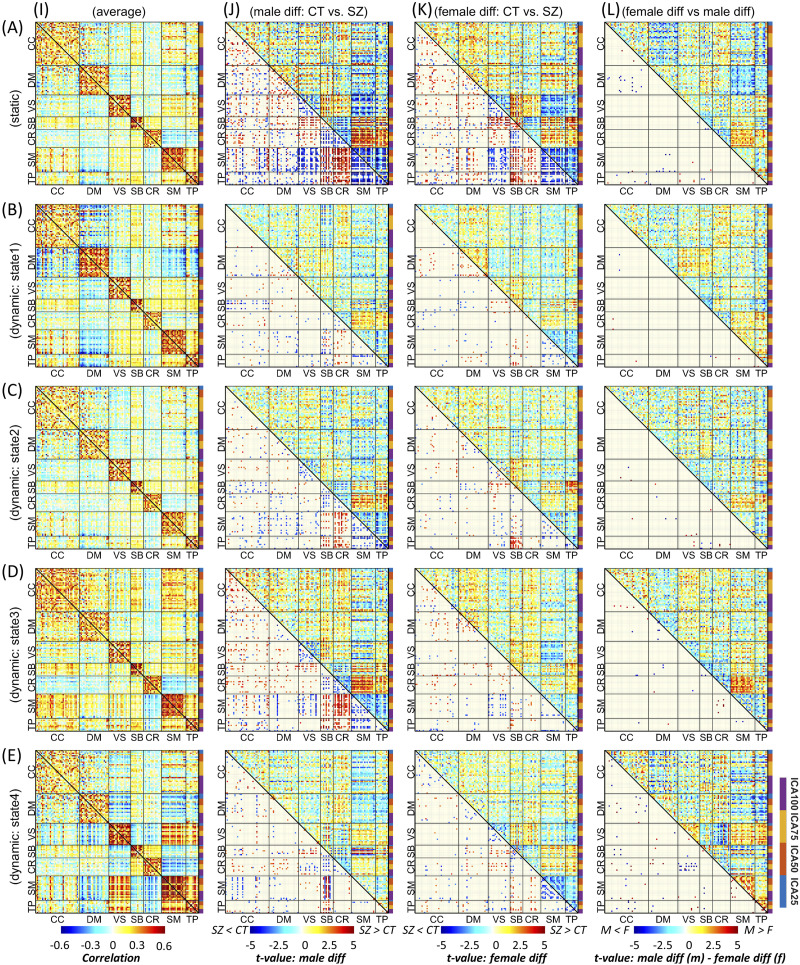
Block plot of multiscale functional integration. ICNs are sorted by functional domain and then by model order. Row A is the result of static FNC analysis, and rows B to E represent the four dynamic states. Column I is the average FNC matrix for static FNC and dynamic FNC states. Column J shows the result of group comparison between male individuals with schizophrenia (SZ) and the male control group (CT). Column K shows the result of group comparison between SZ and CT individuals in the female cohort. In columns J and K, the upper triangular shows the *t* value of statistical comparisons, and the lower triangular shows statistically significant differences after FDR correction for multiple comparisons (FDR-corrected threshold = 0.05). Column L shows the result of the statistical comparison between the differences observed in the male cohort versus the female cohort. The upper triangular in column L shows the differences between the *t* value of statistical comparisons in male and female cohorts (“*t* value of maleSZ vs. maleCT” − “*t* value of femaleSZ vs. femaleCT”), and the lower triangular shows the SZ-associated abnormal patterns that are significantly different between male and female cohorts after FDR correction. Cognitive control (CC), default mode (DM), visual (VS), subcortical (SB), cerebellum (CR), somatomotor (SM), and temporal (TP).

**Figure 4.**
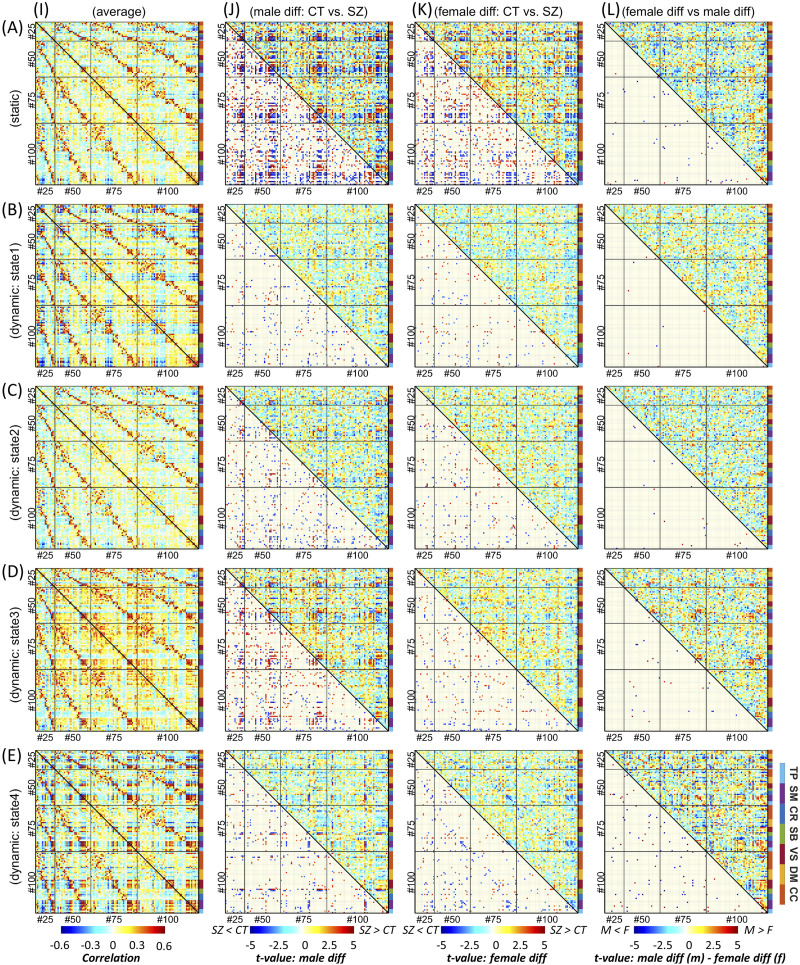
Finger plot of multiscale functional integration. ICNs are sorted by model order and then by functional domain. Row A is the result of static FNC analysis, and rows B to E represent the four dynamic states. Column I is the average FNC matrix for static FNC and dynamic FNC states. Column J shows the result of group comparison between male individuals with schizophrenia (SZ) and the male control group (CT). Column K shows the result of group comparison between SZ and CT individuals in the female cohort. In columns J and K, the upper triangular shows the *t* value of statistical comparisons, and the lower triangular shows statistically significant differences after FDR correction for multiple comparisons (FDR-corrected threshold = 0.05). Column L shows the result of the statistical comparison between the differences observed in the male cohort versus the female cohort. The upper triangular in column L shows the differences between the *t* value of statistical comparisons in male and female cohorts (“*t* value of maleSZ vs. maleCT” − “*t* value of femaleSZ vs. femaleCT”), and the lower triangular shows the SZ-associated abnormal patterns that are significantly different between male and female cohorts after FDR correction. Cognitive control (CC), default mode (DM), visual (VS), subcortical (SB), cerebellum (CR), somatomotor (SM), and temporal (TP).

Figure 3 (A, I) and Figure 4 (A, I) display block and finger plots of the group-level multiscale functional integration computed using the entire scan length (i.e., static functional network connectivity, sFNC). Static FNC shows similar patterns for control groups, individuals with schizophrenia, males, and females. In the block plot, we sort ICNs by functional domain and then by model order. The block plot of sFNC resembles previous single model-order studies, showing modular organization within functional domains across model orders. Consistent with prior literature (Allen et al., 2011), we observed an overall negative association (anticorrelation) between the default model and the rest of the brain, particularly the visual, somatomotor, and temporal domains, during rest. Interestingly, this negative association was more prominent between model orders, for example, between the default mode of model order 25 (DM25) and the somatomotor of model order 100 (SM100). We also observed strong FNC between the somatomotor, temporal, and visual domains, and between the subcortical and cerebellum domains. Figure 3 suggests that the FNC within functional domains is stronger than between functional domains, and this pattern is consistent for both within and between model orders. The similarity in FNC pattern within and between model orders can be observed in the finger plots (Figure 4), where ICNs are sorted first by model order and then by functional domains. The finger plot (Figure 4) shows functional domain modular patterns (stronger FNC within functional domains compared with between functional domains) between model orders similar to within model orders.

Focusing on brain dynamics, dynamic FNC (dFNC) analysis shows variations in FNC over time, which give rise to distinct functional integration patterns (dFNC states). The elbow criterion identified four as the optimal number of states. Figure 3 and Figure 4 show the dFNC states. These states are fully reproducible and identified using different clustering procedures (see the Static and Dynamic FNC Calculation section above). State 1 accounts for 23.76% of all windows (percentage of occurrences, POC = 23.76%), and it is dominated by a strong anticorrelation pattern between the default mode and other functional domains, which can be related to the role of the default mode in reconciling information and subserve the baseline mental activity. State 2 (POC = 38.3%) is distinct by weaker FNC, particularly weaker between functional domains potentially representing the brain’s global segregation state. In contrast, State 3 (POC = 21.31%) demonstrates overall positive FNC across the cerebral cortex, potentially representing global functional integration. Of particular note, the cerebellum shows overall negative FNC with cerebral functional domains in State 3. The negative association between the cerebellar domain and sensorimotor functional domains is prominent in State 4 with POC = 16.60%. State 4 can be distinguished with strong functional integration between the visual, somatomotor, and temporal domains, and their anticorrelation patterns with the rest of the brain. This state also shows strong functional integration between the subcortical and cerebellar domains.

### Sex-Specific Differences in Individuals with Schizophrenia

Multiscale functional integration was further studied by evaluating sex-specific differences in multiscale sFNC and dFNC between the control group (CT) and the individuals with schizophrenia (SZ). In Figure 3 and Figure 4, columns J and K show the statistical analysis for each sex cohort using a general linear model (GLM) with age, data acquisition site, and mean framewise displacement as covariates.

In general, sFNC shows more differences between SZ and CT in both sex cohorts than each dFNC state individually; however, the total number of tests that survived FDR correction is comparable between sFNC and dFNC (Supporting Information, Supplementary 2). In the female cohort, 576 FNC pairs show significant differences in both sFNC and dFNC, while we identified 638 and 402 FNC pairs showing significant differences only in sFNC and dFNC, respectively. In the male cohort, the number of FNC pairs that show significant differences in both sFNC and dFNC is 1,076, and the numbers of FNC pairs that show significant differences only in sFNC and dFNC are 720 and 640, respectively. Furthermore, dFNC analysis shows that in the female (male) cohort, 790 (1,246) and 3 (21) FNC pairs, respectively, show significant differences in only one dynamic state and all four dynamic states.

Individuals with schizophrenia show reduced sFNC strength within and between the SM and TP domains in male and female cohorts. Looking at dFNC results, we observed these differences emerge in different states for male and female cohorts, that is, mainly in State 3 for the male cohort and State 4 for females. We observed that the sex-specific differences in the SM and TP domains are more pronounced in dFNC states, particularly in State 4. Individuals with SZ also have weaker sFNC and dFNC within the VS and between VS domain and SM and TP domains. Furthermore, with a few exceptions, we observed an overall sFNC and dFNC increase between the SB and the CR, on the one hand, and the SM, the TP, and the VIS on the other hand. We observed the strongest sex-specific differences in State 4 between the VS and the CR.

The results also show significant differences between male and female cohorts across other functional domains in both sFNC and dFNC. For instance, the sFNC between the CC and DM shows significant differences in SZ-related alterations between male and female cohorts.

**Figure 5.**
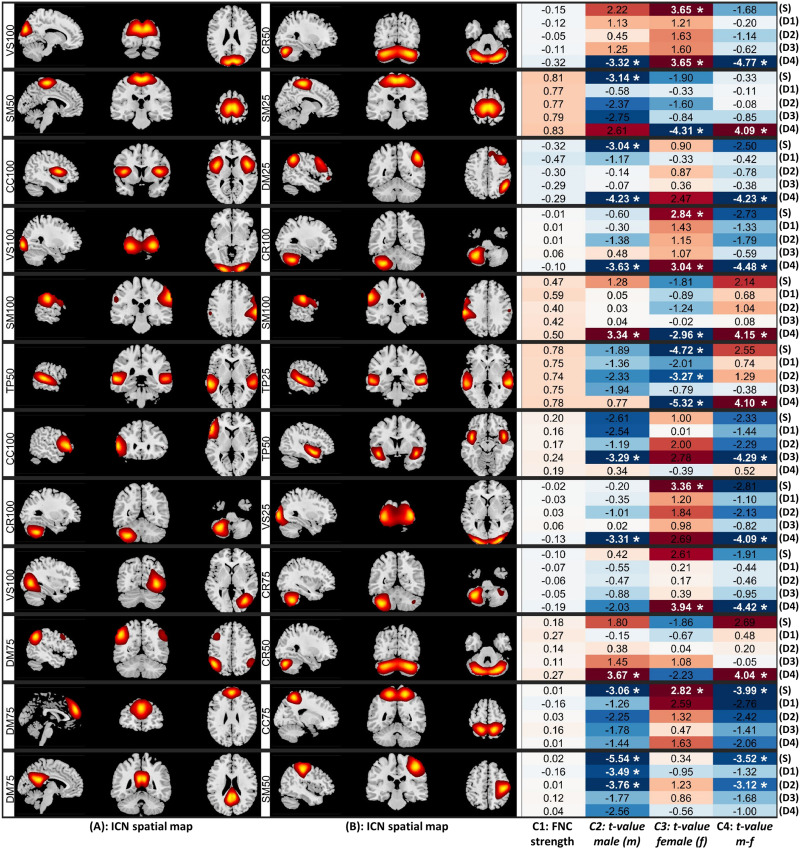
Static and dynamic functional network connectivity (sFNC/dFNC) pairs that show the largest sex-specific multiscale changes in schizophrenia (SZ) presented in 12 rows in order. (S) represents the results of sFNC, and (D1) to (D4) show the results of dFNC for dynamic State 1 to 4, respectively. (A) and (B) display the sagittal, coronal, and axial views of the peak activation of intrinsic connectivity networks (ICNs) associated with each FNC pair. (C1) is the FNC strength. (C2) indicates the *t* value of statistical comparisons between typical control and individual with schizophrenia in the male cohort. Positive (negative) values indicate stronger (weaker) sFNC/dFNC in individuals with schizophrenia (SZ) compared with the control group. (C3) represents the *t* value of statistical comparisons between typical control and individual with schizophrenia in the female cohort, where positive and negative values indicate the same pattern as (C2). (C4) shows the *t* value of comparing schizophrenia-related changes between male and female cohorts (“*t* value of maleSZ vs. maleCT” − “*t* value of femaleSZ vs. femaleCT”). Asterisk sign * indicates the statistical comparisons that survived multiple comparisons (5% false discovery rate, FDR). Cognitive control (CC), default mode (DM), visual (VS), subcortical (SB), cerebellum (CR), somatomotor (SM), and temporal (TP). The number after the functional domain abbreviation is the model number; for example, DM25 means the default model domain from ICA model order 25.

The sex-specific differences are more prevalent in State 4 than sFNC and other dynamic states (Supporting Information, Supplementary 2). The number of FNC pairs that show significant sex differences in both sFNC and dFNC is only nine. The results also suggest that the largest sex-specific changes in schizophrenia are mainly observed in the dFNC State 4, and that they belong to the between model-order FNC (Figure 5). Interestingly, we observed opposite patterns of alterations for male and female cohorts in several significant differences. For instance, Figure 5 (R1, D4) shows significant differences in the dFNC State 4 (D4) in both male (C2) and female (C3) cohorts. However, while in the male cohort, the strength of dFNC in State 4 reduced in SZ (*t* value = −3.32), in the female cohort, the strength of FNC increased in the SZ cohort (*t* value = 3.65) compared with the control group.

One of the advantages of using msICA is that it allows us to see how the same region can contribute to different ICNs at different spatial scales and how the functional connectivity between these ICNs varies across different populations (Figure 5, R2).

**Figure 6.**
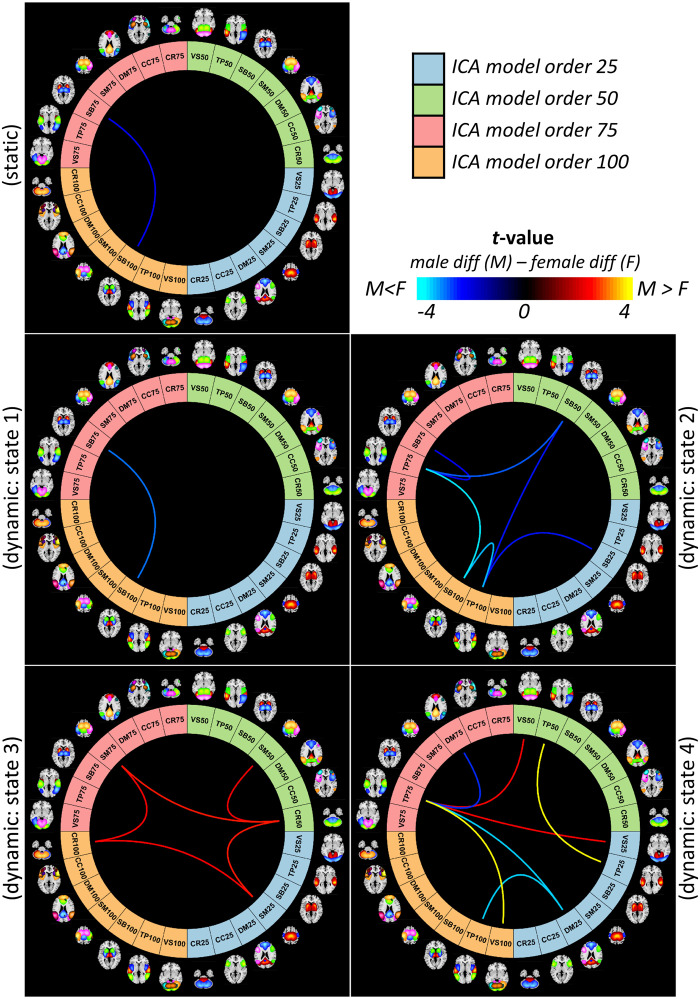
Sex-specific differences at the domain level across different spatial scales. Cognitive control (CC), default mode (DM), visual (VS), subcortical (SB), cerebellum (CR), somatomotor (SM), and temporal (TP). The number after the functional domain abbreviation is the model number; for example, DM25 means the default model domain from ICA model order 25.

Investigating sex-specific differences at the domain level across different spatial scales, we observed that sex-specific differences are more prominent in the dFNC compared with the sFNC. Significant differences exist within the subcortical domain between model order 75 and 100 (SB75-SB100) in sFNC and dFNC State 1 (Figure 6). State 2 shows sex-specific differences between the subcortical and temporal domains within and between several model orders (Figure 6). State 3, on the other hand, shows sex-specific differences between the cerebellar and somatomotor across different model orders (Figure 6). Like ICN-level comparison, dynamic State 4 reveals the most sex-specific differences, including the temporal, visual, and default mode domains.

While sex-specific differences show stronger effects of schizophrenia in males (male diff − female diff > 0) for functional domain connectivity associated with the SM and the VIS, we observe the opposite pattern for the rest of the differences. One exception is the within-temporal domain functional connectivity between model order 25 and 50 in the dFNC State 4.

For sex-specific changes at the domain level, we also evaluated the correlation with the PANSS total score. We observed strong correlations with *p* value < 0.05 in the male but not the female cohorts for four domain-level features. They include the following: (a and b) within the subcortical domain between model order 75 and 100 (SB75-SB100) in sFNC with the correlation values of 0.281/−0.117 (male/female) and dFNC State 1 with the correlation values of 0.173/−0.197 (male/female); (c) within the temporal domain between model order 25 and 50 (TP25-TP50) in dFNC State 4 with the correlation values of 0.277/−0.062 (male/female); and (d) between the visual domain model order 25 and the temporal domain model order 75 (VS25-TP75) in dFNC State 4 with the correlation values of −0.300/−0.024 (male/female). Among these, SB75-SB100 (sFNC) and VS25-TP75 (dFNC State 4) survived multiple comparison corrections (Supporting Information, Supplementary 3).

## DISCUSSION

### Multiscale Dynamic Interactions: Functional Segregation and Integration

Studying brain functional connectivity has improved our understanding of brain functions and the impact of brain disorders. However, currently, studying functional connectivity overwhelmingly disregards functional connectivity across multiple spatial scales. Existing studies, at best, apply data-driven approaches like ICA to study functional interactions at single model order but overlook the FNC within and between multiple spatial scales, while the majority of them uses fixed anatomical locations of the same size (e.g., a sphere with the same radius), which in addition to disregarding multiple spatial scales interaction, ignores differences in the spatial distribution of functional sources.

In this work, we present an approach to study multi-spatial-scale dynamic functional interactions, that is, dynamic changes that occur within and among different spatial scales, a topic that the field has overlooked. We leveraged the approach to study schizophrenia’s alterations and its sex-specific differences, which have also been understudied as most schizophrenia research focuses on only single spatial scale FC and non-sex-specific alterations of schizophrenia.

### Multiscale ICA

Our results show that multiscale ICA (msICA) using the Infomax algorithm is an effective, adaptive tool to identify functional sources at multiple spatial scales. Higher model order ICAs segregate the brain and functional domains into more ICNs with, in general, higher spatial granularity. For instance, the subcortical domain splits into more ICNs as the model order increases from 25 to 100. However, ICA does not enforce a limitation on each ICN’s spatial extent. Instead, ICA considers the multivariate association in the BOLD signal to segment the brain. As a result, msICA enables us to visualize functional segregation occurring at different levels of granularity across the brain. This is a desirable characteristic, as we know functional homogeneity varies across the brain and functional domains. The differences in parcellation granularity across functional domains provide additional information about the brain that needs to be studied in the future.

Furthermore, msICA captures the multifunctionality of brain regions and identifies distinct ICNs with high spatial overlap (for example, see the second row of Figure 5). Additional studies are needed to evaluate the neurophysiological basis to explain these variations. Furthermore, in this study, we focus on only four model orders of 25, 50, 75, and 100. Future studies should reduce the incremental steps and increase the range of model orders to effectively capture ICNs associated with a larger number of spatial levels of functional hierarchy (Iraji, Fu, et al., 2019). Recently, we used 1K-ICA, ICA with a model order of 1,000, to parcel the brain into very fine-grained functional sources (Iraji, Faghiri, et al., 2019). Furthermore, future studies should explore differences across the different back-reconstruction approaches (Erhardt et al., 2011). Developing techniques that simultaneously estimate ICNs for multiple model orders can improve the estimation of ICNs across multiple scales. Finally, considering the recent findings on spatial dynamics (Iraji, Deramus, et al., 2019; Iraji, Fu, et al., 2019; Iraji, Miller, et al., 2020), future works should also consider spatial dynamic functional segregations, as the spatial patterns of functional sources may vary over time.

### Multi-spatial-Scale dFNC

A window-based dFNC approach (Allen et al., 2014; Iraji, Faghiri, et al., 2020) was adopted to characterize the multi-spatial-scale dynamic functional interactions. To our best knowledge, this is the first study that looks at sFNC/dFNC across multiple mode orders. While we observe consistency and similarity of sFNC/dFNC both within and between model orders, there are also distinct differences in FNC patterns across FNC patterns. The differences are more distinguishable when there are larger differences in model orders, such as between model orders 25 and 100 (see, for example, Figure 4A, B, and E). This further highlights the importance of including a wider range of model orders in future studies.

Another important point is how we identify dFNC states. In this study, dFNC states were identified using all 127 ICNs; however, the brain may experience different states and/or temporal changes across different spatial scales. Higher functional hierarchy levels have less homogeneity and more dynamic behavior (Iraji, Fu, et al., 2019). Therefore, we expect more dynamism in the low-model-order ICAs. Future work should focus on variation in dFNC states and their timing across multiple model orders and differentiate between global and scale-specific dFNC states. It would also be interesting to extend the same multiscale idea to the number of clusters for the dFNC analysis. Different cohorts (e.g., male, female, control, and schizophrenia) may depict different characteristics at different scales.

Furthermore, similar to multi-spatial scales, brain functional segregation and integration can occur at different temporal scales and frequencies; thus, future studies can benefit from multitemporal scale functional interactions. Developing multi-spatiotemporal-scale analytic approaches and methodological frameworks to study functional sources is a crucial future avenue of investigation.

Finally, there is a rich repository of dynamic analytical approaches and secondary analysis that can be used to evaluate multi-spatial-scale brain dynamics (Chang & Glover, 2010; Kaboodvand et al., 2020; Karahanoğlu & Van De Ville, 2015; Lindquist et al., 2014; Miller et al., 2016; Yaesoubi et al., 2015).

### Schizophrenia

We further investigated the advantage of multi-spatial-scale analysis in schizophrenia and identifying sex-specific changes. Our results suggest disruptions in sFNC/dFNC across functional domains. Compared with controls, individuals with schizophrenia show reduced sFNC/dFNC within and between the visual, somatomotor, and temporal domains in both male and female cohorts (Figure 3). Previous studies that looked at differences between typical controls and individuals with schizophrenia also report hypoconnectivity across these functional domains using various approaches (Anticevic et al., 2014; Damaraju et al., 2014; Faghiri et al., 2021; Iraji, Deramus, et al., 2019; Iraji, Fu, et al., 2019; Kim et al., 2014; Shinn et al., 2015). Our study both confirms and extends previous findings. We identify significant differences between males and females in several FNC pairs, mainly showing larger schizophrenia-related changes in males than in female cohorts. This can be related to differences in clinical observations, including males presenting more severe overall symptoms, worse outcomes, and slower responses to treatment (Li et al., 2016). Greater SZ-related changes across these domains in males are also present at the domain level in dFNC State 4 within the temporal domain and between the temporal and visual domains (Figure 6).

Individuals with schizophrenia show hyperconnectivity of the subcortical domain with the visual, somatomotor, and temporal domains with notable exceptions in dFNC State 4. Unlike sFNC and dFNC in other states, dFNC State 4 has an overall negative association between the subcortical and the visual, somatomotor, and temporal domains (Figure 3). Certainly, temporal lobe anatomical and functional differences have been linked repeatedly to the expression of positive symptoms in schizophrenia (Barta et al., 1990; Shenton et al., 1992; Woodruff et al., 1997). We also observe different patterns of schizophrenia-related changes in male and female cohorts. Dynamic FNC State 4 also shows distinct sex-specific differences in the cerebellum domain connectivity patterns, where we observe the opposite pattern of alterations, particularly between the cerebellum and visual domain in the male and female cohorts (Figure 3). Cerebellar dysconnectivity patterns have been linked to negative symptom expression in schizophrenia (Brady et al., 2019).

The domain-level analysis suggests that major sex-dependent schizophrenia alterations at a large scale are mainly associated with the subcortical, cerebellar, temporal, and motor domains. Interestingly, most of the sex-specific differences were observed between model order and associated with dFNC states, highlighting the importance of multiscale dynamic analysis (Figure 4 and Figure 6).

In short, our findings are aligned with and extend previous schizophrenia studies, and we observed explicit sex-specific differences, particularly distinct dFNC patterns in State 4. These demand further investigations into the multi-spatial-scale dFNC and sex differences in SZ. However, these findings should be interpreted with caution and considering the limitations of the study.

First and foremost, considering the sex differences in the age of onset, future longitudinal studies should be used to study the role of the age of onset on the sex-specific differences in schizophrenia and evaluate the relationship between time and sex-specific differences over time. Long-term effects of medication and treatment, which cannot be accounted for (Moncrieff & Leo, 2010), might impact observed differences. Including unaffected close relatives sharing genetic risk, that is, at-high-risk unmedicated subjects, can help us better understand changes in brain function (Pearlson & Stevens, 2020). The unbalanced number of samples between groups is another limitation of the studies. While we control for sex difference and the null distribution was created with the same female to male ratio, future studies should focus on datasets with larger numbers of females.

### Biomarkers and the Importance of Sex-Specific Characteristics

According to the NIH Biomarkers Definitions Working Group, a biomarker is defined as “a characteristic that is objectively measured and evaluated as an indicator of normal biological processes, pathogenic processes, or pharmacologic responses to a therapeutic intervention” (Biomarkers Definitions Working Group, 2001). As such, a diagnostic biomarker is defined as a characteristic or feature capable of detecting or confirming the presence of a (subtype of) disease or condition of interest (Califf, 2018). At the same time, numerous studies have observed sex differences in schizophrenia, including in the age of onset, in experiencing negative and positive symptoms, and in response to treatments (Nawka et al., 2013; Li et al., 2016; Seeman, 2019). Therefore, the biomarkers for schizophrenia might be somewhat different for males and females.

This study’s premise is that sex influences differences in schizophrenia characteristics, and we introduce a dynamic multi-spatial-scale framework to obtain candidates for sex-specific biomarkers from rsfMRI data. We observed significant sex-specific differences across several functional domains, including in subcortical and temporal connectivity patterns, which also significantly correlate with symptom scores in males but not females. Interestingly, the affected functional domains have been frequently reported to be altered in SZ and touted as having potential to serve as identifying biomarkers. Our results suggest that sex-specific functional connectivity changes might be related to schizophrenia symptoms and underlying causes and emphasize the importance of carefully incorporating sex in the development of diagnostic/predictive/monitoring biomarkers. While sex and schizophrenia can be identified straightforwardly, there has been very little work looking at sex and schizophrenia differences across different spatial scales in resting fMRI data. The incorporation of sex as a biological variable within the context of schizophrenia may help shed new light on the neurobiological mechanisms of schizophrenia. Future studies should leverage these findings and incorporate sex into feature selection and classification algorithms to identify a set of sensitive schizophrenia-related features for use in updating nosological categories and building diagnostic and predictive models.

## CONCLUSION

Brain dynamic functional interaction can occur at different spatial scales, which has been underappreciated. In this work, we propose an approach that uses multiscale ICA and dFNC to study brain function at different spatial scales. This results in a more comprehensive map of functional interactions across the brain. This not only solves the limitation of using fixed anatomical locations but also eliminates the need for model-order selection in ICA analysis. Therefore, we propose multiscale ICA (msICA), and future multi-spatial-scale methods should be broadly applied in future studies. Going forward, we can further improve the proposed approach by incorporating explicit spatial dynamics and multi-temporal-scale features of functional sources. We leverage the proposed approach to study male/female common and unique aspects of sFNC/dFNC in schizophrenia, which have not been investigated despite previous reports on sex differences on the prevalence, symptoms, and responses to treatment. The majority of sex-specific differences occur in between-model-order and associated with dFNC states, further highlighting the benefits of our proposed approach. Future studies are needed to validate our findings and evaluate the further benefits of multiscale analysis.

## SUPPORTING INFORMATION

Supporting information for this article is available at https://doi.org/10.1162/netn_a_00196.

## AUTHOR CONTRIBUTIONS

Armin Iraji: Conceptualization; Formal analysis; Investigation; Methodology; Software; Visualization; Writing – original draft; Writing – review & editing. Ashkan Faghiri: Formal analysis; Visualization; Writing – review & editing. Zening Fu: Data curation; Writing – review & editing. Srinivas Rachakonda: Software. Peter Kochunov: Resources; Writing – review & editing. Aysenil Belger: Resources. Judy Ford: Resources; Writing – review & editing. Sarah McEwen: Resources. Daniel Mathalon: Resources. Bryon Mueller: Resources. Godfrey Pearlson: Resources; Writing – review & editing. Steven G. Potkin: Resources. Adrian Preda: Resources. Jessica Turner: Resources; Writing – review & editing. Theodorus Van Erp: Resources. Vince Calhoun: Conceptualization; Funding acquisition; Investigation; Methodology; Project administration; Resources; Software; Writing – review & editing.

## FUNDING INFORMATION

Vince Calhoun, Foundation for the National Institutes of Health (https://dx.doi.org/10.13039/100000009), Award ID: 1U24RR021992. Vince Calhoun, Foundation for the National Institutes of Health (https://dx.doi.org/10.13039/100000009), Award ID: 1U24RR025736. Vince Calhoun, Foundation for the National Institutes of Health (https://dx.doi.org/10.13039/100000009), Award ID: R01EB020407. Vince Calhoun, Foundation for the National Institutes of Health (https://dx.doi.org/10.13039/100000009), Award ID: R01MH118695. Vince Calhoun, Foundation for the National Institutes of Health (https://dx.doi.org/10.13039/100000009), Award ID: P20GM103472. Judy Ford, Foundation for the National Institutes of Health (https://dx.doi.org/10.13039/100000009), Award ID: R01MH058262. Judy Ford, U.S. Department of Veterans Affairs (https://dx.doi.org/10.13039/100000738), Award ID: I01CX0004971.

## TECHNICAL TERMS

Functional source: A spatial locality with a temporally synchronized pattern. All voxels within a functional source have similar temporal patterns.
Intrinsic connectivity network (ICN): An estimation of a functional source obtained using ICA and typically referring to the components deemed non-artifactual.
Independent component analysis (ICA): A multivariate technique that decomposes data into a set of maximally independent components.
Functional network connectivity (FNC): The temporal dependence between ICN time courses.
Static functional network connectivity (sFNC): FNC calculated using the entire length of the ICN time course; sFNC represents overall or average FNC.
Dynamic functional network connectivity (dFNC): FNC calculated using a subset of ICN time courses. Using dFNC, we can capture and evaluate how FNCs between ICNs evolve over time.
