# Supplementary material for: Multi-spatial-scale dynamic interactions between functional sources reveal sex-specific changes in schizophrenia
Source: Netw Neurosci. 2022 Jun 1;6(2):357–81. doi: 10.1162/netn_a_00196 (PMC9208002; doi:10.1162/netn_a_00196)
Supplement: Supplementary file 1 [file netn-06-357-s001.zip › icatb_gica_html_report100.pdf]

Table of Contents

 1
Group ICA Parameters 1
ICASSO Plots 2
Mean Components 5
Spectral Summary 104
Temporal Stats On Beta Weights 106
Kurtosis of timecourses and spatial maps 106
FNC correlations 110
FNC metrics of component spatial maps 111

Group ICA Parameters

...

Number of Subjects : 856

Number of Sessions : 1

Number of Independent Components : 100

ICA Algorithm : Infomax

Number Of Scans/Timepoints : 157

Mask File : mask_common

Data Pre-processing Type : Variance Normalization

PCA Type : Standard

Group PCA Type : Subject Specific

Group ICA Type : Spatial

Back Reconstruction Type : Spatial-temporal Regression

Scaling Components : Z-scores

Stability analysis type : ICASSO

Group analysis mode: Parallel

Anatomical file: /trdapps/linux-x86_64/matlab/toolboxes/GroupICATv4.0b/icatb/icatb_templates/ch2bet.nii
Slice Plane: Axial

Image values: Positive

Convert to Z-scores: yes

Threshold: 1.96

...

ICASSO Plots

Warning: Creates overwhelming number of lines
Warning: Tries to change the limit...
Warning: New limit =0.99175

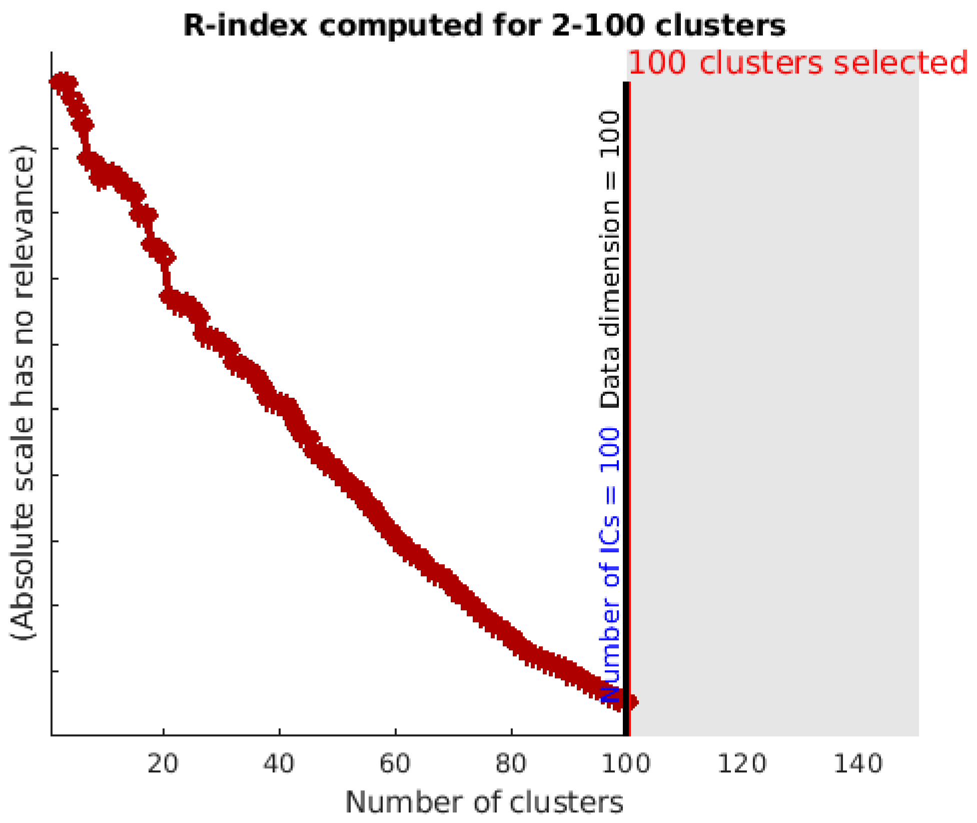

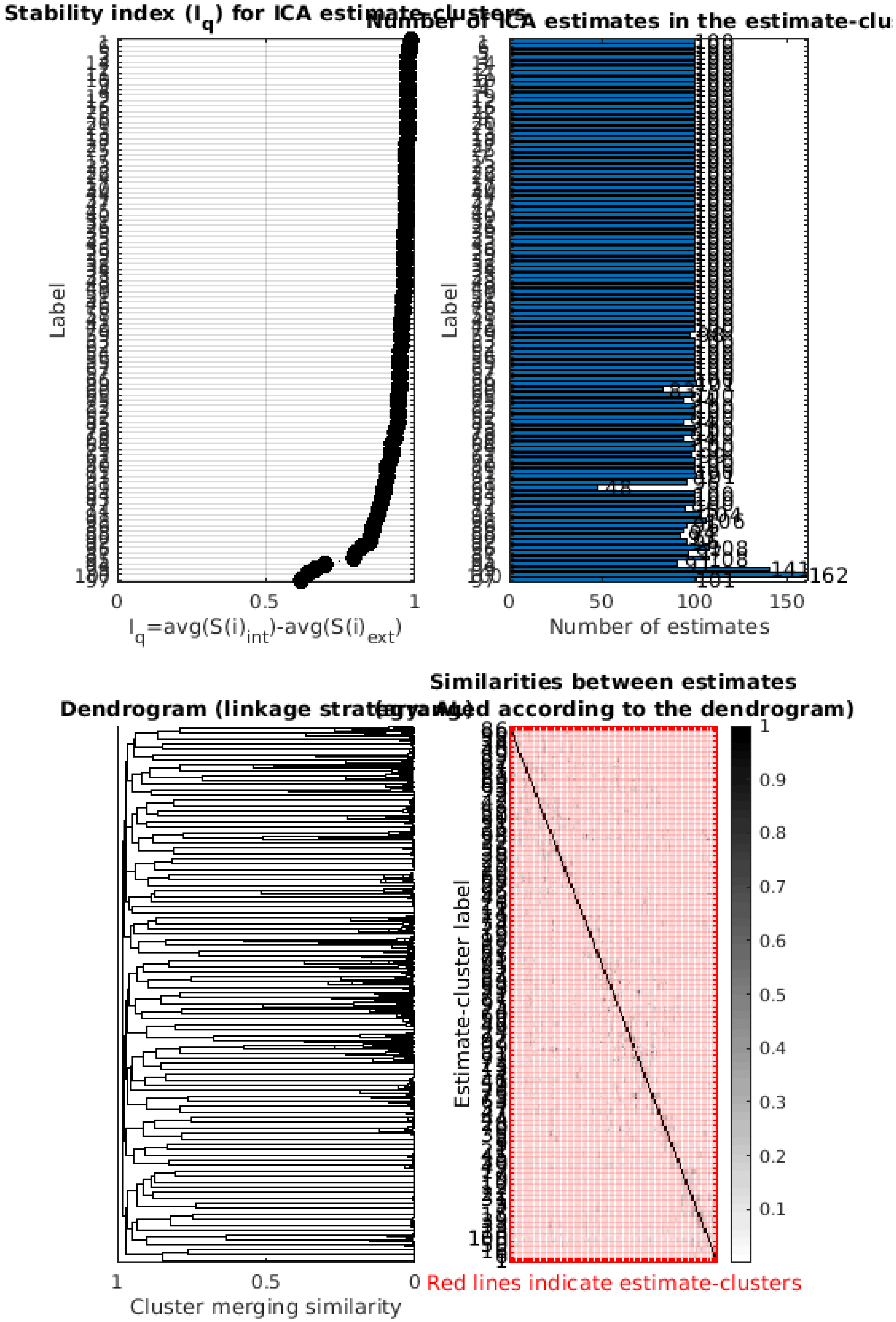
Stability index (I_q) for ICA estimate-clusters
Label
0
0.5
1
I_q=avg(S(i)$_{int}$)-avg(S(i)$_{ext}$)
Number of ICA estimates in the estimate-clu
Label
0
50
100
150
Number of estimates
48
141
162
Dendrogram (linkage strategy: AL)
1
0.5
0
Cluster merging similarity
Similarities between estimates
(arranged according to the dendrogram)
Estimate-cluster label
1
0.9
0.8
0.7
0.6
0.5
0.4
0.3
0.2
0.1
Red lines indicate estimate-clusters

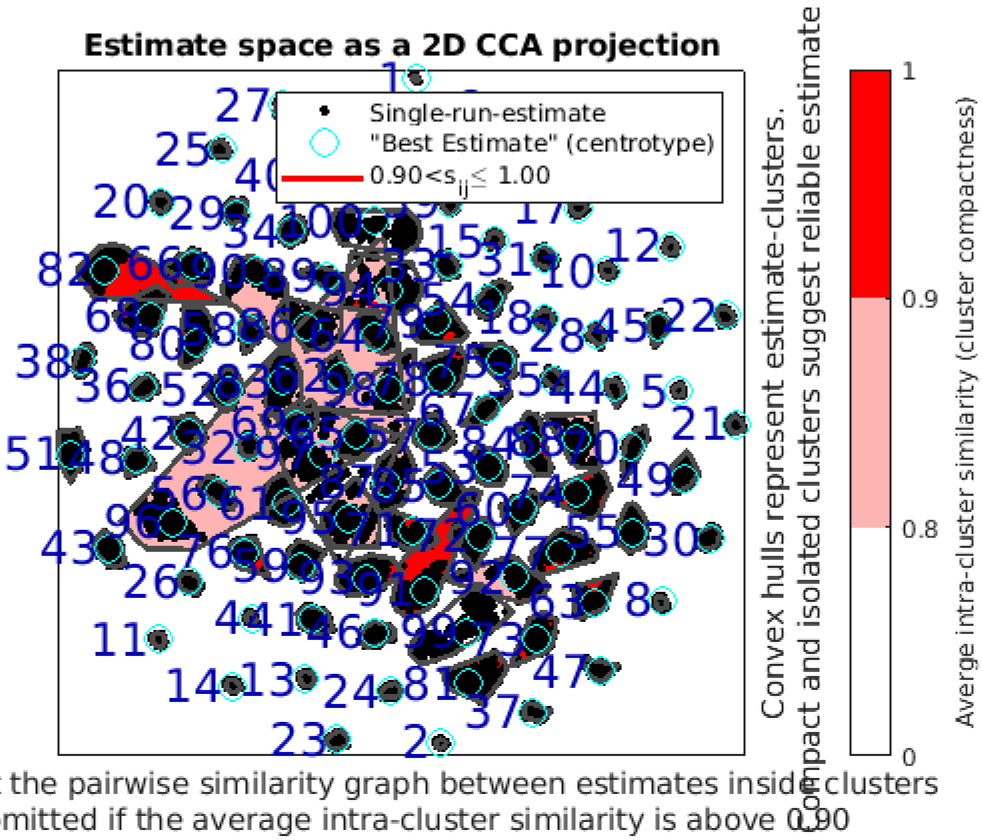

Estimate space as a 2D CCA projection
Single-run-estimate
"Best Estimate" (centrotype)
0.90<s_ij≤ 1.00
Convex hulls represent estimate-clusters.
Compact and isolated clusters suggest reliable estimate
Averge intra-cluster similarity (cluster compactness)
1
0.9
0.8
0
e that the pairwise similarity graph between estimates inside clusters
is omitted if the average intra-cluster similarity is above 0.90

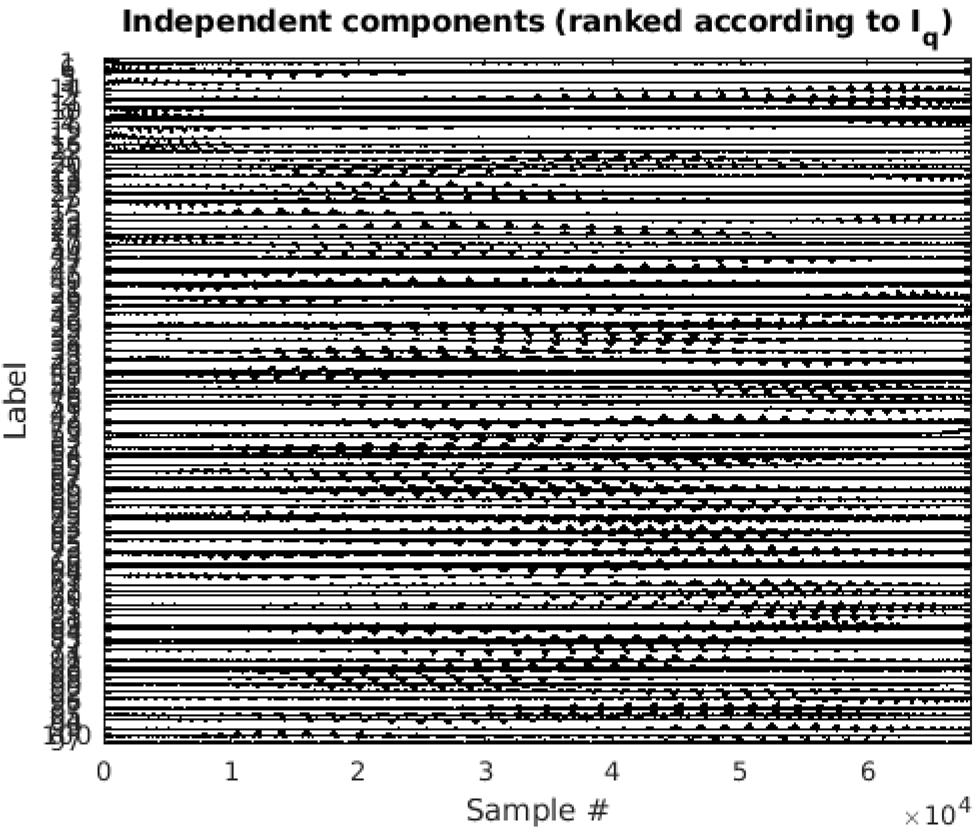

Independent components (ranked according to I_q)
Label
0
1
2
3
4
5
6
Sample #
×10^4

Mean Components

Mean across all subjects and sessions is computed for each component

- **a) Timecourse** - Mean timecourse is converted to z-scores.
- **b) Spectra** - Timecourses spectra is computed for each data-set and averaged across sessions. Mean and standard error of mean is shown in the figure.
- **c) Montage** - Axial slices are shown.
- **d) Ortho slices** - Ortho plot is shown for the peak voxel and coordinates are reported.

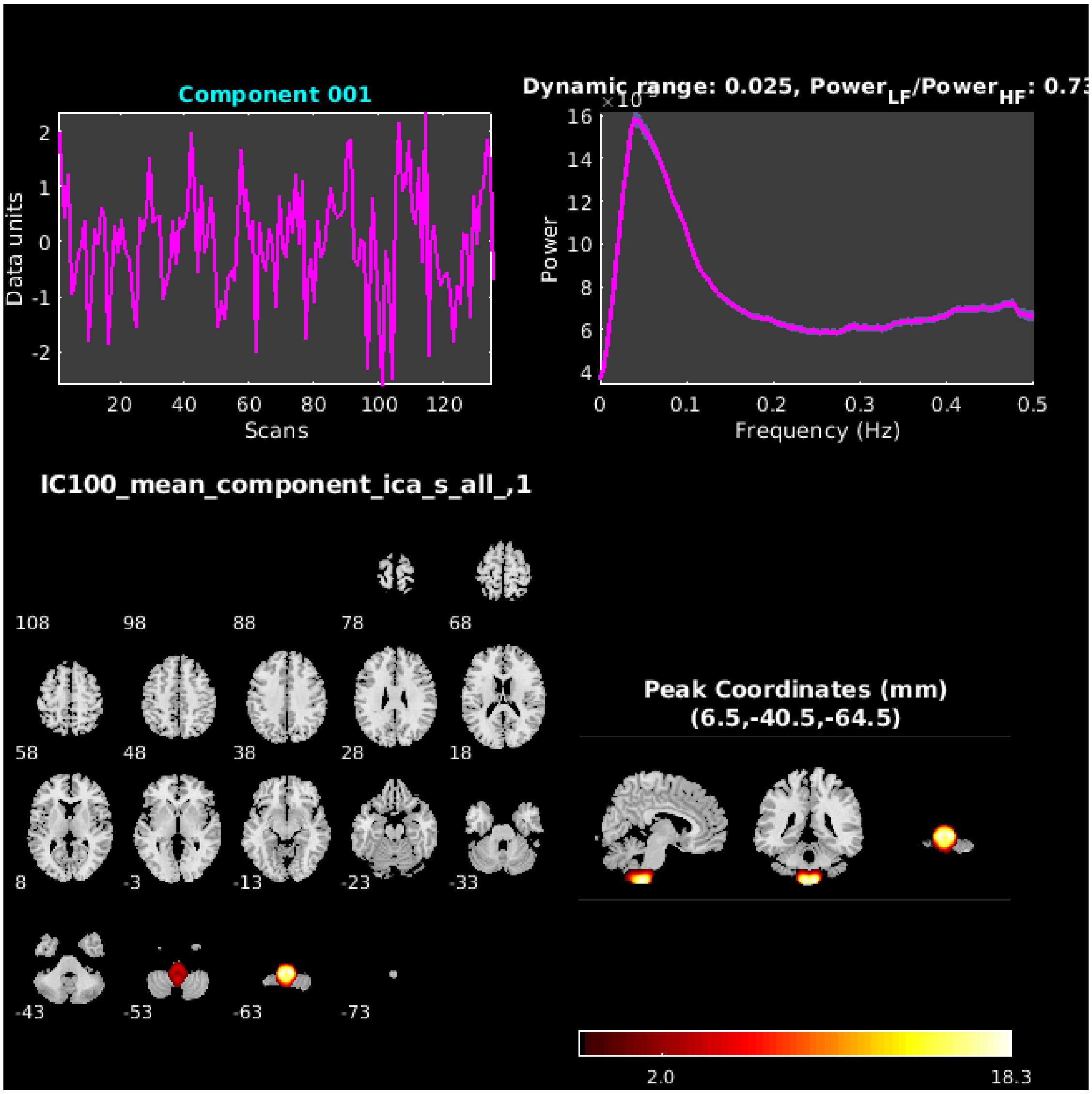

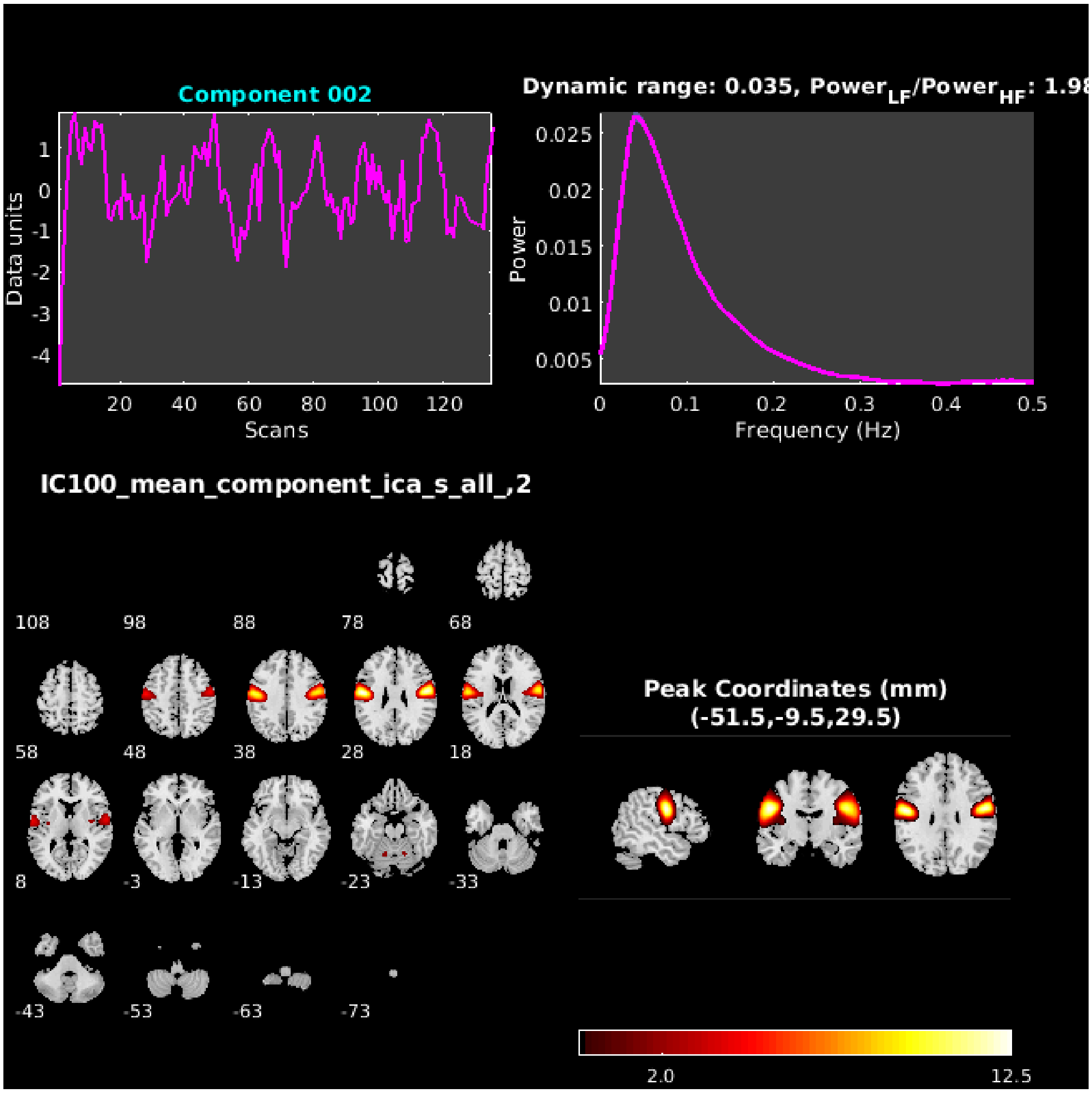

Component 002
Dynamic range: 0.035, Power_LF/Power_HF: 1.98
Data units
Scans
Power
Frequency (Hz)
IC100_mean_component_ica_s_all_,2
108 98 88 78 68
58 48 38 28 18
8 -3 -13 -23 -33
-43 -53 -63 -73
Peak Coordinates (mm)
(-51.5,-9.5,29.5)
2.0
12.5

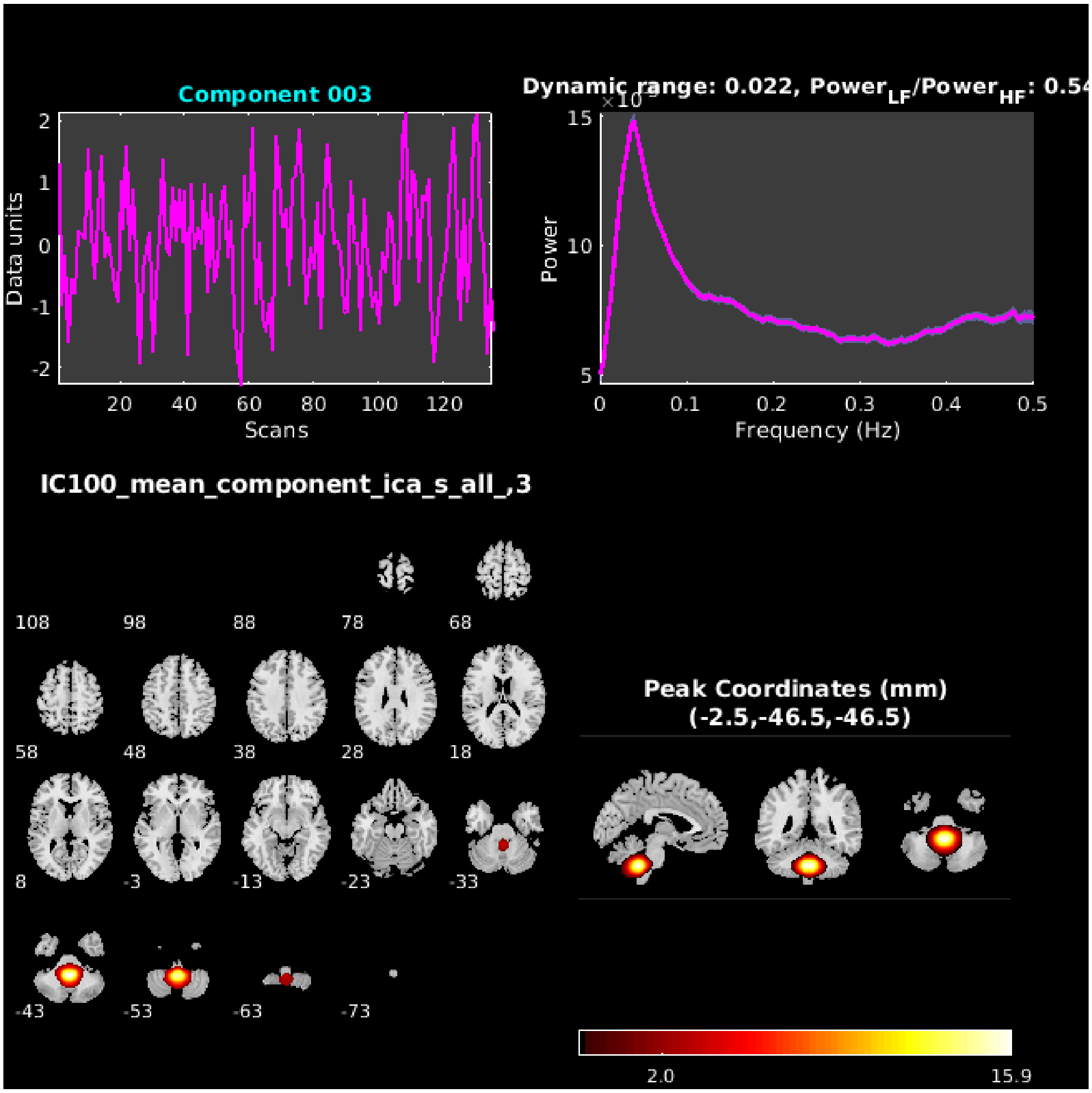

Component 003
Dynamic range: 0.022, Power$_{LF}$/Power$_{HF}$: 0.54
Data units
Scans
Power
Frequency (Hz)
IC100_mean_component_ica_s_all_,3
Peak Coordinates (mm)
(-2.5,-46.5,-46.5)
2.0
15.9

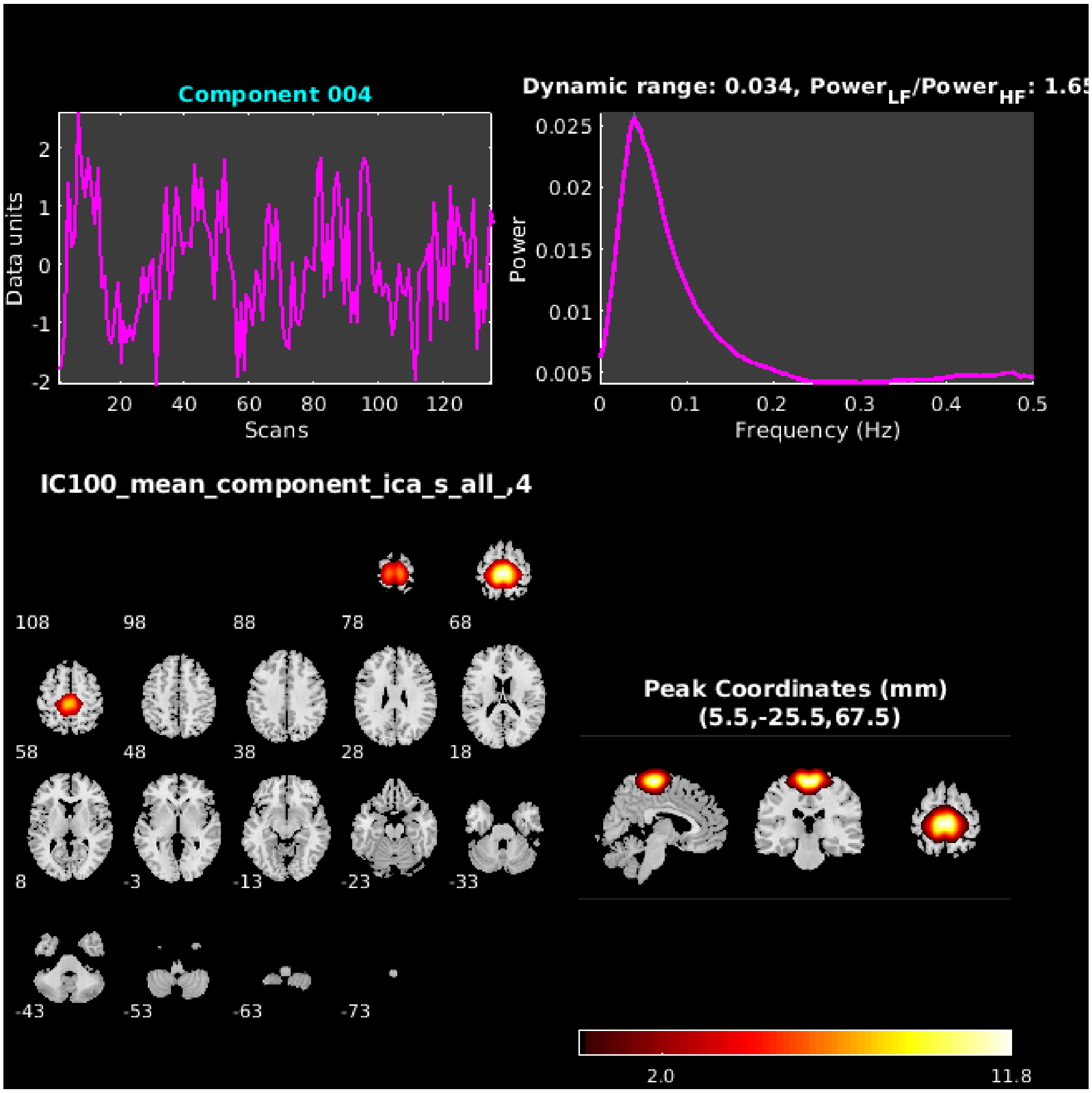

Component 004
Dynamic range: 0.034, Power$_{LF}$/Power$_{HF}$: 1.65
Data units
Scans
Power
Frequency (Hz)
IC100_mean_component_ica_s_all_,4
Peak Coordinates (mm)
(5.5,-25.5,67.5)
2.0
11.8

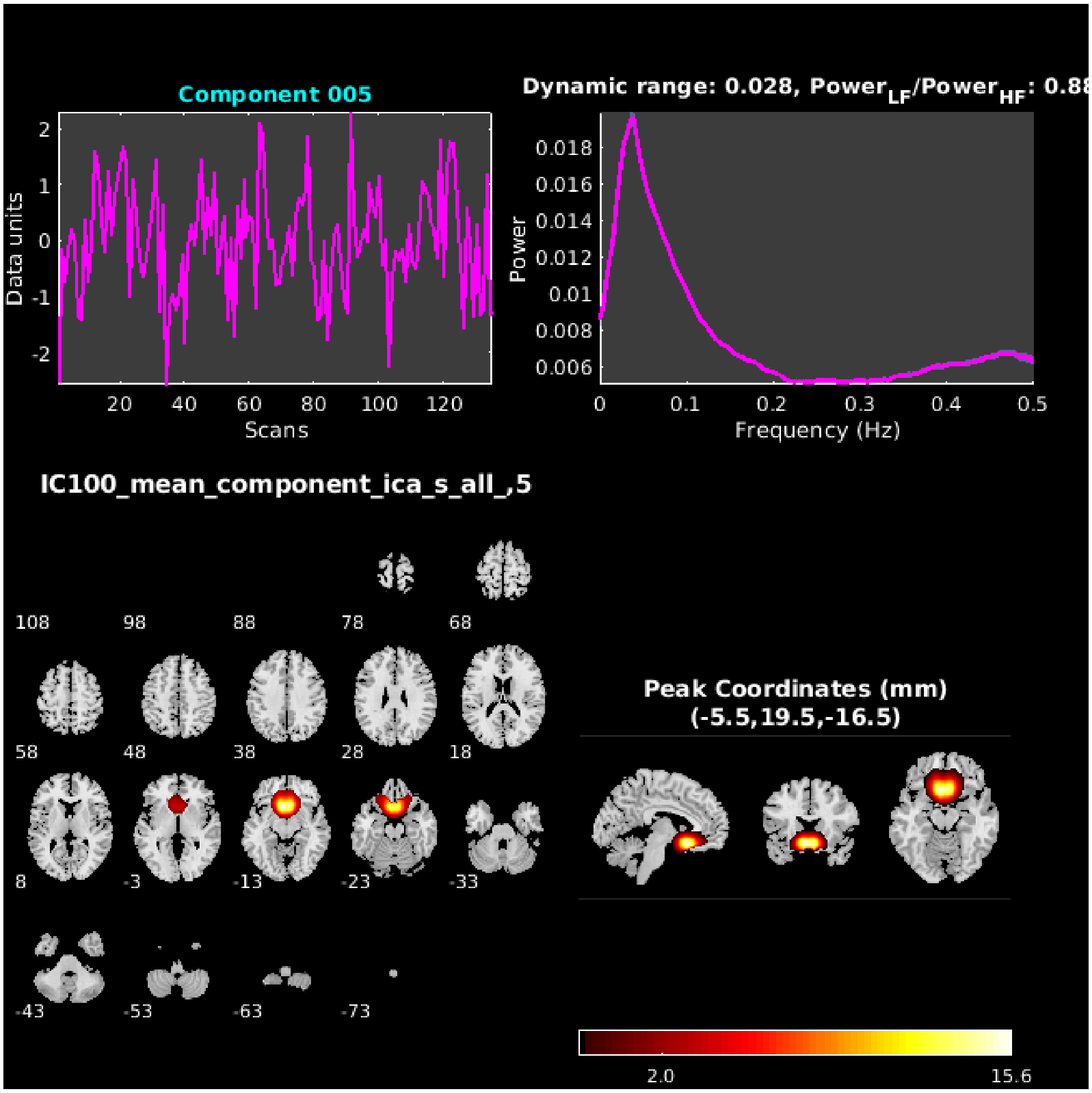

Component 005
Data units
Scans
Dynamic range: 0.028, Power$_{LF}$/Power$_{HF}$: 0.88
Power
Frequency (Hz)
IC100_mean_component_ica_s_all_,5
Peak Coordinates (mm)
(-5.5,19.5,-16.5)
2.0
15.6

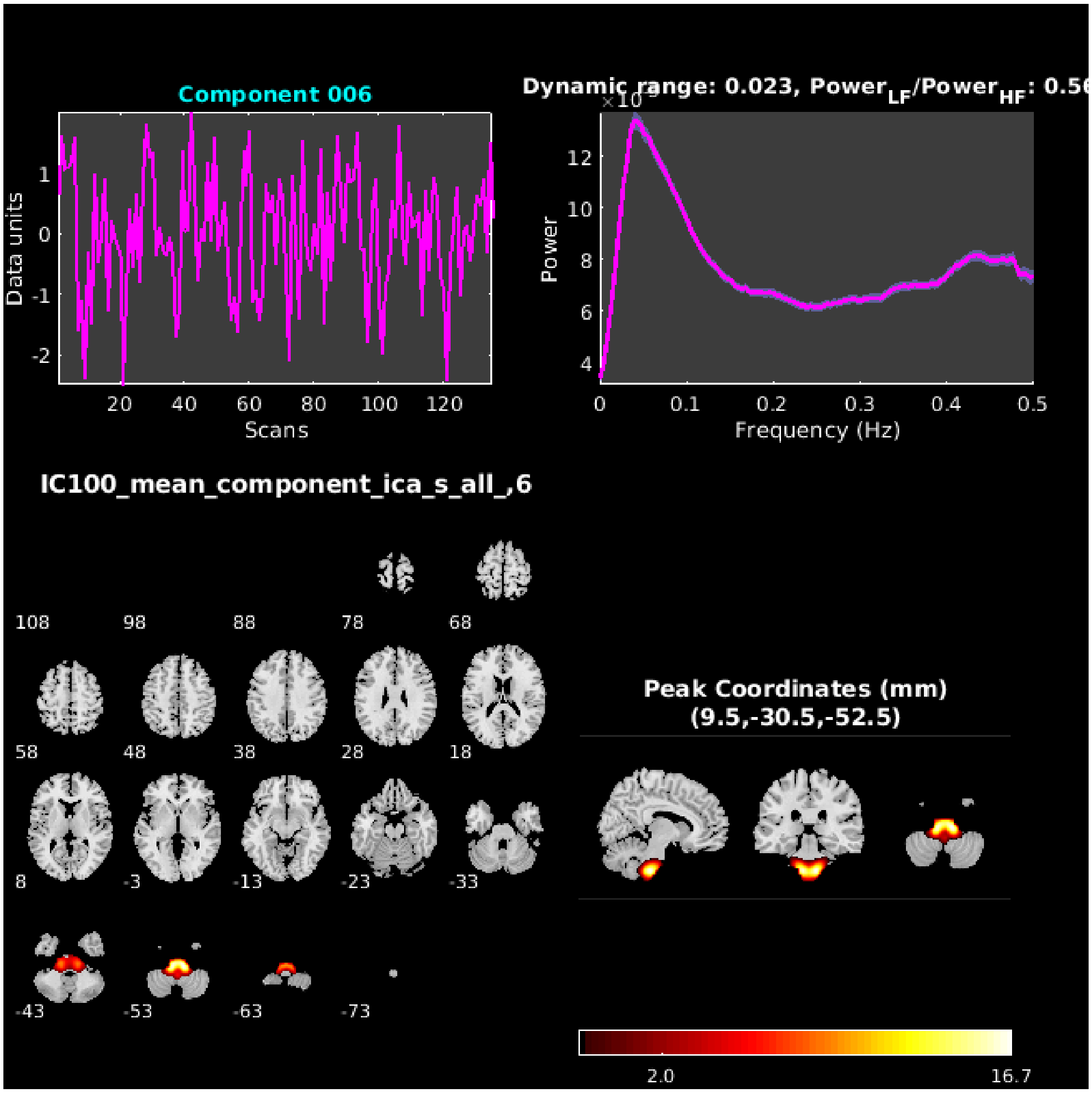
Component 006
Dynamic range: 0.023, Power_LF/Power_HF: 0.56
Data units
Scans
Power
Frequency (Hz)
IC100_mean_component_ica_s_all_,6
Peak Coordinates (mm)
(9.5,-30.5,-52.5)
2.0
16.7

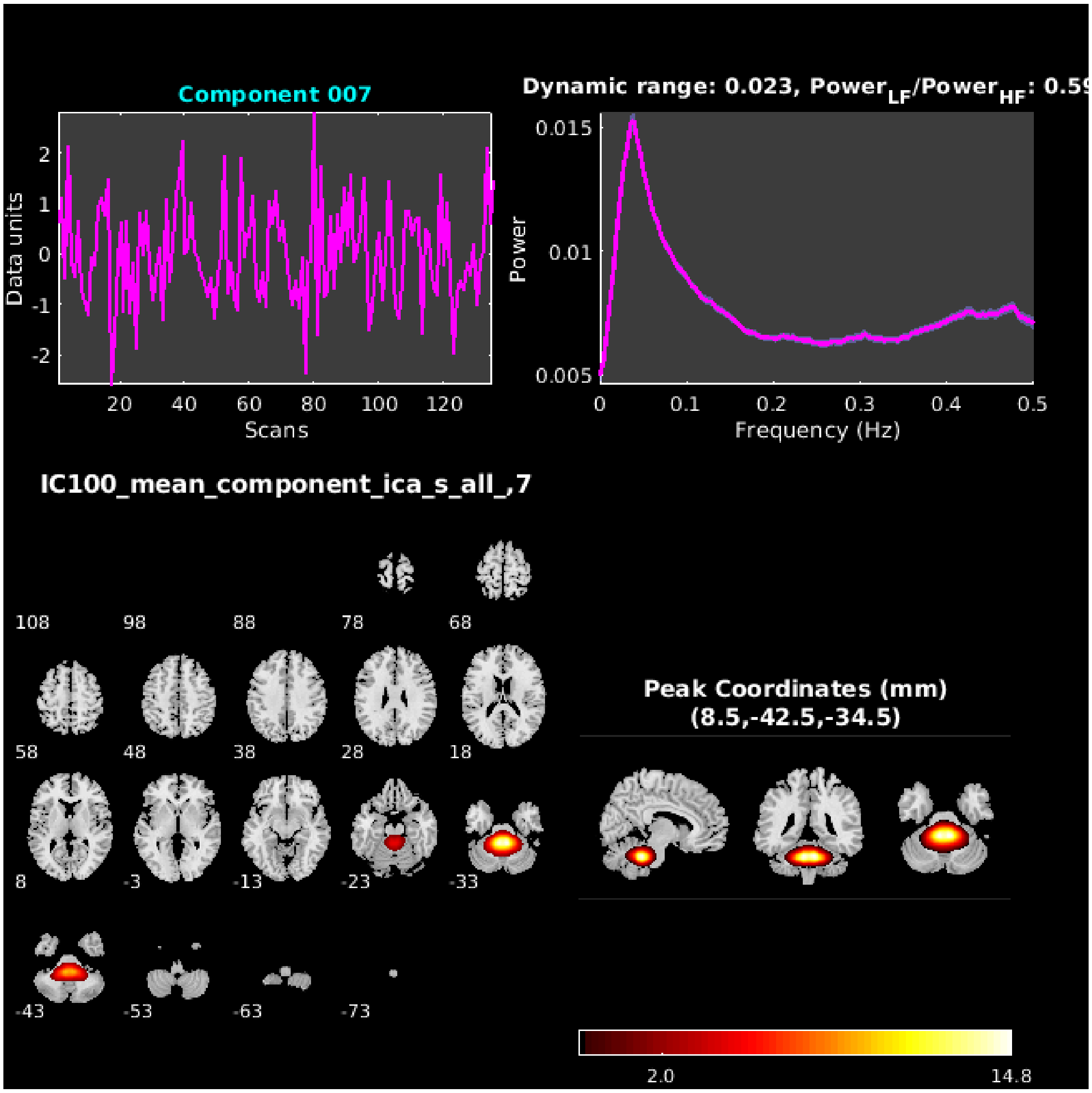
Component 007
Dynamic range: 0.023, Power$_{LF}$/Power$_{HF}$: 0.59
Data units
Scans
Power
Frequency (Hz)
IC100_mean_component_ica_s_all_,7
Peak Coordinates (mm)
(8.5,-42.5,-34.5)
2.0
14.8

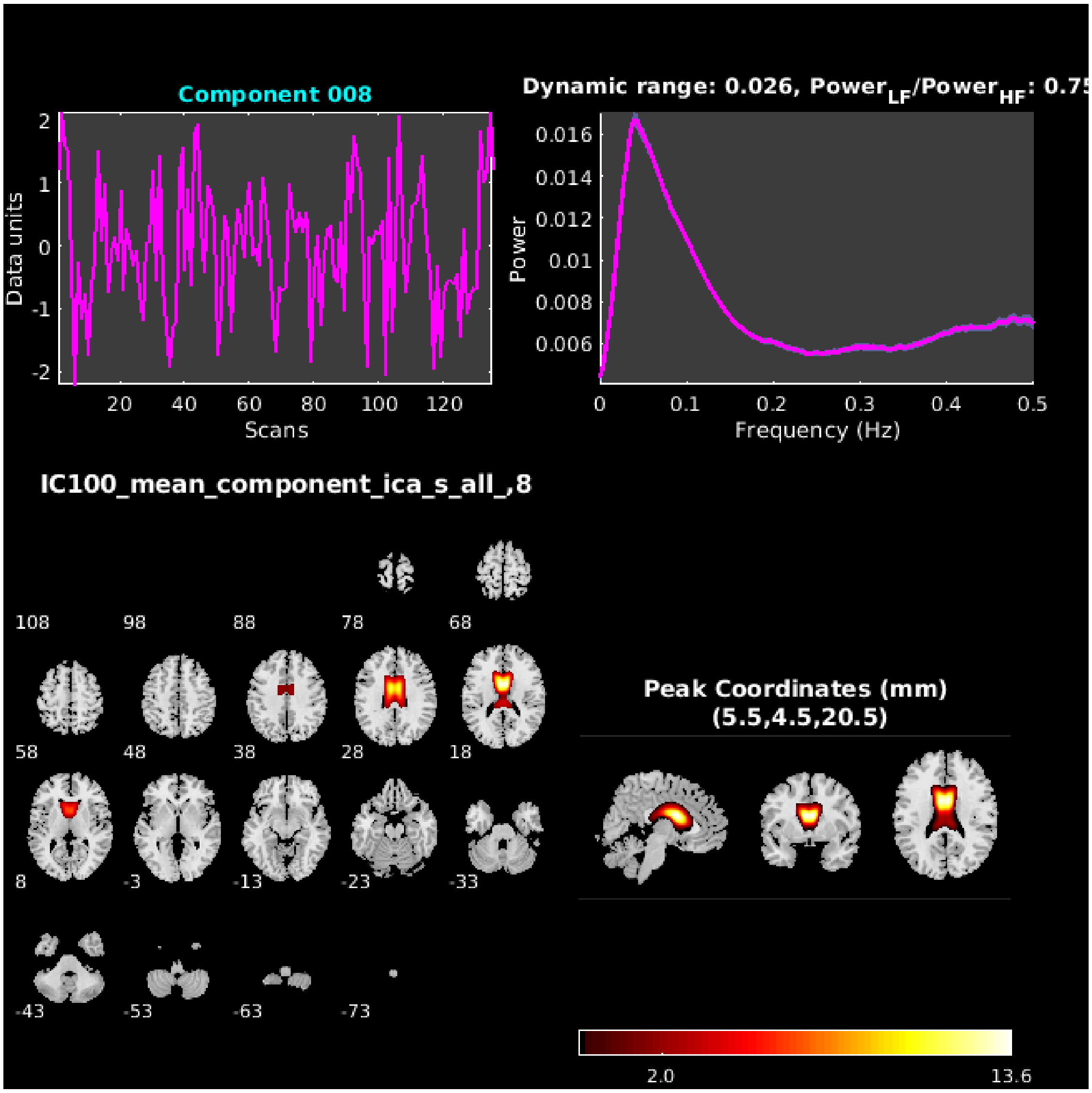
Component 008
Dynamic range: 0.026, Power$_{LF}$/Power$_{HF}$: 0.75
Data units
Scans
Power
Frequency (Hz)
IC100_mean_component_ica_s_all_,8
Peak Coordinates (mm)
(5.5,4.5,20.5)
2.0
13.6

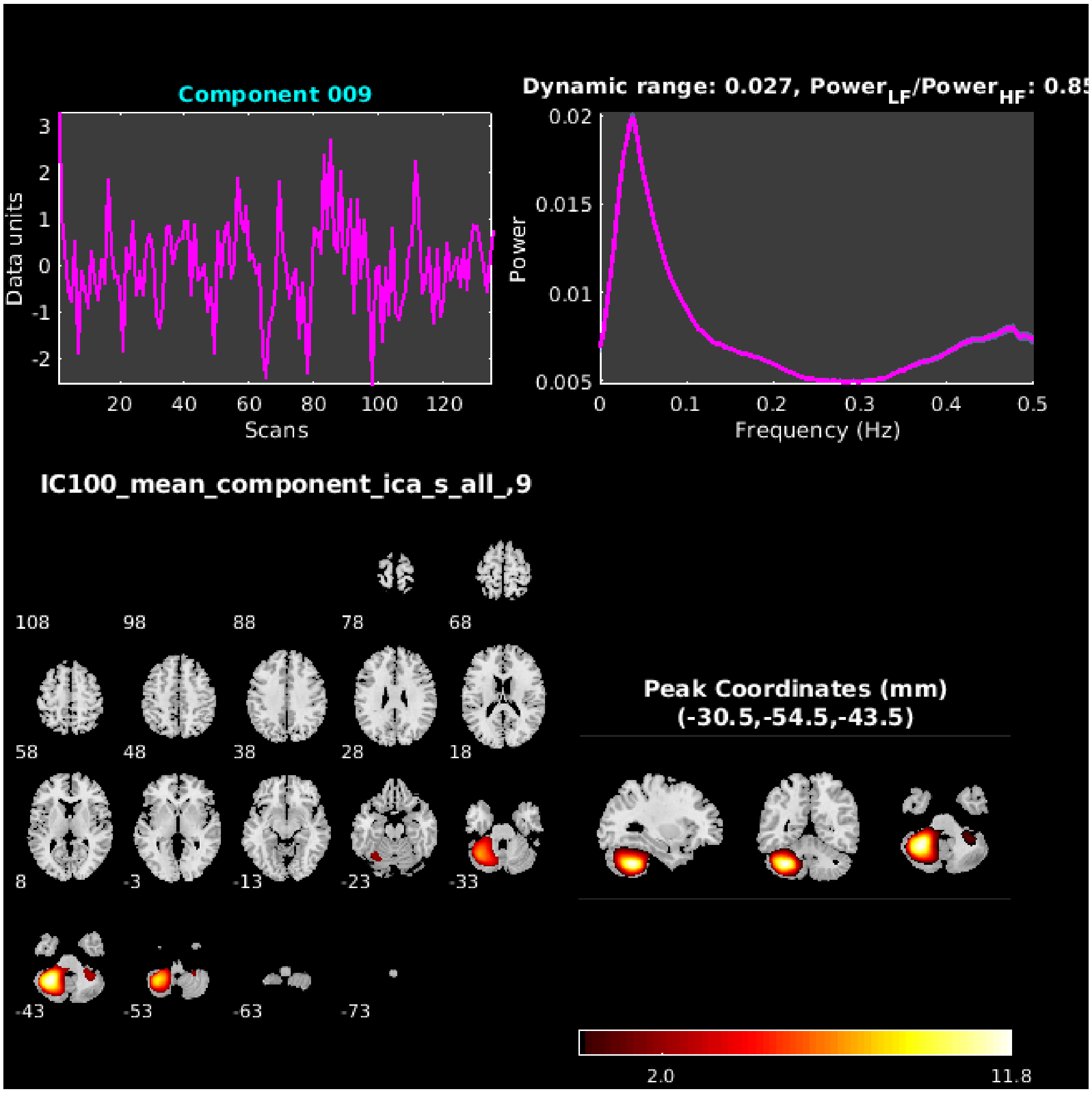

Component 009
Data units
Scans
Dynamic range: 0.027, Power$_{LF}$/Power$_{HF}$: 0.85
Power
Frequency (Hz)
IC100_mean_component_ica_s_all_,9
Peak Coordinates (mm)
(-30.5,-54.5,-43.5)
2.0
11.8

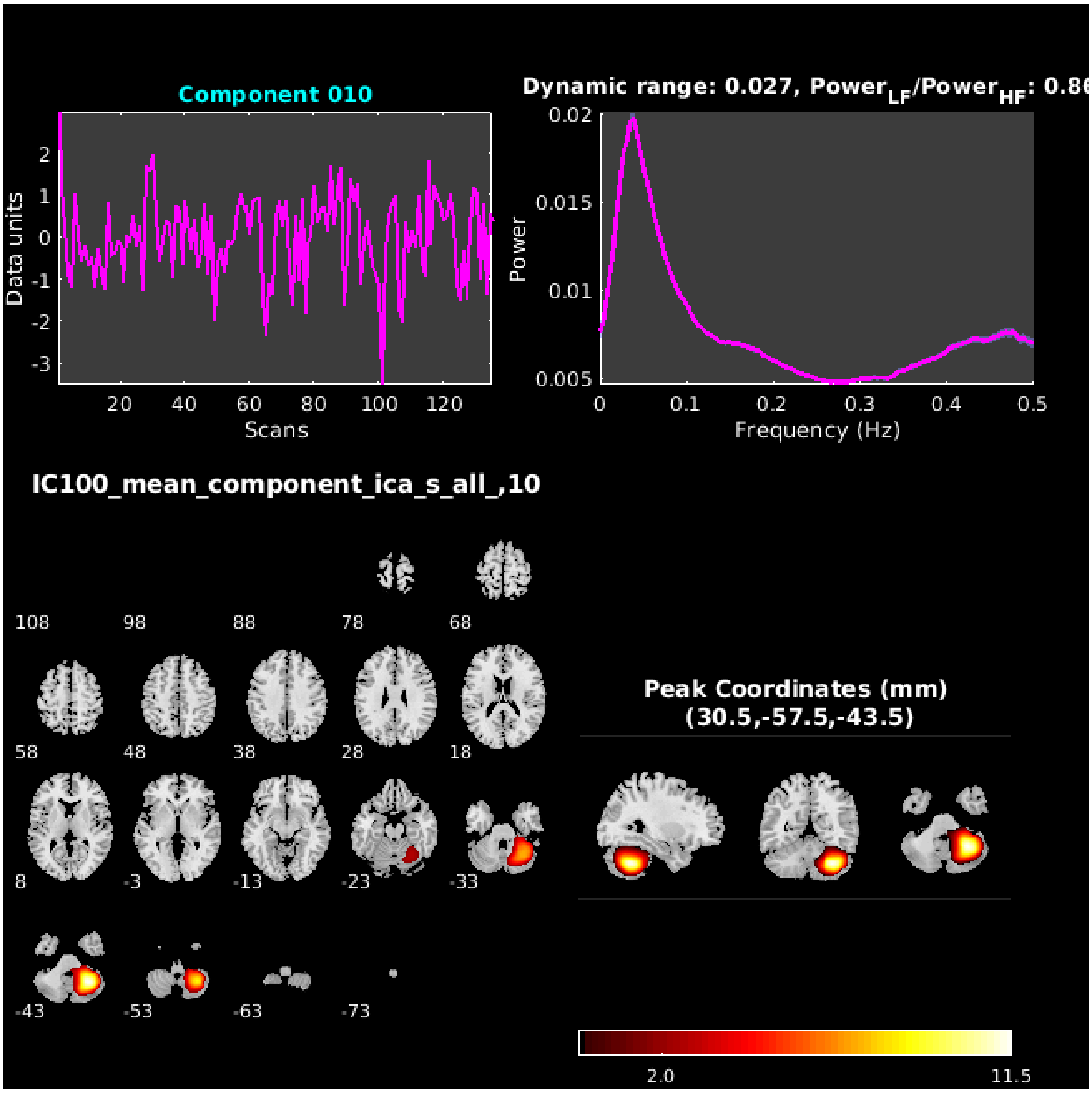
Component 010
Dynamic range: 0.027, Power_LF/Power_HF: 0.8
Data units
Scans
Power
Frequency (Hz)
IC100_mean_component_ica_s_all_,10
Peak Coordinates (mm)
(30.5,-57.5,-43.5)
2.0
11.5

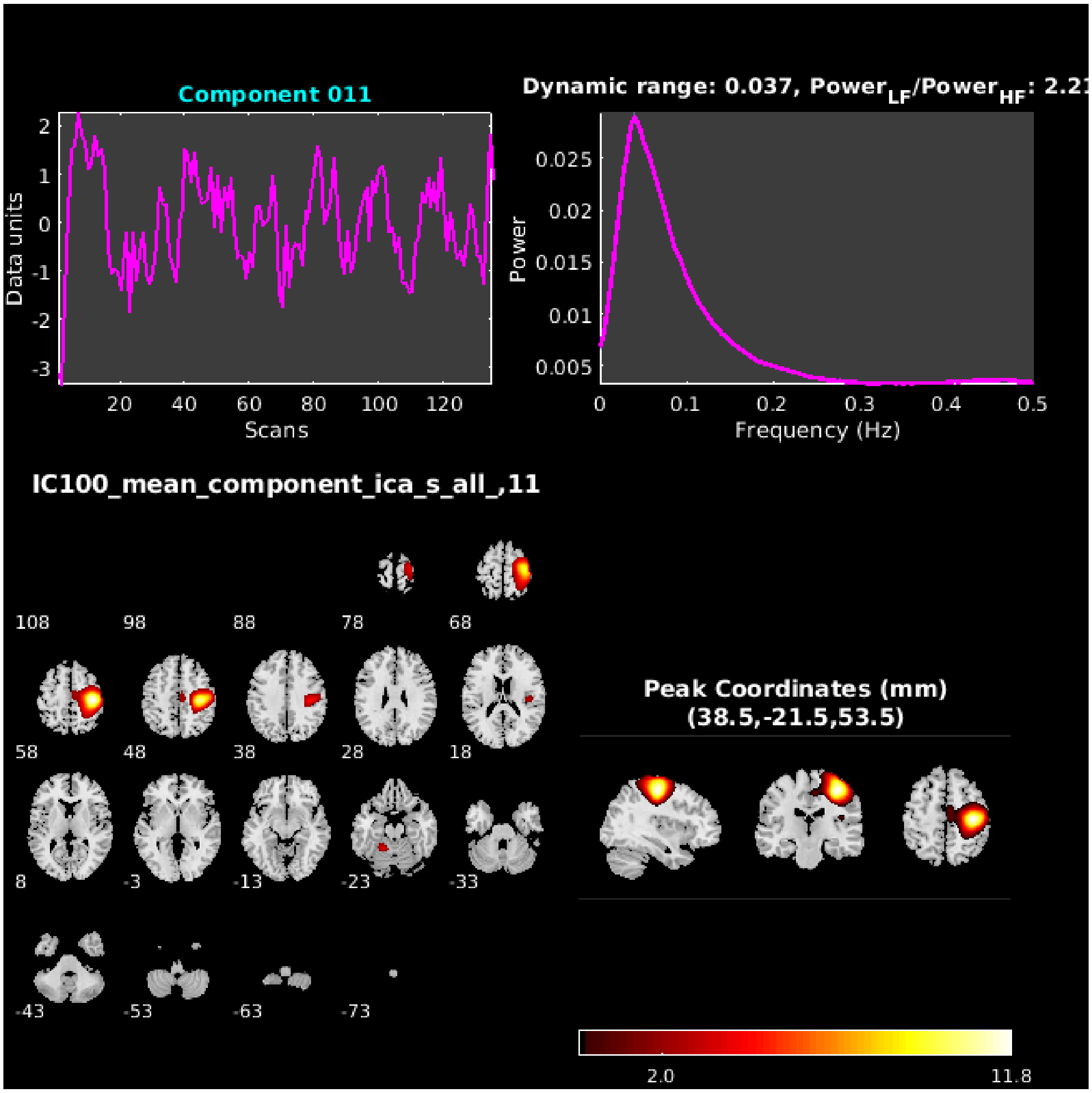
Component 011
Dynamic range: 0.037, Power$_{LF}$/Power$_{HF}$: 2.21
Data units
Scans
Power
Frequency (Hz)
IC100_mean_component_ica_s_all_,11
Peak Coordinates (mm)
(38.5,-21.5,53.5)
2.0
11.8

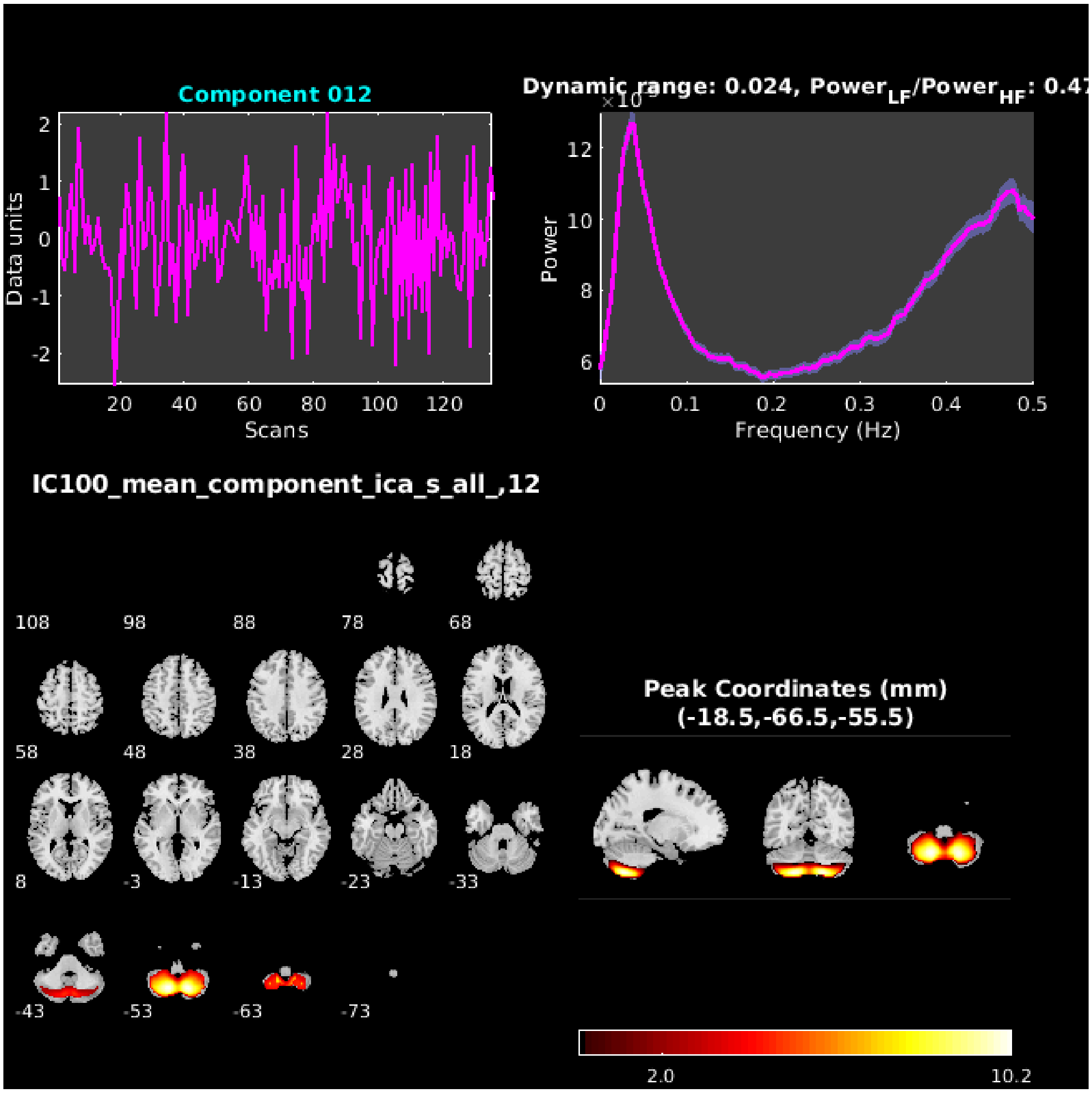
Component 012
Data units
Scans
Dynamic range: 0.024, Power$_{LF}$/Power$_{HF}$: 0.47
Power
Frequency (Hz)
IC100_mean_component_ica_s_all_,12
Peak Coordinates (mm)
(-18.5,-66.5,-55.5)
2.0
10.2

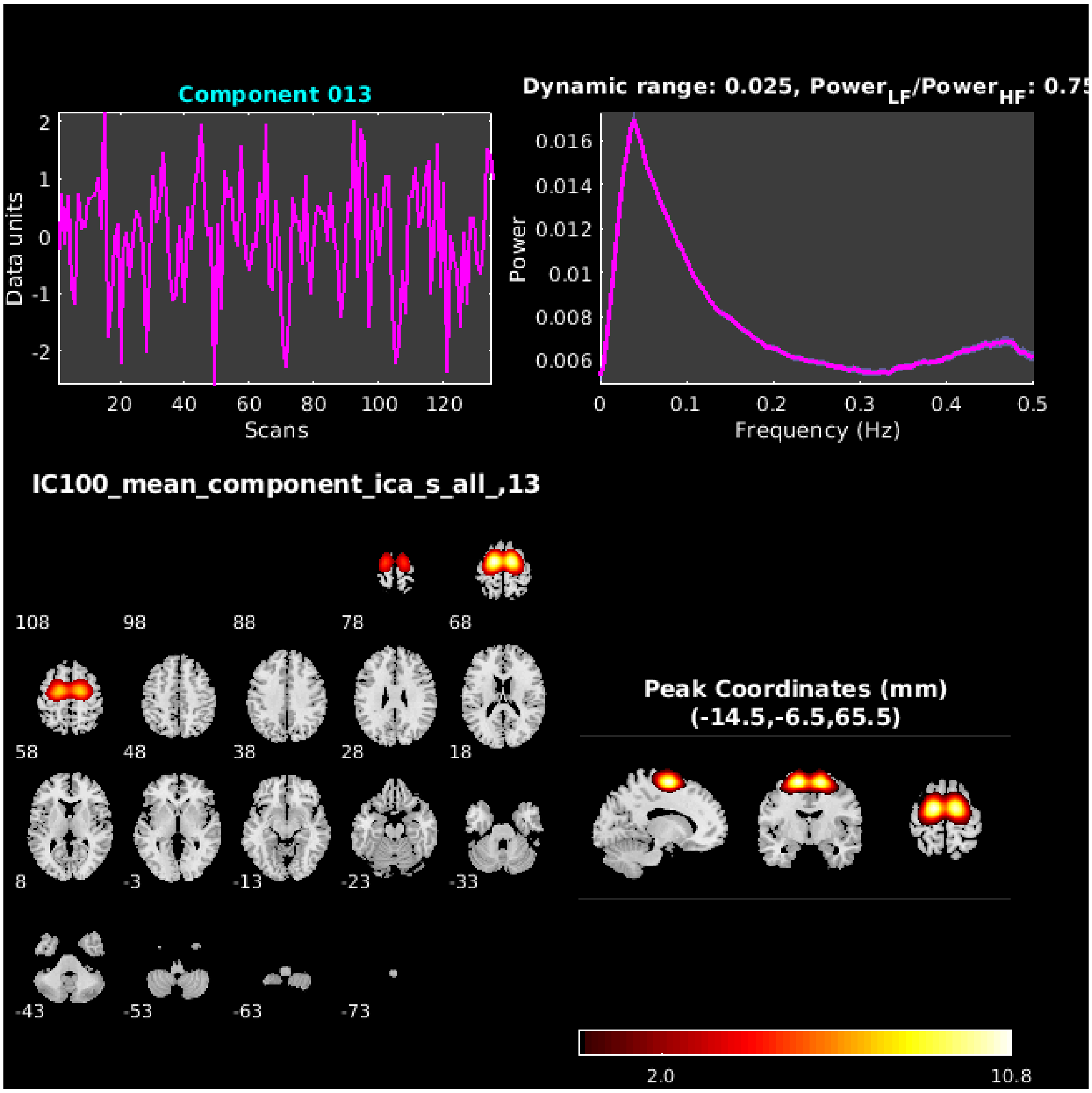

Component 013
Dynamic range: 0.025, Power$_{LF}$/Power$_{HF}$: 0.75
Data units
Scans
Power
Frequency (Hz)
IC100_mean_component_ica_s_all_,13
Peak Coordinates (mm)
(-14.5,-6.5,65.5)
2.0
10.8

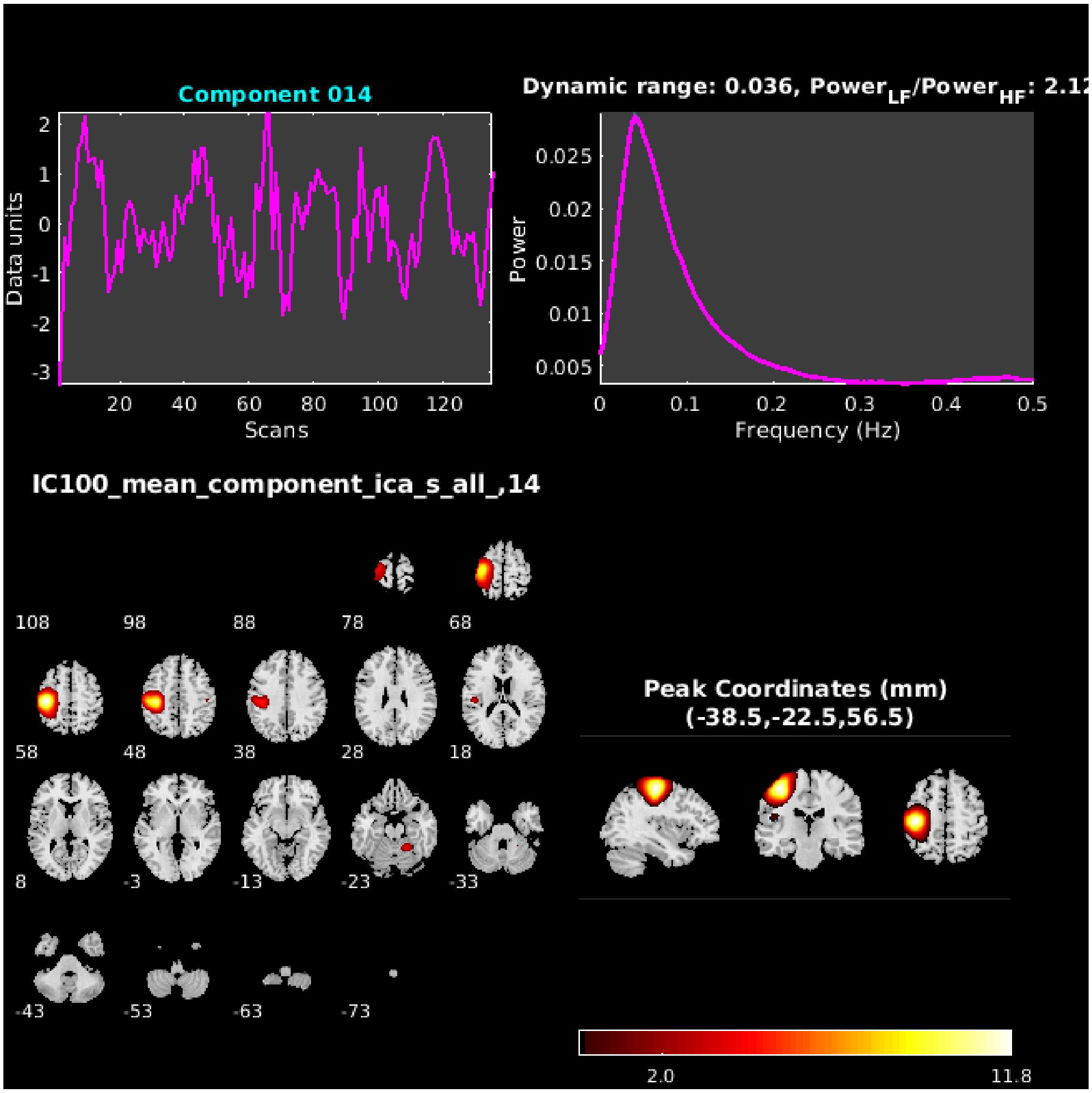

Component 014
Dynamic range: 0.036, Power$_{LF}$/Power$_{HF}$: 2.12
Data units
Scans
Power
Frequency (Hz)
IC100_mean_component_ica_s_all_,14
Peak Coordinates (mm)
(-38.5,-22.5,56.5)
2.0
11.8

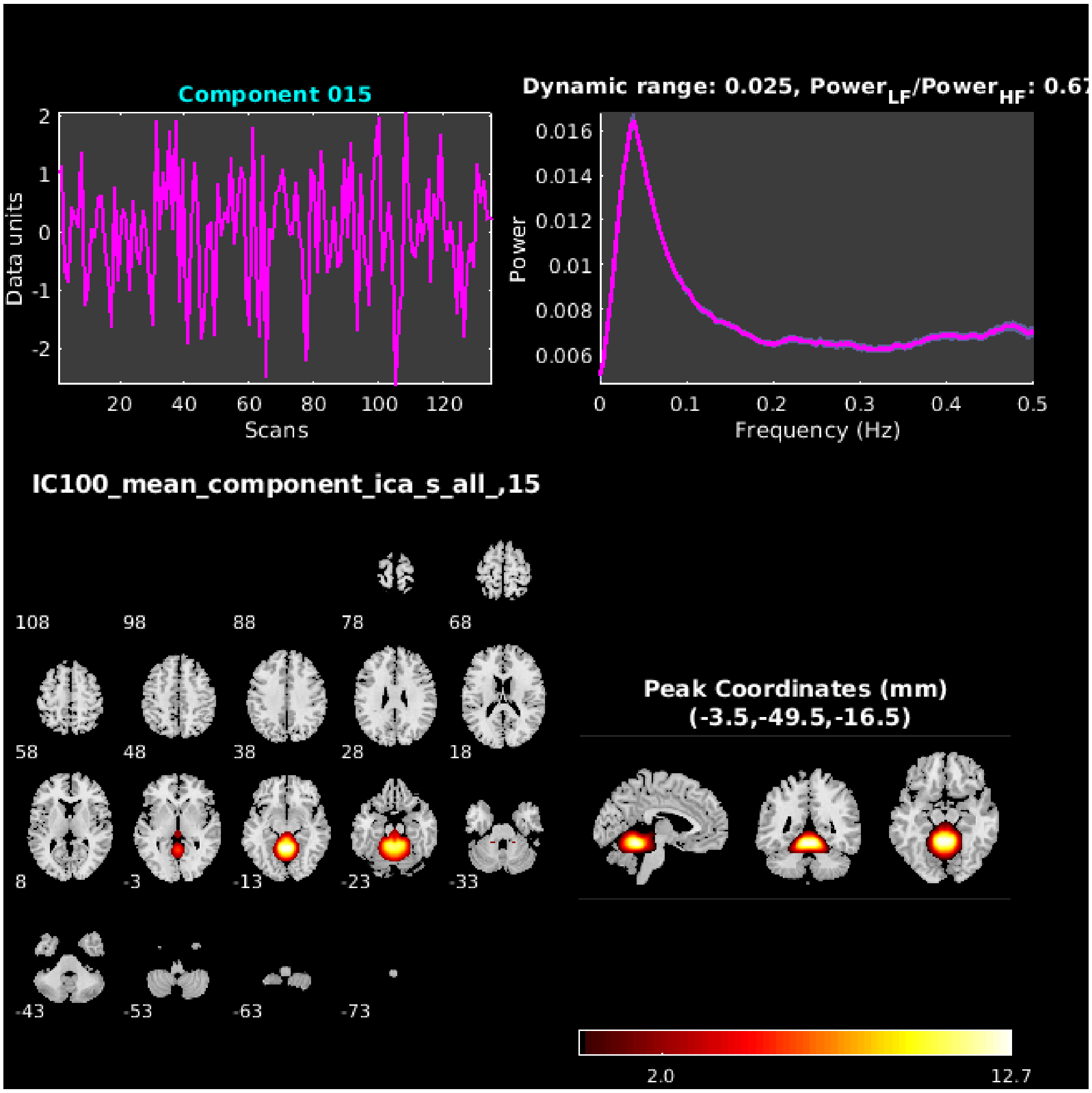

Component 015
Dynamic range: 0.025, Power$_{LF}$/Power$_{HF}$: 0.67
Data units
Scans
Power
Frequency (Hz)
IC100_mean_component_ica_s_all_,15
Peak Coordinates (mm)
(-3.5,-49.5,-16.5)

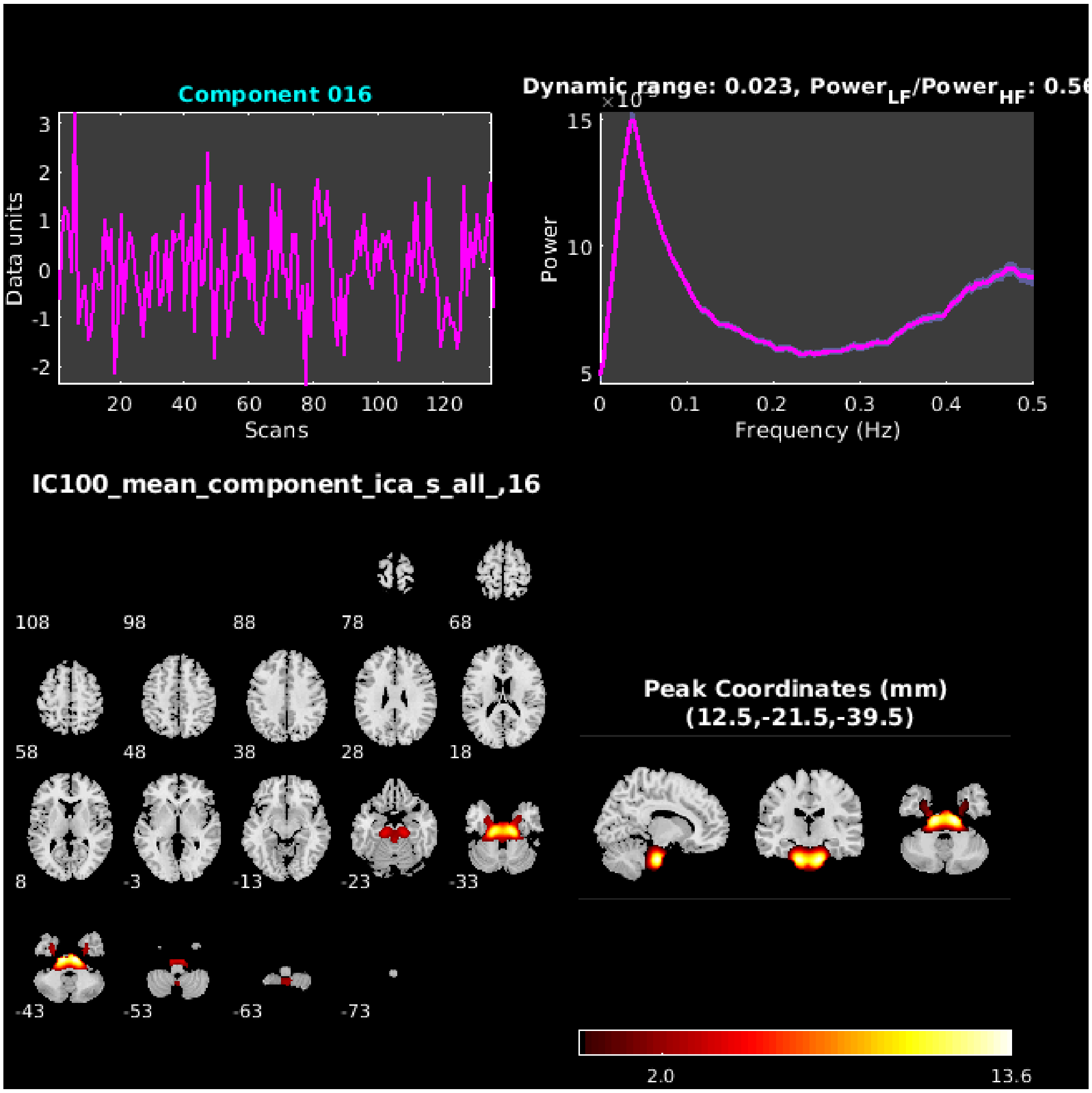
Component 016
Dynamic range: 0.023, Power$_{LF}$/Power$_{HF}$: 0.56
Data units
Scans
Power
Frequency (Hz)
IC100_mean_component_ica_s_all_,16
Peak Coordinates (mm)
(12.5,-21.5,-39.5)
2.0
13.6

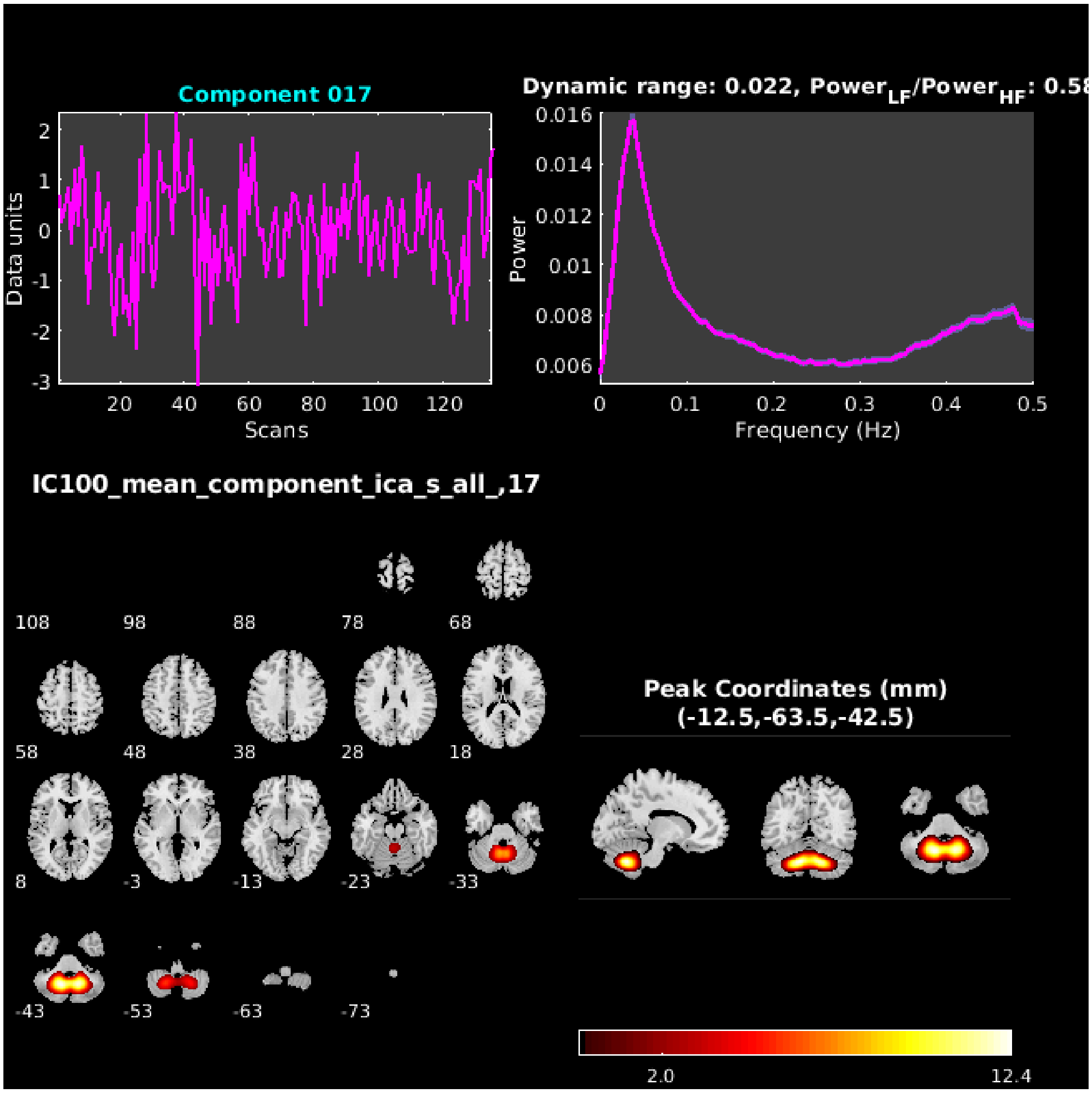

Component 017
Dynamic range: 0.022, Power$_{LF}$/Power$_{HF}$: 0.58
Data units
Scans
Power
Frequency (Hz)
IC100_mean_component_ica_s_all_,17
Peak Coordinates (mm)
(-12.5,-63.5,-42.5)
2.0
12.4

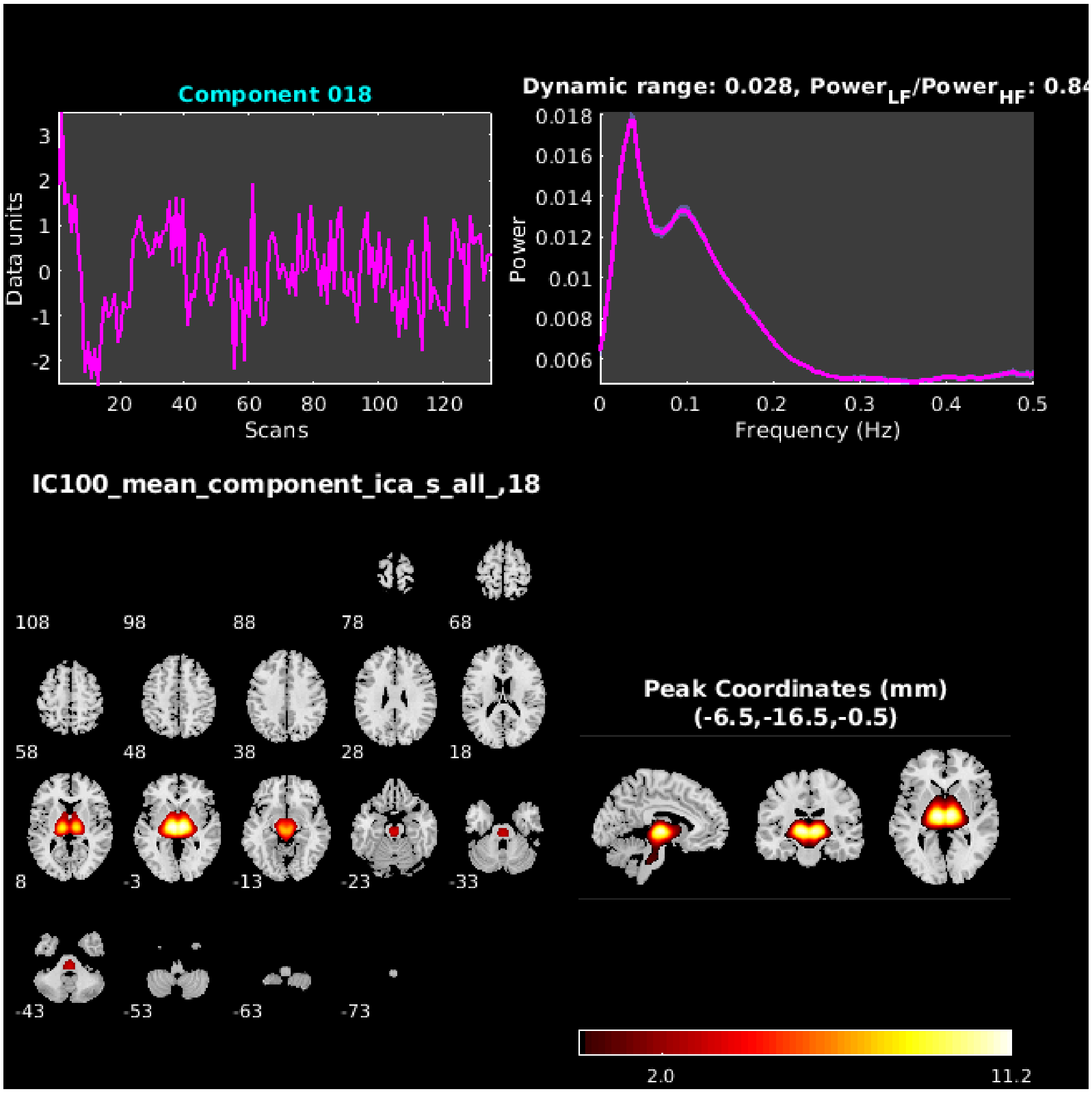
Component 018
Dynamic range: 0.028, Power_LF/Power_HF: 0.84
Data units
Scans
Power
Frequency (Hz)
IC100_mean_component_ica_s_all_,18
Peak Coordinates (mm)
(-6.5,-16.5,-0.5)
2.0
11.2

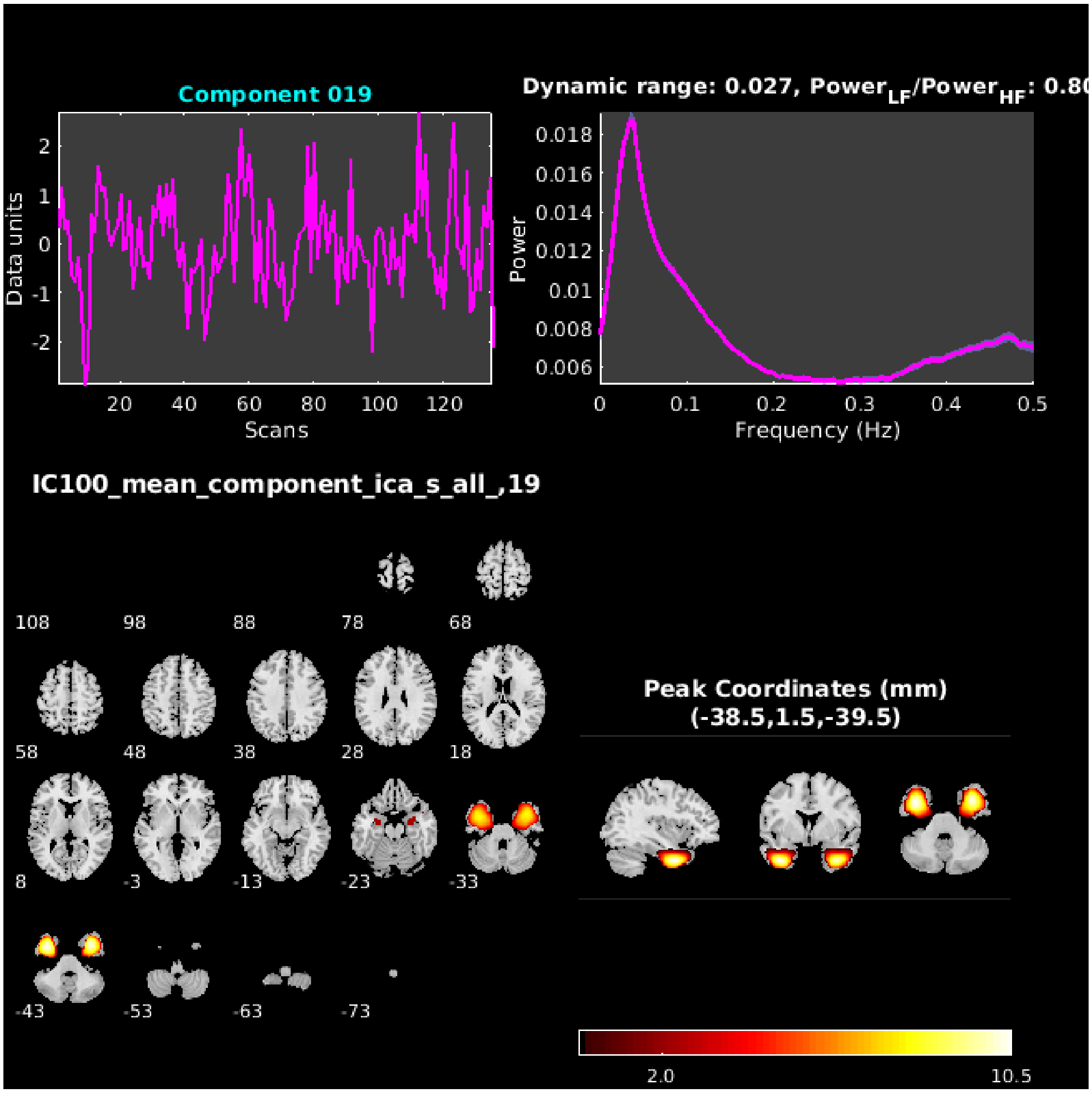
Component 019
Dynamic range: 0.027, Power_LF/Power_HF: 0.8
Data units
Scans
Power
Frequency (Hz)
IC100_mean_component_ica_s_all_,19
108
98
88
78
68
58
48
38
28
18
8
-3
-13
-23
-33
-43
-53
-63
-73
Peak Coordinates (mm)
(-38.5,1.5,-39.5)
2.0
10.5

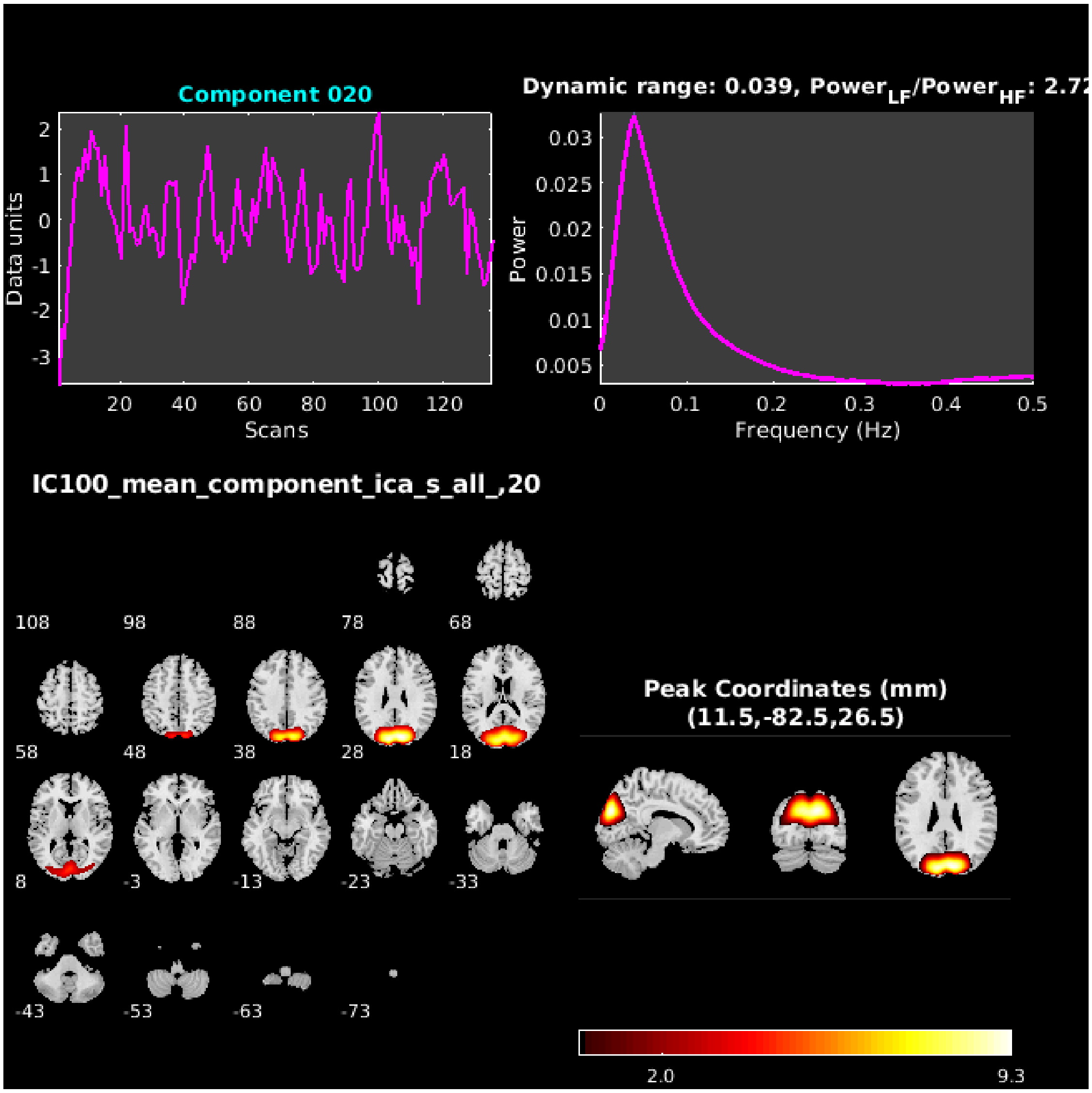
Component 020
Dynamic range: 0.039, Power$_{LF}$/Power$_{HF}$: 2.72
Data units
Scans
Power
Frequency (Hz)
IC100_mean_component_ica_s_all_,20
Peak Coordinates (mm)
(11.5,-82.5,26.5)
2.0
9.3

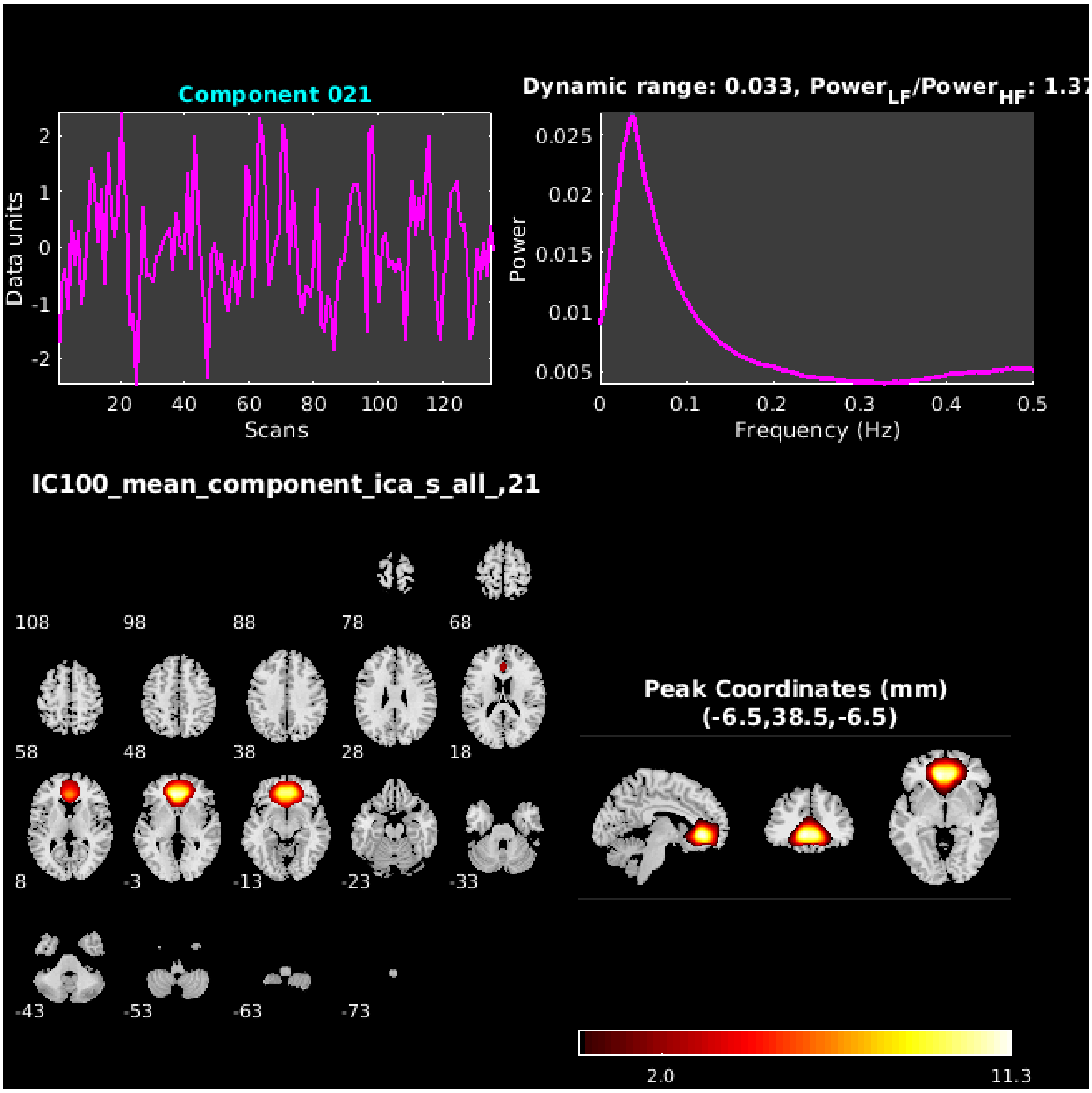
Component 021
Dynamic range: 0.033, Power_LF/Power_HF: 1.37
Data units
Scans
Power
Frequency (Hz)
IC100_mean_component_ica_s_all_,21
Peak Coordinates (mm)
(-6.5,38.5,-6.5)
2.0
11.3

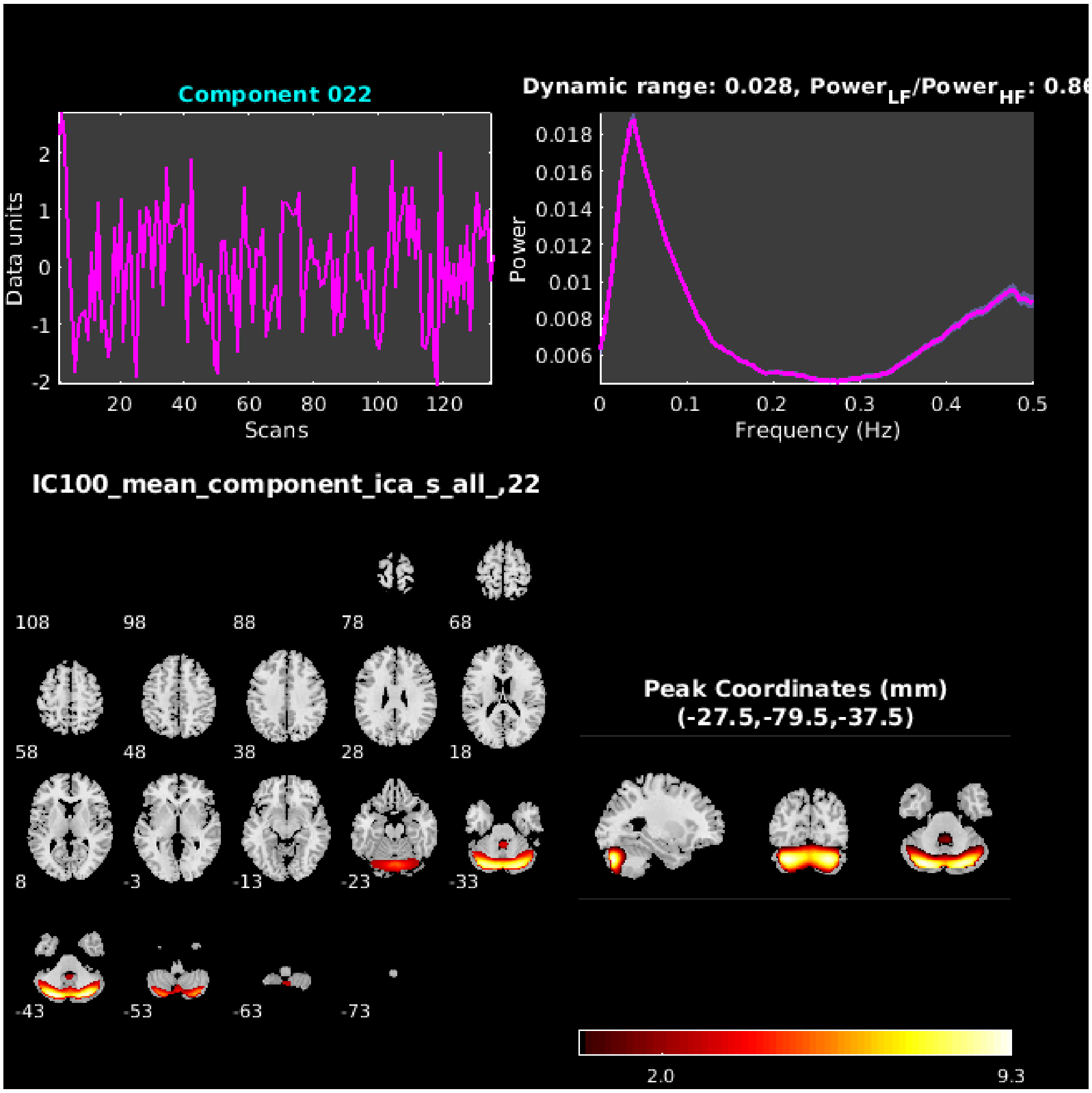
Component 022
Dynamic range: 0.028, Power$_{LF}$/Power$_{HF}$: 0.86
Data units
Scans
Power
Frequency (Hz)
IC100_mean_component_ica_s_all_,22
Peak Coordinates (mm)
(-27.5,-79.5,-37.5)
2.0
9.3

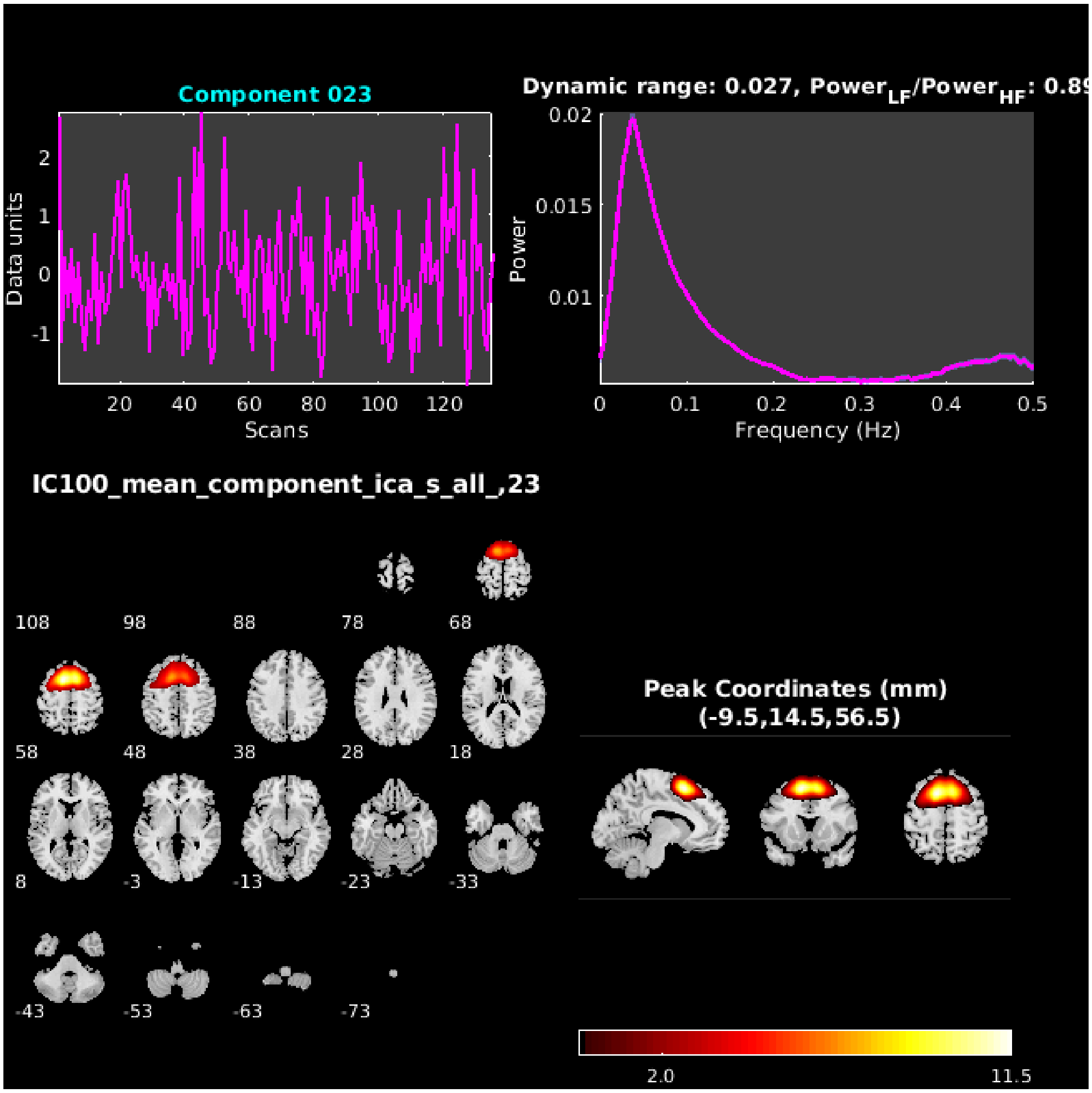

Component 023
Dynamic range: 0.027, Power$_{LF}$/Power$_{HF}$: 0.89
Data units
Scans
Power
Frequency (Hz)
IC100_mean_component_ica_s_all_,23
108
98
88
78
68
58
48
38
28
18
8
-3
-13
-23
-33
-43
-53
-63
-73
Peak Coordinates (mm)
(-9.5,14.5,56.5)
2.0
11.5

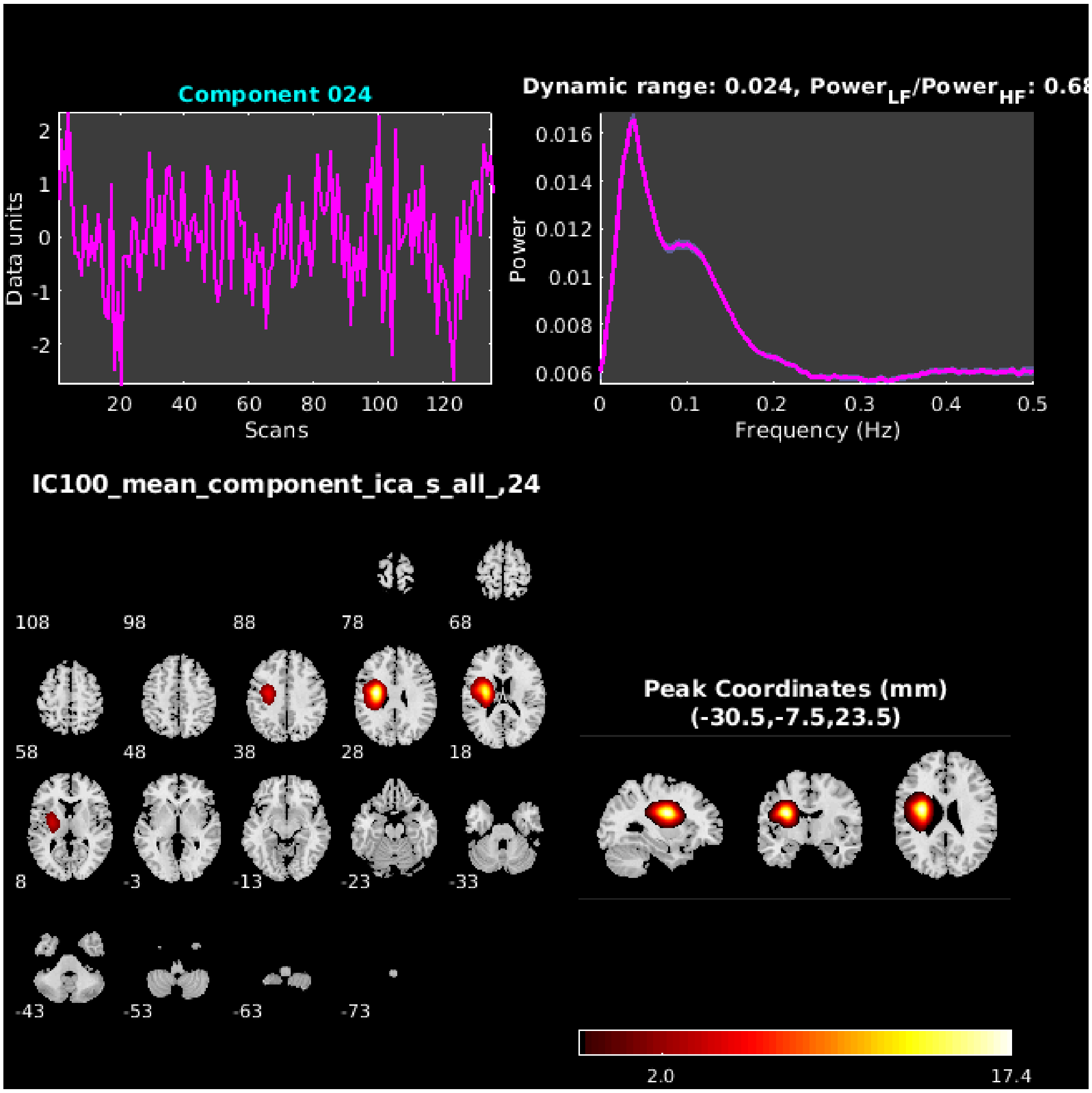
Component 024
Dynamic range: 0.024, Power_LF/Power_HF: 0.68
Data units
Scans
Power
Frequency (Hz)
IC100_mean_component_ica_s_all_,24
Peak Coordinates (mm)
(-30.5,-7.5,23.5)
2.0
17.4

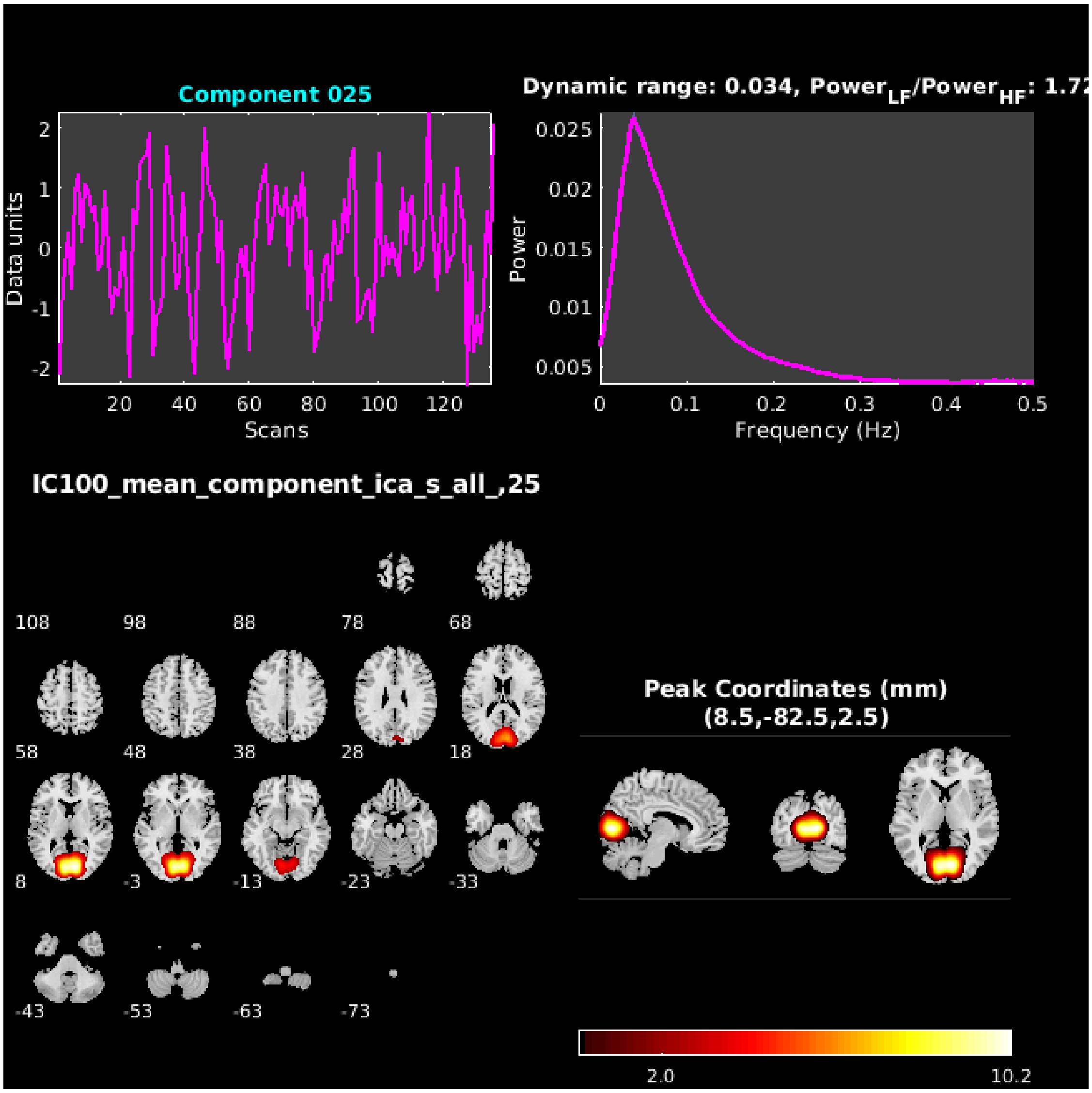
Component 025
Dynamic range: 0.034, Power$_{LF}$/Power$_{HF}$: 1.72
Data units
Scans
Power
Frequency (Hz)
IC100_mean_component_ica_s_all_,25
108
98
88
78
68
58
48
38
28
18
8
-3
-13
-23
-33
-43
-53
-63
-73
Peak Coordinates (mm)
(8.5,-82.5,2.5)
2.0
10.2

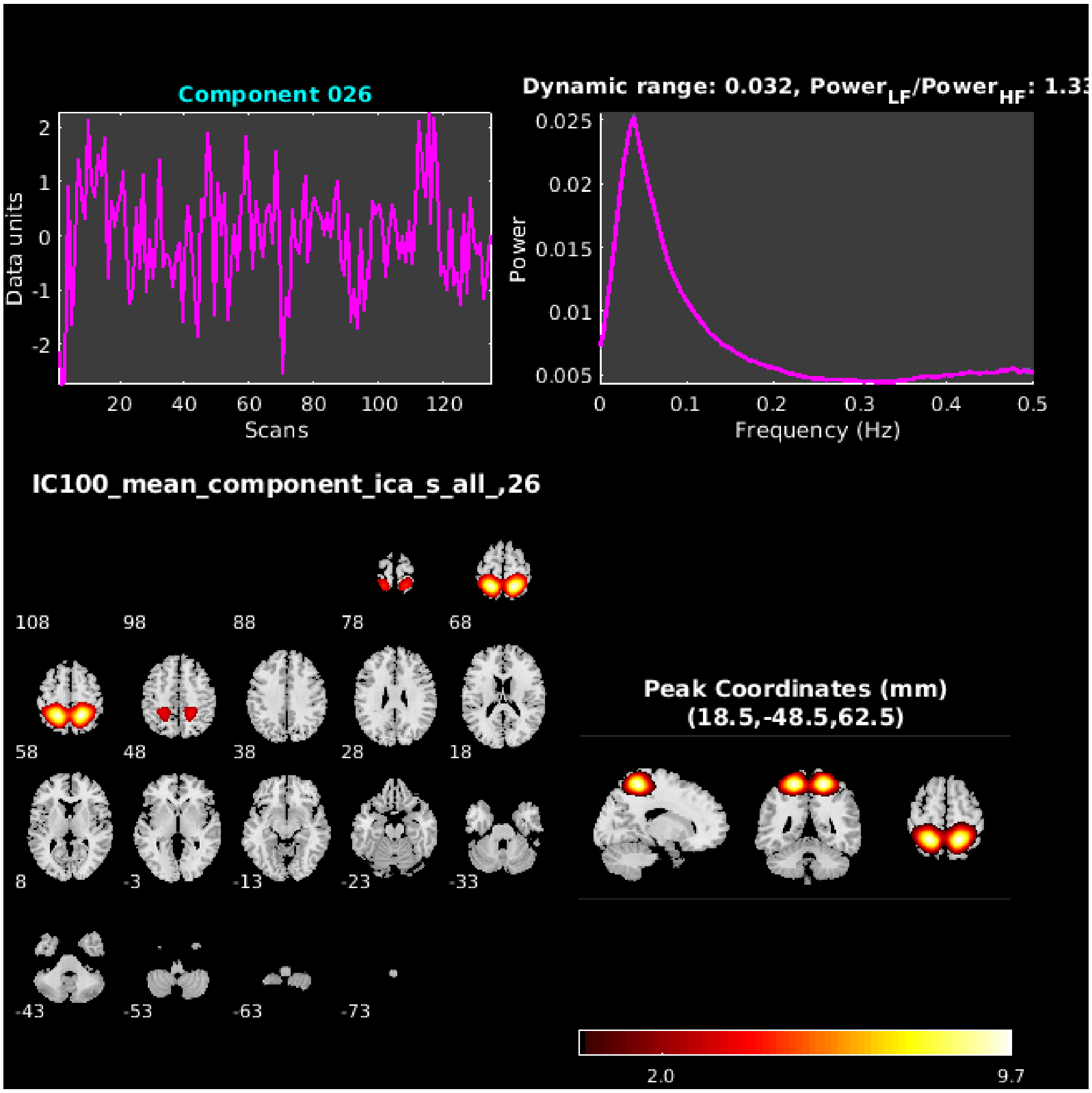
Component 026
Dynamic range: 0.032, Power$_{LF}$/Power$_{HF}$: 1.33
Data units
Scans
Power
Frequency (Hz)
IC100_mean_component_ica_s_all_,26
Peak Coordinates (mm)
(18.5,-48.5,62.5)
2.0
9.7

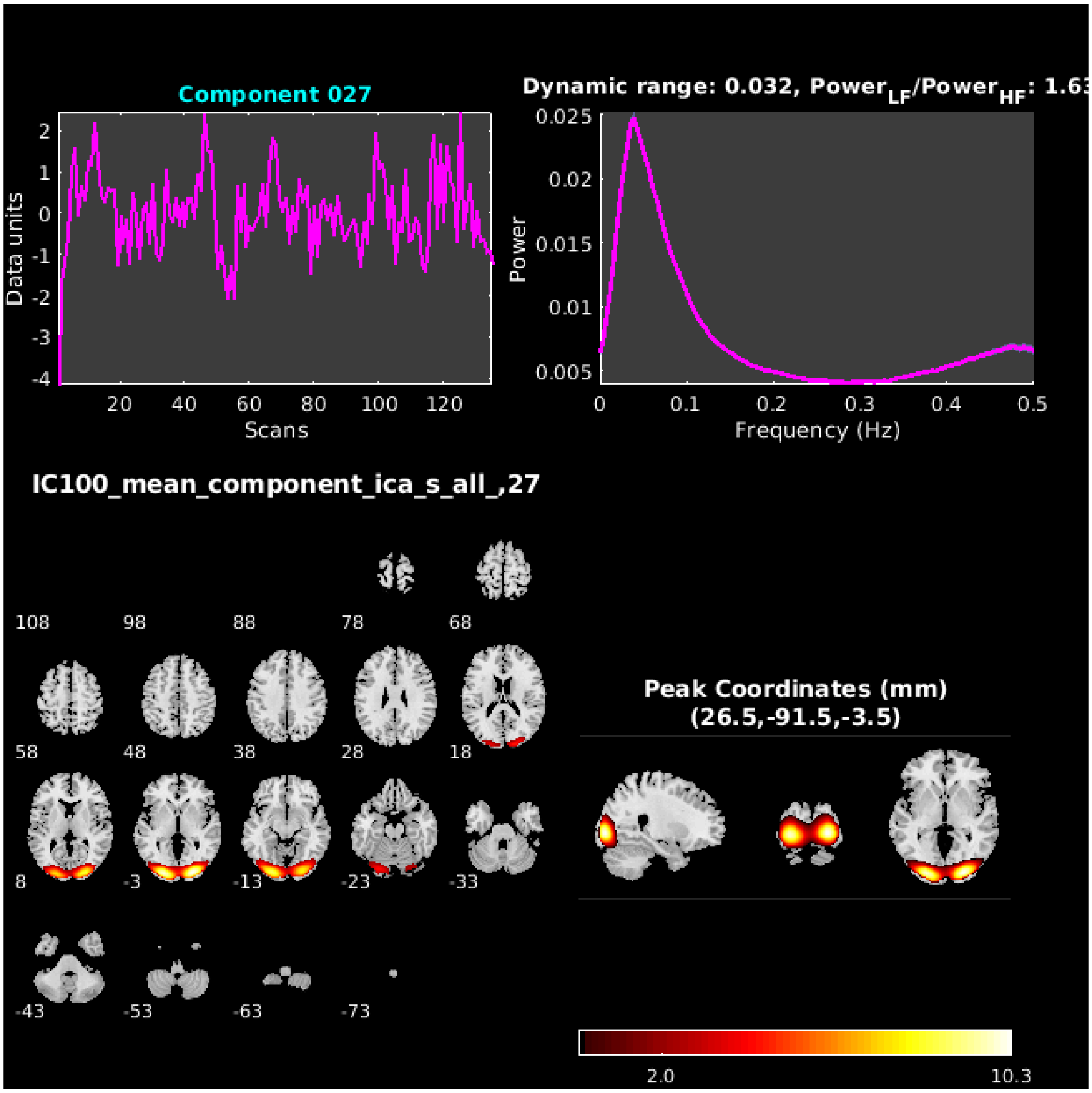
Component 027
Dynamic range: 0.032, Power$_{LF}$/Power$_{HF}$: 1.63
Data units
Scans
Power
Frequency (Hz)
IC100_mean_component_ica_s_all_,27
Peak Coordinates (mm)
(26.5,-91.5,-3.5)
2.0
10.3

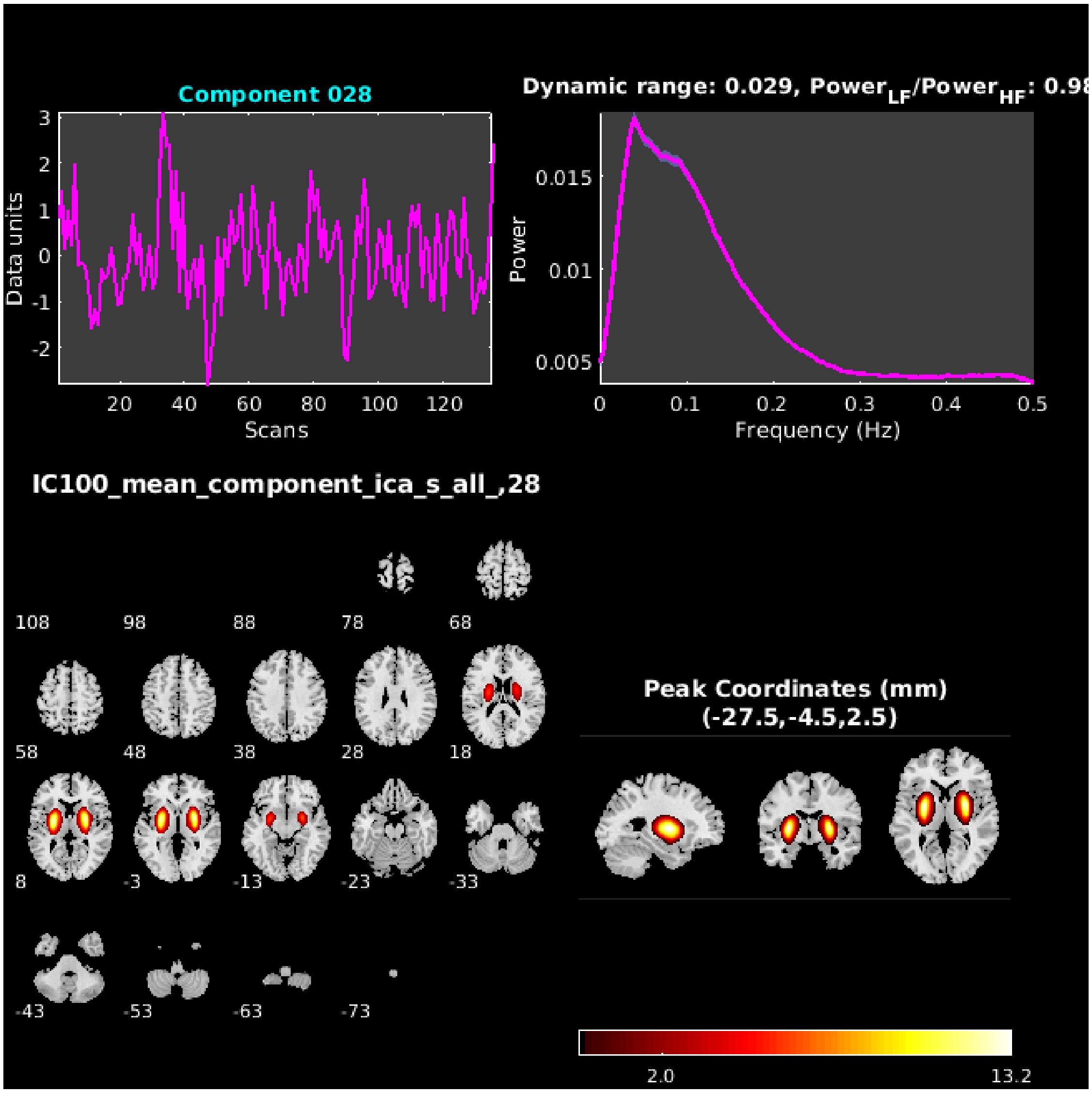

Component 028
Dynamic range: 0.029, Power_LF/Power_HF: 0.98
Data units
Scans
Power
Frequency (Hz)
IC100_mean_component_ica_s_all_,28
108
98
88
78
68
58
48
38
28
18
8
-3
-13
-23
-33
-43
-53
-63
-73
Peak Coordinates (mm)
(-27.5,-4.5,2.5)
2.0
13.2

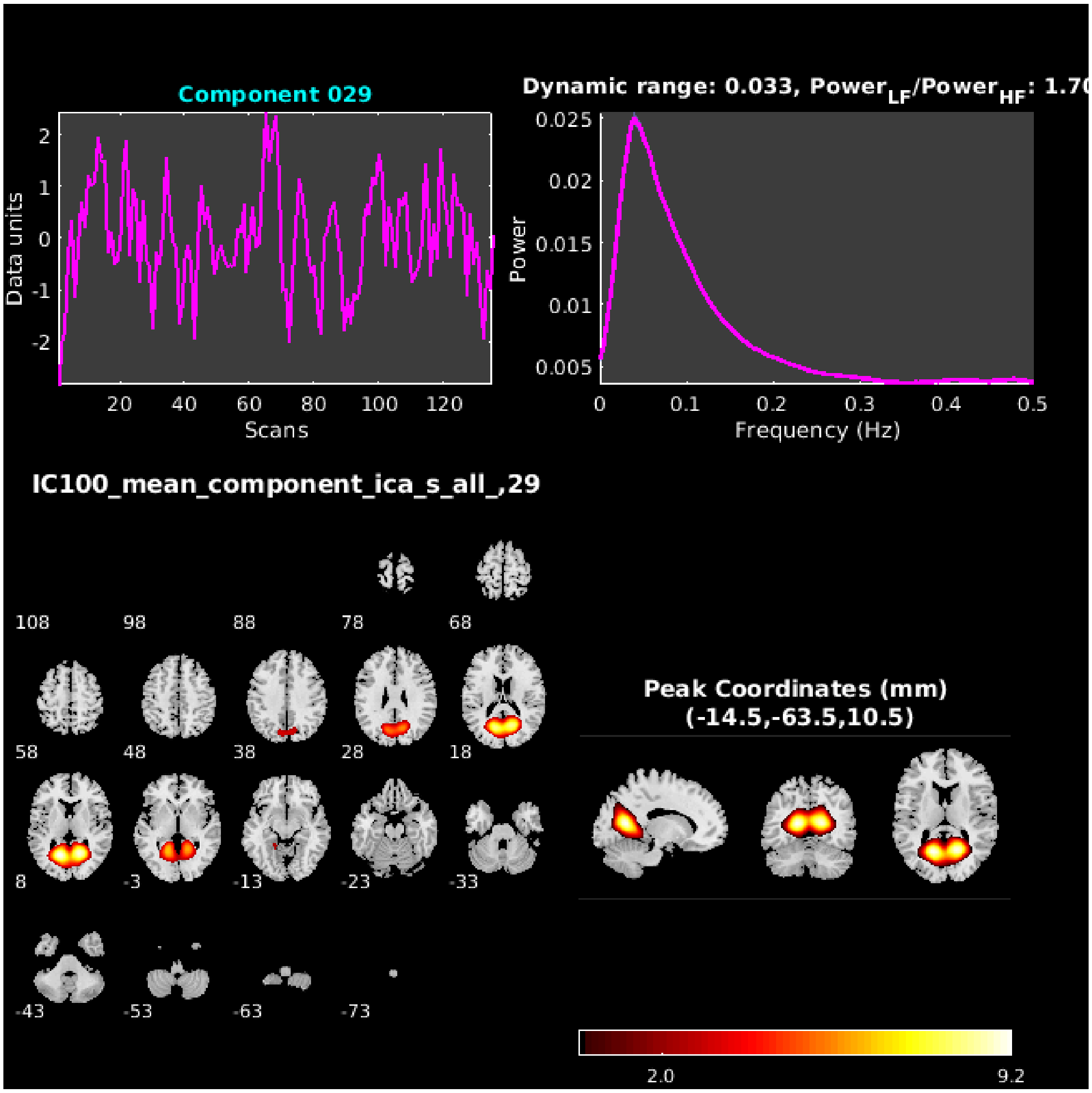

Component 029
Dynamic range: 0.033, Power$_{LF}$/Power$_{HF}$: 1.7
Data units
Scans
Power
Frequency (Hz)
IC100_mean_component_ica_s_all_,29
Peak Coordinates (mm)
(-14.5,-63.5,10.5)
2.0
9.2

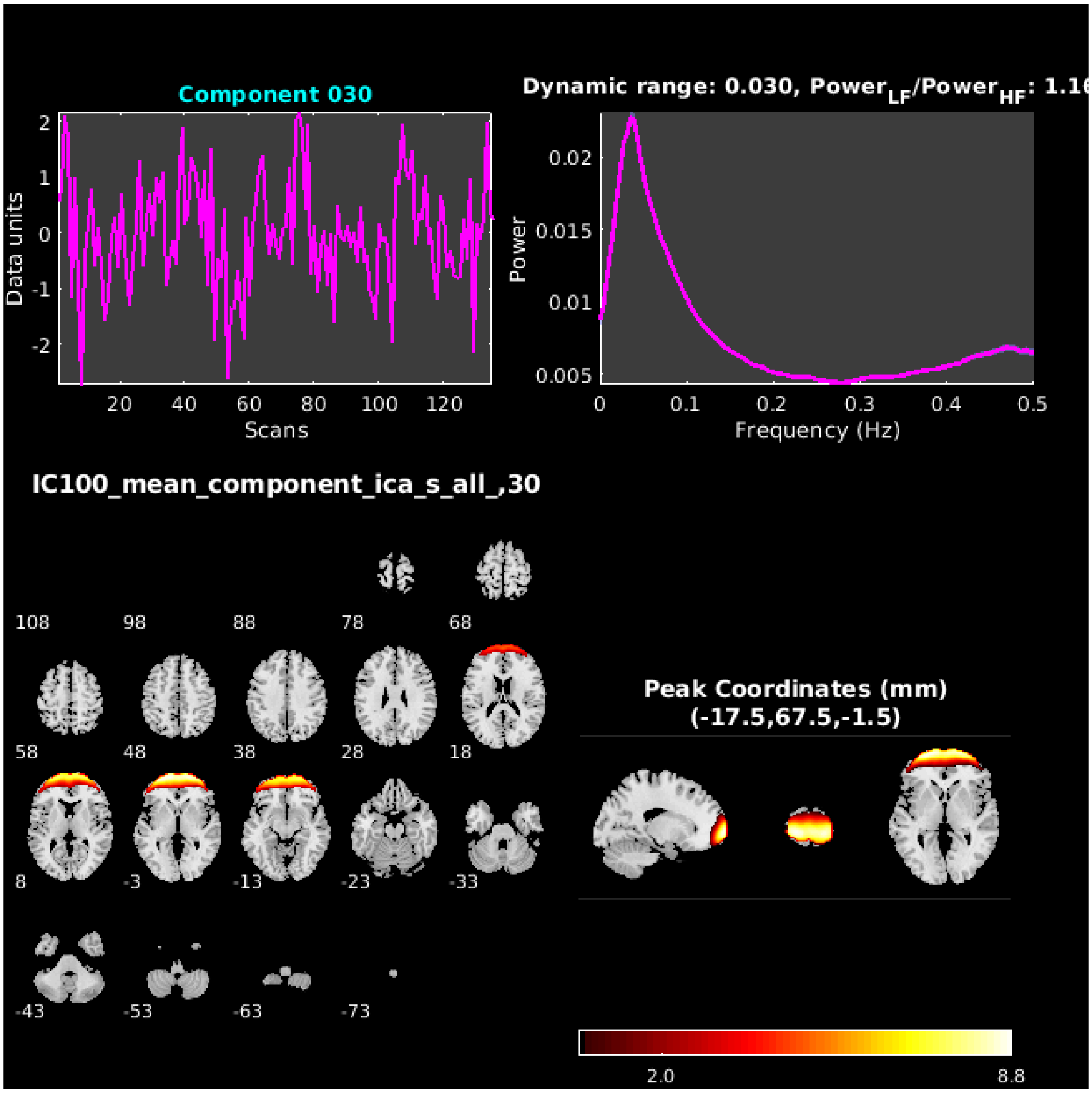

Component 030
Dynamic range: 0.030, Power_LF/Power_HF: 1.16
Data units
Scans
Power
Frequency (Hz)
IC100_mean_component_ica_s_all_,30
108
98
88
78
68
58
48
38
28
18
8
-3
-13
-23
-33
-43
-53
-63
-73
Peak Coordinates (mm)
(-17.5,67.5,-1.5)
2.0
8.8

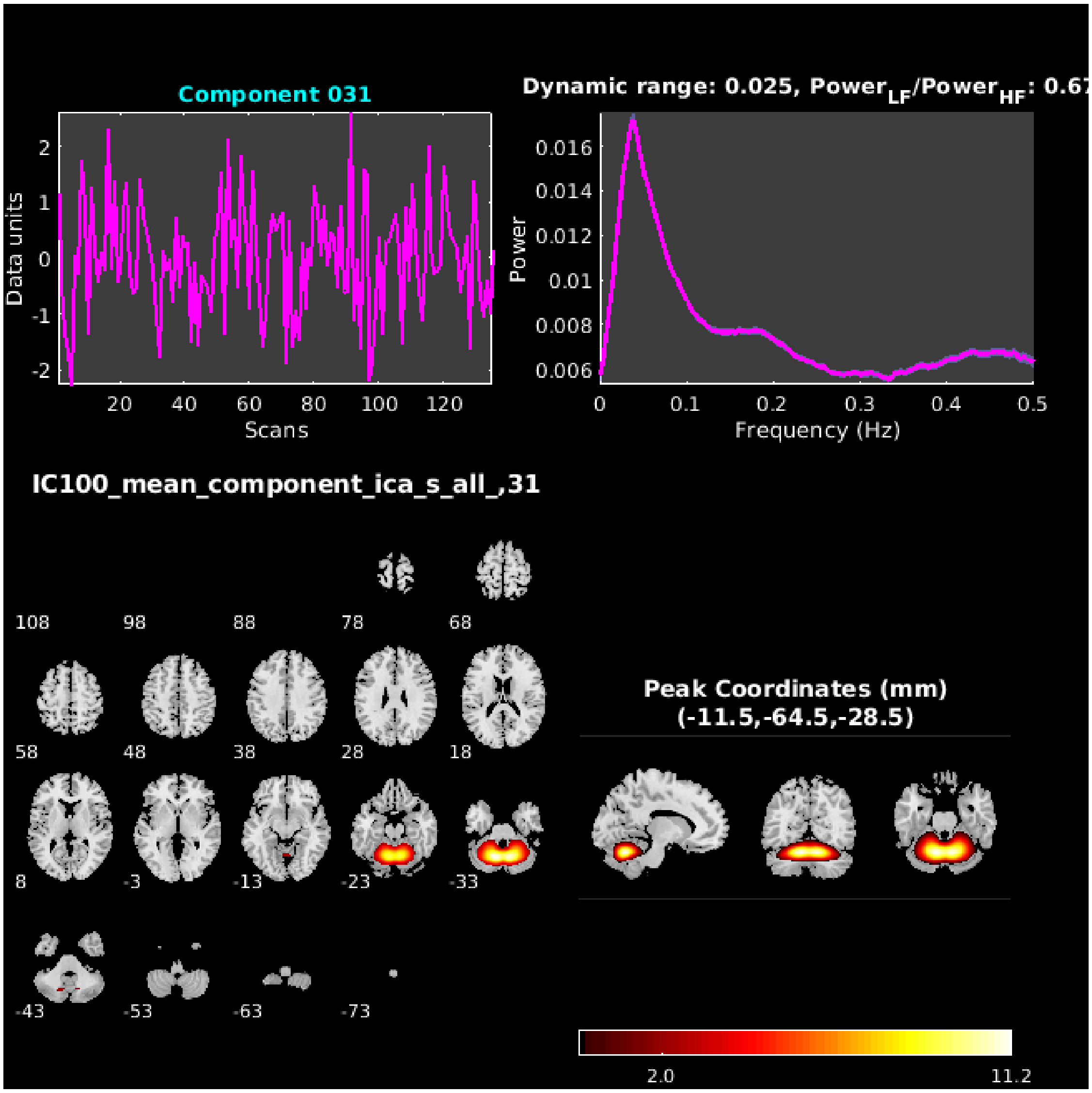
Component 031
Dynamic range: 0.025, Power$_{LF}$/Power$_{HF}$: 0.67
Data units
Scans
Power
Frequency (Hz)
IC100_mean_component_ica_s_all_,31
Peak Coordinates (mm)
(-11.5,-64.5,-28.5)
2.0
11.2

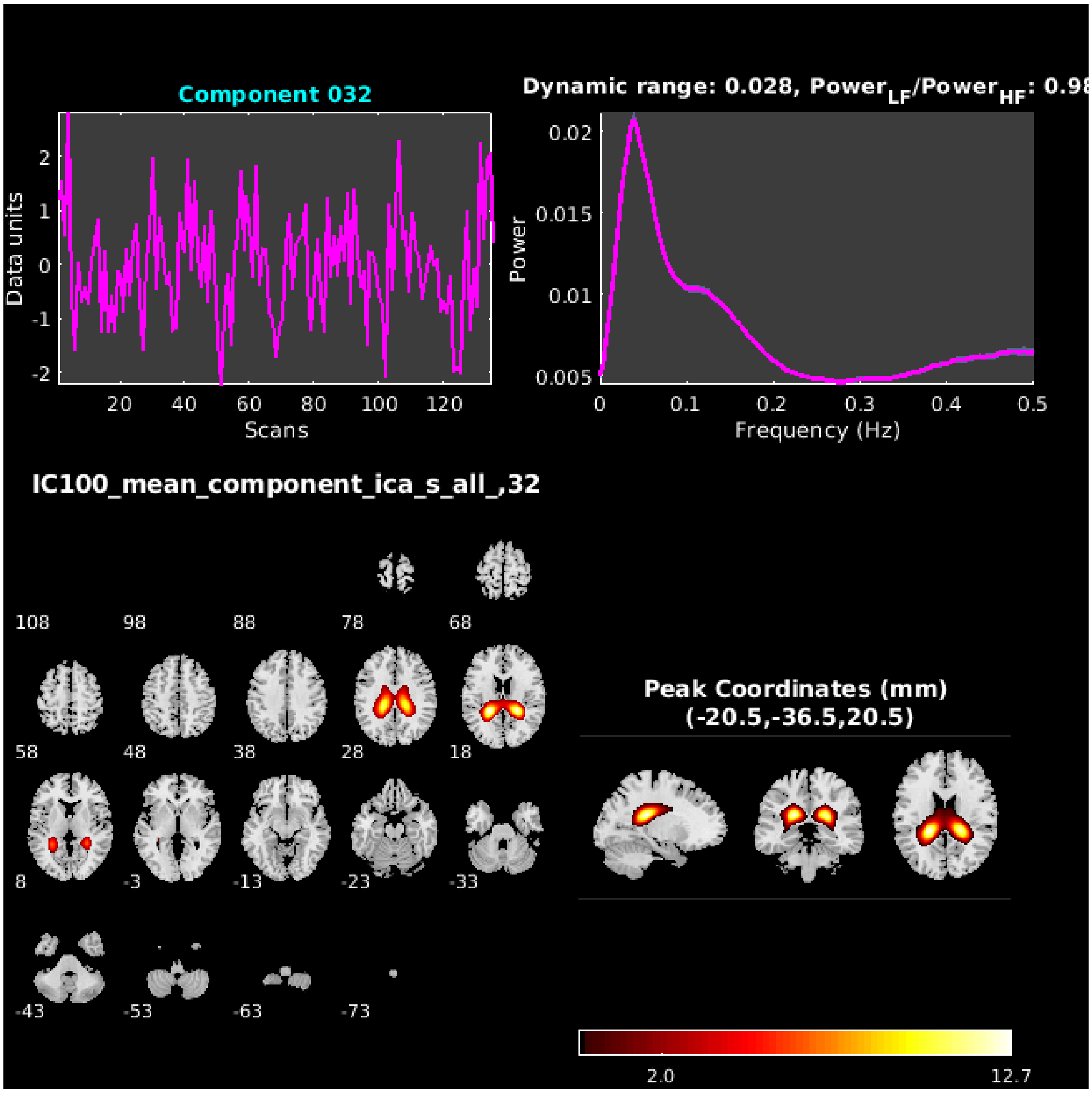
Component 032
Dynamic range: 0.028, Power$_{LF}$/Power$_{HF}$: 0.98
Data units
Scans
Power
Frequency (Hz)
IC100_mean_component_ica_s_all_,32
Peak Coordinates (mm)
(-20.5,-36.5,20.5)
2.0
12.7

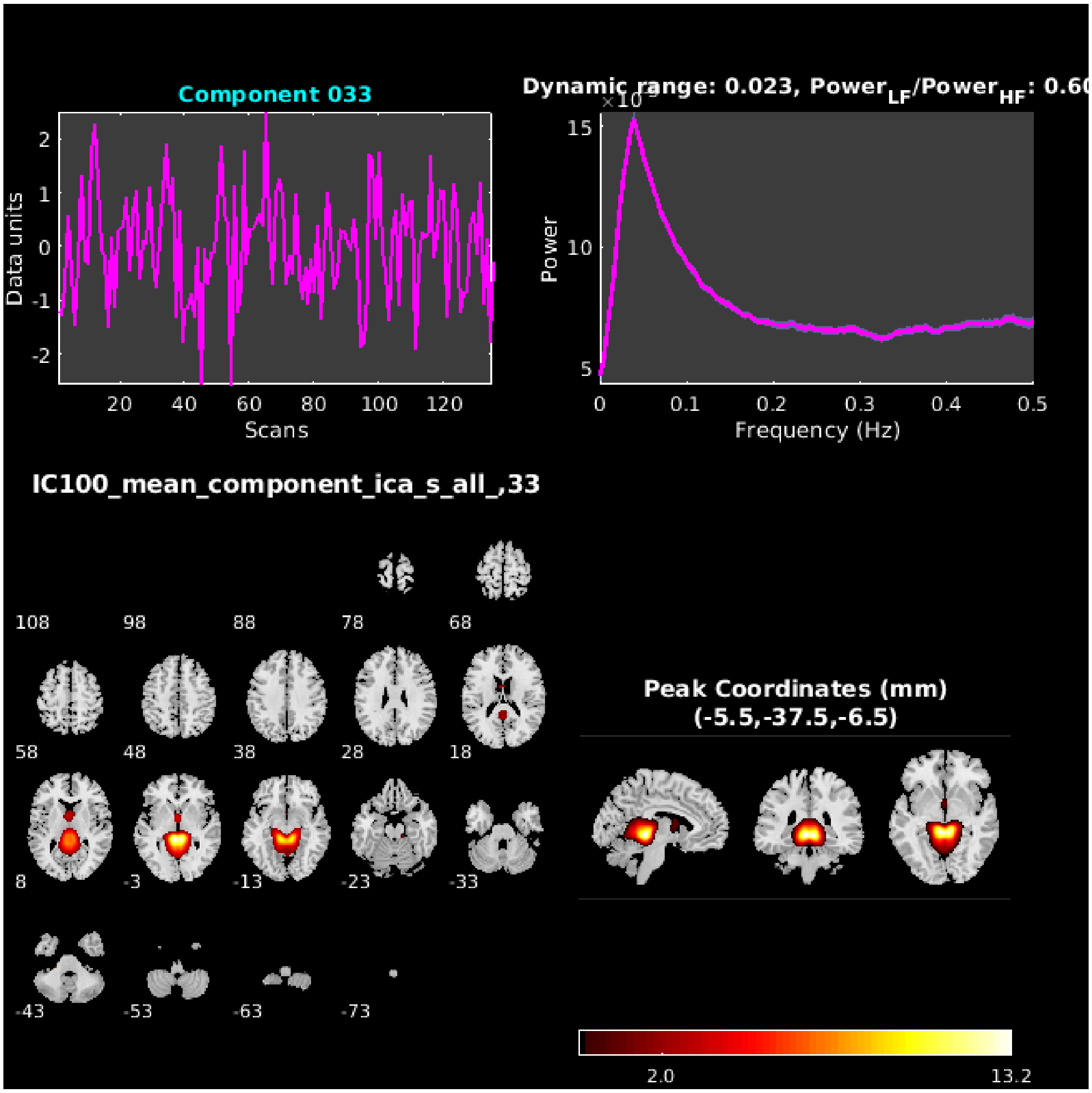
Component 033
Dynamic range: 0.023, Power$_{LF}$/Power$_{HF}$: 0.60
Data units
Scans
Power
Frequency (Hz)
IC100_mean_component_ica_s_all_,33
Peak Coordinates (mm)
(-5.5,-37.5,-6.5)
2.0
13.2

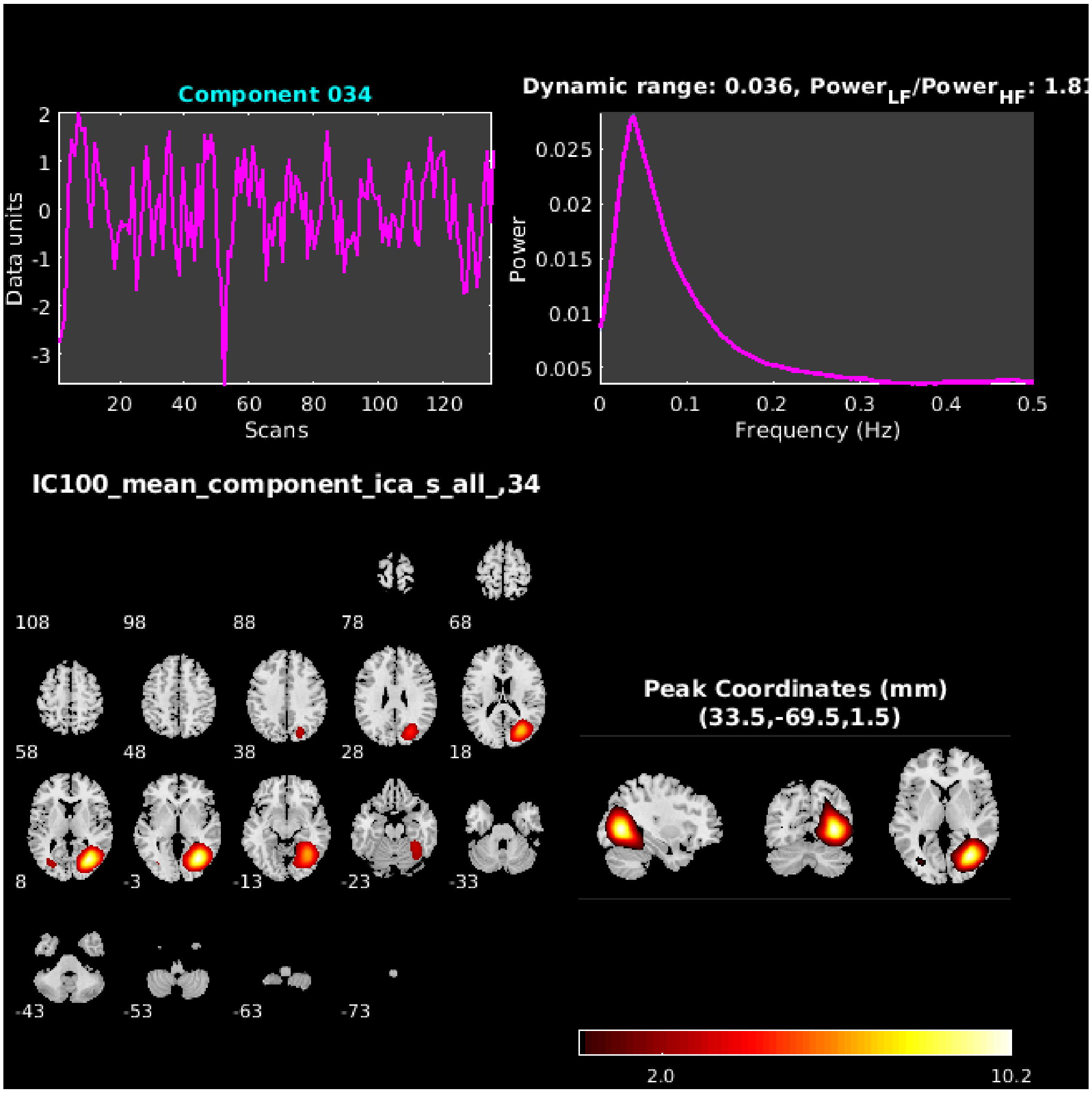
Component 034
Dynamic range: 0.036, Power$_{LF}$/Power$_{HF}$: 1.81
Data units
Scans
Power
Frequency (Hz)
IC100_mean_component_ica_s_all_,34
Peak Coordinates (mm)
(33.5,-69.5,1.5)
2.0
10.2

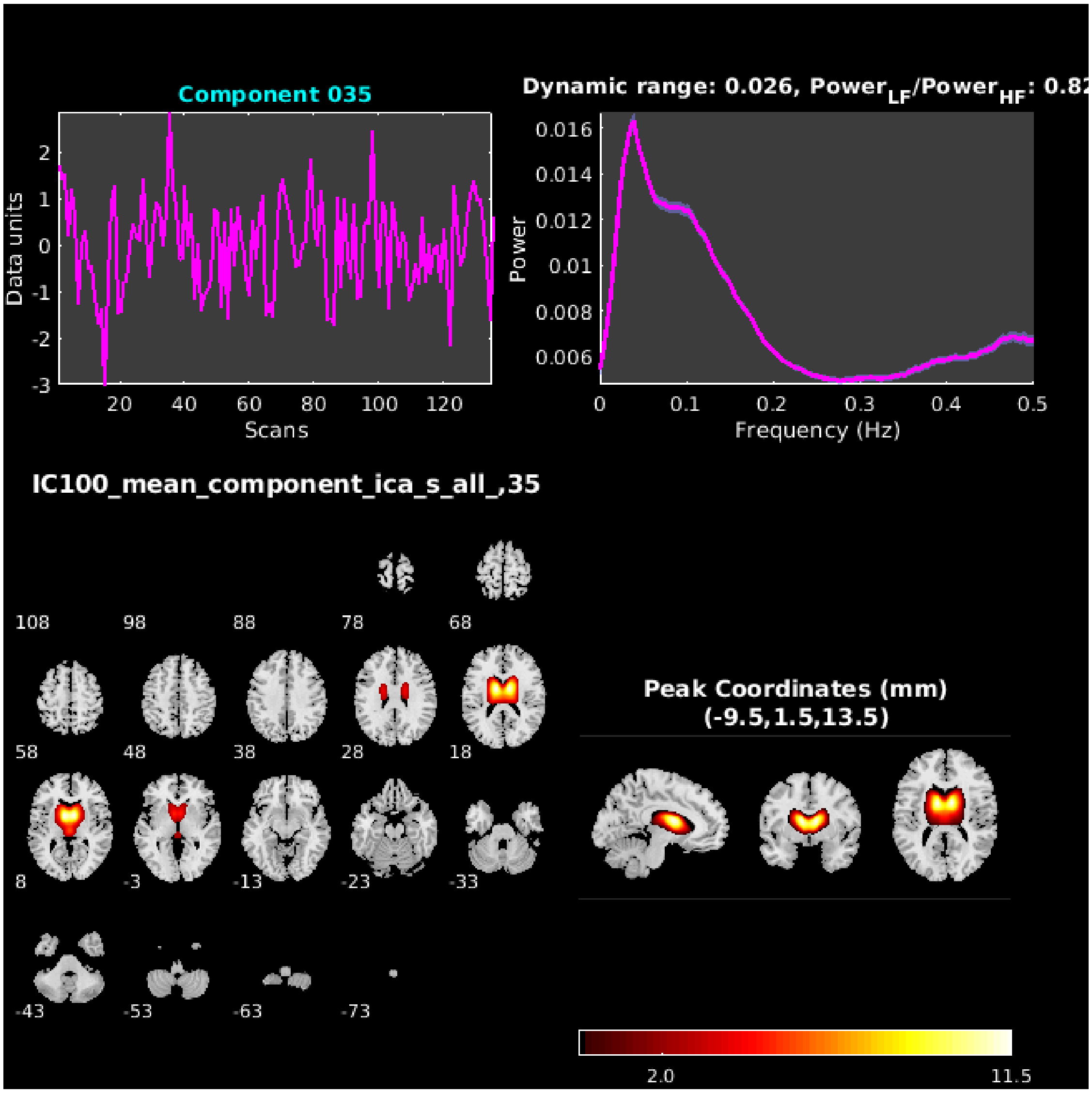
Component 035
Dynamic range: 0.026, Power_LF/Power_HF: 0.82
Data units
Scans
Power
Frequency (Hz)
IC100_mean_component_ica_s_all_,35
Peak Coordinates (mm)
(-9.5,1.5,13.5)
2.0
11.5

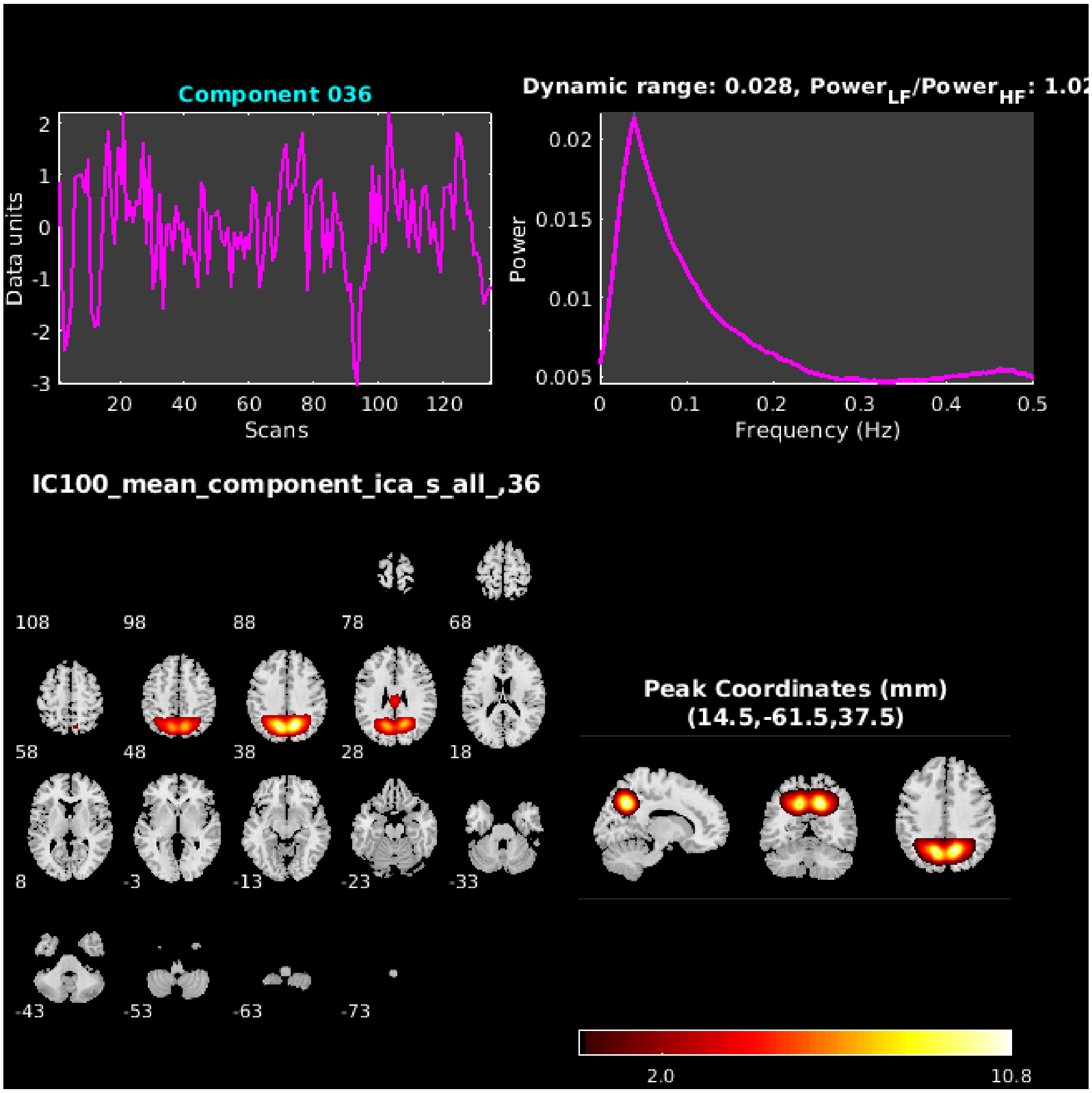
Component 036
Dynamic range: 0.028, Power$_{LF}$/Power$_{HF}$: 1.02
Data units
Scans
Power
Frequency (Hz)
IC100_mean_component_ica_s_all_,36
Peak Coordinates (mm)
(14.5,-61.5,37.5)
2.0
10.8

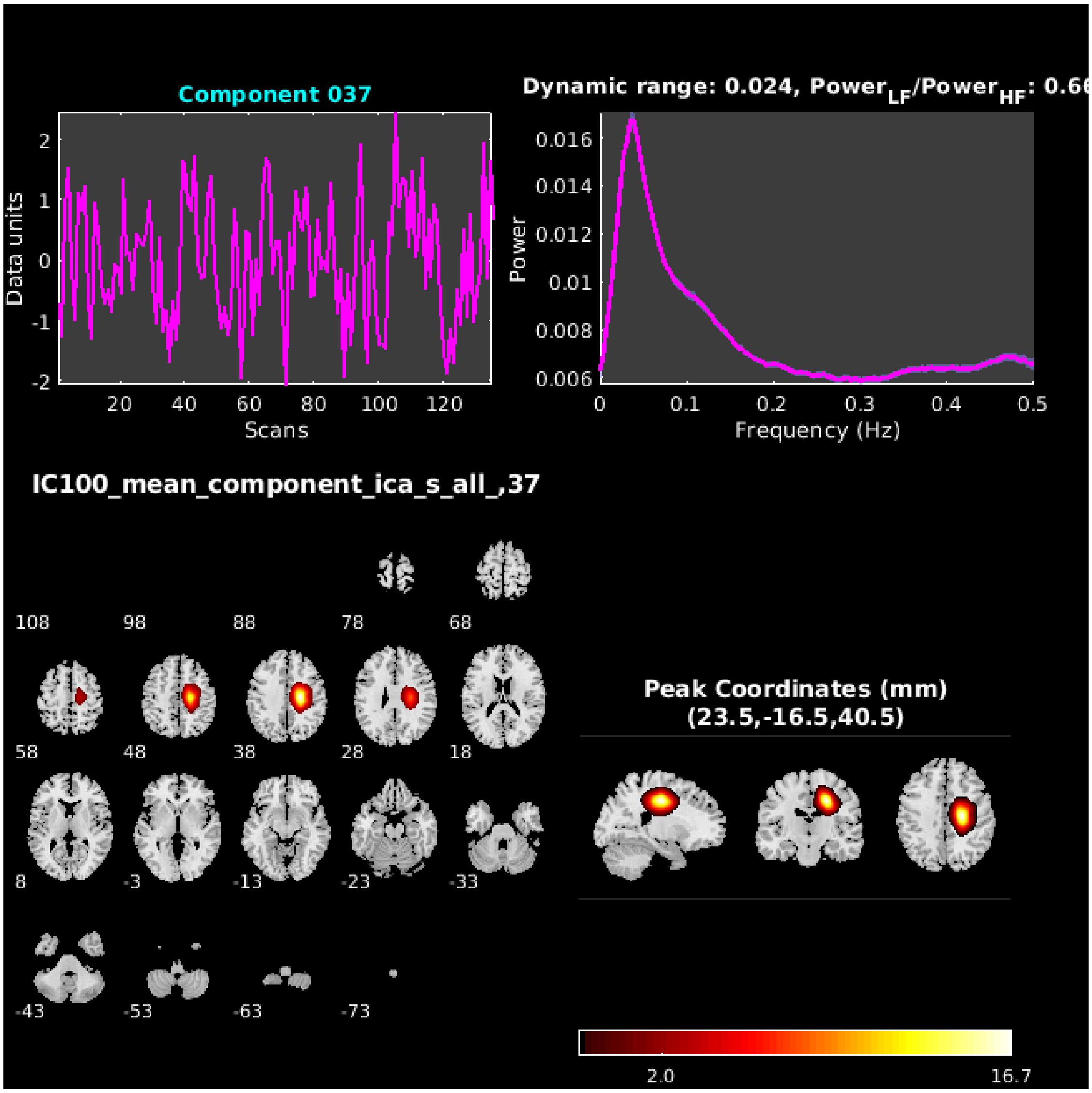
Component 037
Dynamic range: 0.024, Power$_{LF}$/Power$_{HF}$: 0.66
Data units
Scans
Power
Frequency (Hz)
IC100_mean_component_ica_s_all_,37
Peak Coordinates (mm)
(23.5,-16.5,40.5)
2.0
16.7

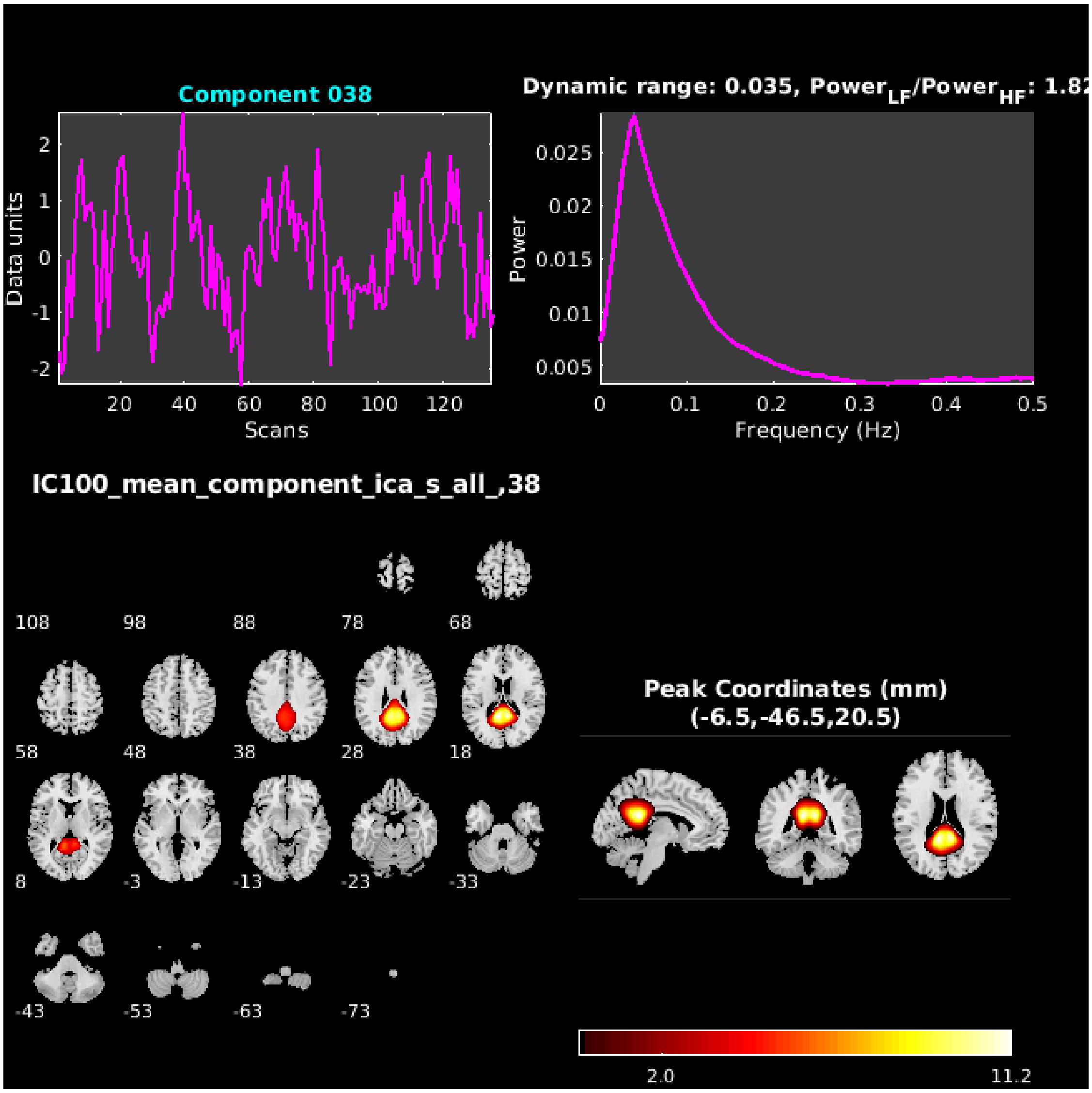
Component 038
Dynamic range: 0.035, Power_LF/Power_HF: 1.82
Data units
Scans
Power
Frequency (Hz)
IC100_mean_component_ica_s_all_,38
Peak Coordinates (mm)
(-6.5,-46.5,20.5)
2.0
11.2

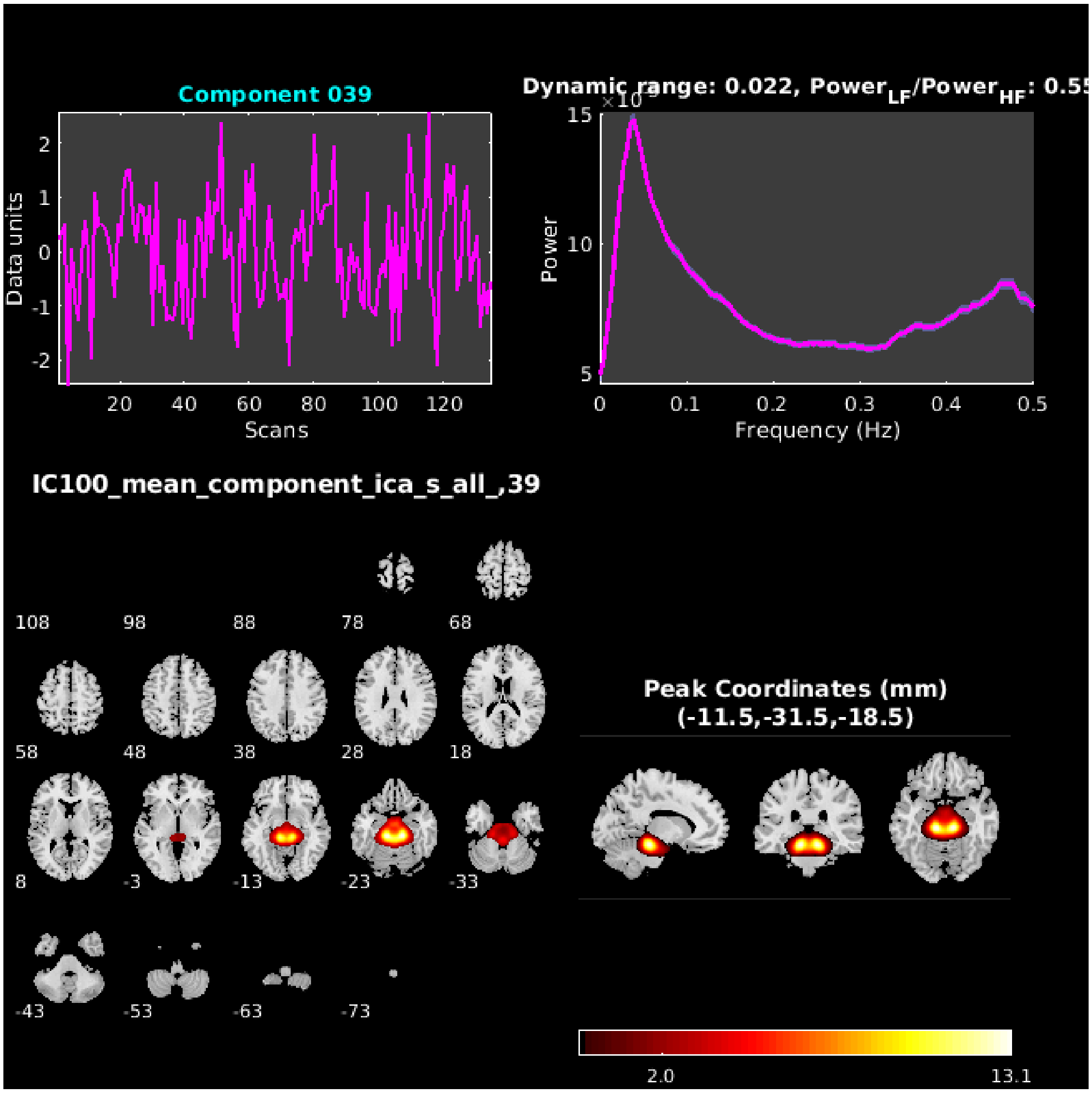
Component 039
Dynamic range: 0.022, Power$_{LF}$/Power$_{HF}$: 0.55
Data units
Scans
Power
Frequency (Hz)
IC100_mean_component_ica_s_all_,39
Peak Coordinates (mm)
(-11.5,-31.5,-18.5)
2.0
13.1

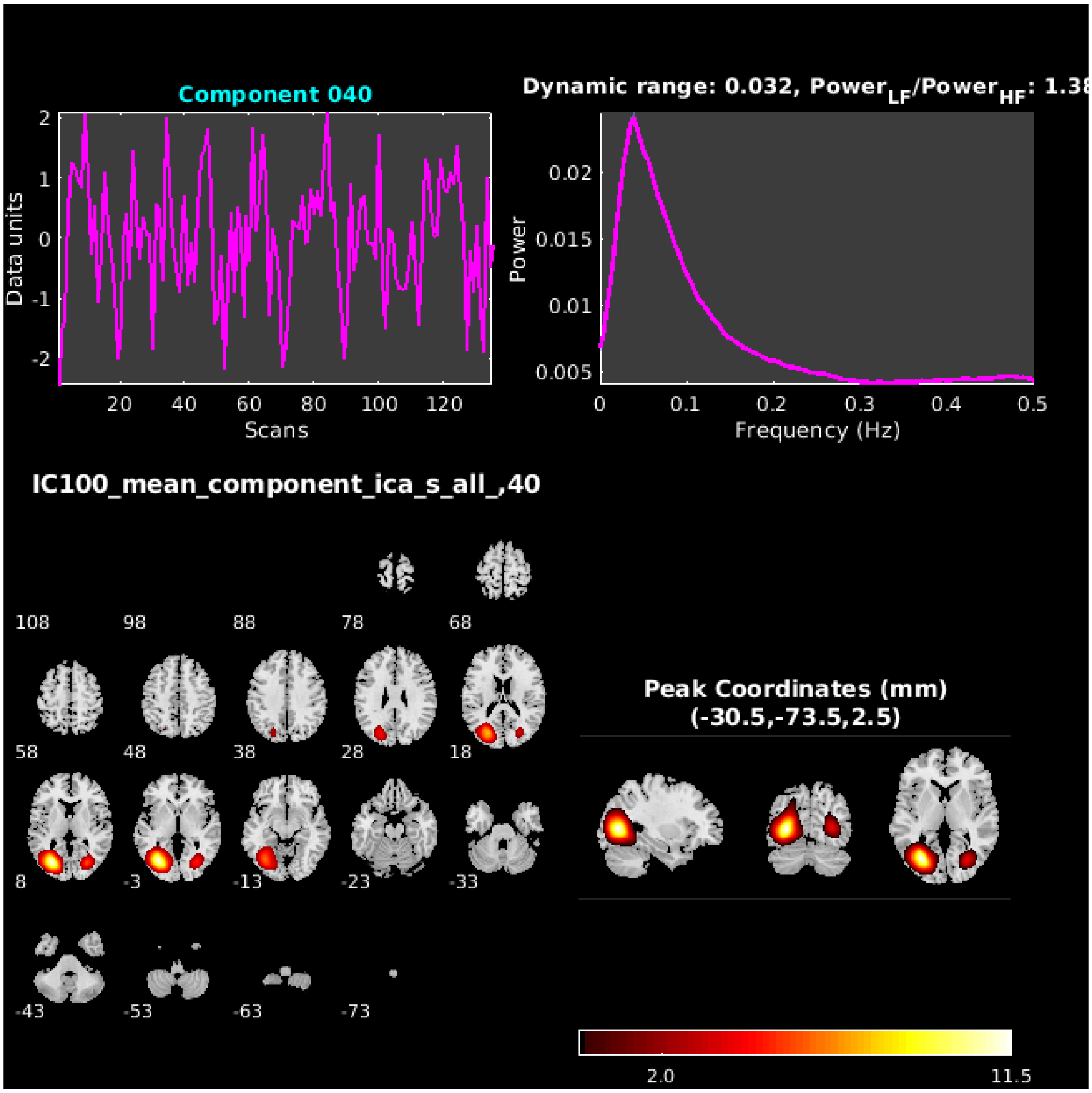

Component 040
Dynamic range: 0.032, Power$_{LF}$/Power$_{HF}$: 1.38
Data units
Scans
Power
Frequency (Hz)
IC100_mean_component_ica_s_all_,40
108
98
88
78
68
58
48
38
28
18
8
-3
-13
-23
-33
-43
-53
-63
-73
Peak Coordinates (mm)
(-30.5,-73.5,2.5)
2.0
11.5

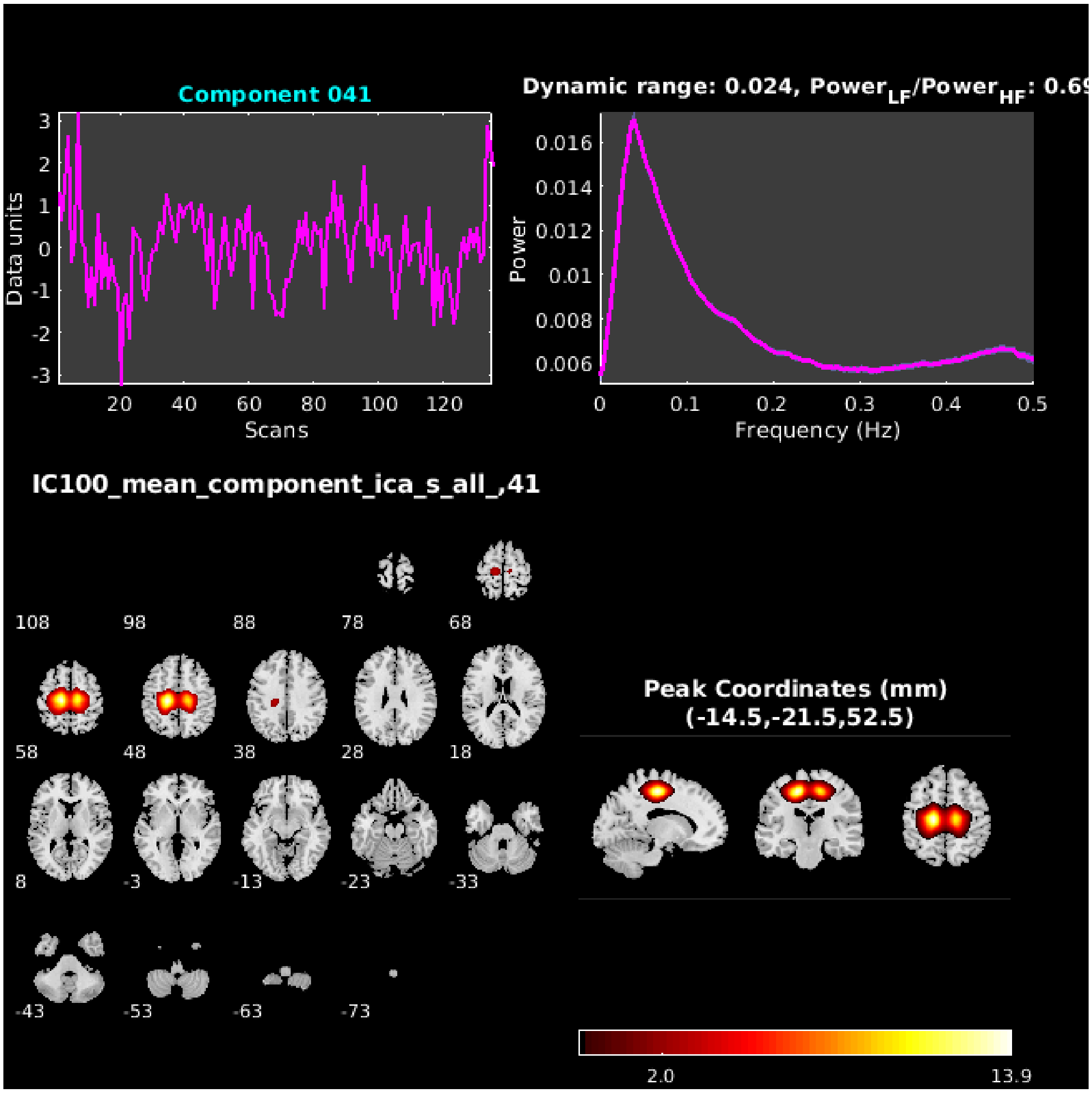

Component 041
Dynamic range: 0.024, Power_LF/Power_HF: 0.69
Data units
Scans
Power
Frequency (Hz)
IC100_mean_component_ica_s_all_,41
Peak Coordinates (mm)
(-14.5,-21.5,52.5)
2.0
13.9

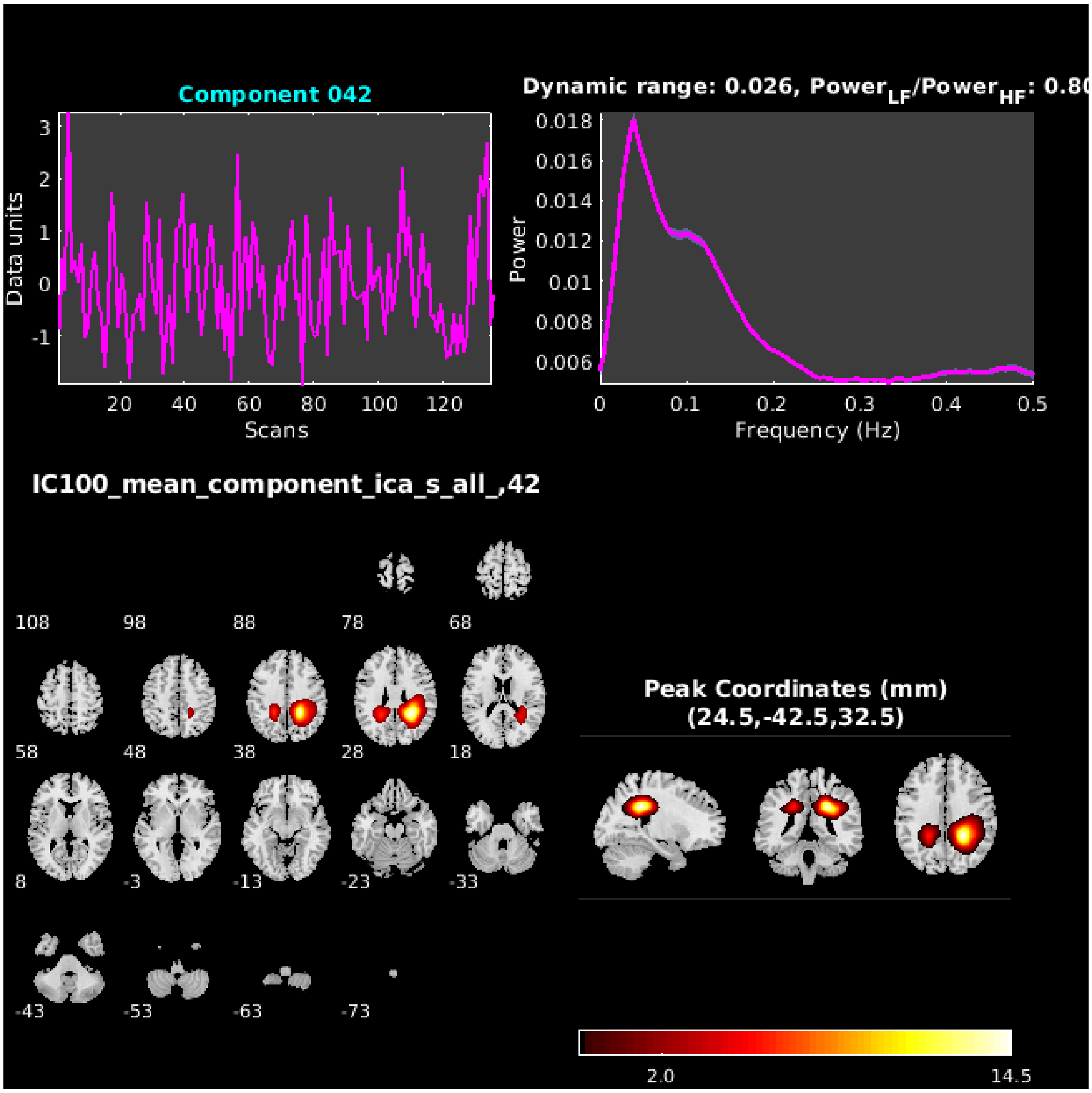
Component 042
Dynamic range: 0.026, Power$_{LF}$/Power$_{HF}$: 0.8
Data units
Scans
Power
Frequency (Hz)
IC100_mean_component_ica_s_all_,42
Peak Coordinates (mm)
(24.5,-42.5,32.5)
2.0
14.5

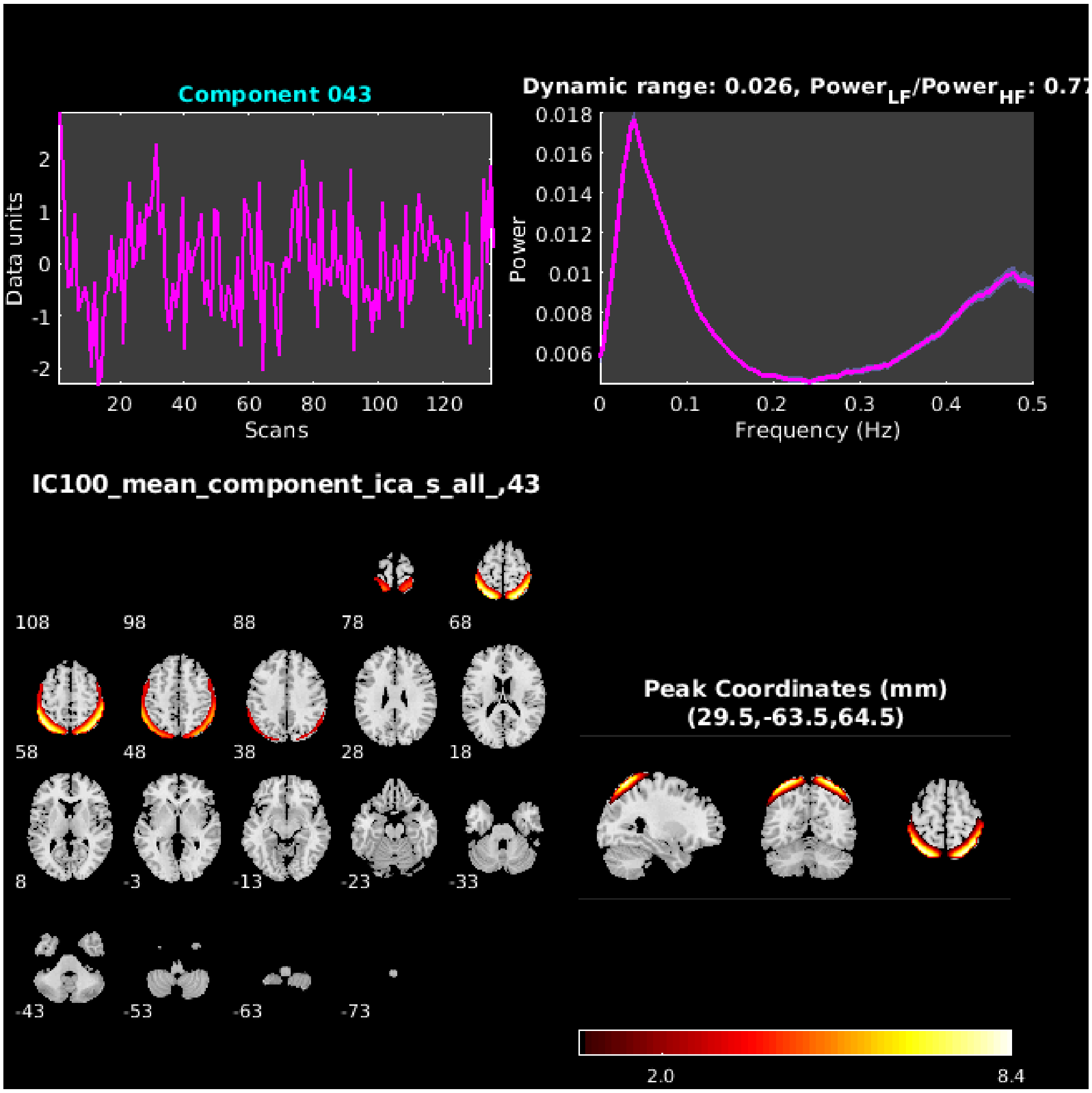
Component 043
Data units
Scans
Dynamic range: 0.026, Power$_{LF}$/Power$_{HF}$: 0.77
Power
Frequency (Hz)
IC100_mean_component_ica_s_all_,43
Peak Coordinates (mm)
(29.5,-63.5,64.5)
2.0
8.4

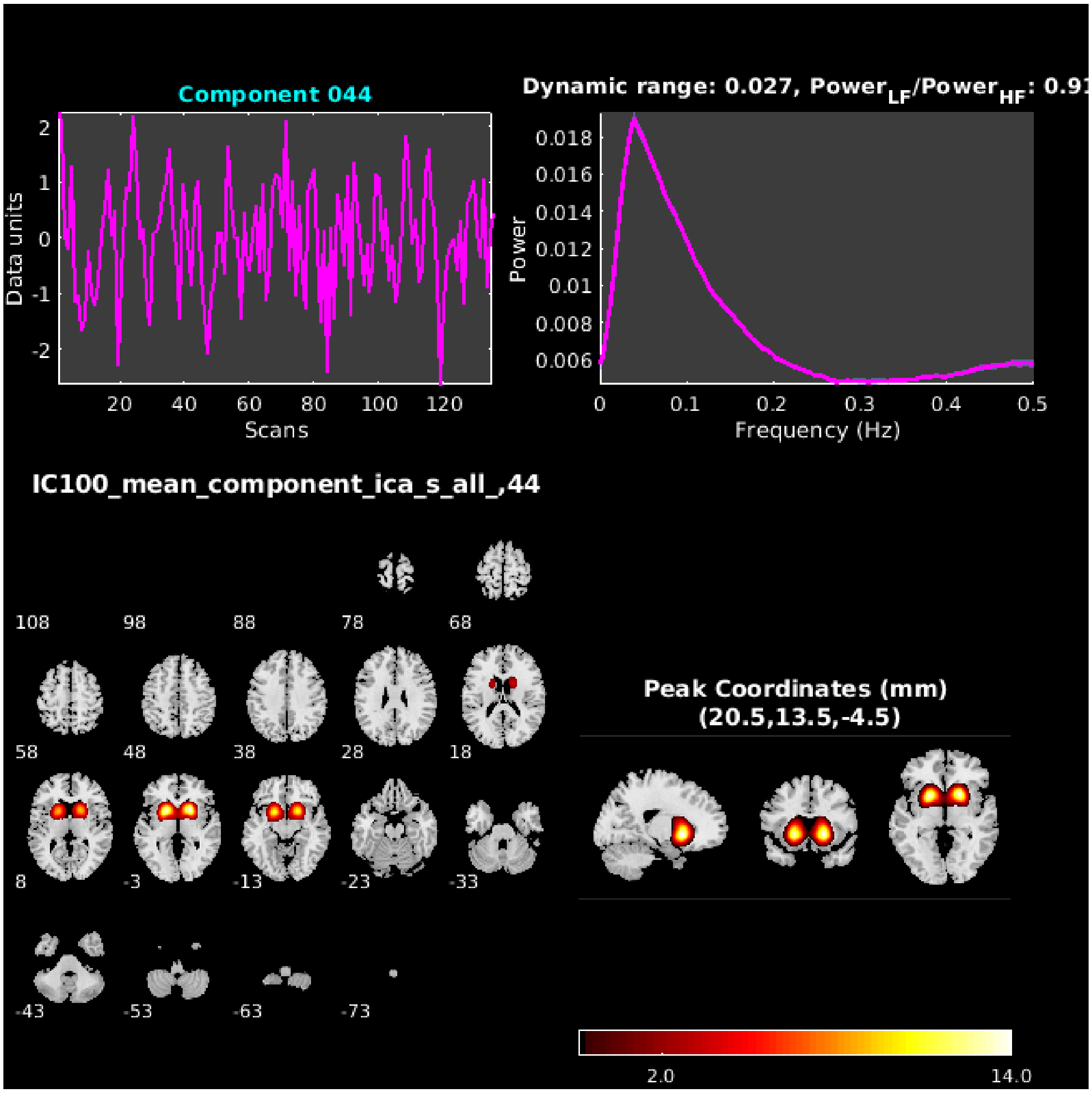
Component 044
Dynamic range: 0.027, Power$_{LF}$/Power$_{HF}$: 0.91
Data units
Scans
Power
Frequency (Hz)
IC100_mean_component_ica_s_all_,44
Peak Coordinates (mm)
(20.5,13.5,-4.5)
2.0
14.0

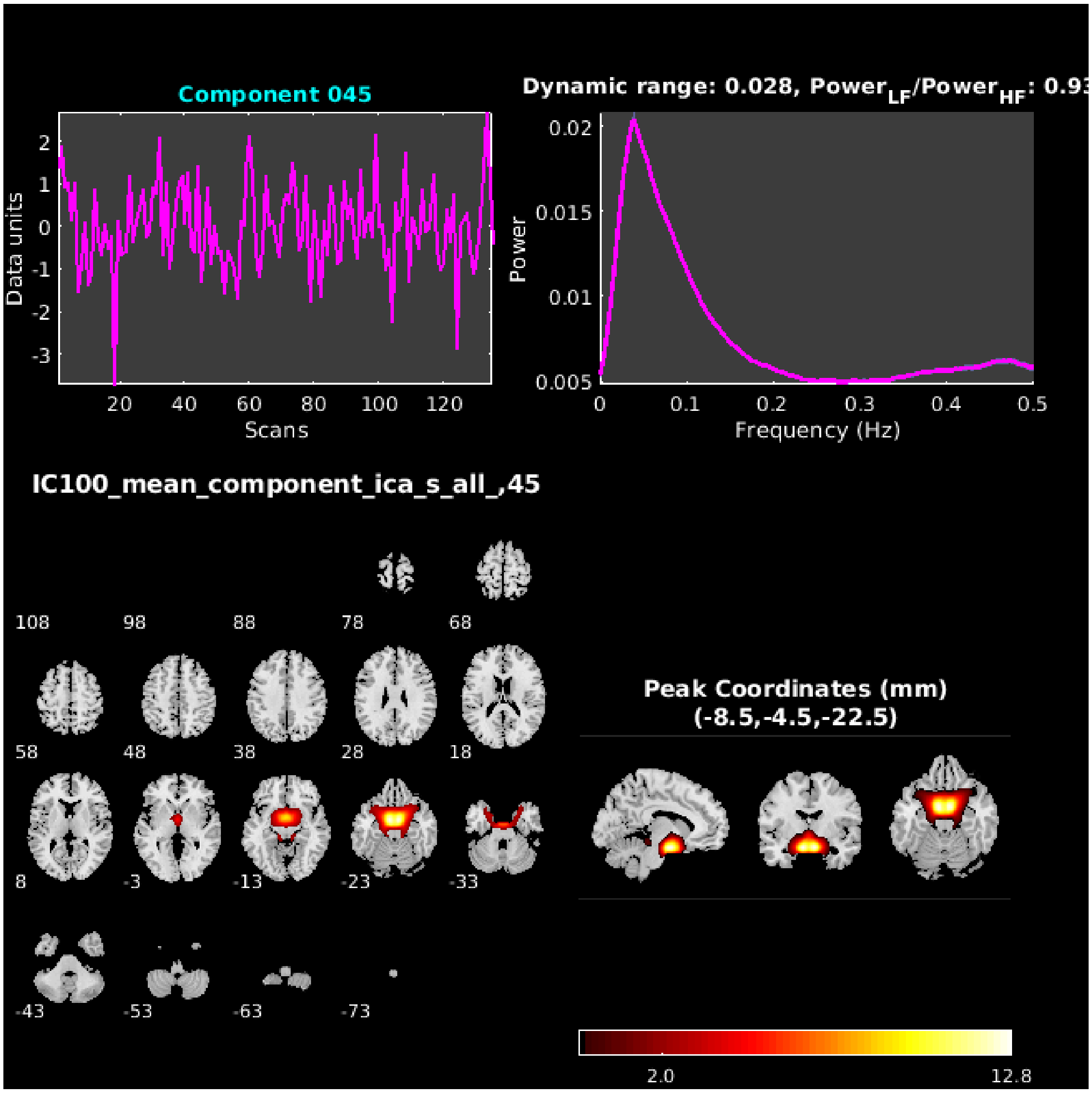
Component 045
Dynamic range: 0.028, Power_LF/Power_HF: 0.93
Data units
Scans
Power
Frequency (Hz)
IC100_mean_component_ica_s_all_,45
108
98
88
78
68
58
48
38
28
18
8
-3
-13
-23
-33
-43
-53
-63
-73
Peak Coordinates (mm)
(-8.5,-4.5,-22.5)
2.0
12.8

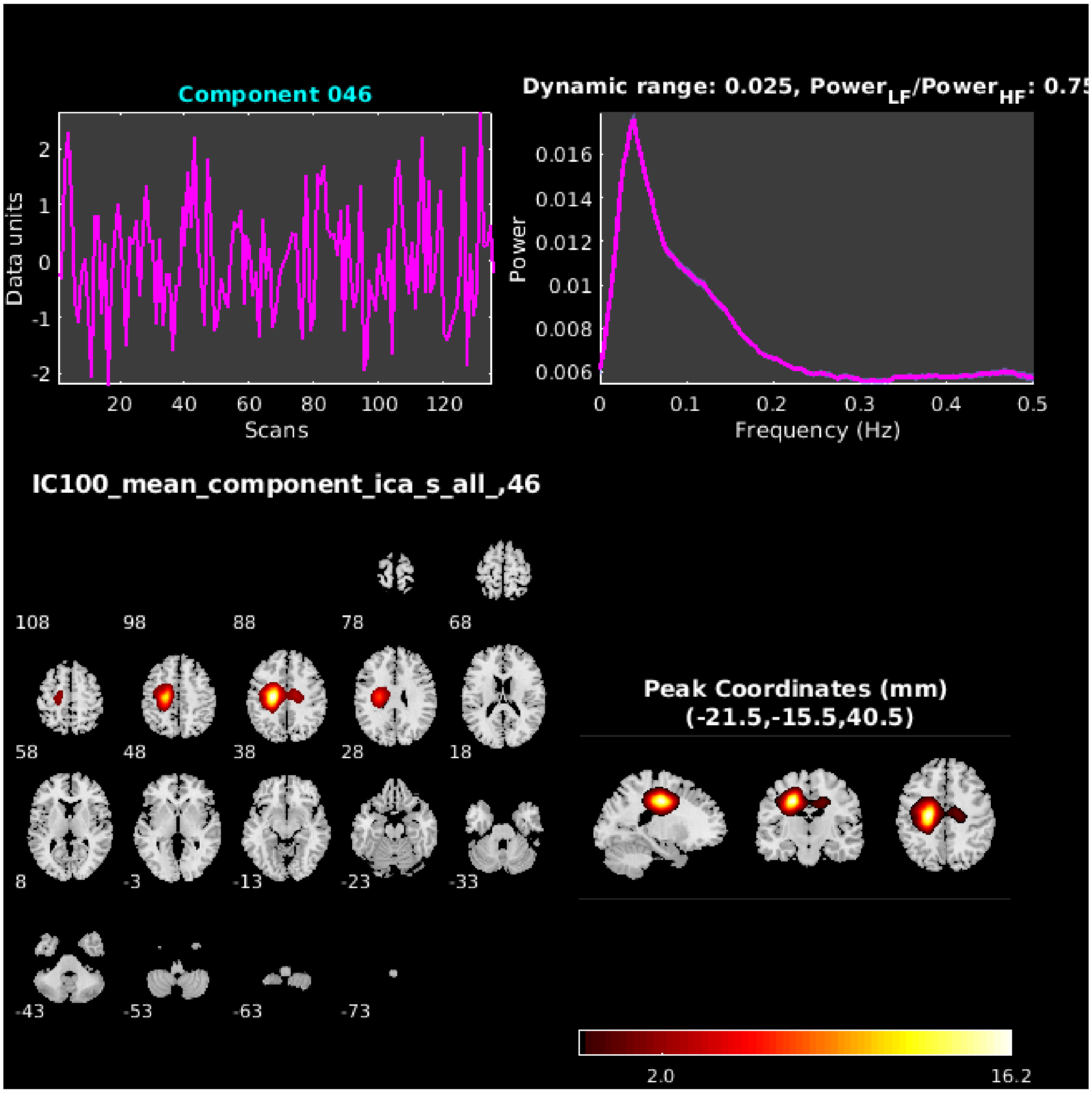
Component 046
Dynamic range: 0.025, Power$_{LF}$/Power$_{HF}$: 0.75
Data units
Scans
Power
Frequency (Hz)
IC100_mean_component_ica_s_all_,46
Peak Coordinates (mm)
(-21.5,-15.5,40.5)
2.0
16.2

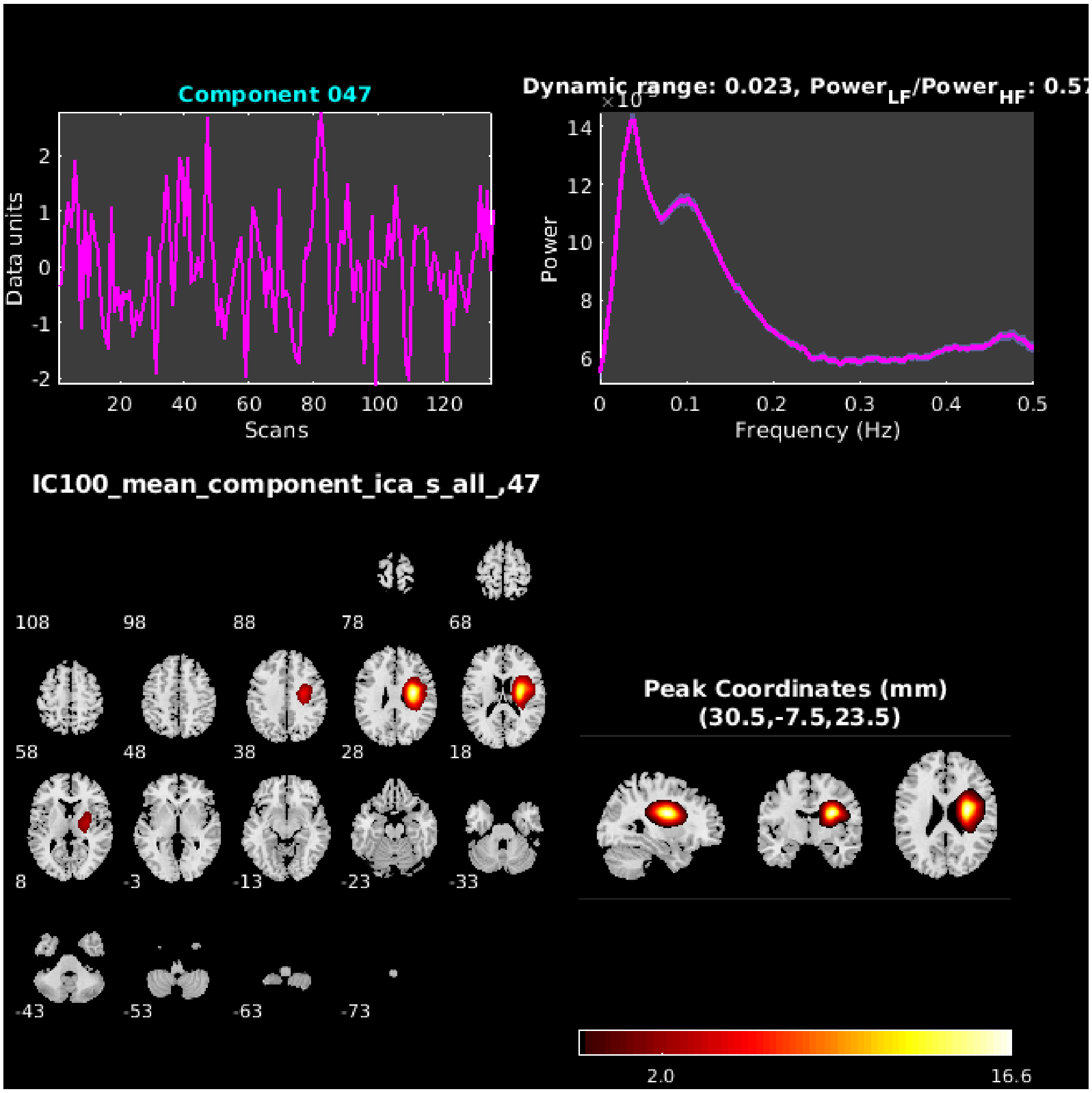
Component 047
Dynamic range: 0.023, Power_LF/Power_HF: 0.57
Data units
Scans
Power
Frequency (Hz)
IC100_mean_component_ica_s_all_,47
Peak Coordinates (mm)
(30.5,-7.5,23.5)
2.0
16.6

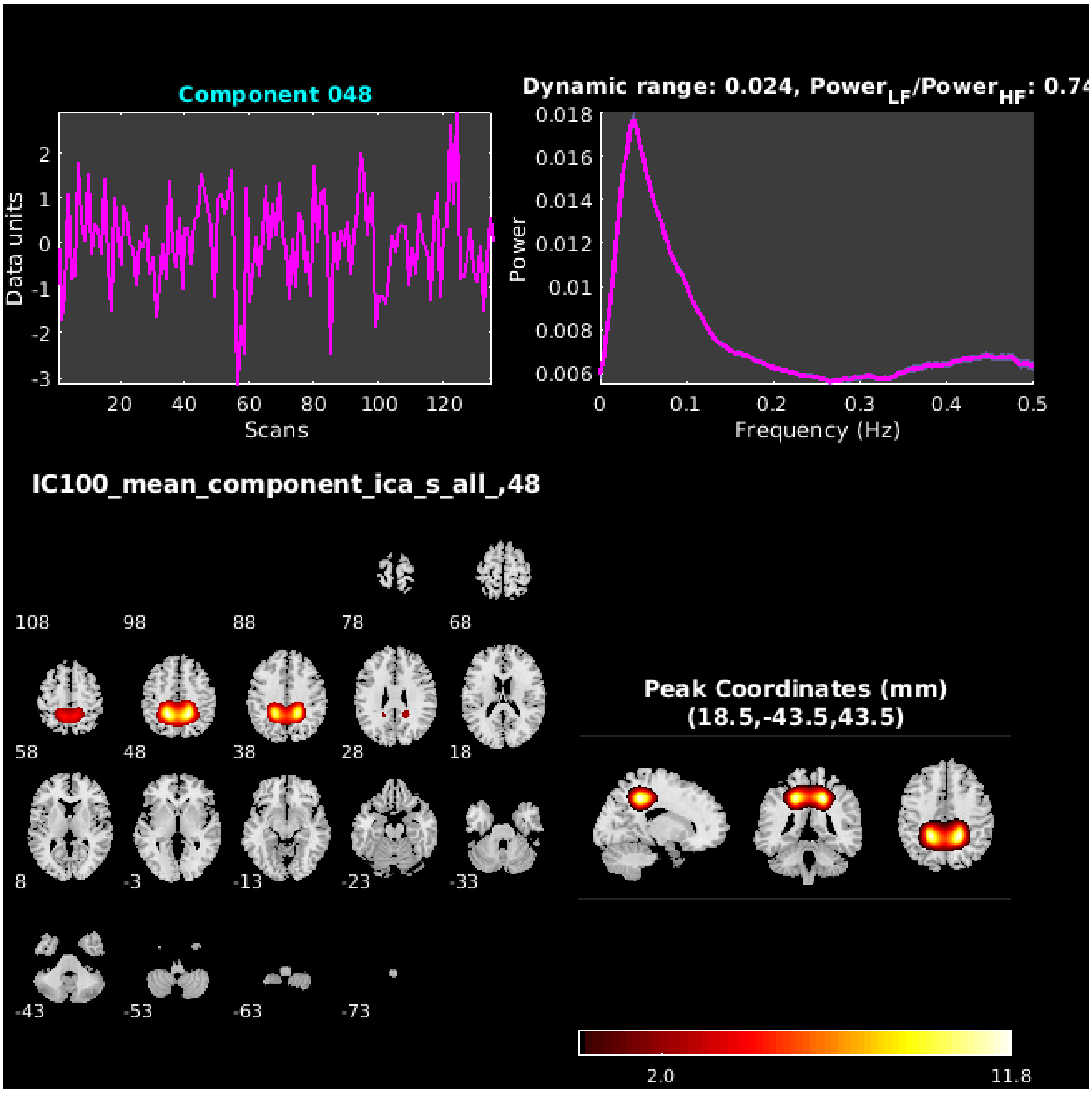

Component 048
Dynamic range: 0.024, Power$_{LF}$/Power$_{HF}$: 0.74
Data units
Scans
Power
Frequency (Hz)
IC100_mean_component_ica_s_all_,48
Peak Coordinates (mm)
(18.5,-43.5,43.5)
2.0
11.8

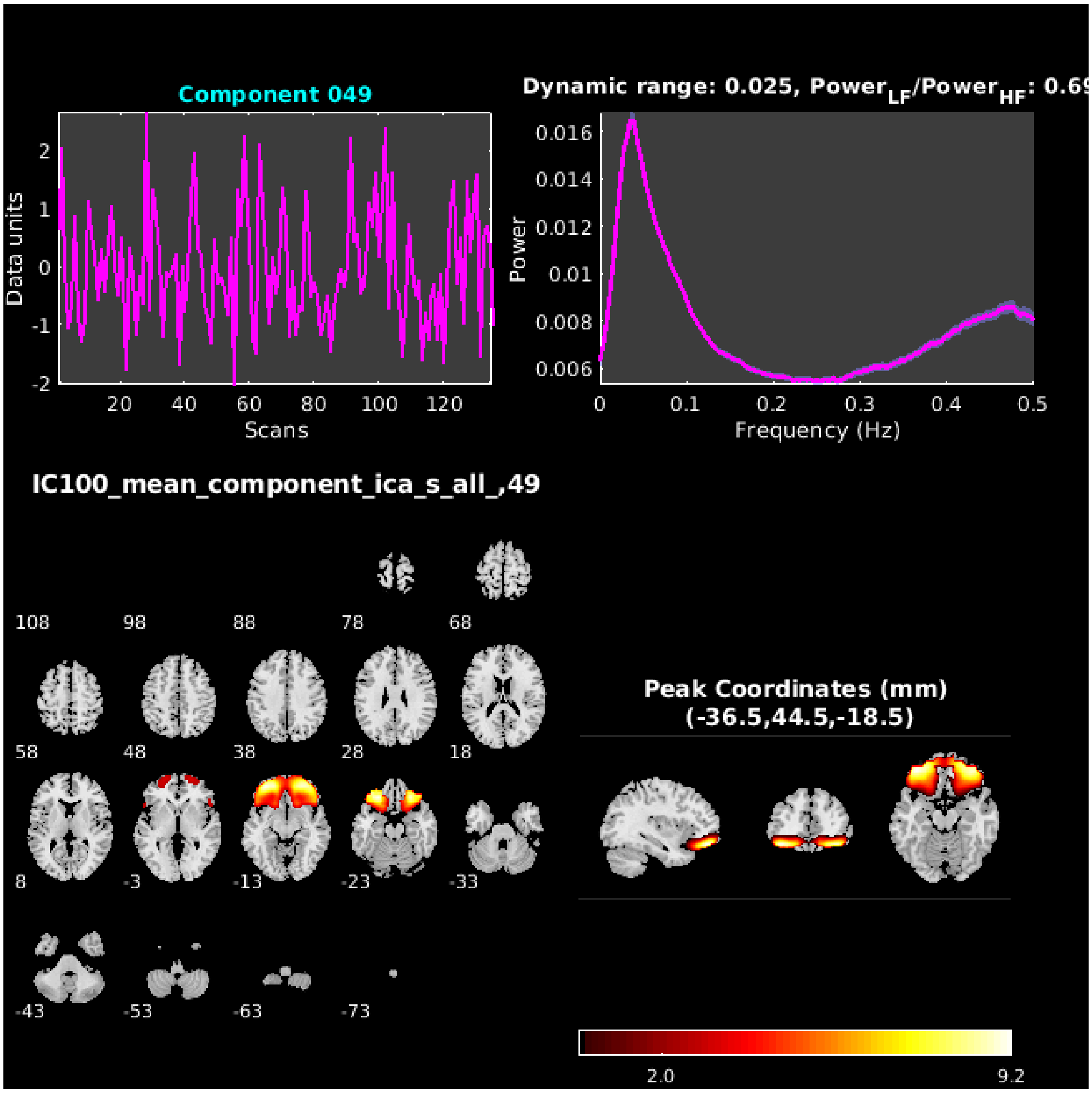
Component 049
Dynamic range: 0.025, Power$_{LF}$/Power$_{HF}$: 0.69
Data units
Scans
Power
Frequency (Hz)
IC100_mean_component_ica_s_all_,49
Peak Coordinates (mm)
(-36.5,44.5,-18.5)
2.0
9.2

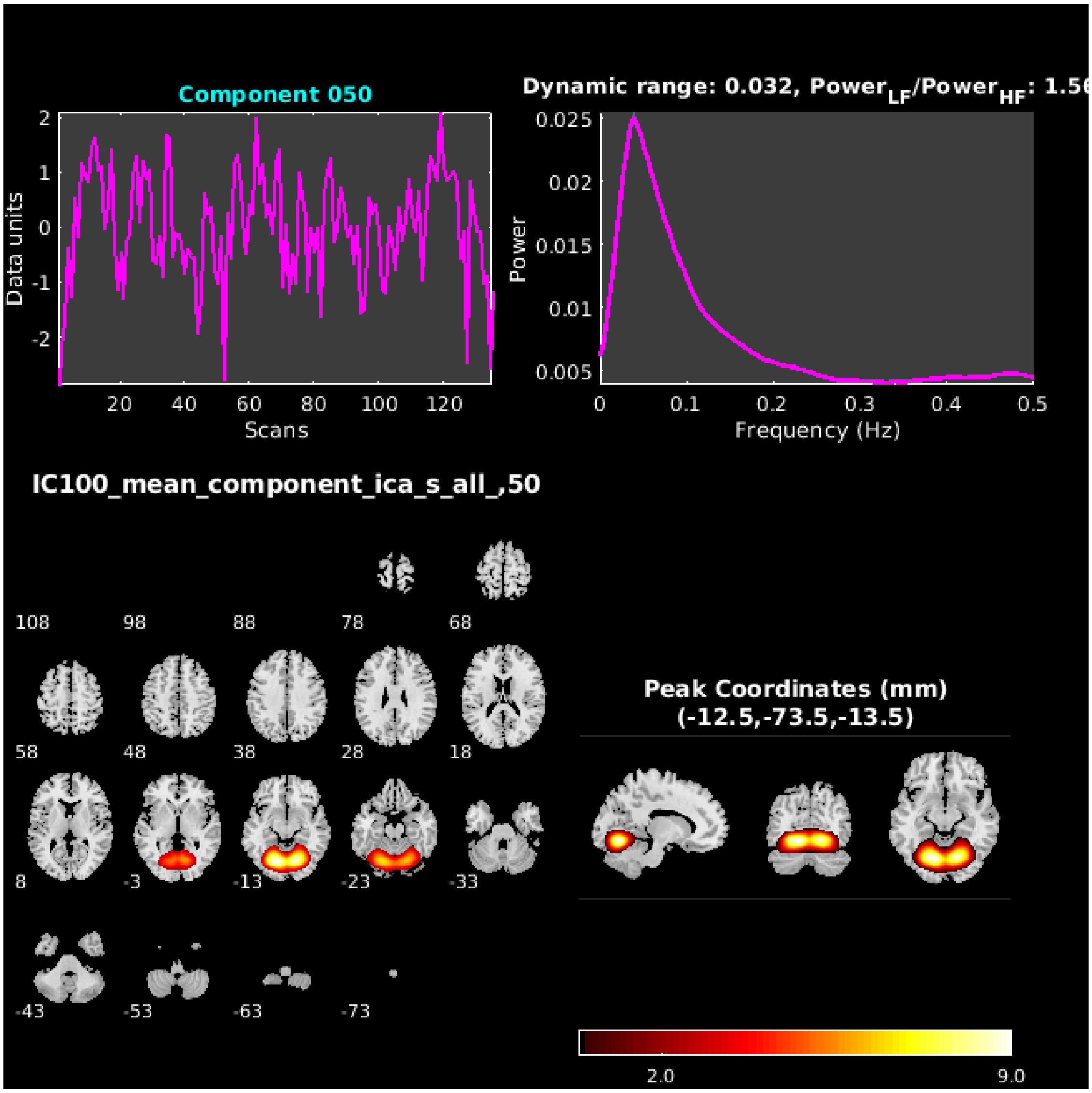

Component 050
Data units
Scans
Dynamic range: 0.032, Power$_{LF}$/Power$_{HF}$: 1.56
Power
Frequency (Hz)
IC100_mean_component_ica_s_all_,50
Peak Coordinates (mm)
(-12.5,-73.5,-13.5)
2.0
9.0

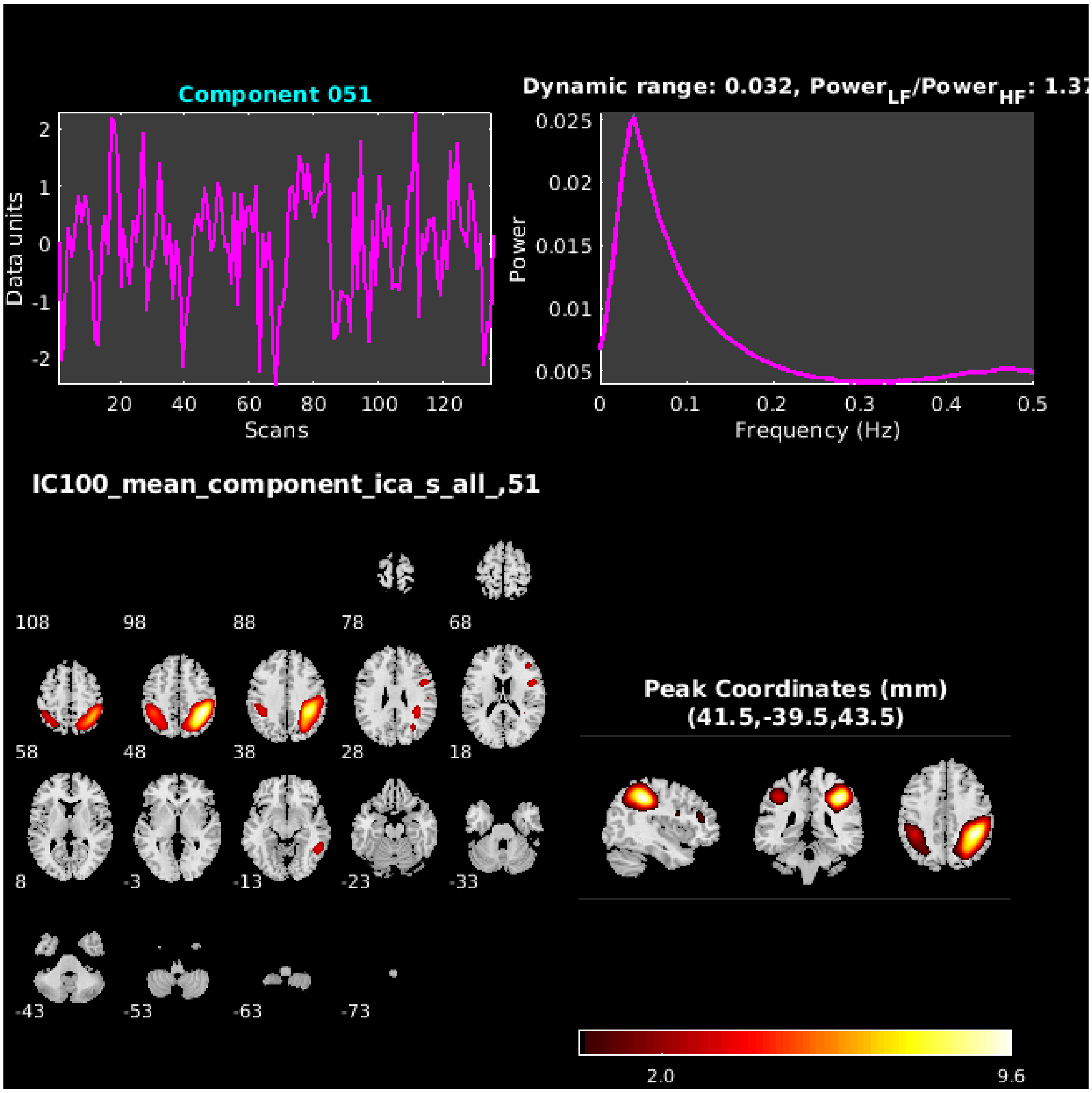

Component 051
Dynamic range: 0.032, Power$_{LF}$/Power$_{HF}$: 1.37
Data units
Scans
Power
Frequency (Hz)
IC100_mean_component_ica_s_all_,51
Peak Coordinates (mm)
(41.5,-39.5,43.5)
2.0
9.6

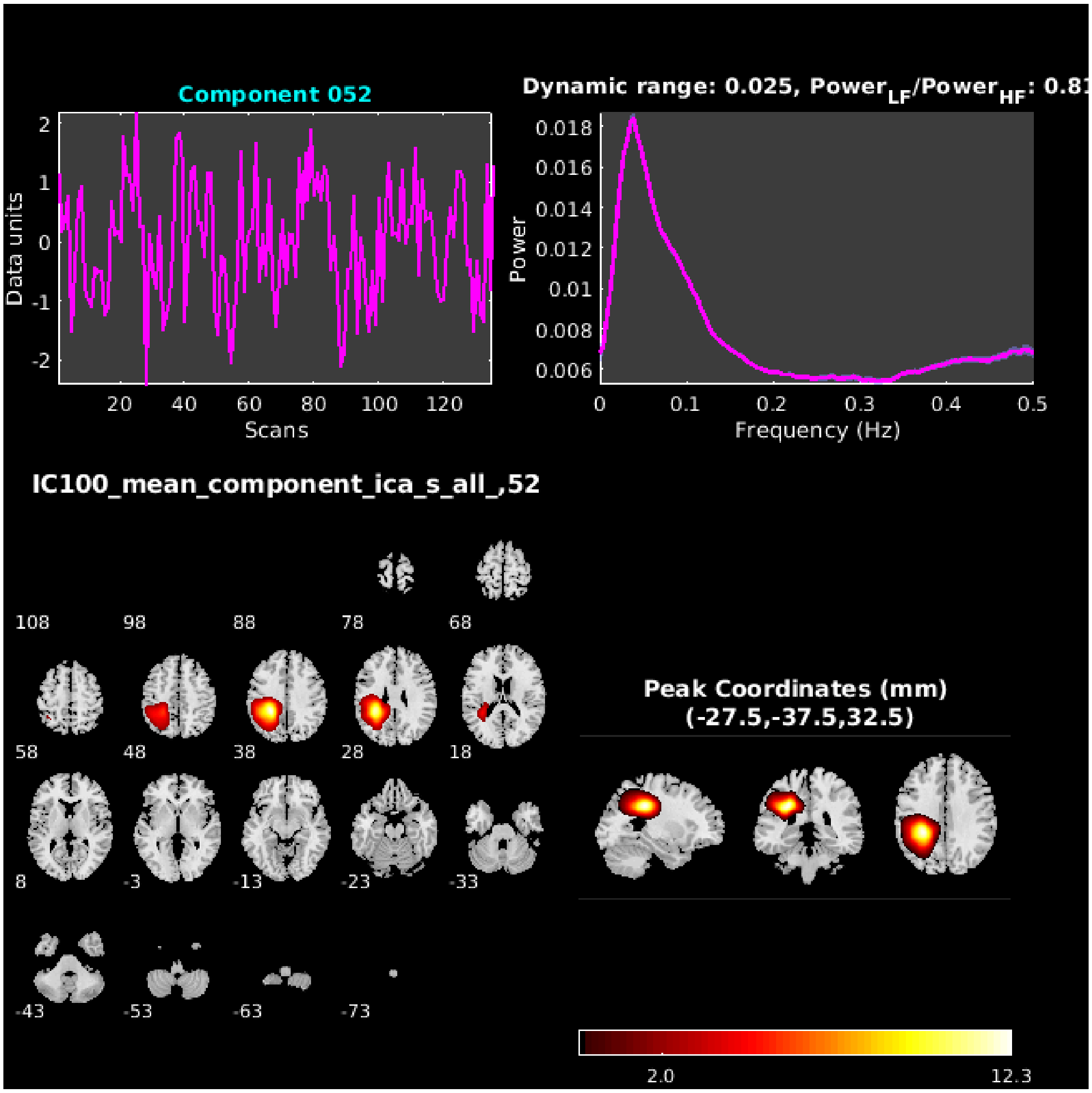

Component 052
Dynamic range: 0.025, Power$_{LF}$/Power$_{HF}$: 0.81
Data units
Scans
Power
Frequency (Hz)
IC100_mean_component_ica_s_all_,52
Peak Coordinates (mm)
(-27.5,-37.5,32.5)
2.0
12.3

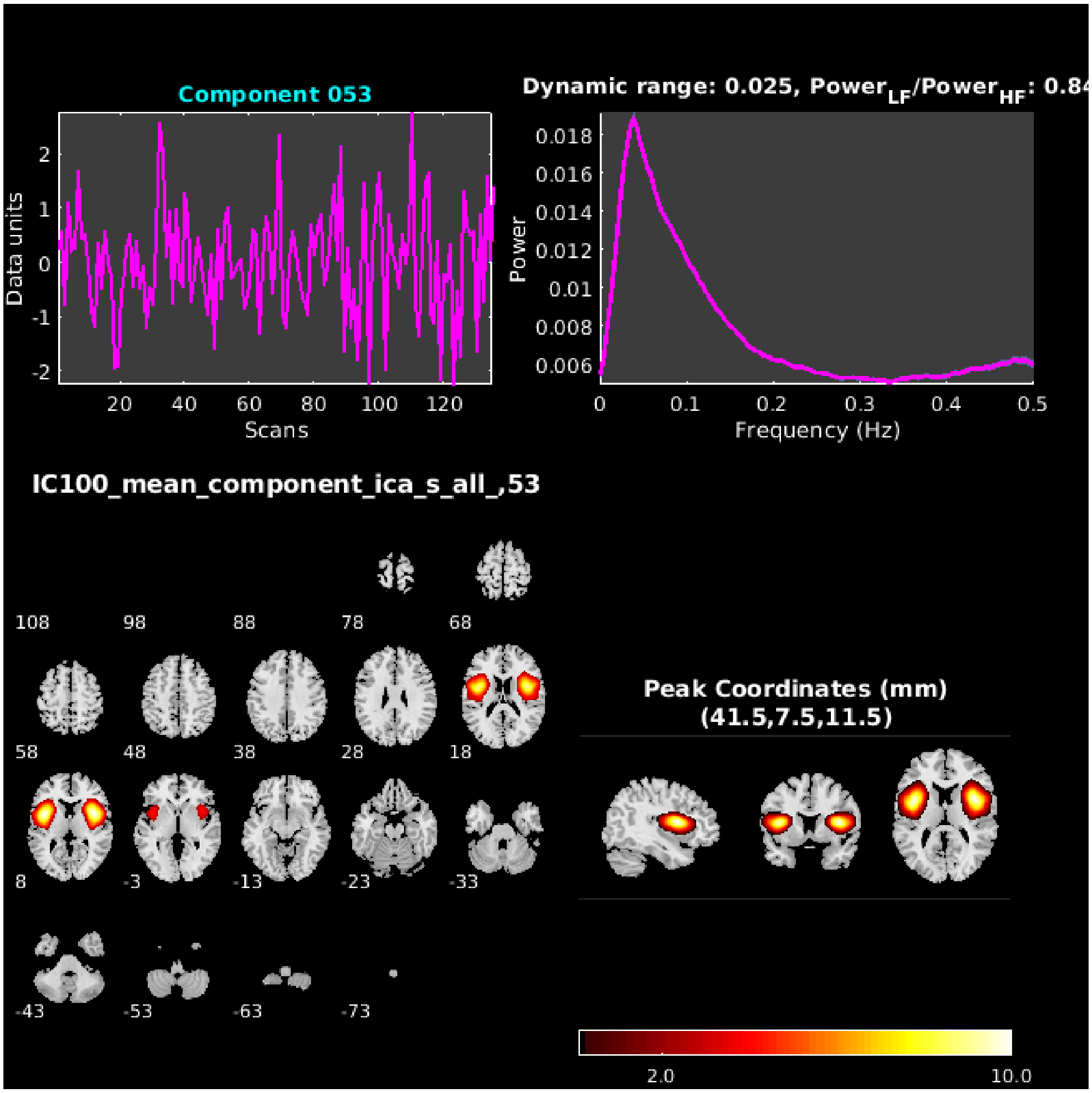
Component 053
Dynamic range: 0.025, Power$_{LF}$/Power$_{HF}$: 0.84
Data units
Scans
Power
Frequency (Hz)
IC100_mean_component_ica_s_all_,53
108
98
88
78
68
58
48
38
28
18
8
-3
-13
-23
-33
-43
-53
-63
-73
Peak Coordinates (mm)
(41.5,7.5,11.5)
2.0
10.0

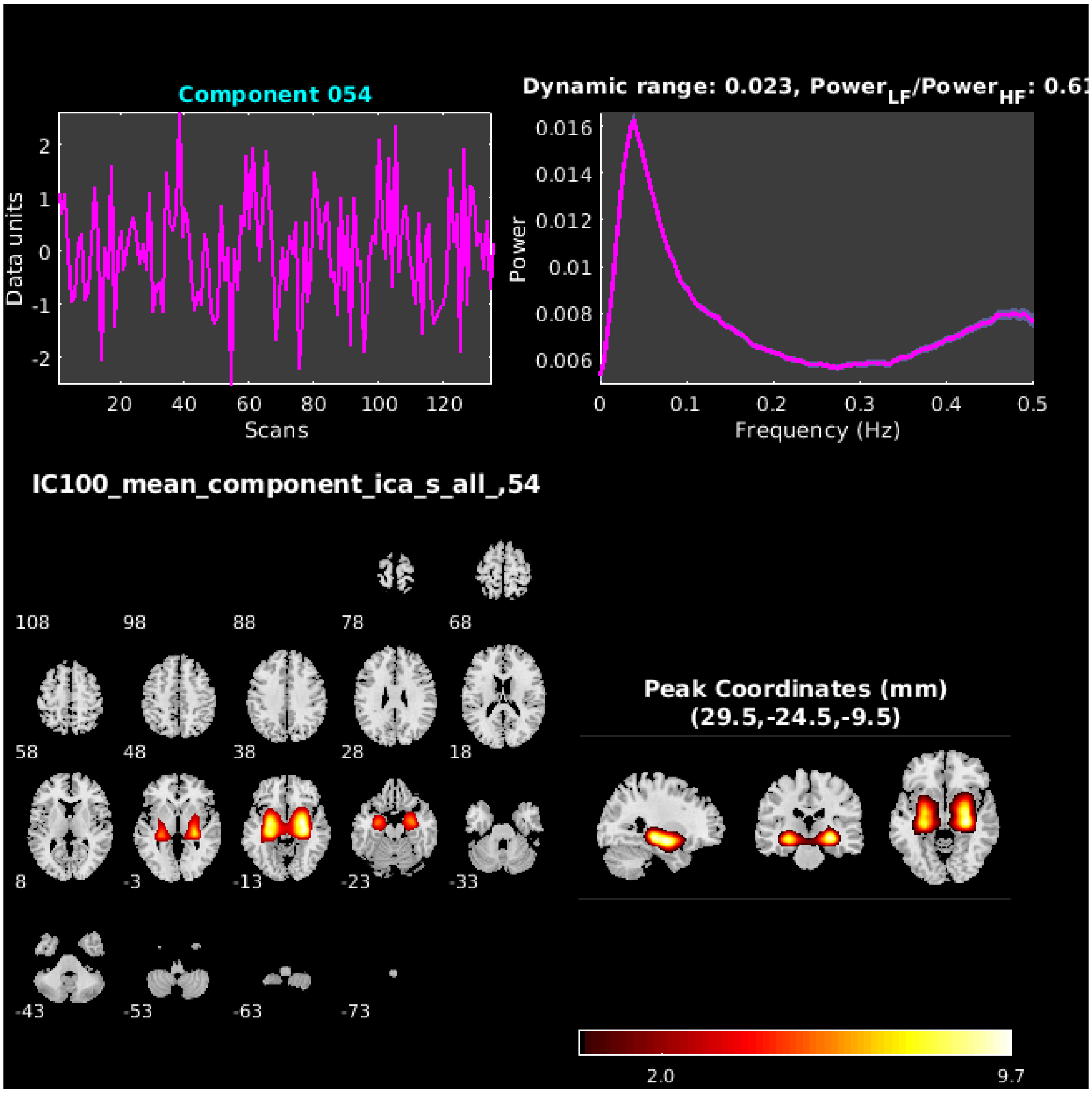

Component 054
Dynamic range: 0.023, Power$_{LF}$/Power$_{HF}$: 0.61
Data units
Scans
Power
Frequency (Hz)
IC100_mean_component_ica_s_all_,54
Peak Coordinates (mm)
(29.5,-24.5,-9.5)
2.0
9.7

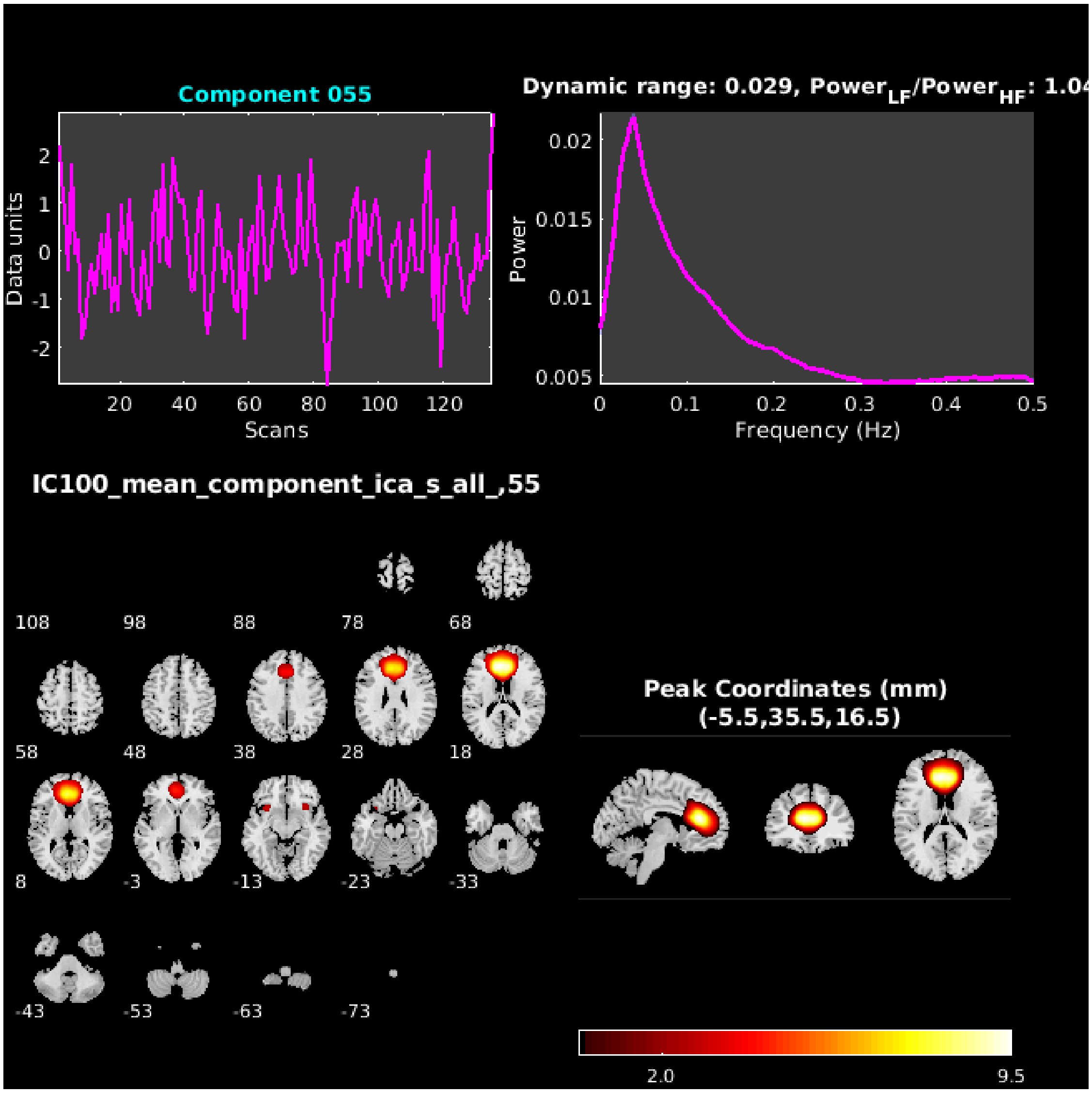

Component 055
Dynamic range: 0.029, Power$_{LF}$/Power$_{HF}$: 1.04
Data units
Scans
Power
Frequency (Hz)
IC100_mean_component_ica_s_all_,55
Peak Coordinates (mm)
(-5.5,35.5,16.5)
2.0
9.5

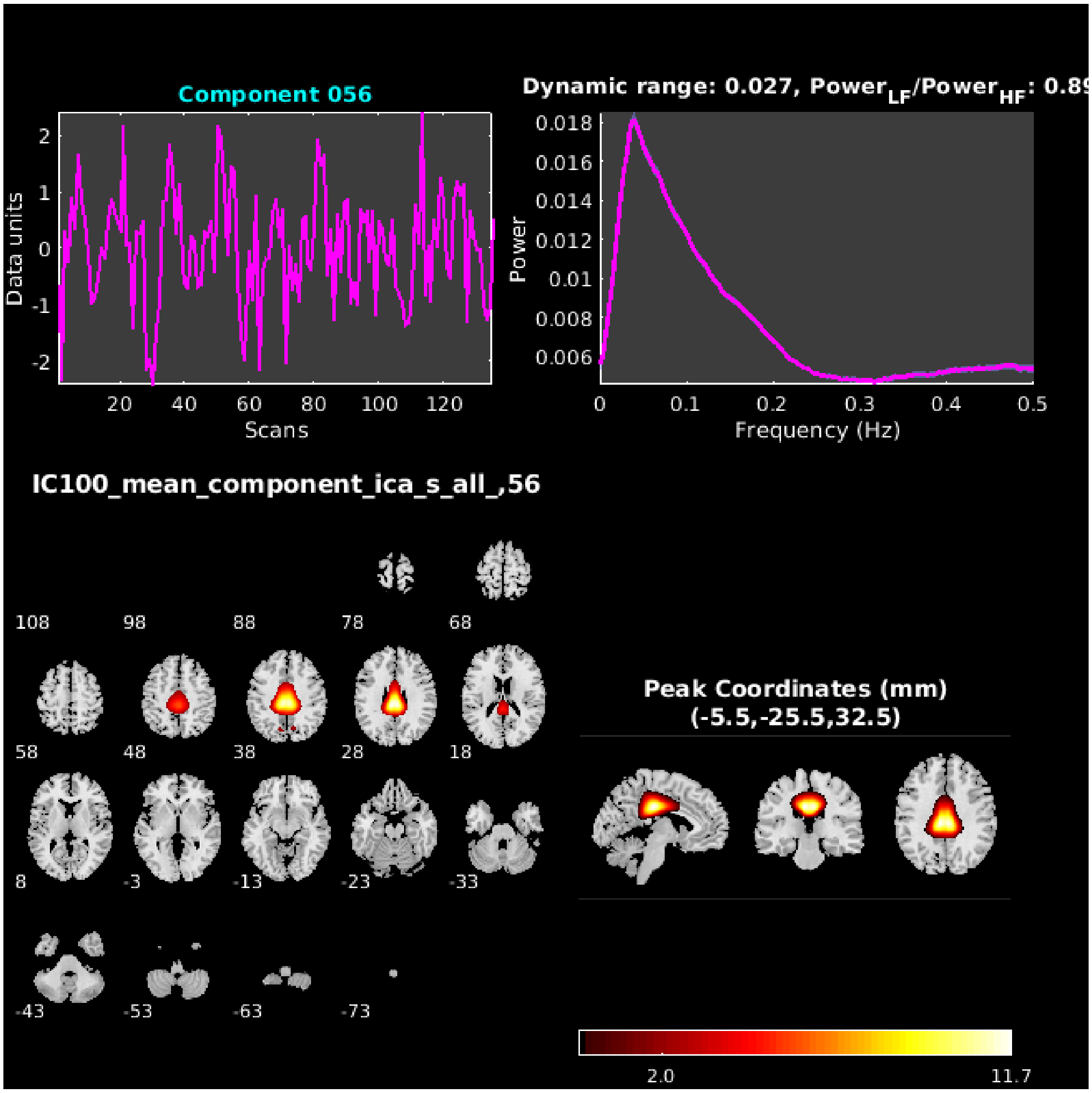
Component 056
Dynamic range: 0.027, Power_LF/Power_HF: 0.8
Data units
Scans
Power
Frequency (Hz)
IC100_mean_component_ica_s_all_,56
Peak Coordinates (mm)
(-5.5,-25.5,32.5)
2.0
11.7

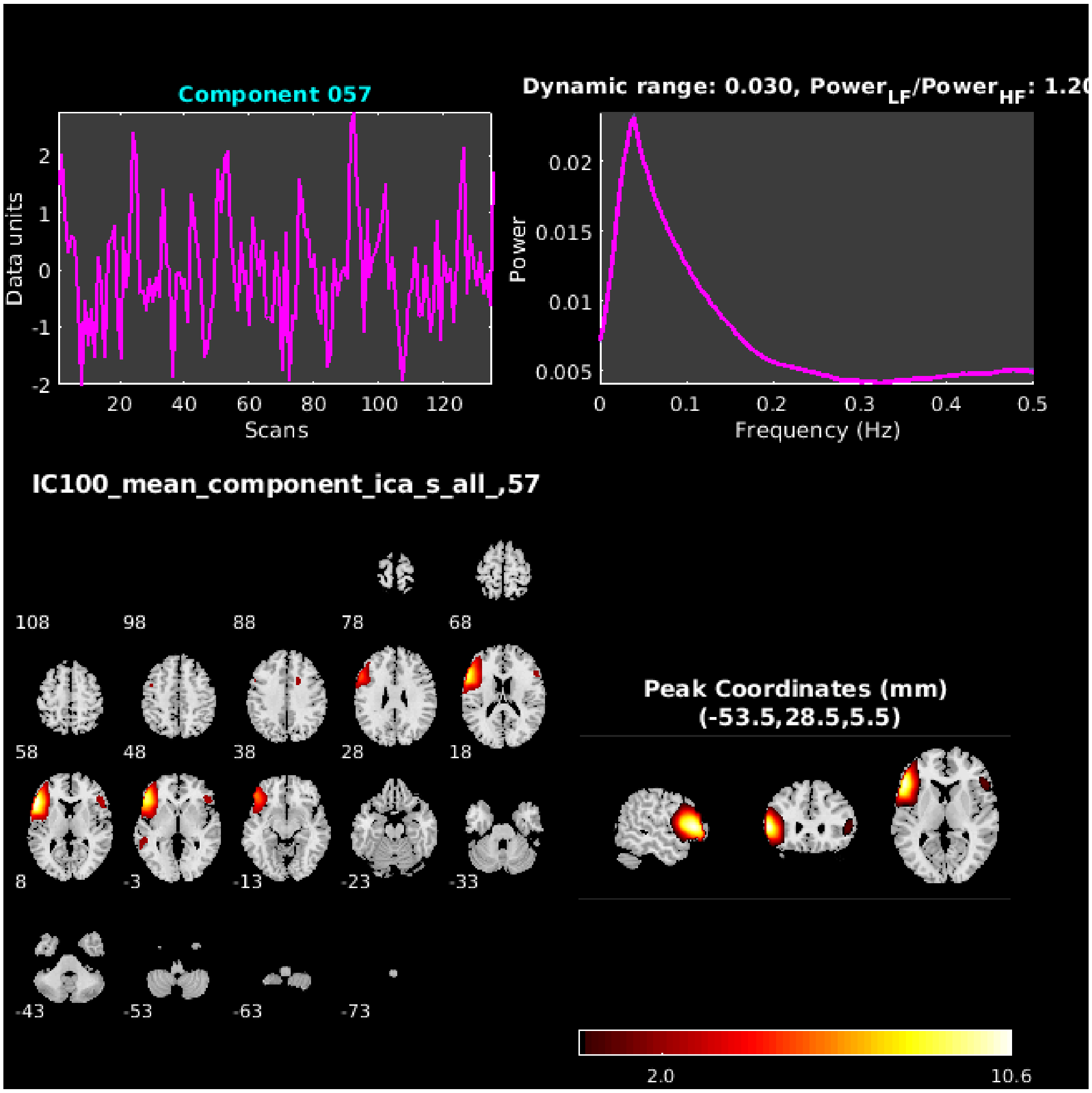

Component 057
Dynamic range: 0.030, Power$_{LF}$/Power$_{HF}$: 1.20
Data units
Scans
Power
Frequency (Hz)
IC100_mean_component_ica_s_all_,57
Peak Coordinates (mm)
(-53.5,28.5,5.5)

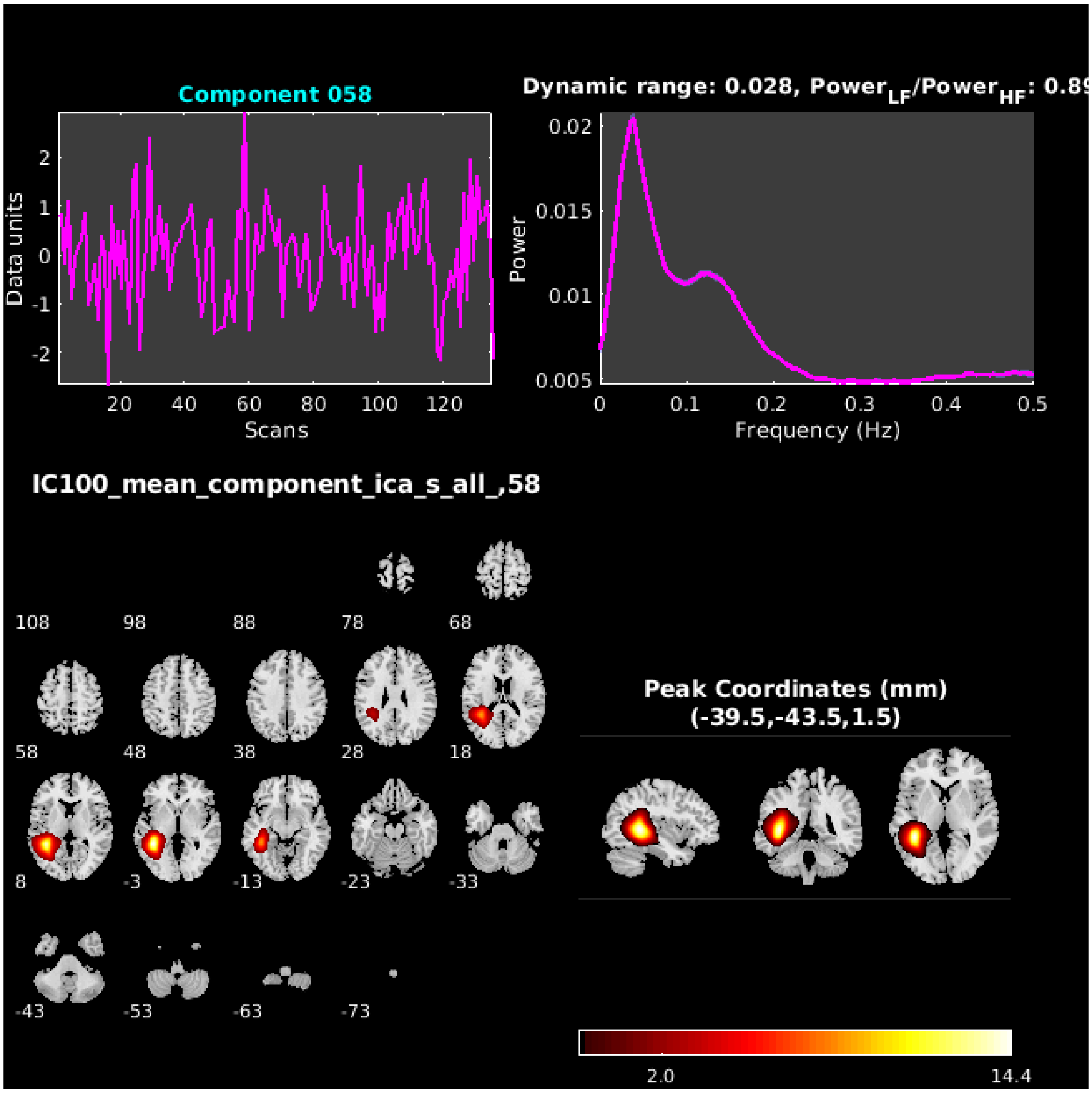
Component 058
Dynamic range: 0.028, Power$_{LF}$/Power$_{HF}$: 0.89
Data units
Scans
Power
Frequency (Hz)
IC100_mean_component_ica_s_all_,58
Peak Coordinates (mm)
(-39.5,-43.5,1.5)
2.0
14.4

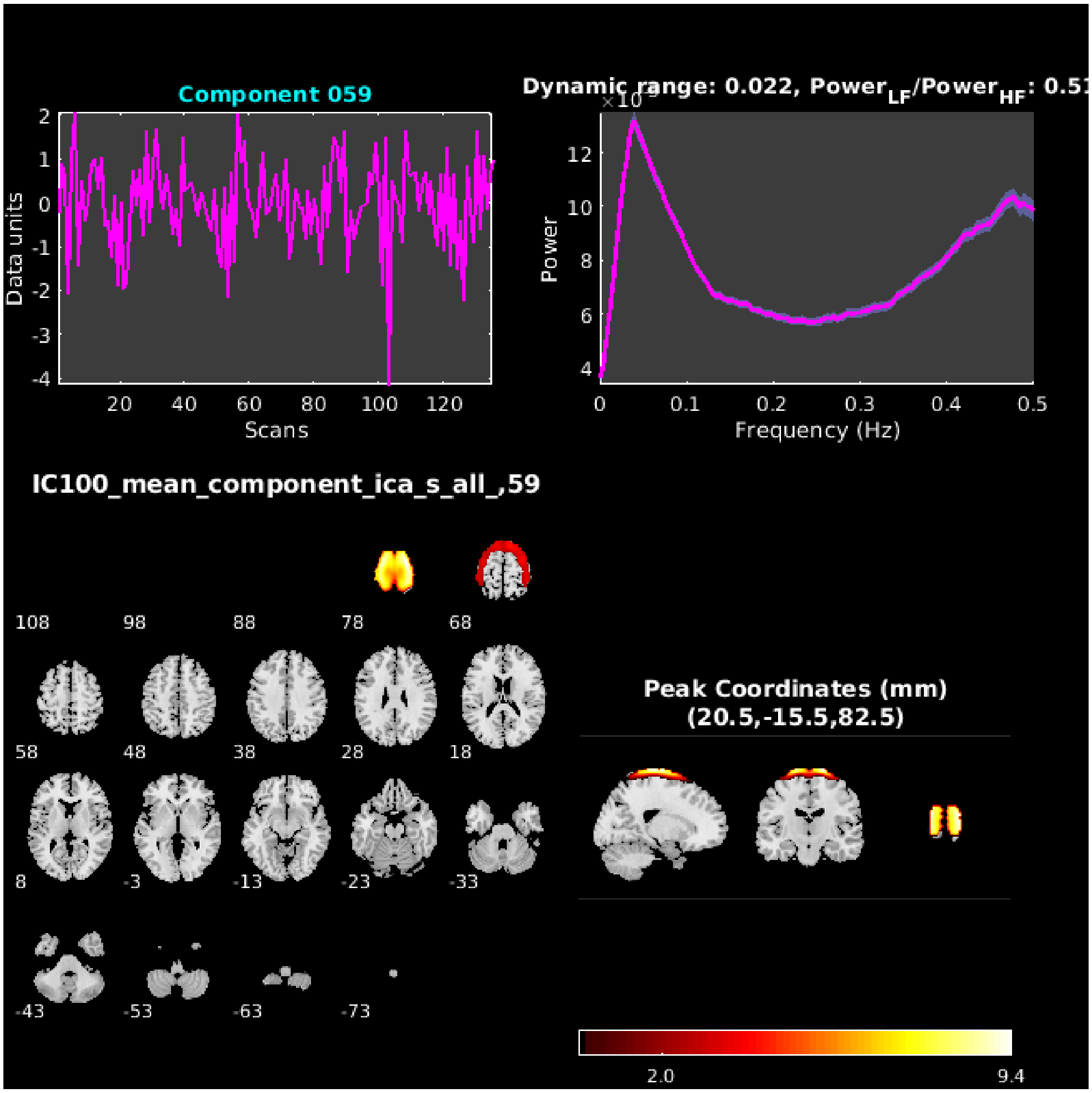
Component 059
Dynamic range: 0.022, Power$_{LF}$/Power$_{HF}$: 0.51
Data units
Scans
Power
Frequency (Hz)
IC100_mean_component_ica_s_all_,59
Peak Coordinates (mm)
(20.5,-15.5,82.5)
2.0
9.4

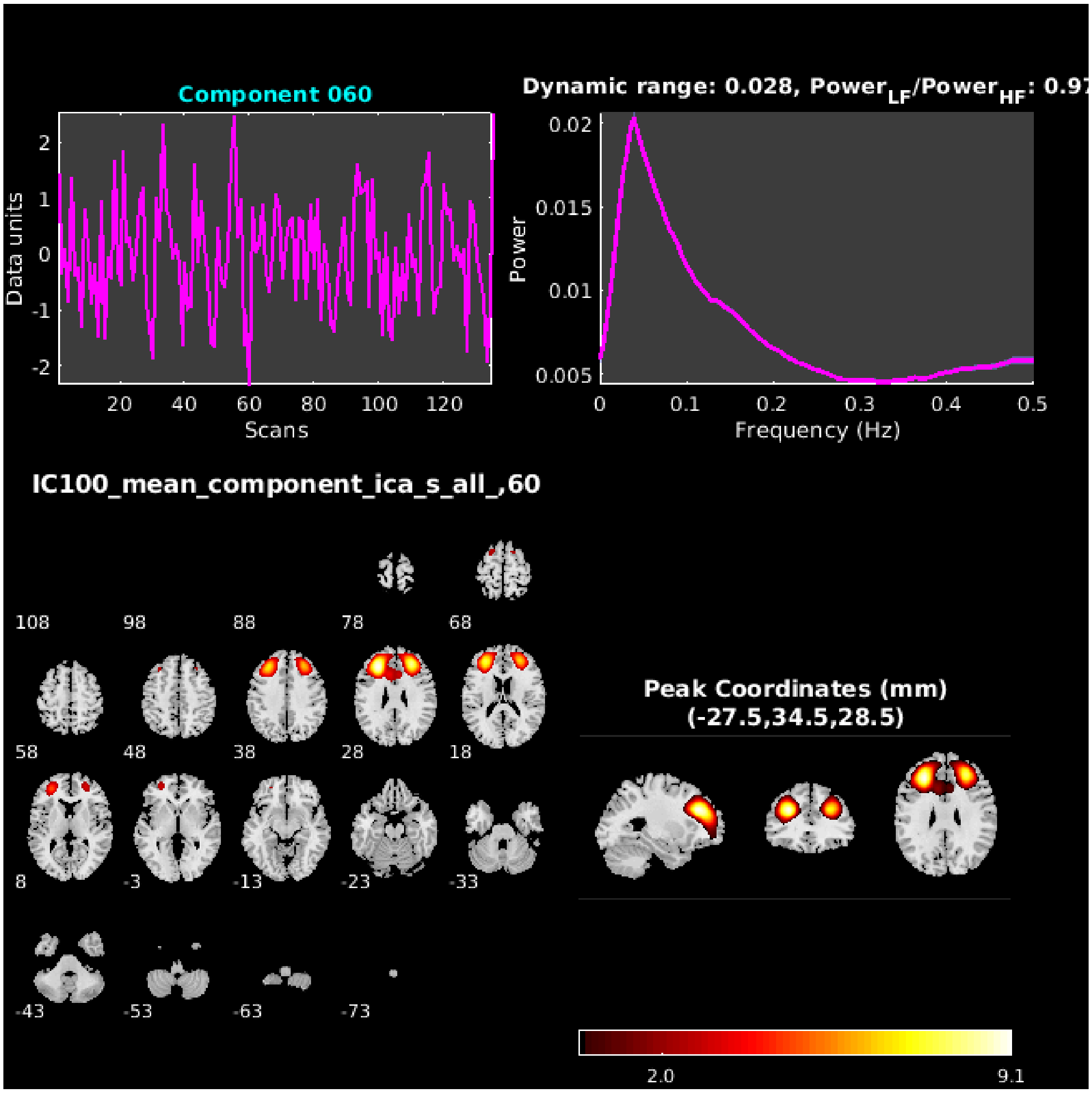

Component 060
Dynamic range: 0.028, Power_LF/Power_HF: 0.97
Data units
Scans
Power
Frequency (Hz)
IC100_mean_component_ica_s_all_,60
108
98
88
78
68
58
48
38
28
18
8
-3
-13
-23
-33
-43
-53
-63
-73
Peak Coordinates (mm)
(-27.5,34.5,28.5)
2.0
9.1

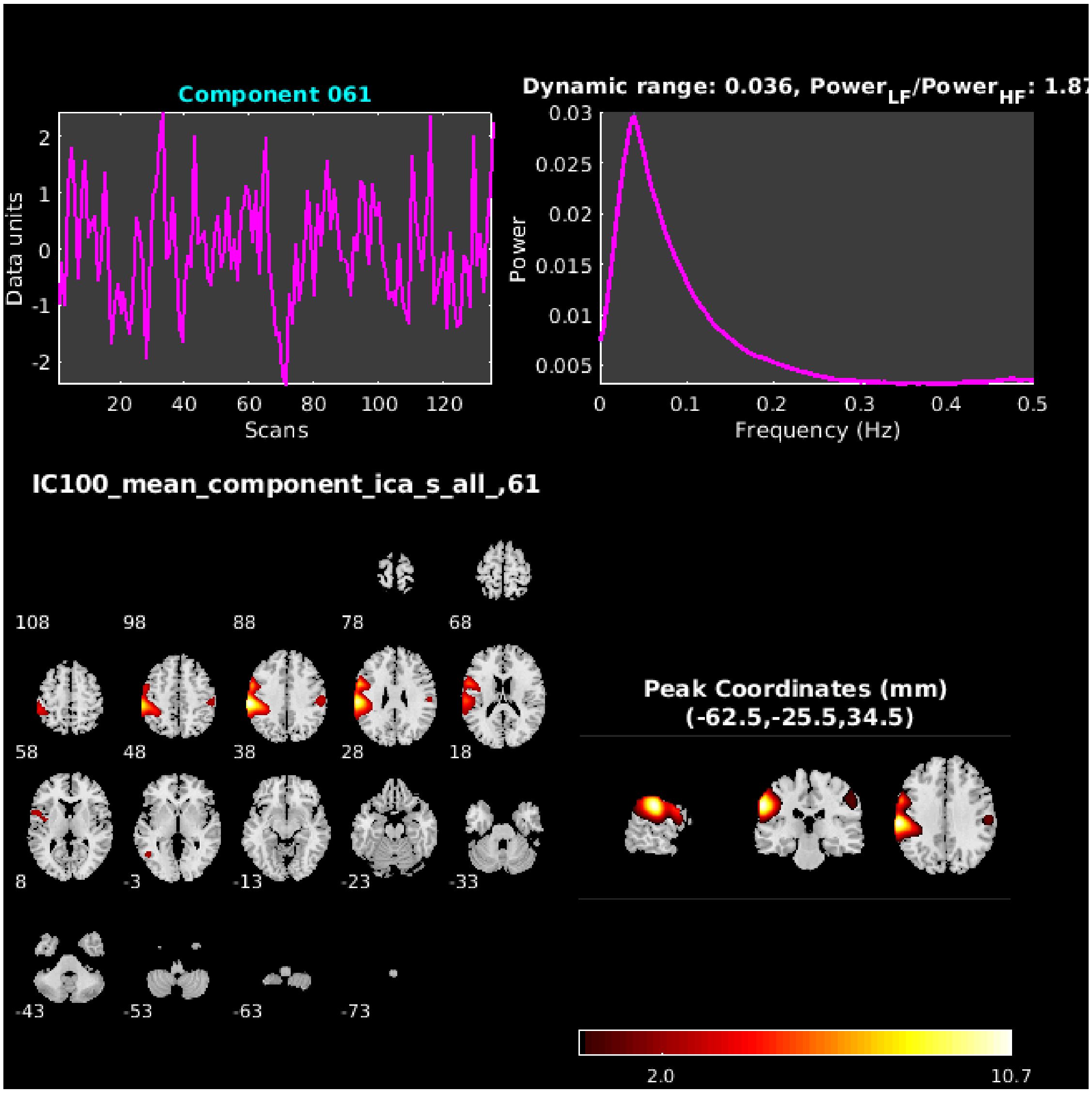

Component 061
Dynamic range: 0.036, Power$_{LF}$/Power$_{HF}$: 1.87
Data units
Scans
Power
Frequency (Hz)
IC100_mean_component_ica_s_all_,61
Peak Coordinates (mm)
(-62.5,-25.5,34.5)
2.0
10.7

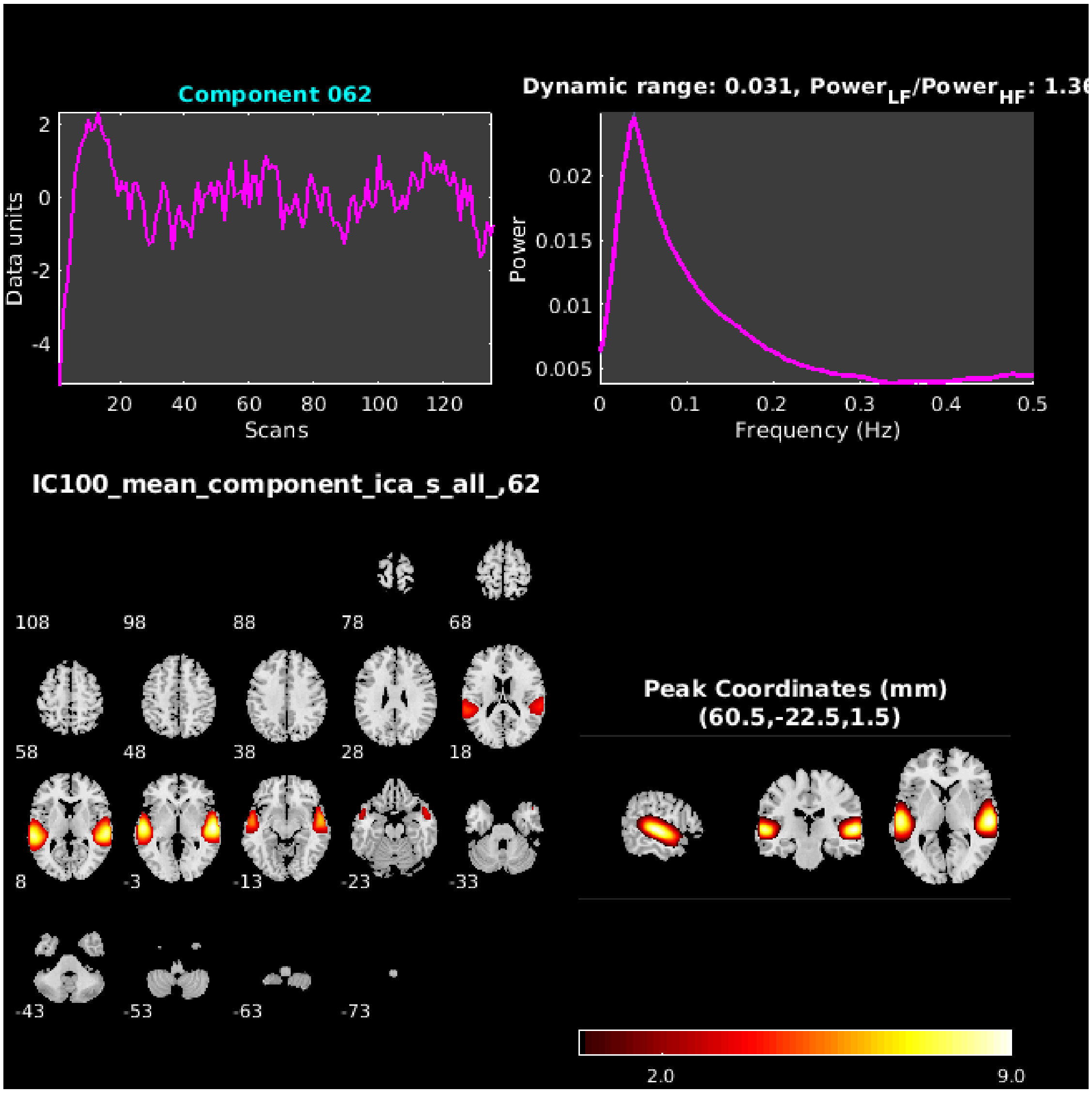

Component 062
Dynamic range: 0.031, Power$_{LF}$/Power$_{HF}$: 1.36
Data units
Scans
Power
Frequency (Hz)
IC100_mean_component_ica_s_all_,62
Peak Coordinates (mm)
(60.5,-22.5,1.5)
2.0
9.0

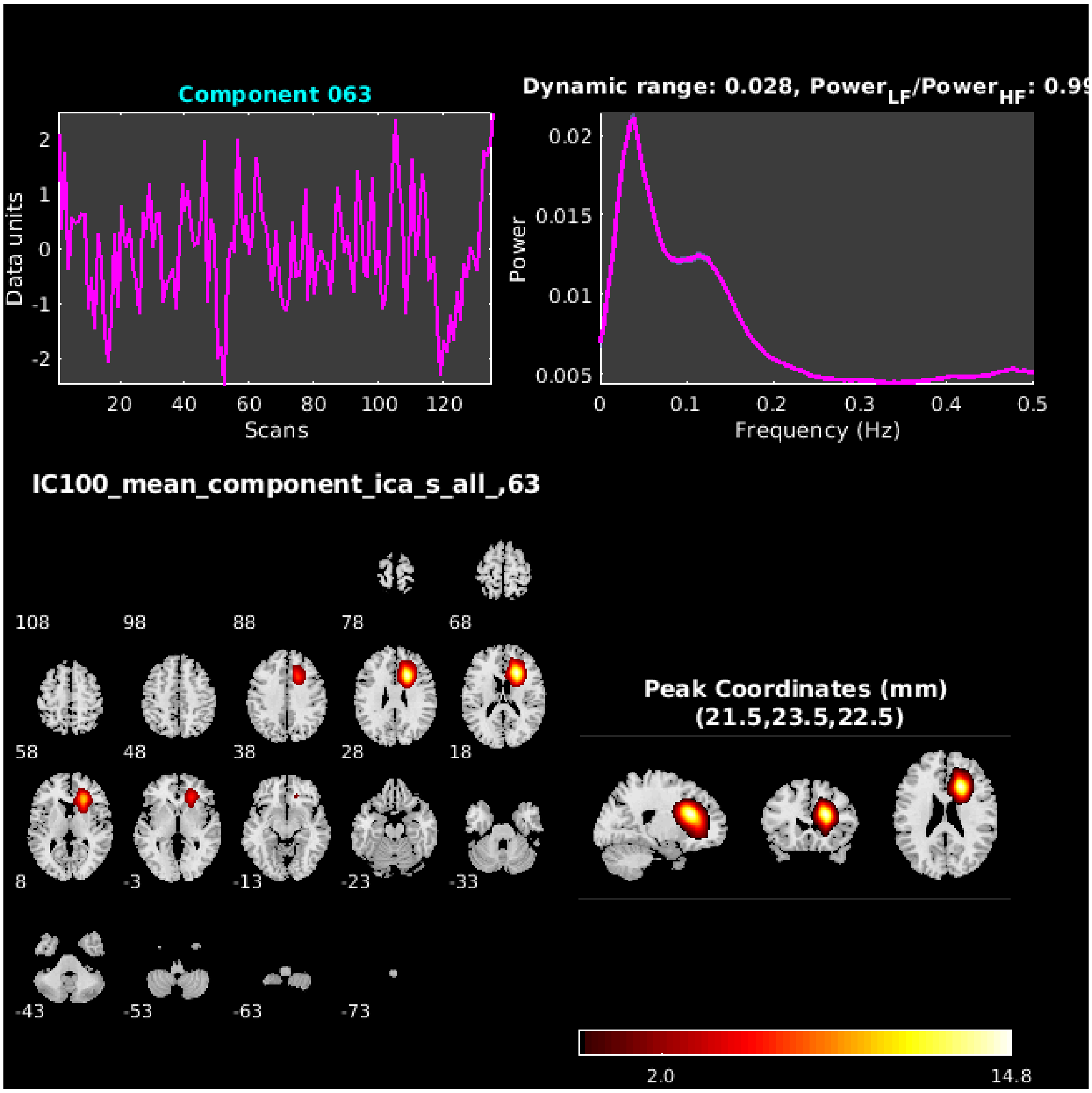
Component 063
Dynamic range: 0.028, Power$_{LF}$/Power$_{HF}$: 0.99
Data units
Scans
Power
Frequency (Hz)
IC100_mean_component_ica_s_all_,63
Peak Coordinates (mm)
(21.5,23.5,22.5)
2.0
14.8

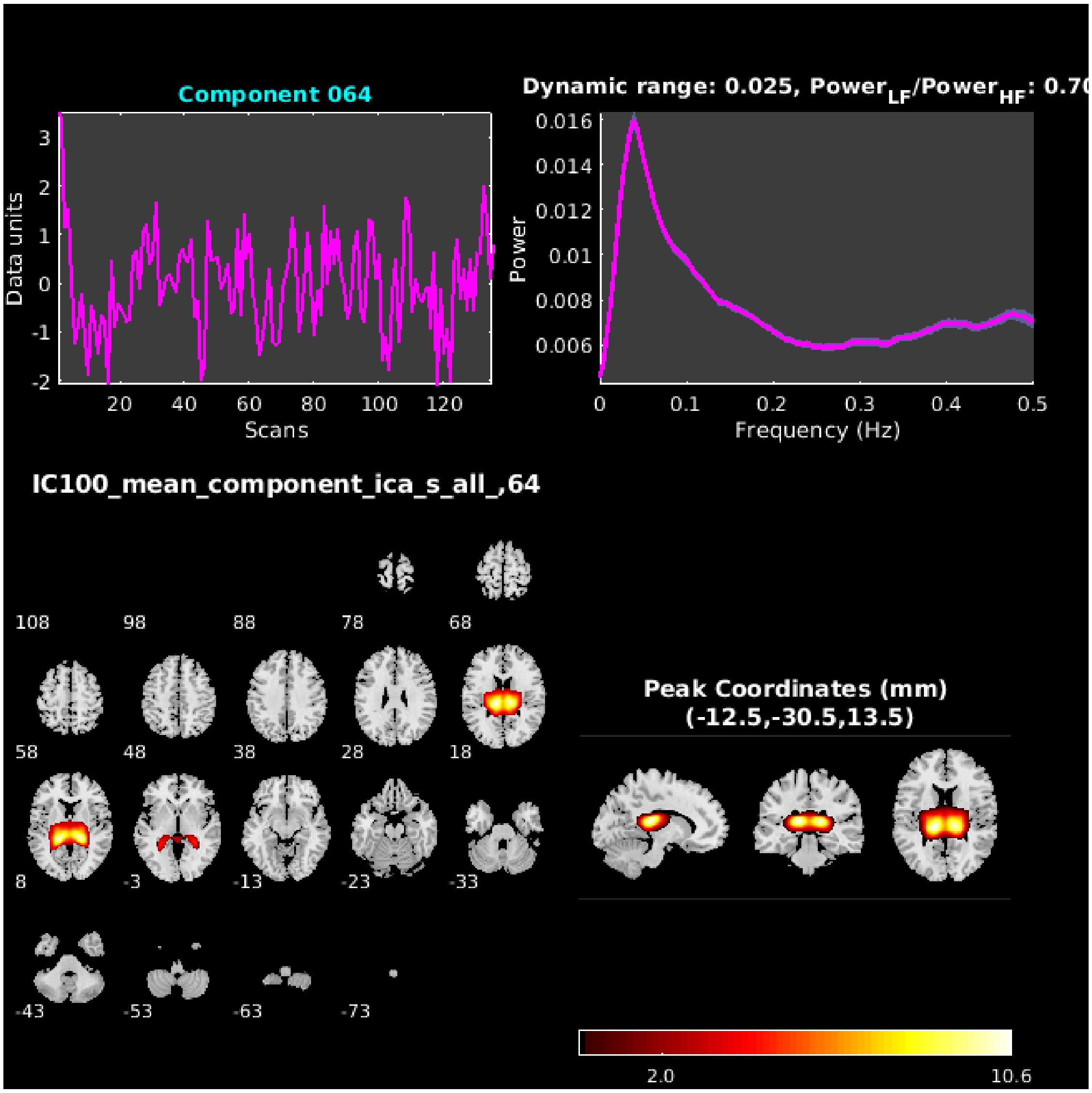
Component 064
Dynamic range: 0.025, Power$_{LF}$/Power$_{HF}$: 0.70
Data units
Scans
Power
Frequency (Hz)
IC100_mean_component_ica_s_all_,64
Peak Coordinates (mm)
(-12.5,-30.5,13.5)
2.0
10.6

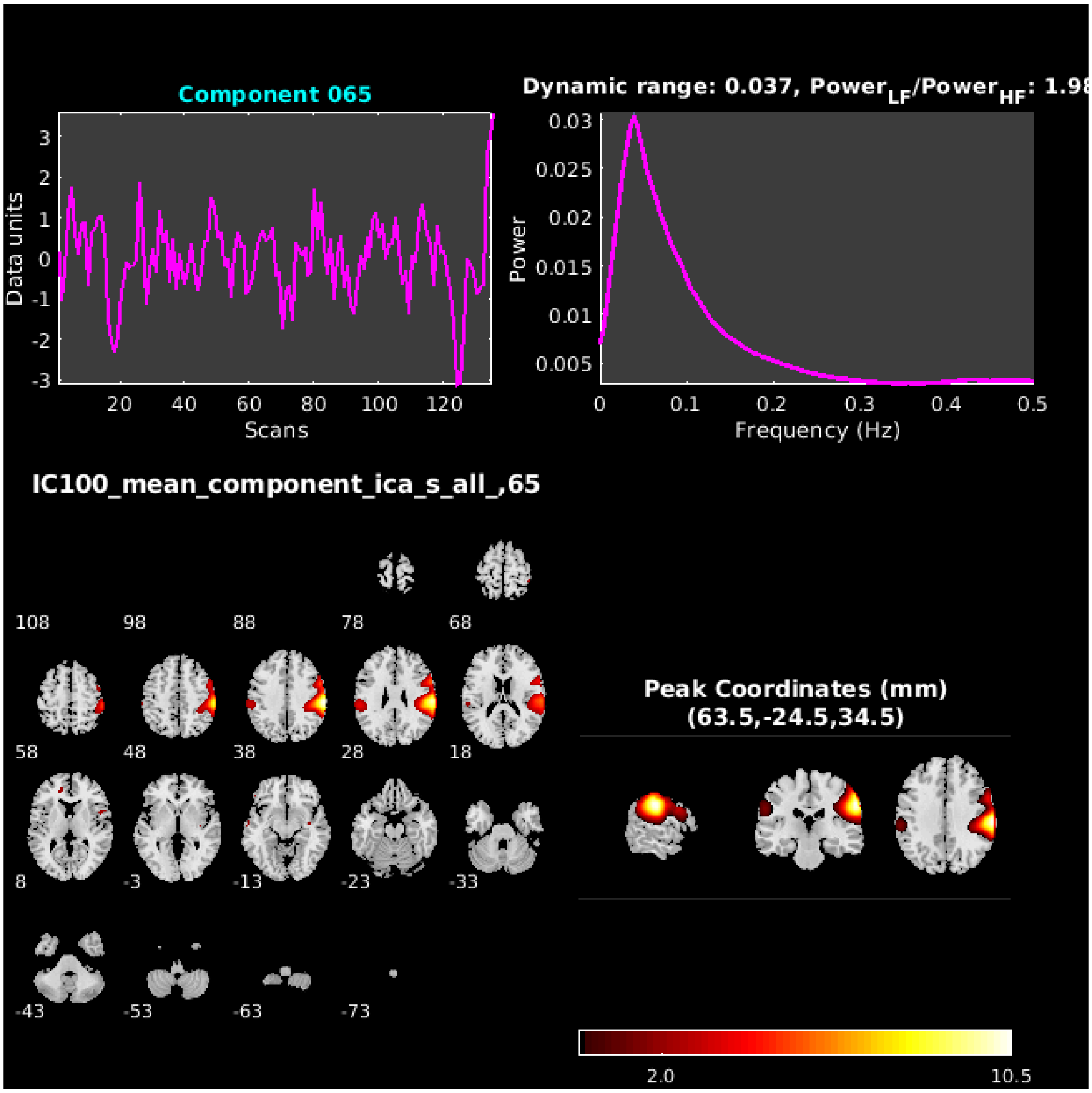
Component 065
Dynamic range: 0.037, Power_LF/Power_HF: 1.98
Data units
Scans
Power
Frequency (Hz)
IC100_mean_component_ica_s_all_,65
Peak Coordinates (mm)
(63.5,-24.5,34.5)
2.0
10.5

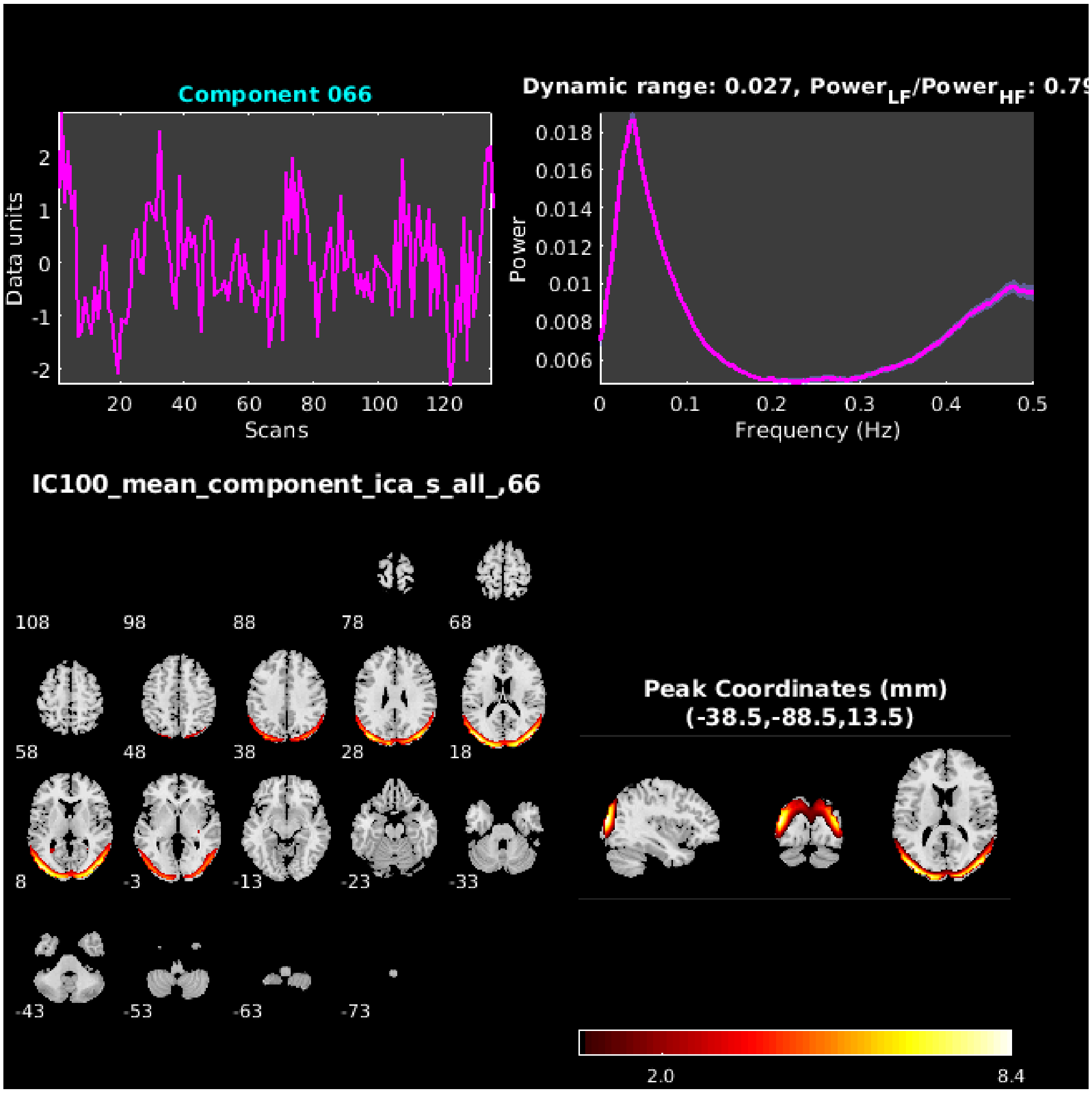

Component 066
Dynamic range: 0.027, Power$_{LF}$/Power$_{HF}$: 0.79
Data units
Scans
Power
Frequency (Hz)
IC100_mean_component_ica_s_all_,66
Peak Coordinates (mm)
(-38.5,-88.5,13.5)
2.0
8.4

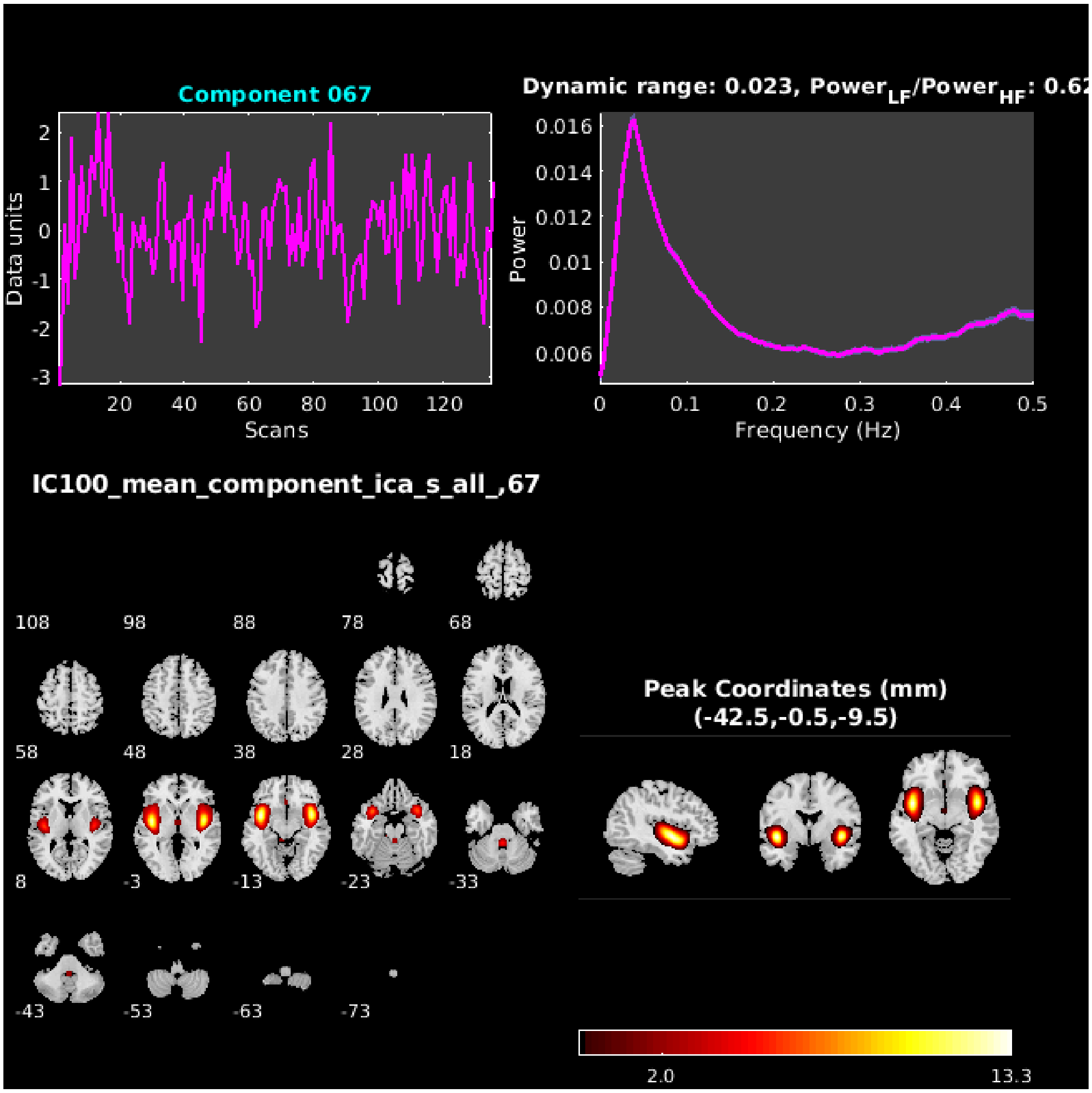
Component 067
Dynamic range: 0.023, Power_LF/Power_HF: 0.62
Data units
Scans
Power
Frequency (Hz)
IC100_mean_component_ica_s_all_,67
Peak Coordinates (mm)
(-42.5,-0.5,-9.5)
108
98
88
78
68
58
48
38
28
18
8
-3
-13
-23
-33
-43
-53
-63
-73
2.0
13.3

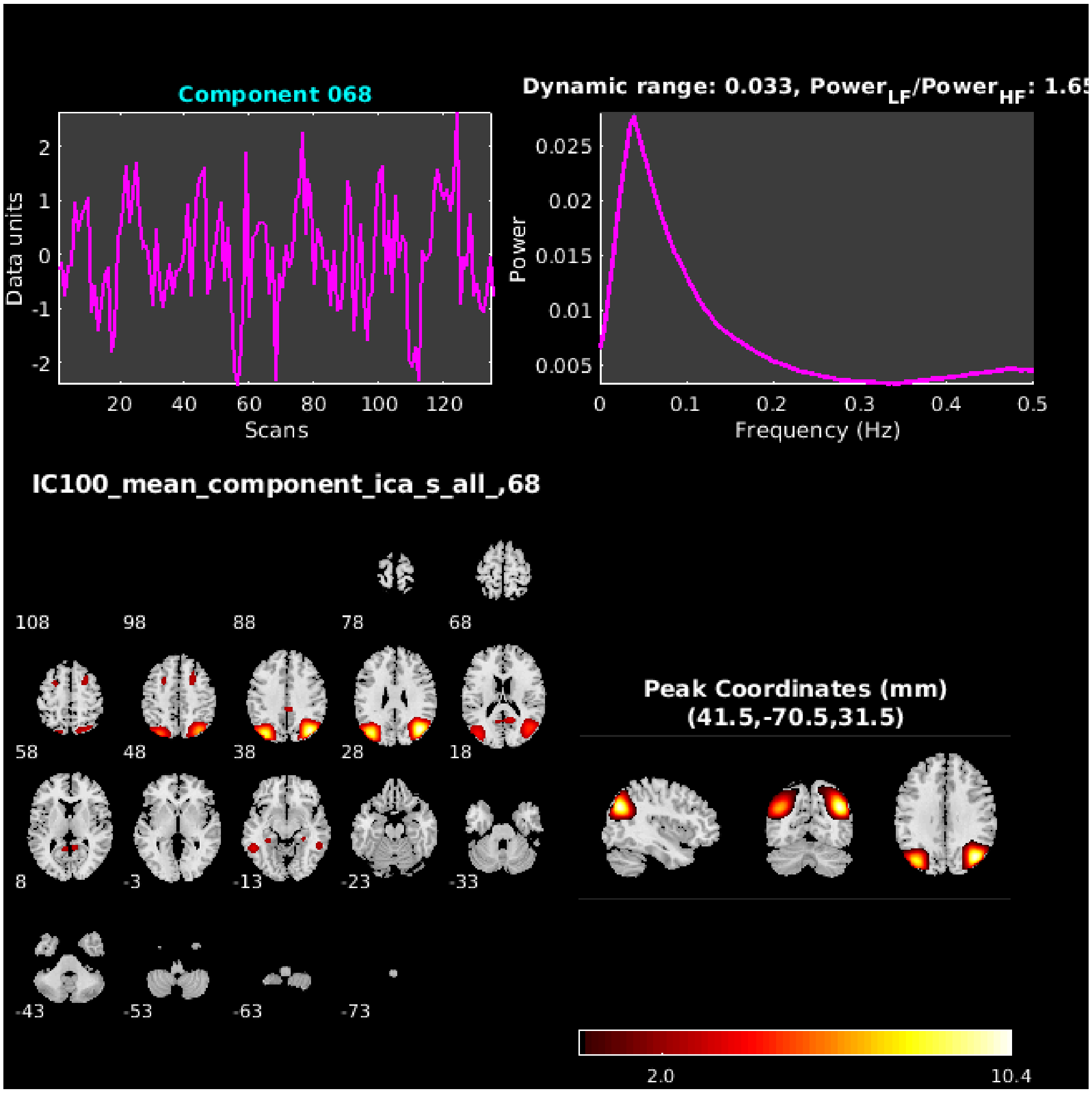
Component 068
Dynamic range: 0.033, Power_LF/Power_HF: 1.65
Data units
Scans
Power
Frequency (Hz)
IC100_mean_component_ica_s_all_,68
Peak Coordinates (mm)
(41.5,-70.5,31.5)
2.0
10.4

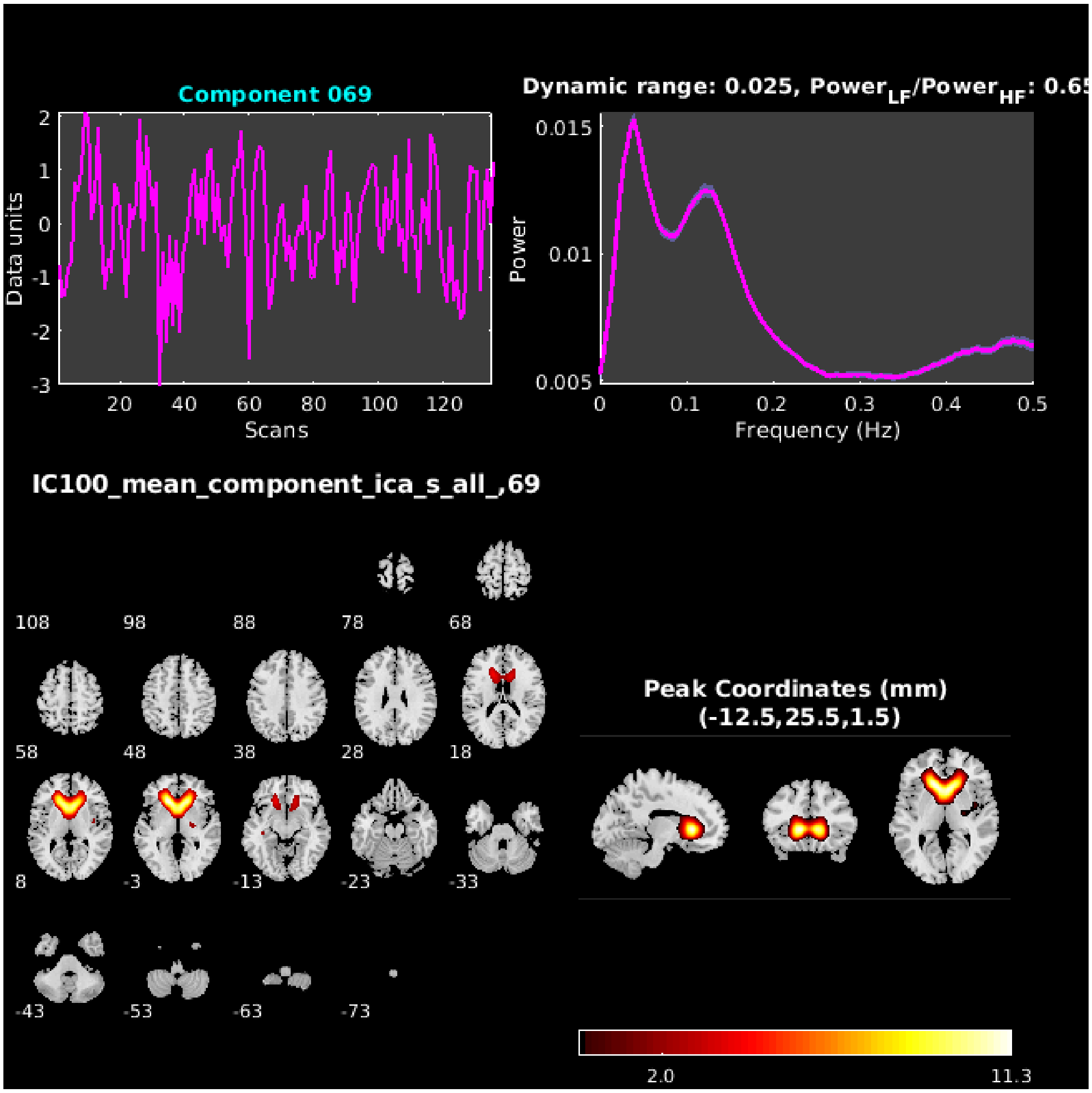
Component 069
Dynamic range: 0.025, Power$_{LF}$/Power$_{HF}$: 0.65
Data units
Scans
Power
Frequency (Hz)
IC100_mean_component_ica_s_all_,69
Peak Coordinates (mm)
(-12.5,25.5,1.5)
2.0
11.3

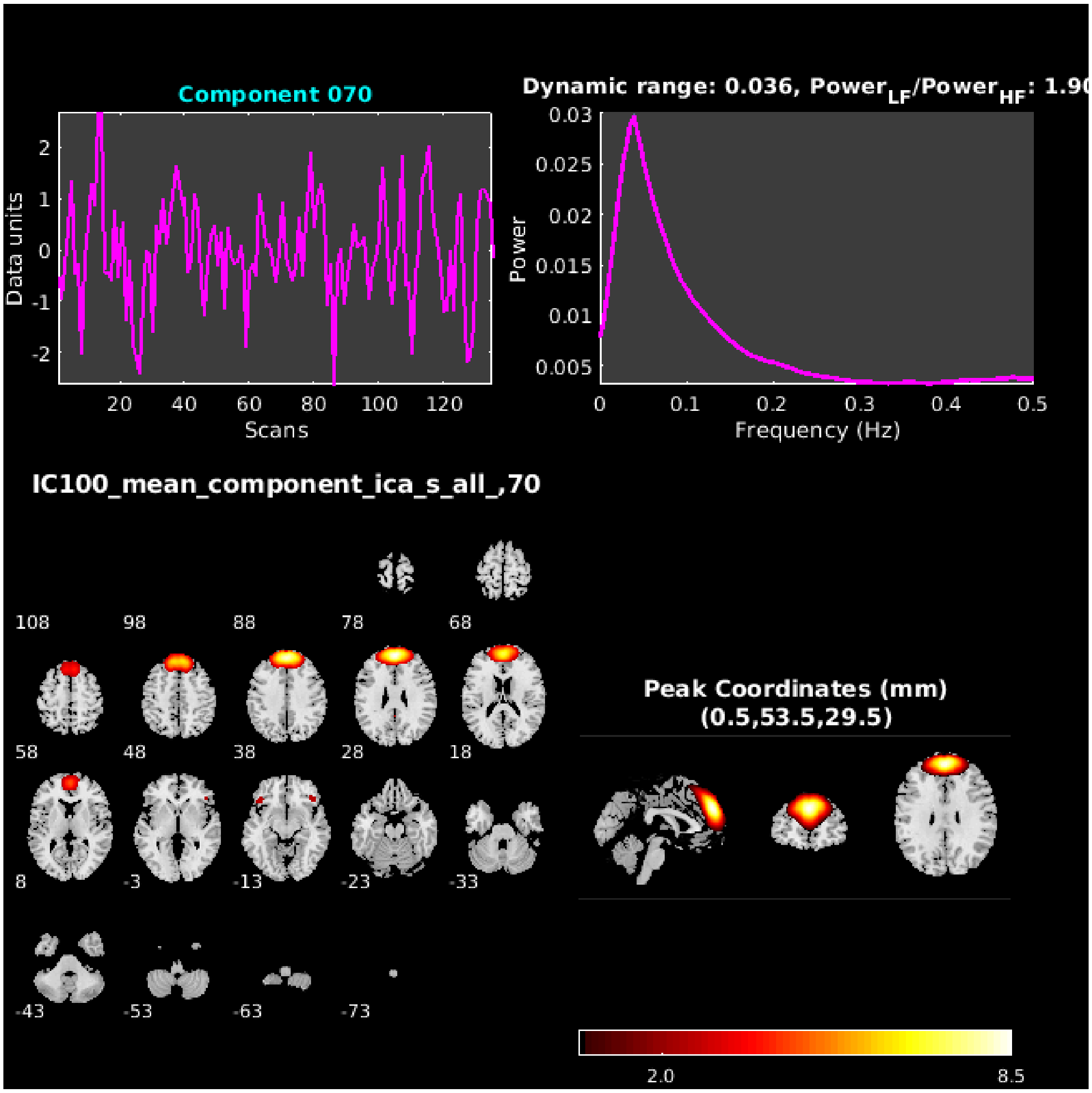

Component 070
Dynamic range: 0.036, Power$_{LF}$/Power$_{HF}$: 1.9
Data units
Scans
Power
Frequency (Hz)
IC100_mean_component_ica_s_all_,70
Peak Coordinates (mm)
(0.5,53.5,29.5)
2.0
8.5

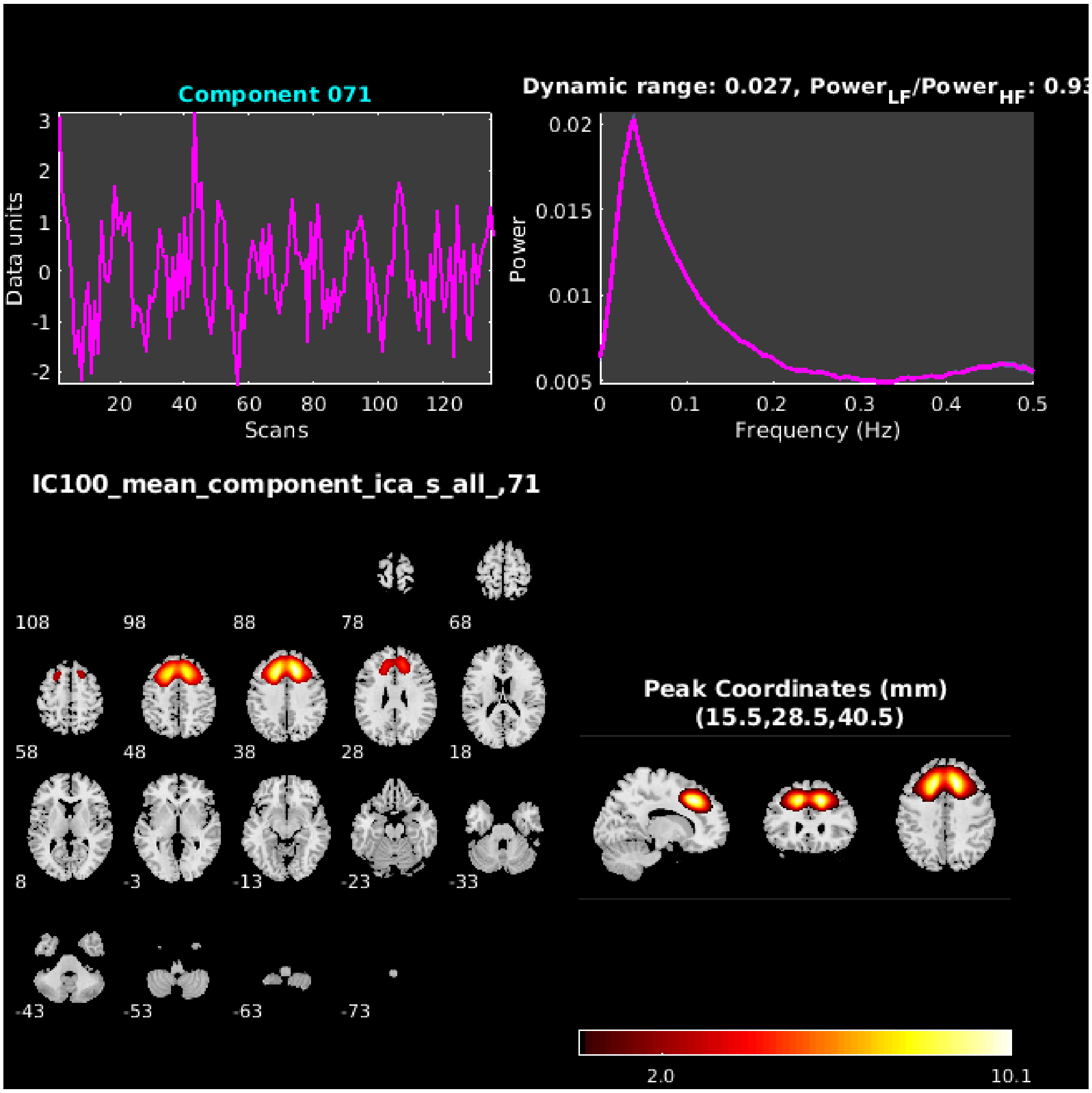
Component 071
Dynamic range: 0.027, Power$_{LF}$/Power$_{HF}$: 0.93
Data units
Scans
Power
Frequency (Hz)
IC100_mean_component_ica_s_all_,71
Peak Coordinates (mm)
(15.5,28.5,40.5)
2.0
10.1

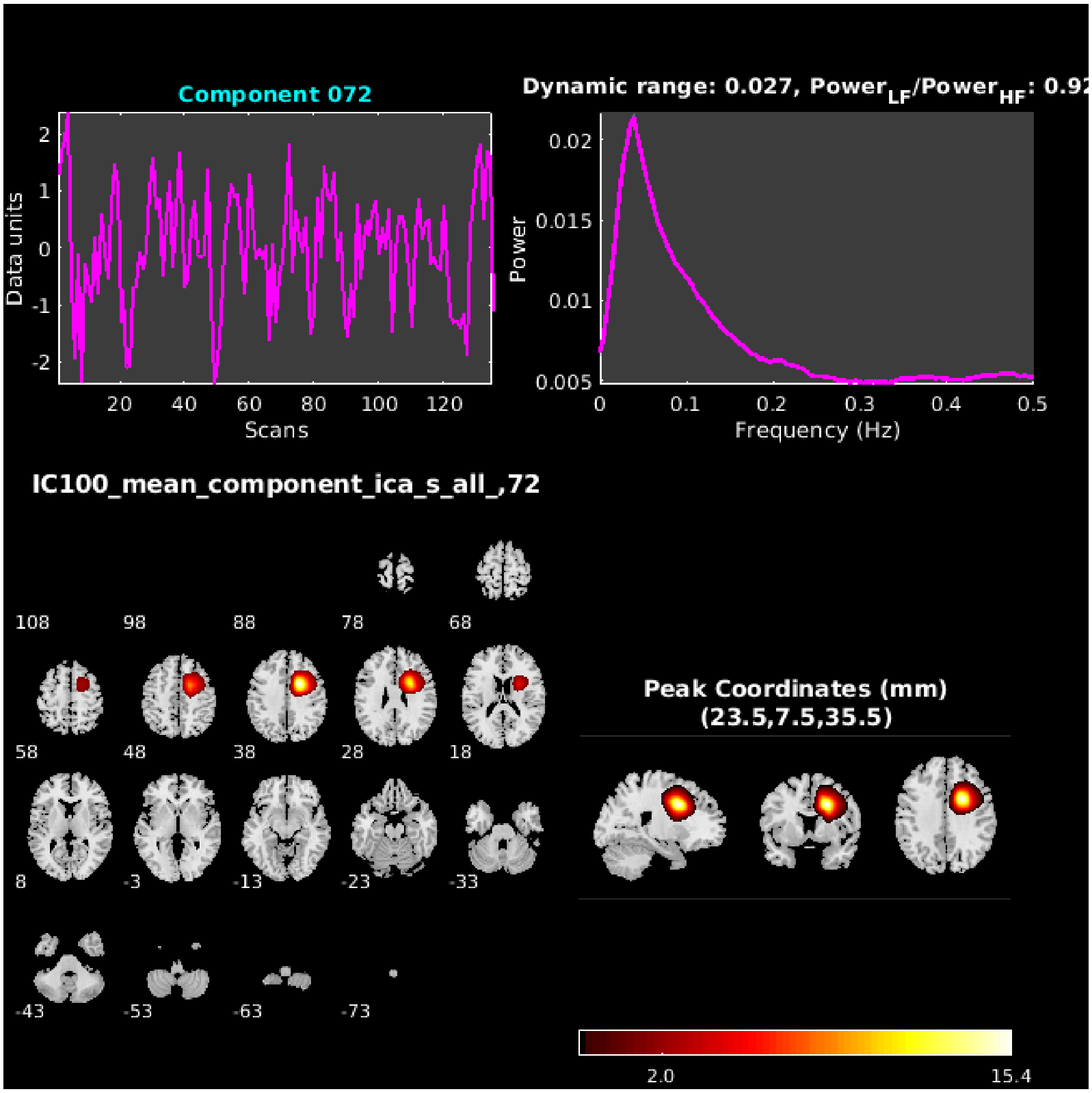
Component 072
Dynamic range: 0.027, Power$_{LF}$/Power$_{HF}$: 0.92
Data units
Scans
Power
Frequency (Hz)
IC100_mean_component_ica_s_all_,72
Peak Coordinates (mm)
(23.5,7.5,35.5)
2.0
15.4

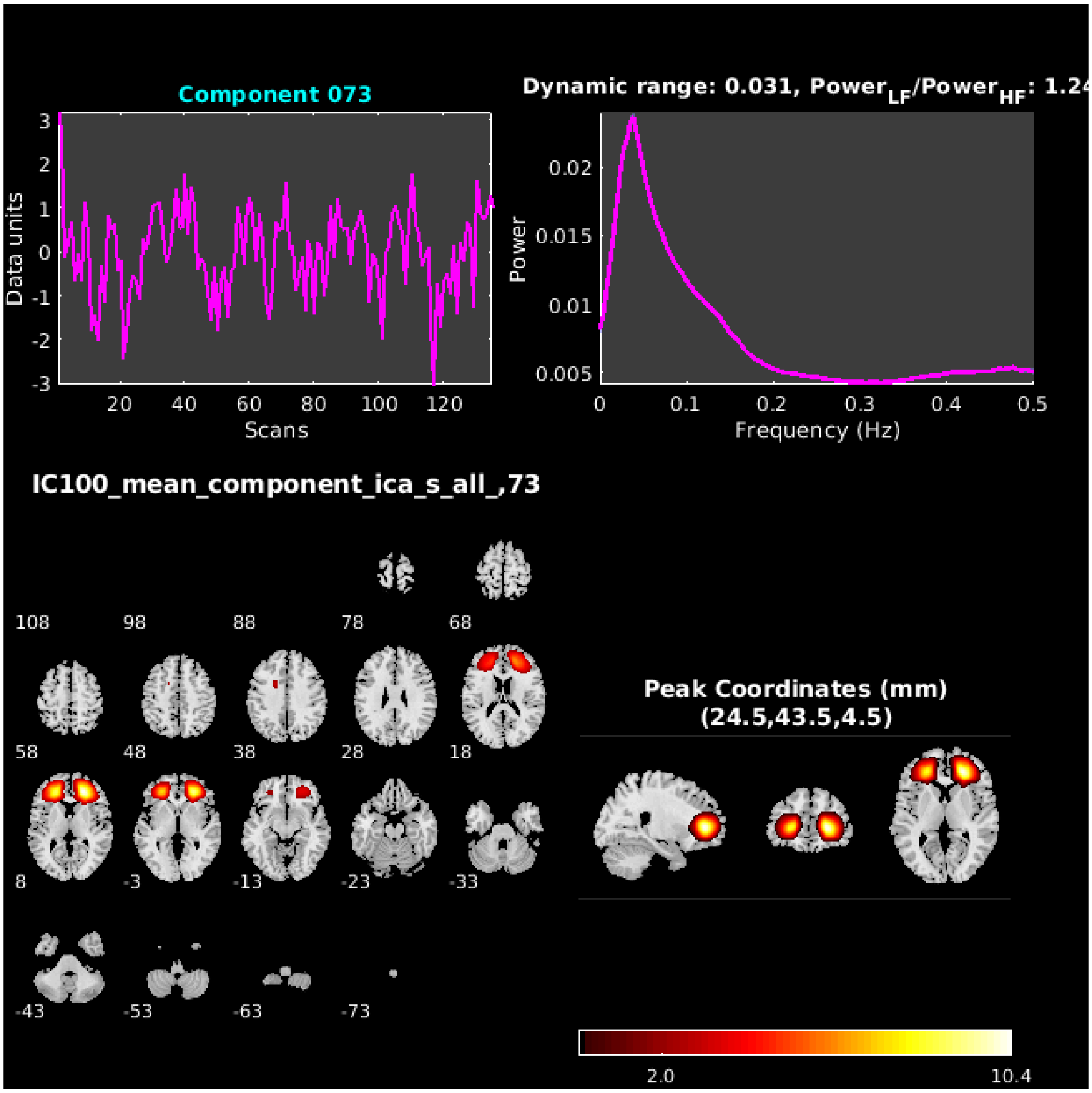
Component 073
Dynamic range: 0.031, Power$_{LF}$/Power$_{HF}$: 1.24
Data units
Scans
Power
Frequency (Hz)
IC100_mean_component_ica_s_all_,73
Peak Coordinates (mm)
(24.5,43.5,4.5)
2.0
10.4

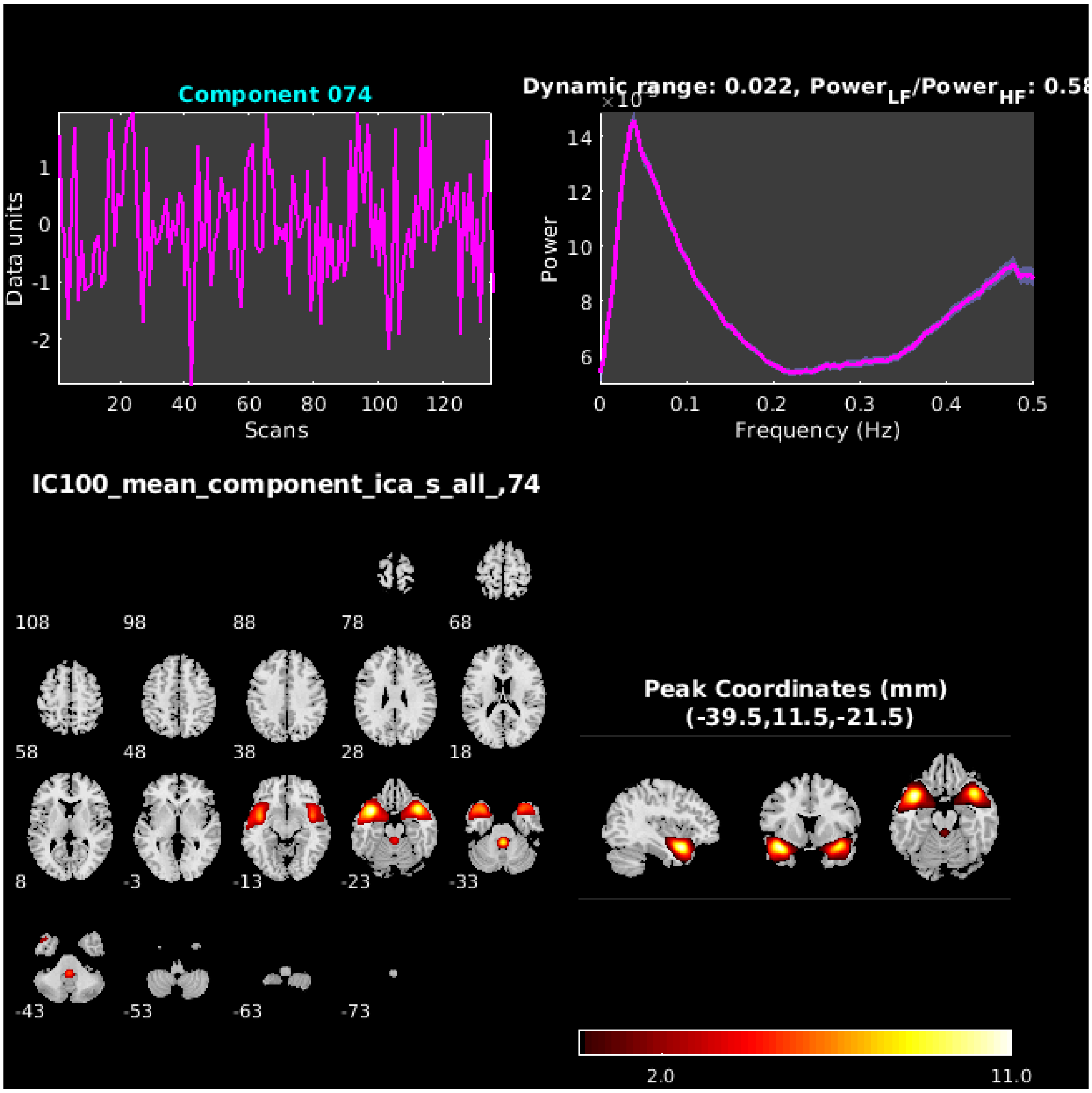
Component 074
Data units
Scans
Dynamic range: 0.022, Power_LF/Power_HF: 0.58
Power
Frequency (Hz)
IC100_mean_component_ica_s_all_,74
Peak Coordinates (mm)
(-39.5,11.5,-21.5)
2.0
11.0

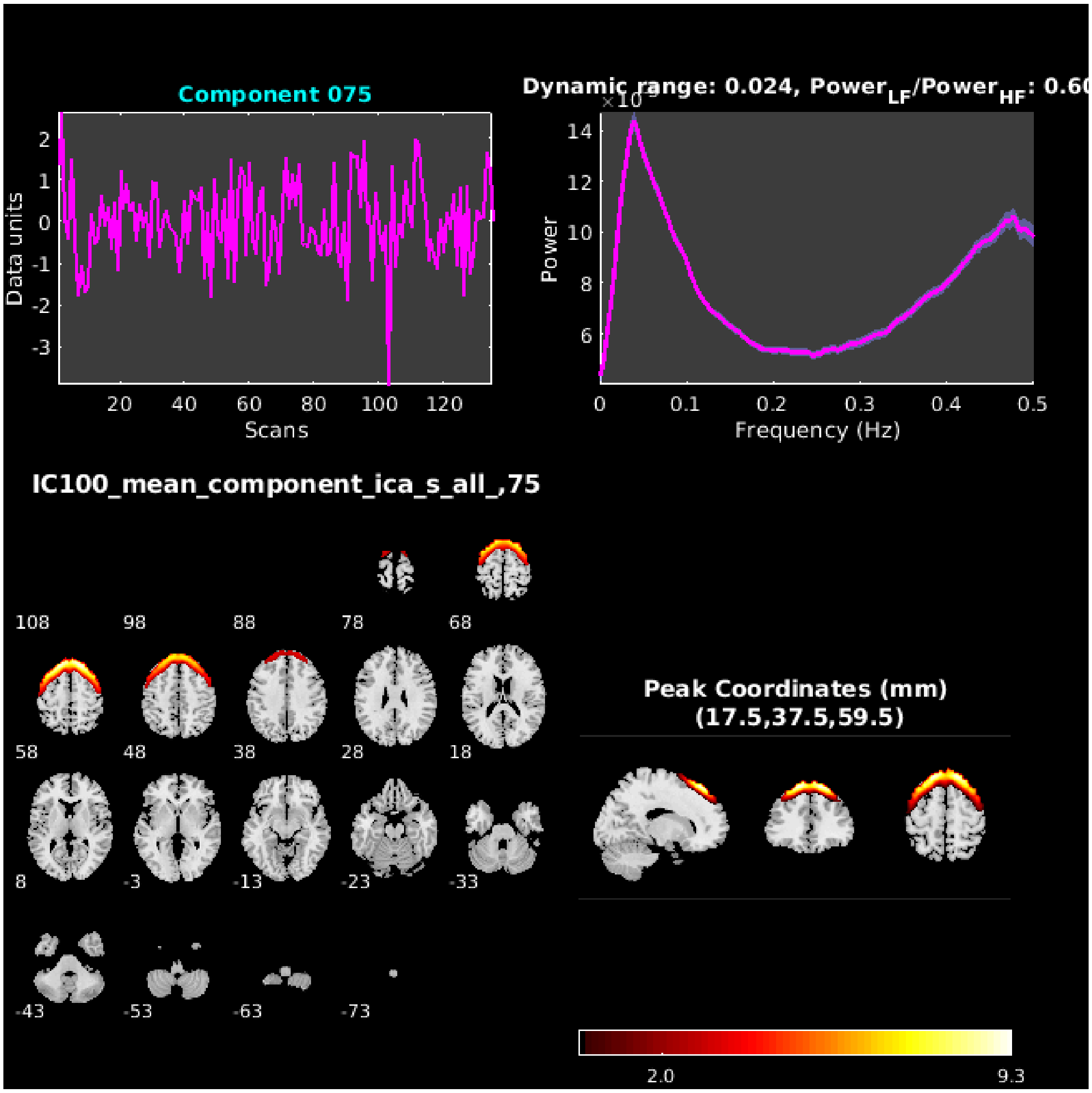
Component 075
Dynamic range: 0.024, Power$_{LF}$/Power$_{HF}$: 0.60
Data units
Scans
Power
Frequency (Hz)
IC100_mean_component_ica_s_all_,75
Peak Coordinates (mm)
(17.5,37.5,59.5)
2.0
9.3

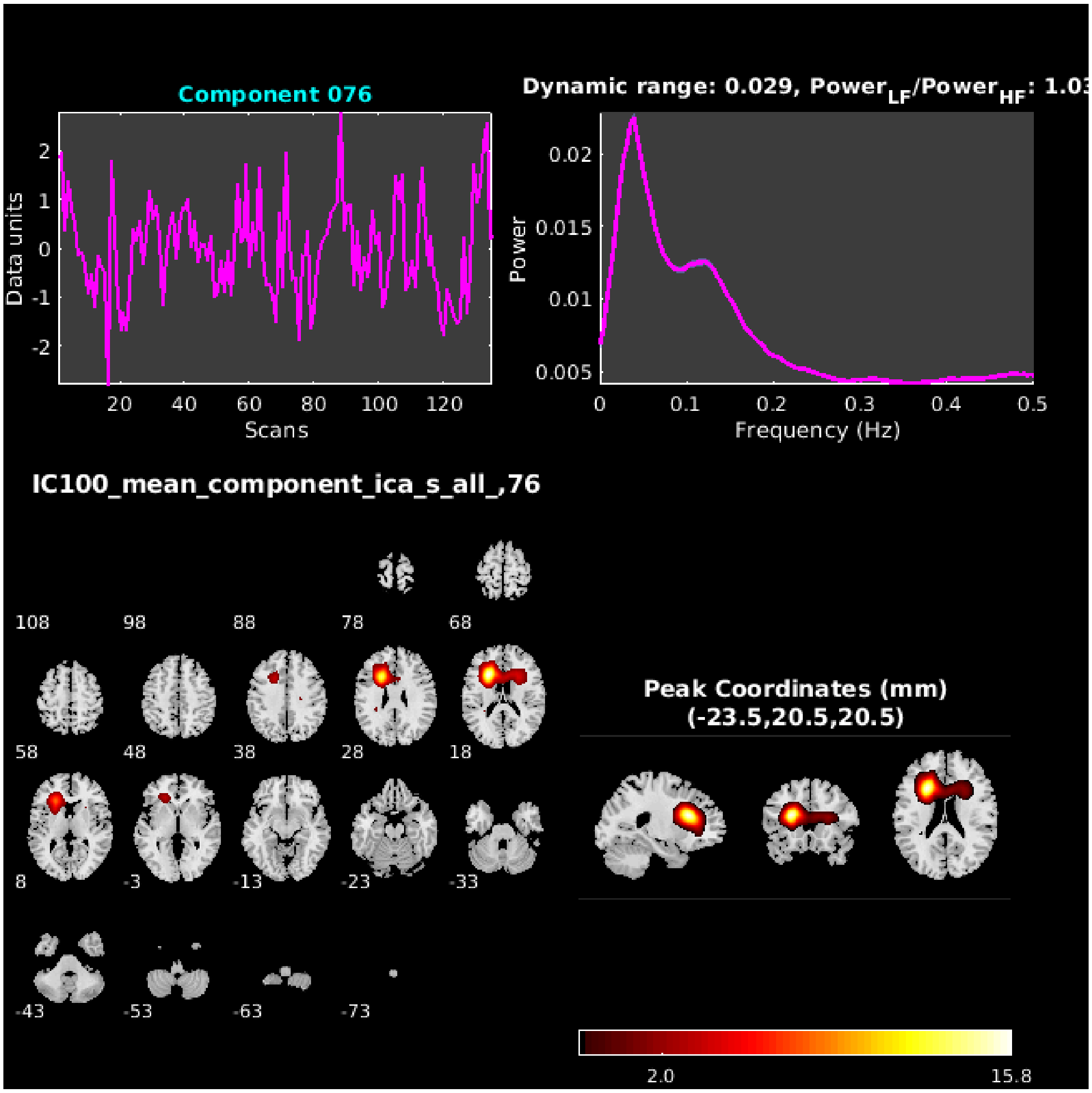
Component 076
Dynamic range: 0.029, Power$_{LF}$/Power$_{HF}$: 1.03
Data units
Scans
Power
Frequency (Hz)
IC100_mean_component_ica_s_all_,76
Peak Coordinates (mm)
(-23.5,20.5,20.5)

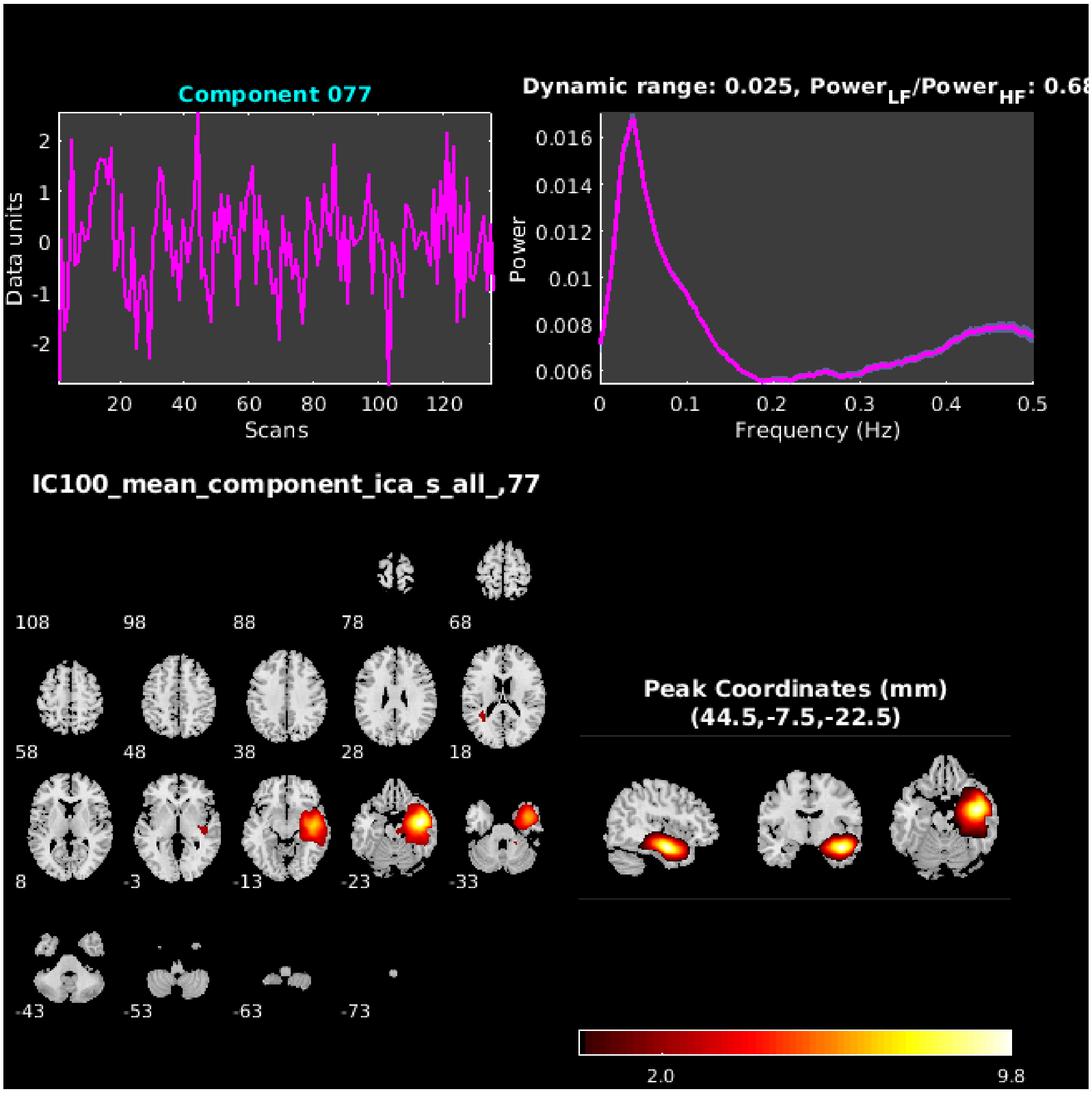

Component 077
Dynamic range: 0.025, Power_LF/Power_HF: 0.68
Data units
Scans
Power
Frequency (Hz)
IC100_mean_component_ica_s_all_,77
Peak Coordinates (mm)
(44.5,-7.5,-22.5)
2.0
9.8

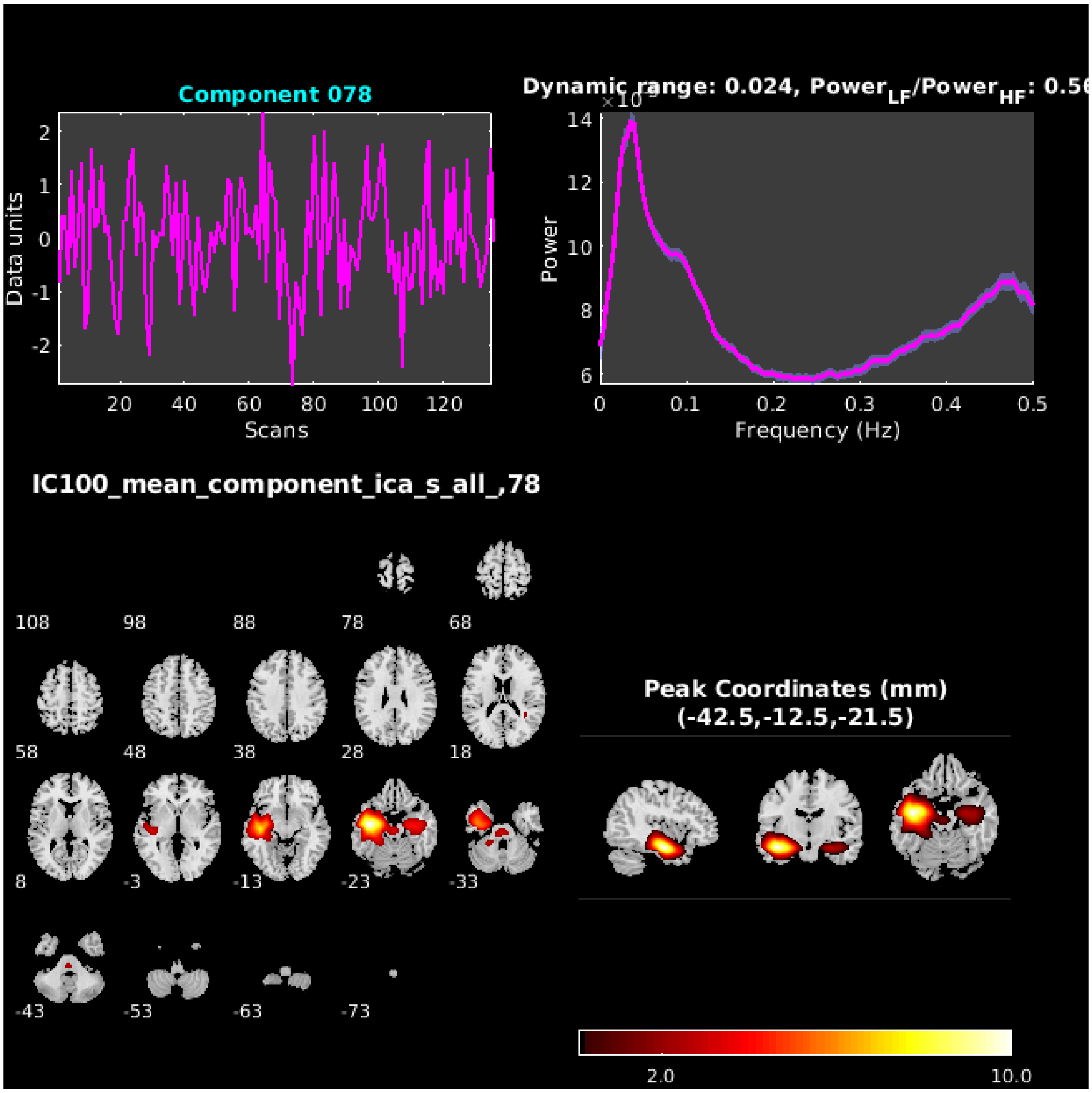

Component 078
Dynamic range: 0.024, Power_LF/Power_HF: 0.56
Data units
Scans
Power
Frequency (Hz)
IC100_mean_component_ica_s_all_,78
Peak Coordinates (mm)
(-42.5,-12.5,-21.5)
2.0
10.0

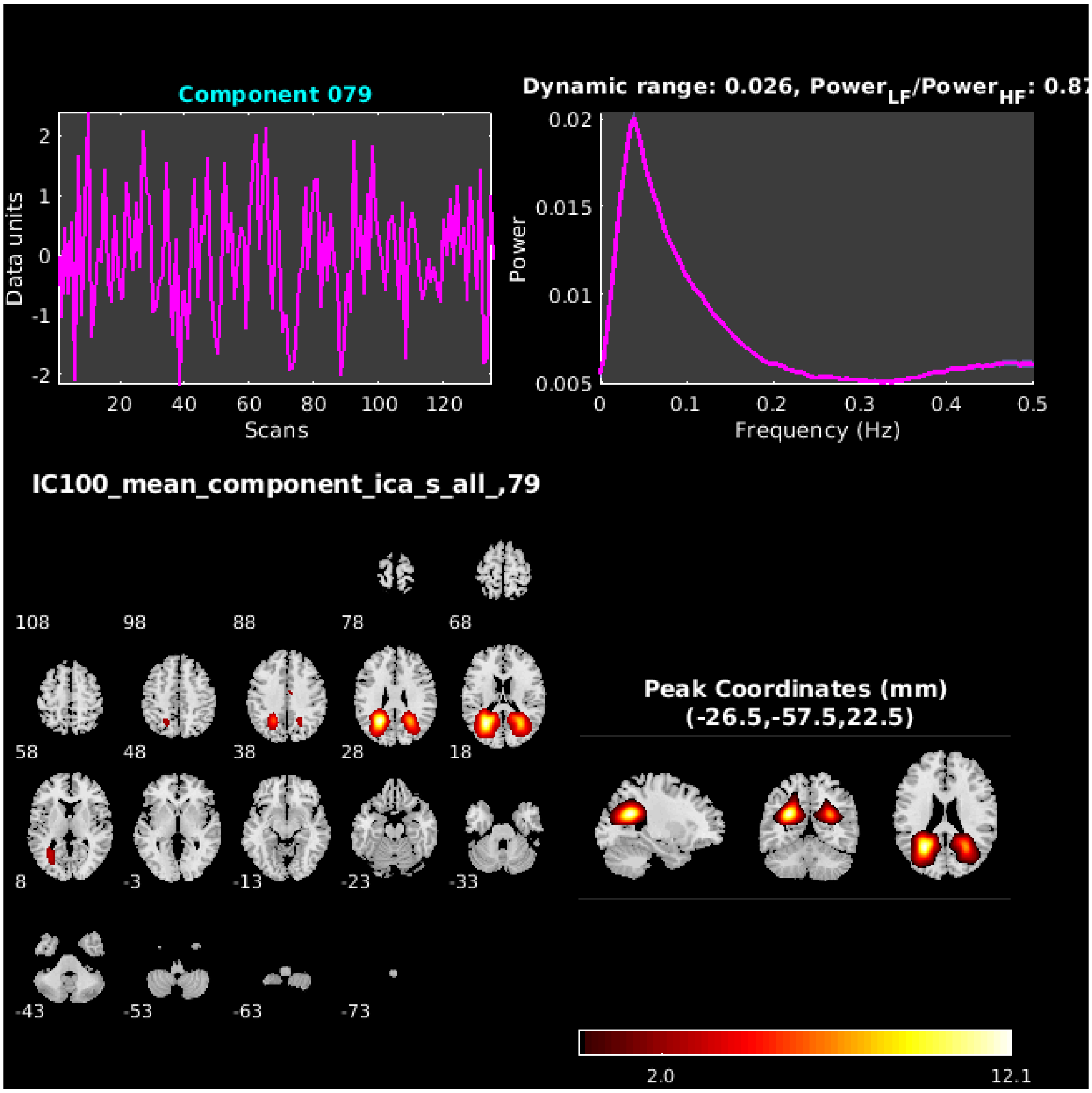
Component 079
Dynamic range: 0.026, Power$_{LF}$/Power$_{HF}$: 0.87
Data units
Scans
Power
Frequency (Hz)
IC100_mean_component_ica_s_all_,79
Peak Coordinates (mm)
(-26.5,-57.5,22.5)
2.0
12.1

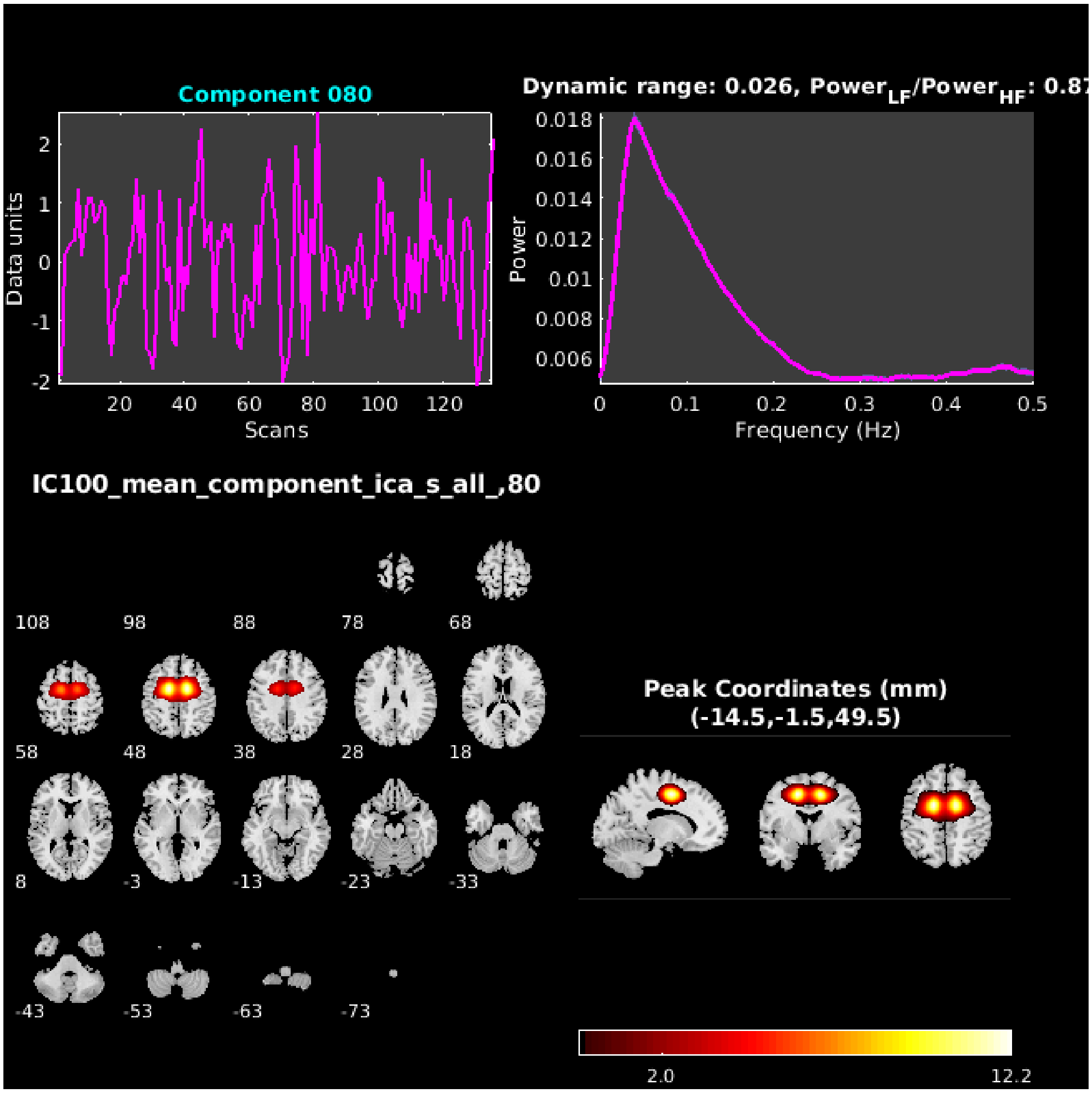
Component 080
Dynamic range: 0.026, Power_LF/Power_HF: 0.87
Data units
Scans
Power
Frequency (Hz)
IC100_mean_component_ica_s_all_,80
Peak Coordinates (mm)
(-14.5,-1.5,49.5)
2.0
12.2

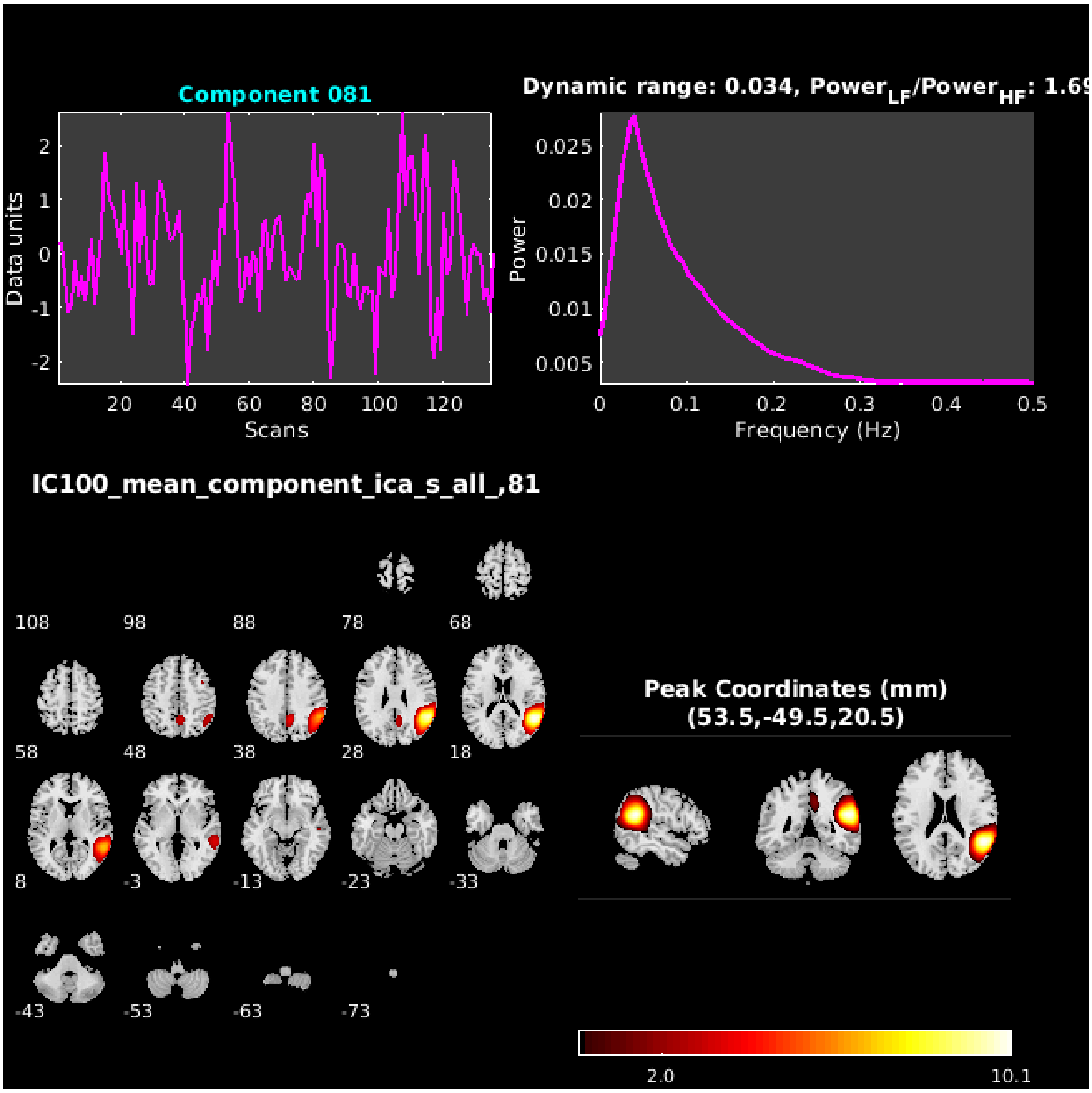
Component 081
Dynamic range: 0.034, Power$_{LF}$/Power$_{HF}$: 1.69
Data units
Scans
Power
Frequency (Hz)
IC100_mean_component_ica_s_all_,81
Peak Coordinates (mm)
(53.5,-49.5,20.5)
2.0
10.1

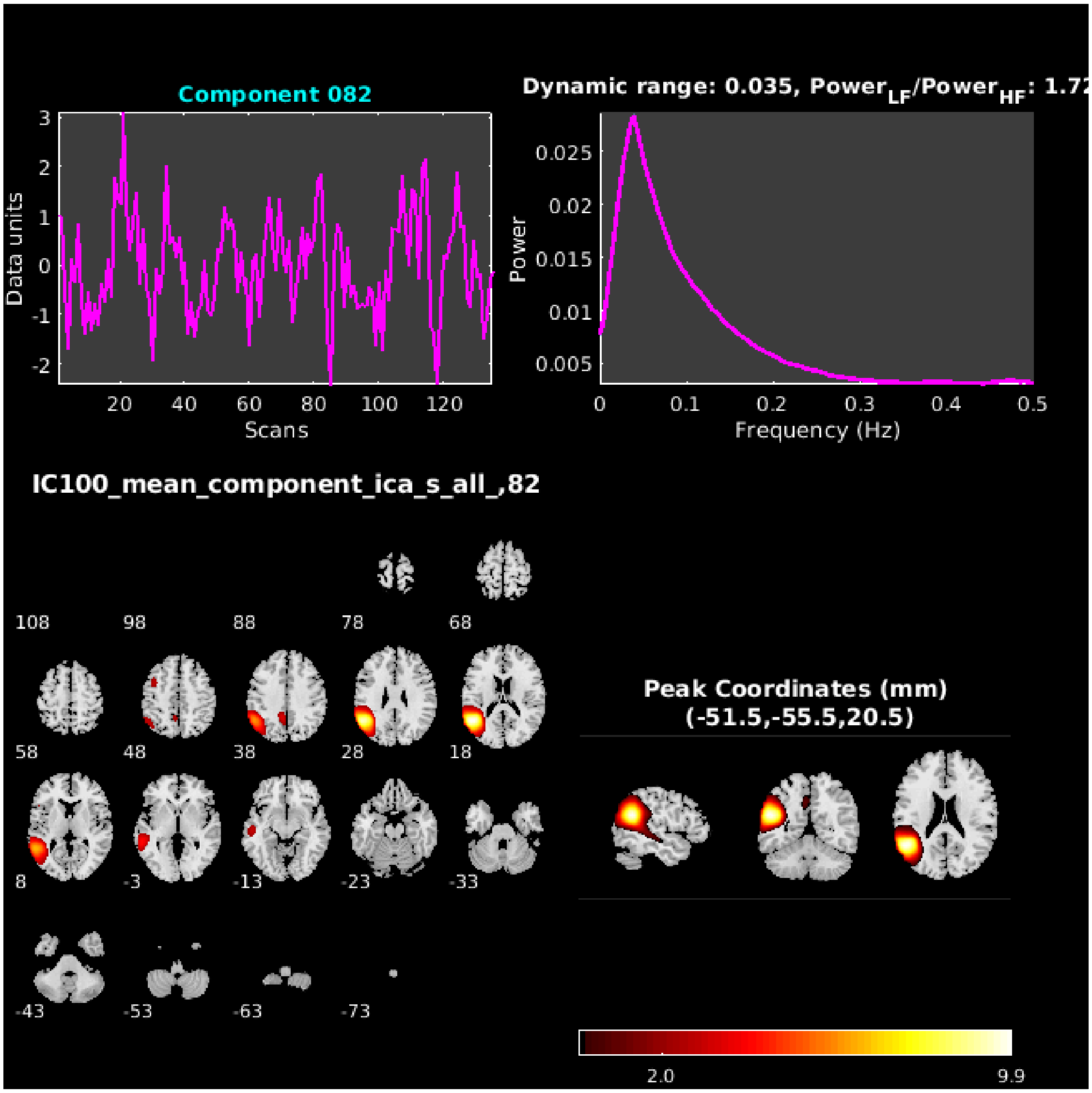

Component 082
Dynamic range: 0.035, Power$_{LF}$/Power$_{HF}$: 1.72
Data units
Scans
Power
Frequency (Hz)
IC100_mean_component_ica_s_all_,82
Peak Coordinates (mm)
(-51.5,-55.5,20.5)
2.0
9.9

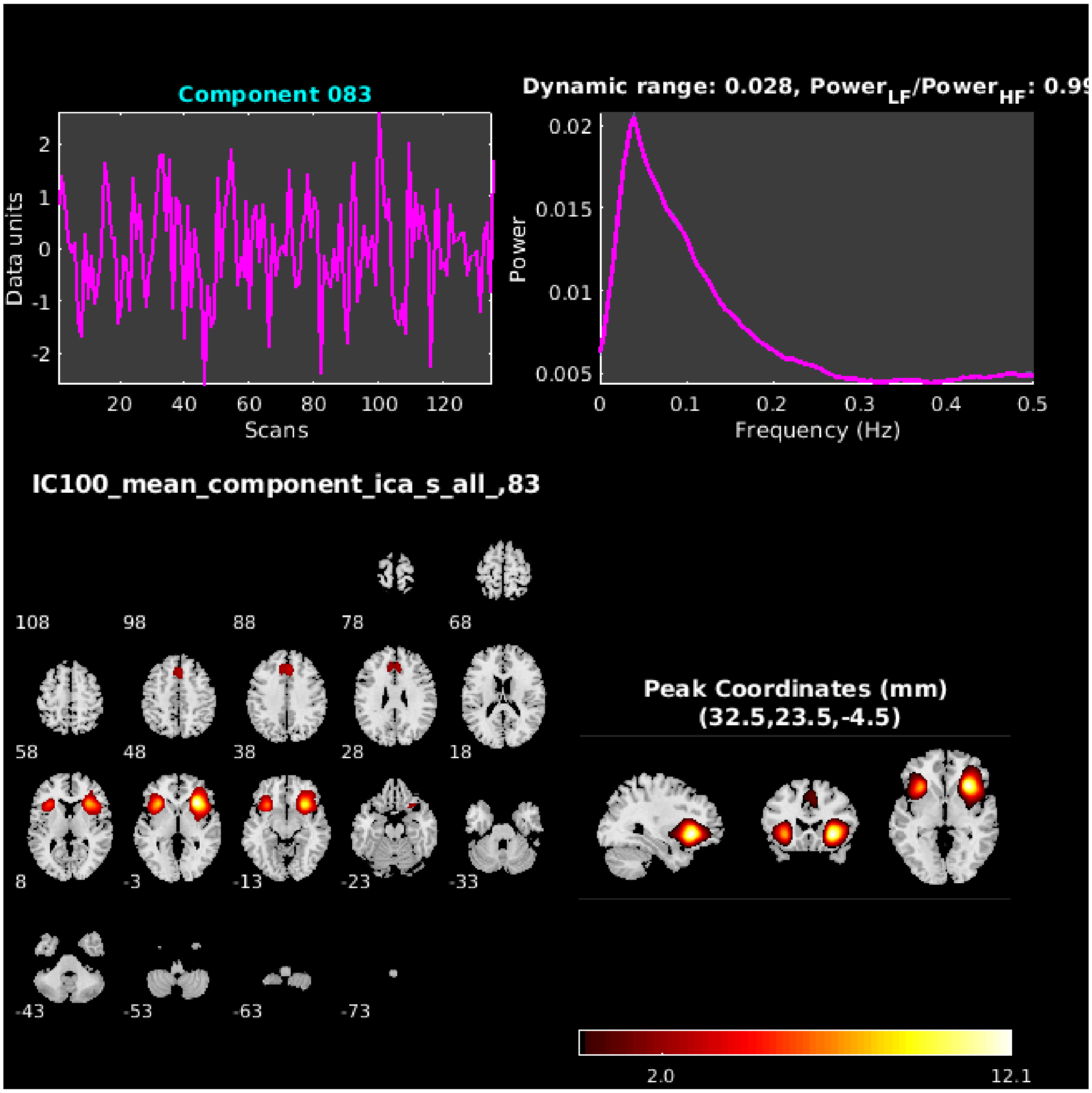
Component 083
Dynamic range: 0.028, Power$_{LF}$/Power$_{HF}$: 0.99
Data units
Scans
Power
Frequency (Hz)
IC100_mean_component_ica_s_all_,83
Peak Coordinates (mm)
(32.5,23.5,-4.5)
2.0
12.1

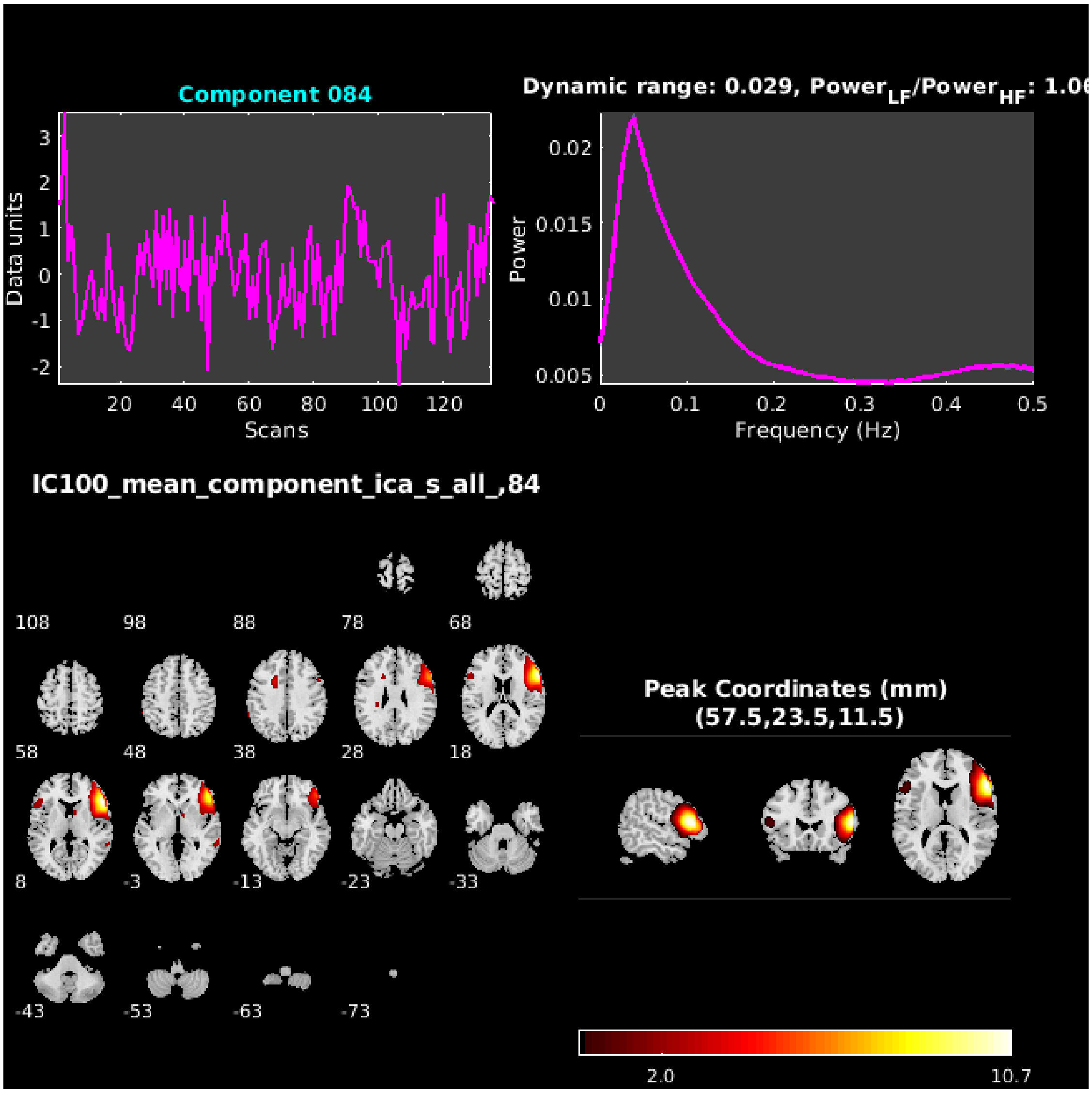
Component 084
Dynamic range: 0.029, Power$_{LF}$/Power$_{HF}$: 1.06
Data units
Scans
Power
Frequency (Hz)
IC100_mean_component_ica_s_all_,84
Peak Coordinates (mm)
(57.5,23.5,11.5)
2.0
10.7

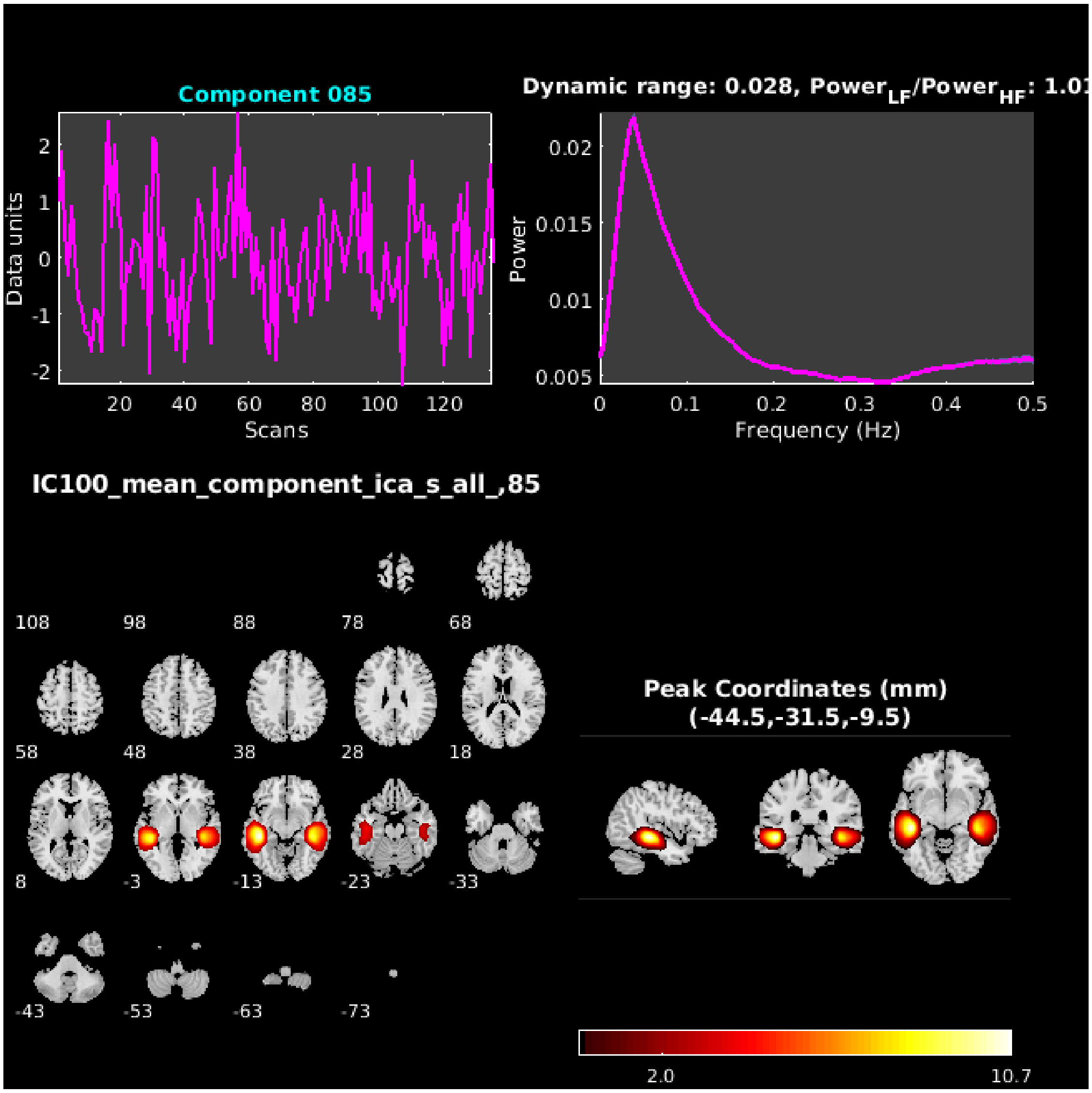
Component 085
Dynamic range: 0.028, Power$_{LF}$/Power$_{HF}$: 1.01
Data units
Scans
Power
Frequency (Hz)
IC100_mean_component_ica_s_all_,85
Peak Coordinates (mm)
(-44.5,-31.5,-9.5)
2.0
10.7

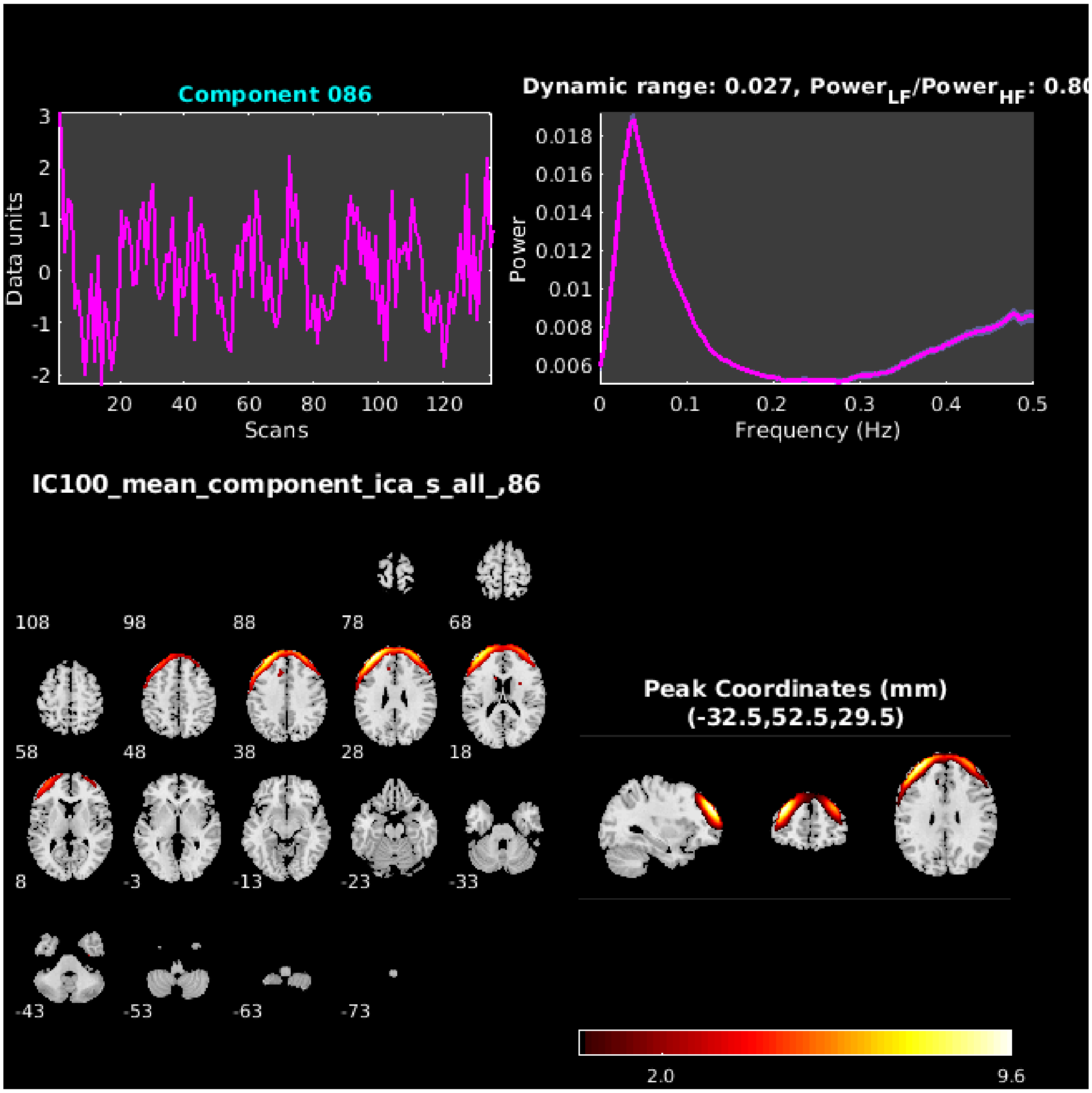
Component 086
Dynamic range: 0.027, Power$_{LF}$/Power$_{HF}$: 0.8[illegible]
Data units
Scans
Power
Frequency (Hz)
IC100_mean_component_ica_s_all_,86
108
98
88
78
68
58
48
38
28
18
8
-3
-13
-23
-33
-43
-53
-63
-73
Peak Coordinates (mm)
(-32.5,52.5,29.5)
2.0
9.6

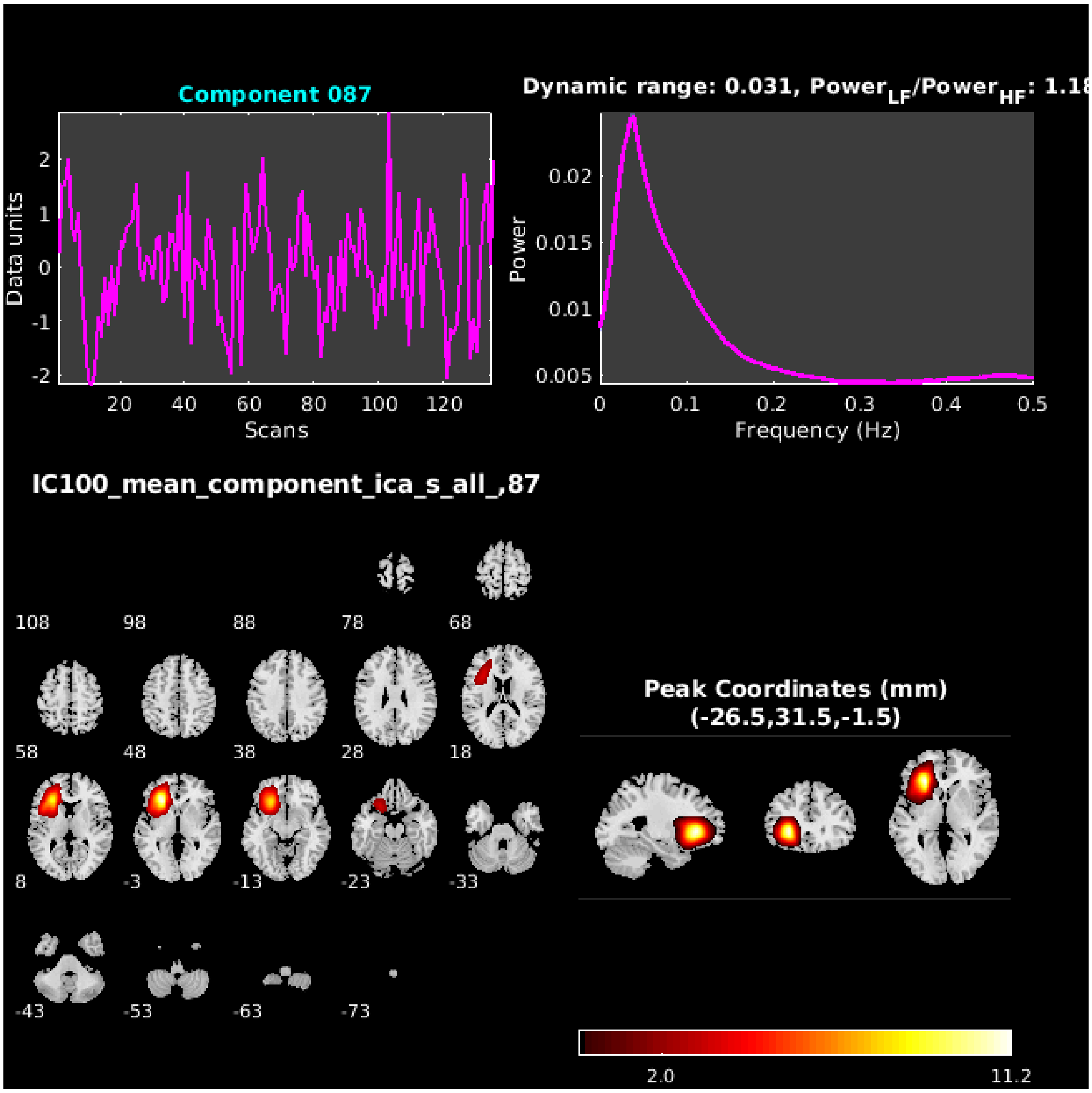
Component 087
Dynamic range: 0.031, Power$_{LF}$/Power$_{HF}$: 1.18
Data units
Scans
Power
Frequency (Hz)
IC100_mean_component_ica_s_all_,87
Peak Coordinates (mm)
(-26.5,31.5,-1.5)
2.0
11.2

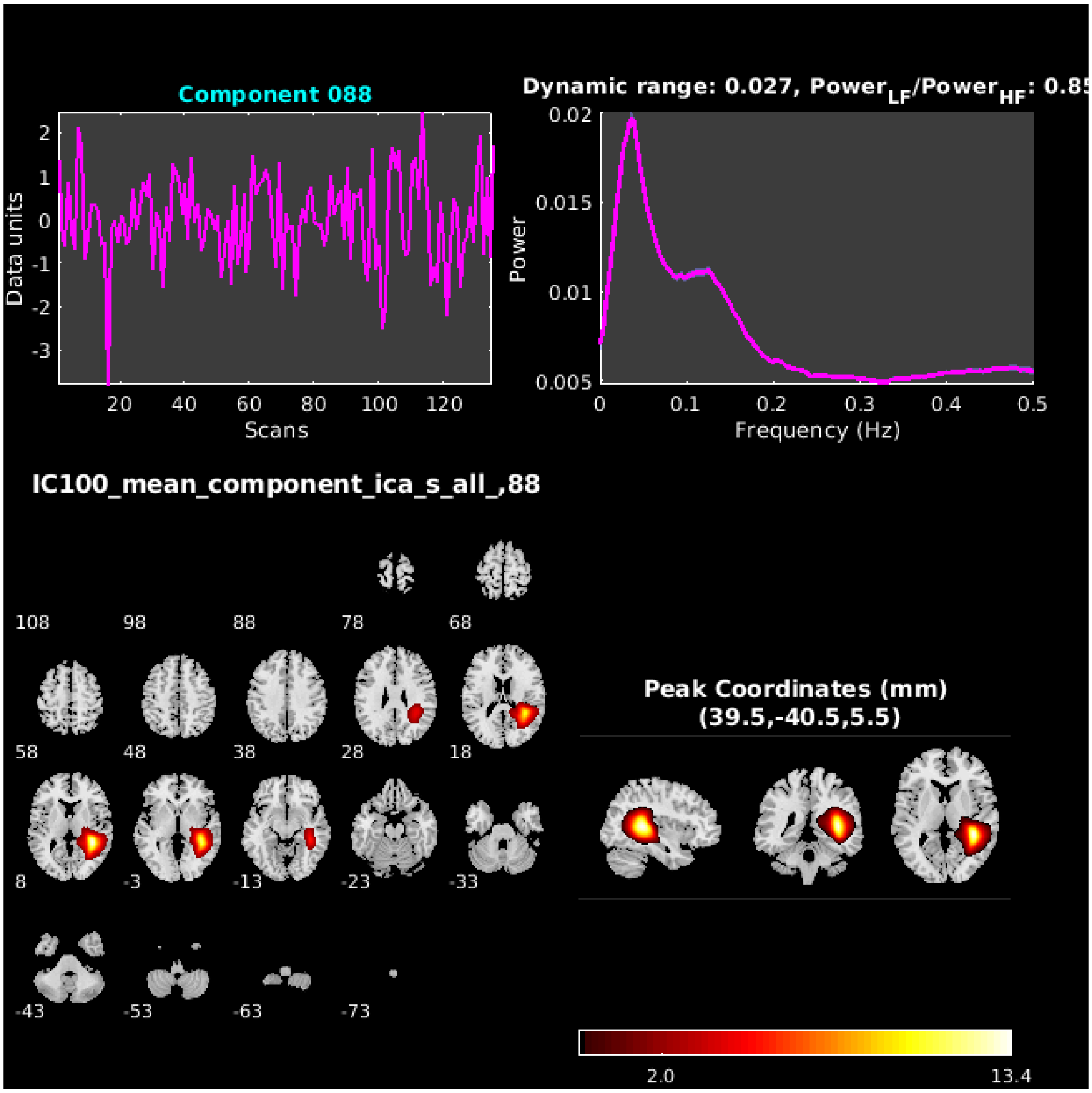
Component 088
Dynamic range: 0.027, Power$_{LF}$/Power$_{HF}$: 0.85
Data units
Scans
Power
Frequency (Hz)
IC100_mean_component_ica_s_all_,88
Peak Coordinates (mm)
(39.5,-40.5,5.5)
2.0
13.4

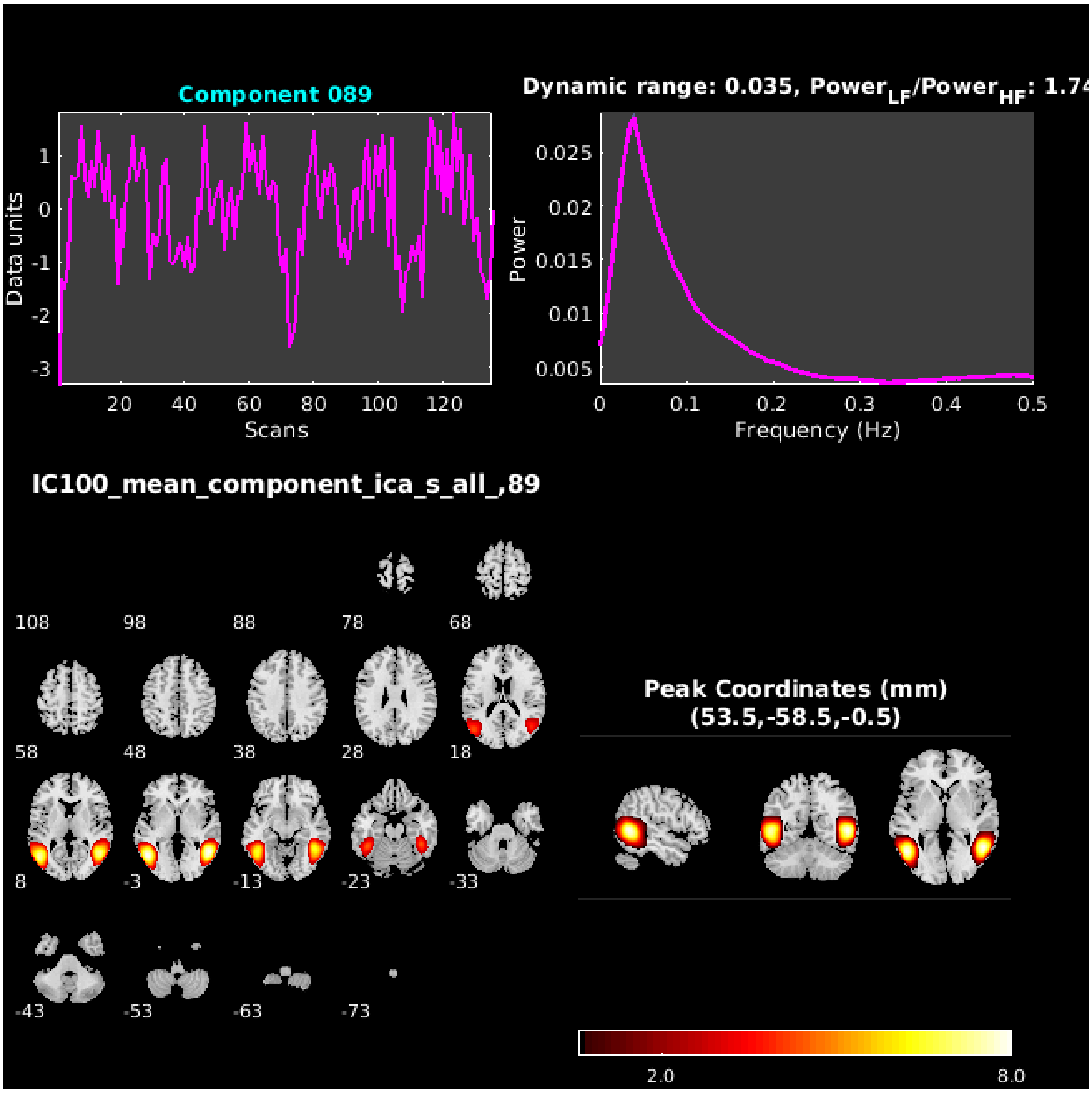
Component 089
Dynamic range: 0.035, Power$_{LF}$/Power$_{HF}$: 1.74
Data units
Scans
Power
Frequency (Hz)
IC100_mean_component_ica_s_all_,89
Peak Coordinates (mm)
(53.5,-58.5,-0.5)
2.0
8.0

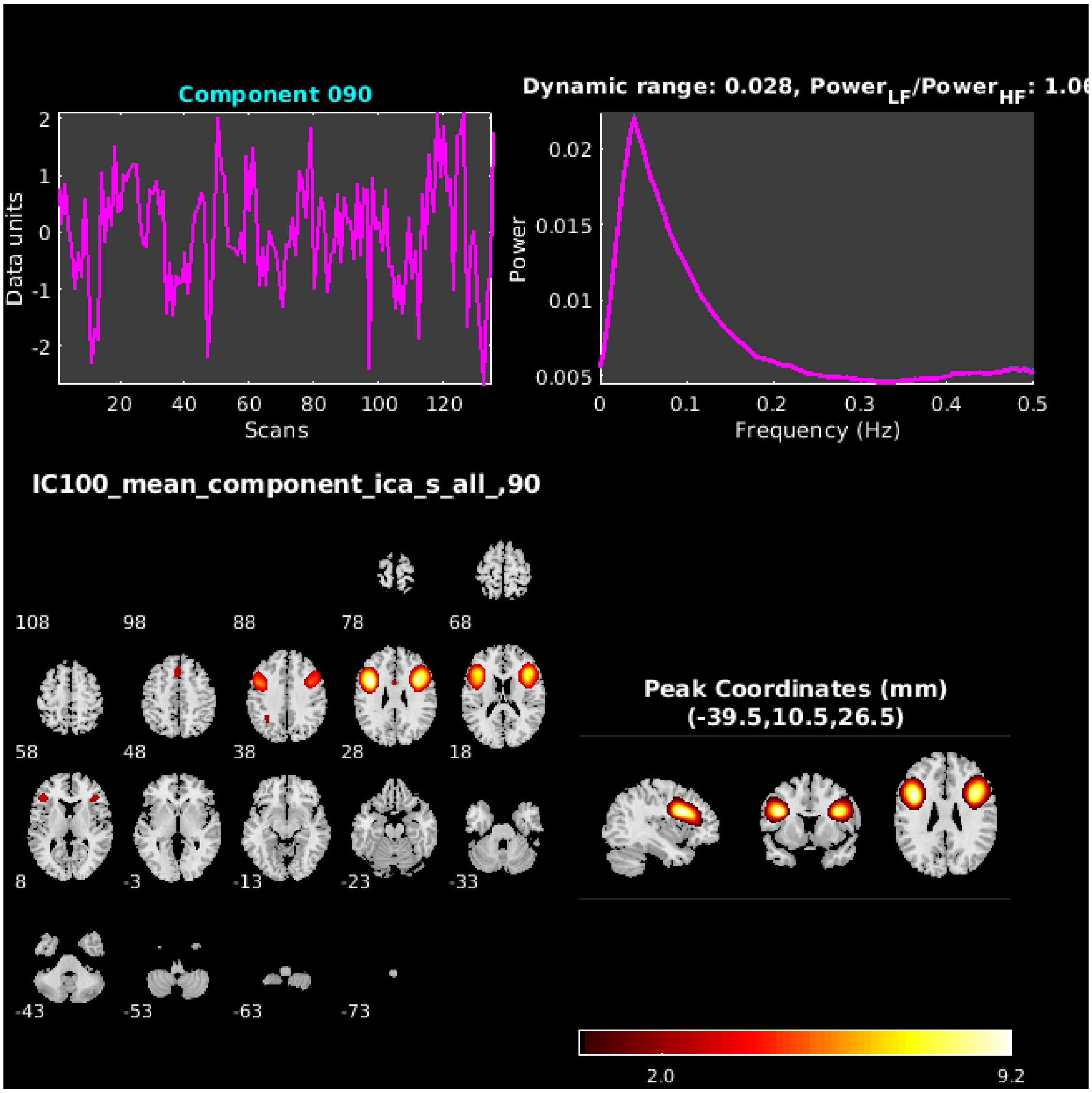
Component 090
Dynamic range: 0.028, Power$_{LF}$/Power$_{HF}$: 1.06
Data units
Scans
Power
Frequency (Hz)
IC100_mean_component_ica_s_all_,90
108 98 88 78 68
58 48 38 28 18
8 -3 -13 -23 -33
-43 -53 -63 -73
Peak Coordinates (mm)
(-39.5,10.5,26.5)
2.0
9.2

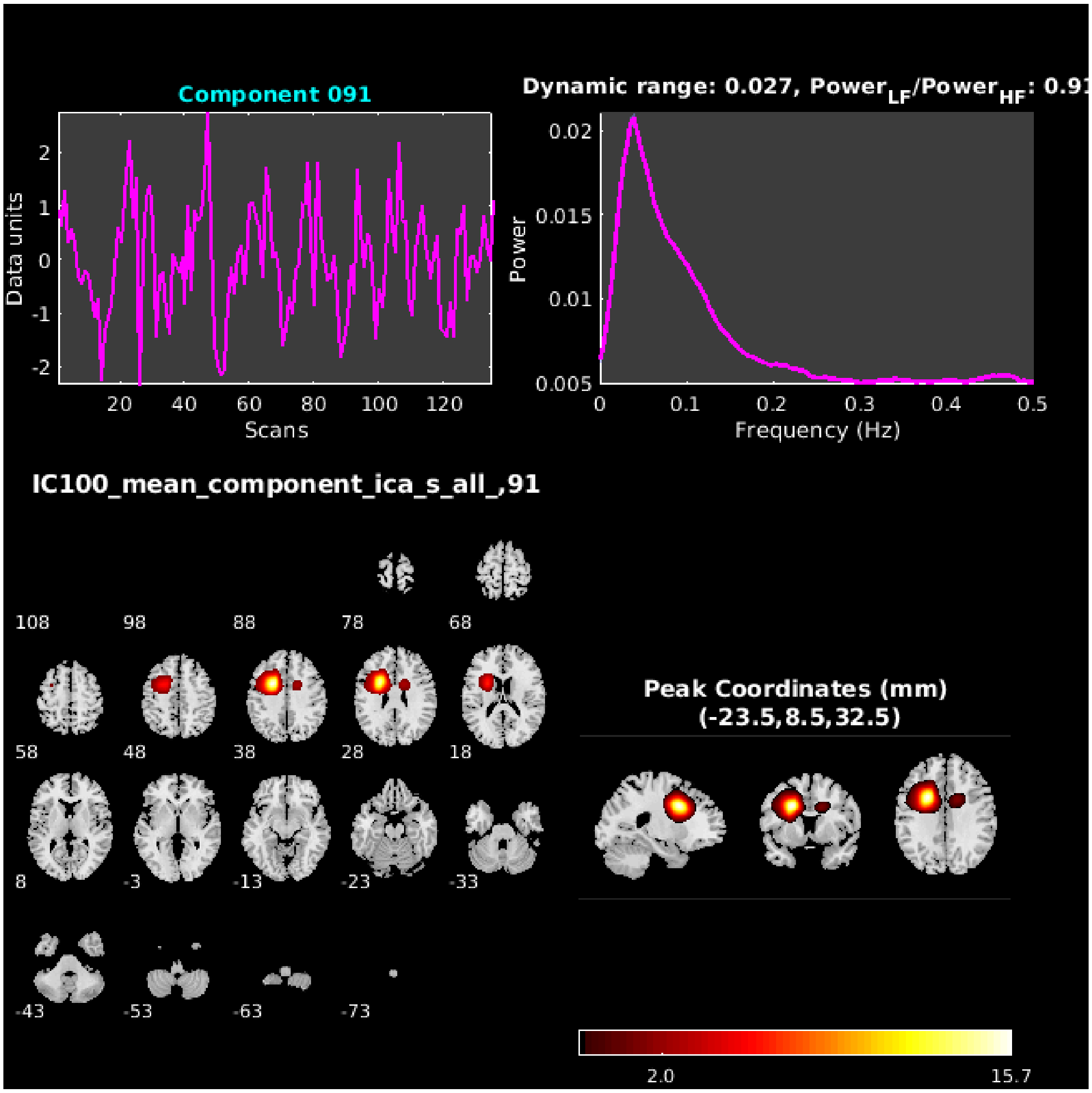

Component 091
Dynamic range: 0.027, Power$_{LF}$/Power$_{HF}$: 0.91
Data units
Scans
Power
Frequency (Hz)
IC100_mean_component_ica_s_all_,91
Peak Coordinates (mm)
(-23.5,8.5,32.5)
2.0
15.7

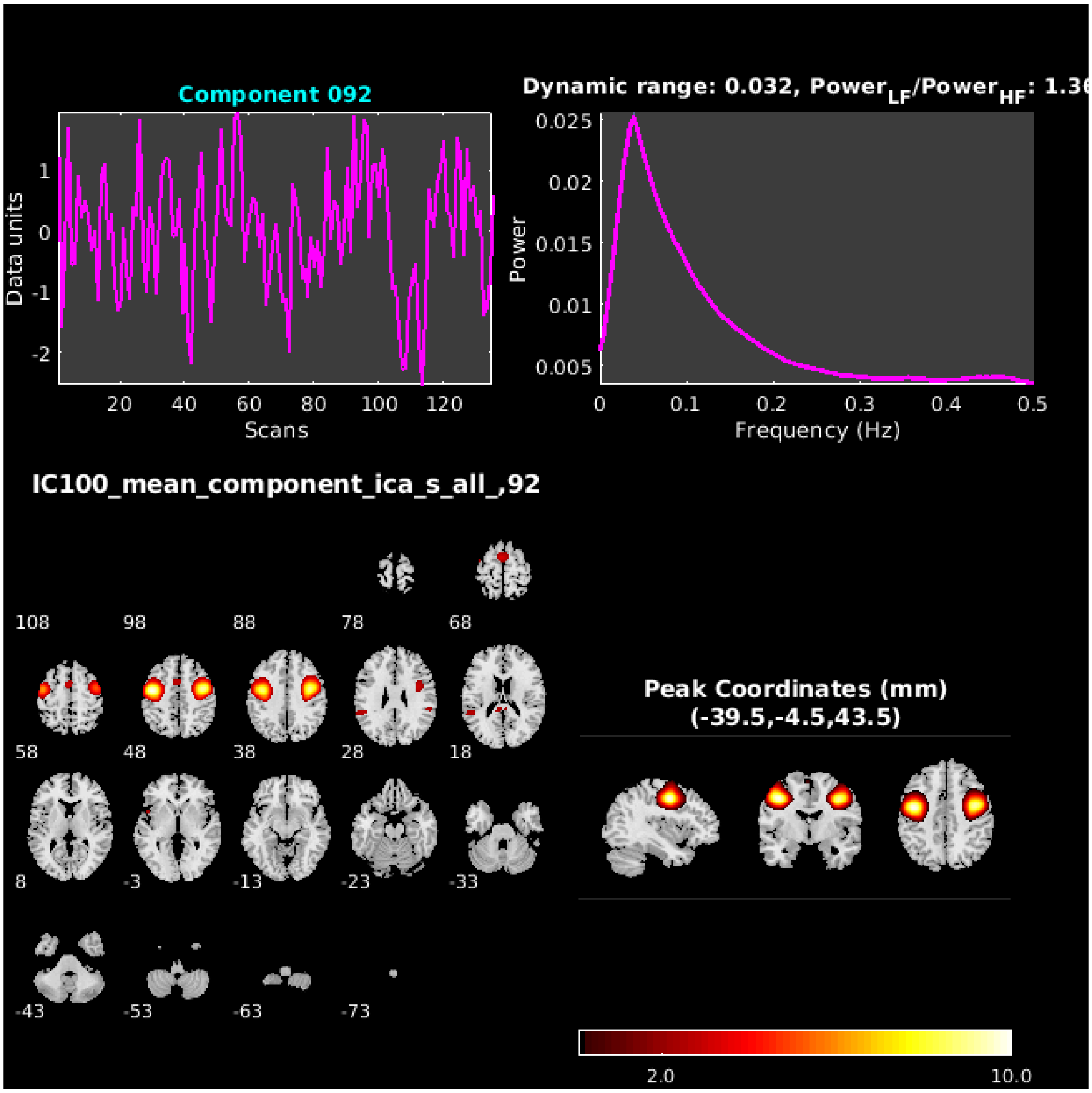
Component 092
Dynamic range: 0.032, $Power_{LF}/Power_{HF}$: 1.36
Data units
Scans
Power
Frequency (Hz)
IC100_mean_component_ica_s_all_,92
Peak Coordinates (mm)
(-39.5,-4.5,43.5)
2.0
10.0

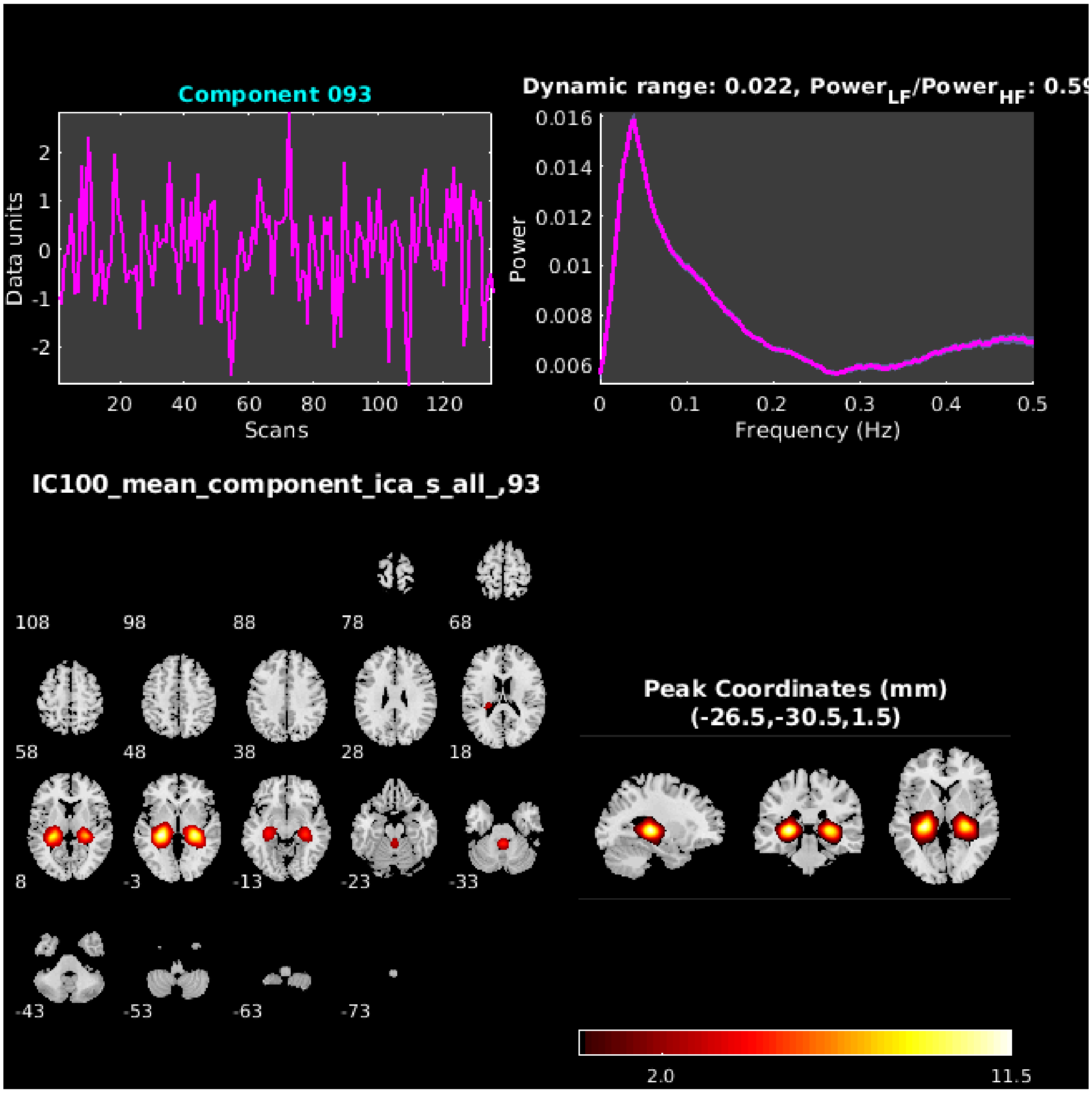
Component 093
Dynamic range: 0.022, Power$_{LF}$/Power$_{HF}$: 0.59
Data units
Scans
Power
Frequency (Hz)
IC100_mean_component_ica_s_all_,93
108
98
88
78
68
58
48
38
28
18
8
-3
-13
-23
-33
-43
-53
-63
-73
Peak Coordinates (mm)
(-26.5,-30.5,1.5)
2.0
11.5

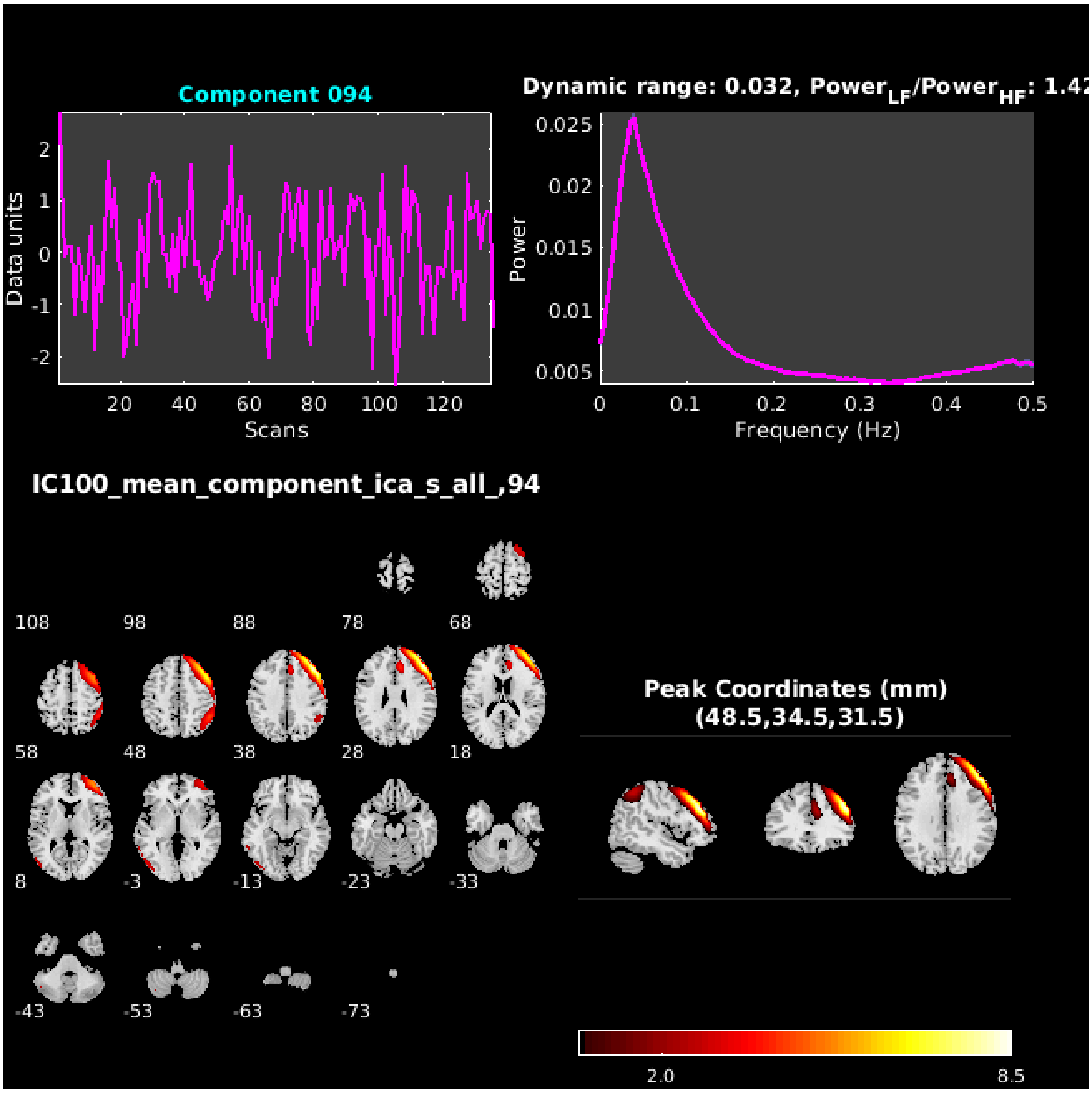
Component 094
Dynamic range: 0.032, Power$_{LF}$/Power$_{HF}$: 1.42
Data units
Scans
Power
Frequency (Hz)
IC100_mean_component_ica_s_all_,94
Peak Coordinates (mm)
(48.5,34.5,31.5)

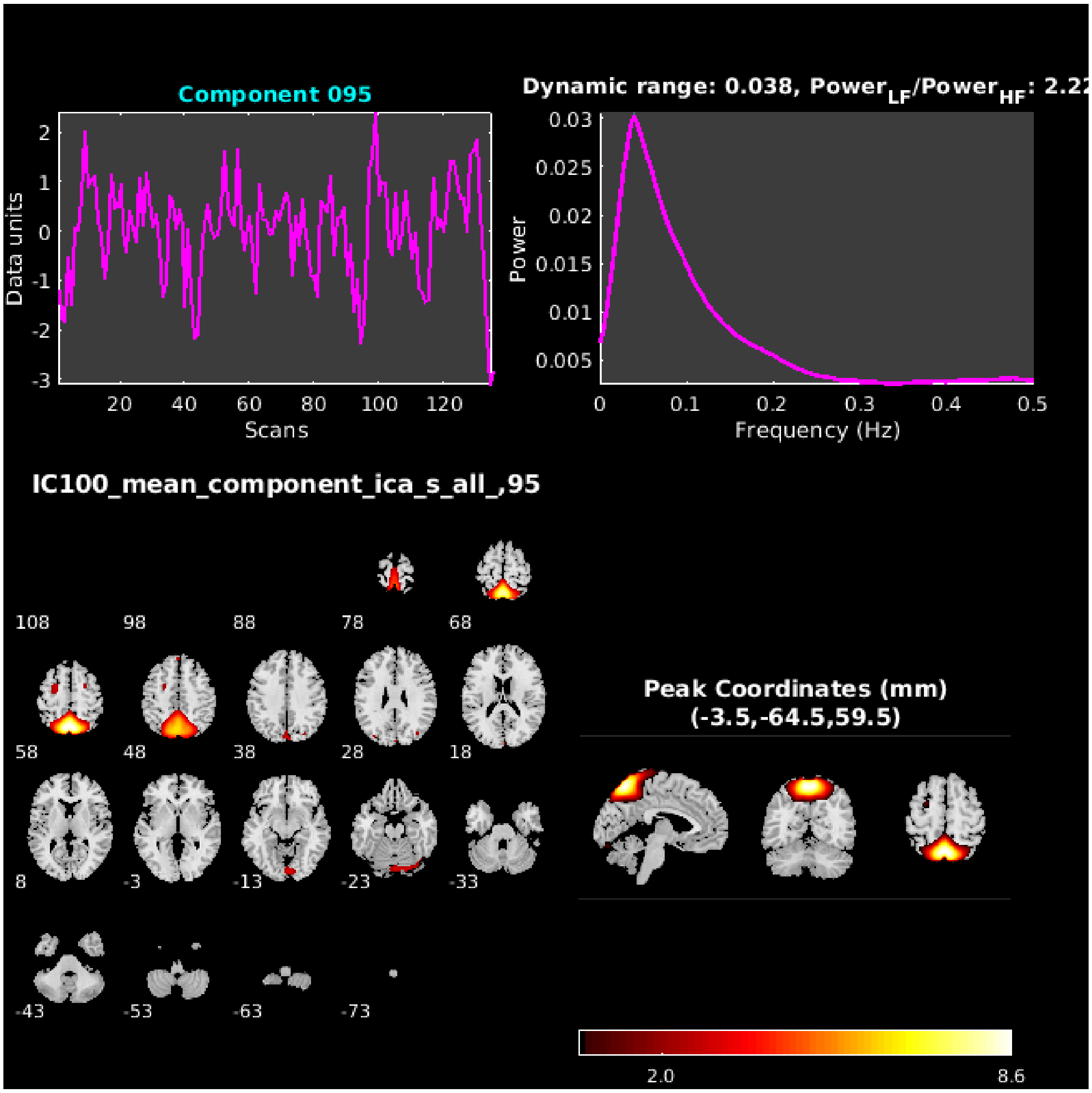
Component 095
Dynamic range: 0.038, Power_LF/Power_HF: 2.22
Data units
Scans
Power
Frequency (Hz)
IC100_mean_component_ica_s_all_,95
108
98
88
78
68
58
48
38
28
18
8
-3
-13
-23
-33
-43
-53
-63
-73
Peak Coordinates (mm)
(-3.5,-64.5,59.5)
2.0
8.6

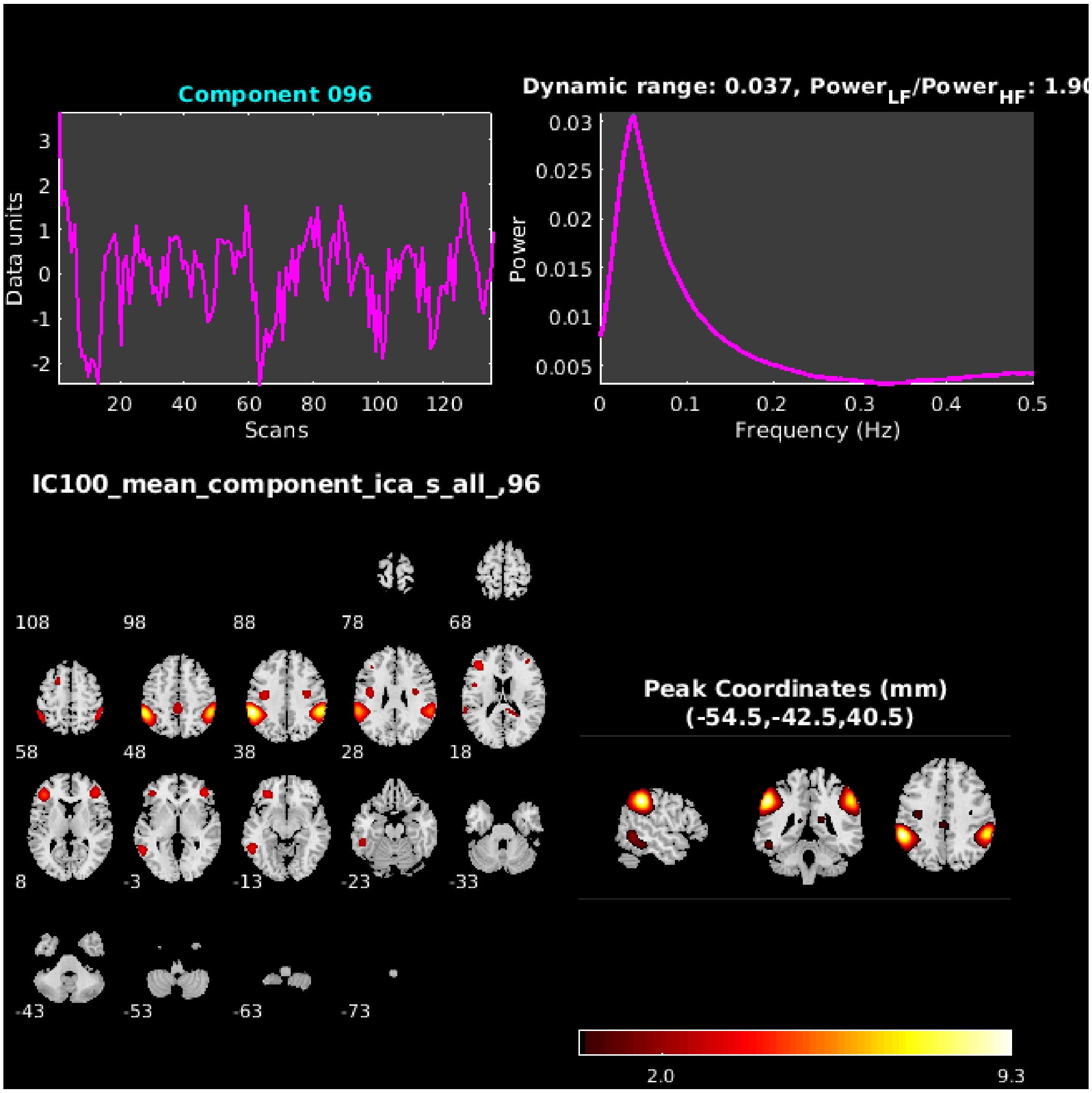
Component 096
Dynamic range: 0.037, Power_LF/Power_HF: 1.9
Data units
Scans
Power
Frequency (Hz)
IC100_mean_component_ica_s_all_,96
Peak Coordinates (mm)
(-54.5,-42.5,40.5)
2.0
9.3

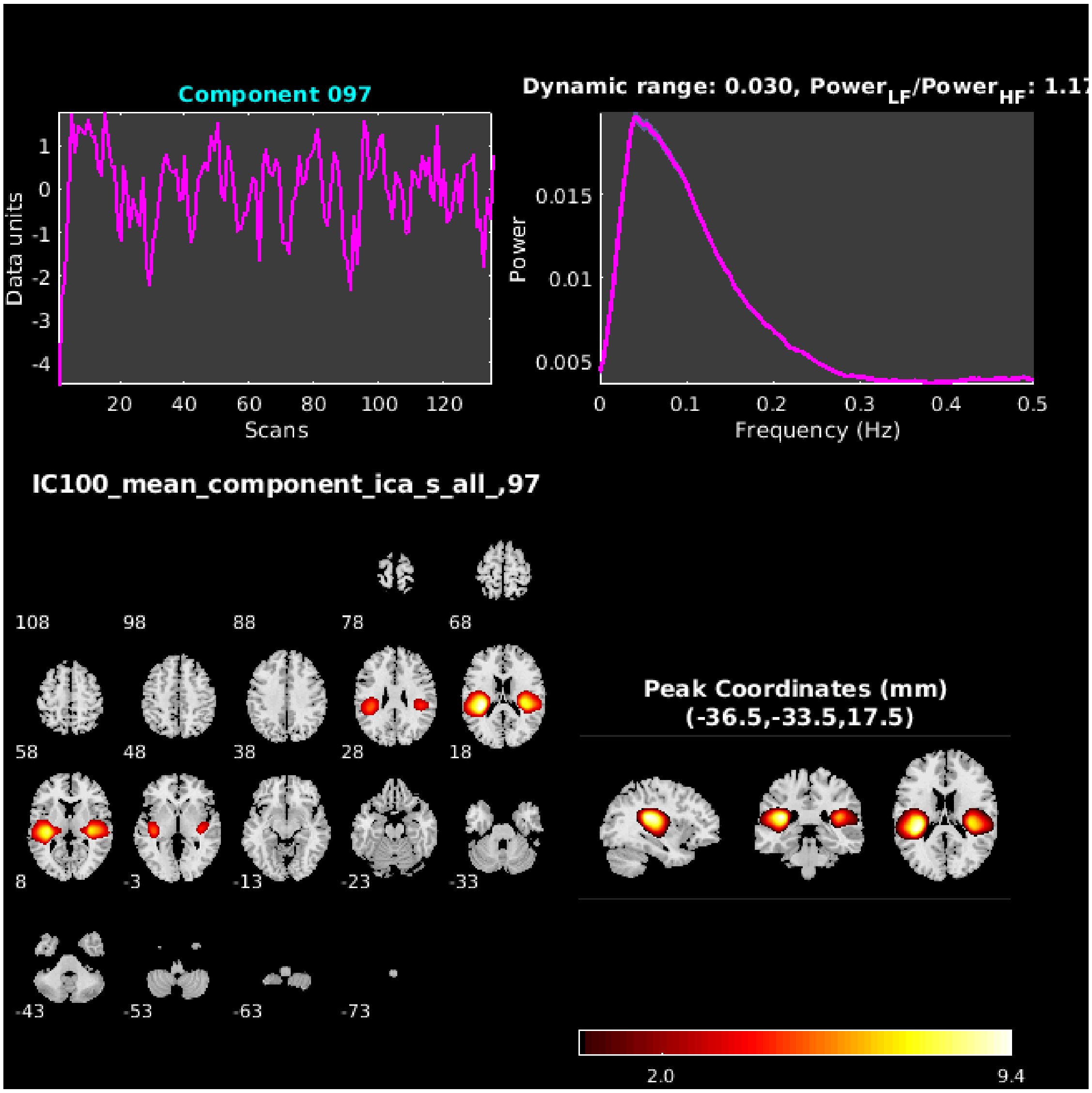
Component 097
Dynamic range: 0.030, Power_LF/Power_HF: 1.17
Data units
Scans
Power
Frequency (Hz)
IC100_mean_component_ica_s_all_,97
Peak Coordinates (mm)
(-36.5,-33.5,17.5)
2.0
9.4

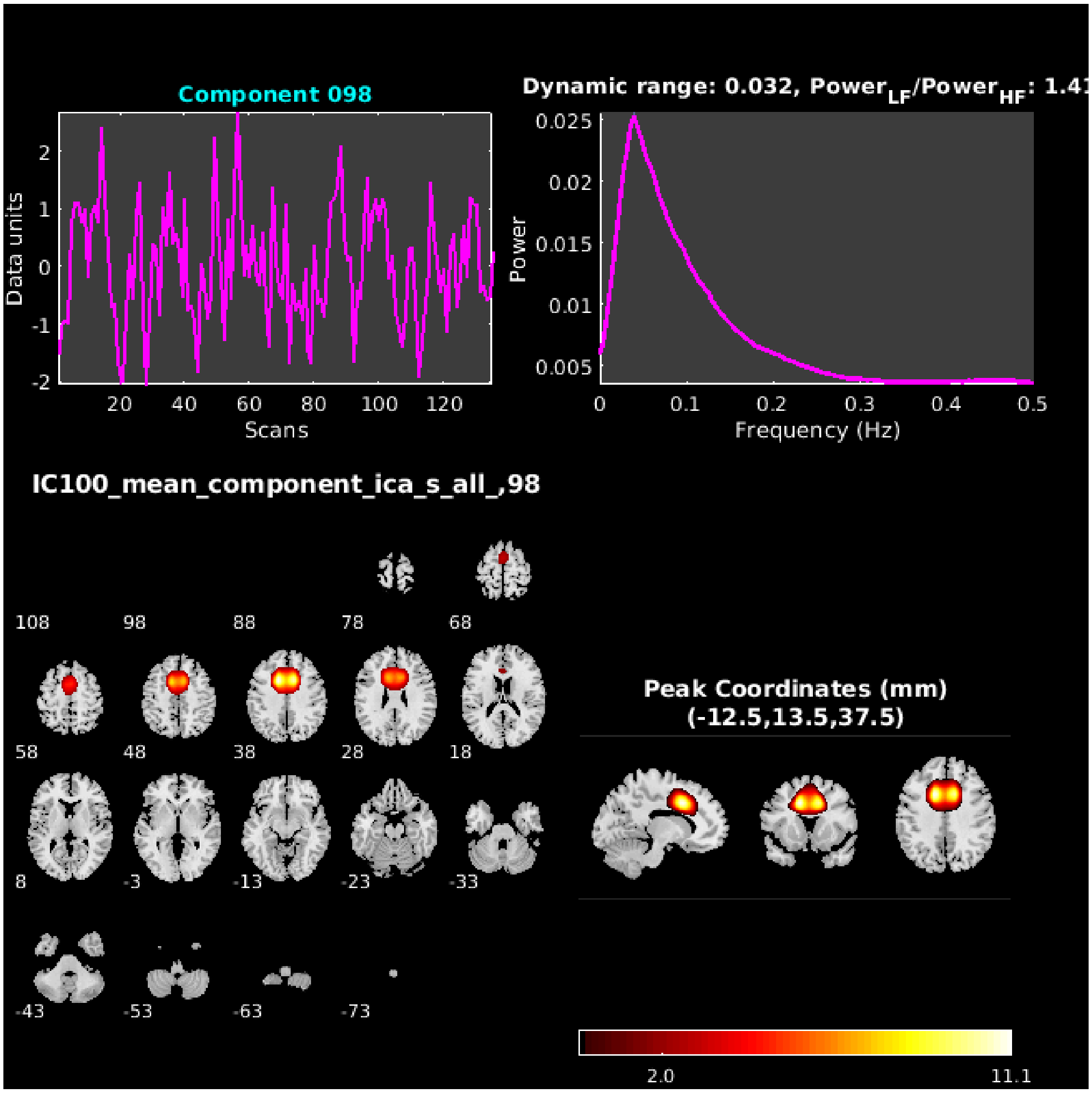

Component 098
Dynamic range: 0.032, Power$_{LF}$/Power$_{HF}$: 1.41
Data units
Scans
Power
Frequency (Hz)
IC100_mean_component_ica_s_all_,98
Peak Coordinates (mm)
(-12.5,13.5,37.5)
2.0
11.1

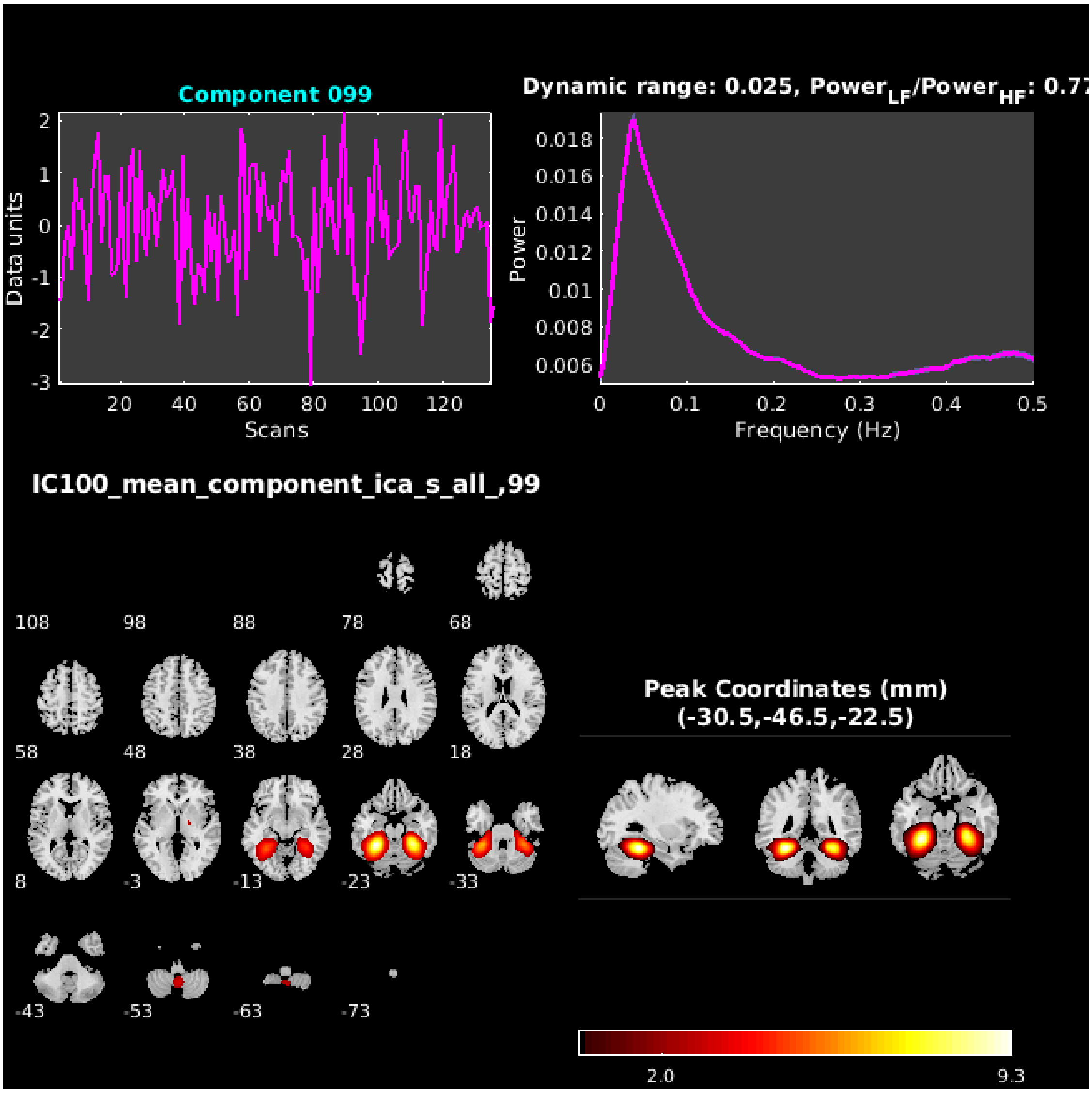
Component 099
Dynamic range: 0.025, Power$_{LF}$/Power$_{HF}$: 0.77
Data units
Scans
Power
Frequency (Hz)
IC100_mean_component_ica_s_all_,99
Peak Coordinates (mm)
(-30.5,-46.5,-22.5)
2.0
9.3

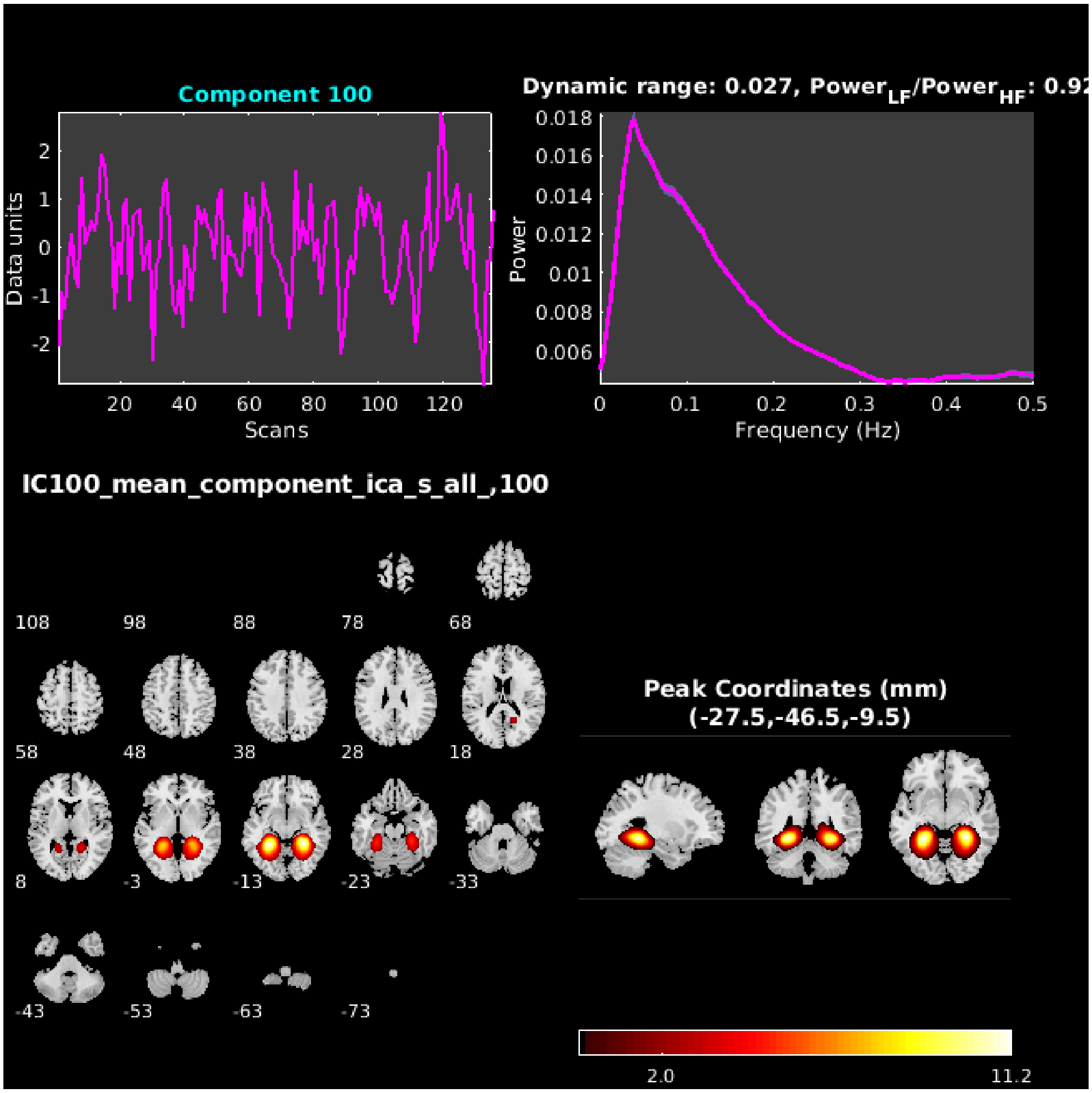

Spectral Summary

- **a) dynamic_range** - Difference between the peak power and minimum power at frequencies to the right of the peak.
- **b) fALFF** - Low frequency to high frequency power ratio.

ComponentNumber	*DynamicRange*	*fALFF*
1	*0.024928*	*0.73197*
2	*0.035478*	*1.9797*
3	*0.022081*	*0.54479*
4	*0.033594*	*1.6501*
5	*0.028119*	*0.88107*
6	*0.023493*	*0.56895*
7	*0.023267*	*0.59653*

8	0.025609	0.75782
9	0.026897	0.8537
10	0.027453	0.86857
11	0.036853	2.2174
12	0.024085	0.4716
13	0.024801	0.7499
14	0.036431	2.1254
15	0.025182	0.67299
16	0.022628	0.56008
17	0.022311	0.58257
18	0.027535	0.8424
19	0.0272	0.80071
20	0.038997	2.7214
21	0.033079	1.3711
22	0.027717	0.86612
23	0.026856	0.89157
24	0.023937	0.68278
25	0.034155	1.7223
26	0.031531	1.3372
27	0.032404	1.6364
28	0.028593	0.98148
29	0.033101	1.7018
30	0.03049	1.1618
31	0.025108	0.67728
32	0.028188	0.9868
33	0.023018	0.60217
34	0.036126	1.8157
35	0.026205	0.82158
36	0.027763	1.0216
37	0.024029	0.65991
38	0.035319	1.8202
39	0.02211	0.55217
40	0.031633	1.3796
41	0.024007	0.69533
42	0.025564	0.80575
43	0.026288	0.77471
44	0.026845	0.91672
45	0.02787	0.93736
46	0.024882	0.75031
47	0.022528	0.57491
48	0.024394	0.7399
49	0.024713	0.69014
50	0.031988	1.5687
51	0.03165	1.3758
52	0.025375	0.81817
53	0.025322	0.8437
54	0.022826	0.61196
55	0.02911	1.0426
56	0.026868	0.89876
57	0.029665	1.2054
58	0.027645	0.89628
59	0.022283	0.51832
60	0.027507	0.97585
61	0.036443	1.877

62	0.031413	1.3659
63	0.028485	0.99606
64	0.024927	0.70077
65	0.03686	1.9848
66	0.026913	0.79479
67	0.022663	0.62129
68	0.033386	1.6591
69	0.024535	0.6532
70	0.035691	1.8997
71	0.027414	0.93362
72	0.026965	0.91951
73	0.031025	1.2432
74	0.022446	0.57984
75	0.023982	0.60055
76	0.029295	1.0368
77	0.025278	0.68392
78	0.024106	0.56446
79	0.025645	0.87883
80	0.026143	0.87147
81	0.034273	1.6985
82	0.034932	1.7263
83	0.028043	0.99167
84	0.028761	1.061
85	0.028031	1.0132
86	0.026832	0.80272
87	0.031114	1.1835
88	0.026839	0.85843
89	0.034907	1.7487
90	0.02835	1.0632
91	0.026871	0.91593
92	0.031875	1.3653
93	0.02213	0.59078
94	0.031845	1.4237
95	0.037849	2.2204
96	0.036508	1.9007
97	0.029662	1.1699
98	0.032019	1.4159
99	0.02532	0.77876
100	0.026982	0.92611

Temporal Stats On Beta Weights

Multiple regression is done using the timecourses from SPM design matrix as model and ICA timecourses as observations. R^2 values for each component are shown in bar plot. For each component, one sample t-test results of each session and condition are shown in the bar plots.

Kurtosis of timecourses and spatial maps

Mean across subjects is reported in table. Figure shows mean+/- SEM across subjects

ComponentNumber	*Timecourses*	*SpatialMaps*

1	3.7453	9.8839
2	4.1276	6.2088
3	4.3457	5.7692
4	4.3189	5.1809
5	4.9107	5.6255
6	3.5921	8.0855
7	3.9501	5.9133
8	4.3862	4.8823
9	4.4145	4.845
10	4.574	4.8038
11	4.2033	4.9988
12	5.2255	5.1374
13	4.2557	4.4265
14	4.1158	5.0716
15	4.3905	5.1912
16	4.1641	5.4619
17	4.5598	5.2936
18	4.065	4.7386
19	4.846	4.8598
20	4.0577	4.2761
21	5.0485	4.6869
22	4.4493	4.3302
23	5.0092	4.5777
24	3.8651	5.4775
25	4.0915	4.6062
26	4.5491	4.1816
27	4.52	4.9188
28	3.8157	5.7187
29	3.9062	4.3898
30	5.5276	4.2415
31	4.6377	4.8312
32	4.0159	4.8889
33	3.9086	4.8124
34	4.067	4.2051
35	4.2707	4.724
36	4.5063	4.4168
37	3.9958	5.3943
38	3.4534	4.6506
39	3.8068	5.1617
40	4.6157	4.3084
41	3.7368	4.7129
42	3.9473	4.849
43	4.5481	4.1424
44	4.2611	5.5129
45	4.3446	5.0009
46	3.7295	5.1863
47	4.2741	5.3441
48	4.2673	4.4304
49	5.166	4.25
50	4.3614	4.2255
51	4.3136	4.1048
52	4.2838	4.5992
53	4.3799	4.2666

54	4.8566	4.2273
55	5.3177	4.3824
56	3.6882	4.5387
57	4.8207	4.223
58	4.446	4.8961
59	5.7756	4.3868
60	4.7622	3.9921
61	3.9146	4.0696
62	3.9679	4.5039
63	4.194	5.1242
64	4.4265	4.4661
65	3.7664	4.0193
66	4.7373	3.9405
67	4.0807	5.0595
68	3.8874	4.0659
69	4.6029	4.5587
70	4.8101	4.0225
71	5.0382	4.3197
72	4.0701	5.0794
73	5.125	4.2404
74	4.038	4.1939
75	6.1368	4.1921
76	3.9725	5.0846
77	4.5538	4.2326
78	4.547	4.1627
79	3.9359	4.2202
80	4.1832	4.5073
81	3.8735	4.1932
82	3.8871	4.1329
83	4.8896	4.2882
84	4.9354	4.1328
85	4.1589	4.1841
86	5.2549	4.011
87	4.184	4.1895
88	4.4249	4.7286
89	3.588	4.0345
90	3.8648	4.1467
91	4.0257	5.1858
92	3.6683	4.0127
93	4.045	4.1895
94	4.3824	3.9196
95	3.4031	3.9532
96	3.4443	3.8453
97	3.7913	4.1741
98	3.5504	4.3516
99	4.5318	4.0219
100	4.1449	4.3482

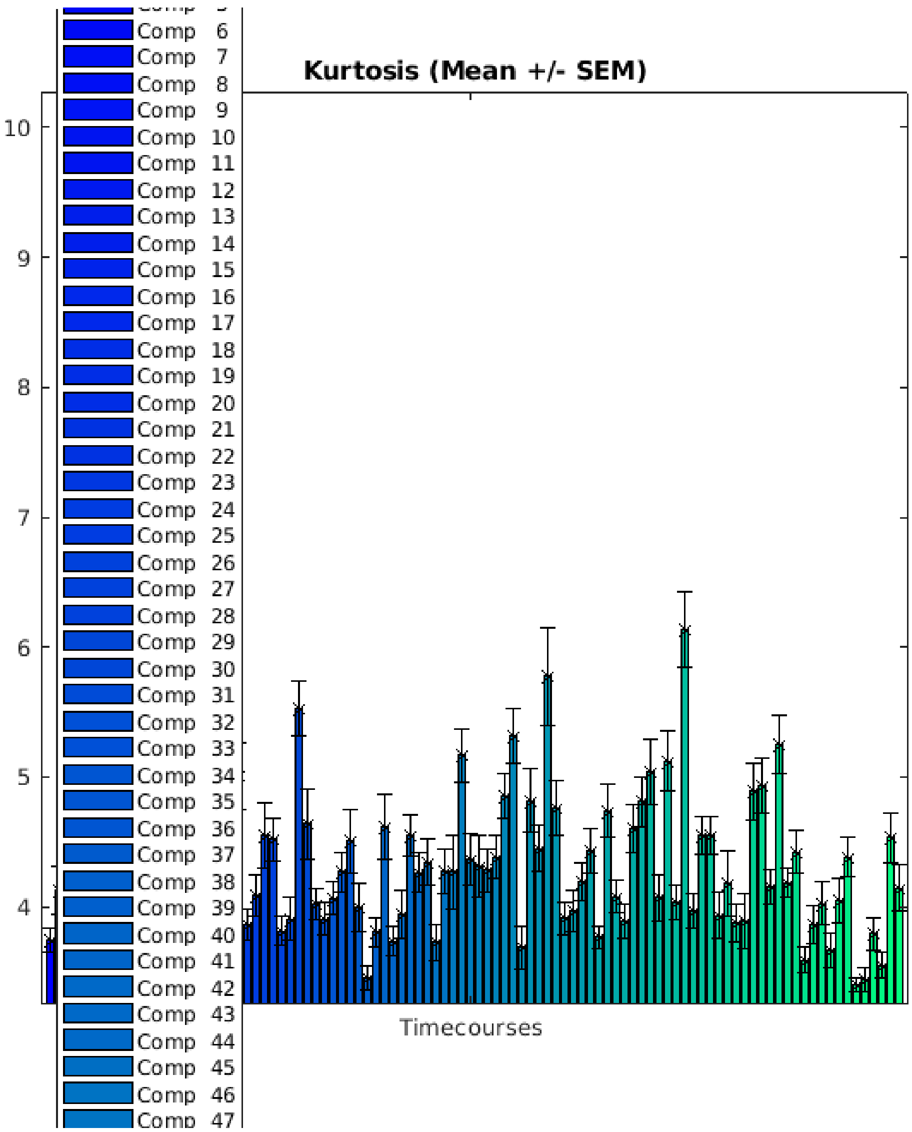
Kurtosis (Mean +/- SEM)
10
9
8
7
6
5
4
Timecourses
Comp 6
Comp 7
Comp 8
Comp 9
Comp 10
Comp 11
Comp 12
Comp 13
Comp 14
Comp 15
Comp 16
Comp 17
Comp 18
Comp 19
Comp 20
Comp 21
Comp 22
Comp 23
Comp 24
Comp 25
Comp 26
Comp 27
Comp 28
Comp 29
Comp 30
Comp 31
Comp 32
Comp 33
Comp 34
Comp 35
Comp 36
Comp 37
Comp 38
Comp 39
Comp 40
Comp 41
Comp 42
Comp 43
Comp 44
Comp 45
Comp 46
Comp 47

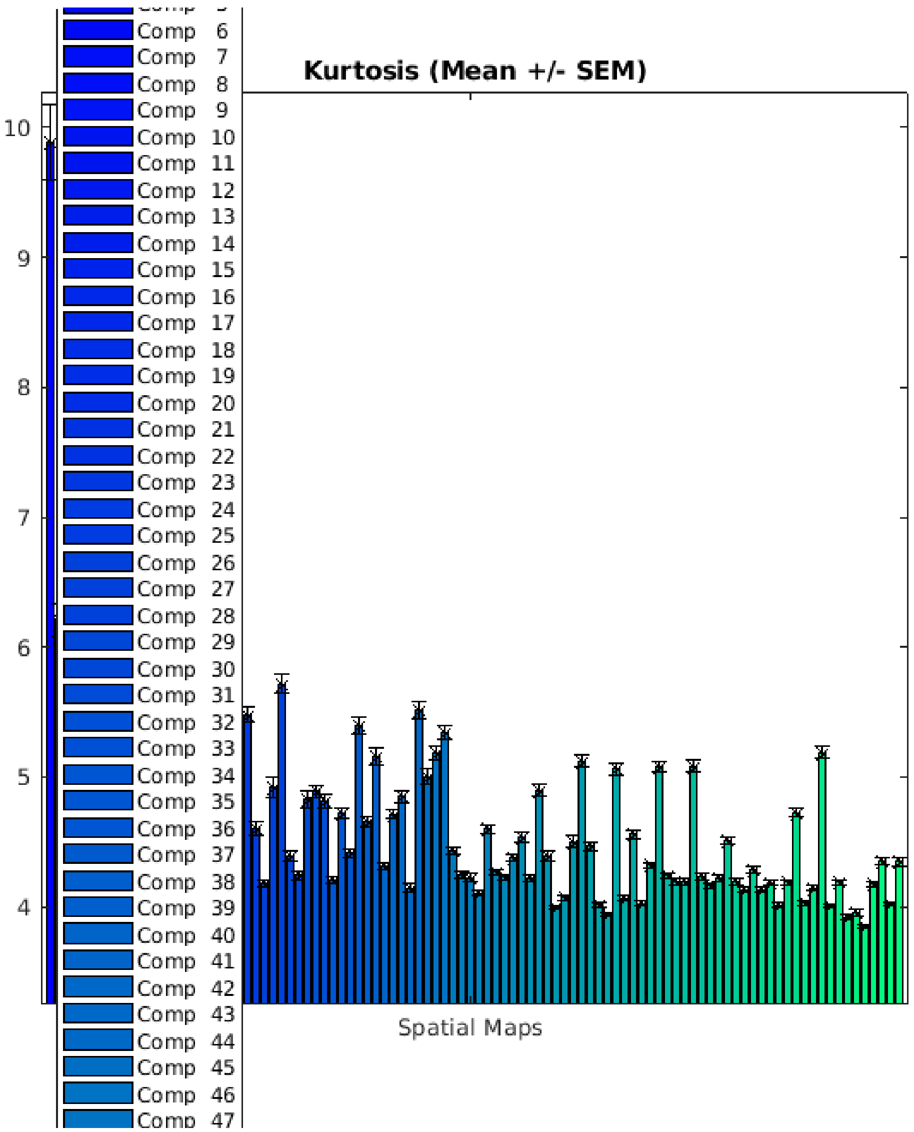

FNC correlations

Functional network connectivity correlations are computed for each data-set and averaged across sessions.

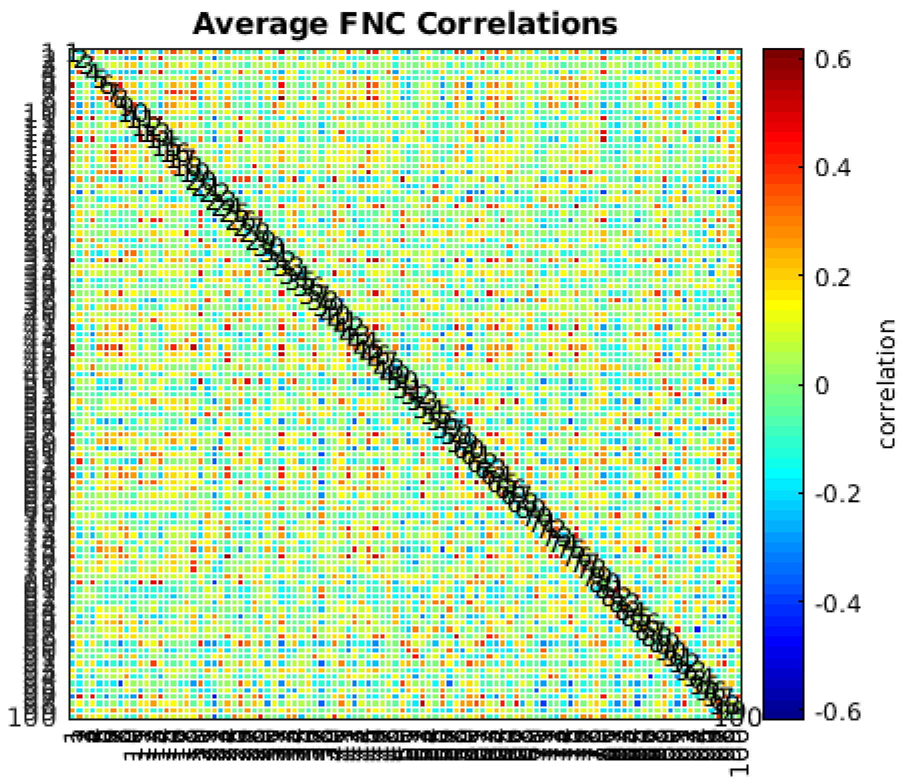

FNC metrics of component spatial maps

Mutual information is computed between components spatially and averaged across data-sets.

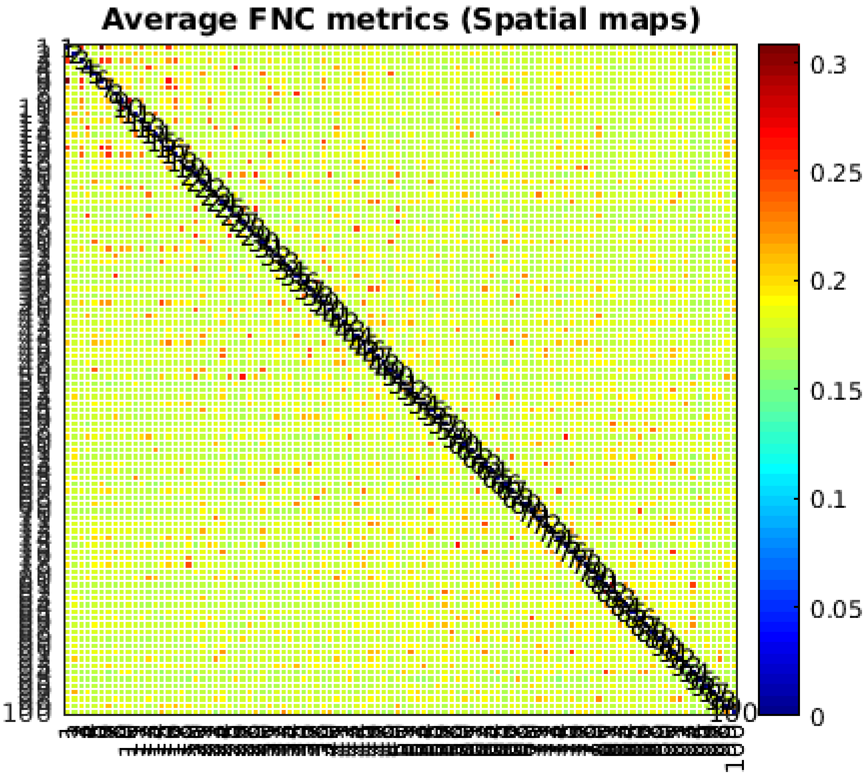

Published with MATLAB® R2019a
