# Supplementary material for: Multi-spatial-scale dynamic interactions between functional sources reveal sex-specific changes in schizophrenia
Source: Netw Neurosci. 2022 Jun 1;6(2):357–81. doi: 10.1162/netn_a_00196 (PMC9208002; doi:10.1162/netn_a_00196)
Supplement: Supplementary file 1 [file netn-06-357-s001.zip › icatb_gica_html_report25.pdf]

Table of Contents

.... 1
Group ICA Parameters 1
ICASSO Plots 2
Mean Components 5
Spectral Summary 29
Temporal Stats On Beta Weights 30
Kurtosis of timecourses and spatial maps 30
FNC correlations 33
FNC metrics of component spatial maps 34

Group ICA Parameters

...

Number of Subjects : 856

Number of Sessions : 1

Number of Independent Components : 25

ICA Algorithm : Infomax

Number Of Scans/Timepoints : 157

Mask File : mask_common

Data Pre-processing Type : Variance Normalization

PCA Type : Standard

Group PCA Type : Subject Specific

Group ICA Type : Spatial

Back Reconstruction Type : Spatial-temporal Regression

Scaling Components : Z-scores

Stability analysis type : ICASSO

Group analysis mode: Parallel

*Anatomical file: /trdapps/linux-x86_64/matlab/toolboxes/
GroupICATv4.0b/icatb/icatb_templates/ch2bet.nii
Slice Plane: Axial*

Image values: Positive

Convert to Z-scores: yes

Threshold: 1.96

..

ICASSO Plots

Warning: Creates overwhelming number of lines
Warning: Tries to change the limit...
Warning: New limit =0.99759

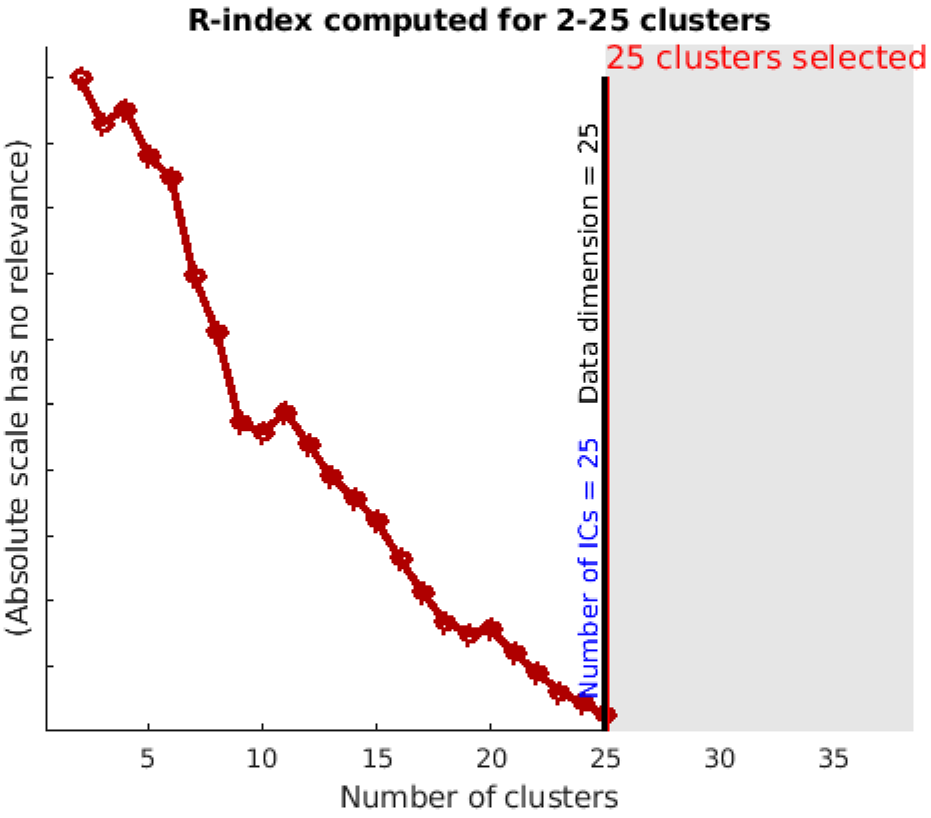

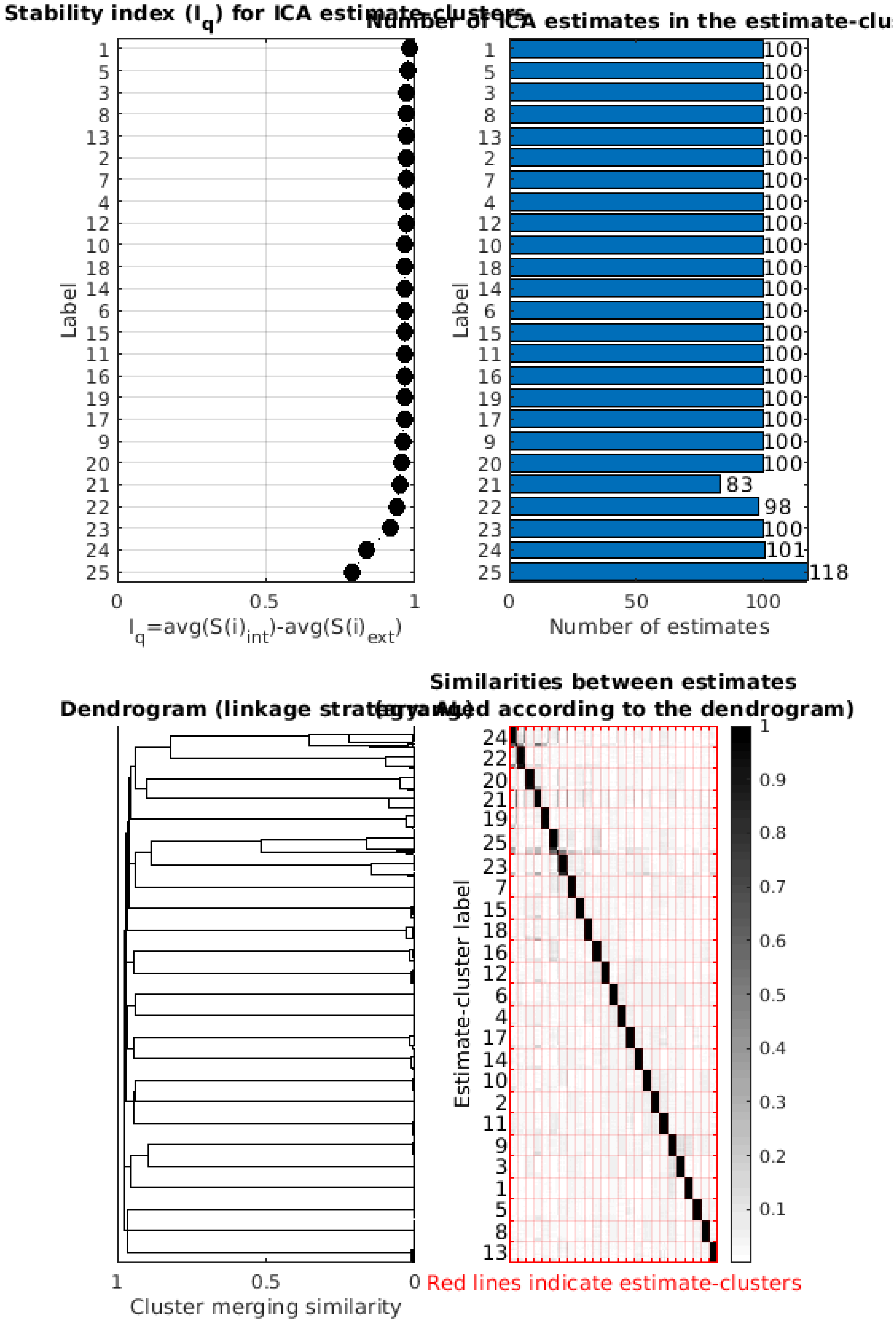
Stability index (I_q) for ICA estimate-clusters
Number of ICA estimates in the estimate-clu
Label
I_q=avg(S(i)$_{int}$)-avg(S(i)$_{ext}$)
Number of estimates
Dendrogram (linkage strategy: AL)
Similarities between estimates
(arranged according to the dendrogram)
Cluster merging similarity
Estimate-cluster label
Red lines indicate estimate-clusters

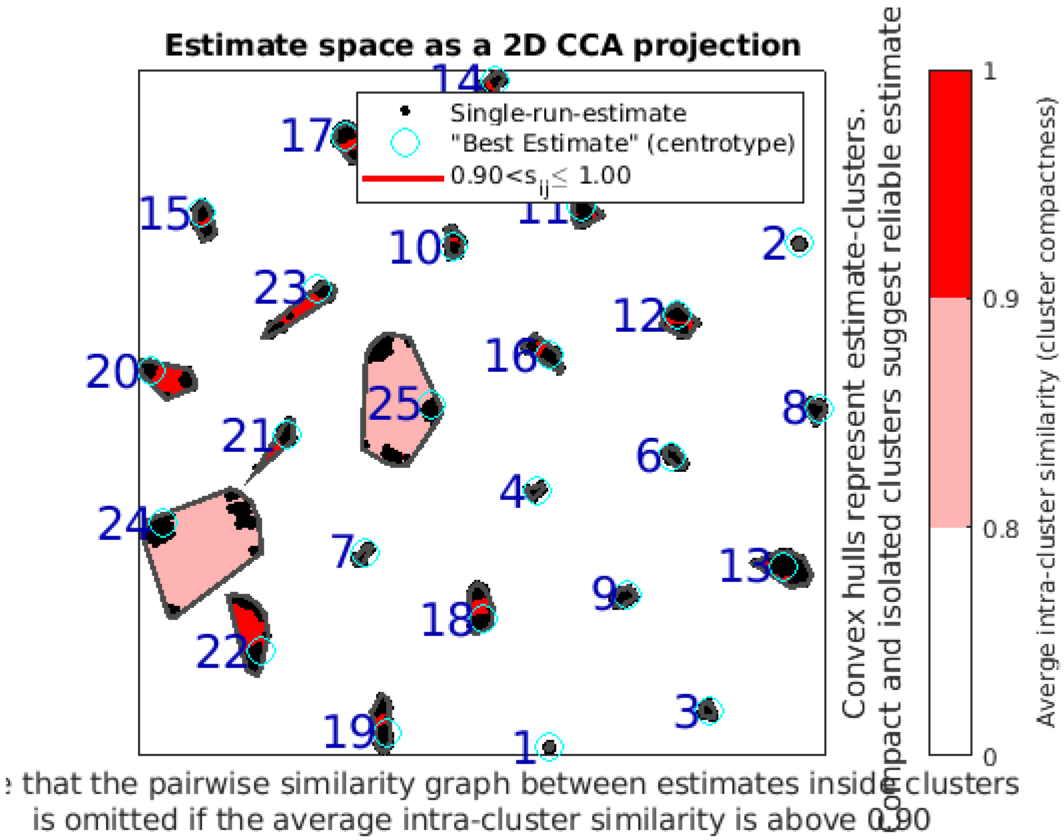
Estimate space as a 2D CCA projection
Single-run-estimate
"Best Estimate" (centrotype)
0.90<s_ij≤ 1.00
Convex hulls represent estimate-clusters.
Compact and isolated clusters suggest reliable estimate
Averge intra-cluster similarity (cluster compactness)
1
0.9
0.8
0
14
17
15
10
11
2
23
12
20
16
25
8
21
6
4
24
7
13
9
18
22
19
1
3
e that the pairwise similarity graph between estimates inside clusters
is omitted if the average intra-cluster similarity is above 0.90

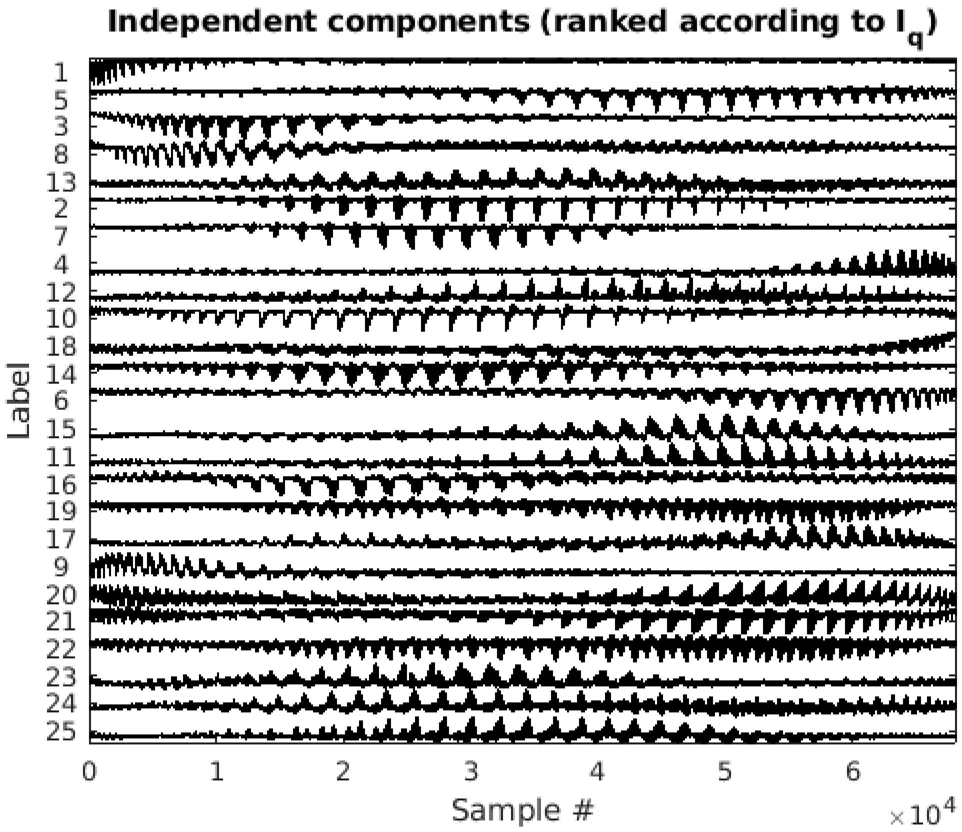
Independent components (ranked according to I_q)
Label
1
5
3
8
13
2
7
4
12
10
18
14
6
15
11
16
19
17
9
20
21
22
23
24
25
0
1
2
3
4
5
6
Sample #
×10^4

Mean Components

Mean across all subjects and sessions is computed for each component

- **a) Timecourse** - Mean timecourse is converted to z-scores.
- **b) Spectra** - Timecourses spectra is computed for each data-set and averaged across sessions. Mean and standard error of mean is shown in the figure.
- **c) Montage** - Axial slices are shown.
- **d) Ortho slices** - Ortho plot is shown for the peak voxel and coordinates are reported.

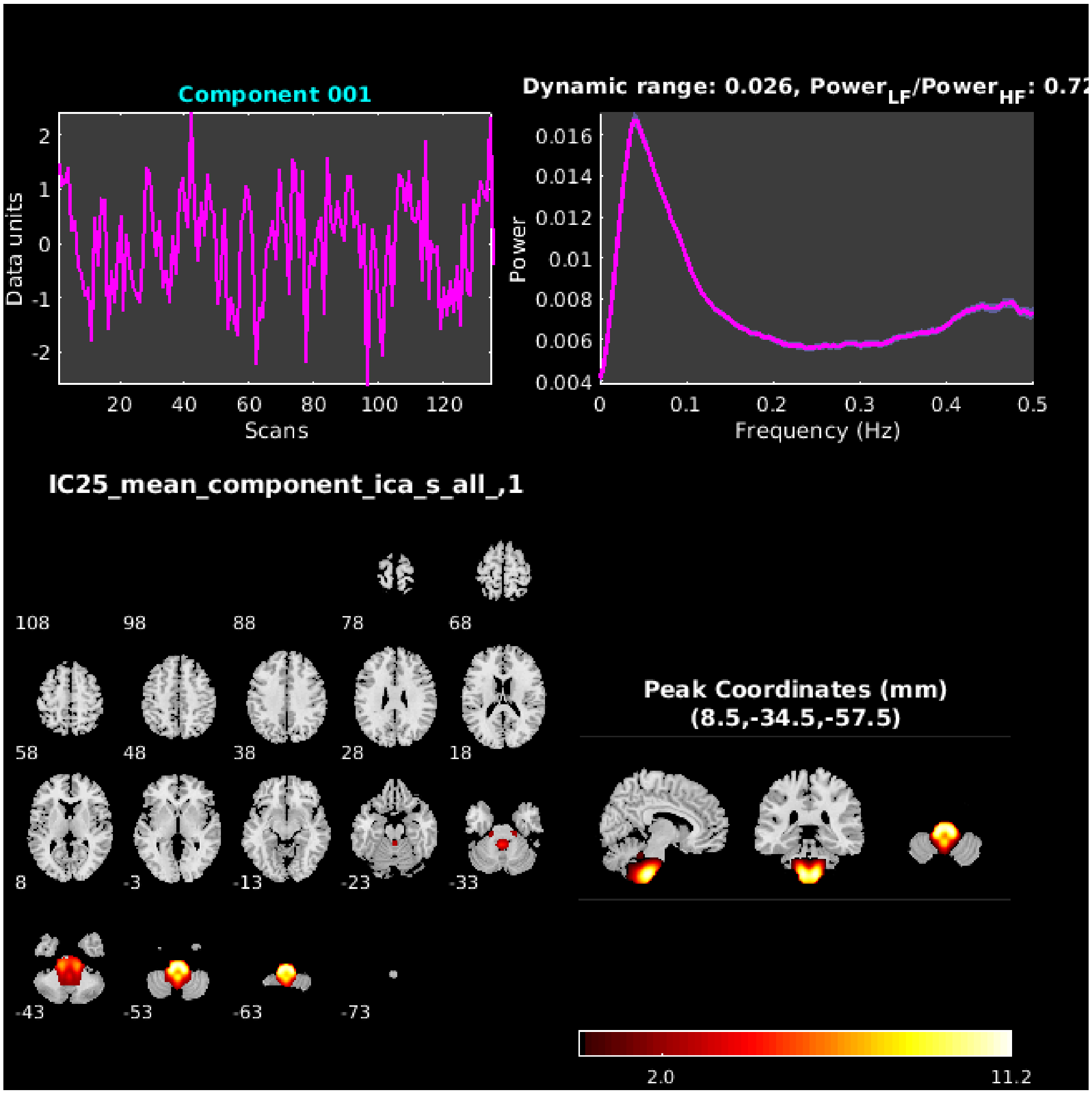

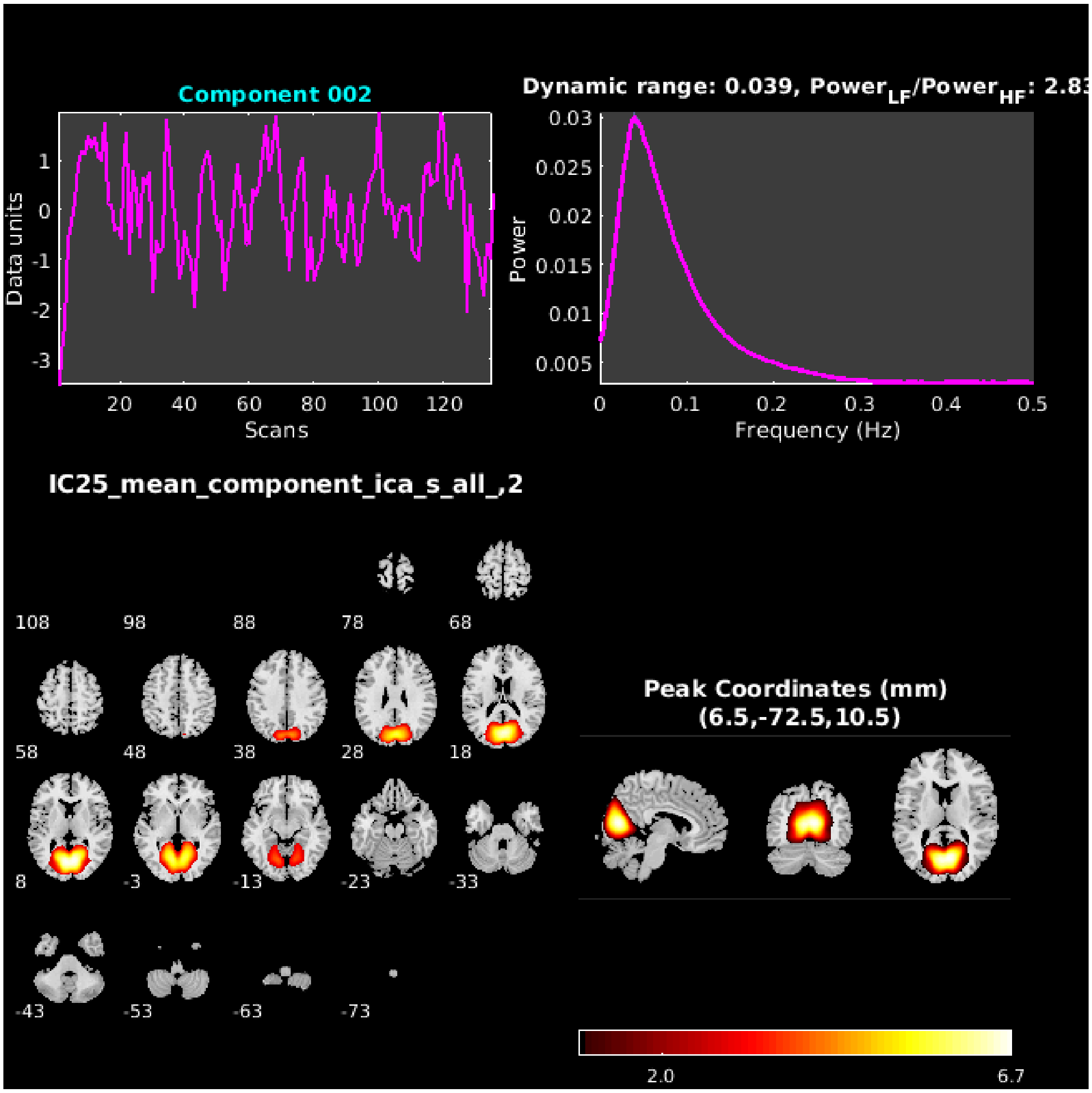

Component 002
Data units
Scans
Dynamic range: 0.039, Power_LF/Power_HF: 2.83
Power
Frequency (Hz)
IC25_mean_component_ica_s_all_,2
Peak Coordinates (mm)
(6.5,-72.5,10.5)
2.0
6.7

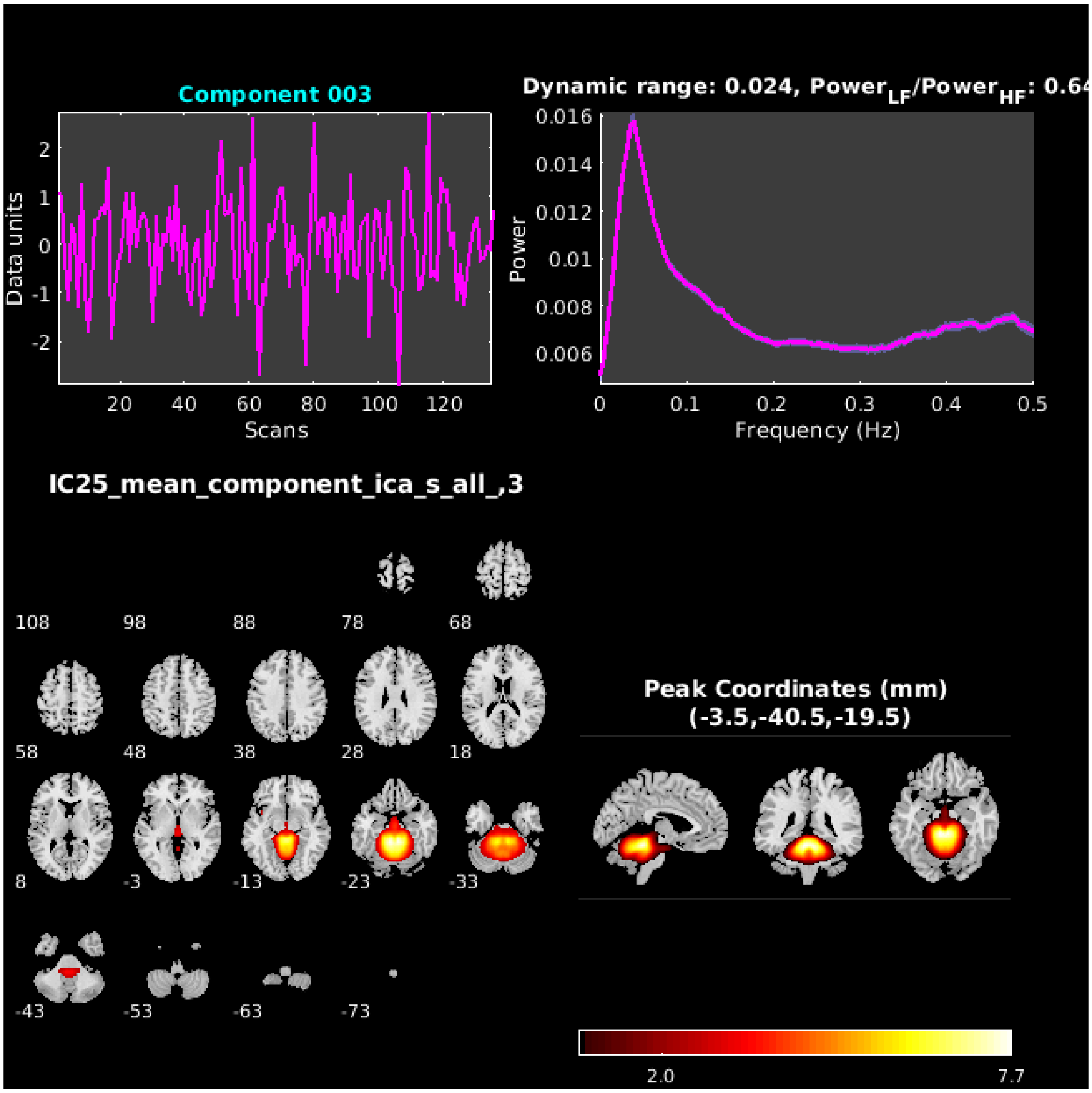
Component 003
Dynamic range: 0.024, Power_LF/Power_HF: 0.64
Data units
Scans
Power
Frequency (Hz)
IC25_mean_component_ica_s_all_,3
Peak Coordinates (mm)
(-3.5,-40.5,-19.5)
2.0
7.7

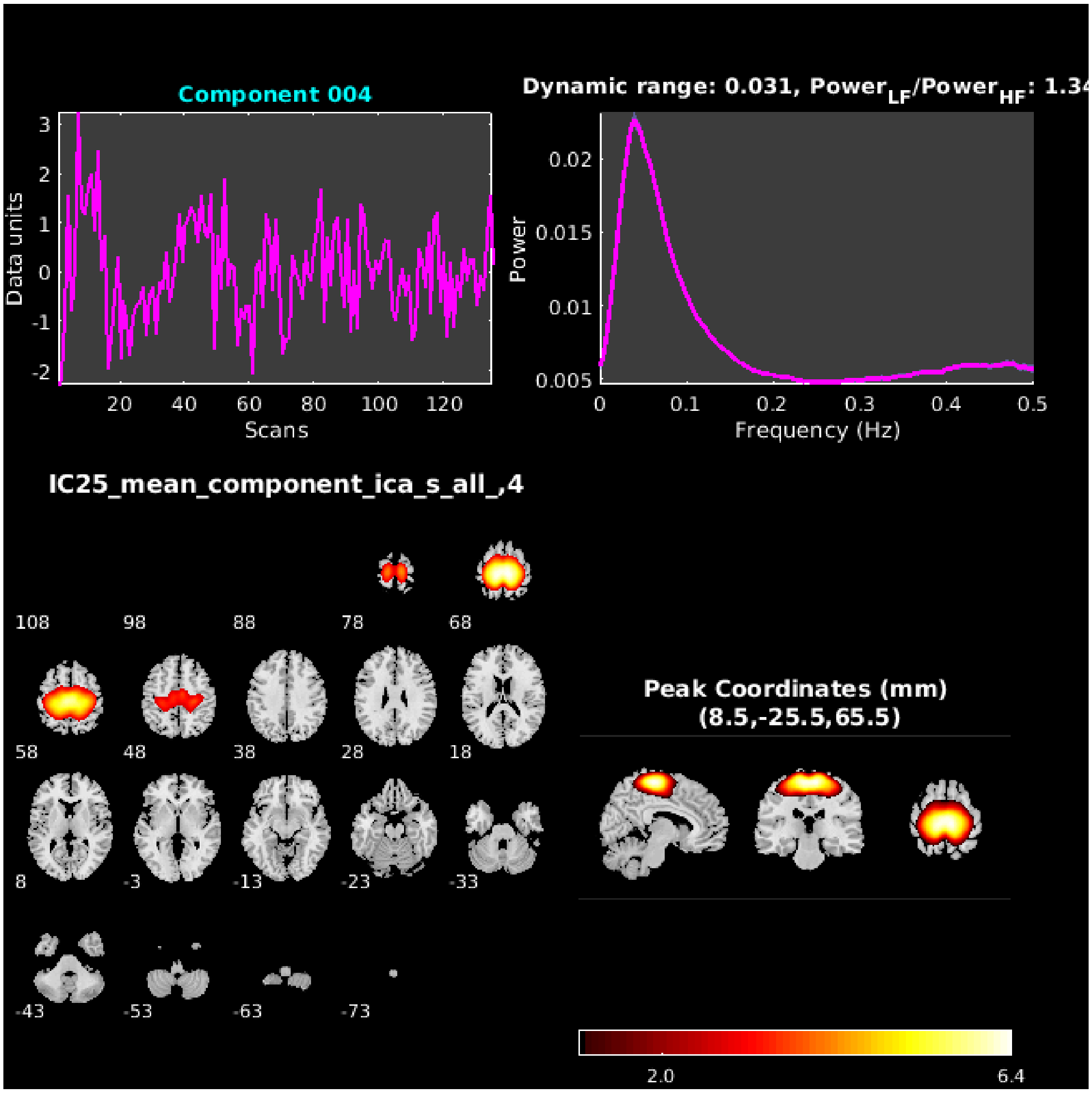

Component 004
Dynamic range: 0.031, Power$_{LF}$/Power$_{HF}$: 1.34
Data units
Scans
Power
Frequency (Hz)
IC25_mean_component_ica_s_all_,4
108
98
88
78
68
58
48
38
28
18
8
-3
-13
-23
-33
-43
-53
-63
-73
Peak Coordinates (mm)
(8.5,-25.5,65.5)
2.0
6.4

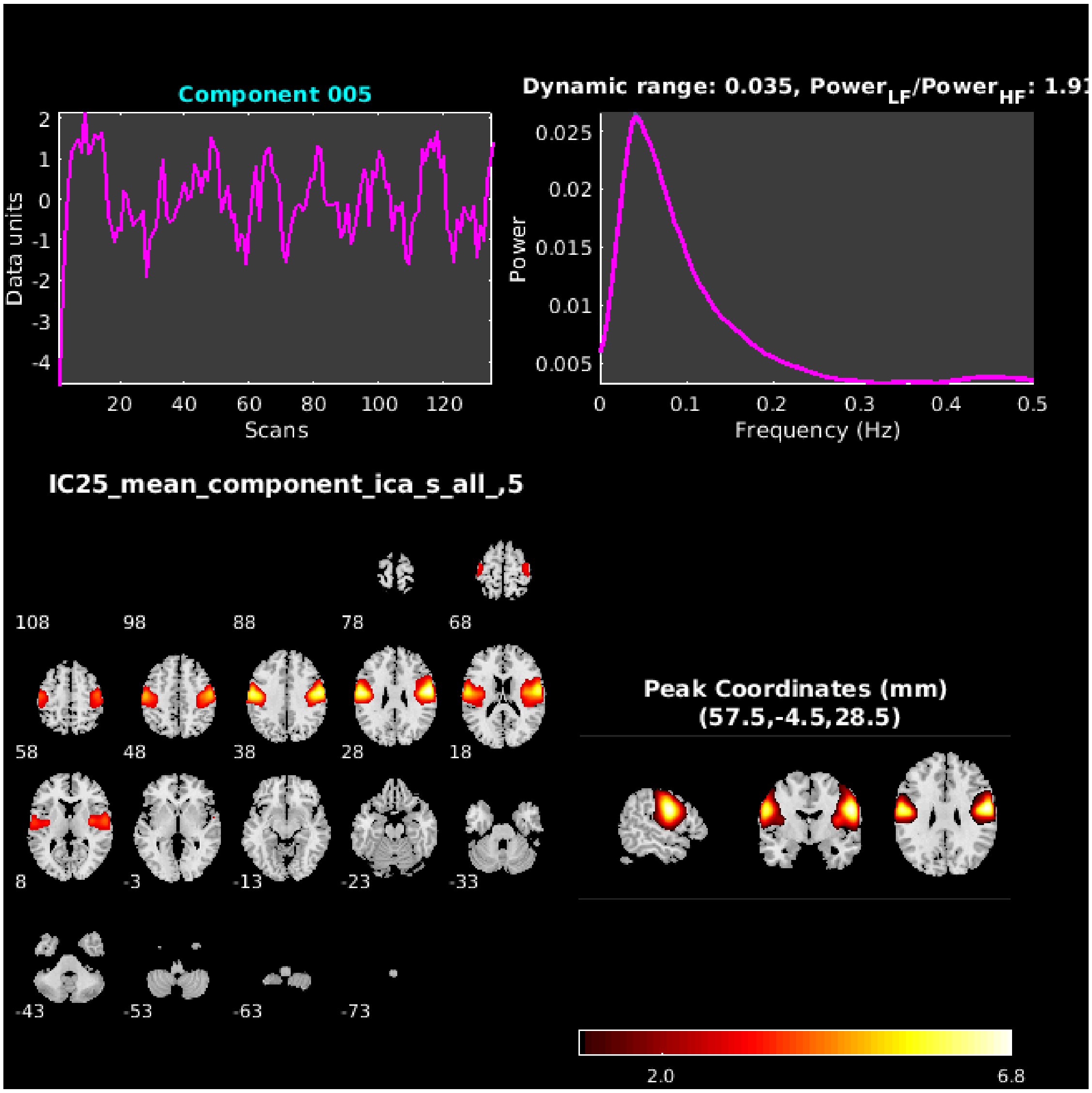
Component 005
Dynamic range: 0.035, Power_LF/Power_HF: 1.91
Data units
Scans
Power
Frequency (Hz)
IC25_mean_component_ica_s_all_,5
Peak Coordinates (mm)
(57.5,-4.5,28.5)
2.0
6.8

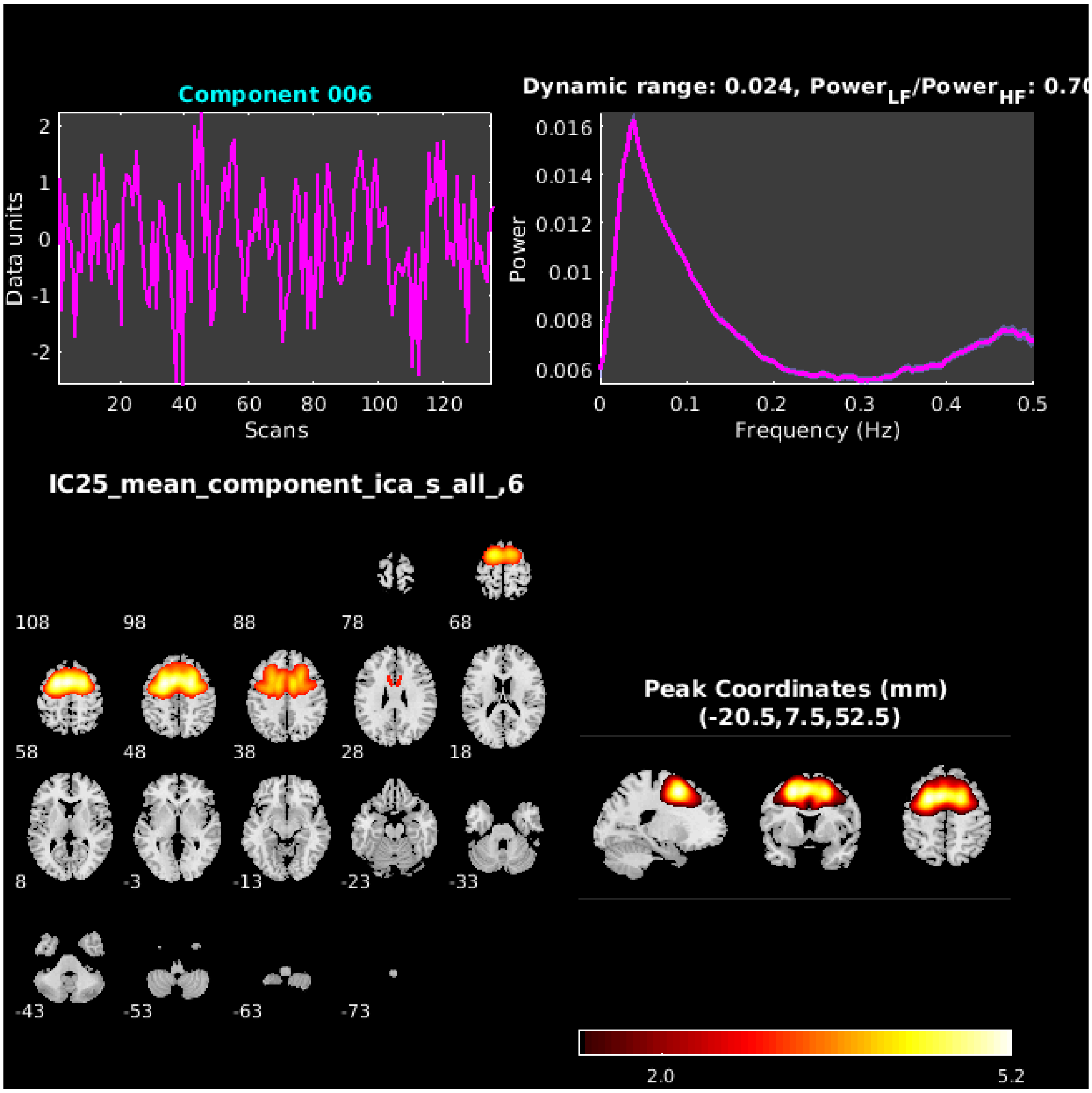
Component 006
Dynamic range: 0.024, Power_LF/Power_HF: 0.7
Data units
Scans
Power
Frequency (Hz)
IC25_mean_component_ica_s_all_,6
Peak Coordinates (mm)
(-20.5,7.5,52.5)
2.0
5.2

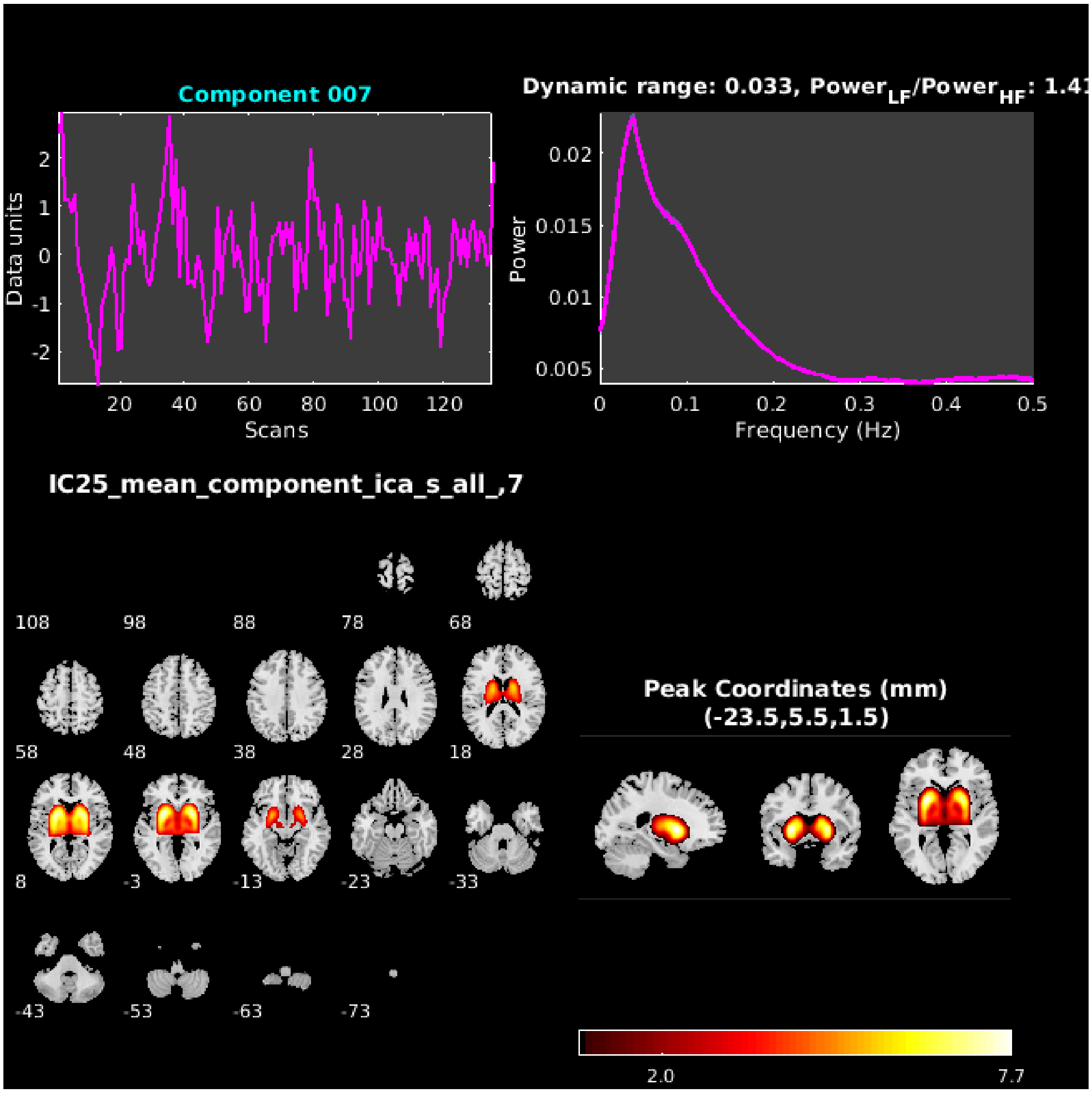
Component 007
Dynamic range: 0.033, Power$_{LF}$/Power$_{HF}$: 1.41
Data units
Scans
Power
Frequency (Hz)
IC25_mean_component_ica_s_all_,7
Peak Coordinates (mm)
(-23.5,5.5,1.5)
2.0
7.7

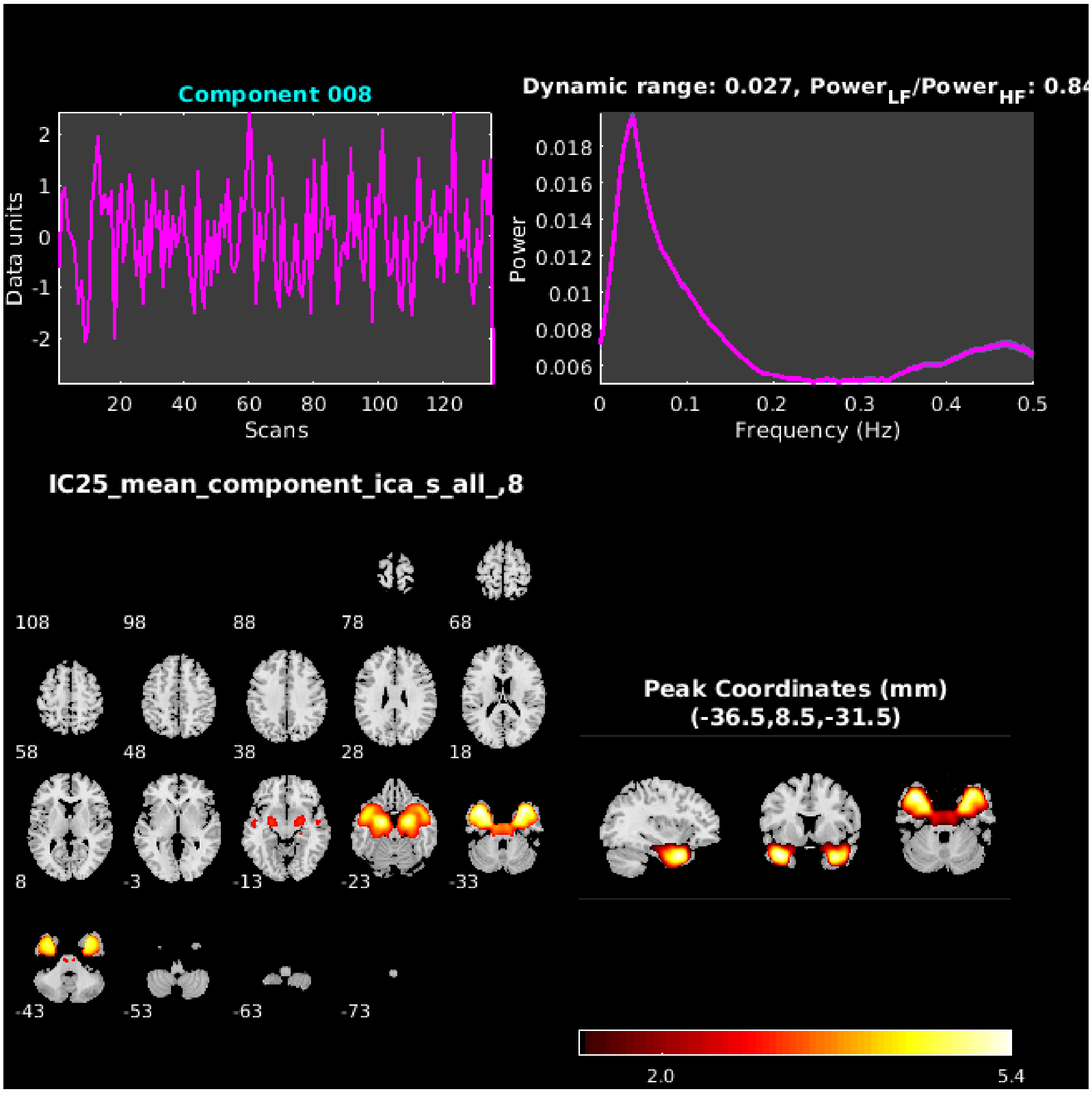

Component 008
Dynamic range: 0.027, Power_LF/Power_HF: 0.84
Data units
Scans
Power
Frequency (Hz)
IC25_mean_component_ica_s_all_,8
Peak Coordinates (mm)
(-36.5,8.5,-31.5)
2.0
5.4

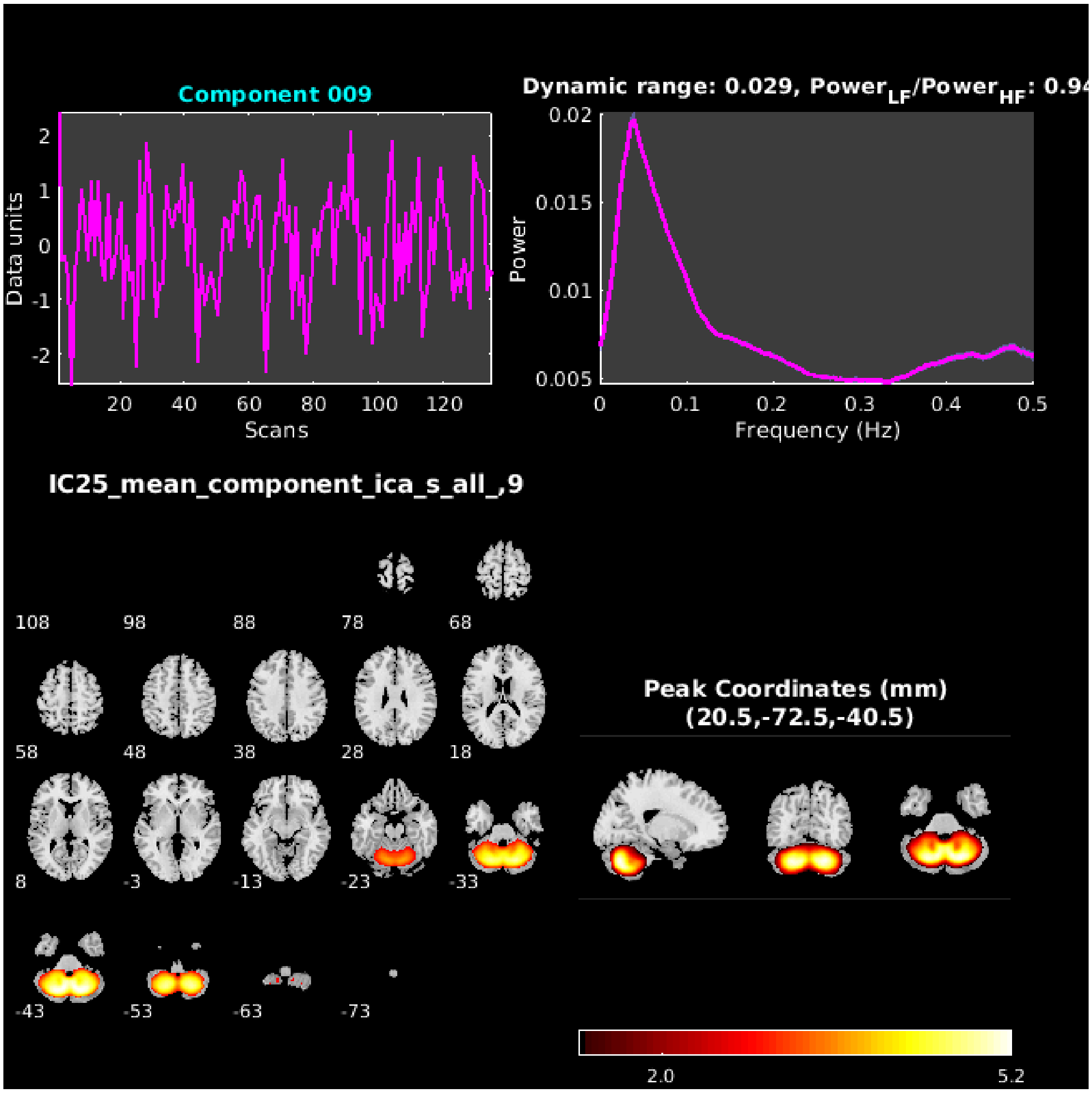

Component 009
Dynamic range: 0.029, Power$_{LF}$/Power$_{HF}$: 0.94
Data units
Scans
Power
Frequency (Hz)
IC25_mean_component_ica_s_all_,9
108
98
88
78
68
58
48
38
28
18
8
-3
-13
-23
-33
-43
-53
-63
-73
Peak Coordinates (mm)
(20.5,-72.5,-40.5)
2.0
5.2

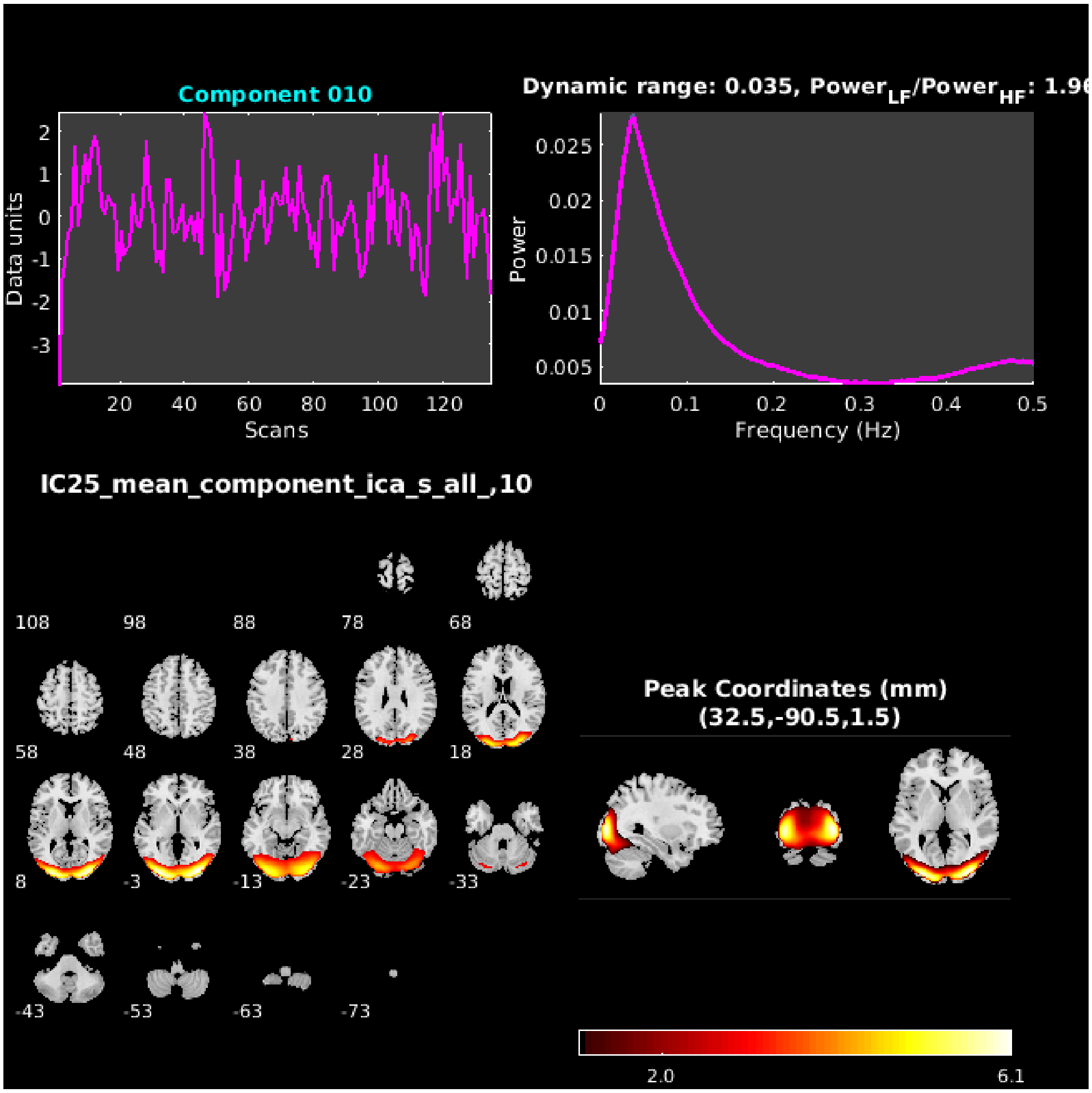
Component 010
Dynamic range: 0.035, Power$_{LF}$/Power$_{HF}$: 1.96
Data units
Scans
Power
Frequency (Hz)
IC25_mean_component_ica_s_all_,10
Peak Coordinates (mm)
(32.5,-90.5,1.5)
2.0
6.1

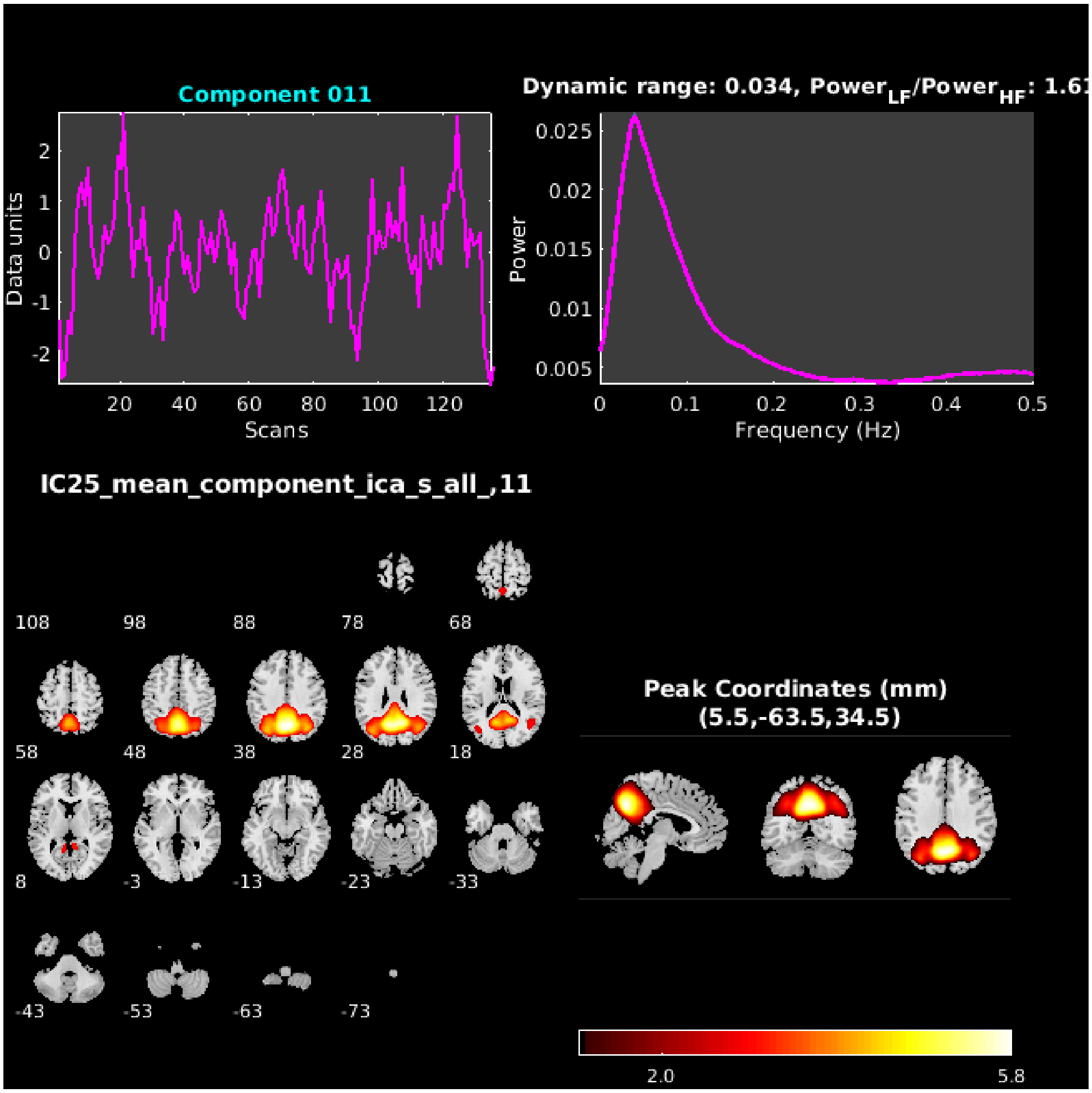
Component 011
Dynamic range: 0.034, Power$_{LF}$/Power$_{HF}$: 1.61
Data units
Scans
Power
Frequency (Hz)
IC25_mean_component_ica_s_all_,11
Peak Coordinates (mm)
(5.5,-63.5,34.5)
2.0
5.8

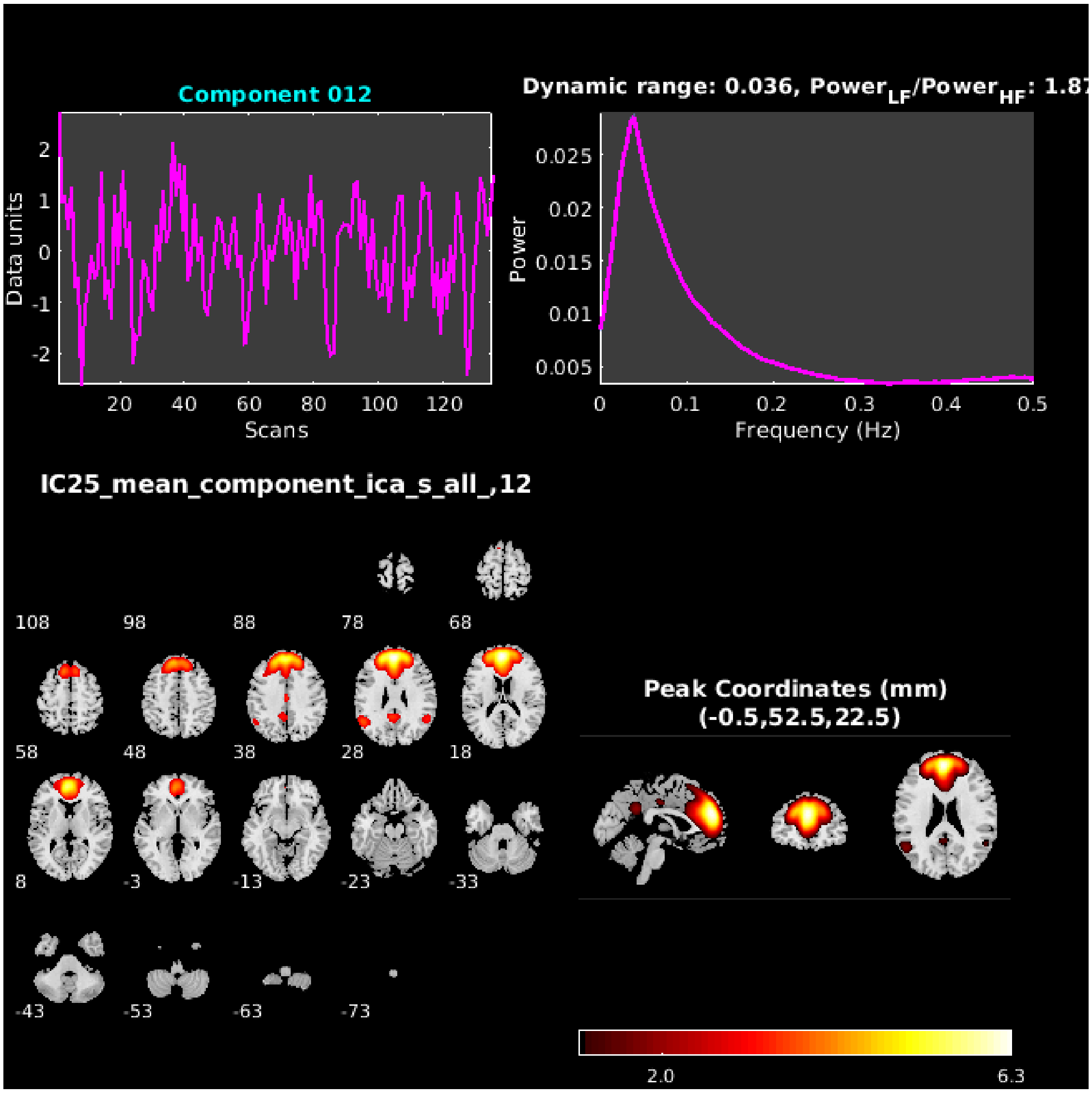
Component 012
Dynamic range: 0.036, Power$_{LF}$/Power$_{HF}$: 1.87
Data units
Scans
Power
Frequency (Hz)
IC25_mean_component_ica_s_all_,12
Peak Coordinates (mm)
(-0.5,52.5,22.5)
2.0
6.3

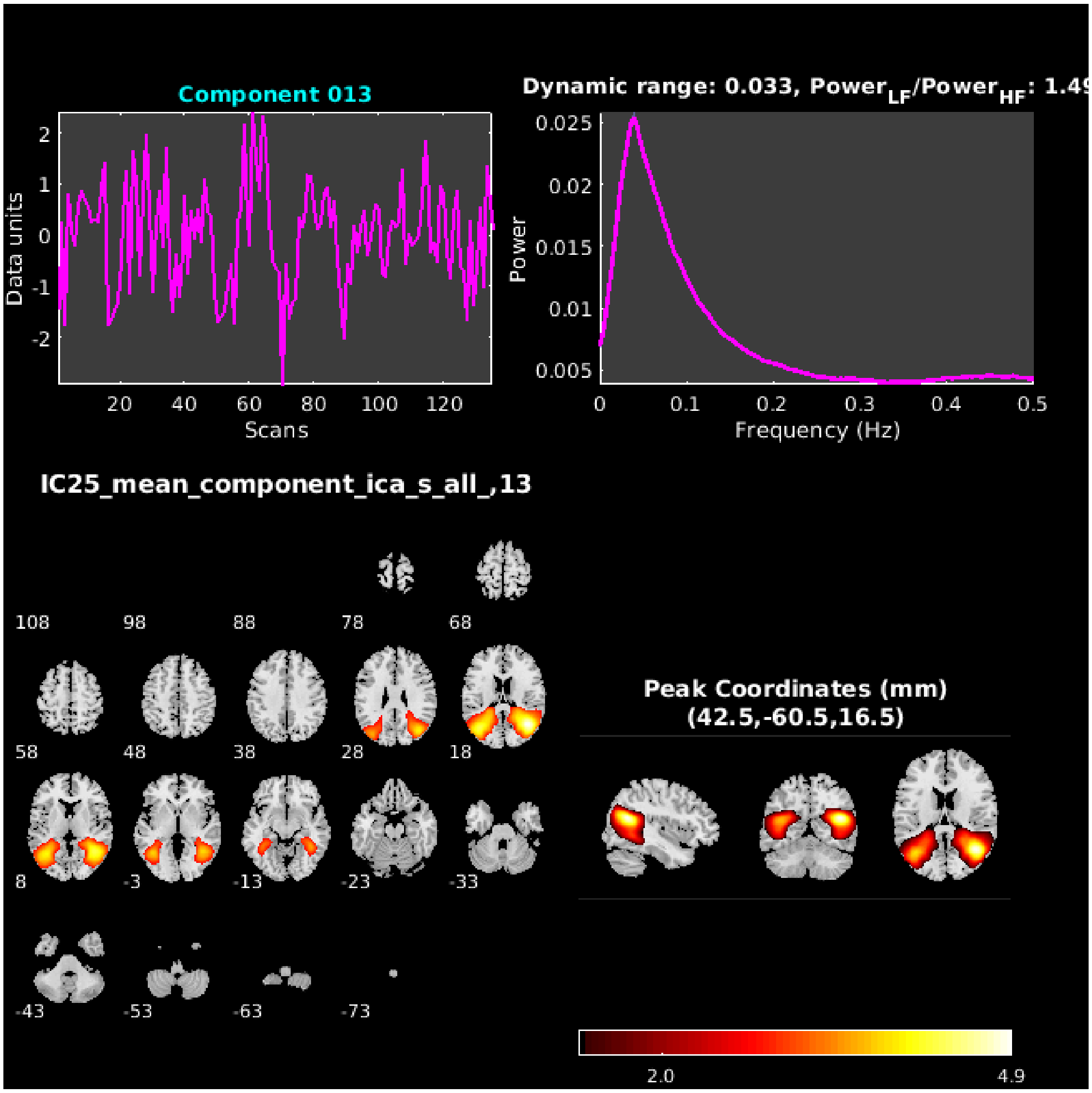
Component 013
Dynamic range: 0.033, Power_LF/Power_HF: 1.49
Data units
Scans
Power
Frequency (Hz)
IC25_mean_component_ica_s_all_,13
Peak Coordinates (mm)
(42.5,-60.5,16.5)
2.0
4.9

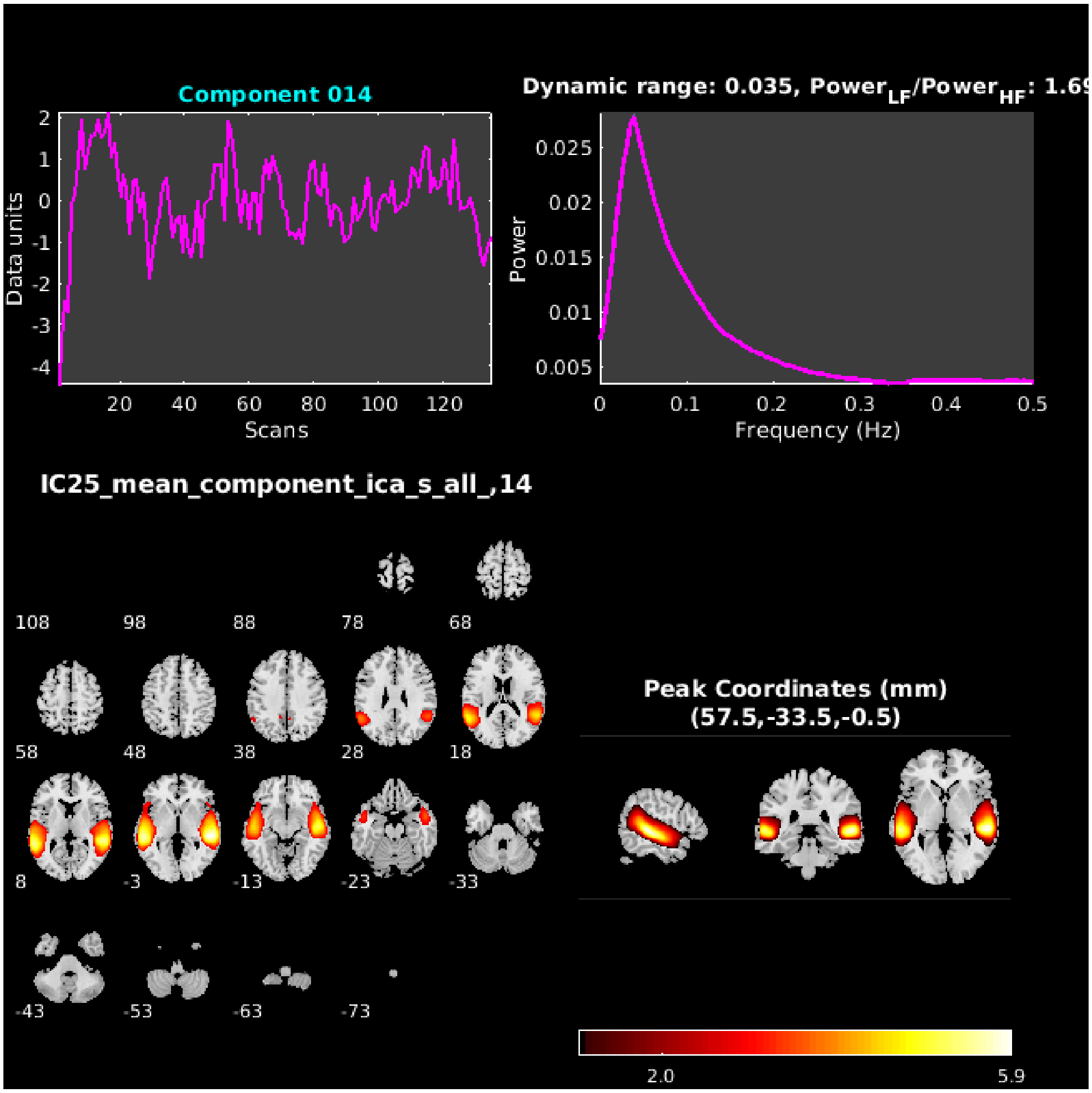

Component 014
Data units
Scans
Dynamic range: 0.035, Power$_{LF}$/Power$_{HF}$: 1.69
Power
Frequency (Hz)
IC25_mean_component_ica_s_all_,14
Peak Coordinates (mm)
(57.5,-33.5,-0.5)
2.0
5.9

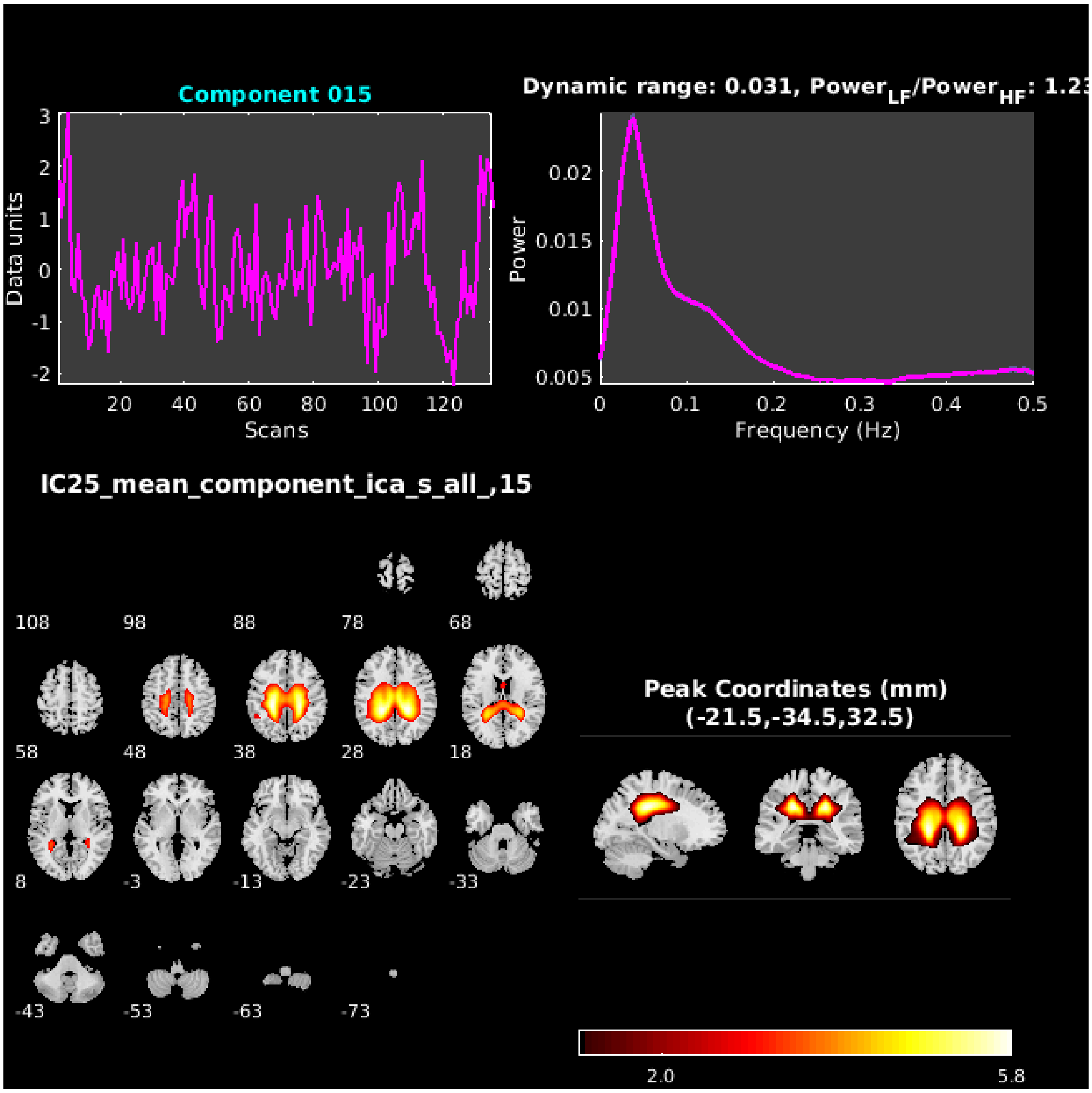
Component 015
Data units
Scans
Dynamic range: 0.031, Power_LF/Power_HF: 1.23
Power
Frequency (Hz)
IC25_mean_component_ica_s_all_,15
Peak Coordinates (mm)
(-21.5,-34.5,32.5)

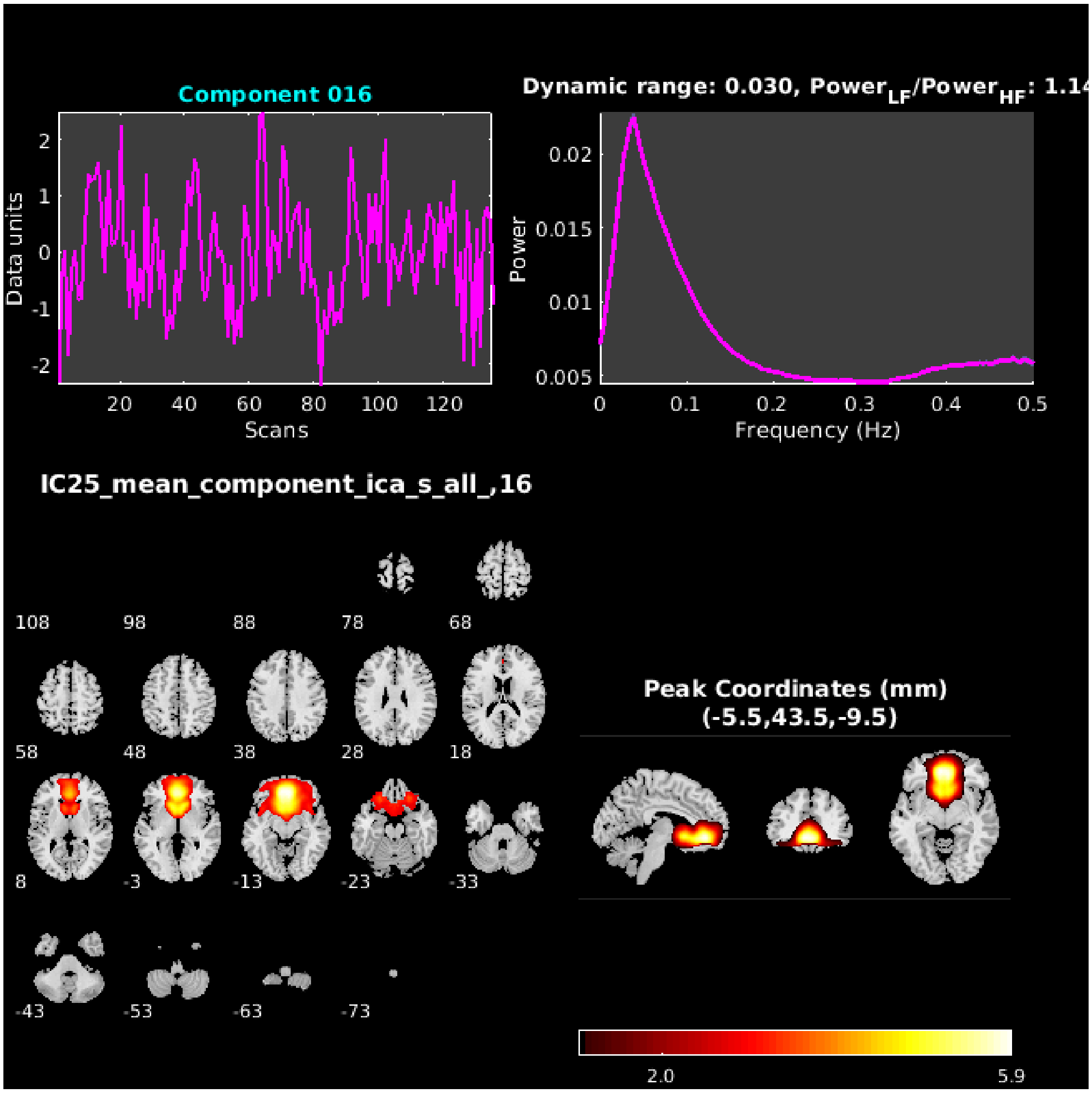

Component 016
Dynamic range: 0.030, Power_LF/Power_HF: 1.14
Data units
Scans
Power
Frequency (Hz)
IC25_mean_component_ica_s_all_,16
Peak Coordinates (mm)
(-5.5,43.5,-9.5)
2.0
5.9

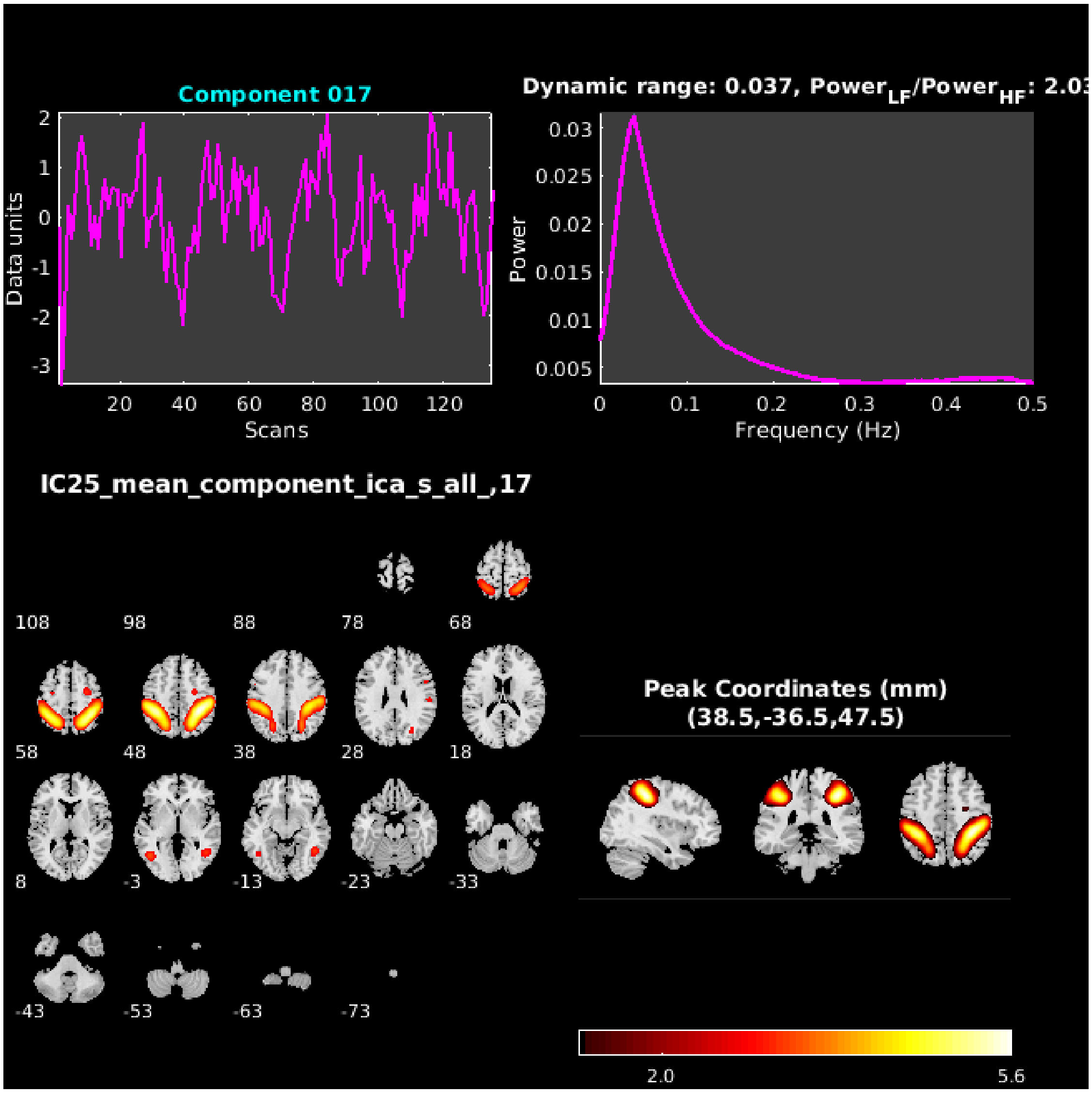
Component 017
Dynamic range: 0.037, Power_LF/Power_HF: 2.0
Data units
Scans
Power
Frequency (Hz)
IC25_mean_component_ica_s_all_,17
108
98
88
78
68
58
48
38
28
18
8
-3
-13
-23
-33
-43
-53
-63
-73
Peak Coordinates (mm)
(38.5,-36.5,47.5)
2.0
5.6

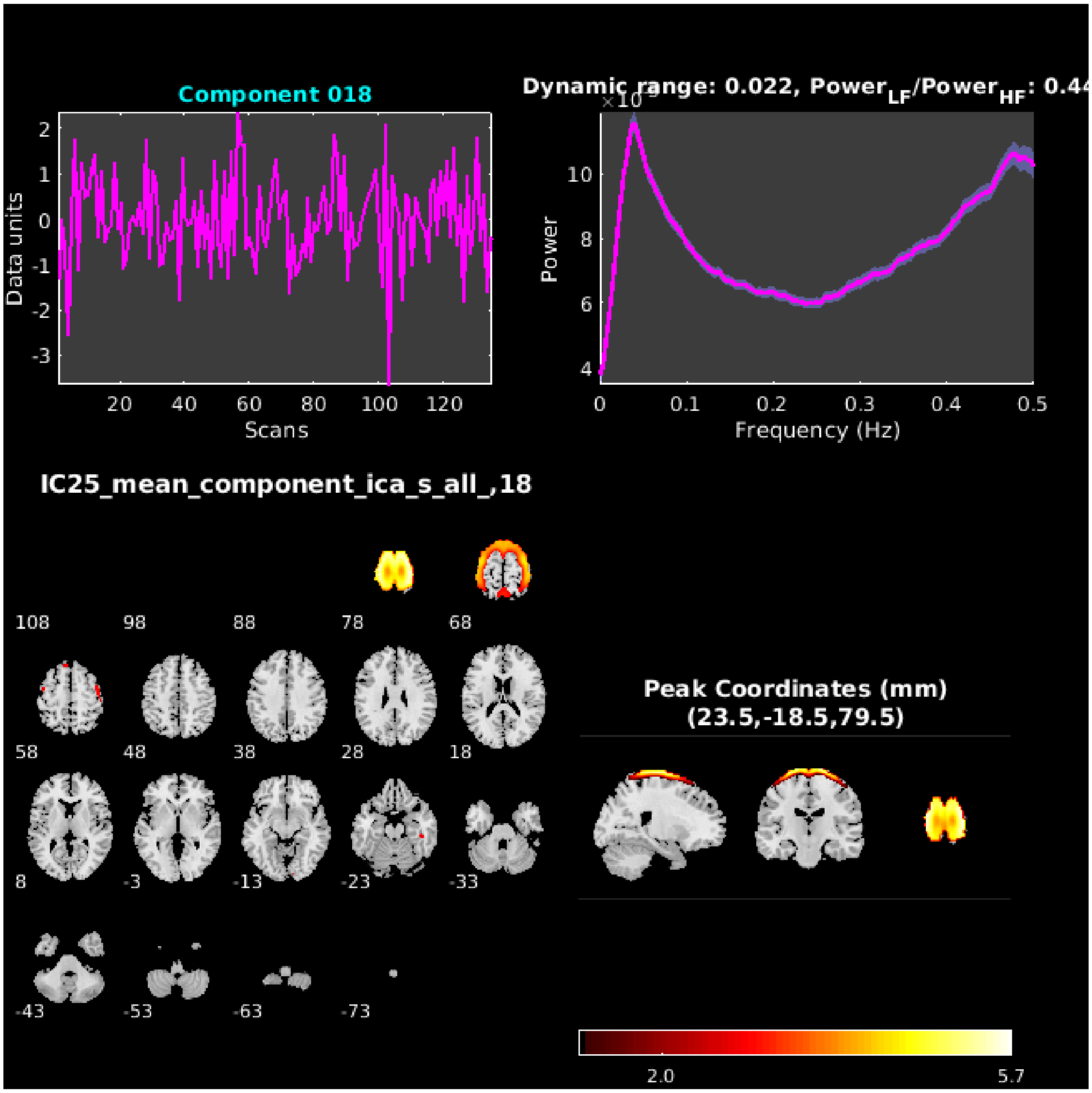
Component 018
Dynamic range: 0.022, Power$_{LF}$/Power$_{HF}$: 0.44
Data units
Scans
Power
Frequency (Hz)
IC25_mean_component_ica_s_all_,18
Peak Coordinates (mm)
(23.5,-18.5,79.5)
2.0
5.7

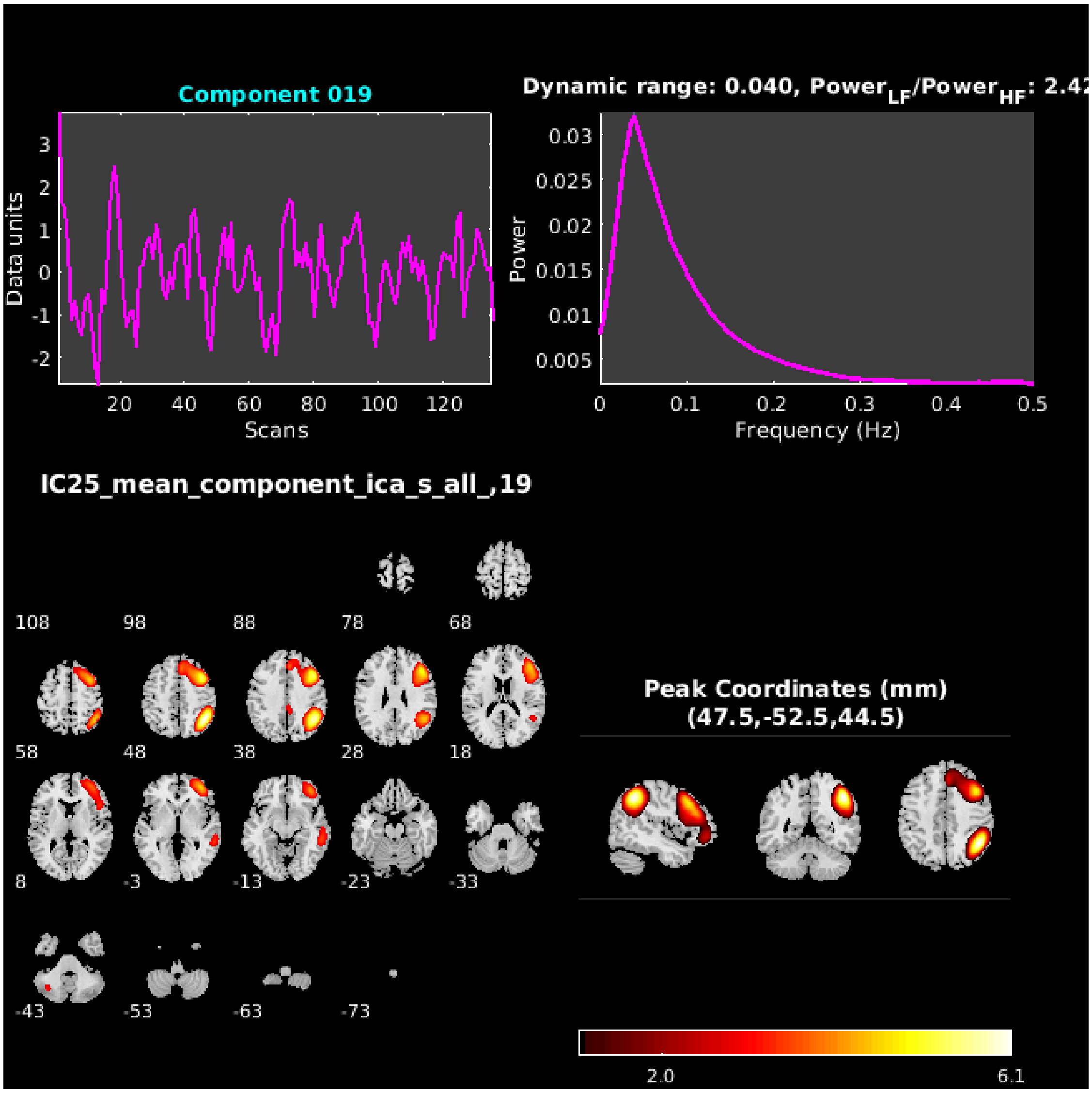

Component 019
Dynamic range: 0.040, Power$_{LF}$/Power$_{HF}$: 2.42
Data units
Scans
Power
Frequency (Hz)
IC25_mean_component_ica_s_all_,19
108
98
88
78
68
58
48
38
28
18
8
-3
-13
-23
-33
-43
-53
-63
-73
Peak Coordinates (mm)
(47.5,-52.5,44.5)
2.0
6.1

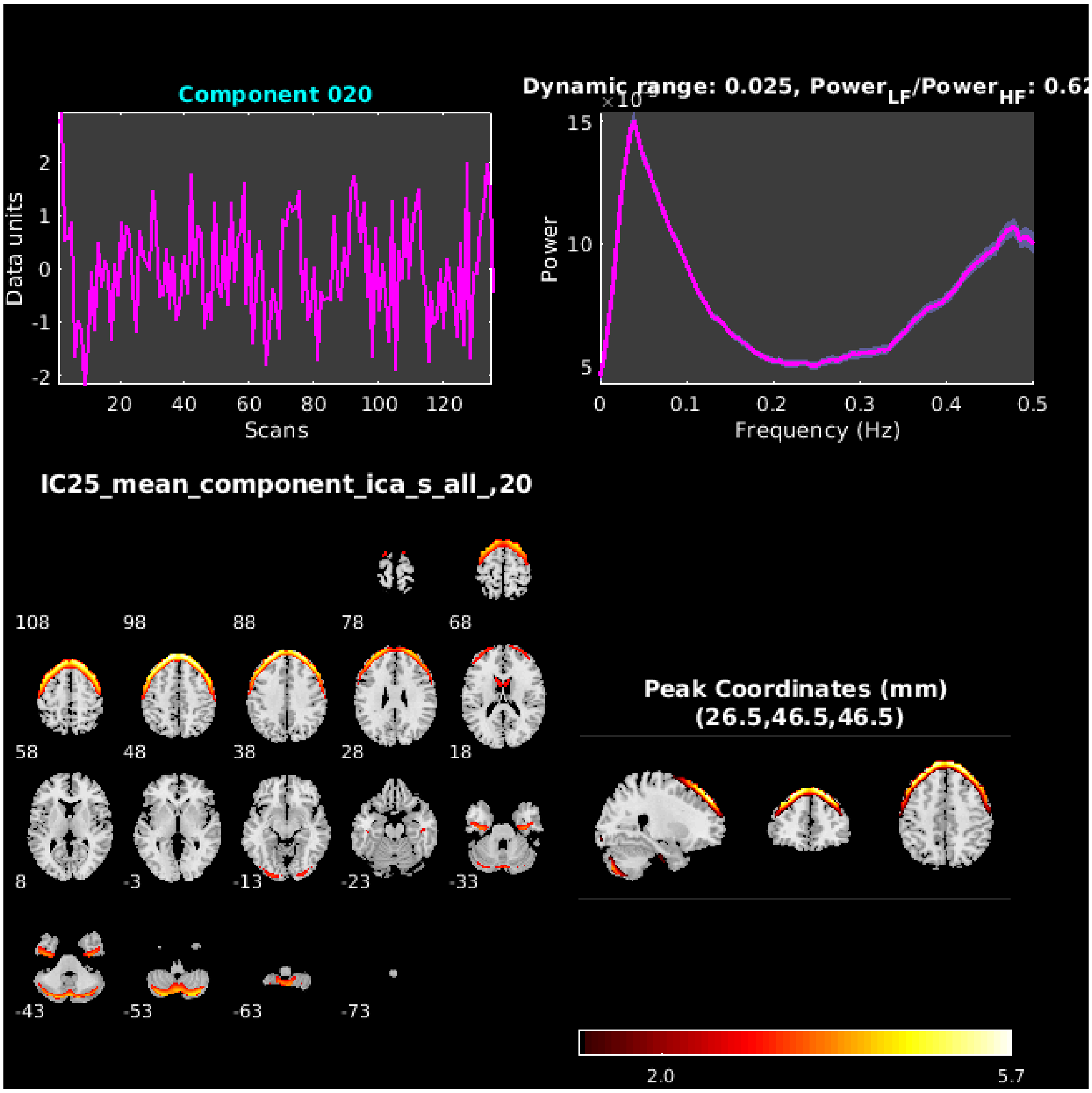
Component 020
Dynamic range: 0.025, Power_LF/Power_HF: 0.62
Data units
Scans
Power
Frequency (Hz)
IC25_mean_component_ica_s_all_,20
Peak Coordinates (mm)
(26.5,46.5,46.5)
2.0
5.7

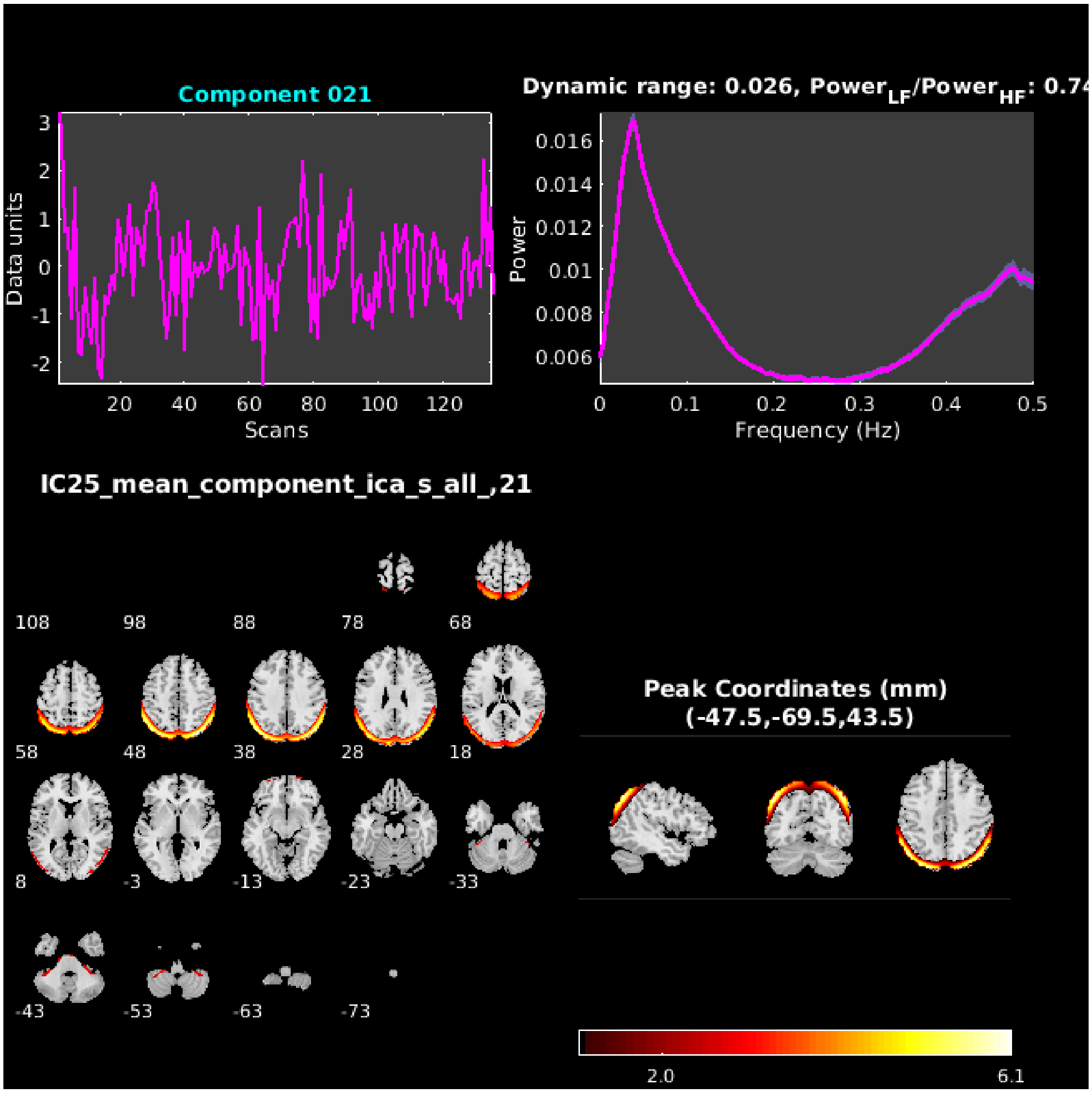
Component 021
Dynamic range: 0.026, Power_LF/Power_HF: 0.74
Data units
Scans
Power
Frequency (Hz)
IC25_mean_component_ica_s_all_,21
108
98
88
78
68
58
48
38
28
18
8
-3
-13
-23
-33
-43
-53
-63
-73
Peak Coordinates (mm)
(-47.5,-69.5,43.5)
2.0
6.1

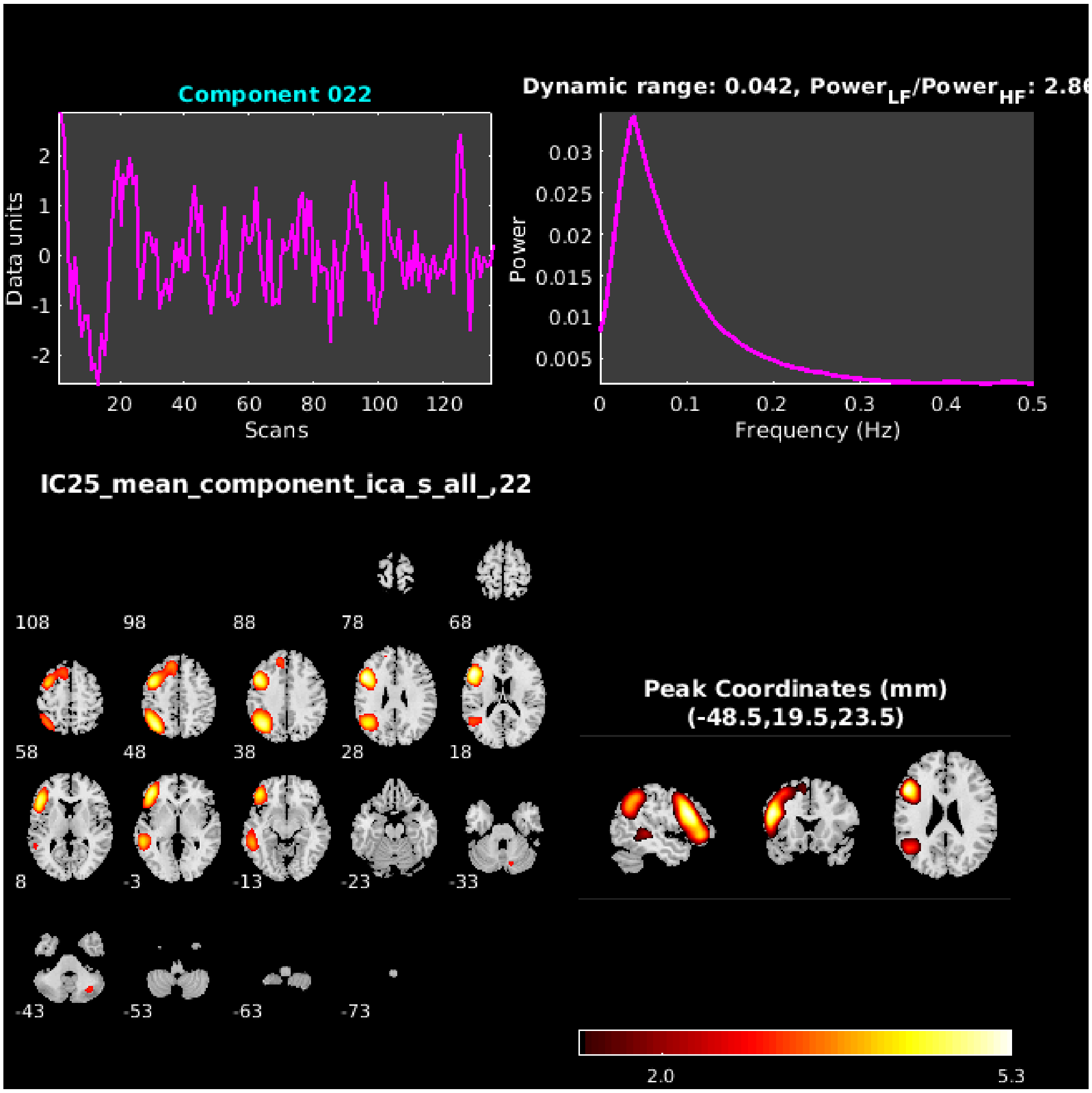
Component 022
Dynamic range: 0.042, Power$_{LF}$/Power$_{HF}$: 2.86
Data units
Scans
Power
Frequency (Hz)
IC25_mean_component_ica_s_all_,22
Peak Coordinates (mm)
(-48.5,19.5,23.5)
2.0
5.3

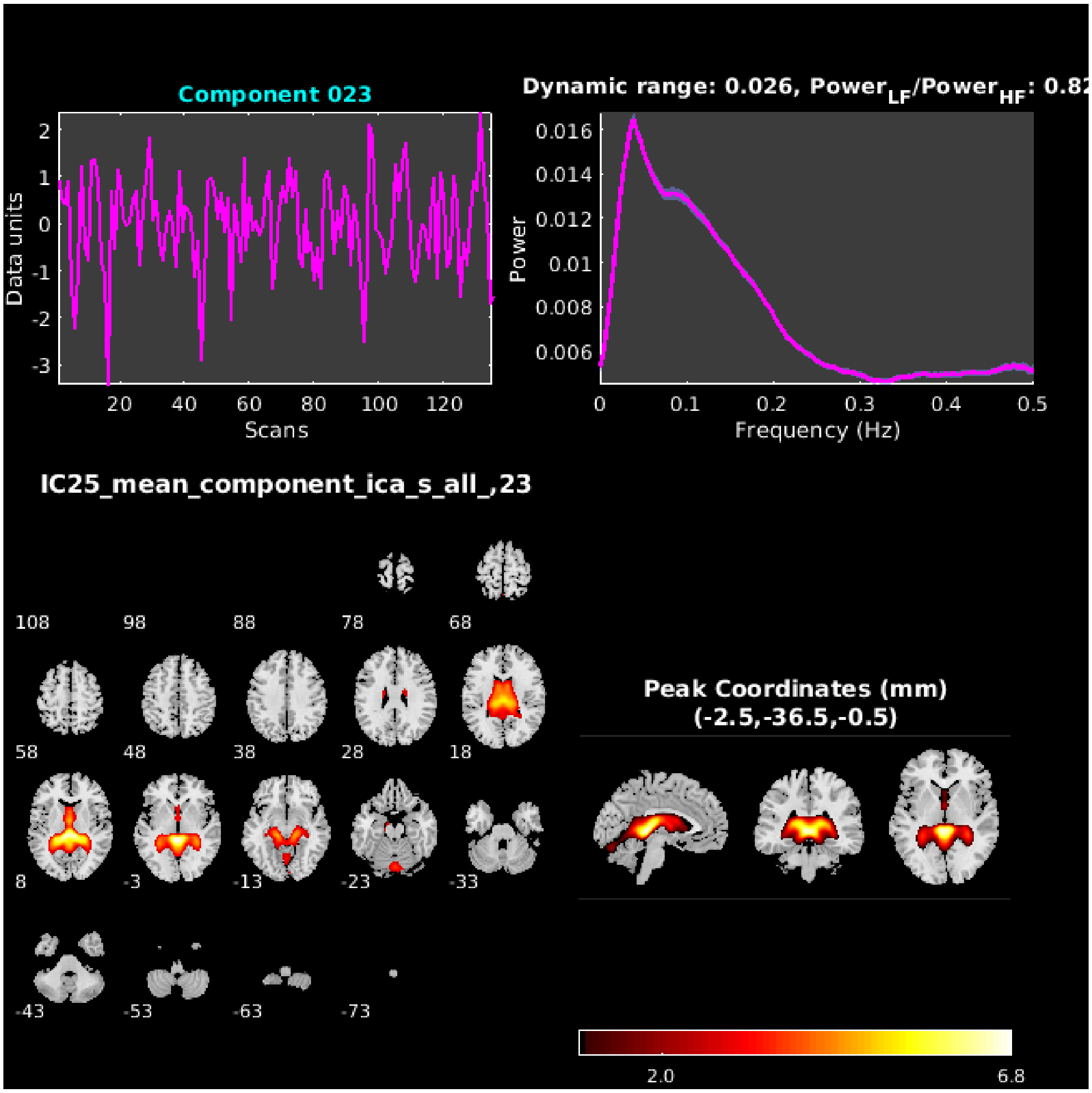
Component 023
Dynamic range: 0.026, Power$_{LF}$/Power$_{HF}$: 0.8
Data units
Scans
Power
Frequency (Hz)
IC25_mean_component_ica_s_all_,23
Peak Coordinates (mm)
(-2.5,-36.5,-0.5)
2.0
6.8

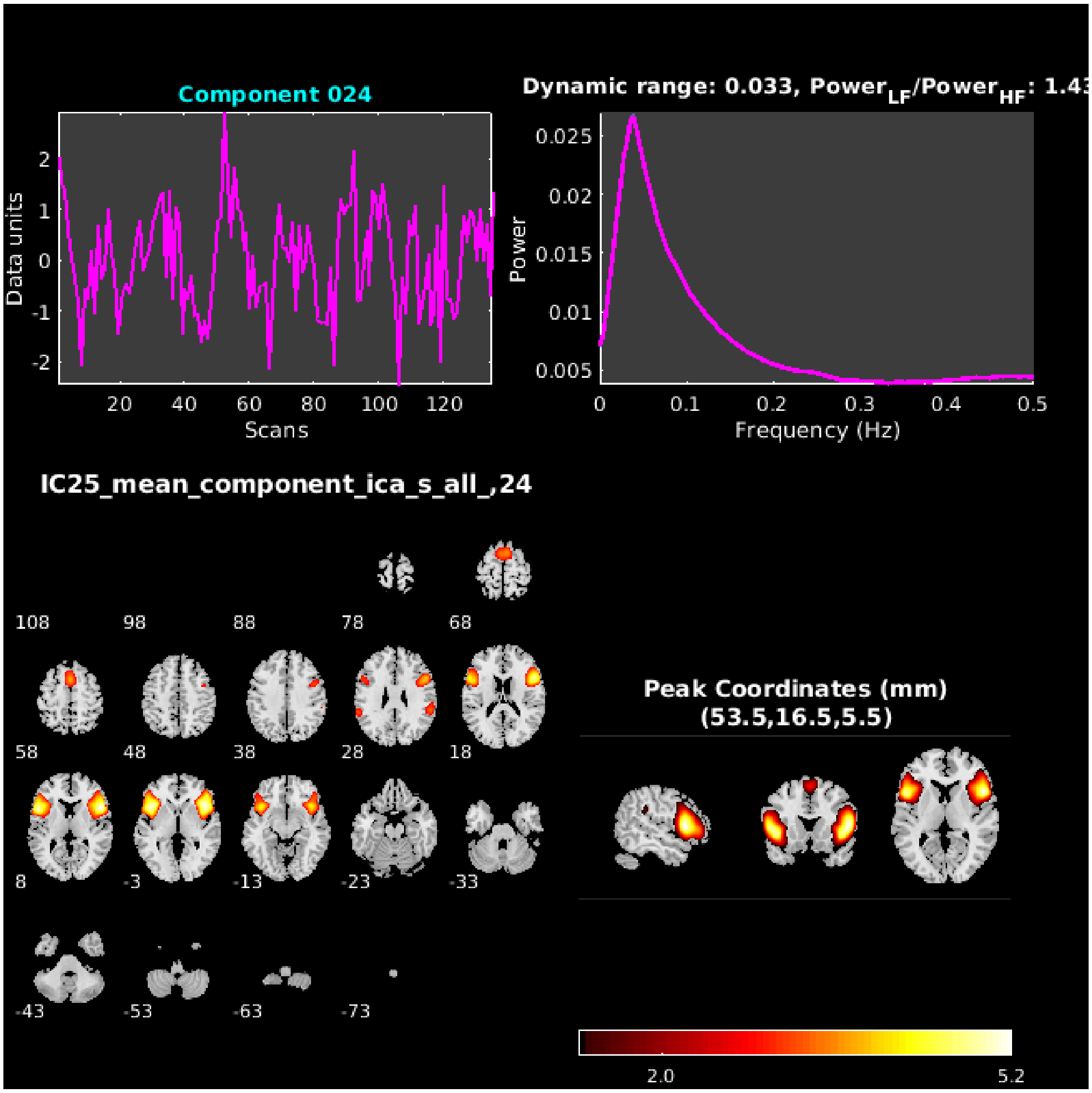

Component 024
Dynamic range: 0.033, Power_LF/Power_HF: 1.43
Data units
Scans
Power
Frequency (Hz)
IC25_mean_component_ica_s_all_,24
108 98 88 78 68
58 48 38 28 18
8 -3 -13 -23 -33
-43 -53 -63 -73
Peak Coordinates (mm)
(53.5,16.5,5.5)
2.0
5.2

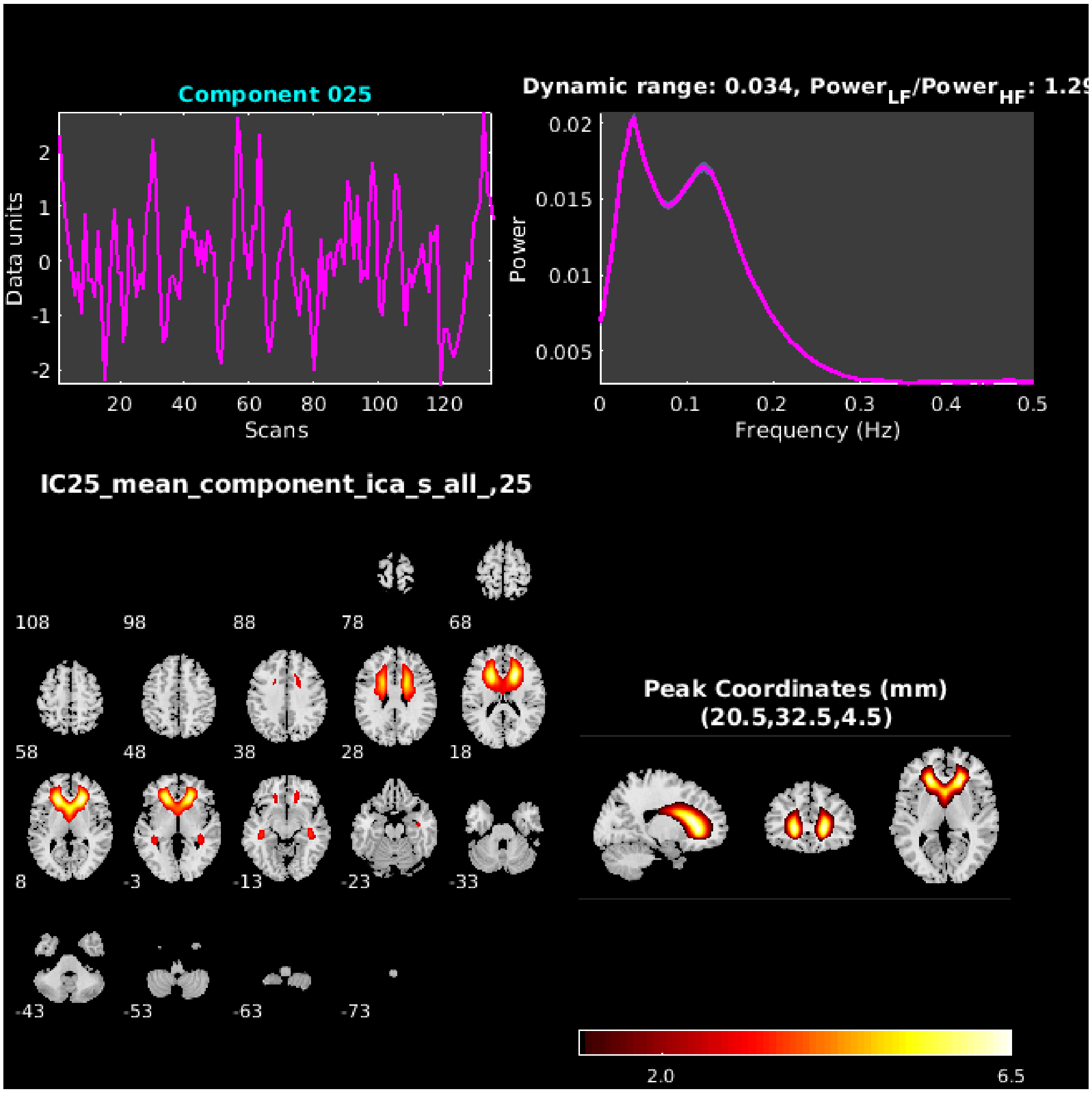

Spectral Summary

- **a) dynamic_range** - Difference between the peak power and minimum power at frequencies to the right of the peak.
- **b) fALFF** - Low frequency to high frequency power ratio.

ComponentNumber	*DynamicRange*	*fALFF*
1	*0.025514*	*0.72891*
2	*0.039029*	*2.8383*
3	*0.024283*	*0.64486*
4	*0.030932*	*1.3423*
5	*0.034979*	*1.9136*
6	*0.024316*	*0.70002*
7	*0.032998*	*1.4156*

8	0.027307	0.84383
9	0.0287	0.94729
10	0.03459	1.9603
11	0.033881	1.613
12	0.035558	1.8742
13	0.032597	1.4945
14	0.035265	1.6987
15	0.030872	1.2306
16	0.029836	1.1443
17	0.037322	2.0316
18	0.021752	0.43973
19	0.039518	2.4209
20	0.024738	0.62654
21	0.025813	0.74946
22	0.041776	2.8693
23	0.02637	0.82335
24	0.032633	1.4301
25	0.033781	1.2906

Temporal Stats On Beta Weights

Multiple regression is done using the timecourses from SPM design matrix as model and ICA timecourses as observations. R^2 values for each component are shown in bar plot. For each component, one sample t-test results of each session and condition are shown in the bar plots.

Kurtosis of timecourses and spatial maps

Mean across subjects is reported in table. Figure shows mean+/- SEM across subjects

ComponentNumber	Timecourses	SpatialMaps
1	3.8204	8.5945
2	4.199	5.0568
3	4.3593	4.9744
4	4.4795	4.7743
5	4.0501	4.2753
6	5.7256	3.6061
7	4.0951	4.7447
8	4.6412	3.937
9	4.9515	4.3788
10	4.4294	4.2854
11	4.1925	4.0585
12	5.1892	4.0065
13	4.4609	3.2826
14	3.8768	3.8795
15	3.9233	3.9626
16	4.5505	3.8993
17	4.1976	3.6842
18	6.6633	3.9427
19	3.6244	3.9821

20	*5.5499*	*4.1725*
21	*4.6842*	*4.0848*
22	*3.5753*	*3.9669*
23	*4.2378*	*4.0705*
24	*4.2534*	*3.4669*
25	*4.3722*	*3.9117*

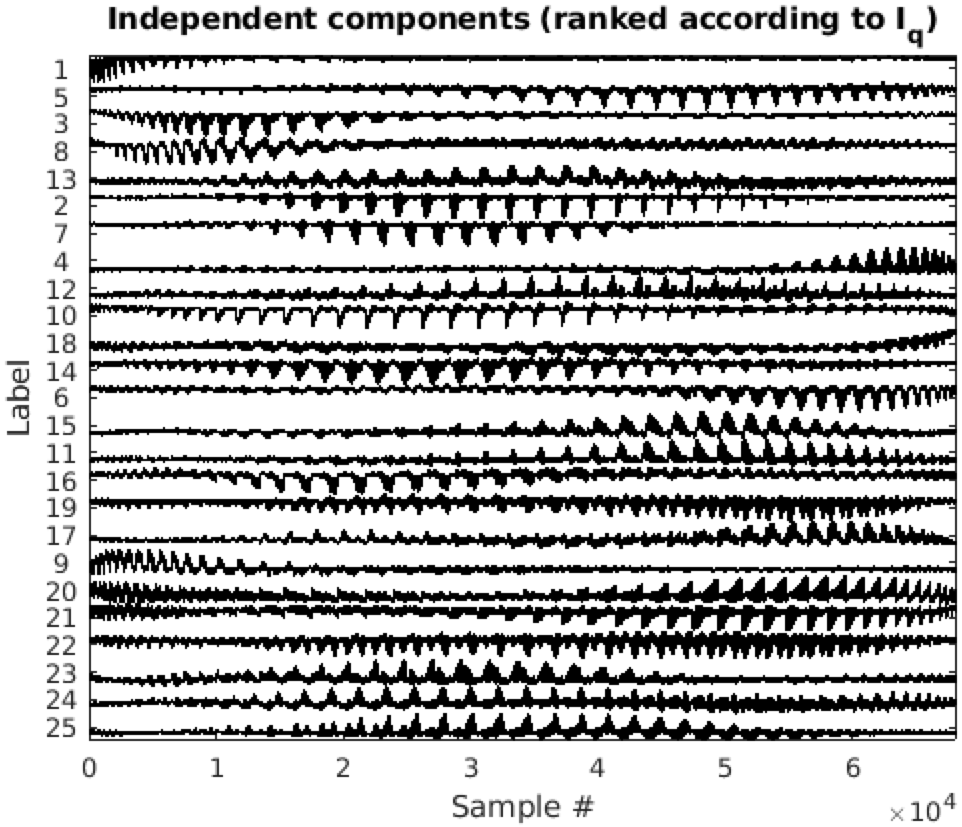

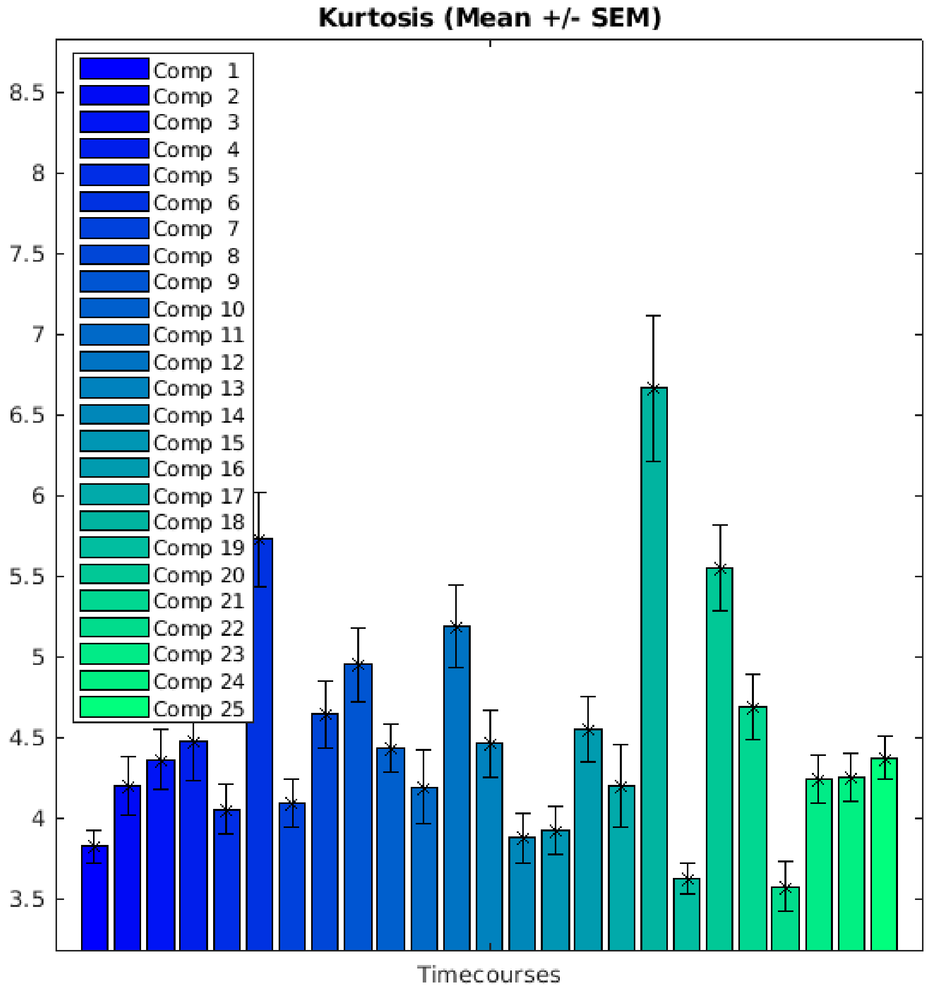
Kurtosis (Mean +/- SEM)
8.5
8
7.5
7
6.5
6
5.5
5
4.5
4
3.5
Comp 1
Comp 2
Comp 3
Comp 4
Comp 5
Comp 6
Comp 7
Comp 8
Comp 9
Comp 10
Comp 11
Comp 12
Comp 13
Comp 14
Comp 15
Comp 16
Comp 17
Comp 18
Comp 19
Comp 20
Comp 21
Comp 22
Comp 23
Comp 24
Comp 25
Timecourses

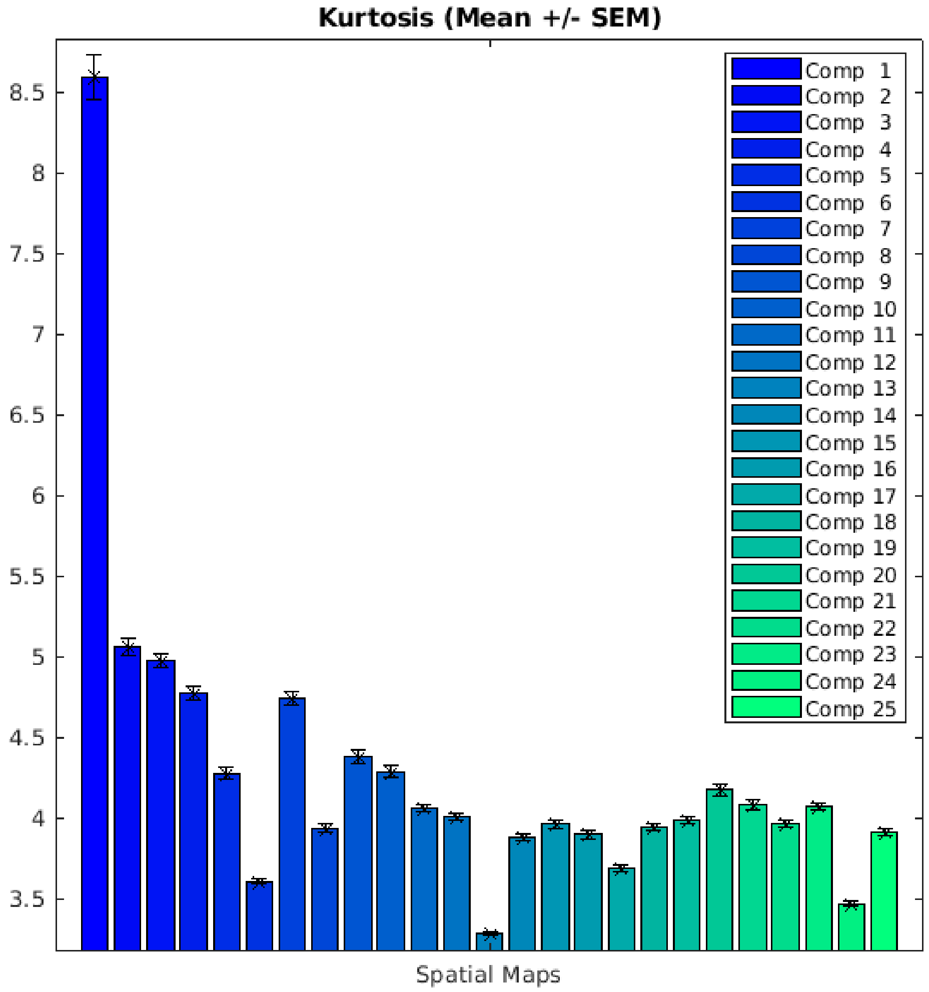

FNC correlations

Functional network connectivity correlations are computed for each data-set and averaged across sessions.

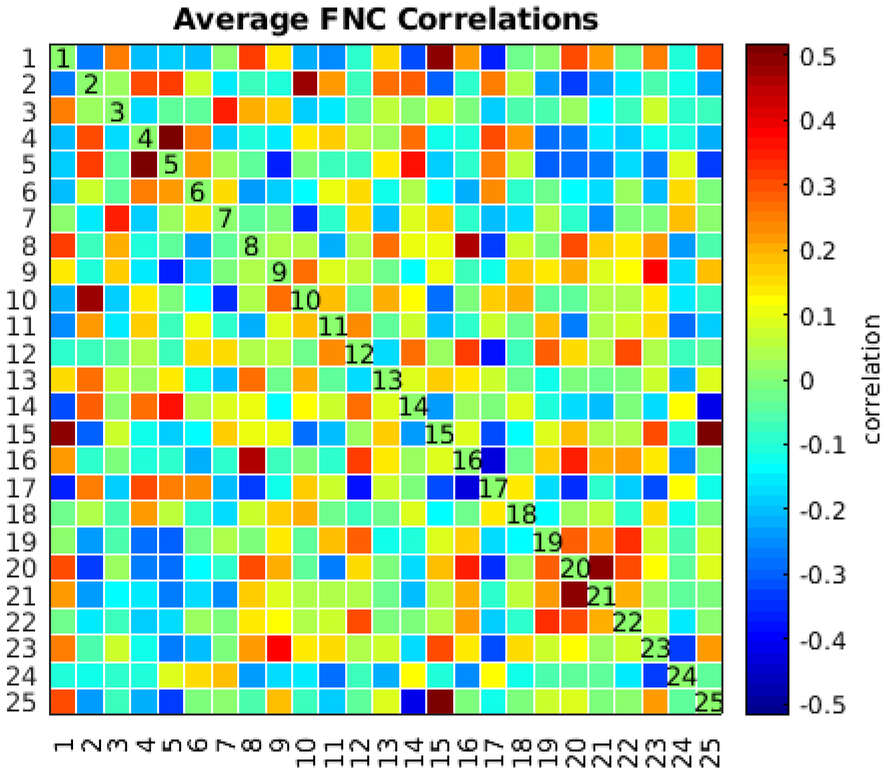

FNC metrics of component spatial maps

Mutual information is computed between components spatially and averaged across data-sets.

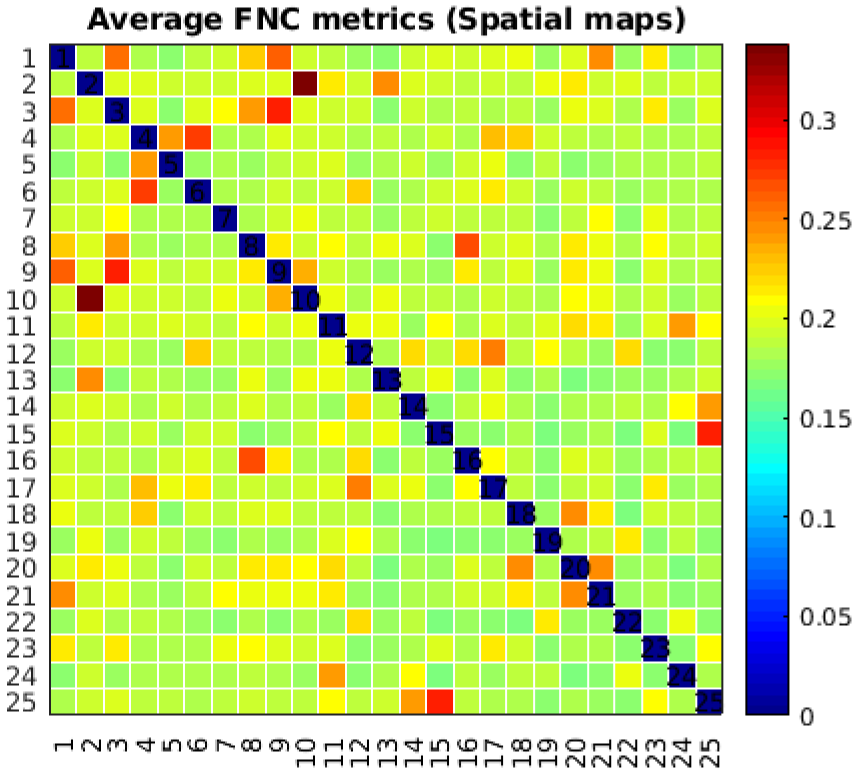

Published with MATLAB® R2019a
