# Supplementary material for: Multi-spatial-scale dynamic interactions between functional sources reveal sex-specific changes in schizophrenia
Source: Netw Neurosci. 2022 Jun 1;6(2):357–81. doi: 10.1162/netn_a_00196 (PMC9208002; doi:10.1162/netn_a_00196)
Supplement: Supplementary file 1 [file netn-06-357-s001.zip › icatb_gica_html_report50.pdf]

Table of Contents

.... 1
Group ICA Parameters 1
ICASSO Plots 2
Mean Components 5
Spectral Summary 54
Temporal Stats On Beta Weights 55
Kurtosis of timecourses and spatial maps 56
FNC correlations 58
FNC metrics of component spatial maps 59

Group ICA Parameters

...

Number of Subjects : 856

Number of Sessions : 1

Number of Independent Components : 50

ICA Algorithm : Infomax

Number Of Scans/Timepoints : 157

Mask File : mask_common

Data Pre-processing Type : Variance Normalization

PCA Type : Standard

Group PCA Type : Subject Specific

Group ICA Type : Spatial

Back Reconstruction Type : Spatial-temporal Regression

Scaling Components : Z-scores

Stability analysis type : ICASSO

Group analysis mode: Parallel

Anatomical file: /trdapps/linux-x86_64/matlab/toolboxes/GroupICATv4.0b/icatb/icatb_templates/ch2bet.nii
Slice Plane: Axial

```
Image values: Positive

Convert to Z-scores: yes

Threshold: 1.96


...................................................
```

ICASSO Plots

```
Warning: Creates overwhelming number of lines
Warning: Tries to change the limit...
Warning: New limit =0.98499
```

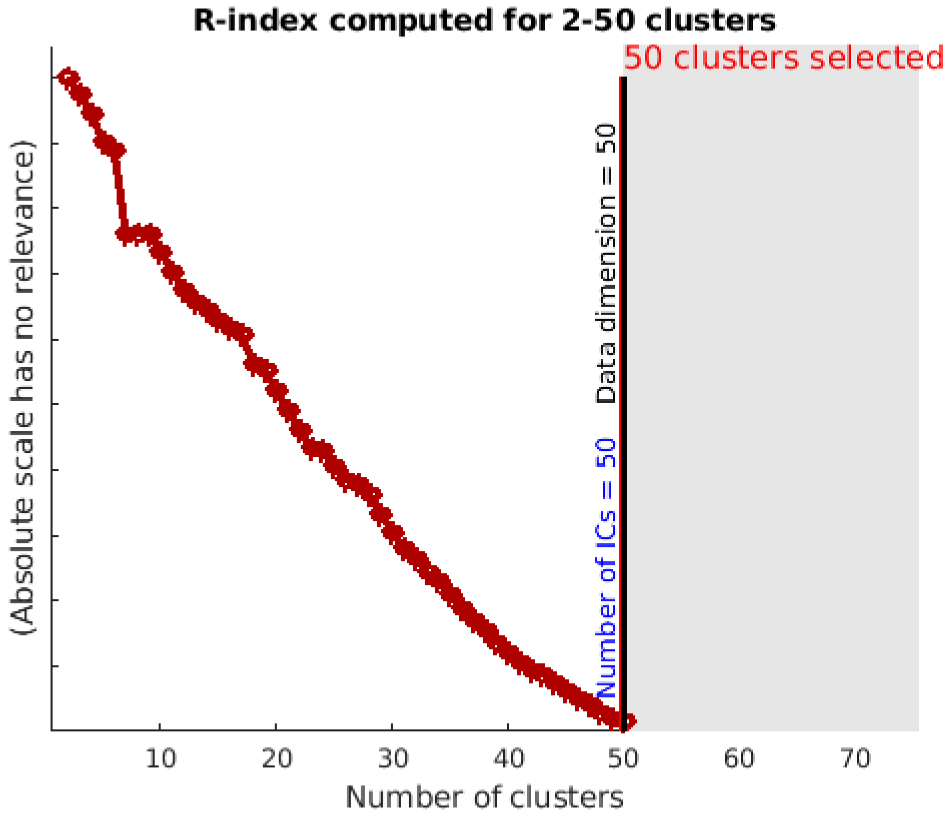

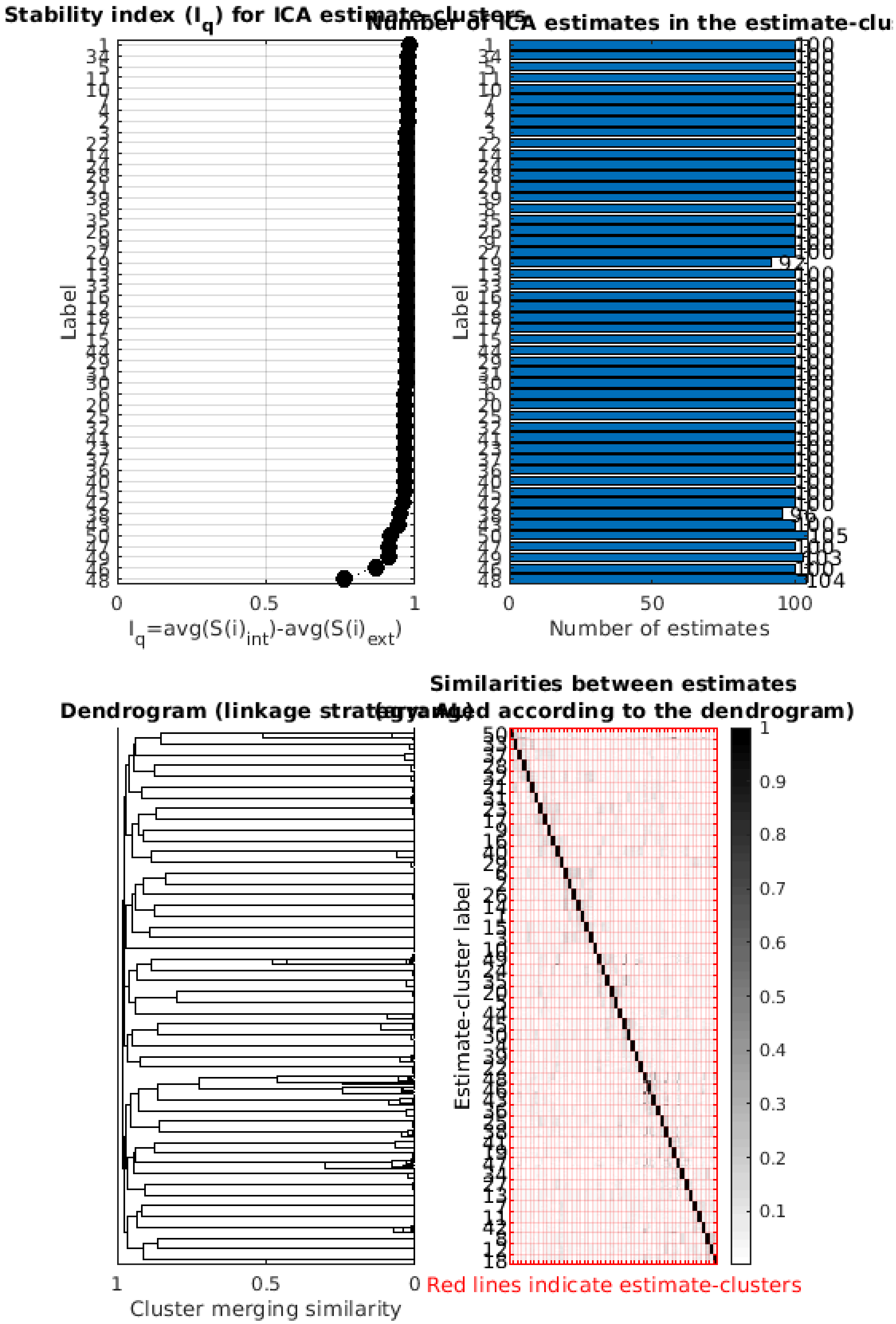
Stability index (I_q) for ICA estimate-clusters
Number of ICA estimates in the estimate-clus
Label
I_q=avg(S(i)_int)-avg(S(i)_ext)
Number of estimates
Dendrogram (linkage strategy: AL)
Cluster merging similarity
Similarities between estimates
(arranged according to the dendrogram)
Estimate-cluster label
Red lines indicate estimate-clusters

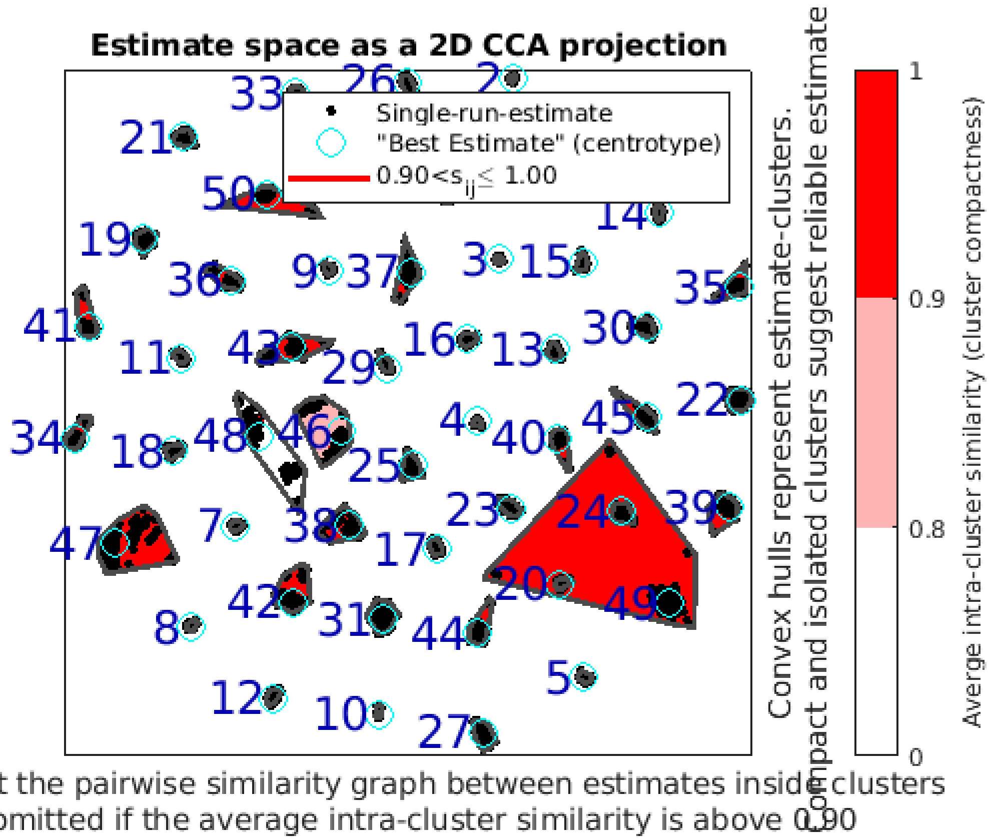
Estimate space as a 2D CCA projection
Single-run-estimate
"Best Estimate" (centrotype)
0.90<s$_{ij}$≤ 1.00
Convex hulls represent estimate-clusters.
Compact and isolated clusters suggest reliable estimate
Averge intra-cluster similarity (cluster compactness)
1
0.9
0.8
0
: that the pairwise similarity graph between estimates inside clusters
is omitted if the average intra-cluster similarity is above 0.90

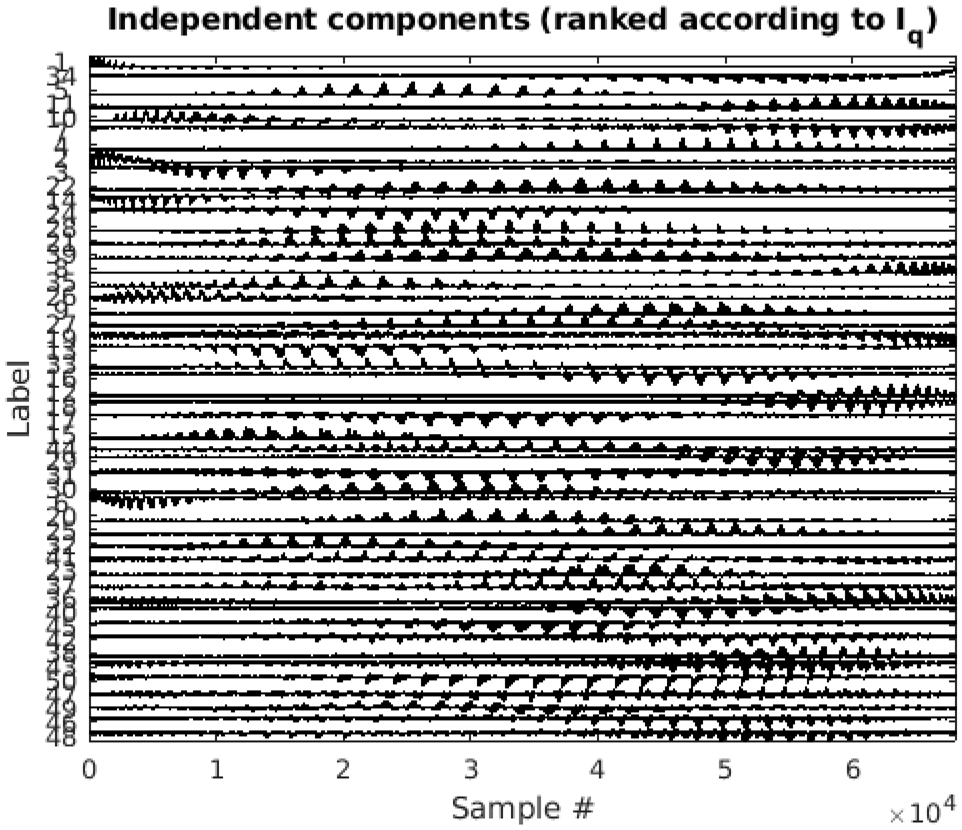
Independent components (ranked according to I$_q$)
Label
Sample #
0
1
2
3
4
5
6
×10^4

Mean Components

Mean across all subjects and sessions is computed for each component

- **a) Timecourse** - Mean timecourse is converted to z-scores.
- **b) Spectra** - Timecourses spectra is computed for each data-set and averaged across sessions. Mean and standard error of mean is shown in the figure.
- **c) Montage** - Axial slices are shown.
- **d) Ortho slices** - Ortho plot is shown for the peak voxel and coordinates are reported.

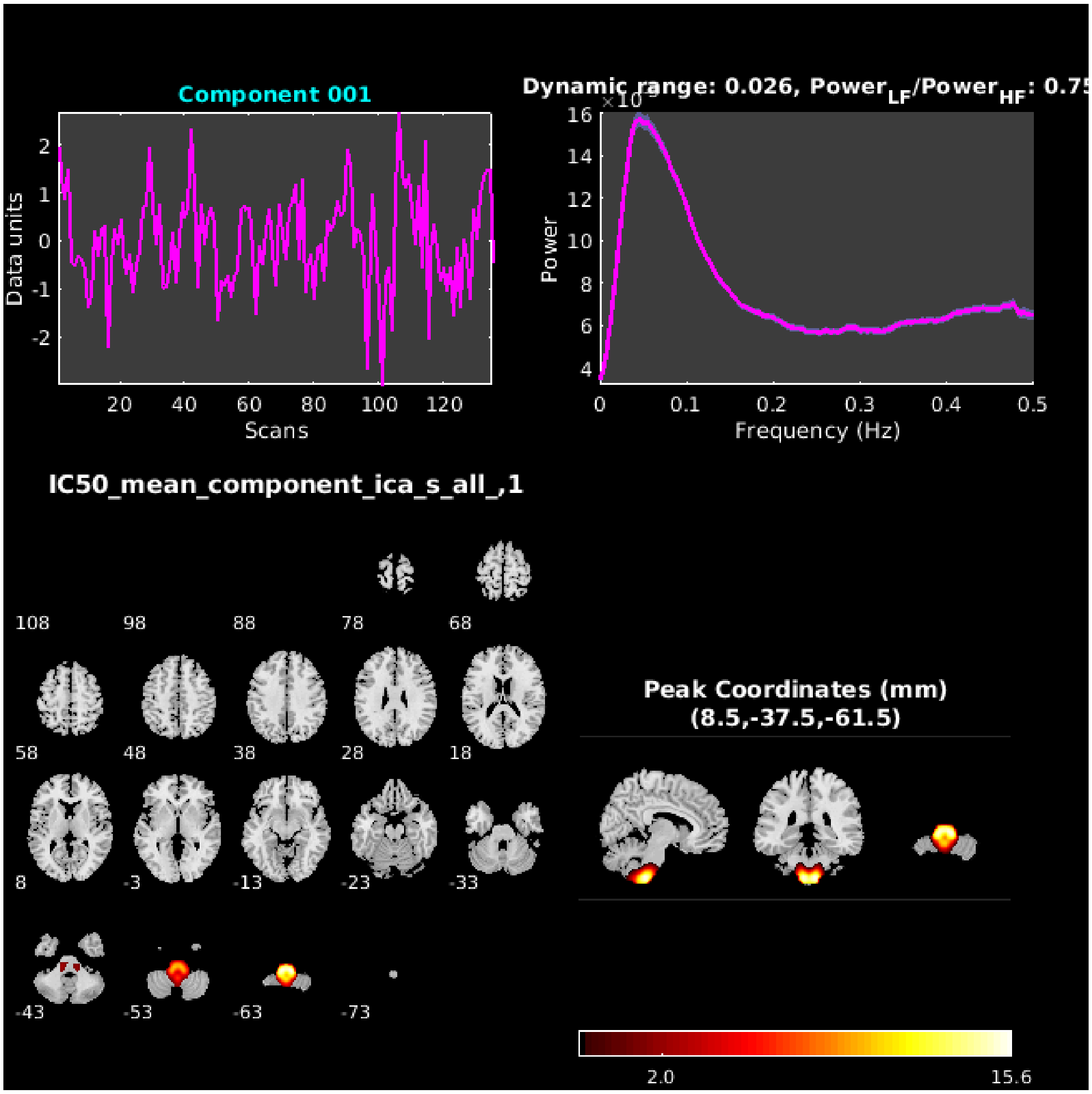

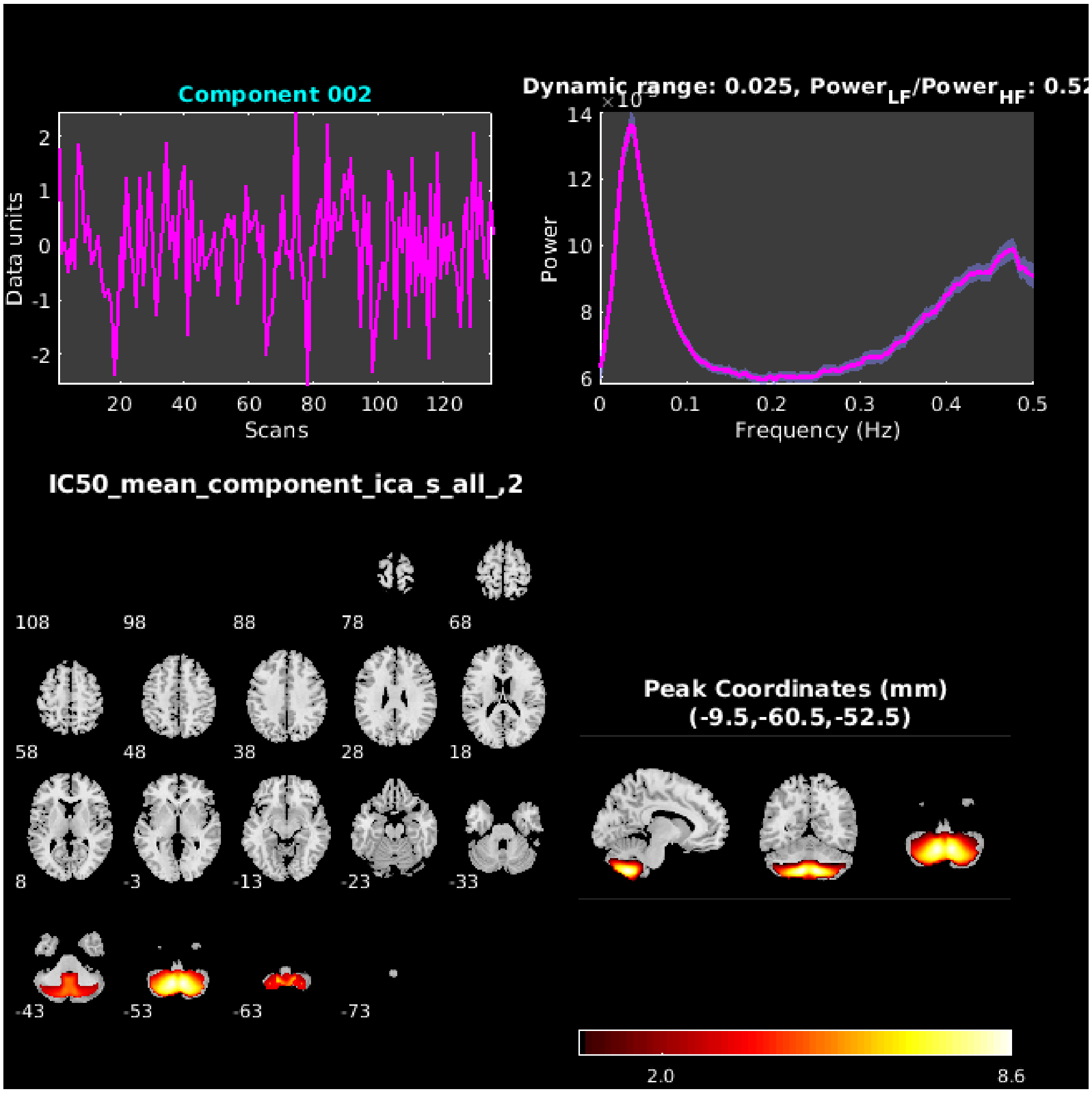

Component 002
Dynamic range: 0.025, Power LF/Power HF: 0.52
Data units
Scans
Power
Frequency (Hz)
IC50_mean_component_ica_s_all_,2
Peak Coordinates (mm)
(-9.5,-60.5,-52.5)
2.0
8.6

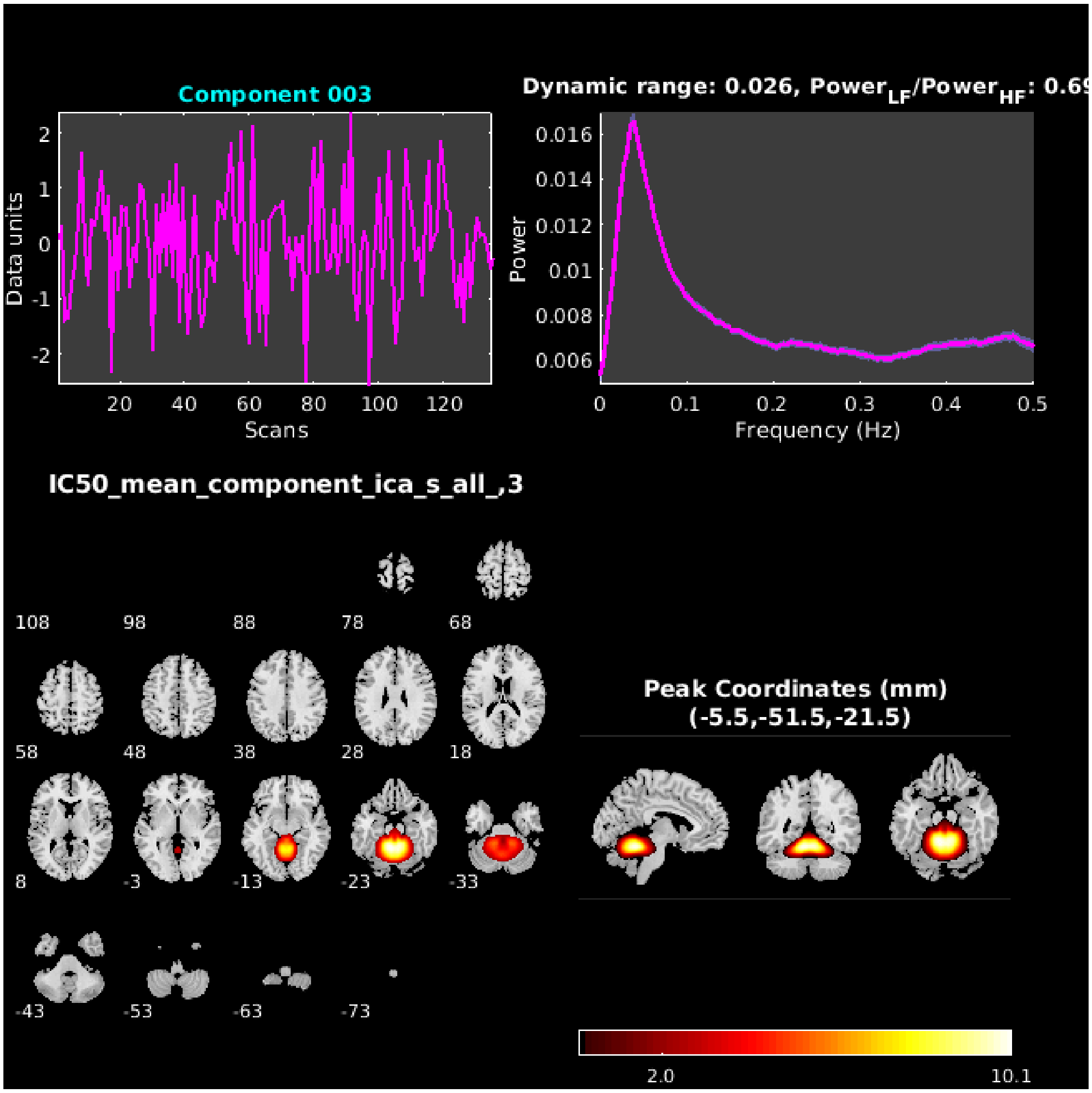

Component 003
Dynamic range: 0.026, Power$_{LF}$/Power$_{HF}$: 0.69
Data units
Scans
Power
Frequency (Hz)
IC50_mean_component_ica_s_all_,3
Peak Coordinates (mm)
(-5.5,-51.5,-21.5)
2.0
10.1

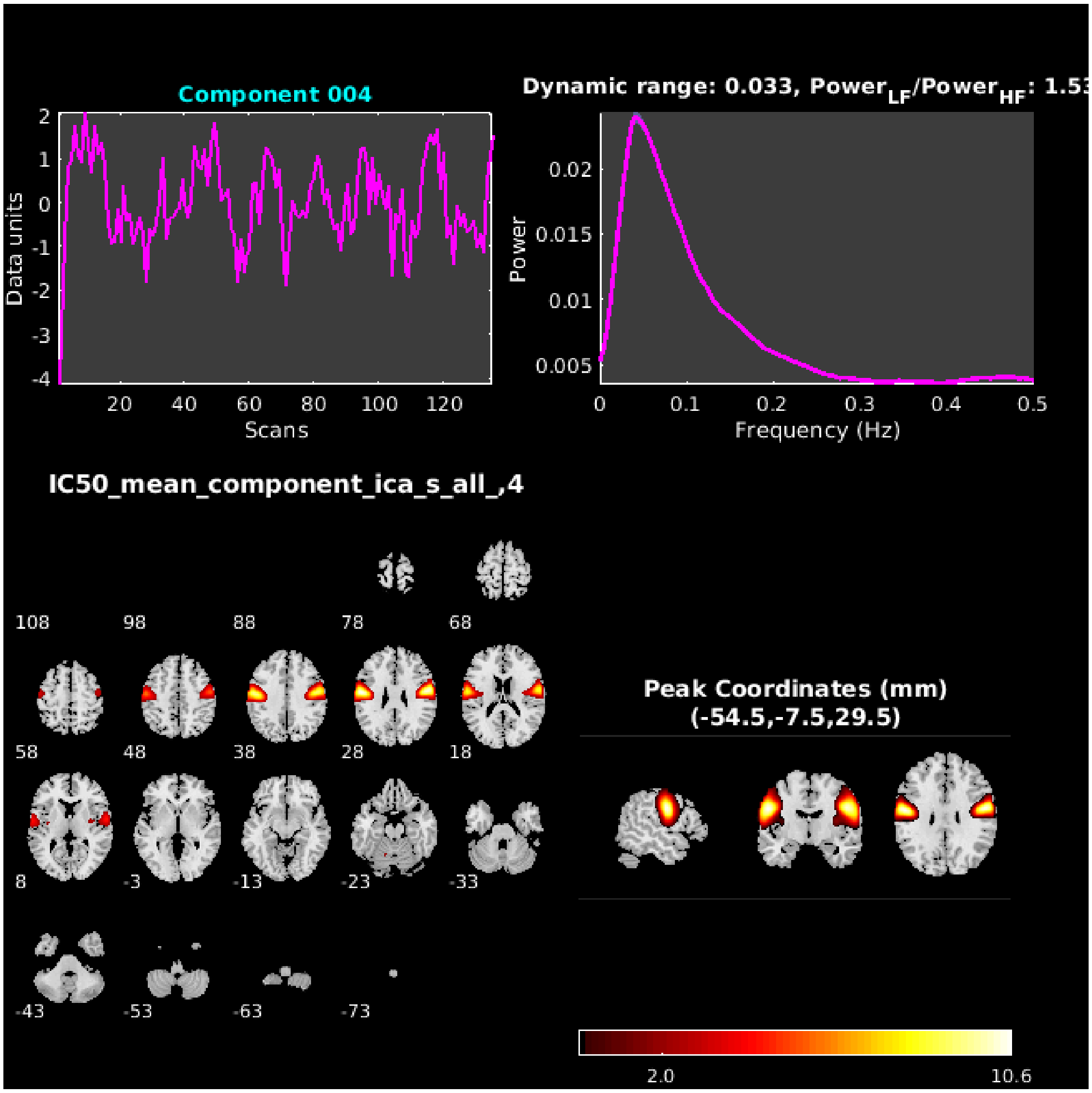
Component 004
Dynamic range: 0.033, Power$_{LF}$/Power$_{HF}$: 1.53
Data units
Scans
Power
Frequency (Hz)
IC50_mean_component_ica_s_all_,4
Peak Coordinates (mm)
(-54.5,-7.5,29.5)
2.0
10.6

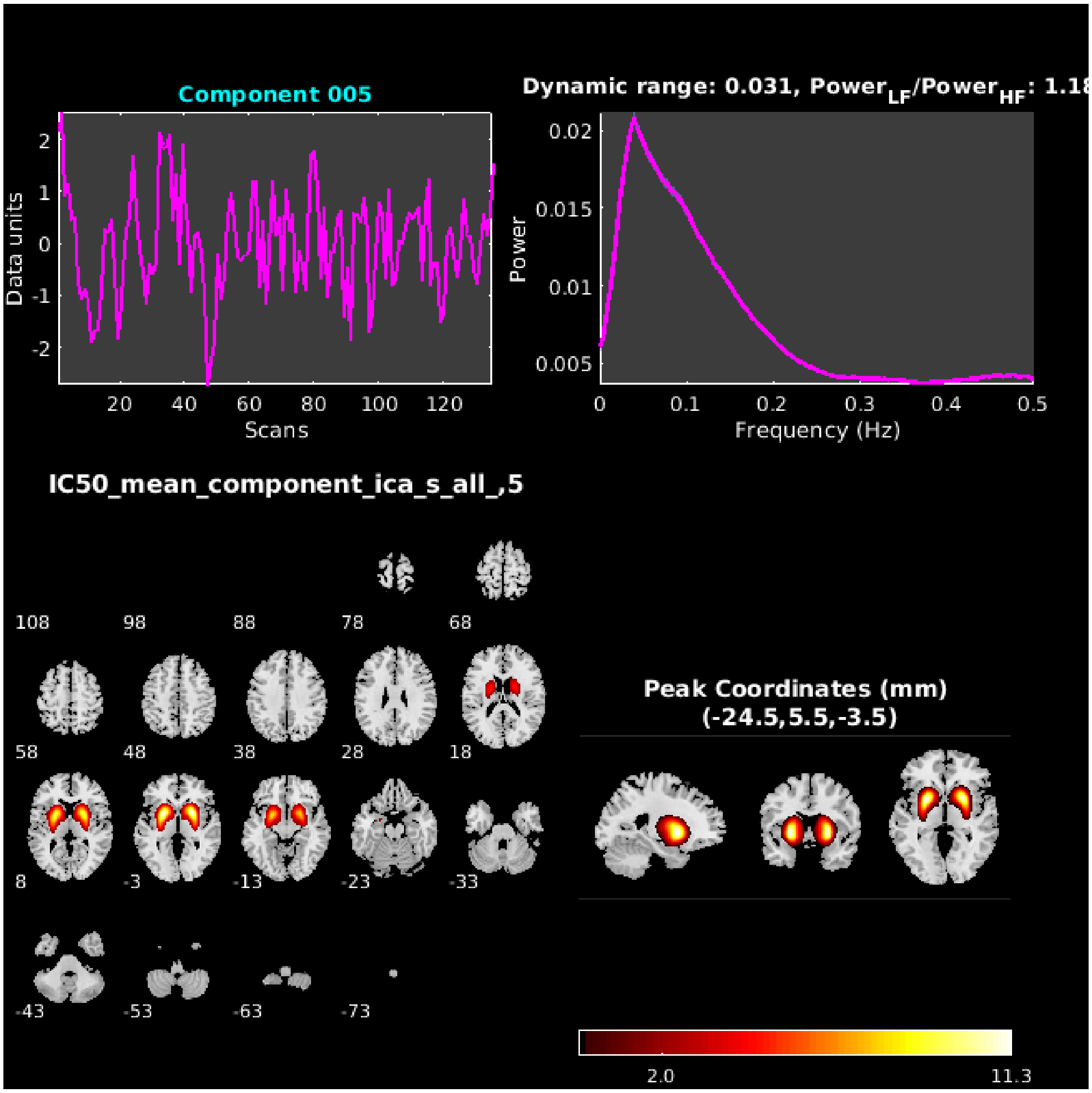
Component 005
Dynamic range: 0.031, Power_LF/Power_HF: 1.18
Data units
Scans
Power
Frequency (Hz)
IC50_mean_component_ica_s_all_,5
108 98 88 78 68
58 48 38 28 18
8 -3 -13 -23 -33
-43 -53 -63 -73
Peak Coordinates (mm)
(-24.5,5.5,-3.5)
2.0
11.3

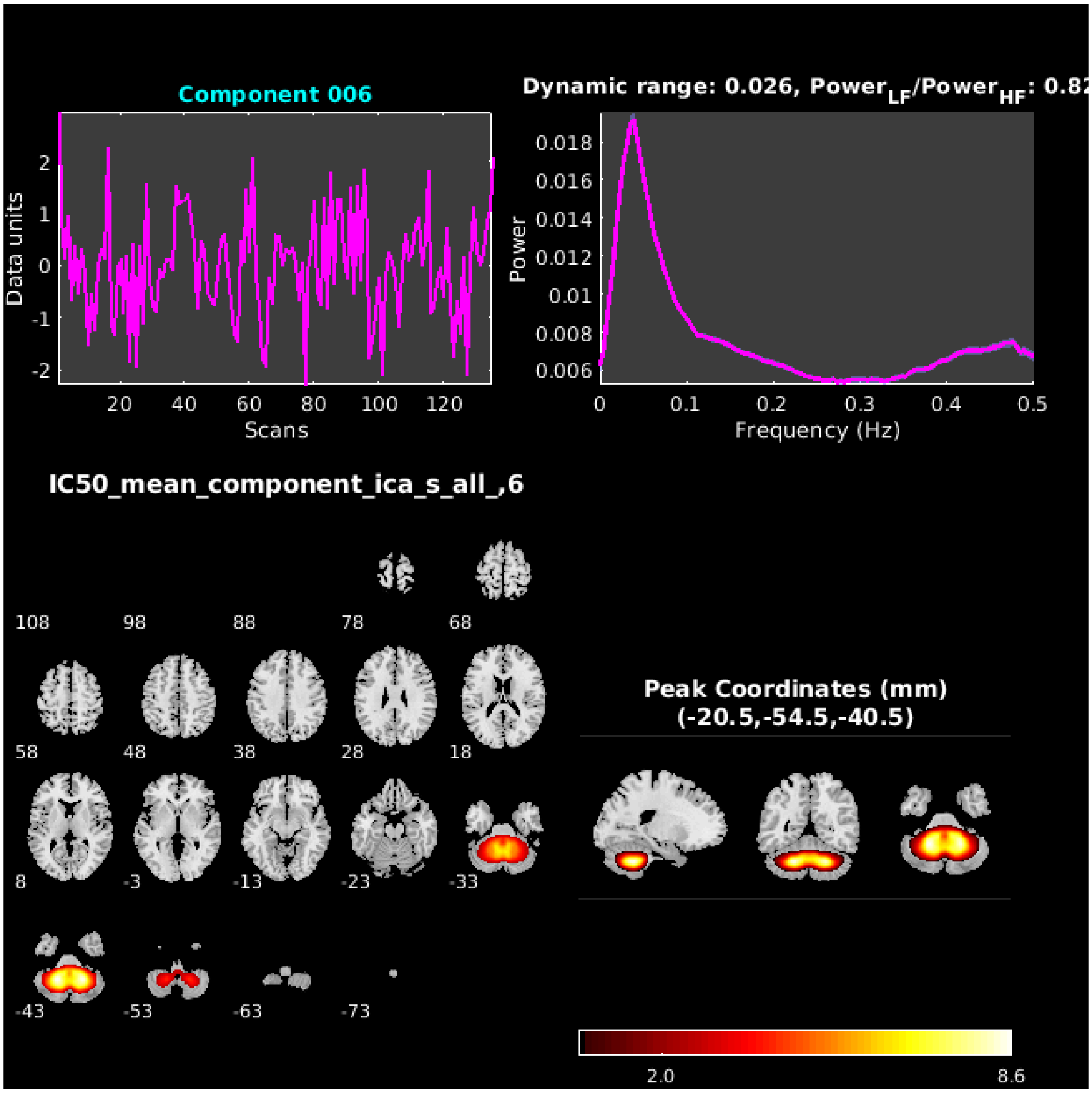

Component 006
Dynamic range: 0.026, Power$_{LF}$/Power$_{HF}$: 0.82
Data units
Scans
Power
Frequency (Hz)
IC50_mean_component_ica_s_all_,6
108
98
88
78
68
58
48
38
28
18
8
-3
-13
-23
-33
-43
-53
-63
-73
Peak Coordinates (mm)
(-20.5,-54.5,-40.5)
2.0
8.6

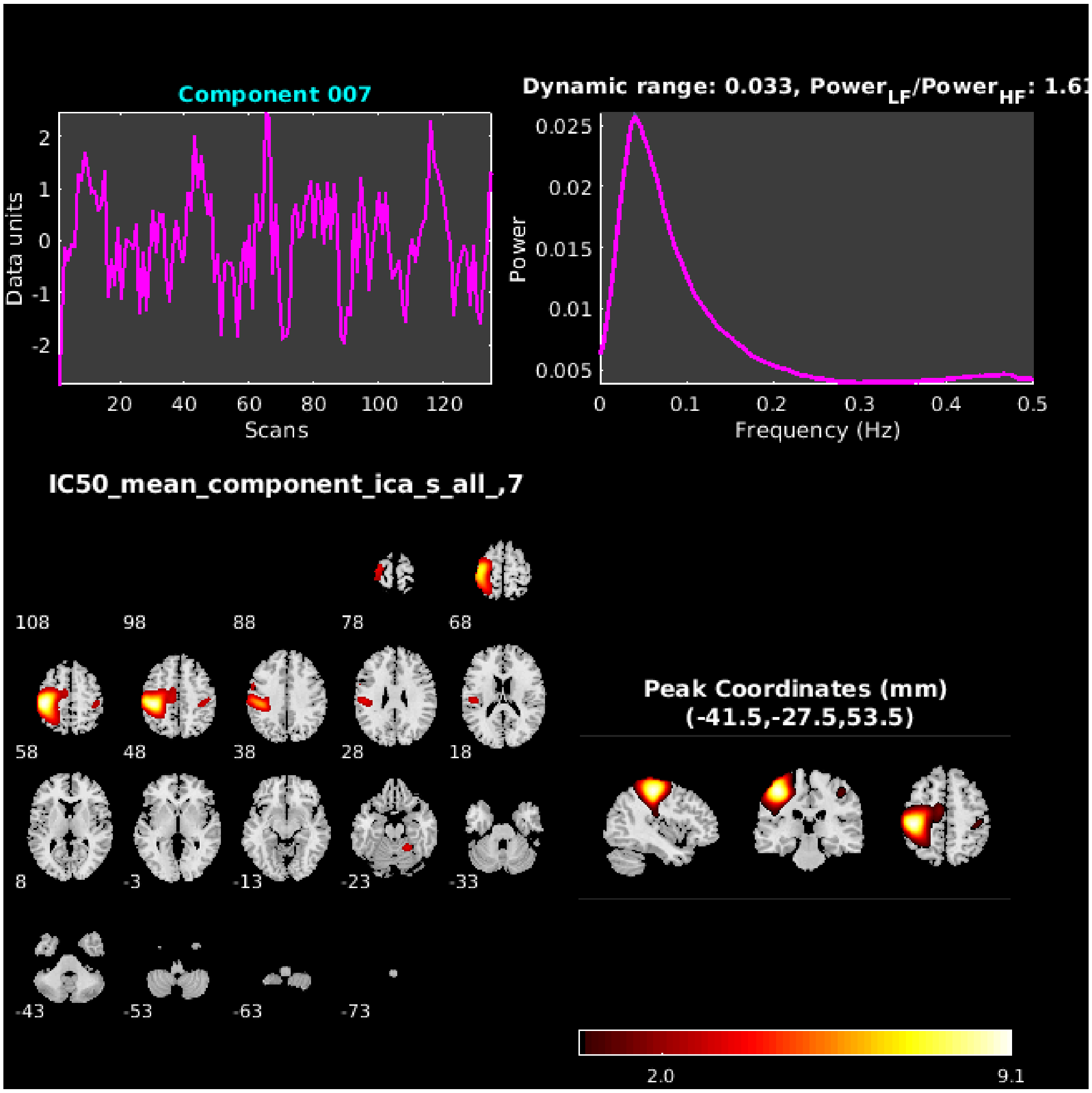

Component 007
Dynamic range: 0.033, Power$_{LF}$/Power$_{HF}$: 1.6
Data units
Scans
Power
Frequency (Hz)
IC50_mean_component_ica_s_all_,7
108
98
88
78
68
58
48
38
28
18
8
-3
-13
-23
-33
-43
-53
-63
-73
Peak Coordinates (mm)
(-41.5,-27.5,53.5)
2.0
9.1

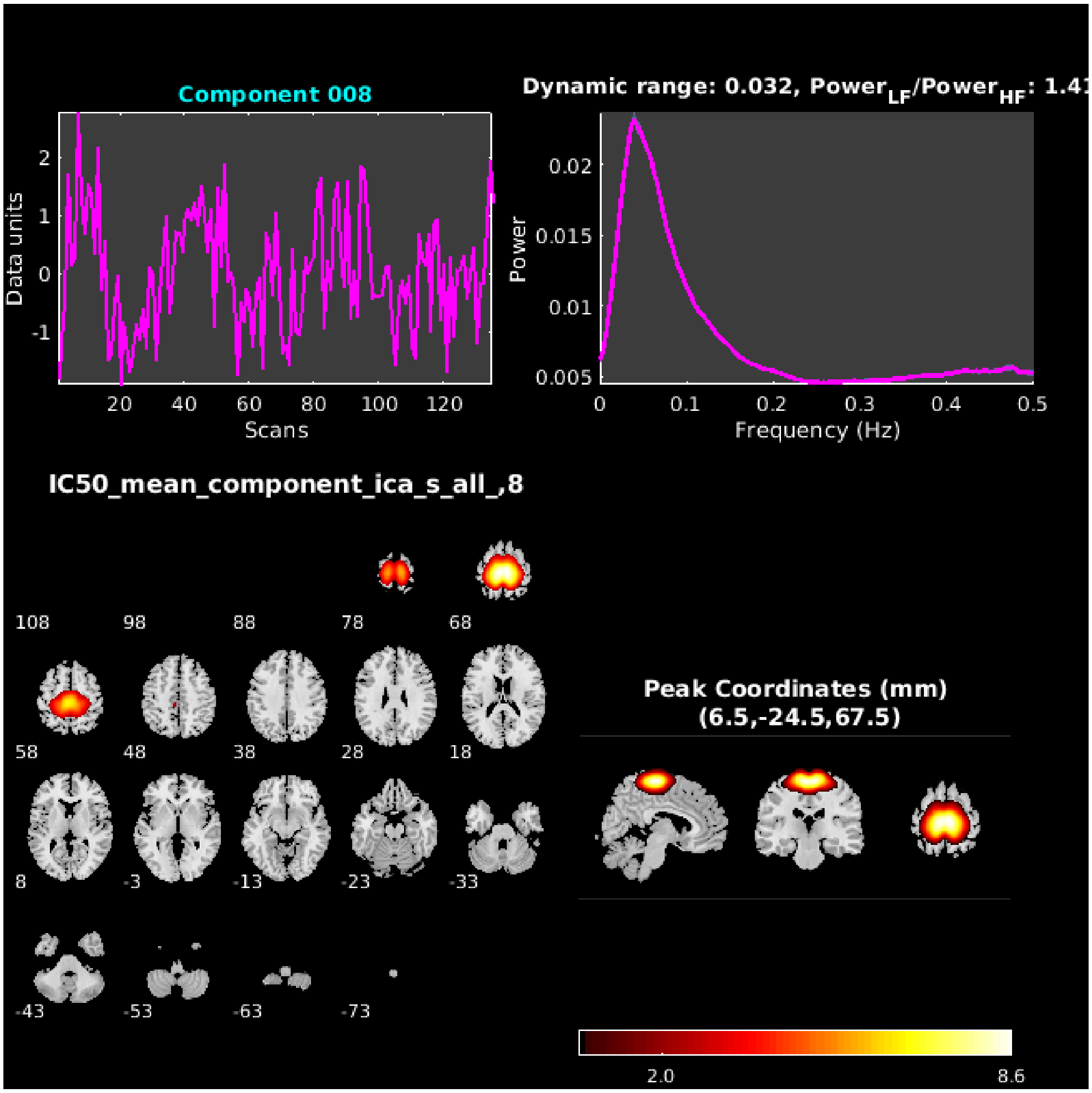

Component 008
Dynamic range: 0.032, Power$_{LF}$/Power$_{HF}$: 1.41
Data units
Scans
Power
Frequency (Hz)
IC50_mean_component_ica_s_all_,8
Peak Coordinates (mm)
(6.5,-24.5,67.5)
2.0
8.6

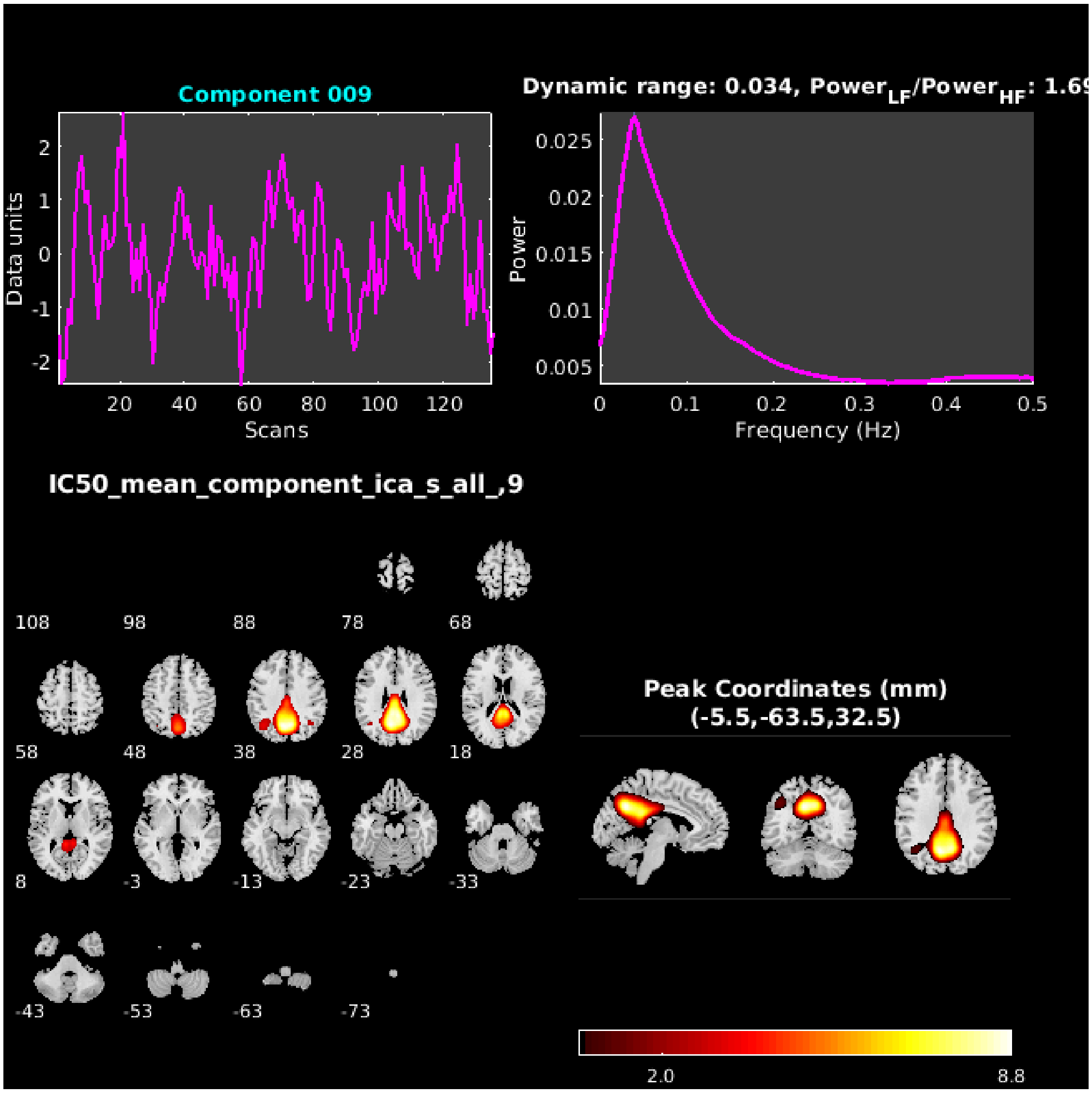

Component 009
Dynamic range: 0.034, Power$_{LF}$/Power$_{HF}$: 1.69
Data units
Scans
Power
Frequency (Hz)
IC50_mean_component_ica_s_all_,9
Peak Coordinates (mm)
(-5.5,-63.5,32.5)
2.0
8.8

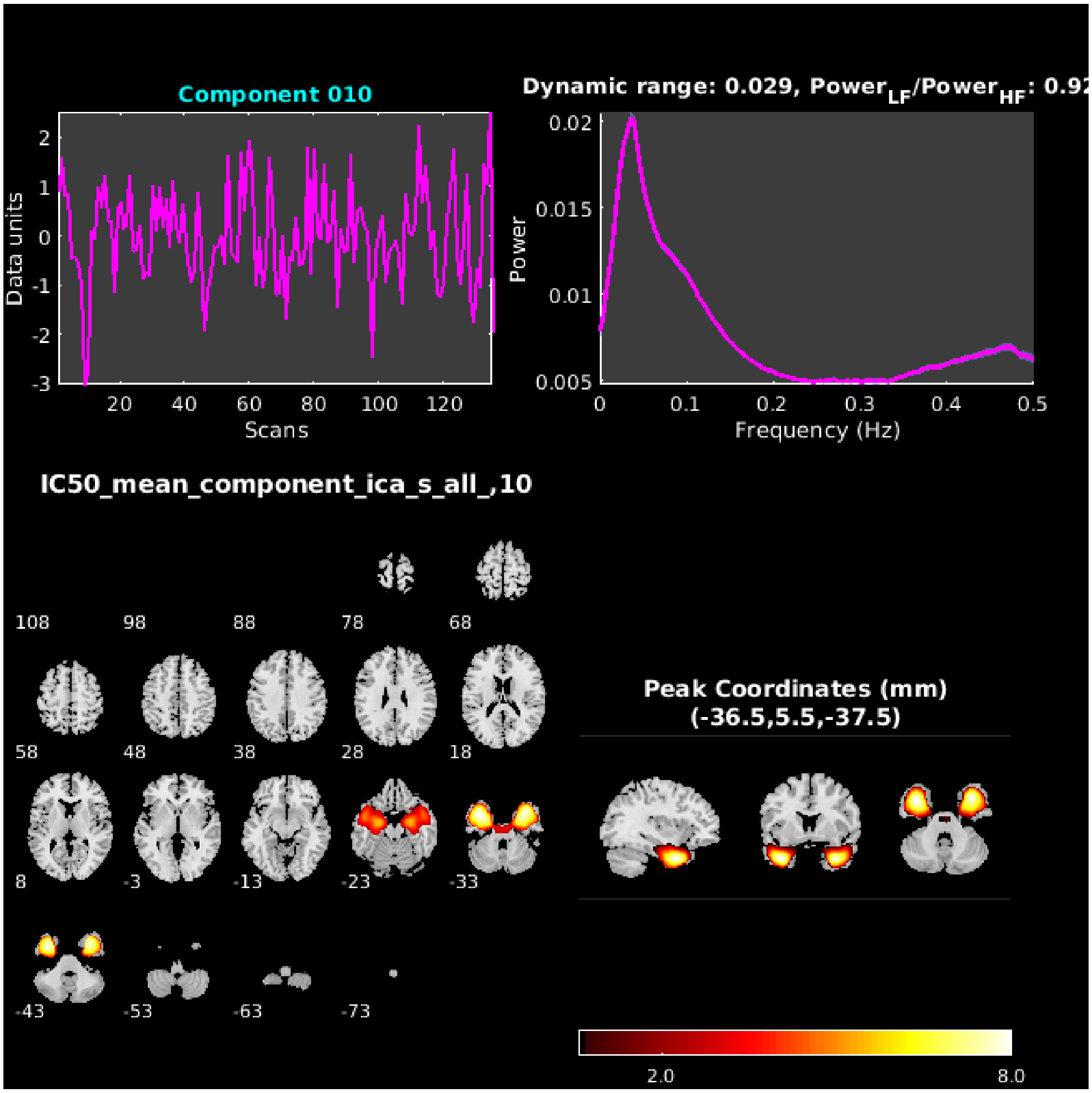

Component 010
Dynamic range: 0.029, Power_LF/Power_HF: 0.9
Data units
Scans
Power
Frequency (Hz)
IC50_mean_component_ica_s_all_,10
108 98 88 78 68
58 48 38 28 18
8 -3 -13 -23 -33
-43 -53 -63 -73
Peak Coordinates (mm)
(-36.5,5.5,-37.5)
2.0
8.0

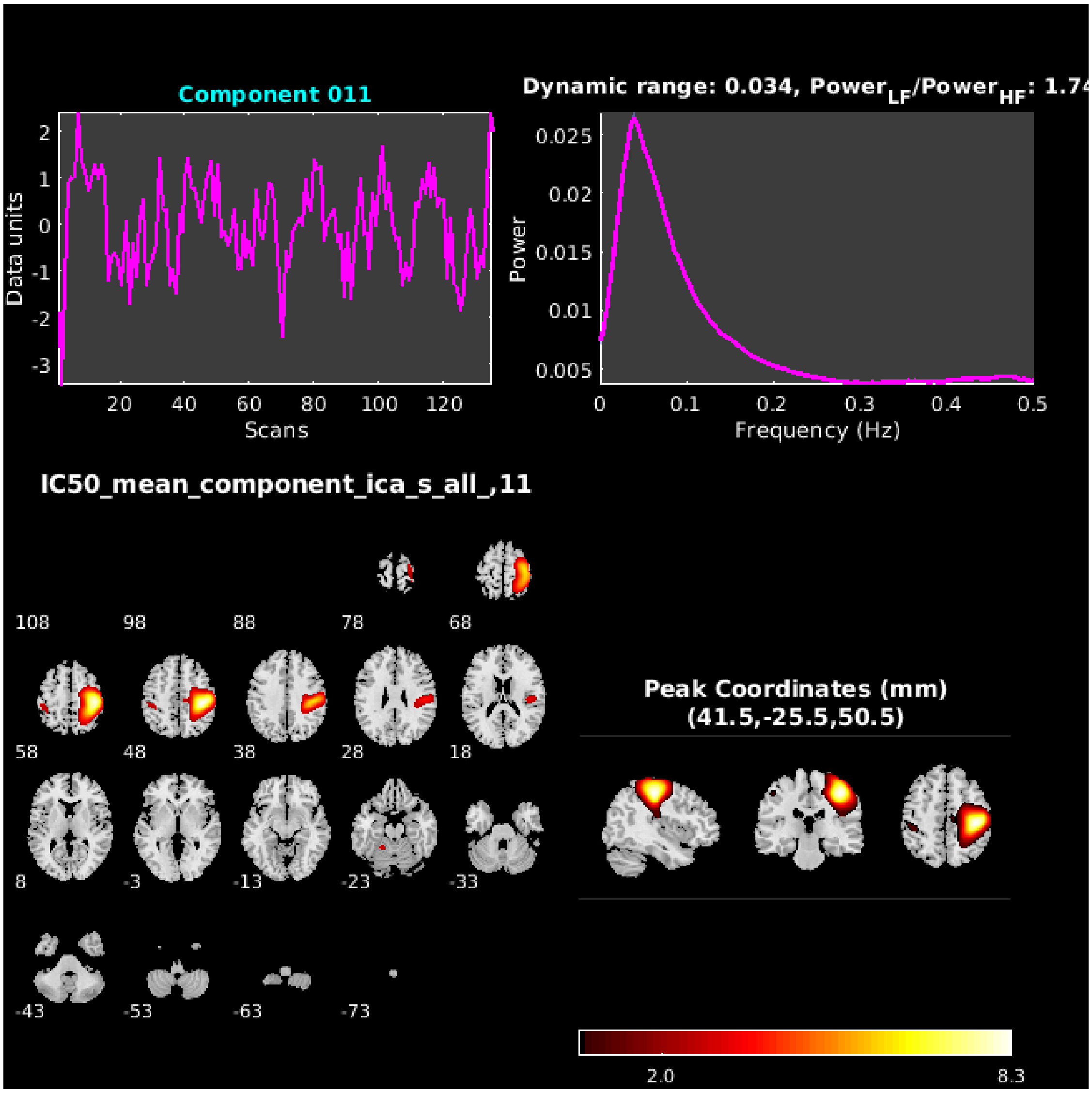
Component 011
Dynamic range: 0.034, Power$_{LF}$/Power$_{HF}$: 1.74
Data units
Scans
Power
Frequency (Hz)
IC50_mean_component_ica_s_all_,11
Peak Coordinates (mm)
(41.5,-25.5,50.5)
2.0
8.3

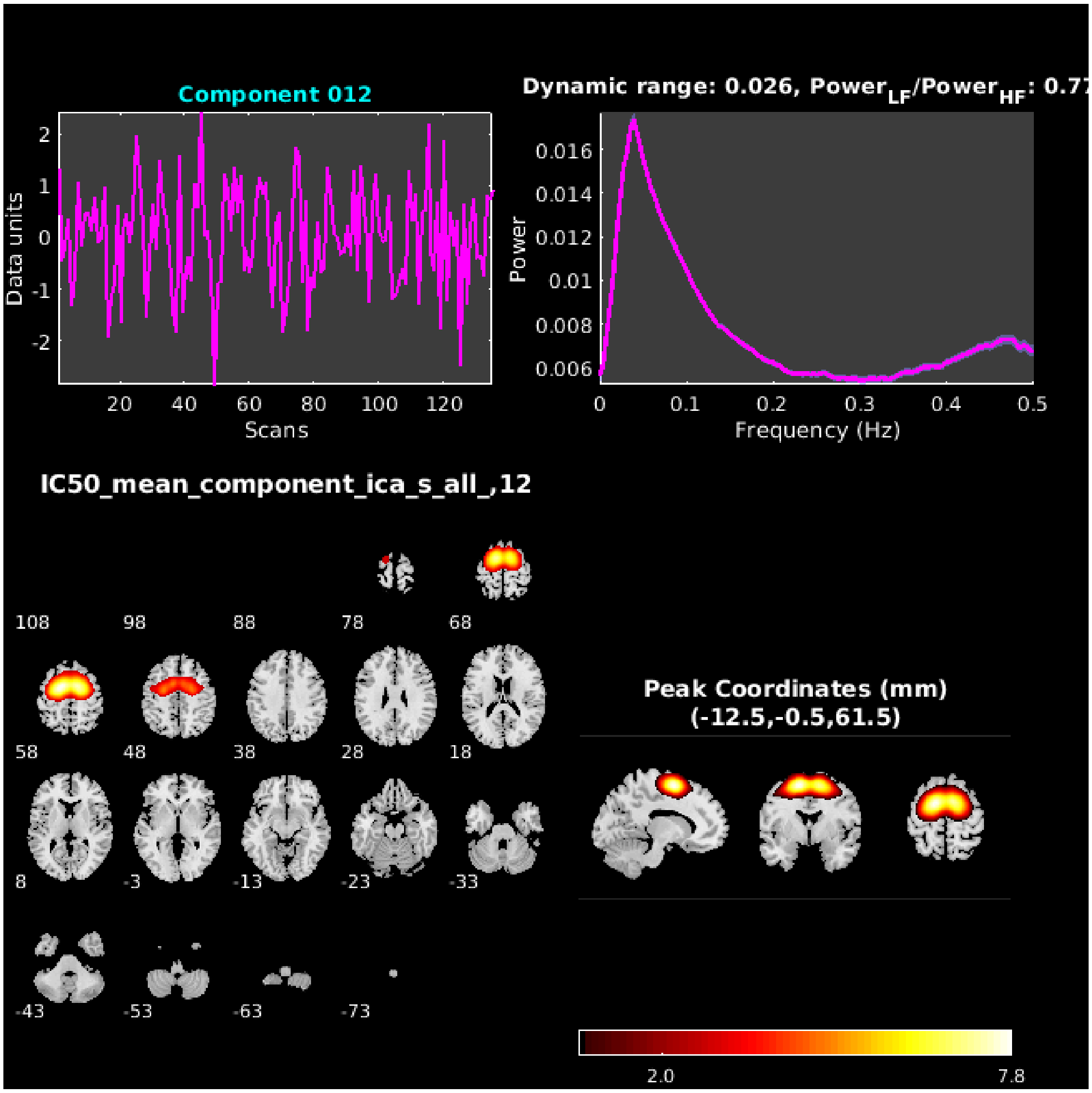
Component 012
Dynamic range: 0.026, Power_LF/Power_HF: 0.77
Data units
Scans
Power
Frequency (Hz)
IC50_mean_component_ica_s_all_,12
108
98
88
78
68
58
48
38
28
18
8
-3
-13
-23
-33
-43
-53
-63
-73
Peak Coordinates (mm)
(-12.5,-0.5,61.5)
2.0
7.8

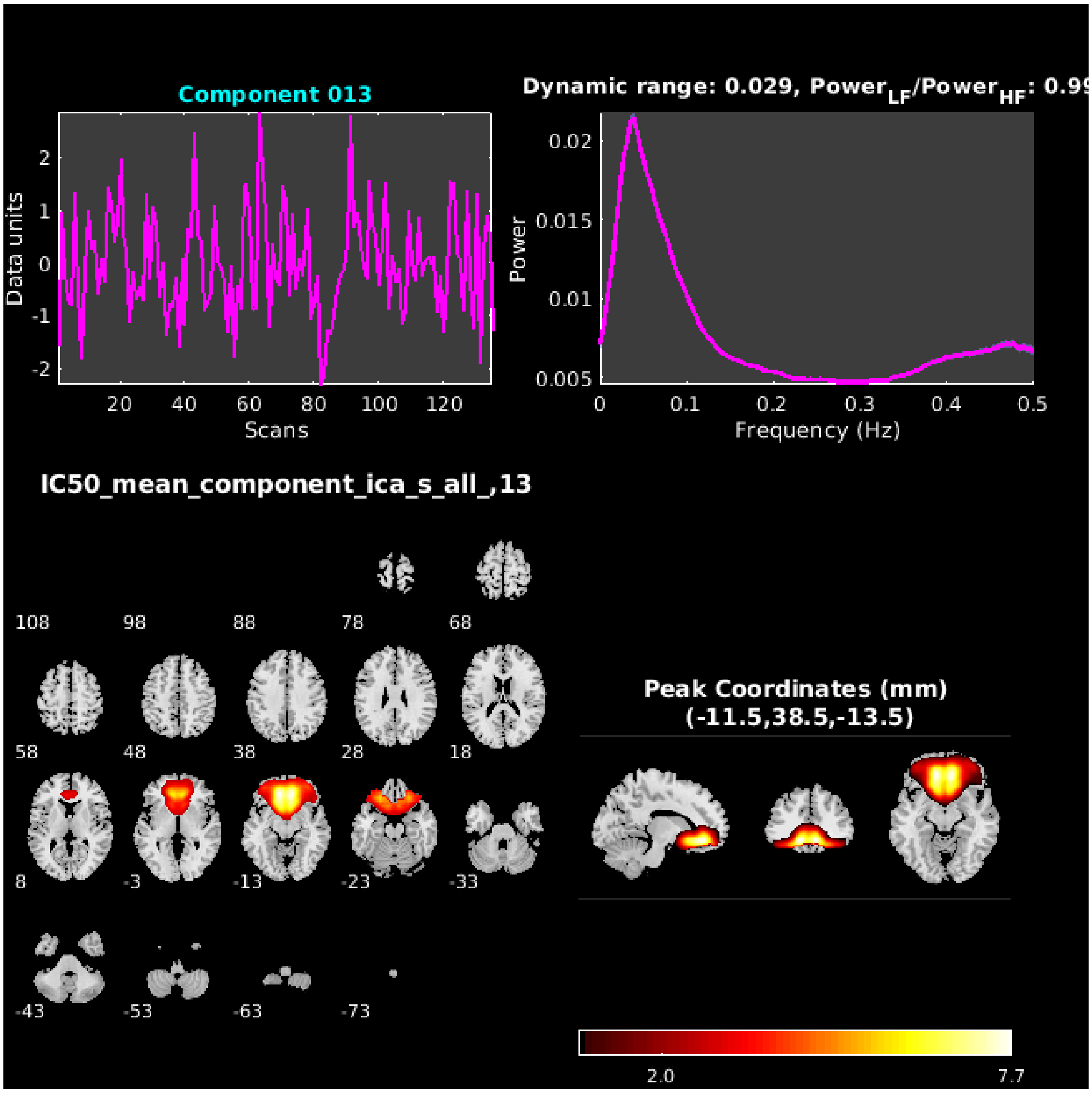

Component 013
Dynamic range: 0.029, Power_LF/Power_HF: 0.99
Data units
Scans
Power
Frequency (Hz)
IC50_mean_component_ica_s_all_,13
Peak Coordinates (mm)
(-11.5,38.5,-13.5)
2.0
7.7

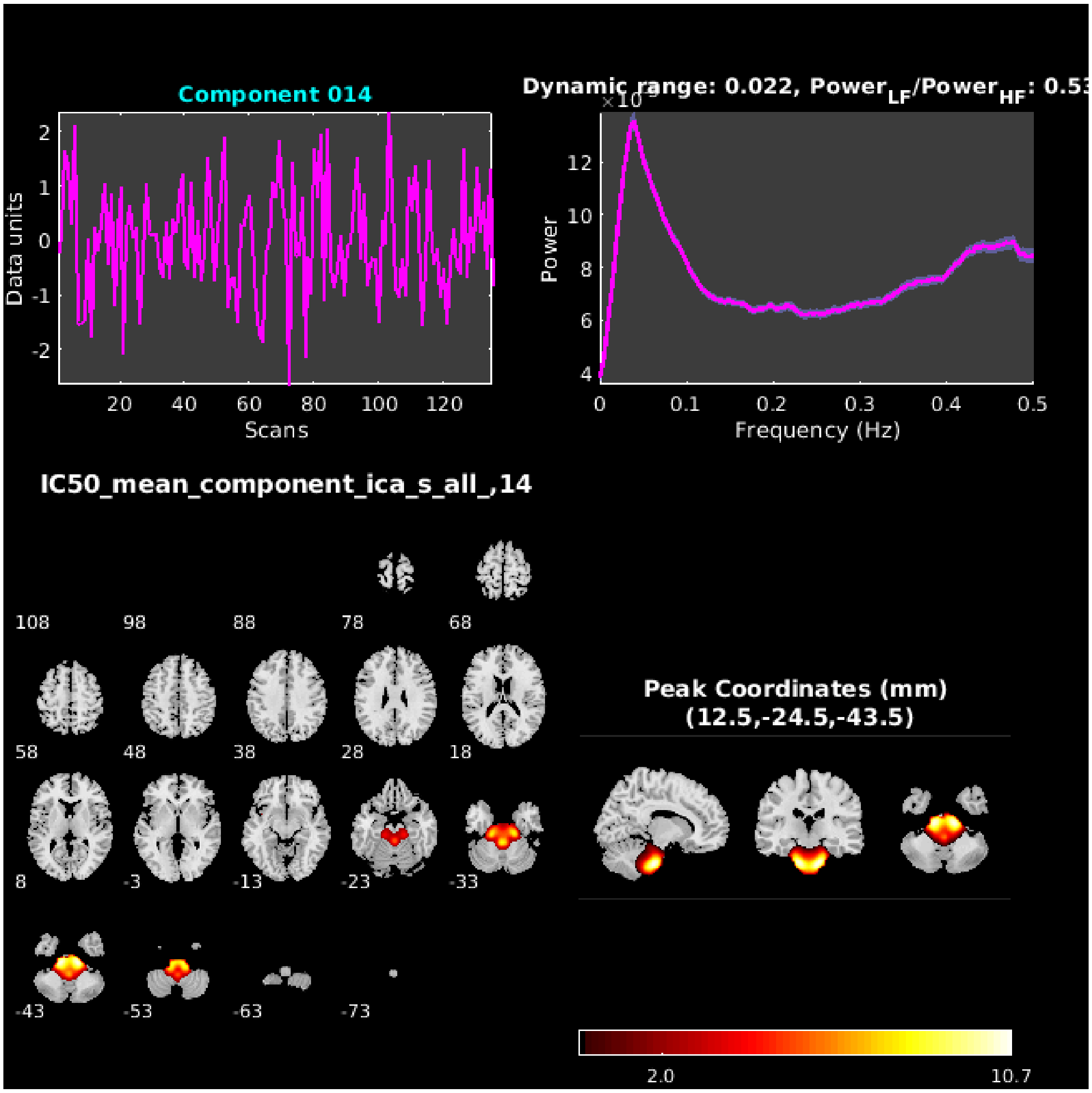

Component 014
Dynamic range: 0.022, Power_LF/Power_HF: 0.53
Data units
Scans
Power
Frequency (Hz)
IC50_mean_component_ica_s_all_,14
Peak Coordinates (mm)
(12.5,-24.5,-43.5)
2.0
10.7

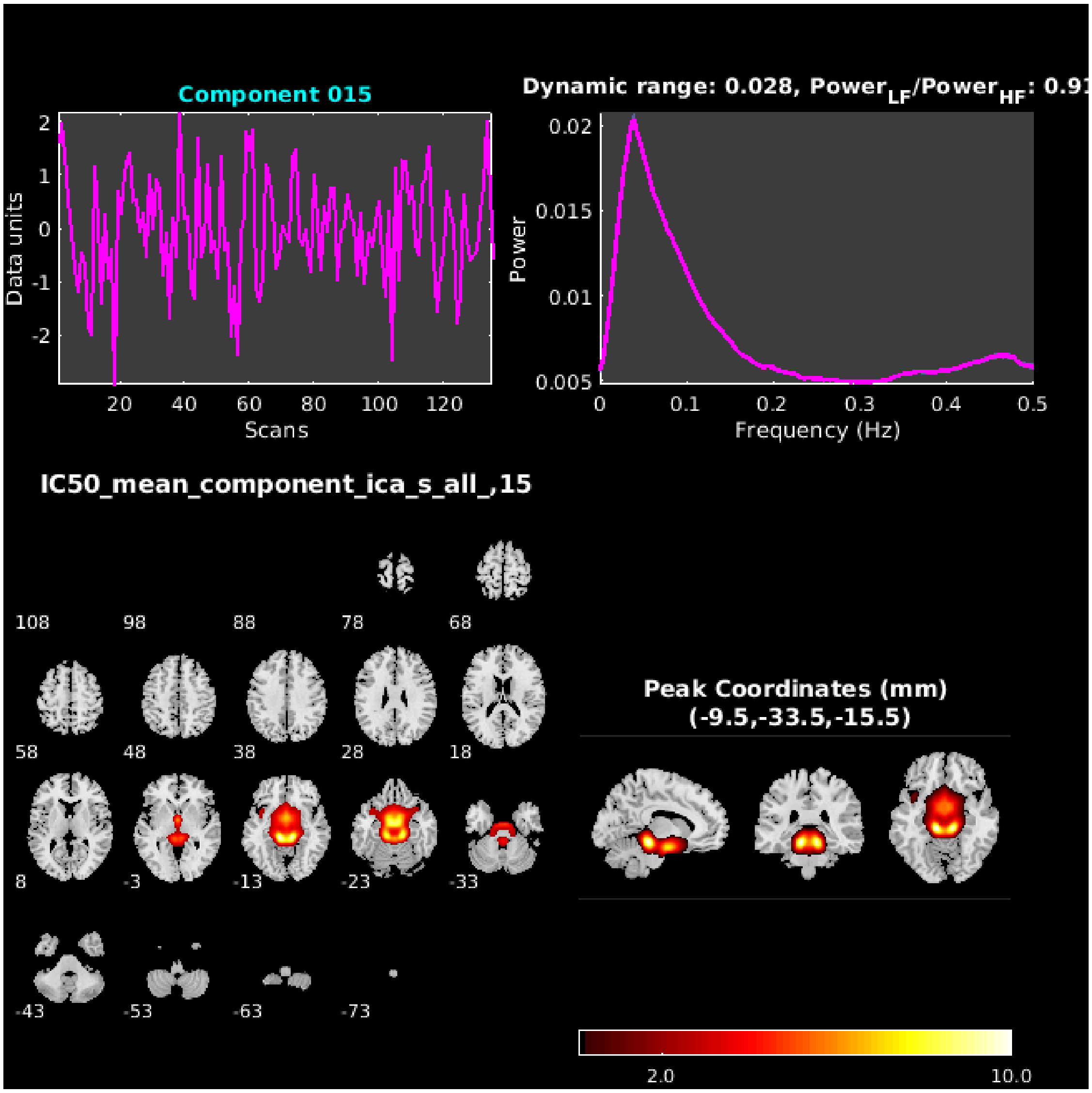

Component 015
Dynamic range: 0.028, Power$_{LF}$/Power$_{HF}$: 0.91
Data units
Scans
Power
Frequency (Hz)
IC50_mean_component_ica_s_all_,15
Peak Coordinates (mm)
(-9.5,-33.5,-15.5)
2.0
10.0

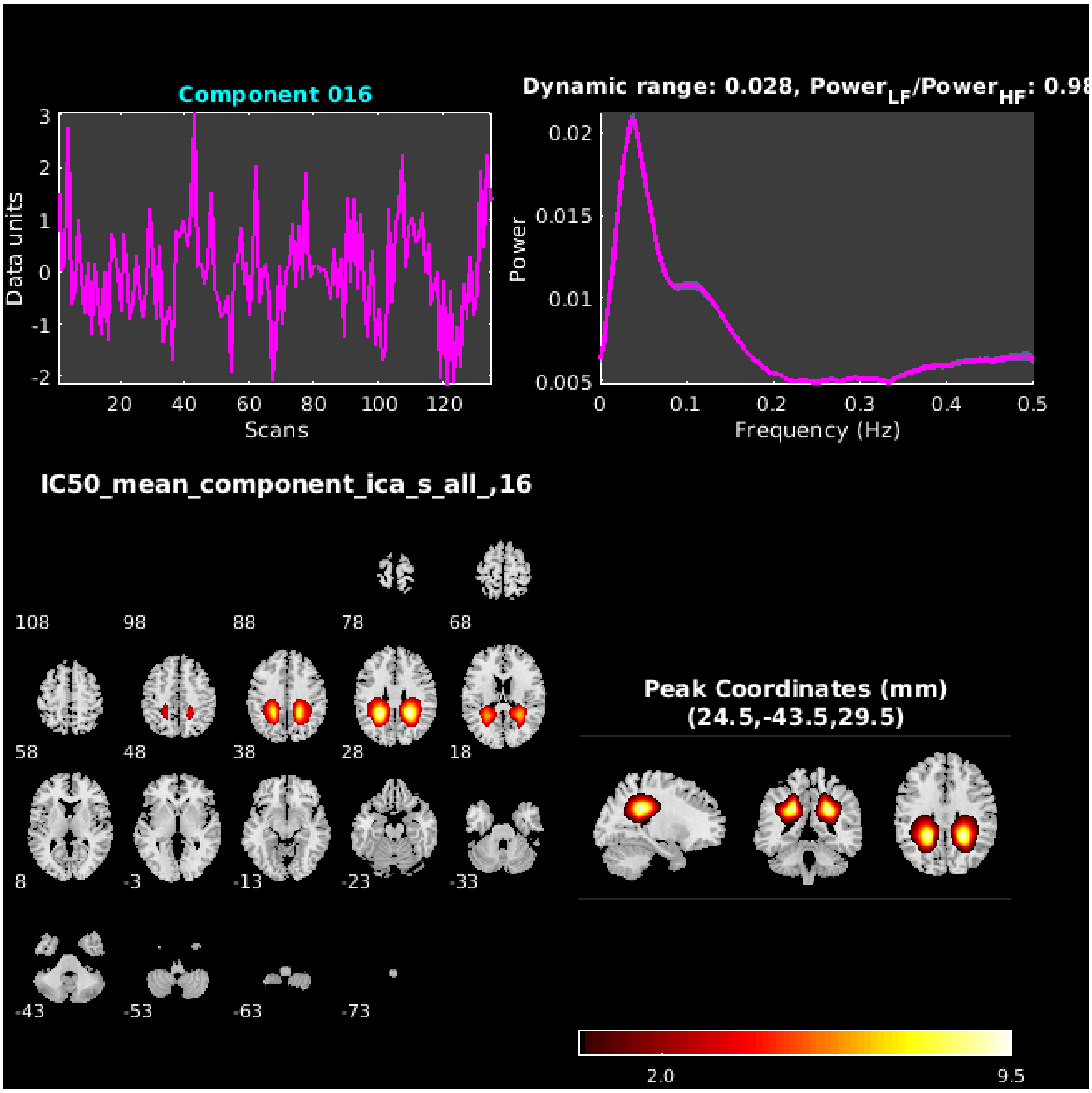

Component 016
Data units
Scans
Dynamic range: 0.028, Power$_{LF}$/Power$_{HF}$: 0.98
Power
Frequency (Hz)
IC50_mean_component_ica_s_all_,16
108
98
88
78
68
58
48
38
28
18
8
-3
-13
-23
-33
-43
-53
-63
-73
Peak Coordinates (mm)
(24.5,-43.5,29.5)
2.0
9.5

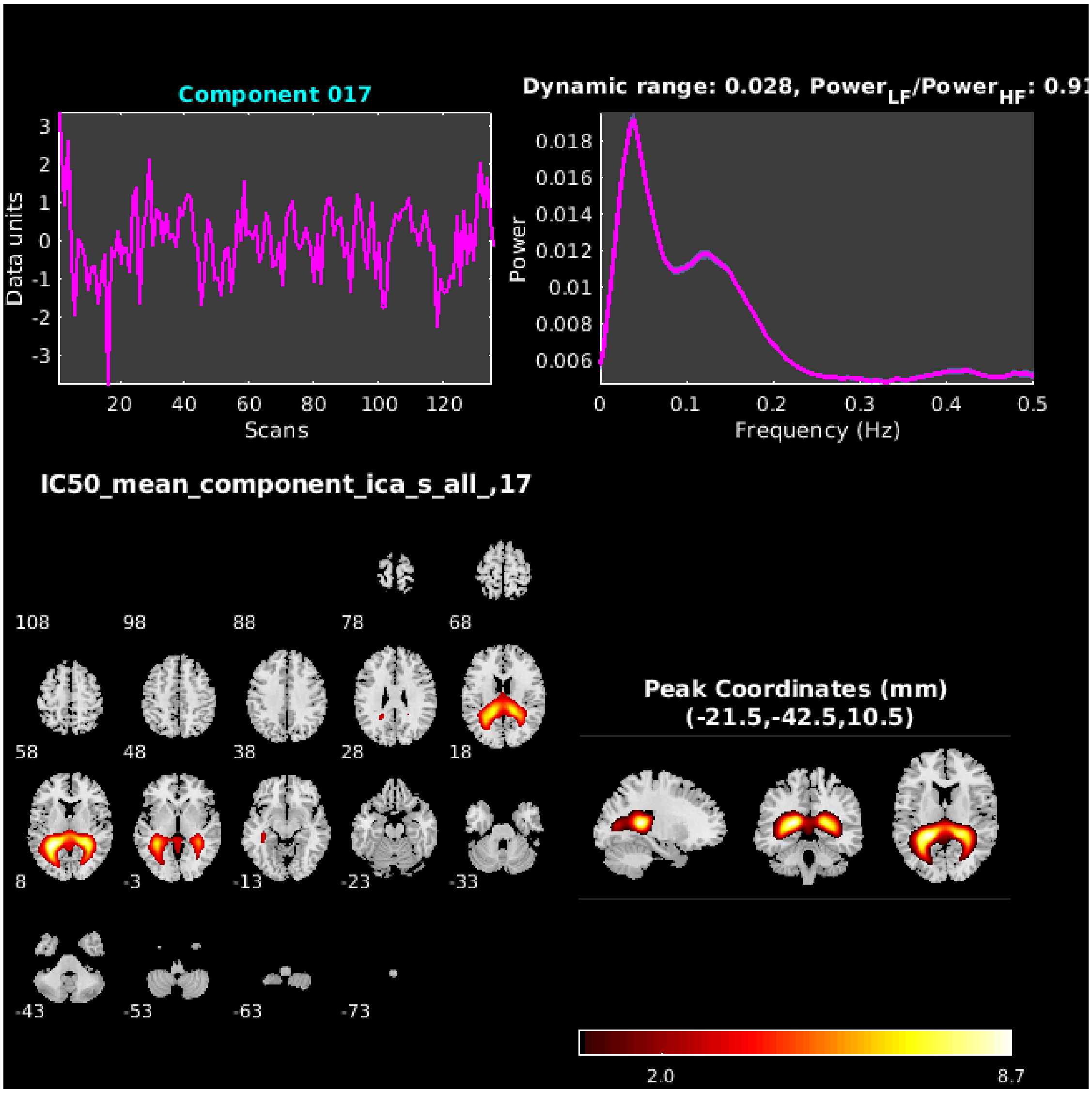

Component 017
Dynamic range: 0.028, Power$_{LF}$/Power$_{HF}$: 0.91
Data units
Scans
Power
Frequency (Hz)
IC50_mean_component_ica_s_all_,17
Peak Coordinates (mm)
(-21.5,-42.5,10.5)
2.0
8.7

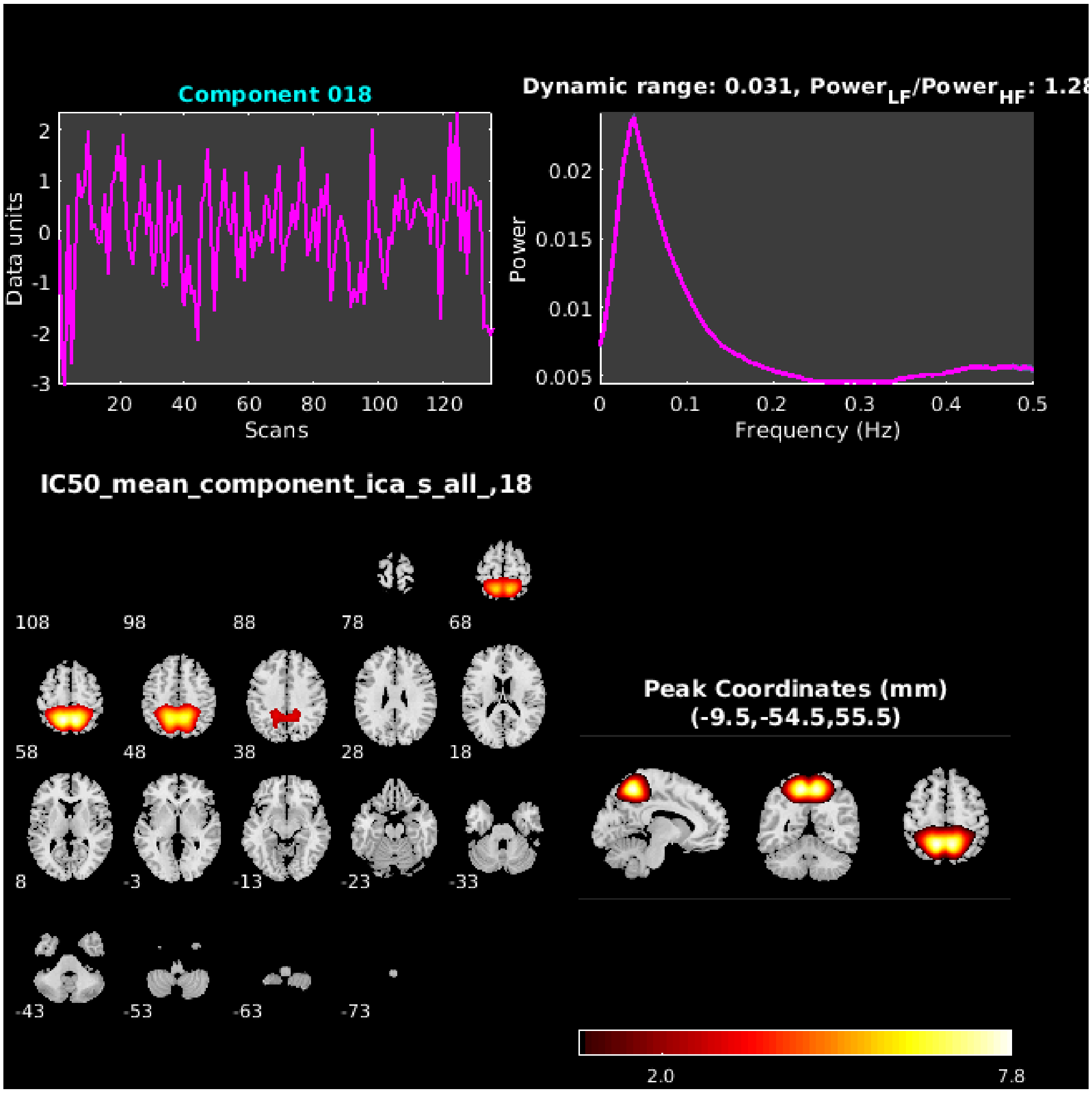
Component 018
Dynamic range: 0.031, Power$_{LF}$/Power$_{HF}$: 1.28
Data units
Scans
Power
Frequency (Hz)
IC50_mean_component_ica_s_all_,18
Peak Coordinates (mm)
(-9.5,-54.5,55.5)
2.0
7.8

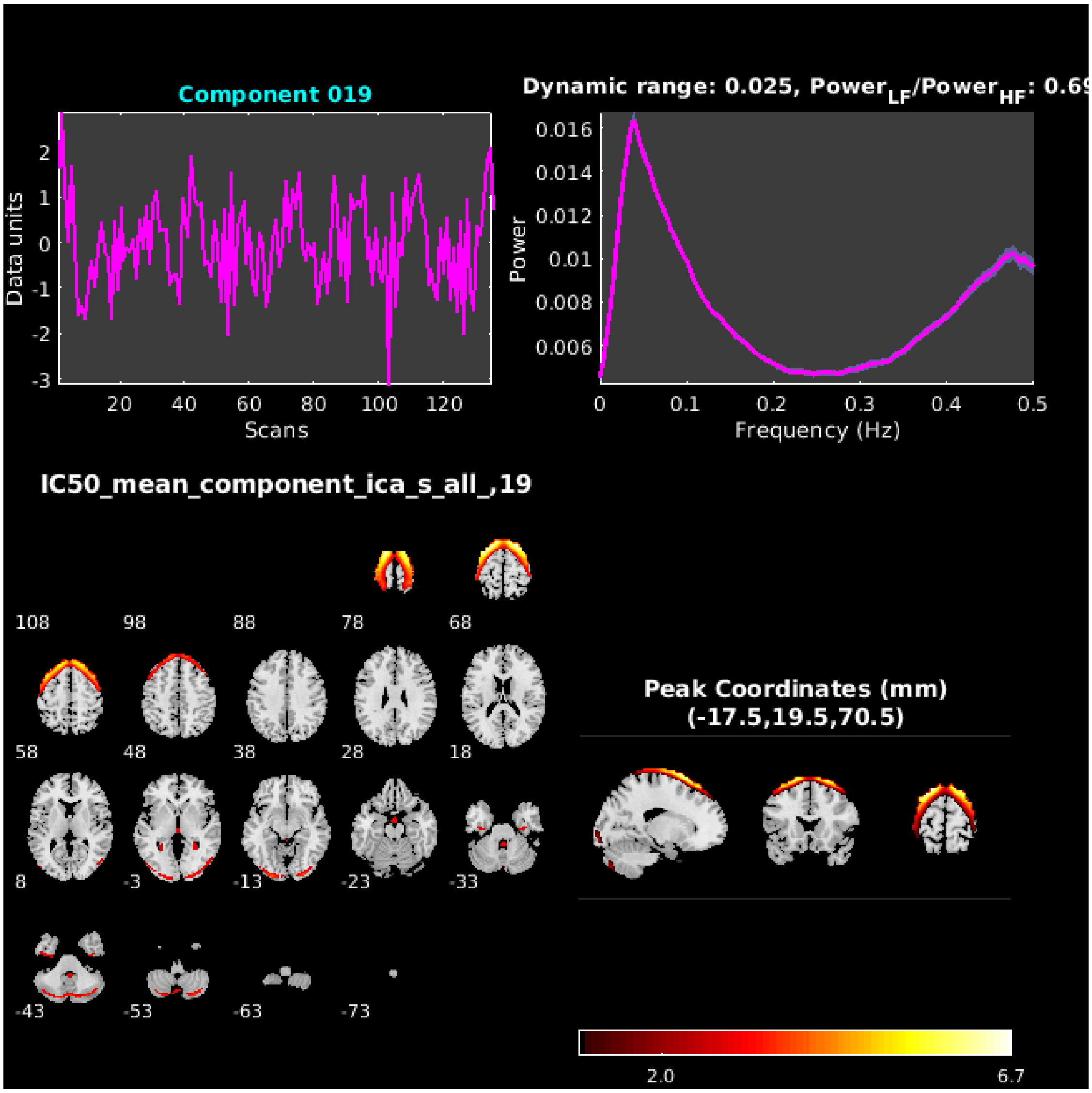

Component 019
Dynamic range: 0.025, Power_LF/Power_HF: 0.69
Data units
Scans
Power
Frequency (Hz)
IC50_mean_component_ica_s_all_,19
108
98
88
78
68
58
48
38
28
18
8
-3
-13
-23
-33
-43
-53
-63
-73
Peak Coordinates (mm)
(-17.5,19.5,70.5)
2.0
6.7

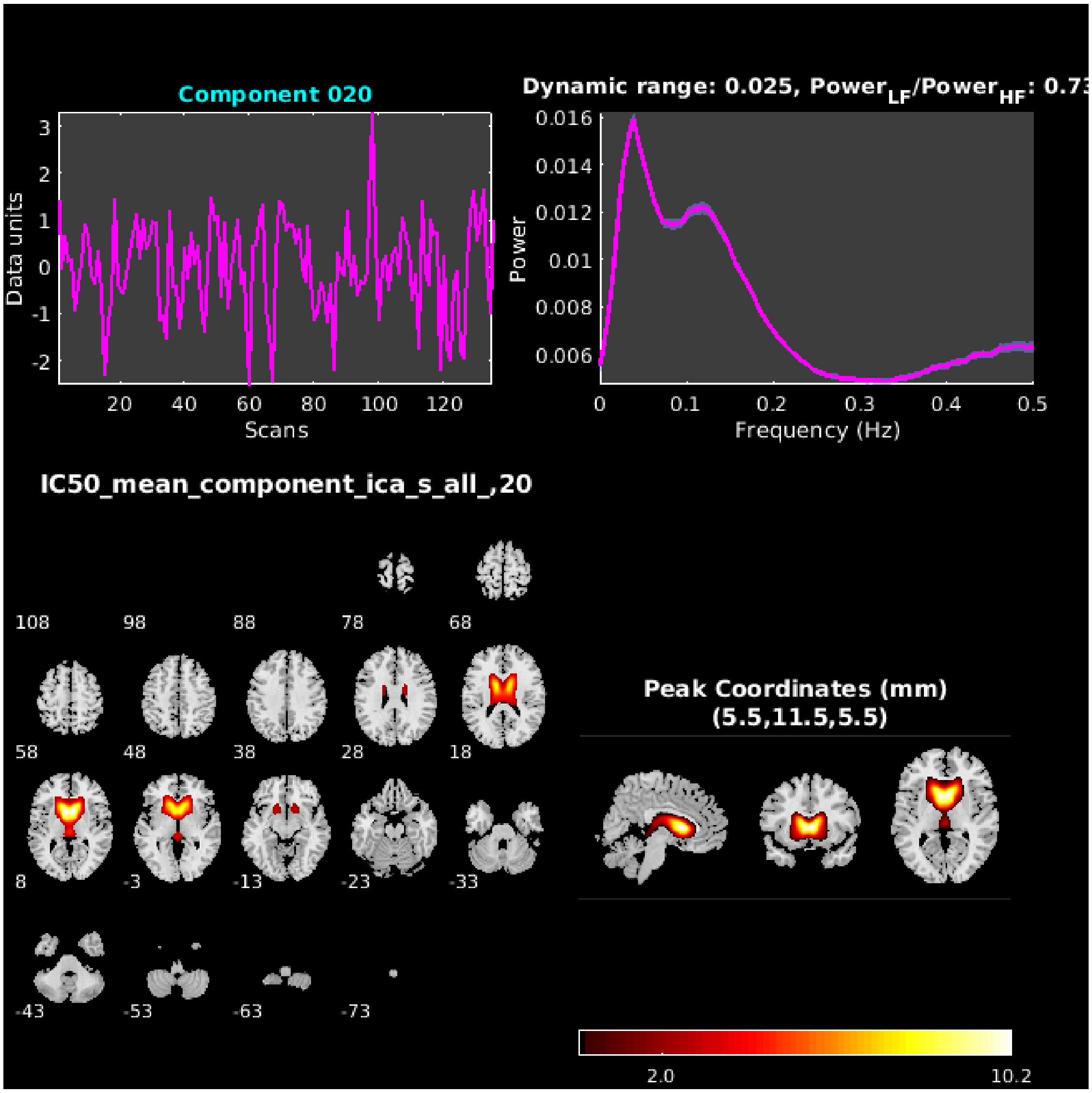

Component 020
Data units
Scans
Dynamic range: 0.025, Power_LF/Power_HF: 0.73
Power
Frequency (Hz)
IC50_mean_component_ica_s_all_,20
Peak Coordinates (mm)
(5.5,11.5,5.5)
2.0
10.2

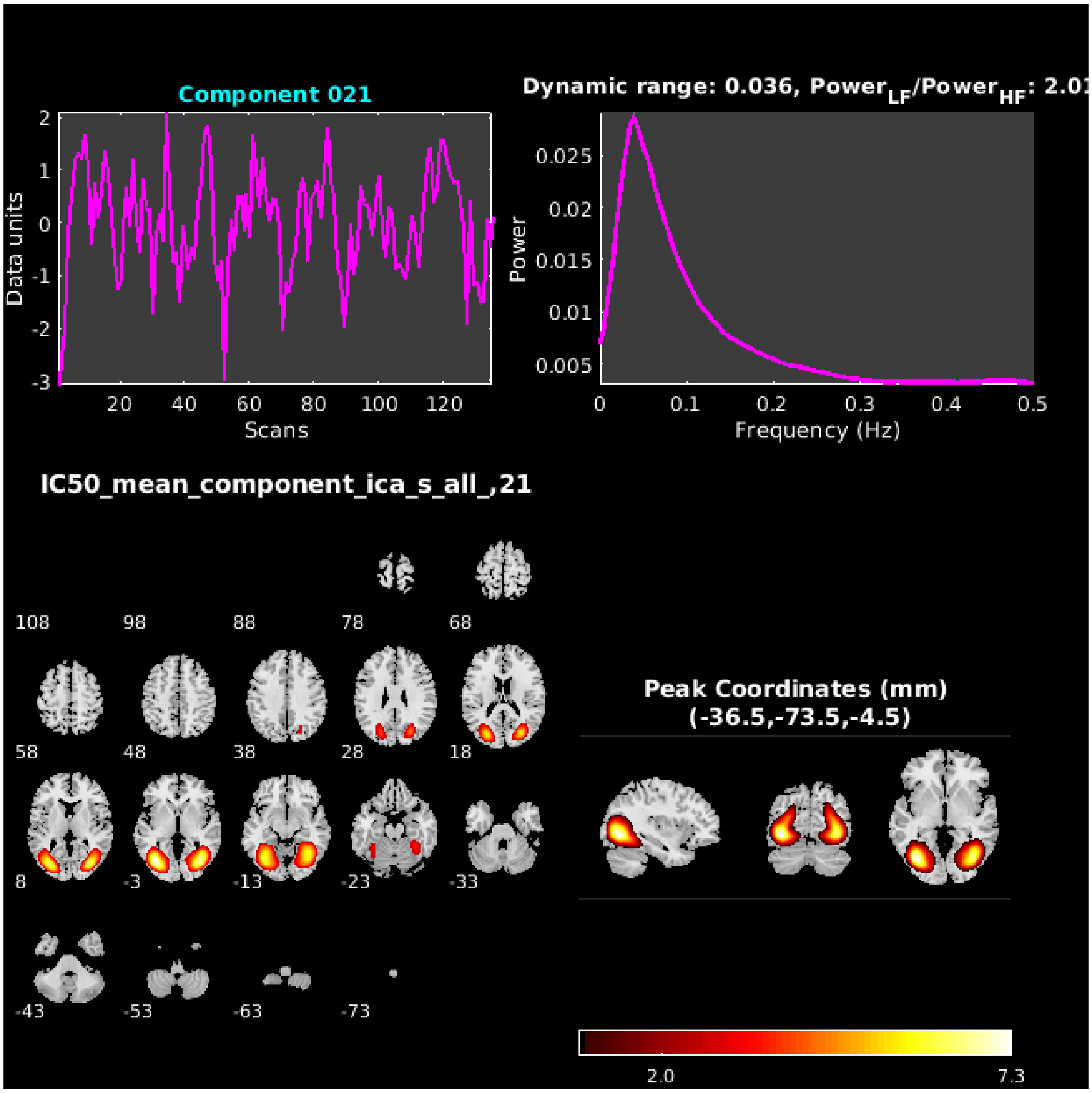

Component 021
Dynamic range: 0.036, Power$_{LF}$/Power$_{HF}$: 2.0
Data units
Scans
Power
Frequency (Hz)
IC50_mean_component_ica_s_all_,21
Peak Coordinates (mm)
(-36.5,-73.5,-4.5)
2.0
7.3

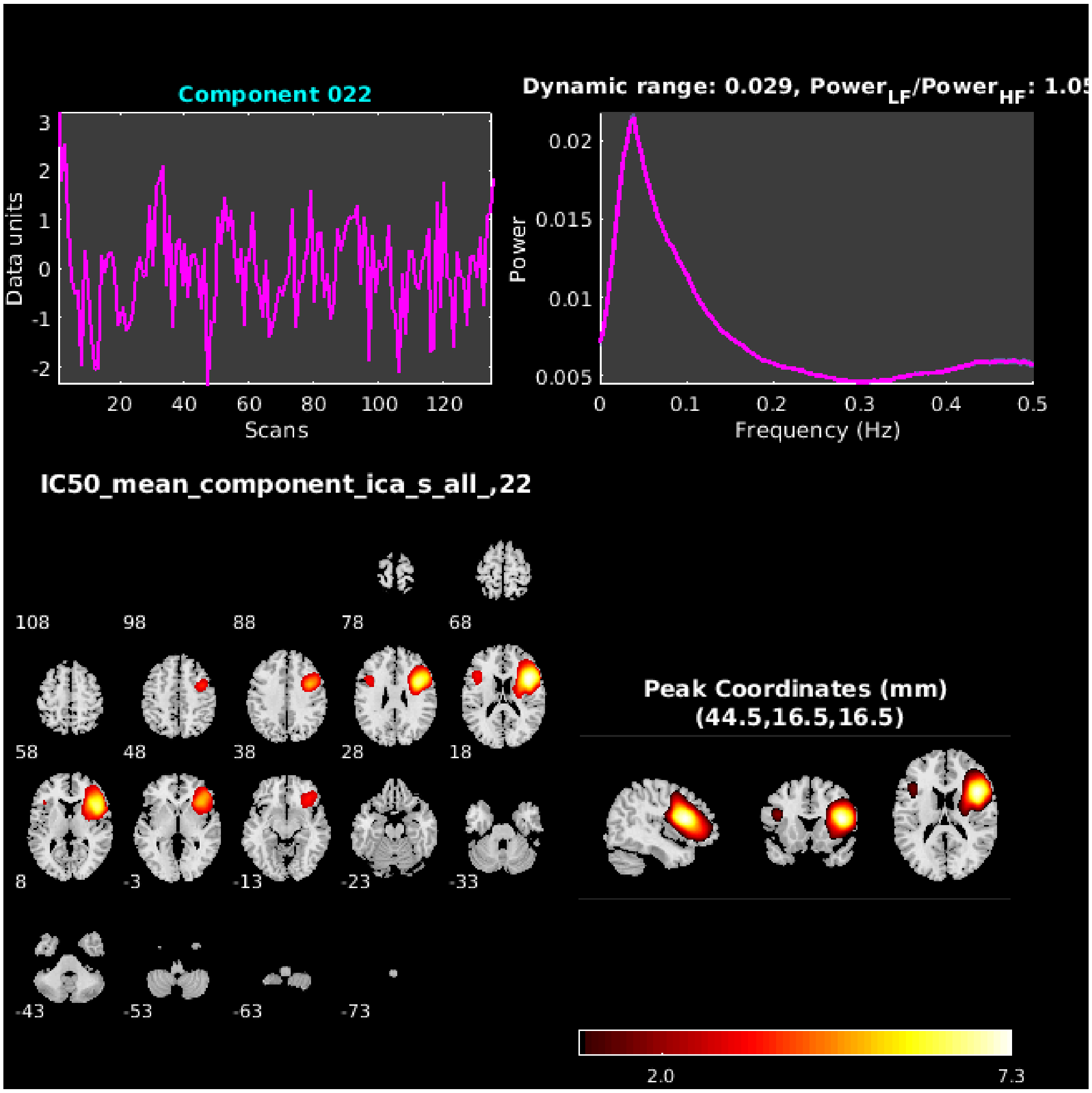

Component 022
Dynamic range: 0.029, Power$_{LF}$/Power$_{HF}$: 1.05
Data units
Scans
Power
Frequency (Hz)
IC50_mean_component_ica_s_all_,22
Peak Coordinates (mm)
(44.5,16.5,16.5)
2.0
7.3

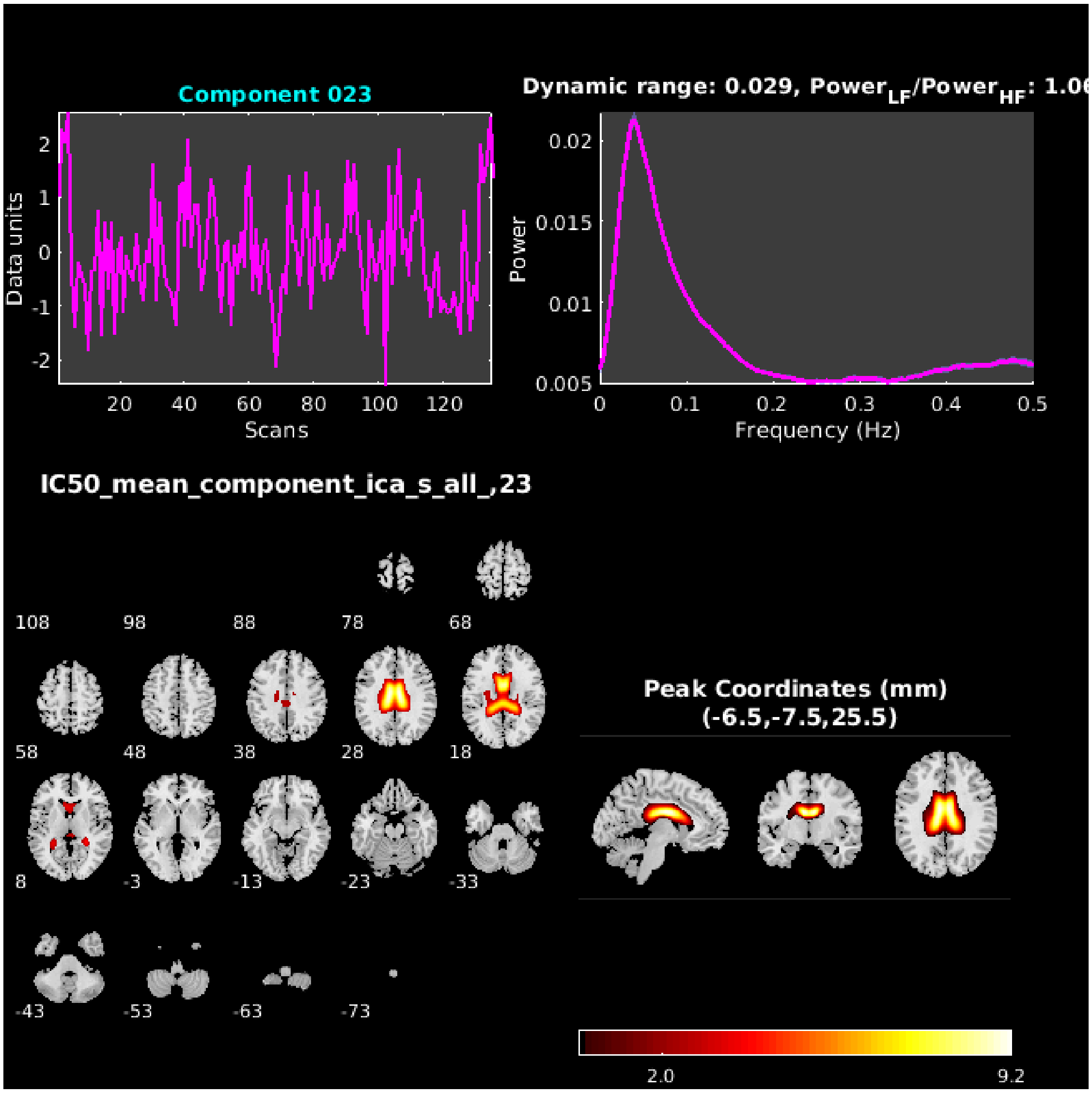
Component 023
Dynamic range: 0.029, Power_LF/Power_HF: 1.06
Data units
Scans
Power
Frequency (Hz)
IC50_mean_component_ica_s_all_,23
108
98
88
78
68
58
48
38
28
18
8
-3
-13
-23
-33
-43
-53
-63
-73
Peak Coordinates (mm)
(-6.5,-7.5,25.5)
2.0
9.2

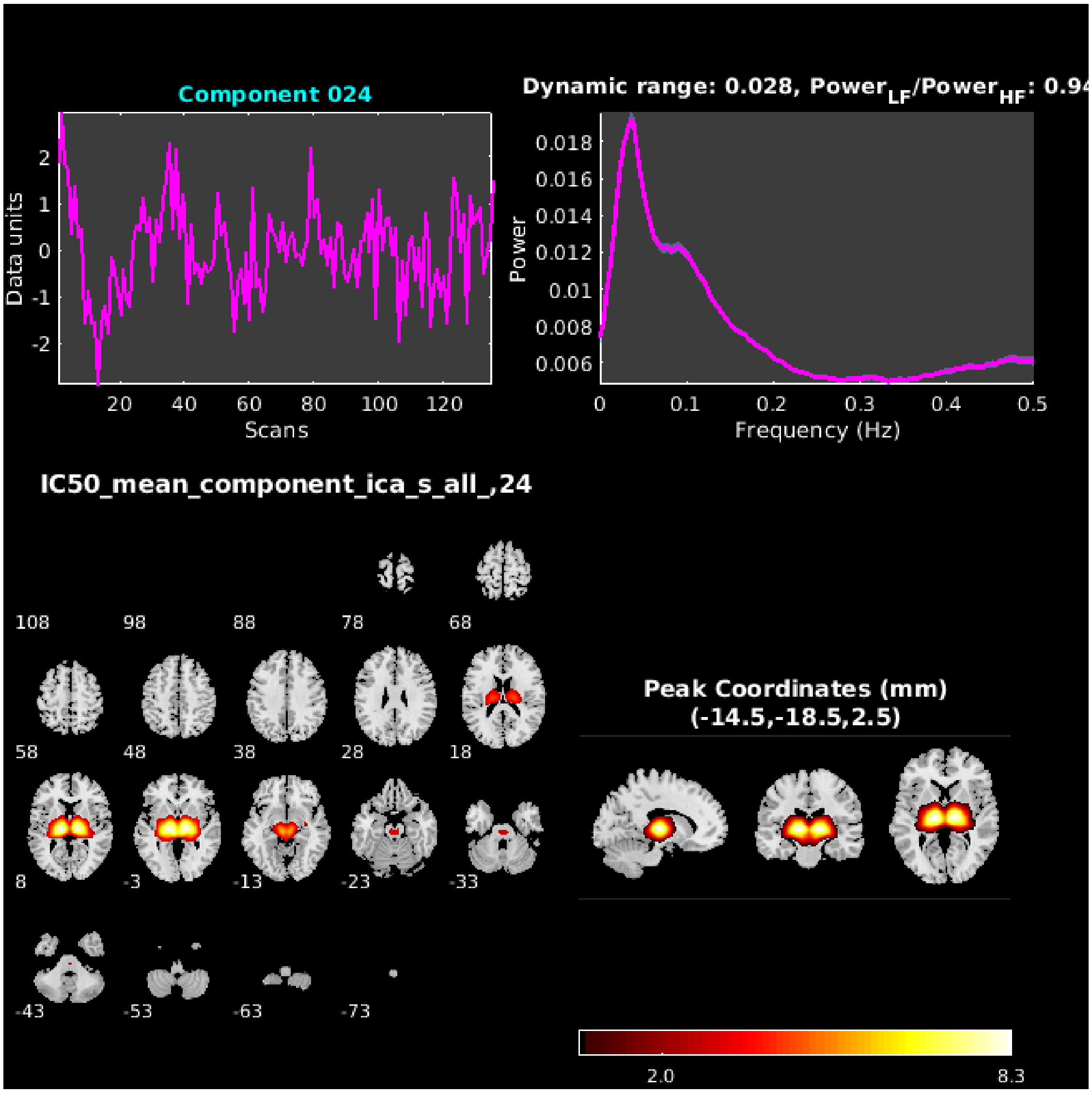
Component 024
Data units
Scans
Dynamic range: 0.028, Power_LF/Power_HF: 0.9
Power
Frequency (Hz)
IC50_mean_component_ica_s_all_,24
108
98
88
78
68
58
48
38
28
18
8
-3
-13
-23
-33
-43
-53
-63
-73
Peak Coordinates (mm)
(-14.5,-18.5,2.5)
2.0
8.3

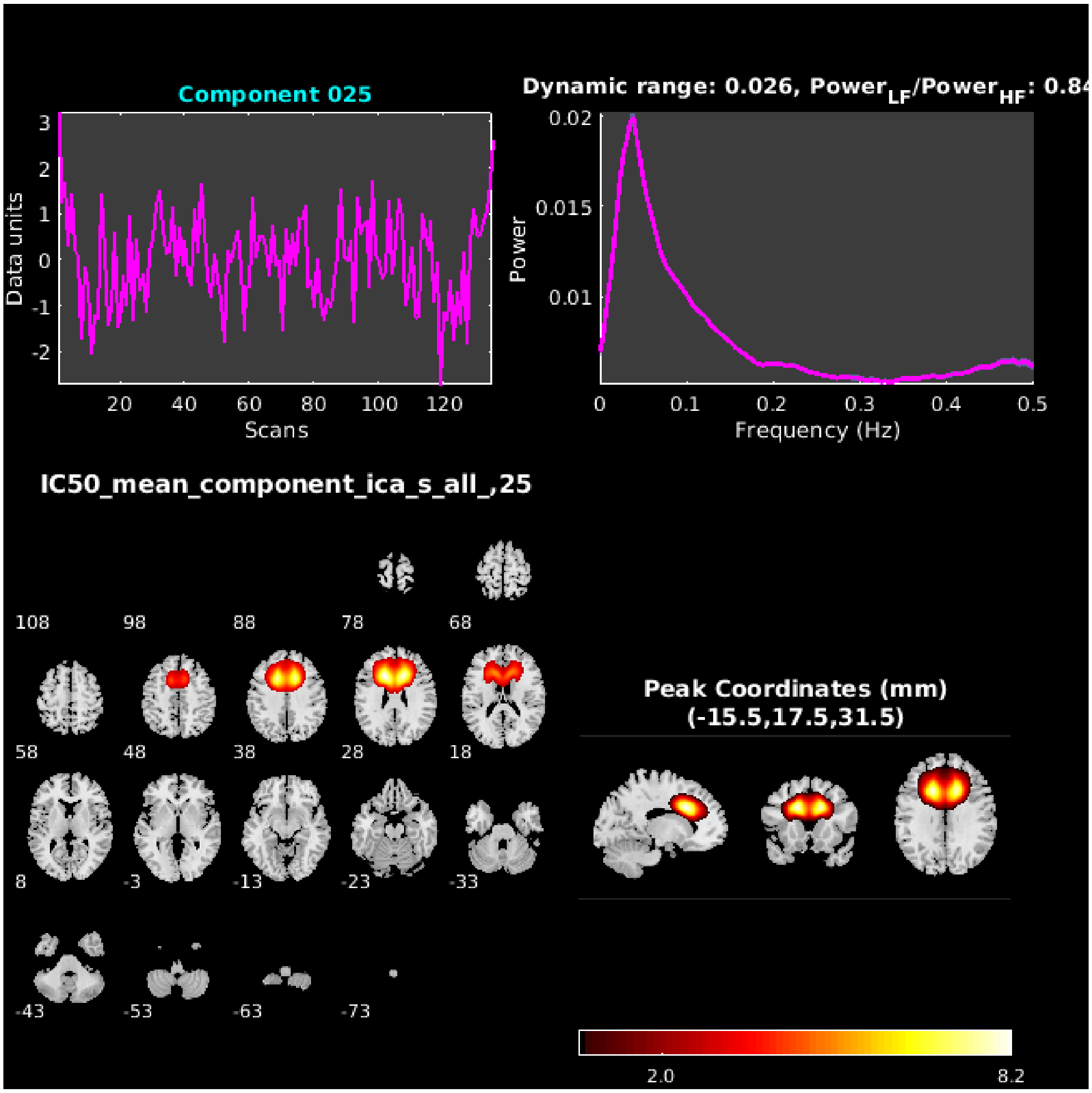

Component 025
Dynamic range: 0.026, Power_LF/Power_HF: 0.84
Data units
Scans
Power
Frequency (Hz)
IC50_mean_component_ica_s_all_,25
108
98
88
78
68
58
48
38
28
18
8
-3
-13
-23
-33
-43
-53
-63
-73
Peak Coordinates (mm)
(-15.5,17.5,31.5)
2.0
8.2

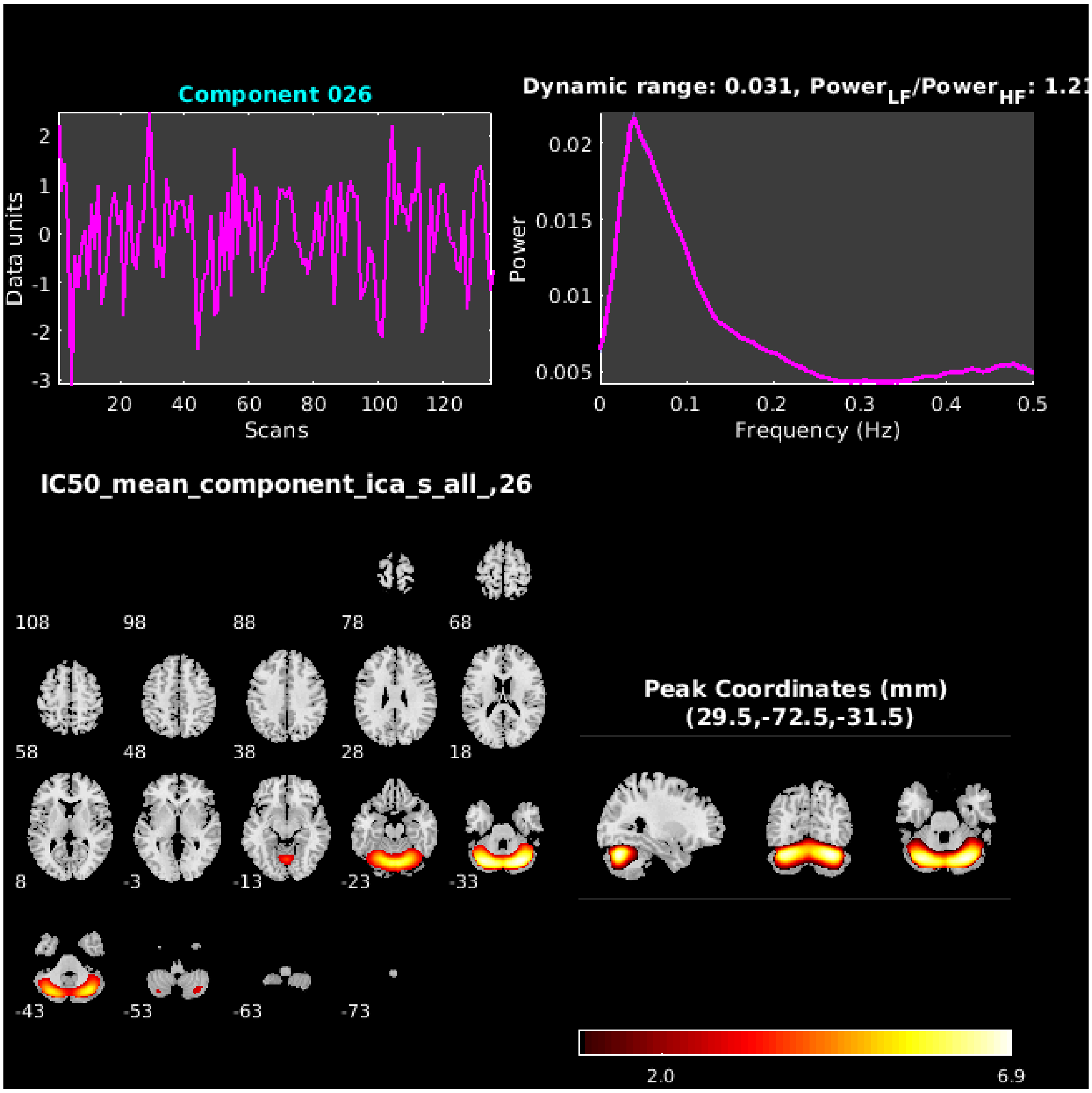

Component 026
Dynamic range: 0.031, Power$_{LF}$/Power$_{HF}$: 1.21
Data units
Scans
Power
Frequency (Hz)
IC50_mean_component_ica_s_all_,26
Peak Coordinates (mm)
(29.5,-72.5,-31.5)
2.0
6.9

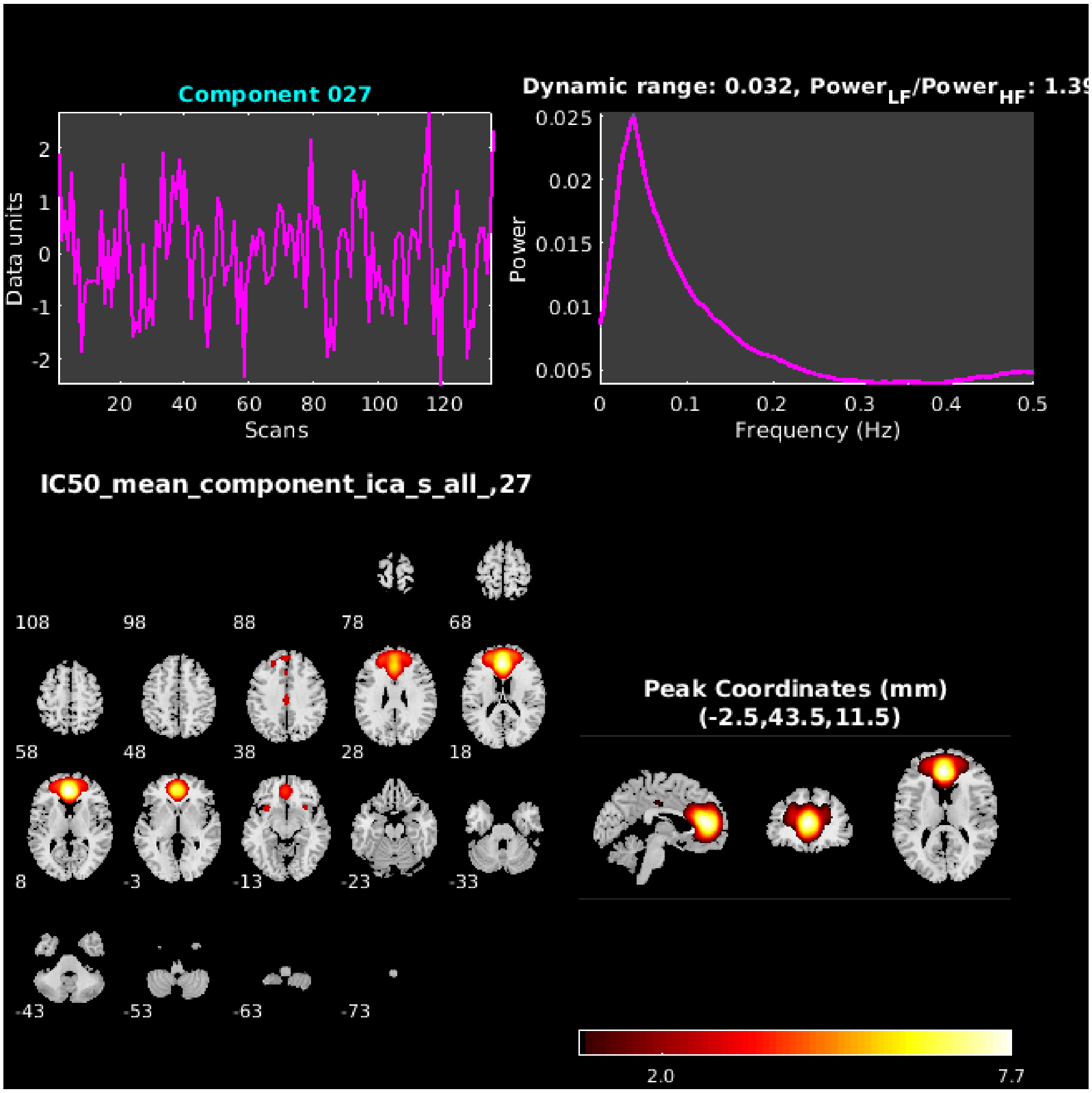

Component 027
Dynamic range: 0.032, Power$_{LF}$/Power$_{HF}$: 1.39
Data units
Scans
Power
Frequency (Hz)
IC50_mean_component_ica_s_all_,27
108
98
88
78
68
58
48
38
28
18
8
-3
-13
-23
-33
-43
-53
-63
-73
Peak Coordinates (mm)
(-2.5,43.5,11.5)
2.0
7.7

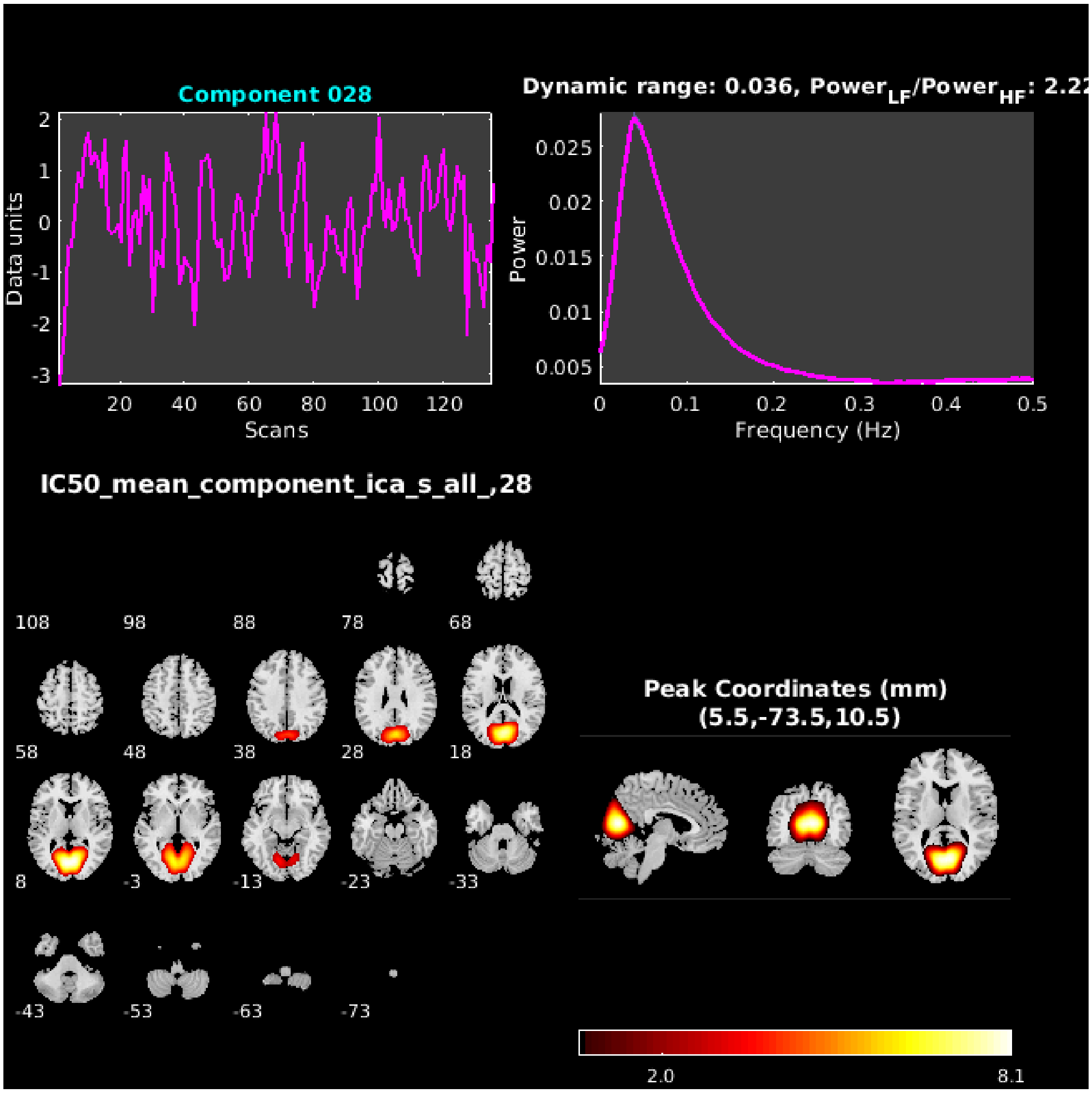

Component 028
Dynamic range: 0.036, Power$_{LF}$/Power$_{HF}$: 2.22
Data units
Scans
Power
Frequency (Hz)
IC50_mean_component_ica_s_all_,28
Peak Coordinates (mm)
(5.5,-73.5,10.5)

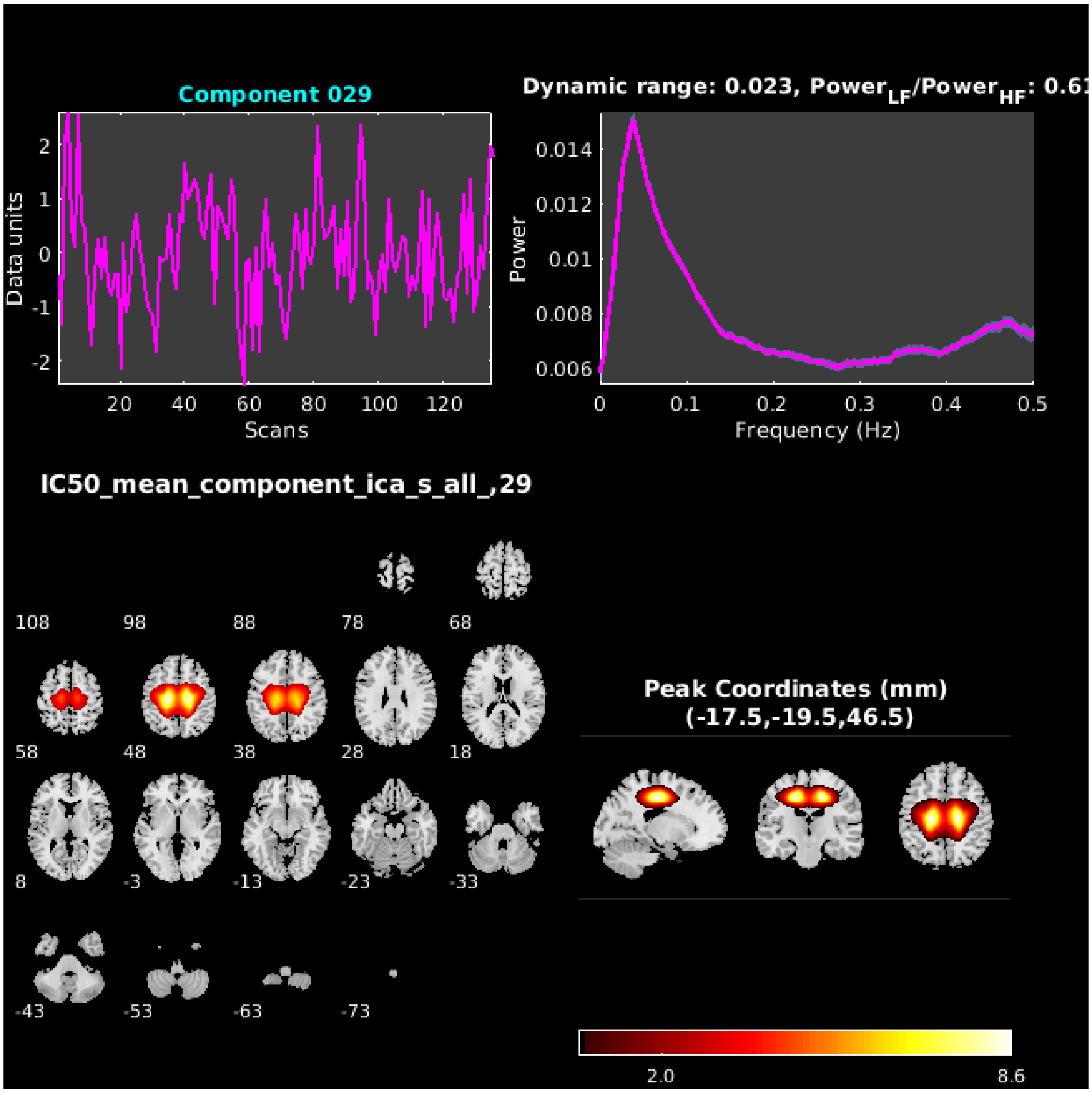
Component 029
Dynamic range: 0.023, Power$_{LF}$/Power$_{HF}$: 0.61
Data units
Scans
Power
Frequency (Hz)
IC50_mean_component_ica_s_all_,29
108
98
88
78
68
58
48
38
28
18
8
-3
-13
-23
-33
-43
-53
-63
-73
Peak Coordinates (mm)
(-17.5,-19.5,46.5)
2.0
8.6

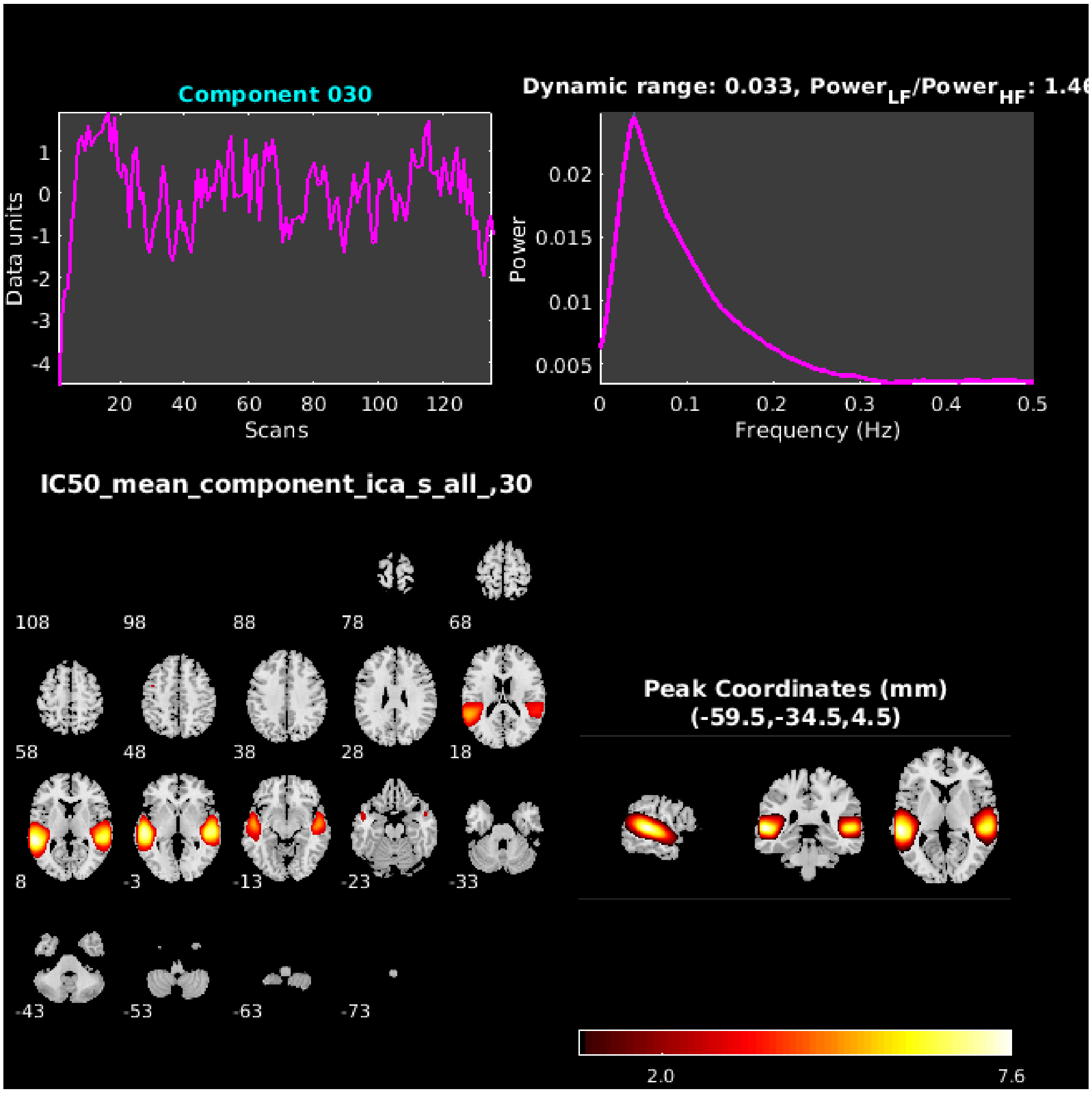

Component 030
Dynamic range: 0.033, Power_LF/Power_HF: 1.46
Data units
Scans
Power
Frequency (Hz)
IC50_mean_component_ica_s_all_,30
Peak Coordinates (mm)
(-59.5,-34.5,4.5)
2.0
7.6

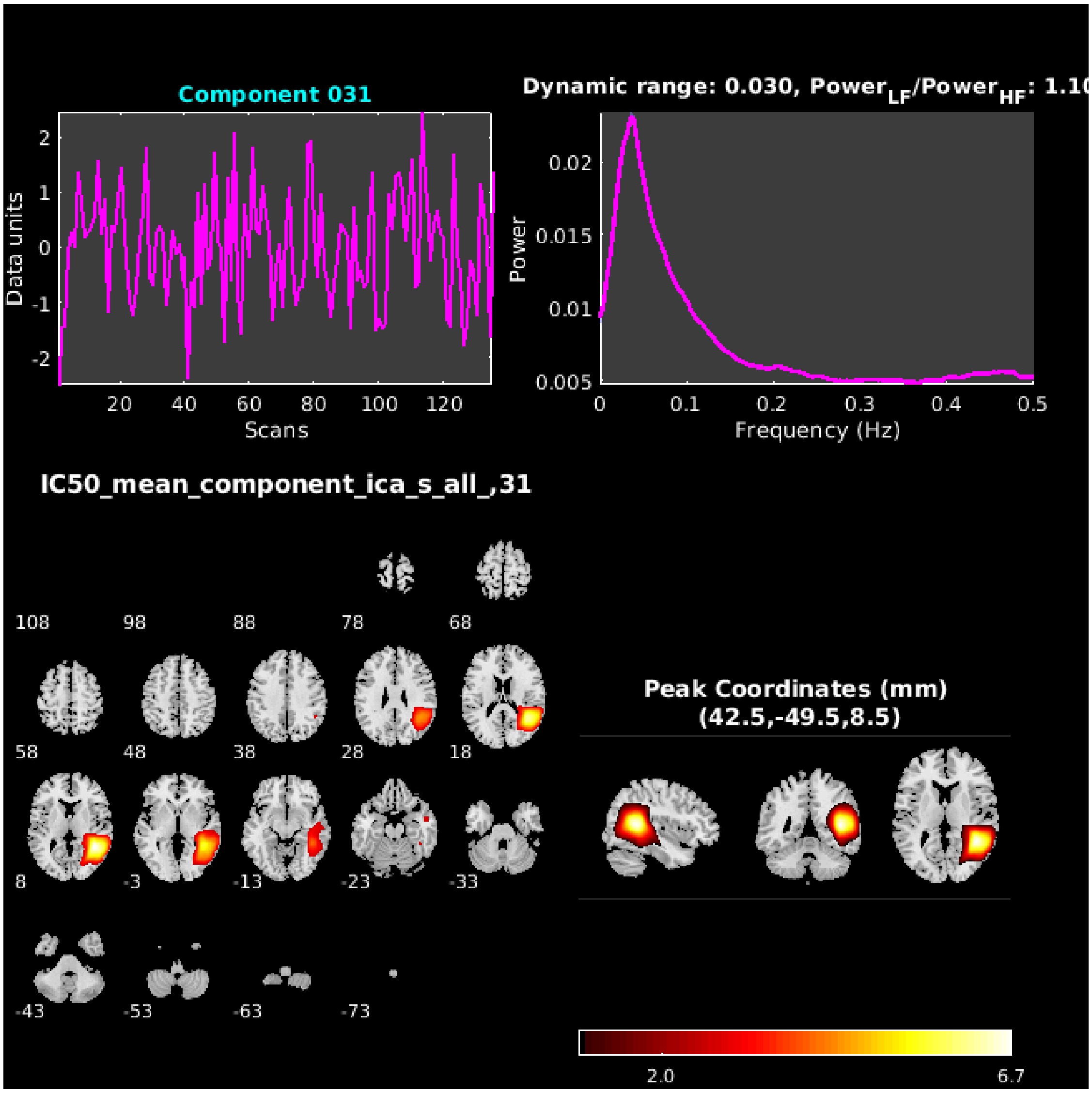

Component 031
Dynamic range: 0.030, Power$_{LF}$/Power$_{HF}$: 1.1
Data units
Scans
Power
Frequency (Hz)
IC50_mean_component_ica_s_all_,31
108
98
88
78
68
58
48
38
28
18
8
-3
-13
-23
-33
-43
-53
-63
-73
Peak Coordinates (mm)
(42.5,-49.5,8.5)
2.0
6.7

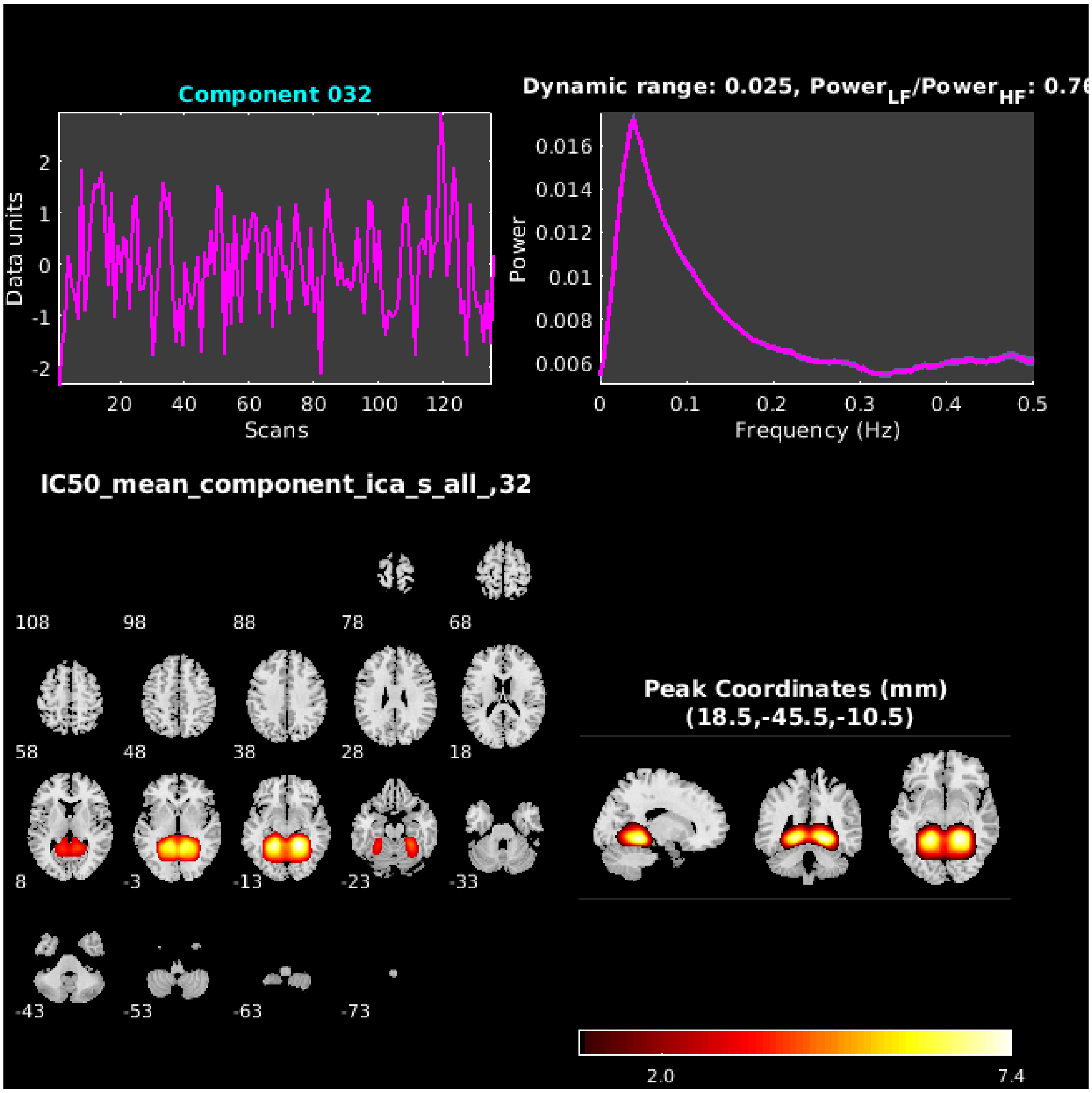

Component 032
Dynamic range: 0.025, Power$_{LF}$/Power$_{HF}$: 0.7
Data units
Scans
Power
Frequency (Hz)
IC50_mean_component_ica_s_all_,32
Peak Coordinates (mm)
(18.5,-45.5,-10.5)
2.0
7.4

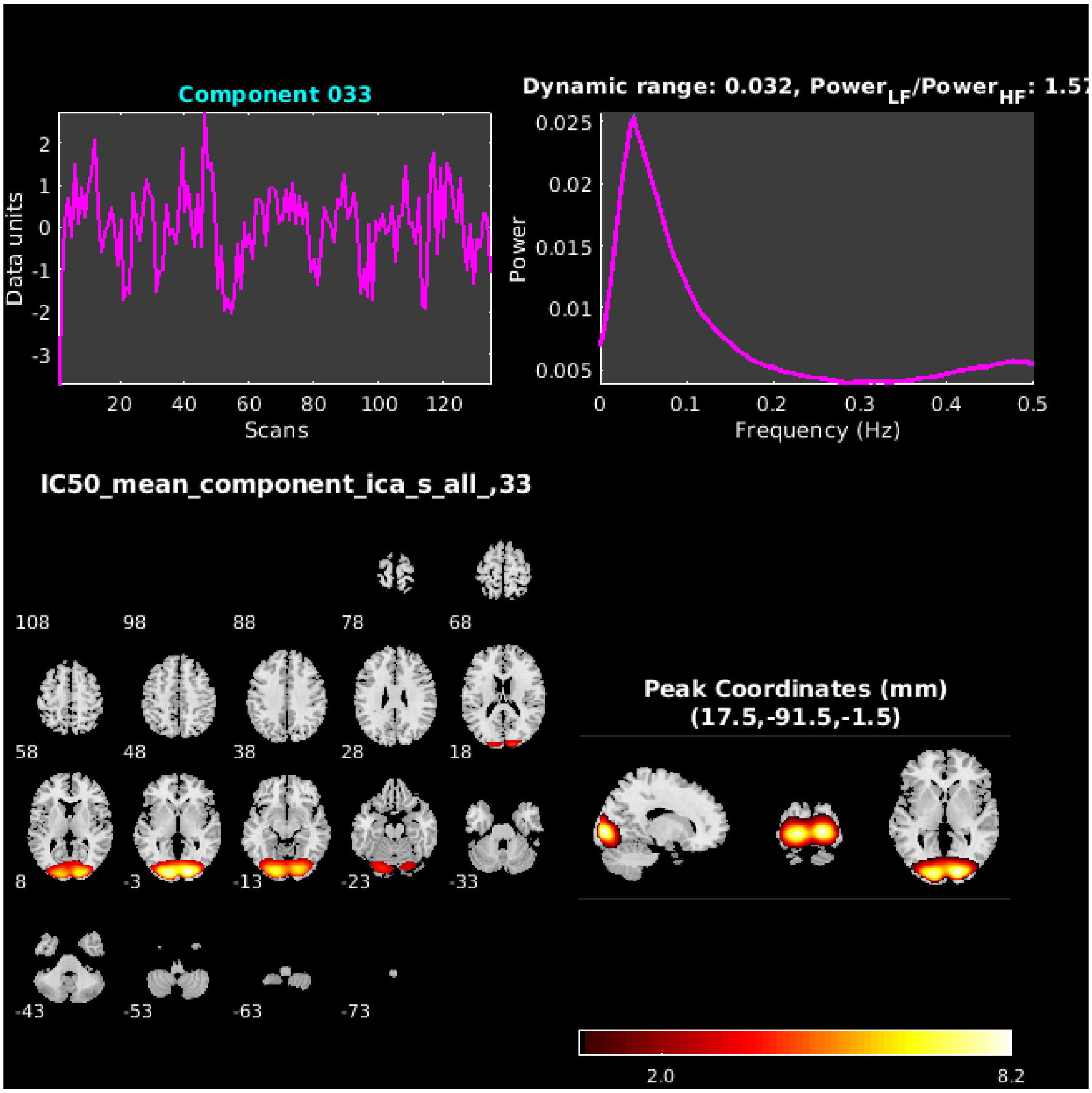
Component 033
Dynamic range: 0.032, Power$_{LF}$/Power$_{HF}$: 1.57
Data units
Scans
Power
Frequency (Hz)
IC50_mean_component_ica_s_all_,33
108
98
88
78
68
58
48
38
28
18
8
-3
-13
-23
-33
-43
-53
-63
-73
Peak Coordinates (mm)
(17.5,-91.5,-1.5)
2.0
8.2

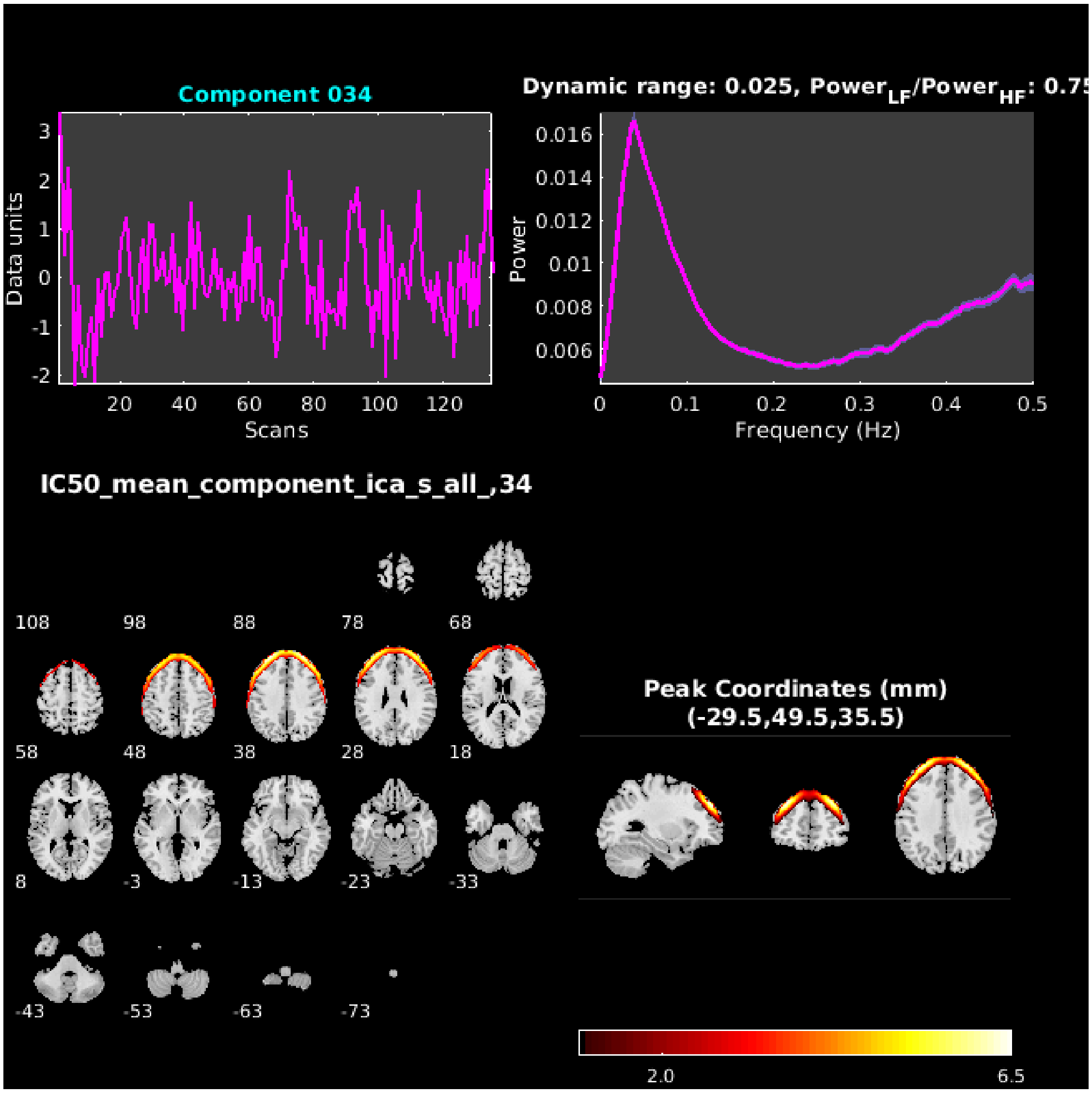

Component 034
Dynamic range: 0.025, Power_LF/Power_HF: 0.75
Data units
Scans
Power
Frequency (Hz)
IC50_mean_component_ica_s_all_,34
108
98
88
78
68
58
48
38
28
18
8
-3
-13
-23
-33
-43
-53
-63
-73
Peak Coordinates (mm)
(-29.5,49.5,35.5)
2.0
6.5

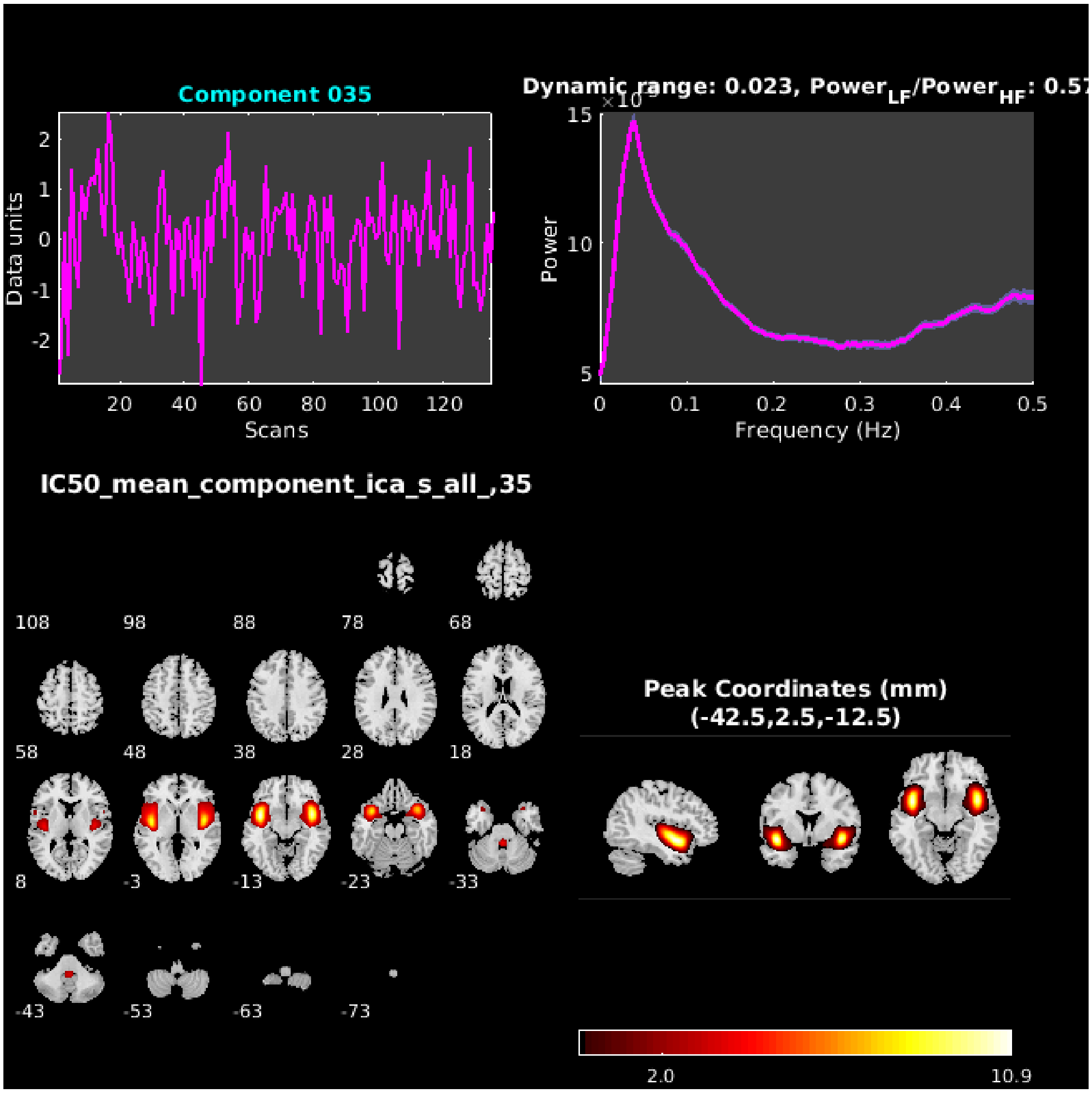
Component 035
Dynamic range: 0.023, Power$_{LF}$/Power$_{HF}$: 0.57
Data units
Scans
Power
Frequency (Hz)
IC50_mean_component_ica_s_all_,35
Peak Coordinates (mm)
(-42.5,2.5,-12.5)
2.0
10.9

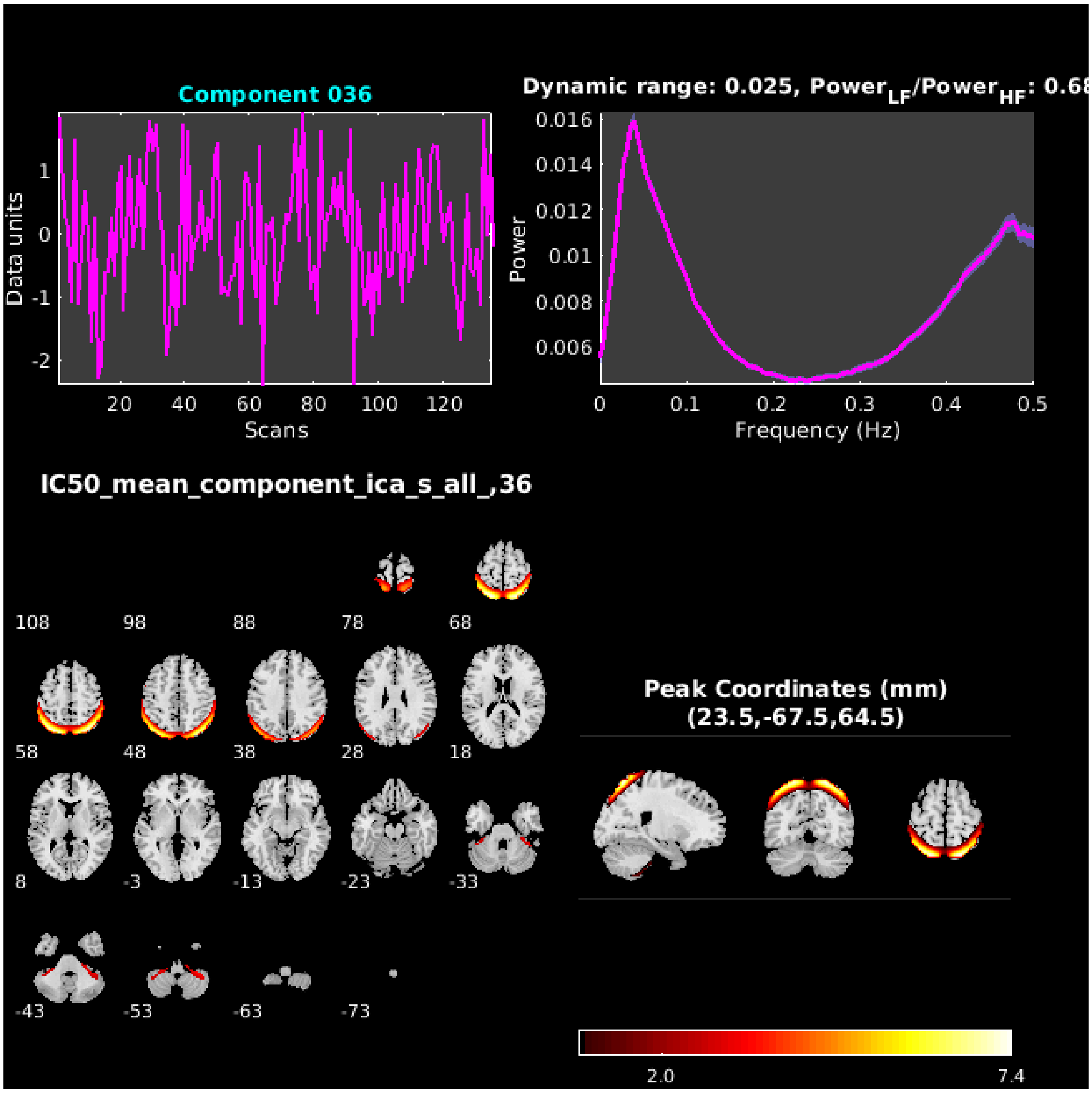

Component 036
Dynamic range: 0.025, Power_LF/Power_HF: 0.68
Data units
Scans
Power
Frequency (Hz)
IC50_mean_component_ica_s_all_,36
Peak Coordinates (mm)
(23.5,-67.5,64.5)
2.0
7.4

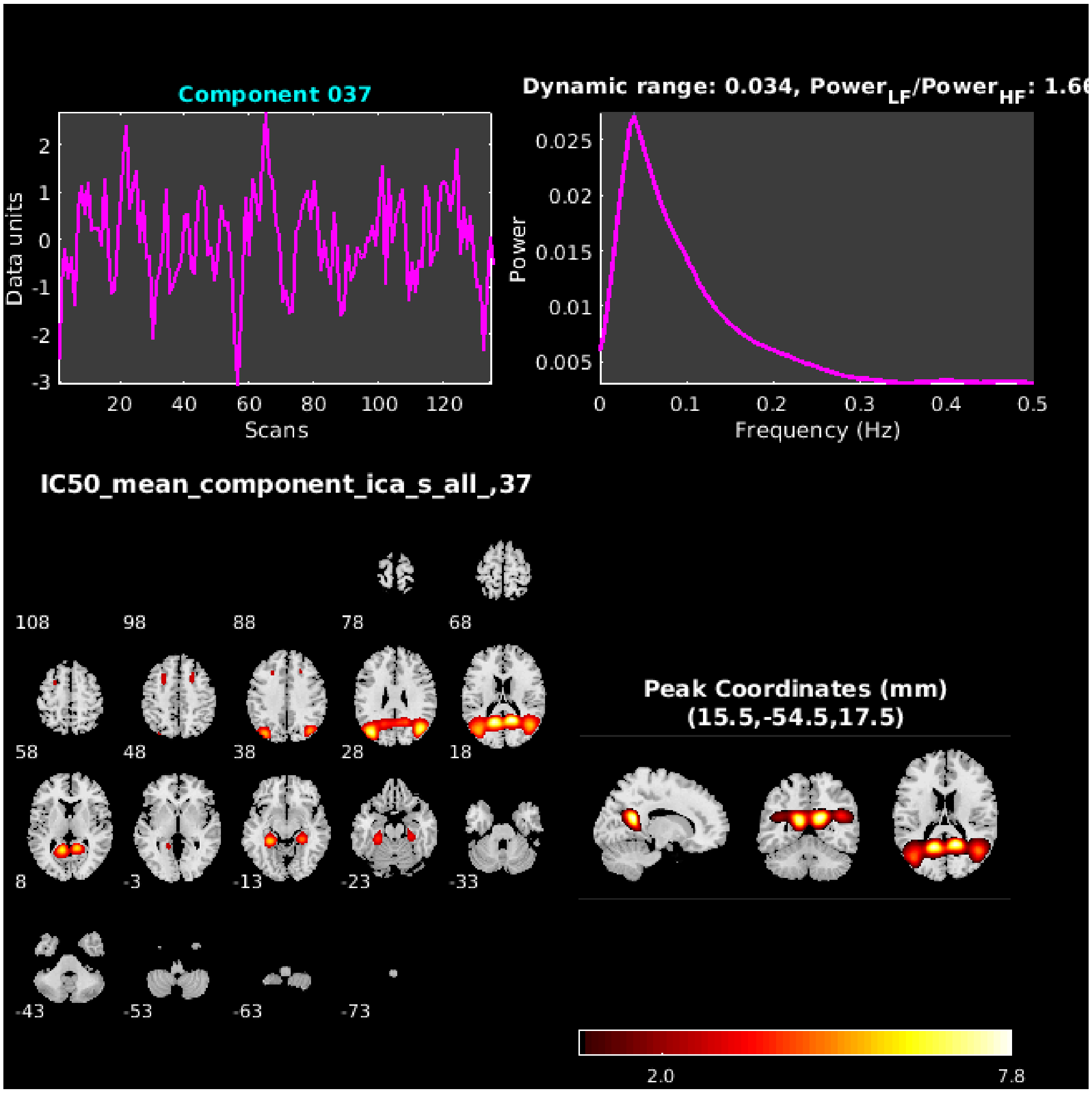
Component 037
Dynamic range: 0.034, Power_LF/Power_HF: 1.66
Data units
Scans
Power
Frequency (Hz)
IC50_mean_component_ica_s_all_,37
108
98
88
78
68
58
48
38
28
18
8
-3
-13
-23
-33
-43
-53
-63
-73
Peak Coordinates (mm)
(15.5,-54.5,17.5)
2.0
7.8

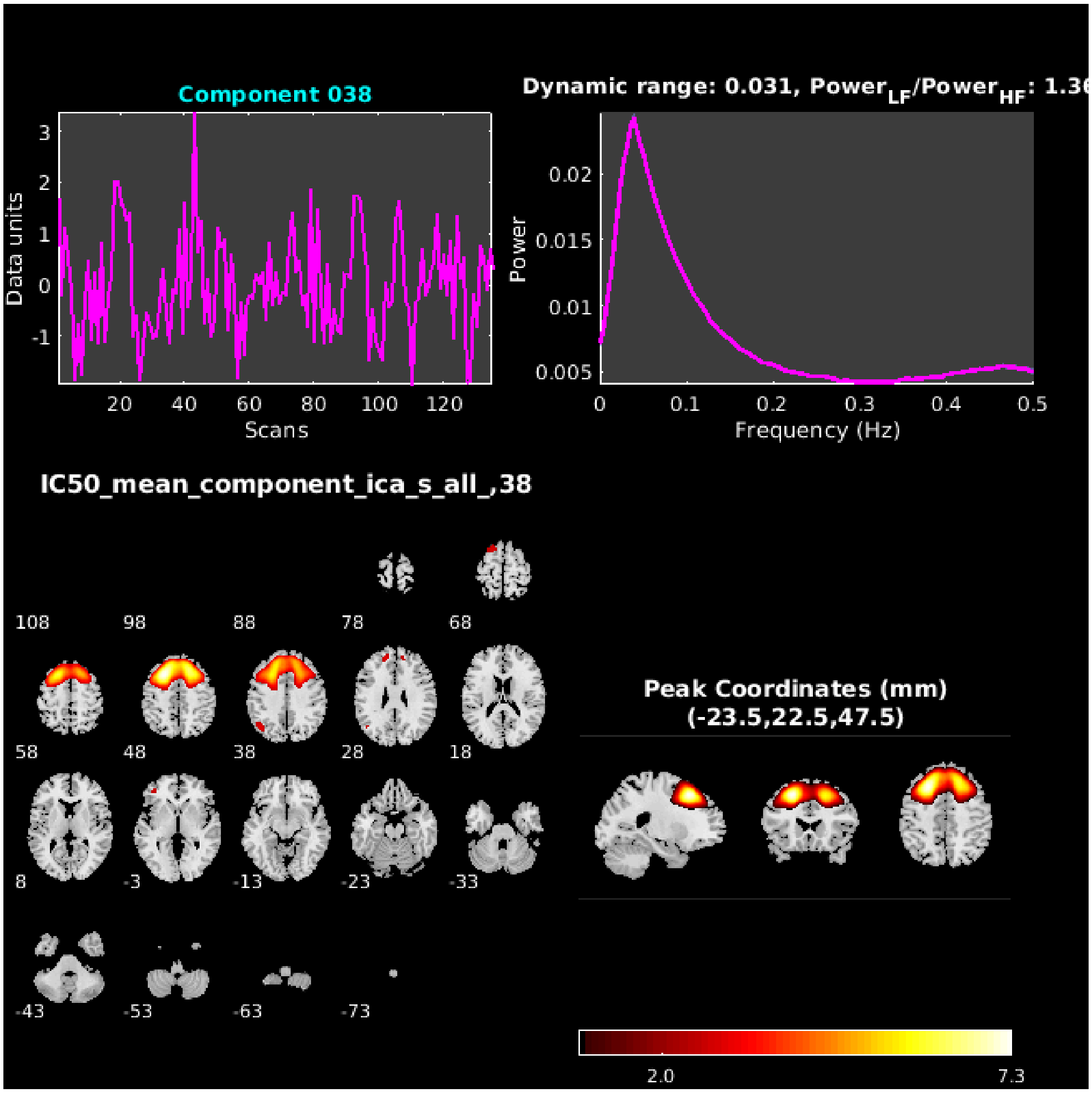
Component 038
Dynamic range: 0.031, Power$_{LF}$/Power$_{HF}$: 1.36
Data units
Scans
Power
Frequency (Hz)
IC50_mean_component_ica_s_all_,38
Peak Coordinates (mm)
(-23.5,22.5,47.5)

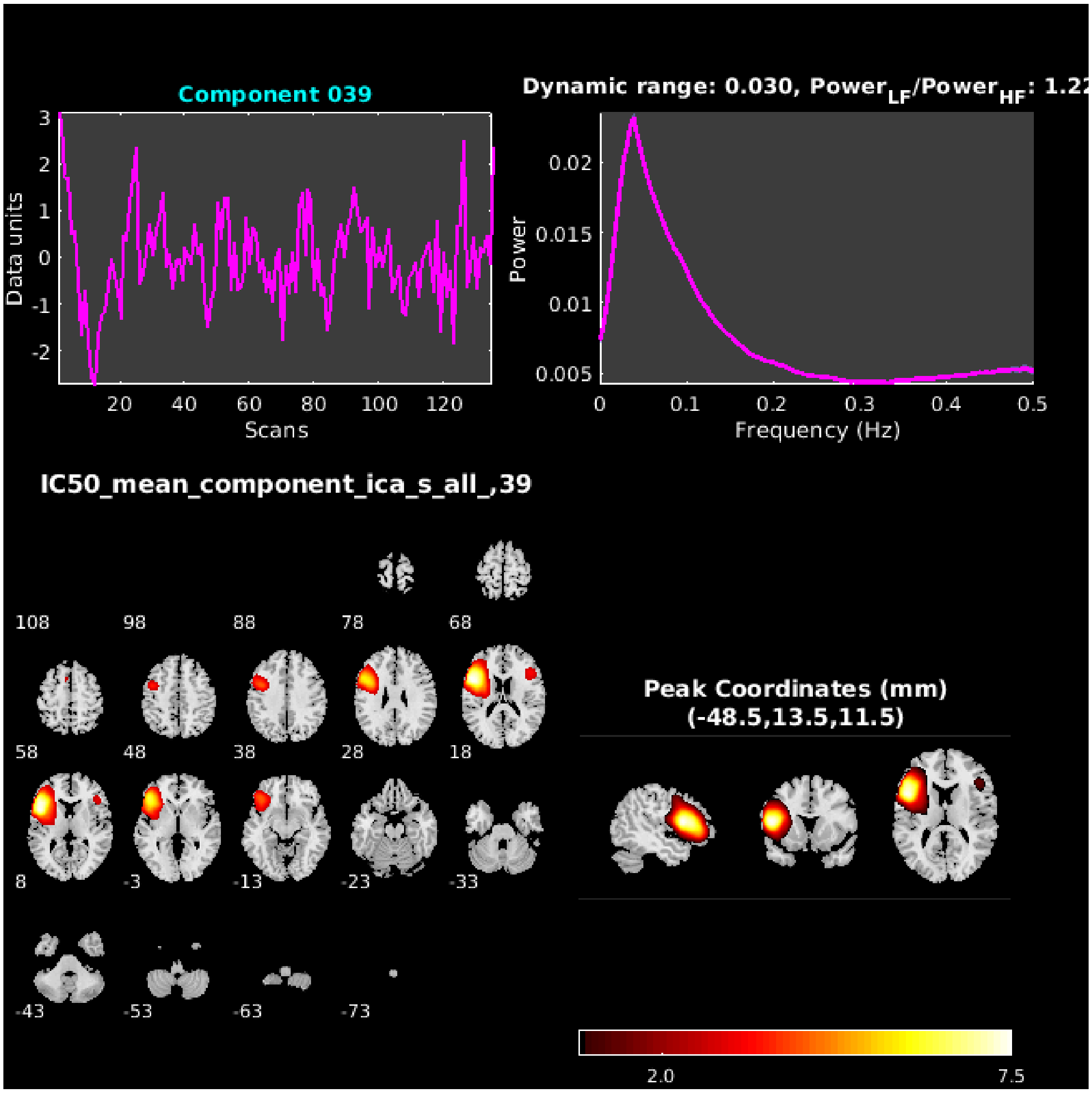
Component 039
Dynamic range: 0.030, Power_LF/Power_HF: 1.22
Data units
Scans
Power
Frequency (Hz)
IC50_mean_component_ica_s_all_,39
108 98 88 78 68
58 48 38 28 18
8 -3 -13 -23 -33
-43 -53 -63 -73
Peak Coordinates (mm)
(-48.5,13.5,11.5)
2.0
7.5

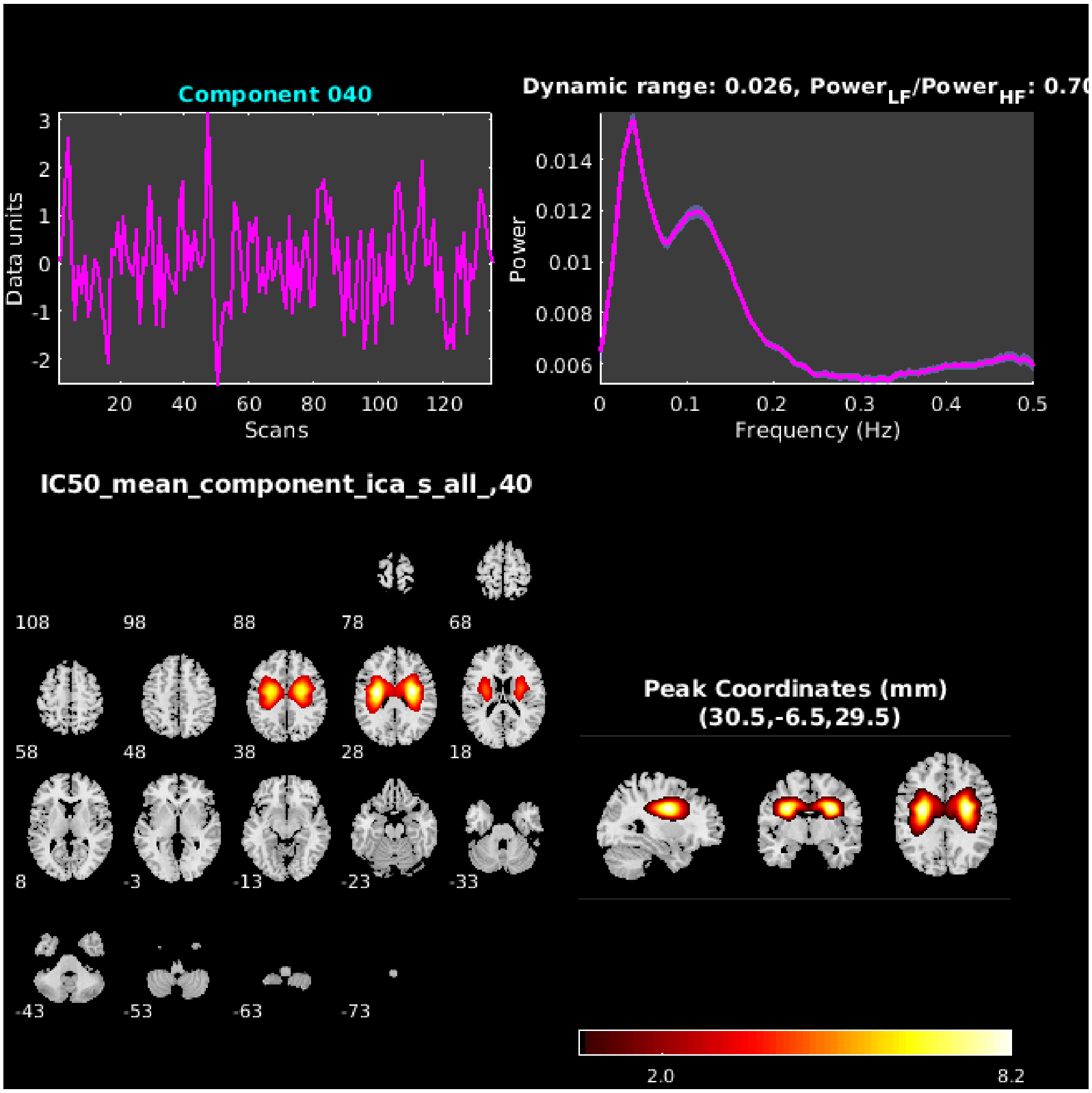

Component 040
Dynamic range: 0.026, Power$_{LF}$/Power$_{HF}$: 0.70
Data units
Scans
Power
Frequency (Hz)
IC50_mean_component_ica_s_all_,40
Peak Coordinates (mm)
(30.5,-6.5,29.5)
2.0
8.2

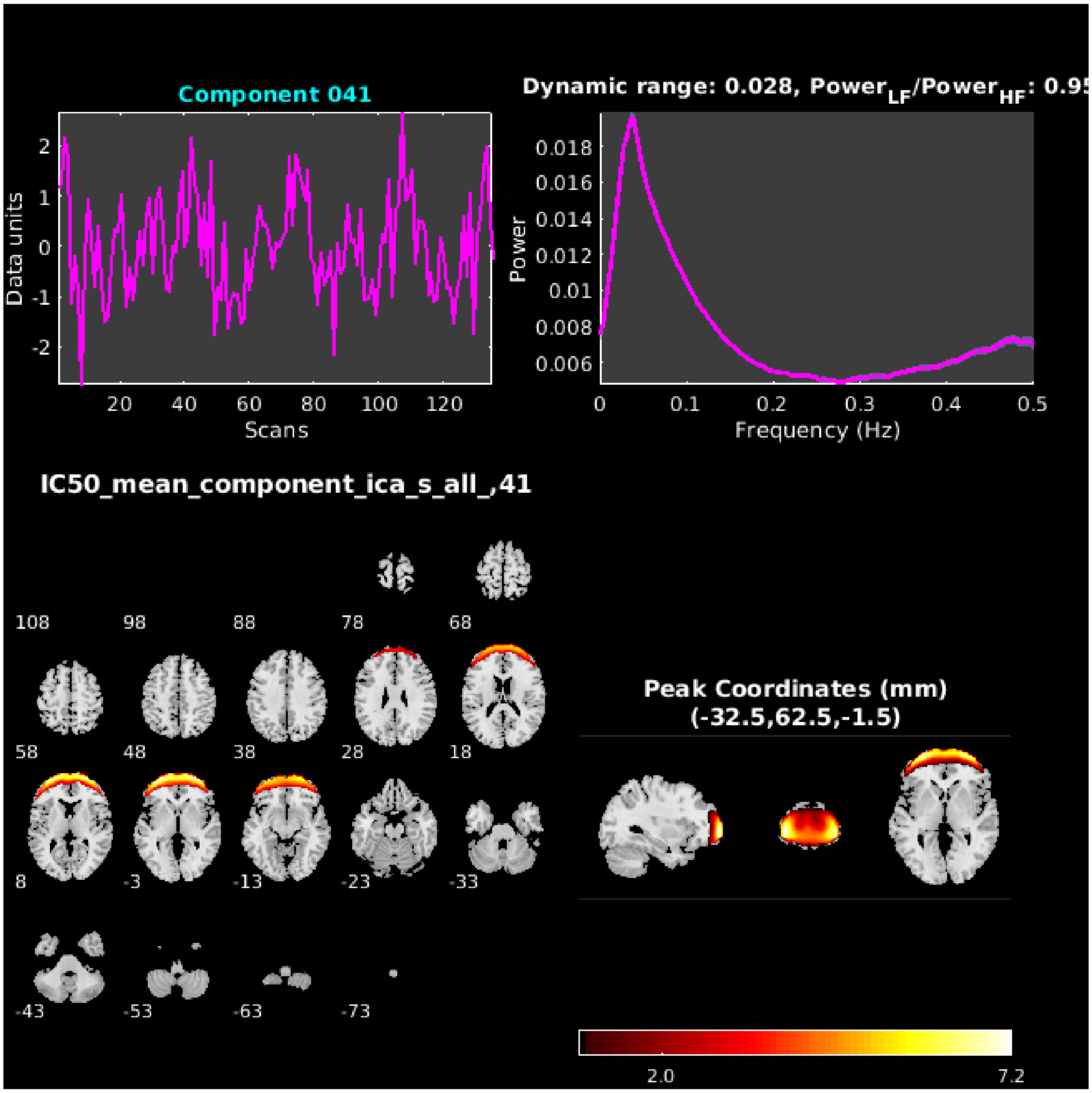
Component 041
Dynamic range: 0.028, Power$_{LF}$/Power$_{HF}$: 0.95
Data units
Scans
Power
Frequency (Hz)
IC50_mean_component_ica_s_all_,41
108
98
88
78
68
58
48
38
28
18
8
-3
-13
-23
-33
-43
-53
-63
-73
Peak Coordinates (mm)
(-32.5,62.5,-1.5)
2.0
7.2

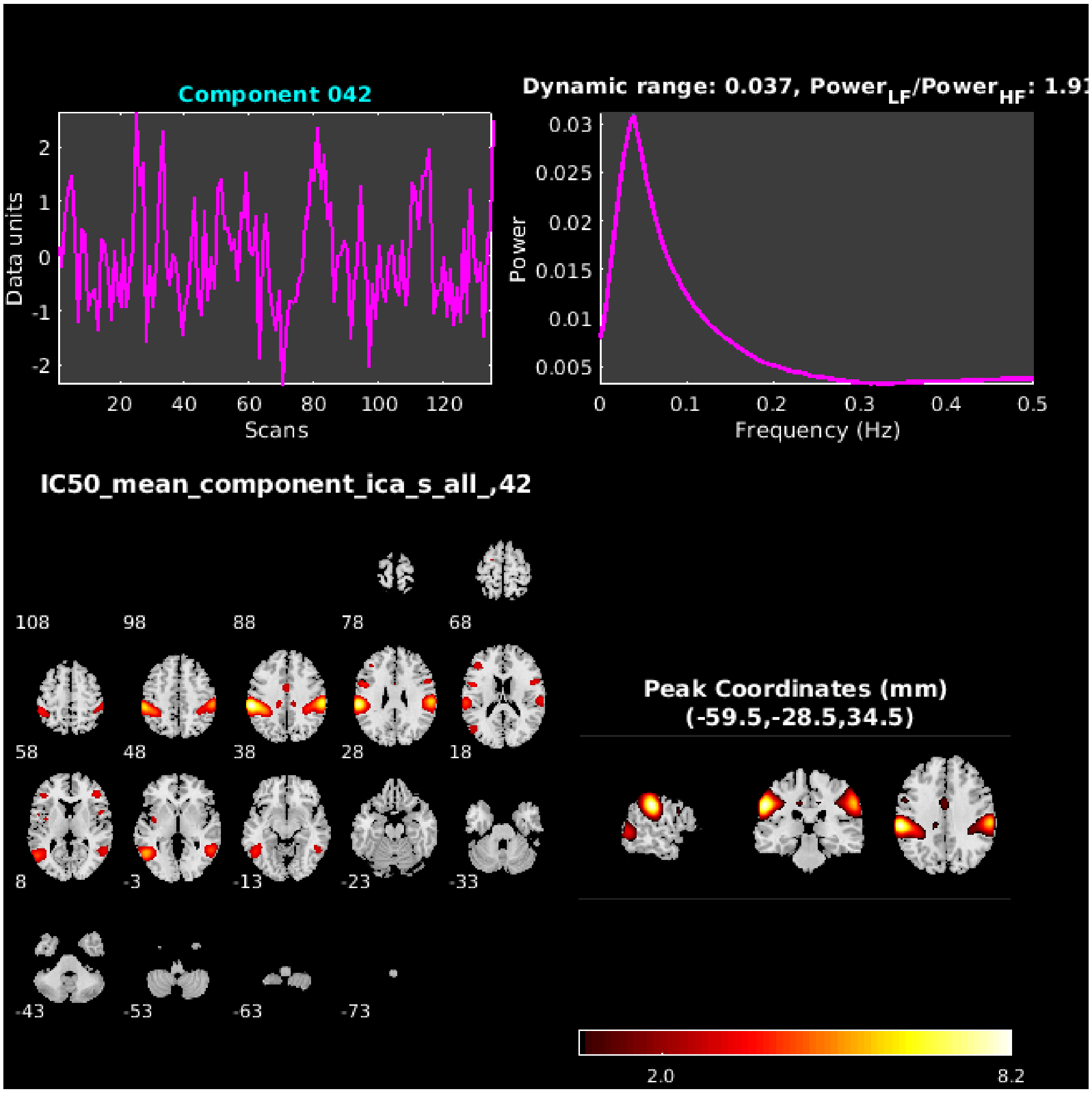

Component 042
Dynamic range: 0.037, Power$_{LF}$/Power$_{HF}$: 1.91
Data units
Scans
Power
Frequency (Hz)
IC50_mean_component_ica_s_all_,42
Peak Coordinates (mm)
(-59.5,-28.5,34.5)
2.0
8.2

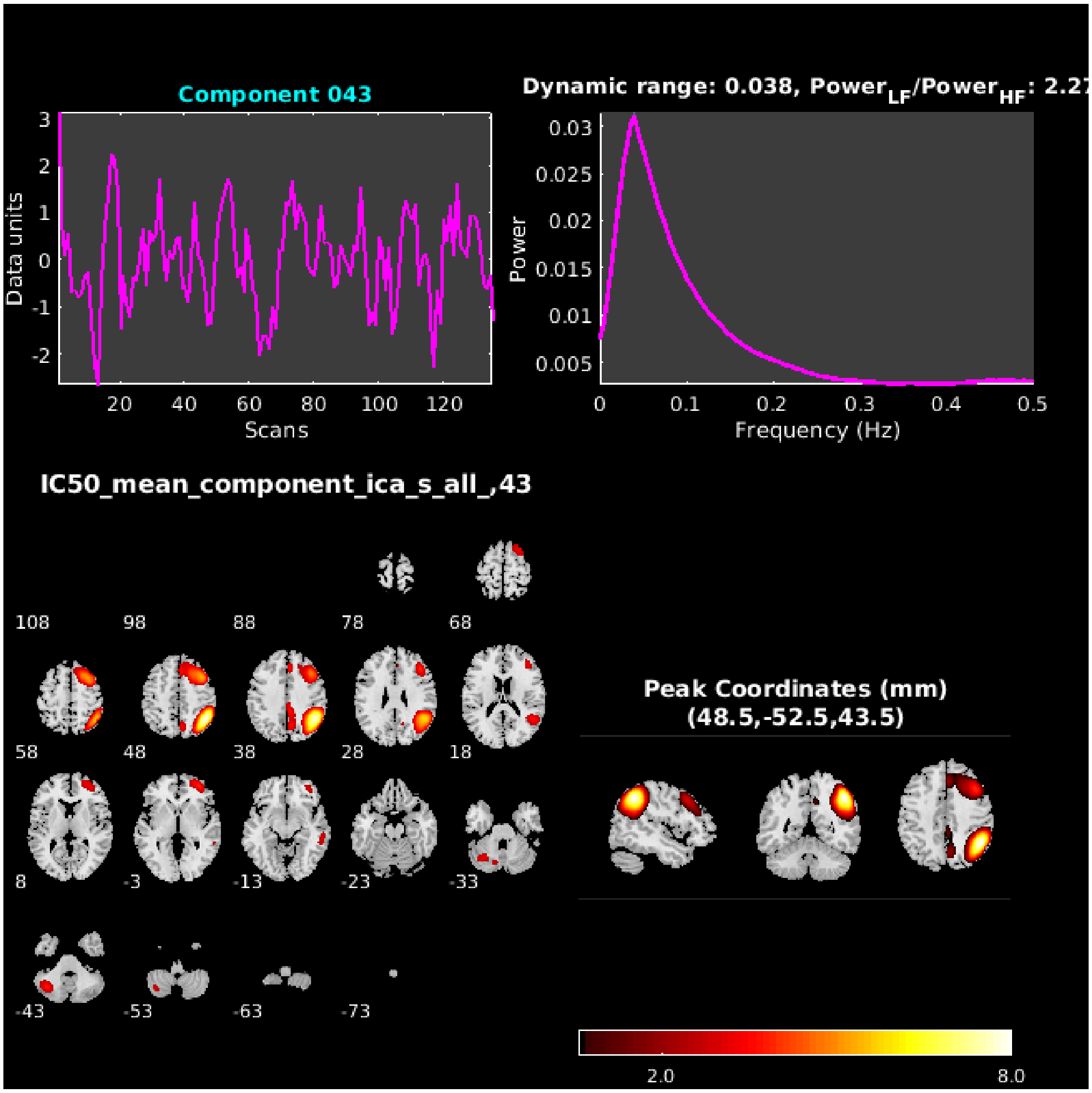

Component 043
Dynamic range: 0.038, Power$_{LF}$/Power$_{HF}$: 2.27
Data units
Scans
Power
Frequency (Hz)
IC50_mean_component_ica_s_all_,43
108
98
88
78
68
58
48
38
28
18
8
-3
-13
-23
-33
-43
-53
-63
-73
Peak Coordinates (mm)
(48.5,-52.5,43.5)
2.0
8.0

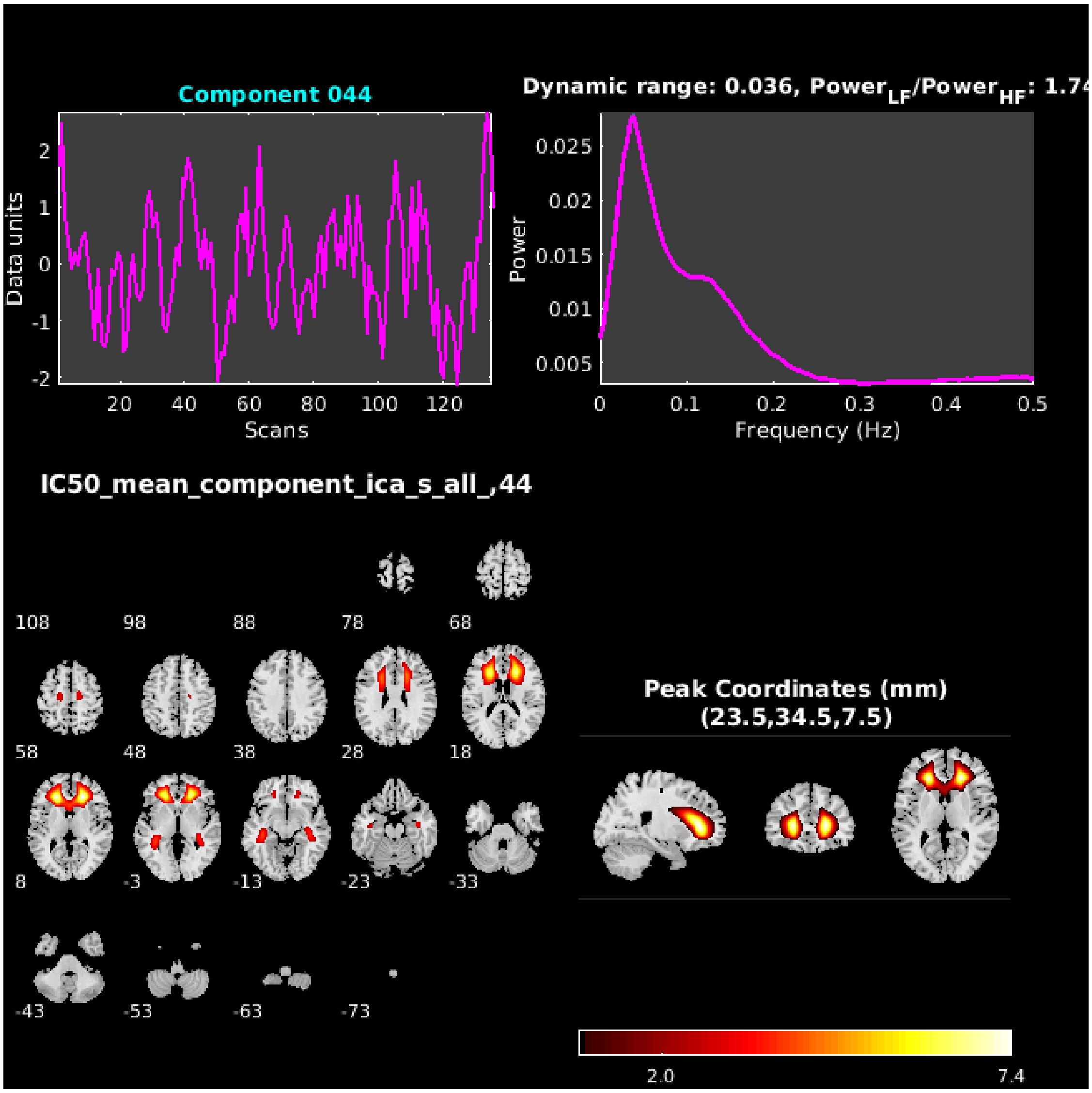
Component 044
Dynamic range: 0.036, Power_LF/Power_HF: 1.74
Data units
Scans
Power
Frequency (Hz)
IC50_mean_component_ica_s_all_,44
Peak Coordinates (mm)
(23.5,34.5,7.5)
2.0
7.4

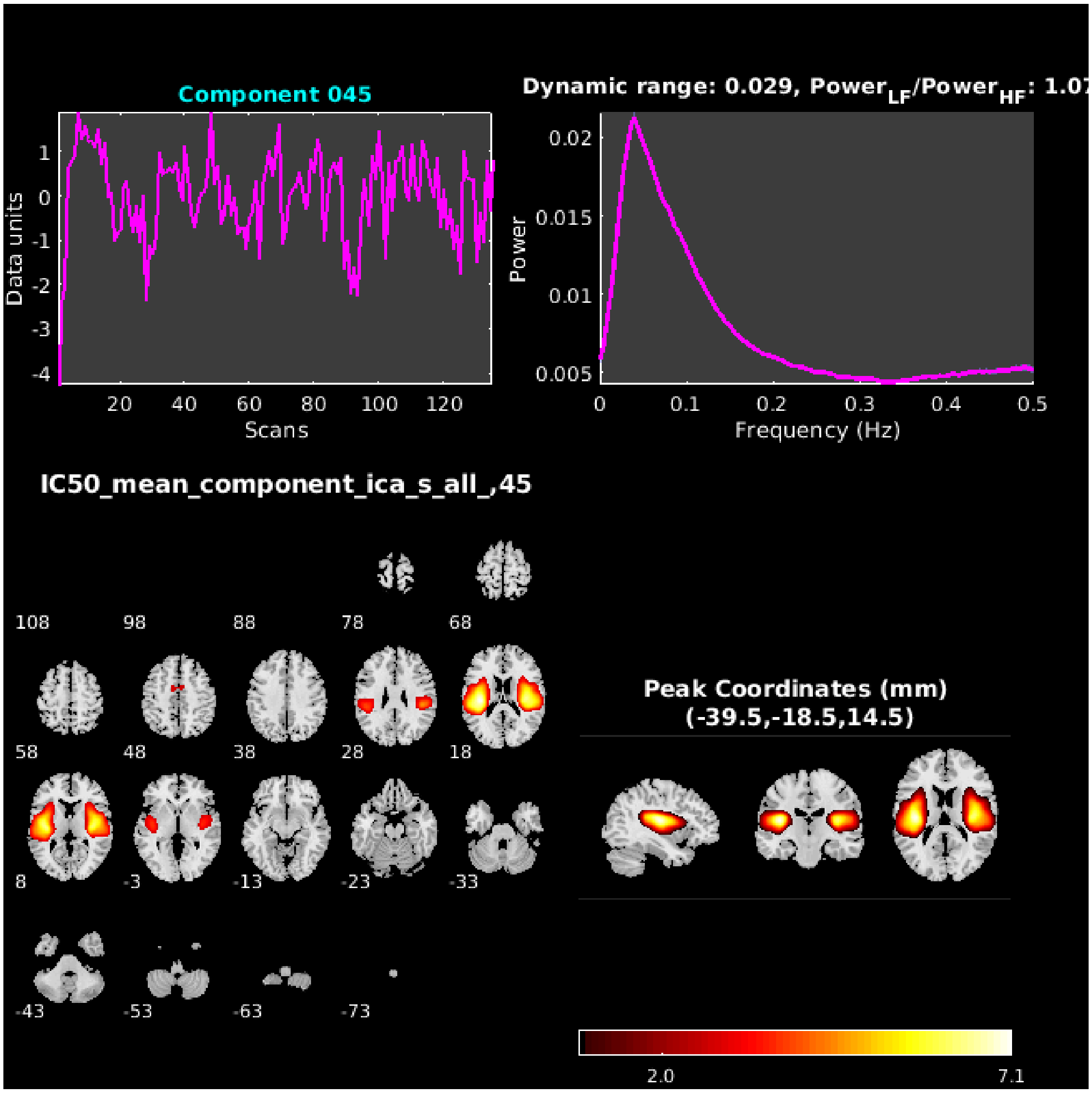

Component 045
Dynamic range: 0.029, Power$_{LF}$/Power$_{HF}$: 1.07
Data units
Scans
Power
Frequency (Hz)
IC50_mean_component_ica_s_all_,45
108
98
88
78
68
58
48
38
28
18
8
-3
-13
-23
-33
-43
-53
-63
-73
Peak Coordinates (mm)
(-39.5,-18.5,14.5)
2.0
7.1

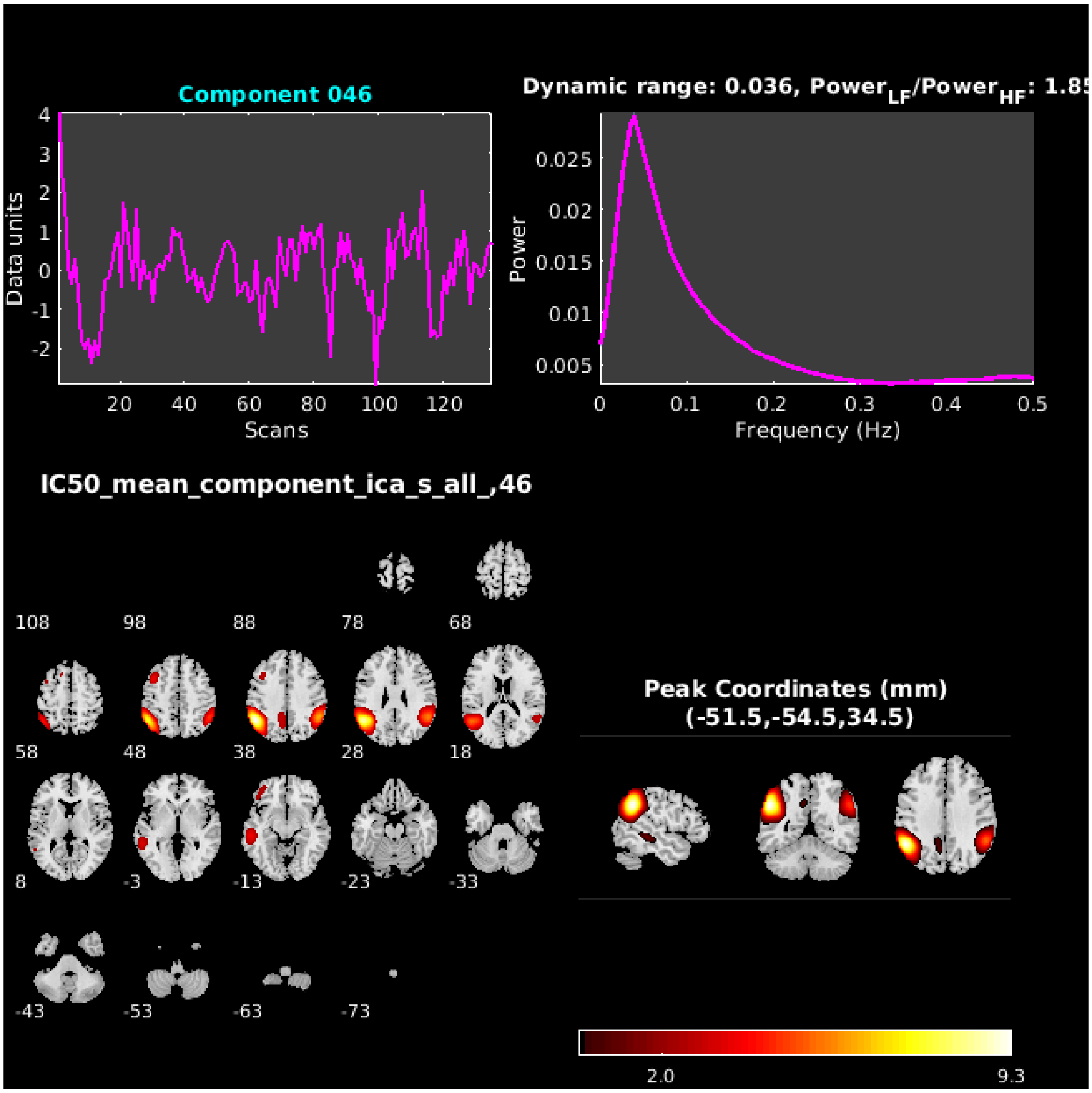
Component 046
Dynamic range: 0.036, Power$_{LF}$/Power$_{HF}$: 1.85
Data units
Scans
Power
Frequency (Hz)
IC50_mean_component_ica_s_all_,46
Peak Coordinates (mm)
(-51.5,-54.5,34.5)
2.0
9.3

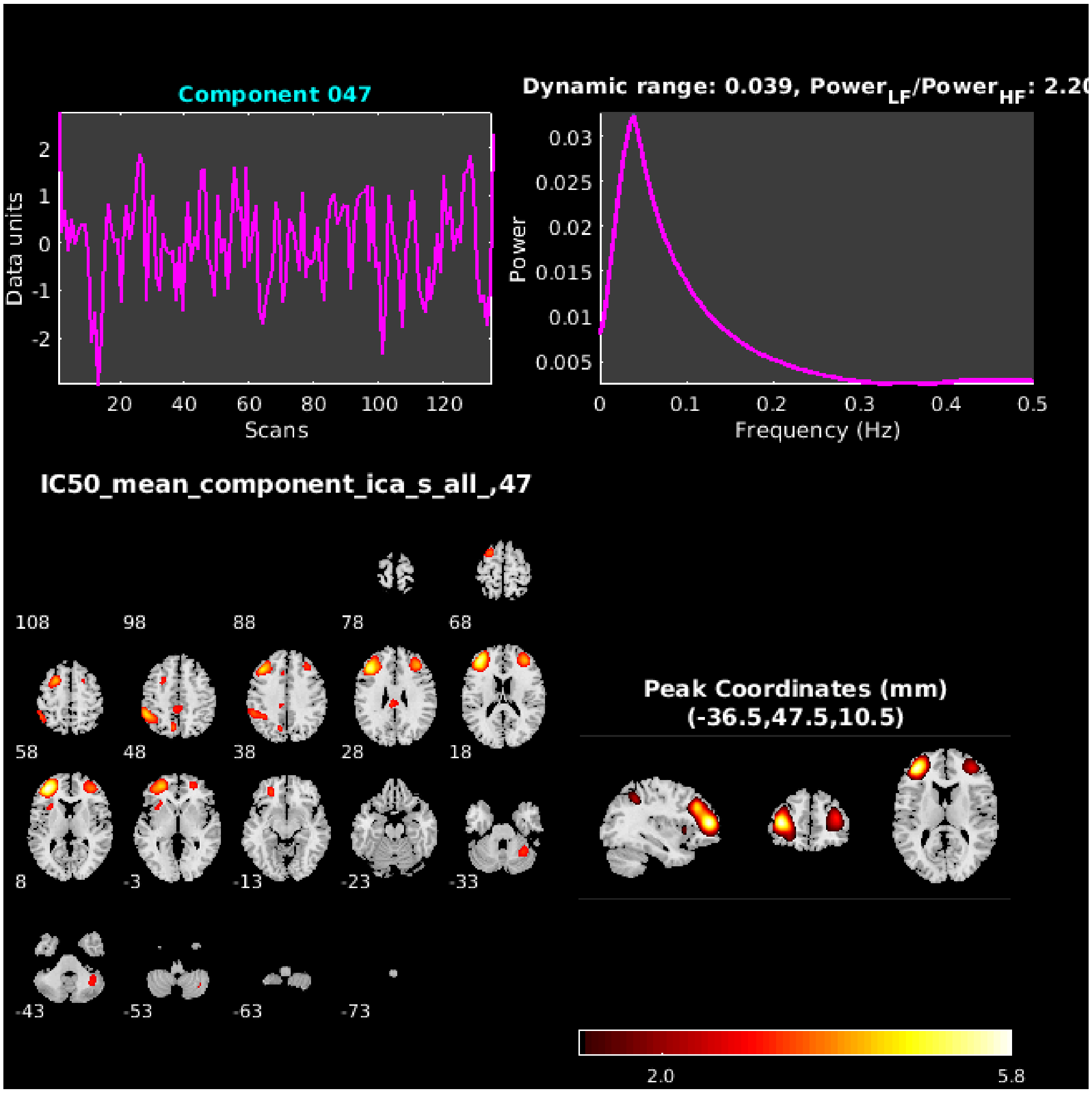
Component 047
Dynamic range: 0.039, Power$_{LF}$/Power$_{HF}$: 2.20
Data units
Scans
Power
Frequency (Hz)
IC50_mean_component_ica_s_all_,47
Peak Coordinates (mm)
(-36.5,47.5,10.5)
2.0
5.8

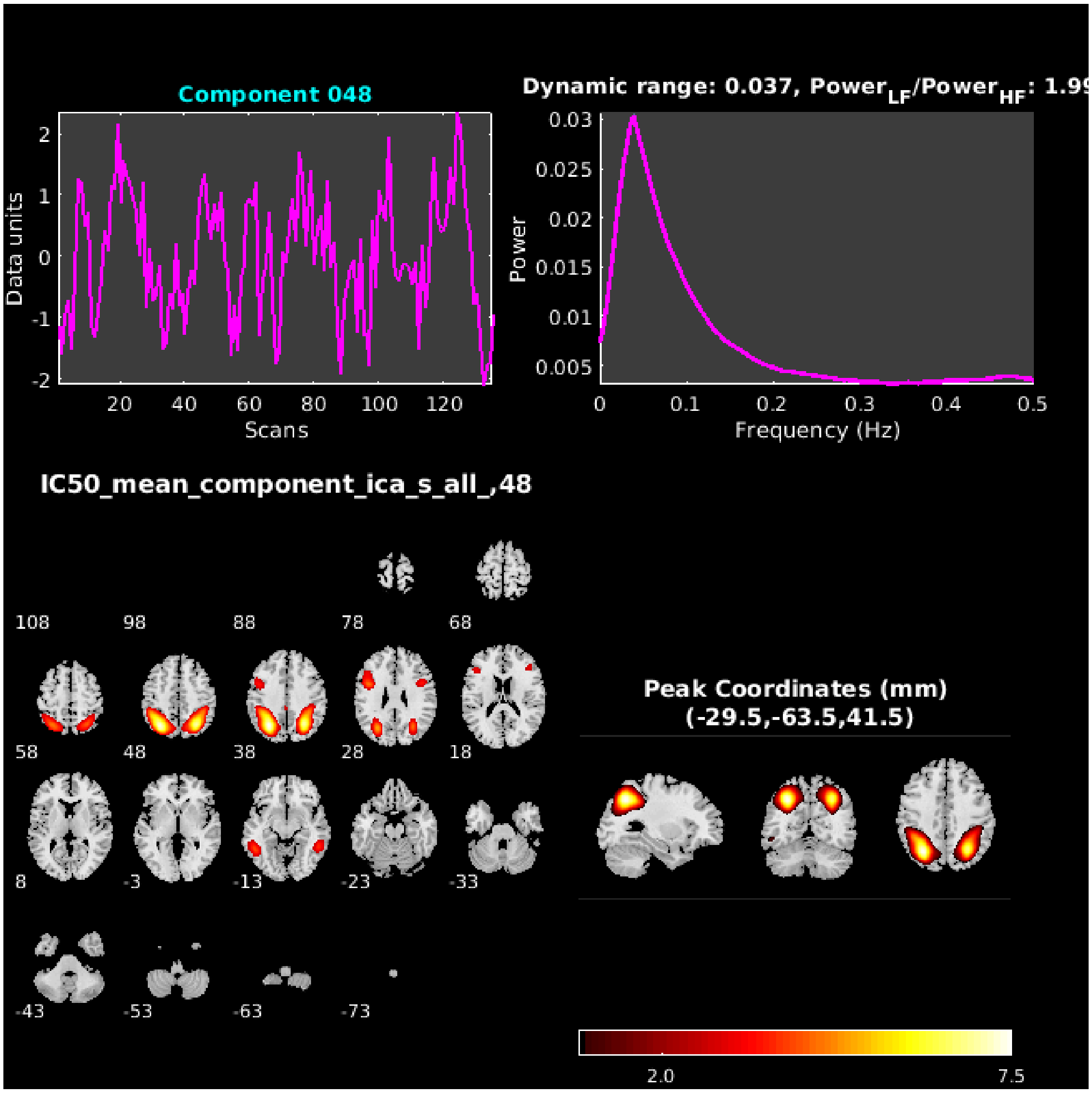

Component 048
Dynamic range: 0.037, Power$_{LF}$/Power$_{HF}$: 1.99
Data units
Scans
Power
Frequency (Hz)
IC50_mean_component_ica_s_all_,48
Peak Coordinates (mm)
(-29.5,-63.5,41.5)
2.0
7.5

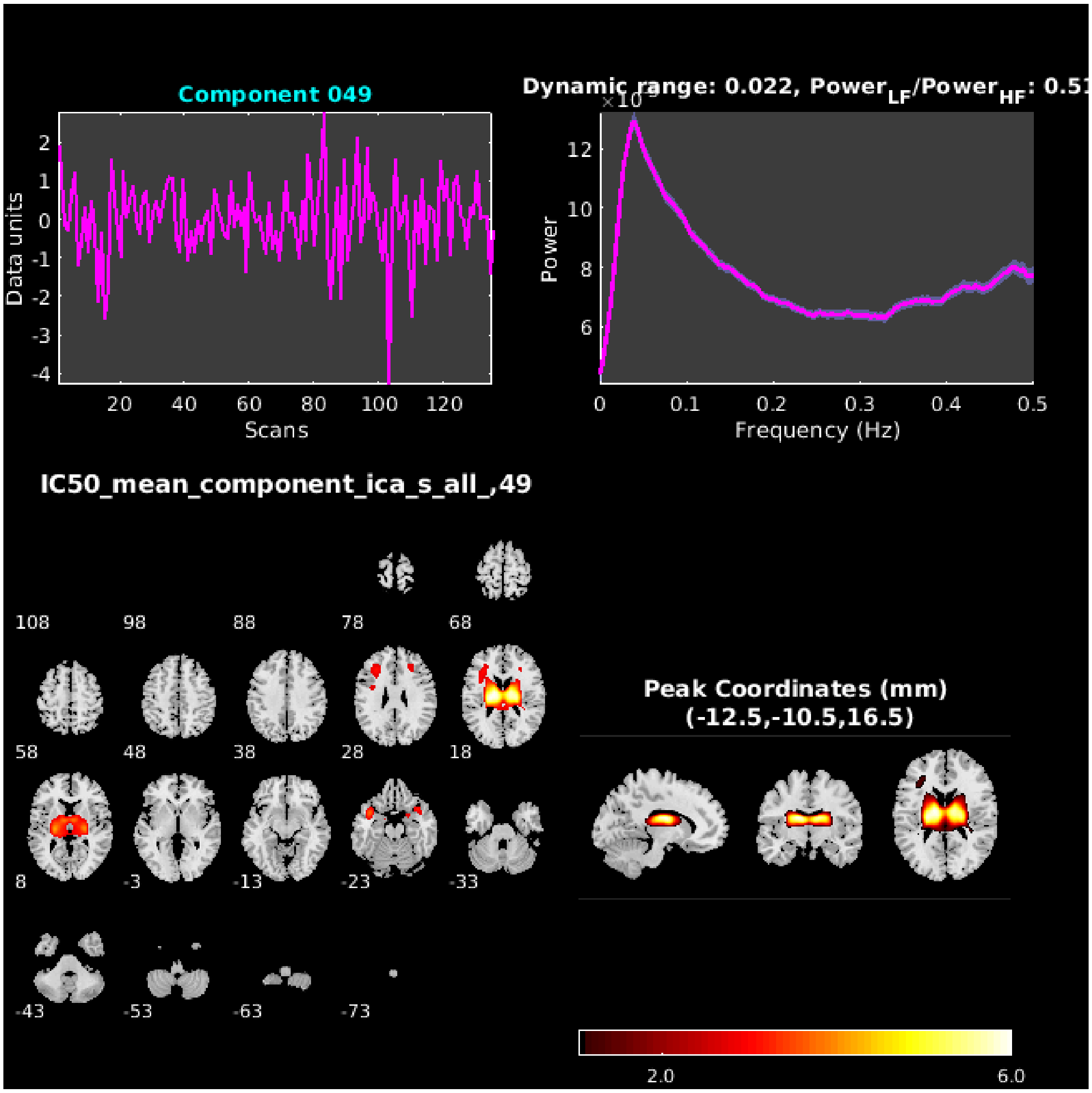

Component 049
Dynamic range: 0.022, Power_LF/Power_HF: 0.51
Data units
Scans
Power
Frequency (Hz)
IC50_mean_component_ica_s_all_,49
Peak Coordinates (mm)
(-12.5,-10.5,16.5)
2.0
6.0

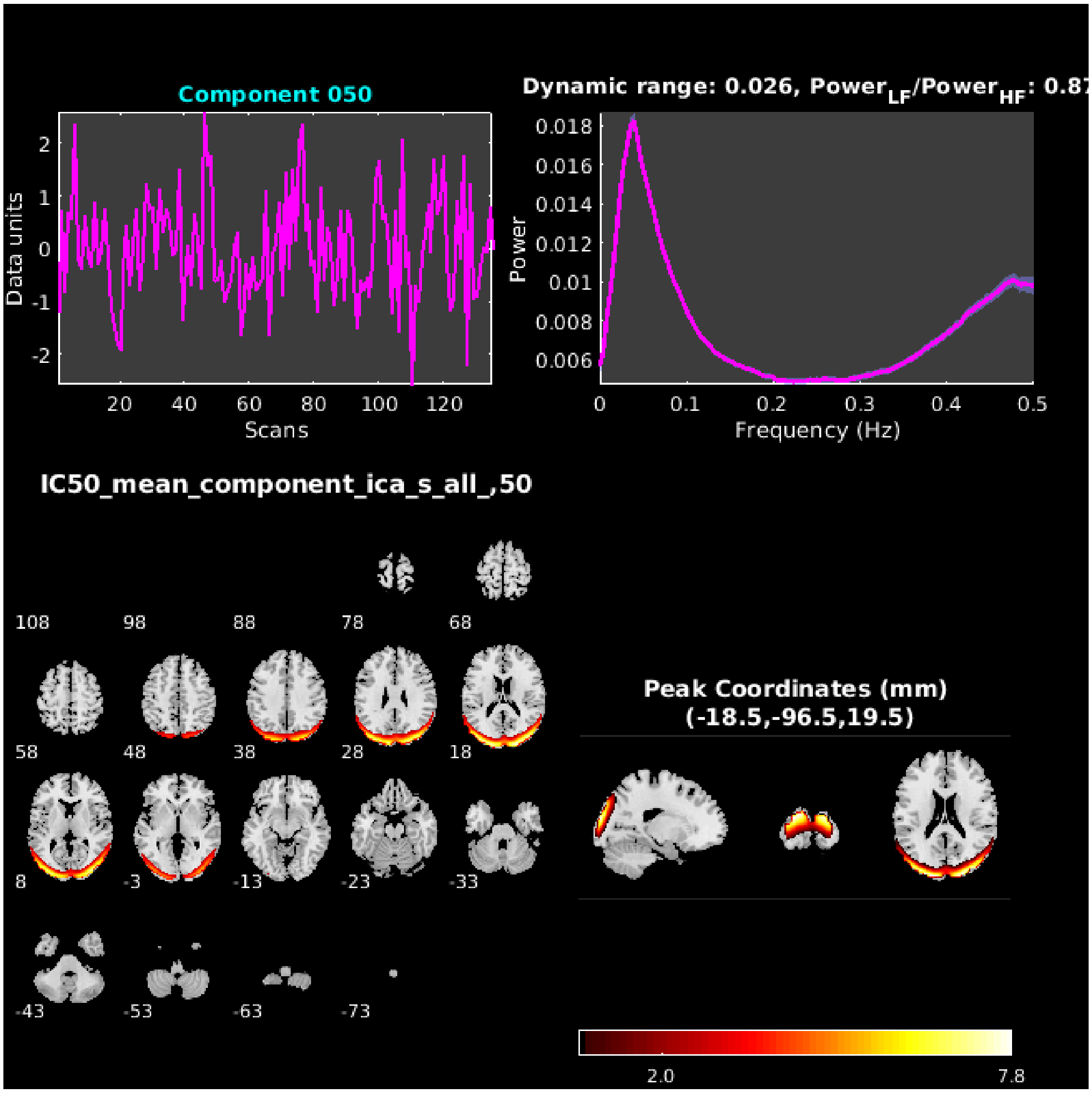

Spectral Summary

- **a) dynamic_range** - Difference between the peak power and minimum power at frequencies to the right of the peak.
- **b) fALFF** - Low frequency to high frequency power ratio.

ComponentNumber	*DynamicRange*	*fALFF*
1	*0.02591*	*0.75718*
2	*0.024652*	*0.52168*
3	*0.025711*	*0.69512*
4	*0.032543*	*1.5382*
5	*0.031325*	*1.1821*
6	*0.025942*	*0.82154*
7	*0.033378*	*1.6117*

8	0.031541	1.4098
9	0.034497	1.6908
10	0.028993	0.92913
11	0.034444	1.7473
12	0.025666	0.77649
13	0.02859	0.99911
14	0.022321	0.53113
15	0.02767	0.91354
16	0.028499	0.98574
17	0.02751	0.90951
18	0.031232	1.2872
19	0.024883	0.69304
20	0.024714	0.7301
21	0.036434	2.0127
22	0.028652	1.05
23	0.029392	1.0665
24	0.028476	0.94631
25	0.026468	0.84367
26	0.03097	1.2193
27	0.032245	1.3943
28	0.036044	2.228
29	0.023216	0.6165
30	0.032745	1.4678
31	0.030156	1.1007
32	0.02481	0.76065
33	0.032321	1.5755
34	0.025232	0.7503
35	0.02251	0.57647
36	0.025412	0.6882
37	0.033584	1.6681
38	0.031487	1.3688
39	0.030302	1.2224
40	0.025949	0.70313
41	0.027609	0.95342
42	0.036664	1.9171
43	0.037744	2.2721
44	0.035516	1.7481
45	0.028755	1.076
46	0.035526	1.8589
47	0.038921	2.2092
48	0.036514	1.9958
49	0.02205	0.50962
50	0.026428	0.87714

Temporal Stats On Beta Weights

Multiple regression is done using the timecourses from SPM design matrix as model and ICA timecourses as observations. R^2 values for each component are shown in bar plot. For each component, one sample t-test results of each session and condition are shown in the bar plots.

Kurtosis of timecourses and spatial maps

Mean across subjects is reported in table. Figure shows mean+/- SEM across subjects

ComponentNumber	Timecourses	SpatialMaps
1	3.5826	12.968
2	5.0446	5.4547
3	4.4576	5.8714
4	4.1943	6.7908
5	4.3728	6.3489
6	4.5304	5.7032
7	4.1905	4.8848
8	4.3134	5.6596
9	3.5755	5.4118
10	4.7607	4.8801
11	4.3025	4.5327
12	4.3395	4.4514
13	4.924	4.7677
14	4.0007	7.2998
15	4.3163	5.6478
16	3.8833	4.7082
17	4.3199	4.8237
18	4.6827	4.1749
19	5.5383	4.022
20	5.303	5.3525
21	4.3327	4.1523
22	4.6146	4.06
23	4.1328	4.5982
24	4.4166	4.3845
25	5.3615	4.3854
26	4.4091	4.2467
27	6.0374	4.4087
28	4.1796	5.5316
29	4.5923	4.7771
30	4.0951	4.5273
31	5.3983	3.7702
32	4.7848	4.1911
33	4.3717	4.7937
34	6.0259	4.2796
35	4.5175	5.5345
36	4.5752	4.4032
37	3.81	4.0542
38	5.0163	4.1043
39	4.6865	4.1625
40	4.0889	4.428
41	5.3014	4.2879
42	3.4424	3.9168
43	3.6032	4.3545
44	4.0837	4.0658
45	4.1397	4.0121
46	3.7271	4.3573

47	*3.4801*	*3.7378*
48	*3.9872*	*3.9823*
49	*4.6138*	*3.6839*
50	*4.9609*	*4.3507*

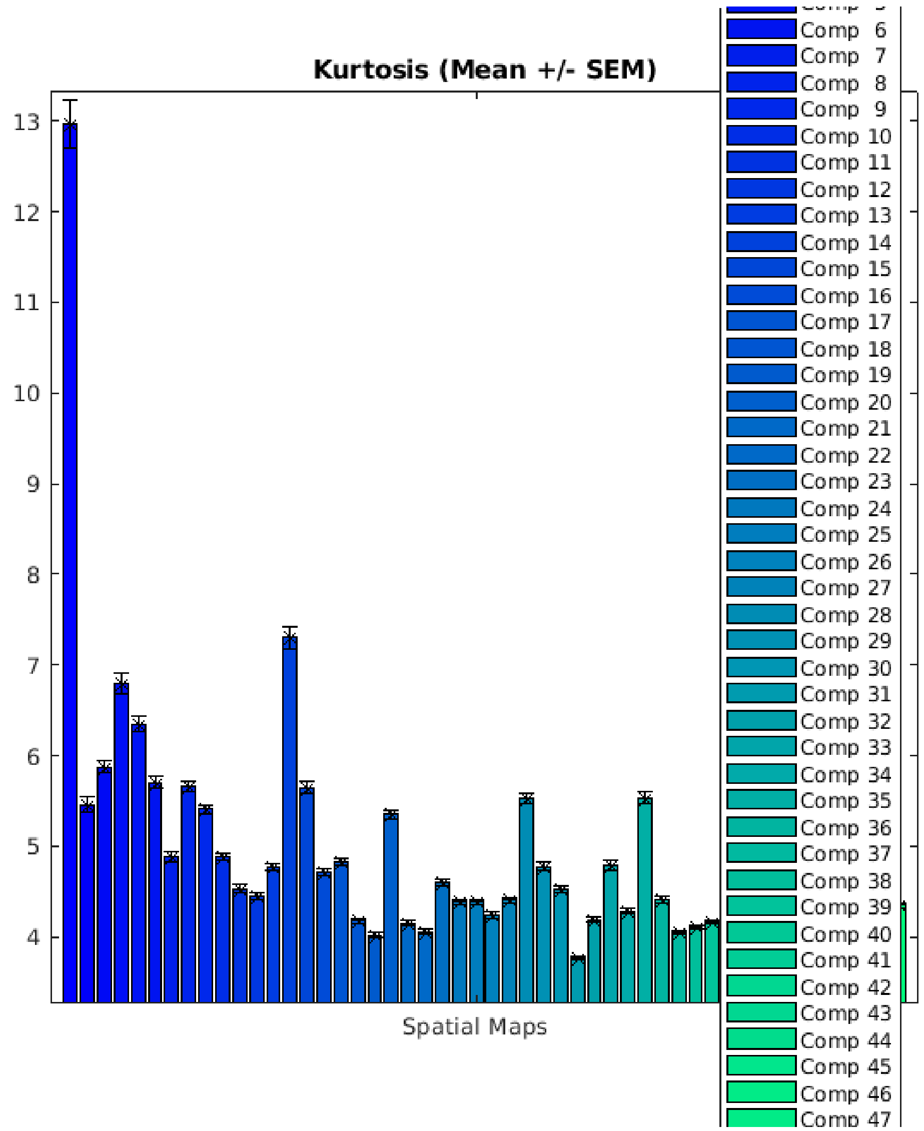

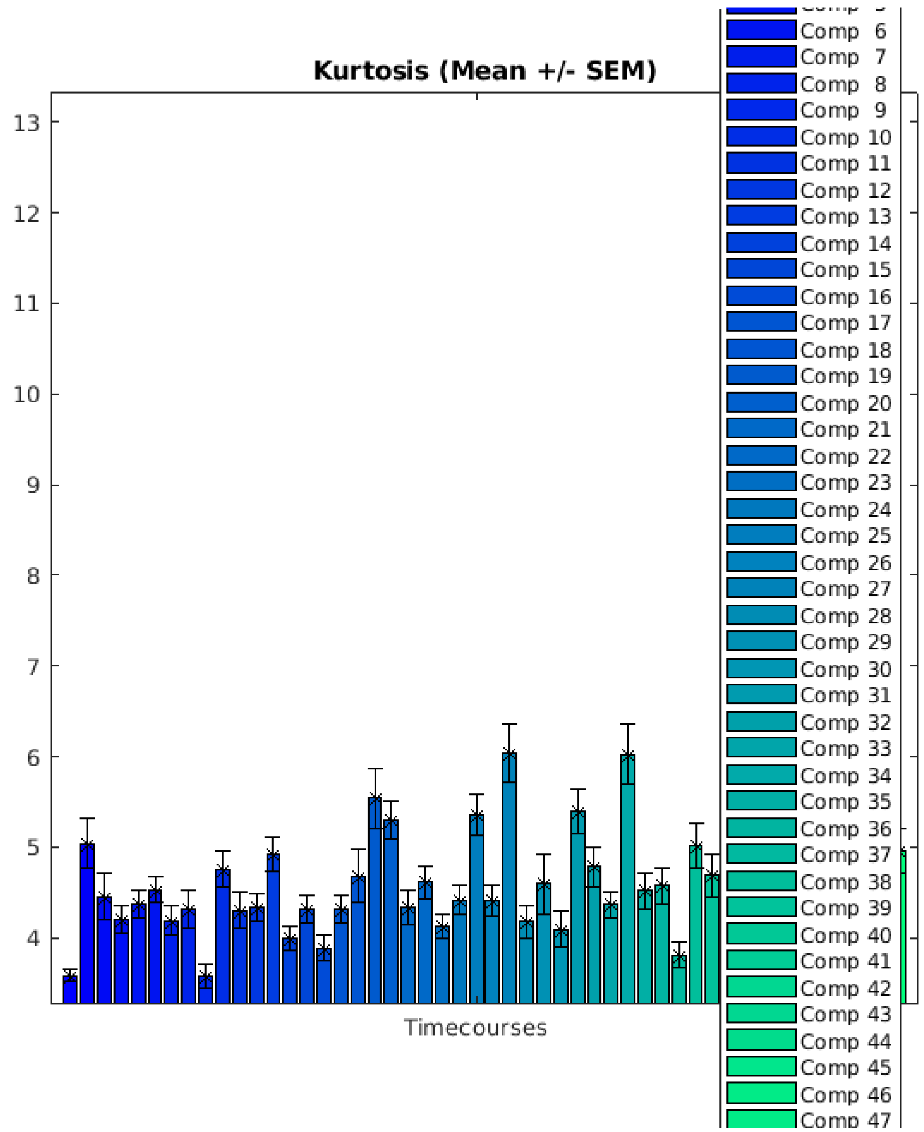

FNC correlations

Functional network connectivity correlations are computed for each data-set and averaged across sessions.

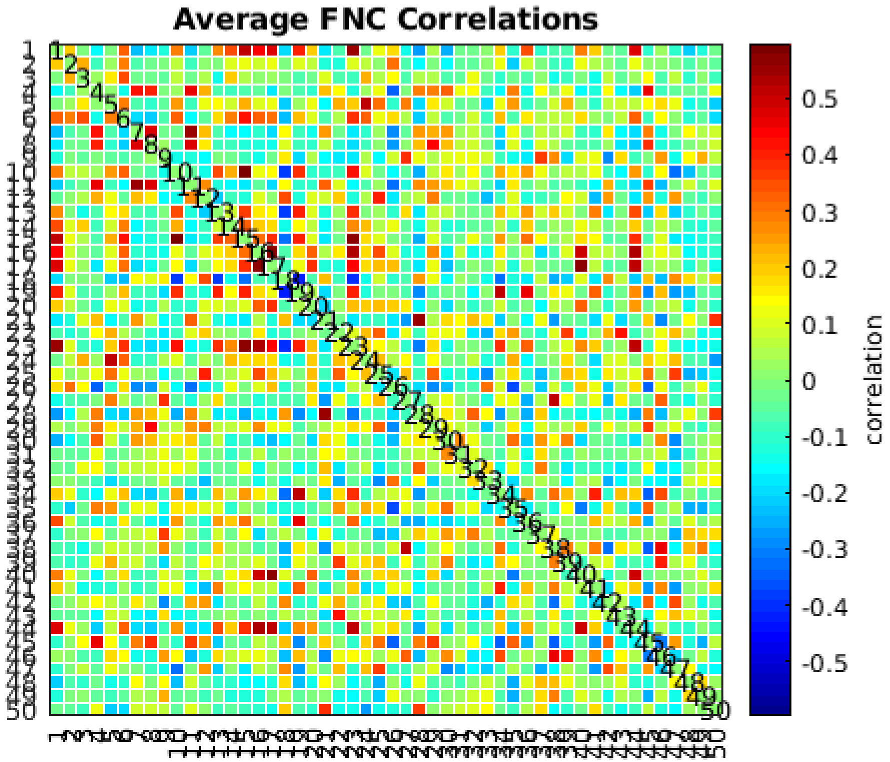

FNC metrics of component spatial maps

Mutual information is computed between components spatially and averaged across data-sets.

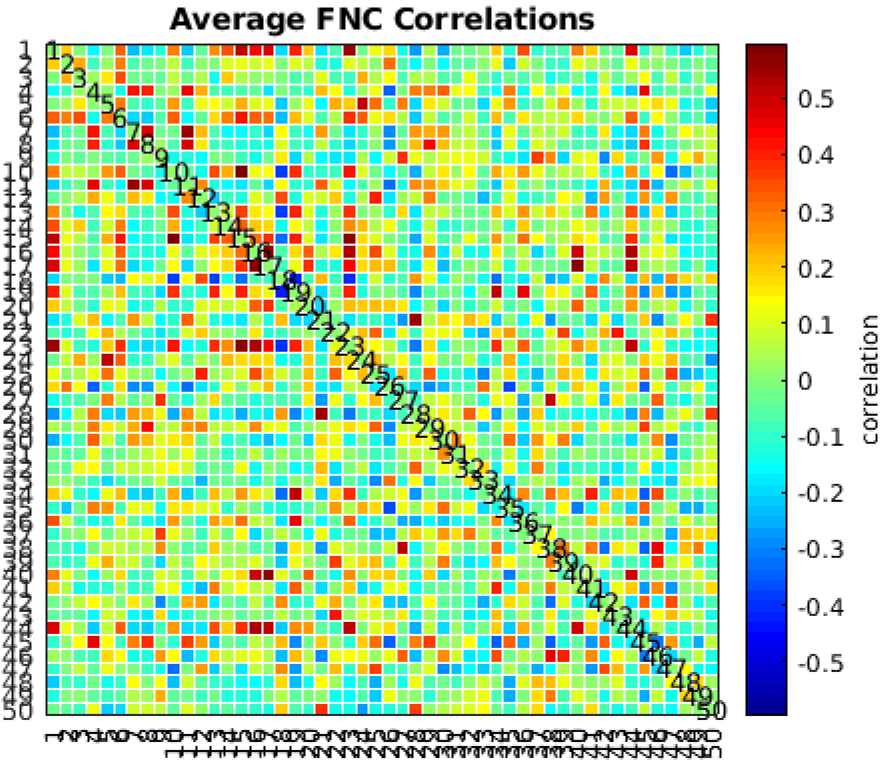
Average FNC Correlations
0.5
0.4
0.3
0.2
0.1
0
-0.1
-0.2
-0.3
-0.4
-0.5
correlation

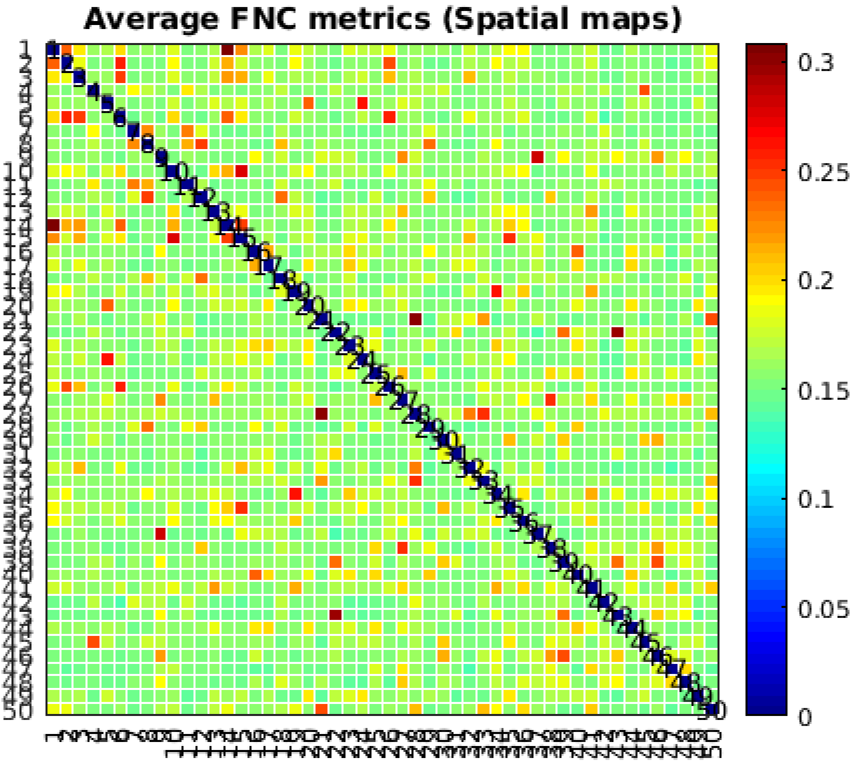
Average FNC metrics (Spatial maps)
0.3
0.25
0.2
0.15
0.1
0.05
0

Published with MATLAB® R2019a
