# Supplementary material for: Multi-spatial-scale dynamic interactions between functional sources reveal sex-specific changes in schizophrenia
Source: Netw Neurosci. 2022 Jun 1;6(2):357–81. doi: 10.1162/netn_a_00196 (PMC9208002; doi:10.1162/netn_a_00196)
Supplement: Supplementary file 1 [file netn-06-357-s001.zip › icatb_gica_html_report75.pdf]

Table of Contents

.... 1
Group ICA Parameters 1
ICASSO Plots 2
Mean Components 5
Spectral Summary 79
Temporal Stats On Beta Weights 81
Kurtosis of timecourses and spatial maps 81
FNC correlations 84
FNC metrics of component spatial maps 85

Group ICA Parameters

...

Number of Subjects : 856

Number of Sessions : 1

Number of Independent Components : 75

ICA Algorithm : Infomax

Number Of Scans/Timepoints : 157

Mask File : mask_common

Data Pre-processing Type : Variance Normalization

PCA Type : Standard

Group PCA Type : Subject Specific

Group ICA Type : Spatial

Back Reconstruction Type : Spatial-temporal Regression

Scaling Components : Z-scores

Stability analysis type : ICASSO

Group analysis mode: Parallel

Anatomical file: /trdapps/linux-x86_64/matlab/toolboxes/GroupICATv4.0b/icatb/icatb_templates/ch2bet.nii
Slice Plane: Axial

```
Image values: Positive

Convert to Z-scores: yes

Threshold: 1.96


...................................................
```

ICASSO Plots

```
Warning: Creates overwhelming number of lines
Warning: Tries to change the limit...
Warning: New limit =0.99563
```

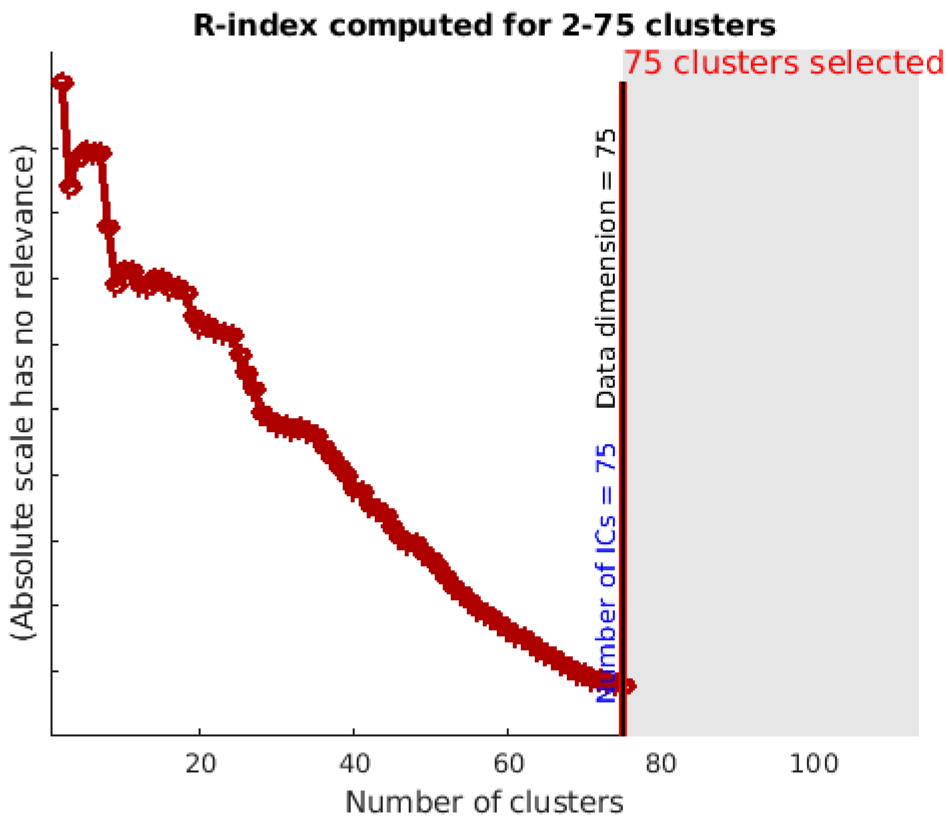

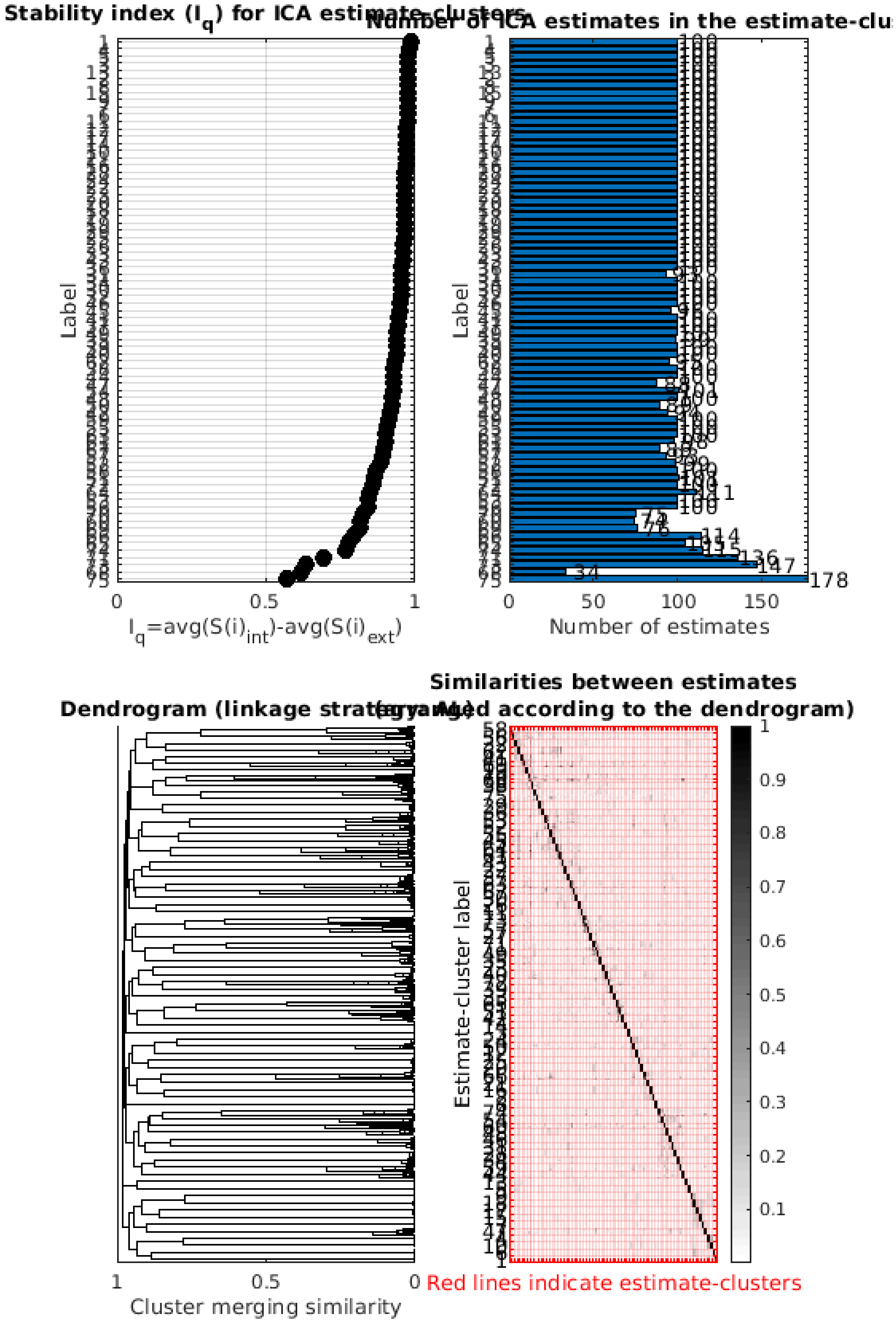
Stability index (I_q) for ICA estimate-clusters
Number of ICA estimates in the estimate-clus
Label
0
0.5
1
I_q=avg(S(i)$_{int}$)-avg(S(i)$_{ext}$)
Label
0
50
100
150
Number of estimates
Dendrogram (linkage strategy: AL)
1
0.5
0
Cluster merging similarity
Similarities between estimates
(arranged according to the dendrogram)
Estimate-cluster label
1
0.9
0.8
0.7
0.6
0.5
0.4
0.3
0.2
0.1
Red lines indicate estimate-clusters

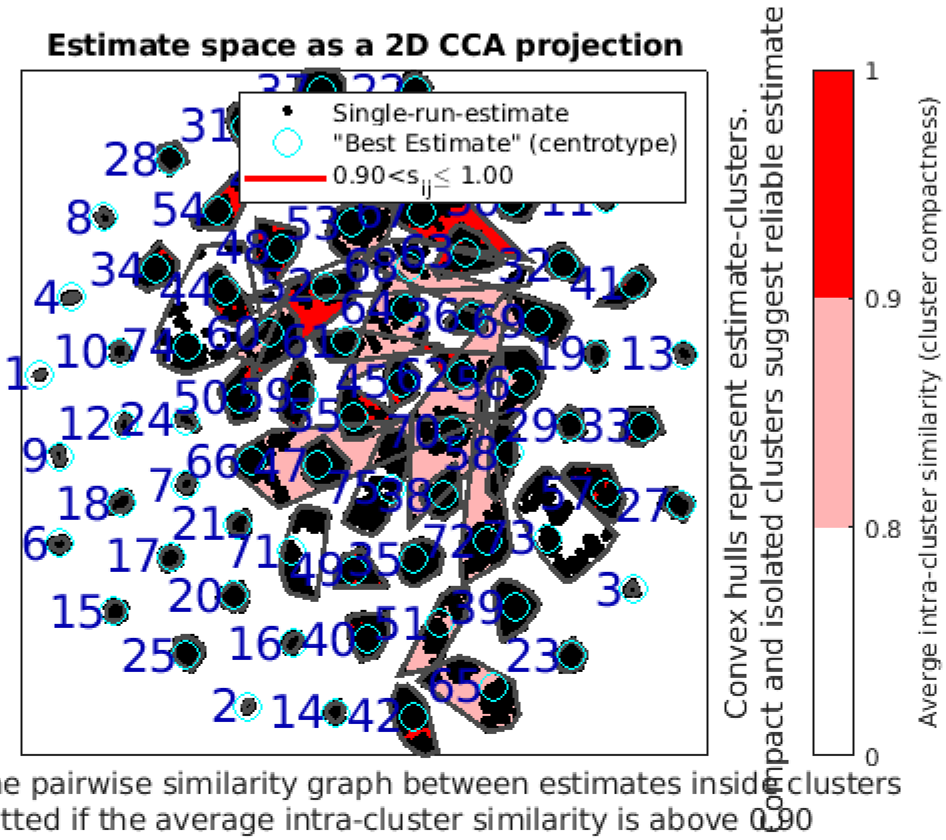
Estimate space as a 2D CCA projection
Single-run-estimate
"Best Estimate" (centrotype)
0.90<s$_{ij}$≤ 1.00
Convex hulls represent estimate-clusters.
Compact and isolated clusters suggest reliable estimate
Averge intra-cluster similarity (cluster compactness)
1
0.9
0.8
0
e that the pairwise similarity graph between estimates inside clusters
is omitted if the average intra-cluster similarity is above 0.90

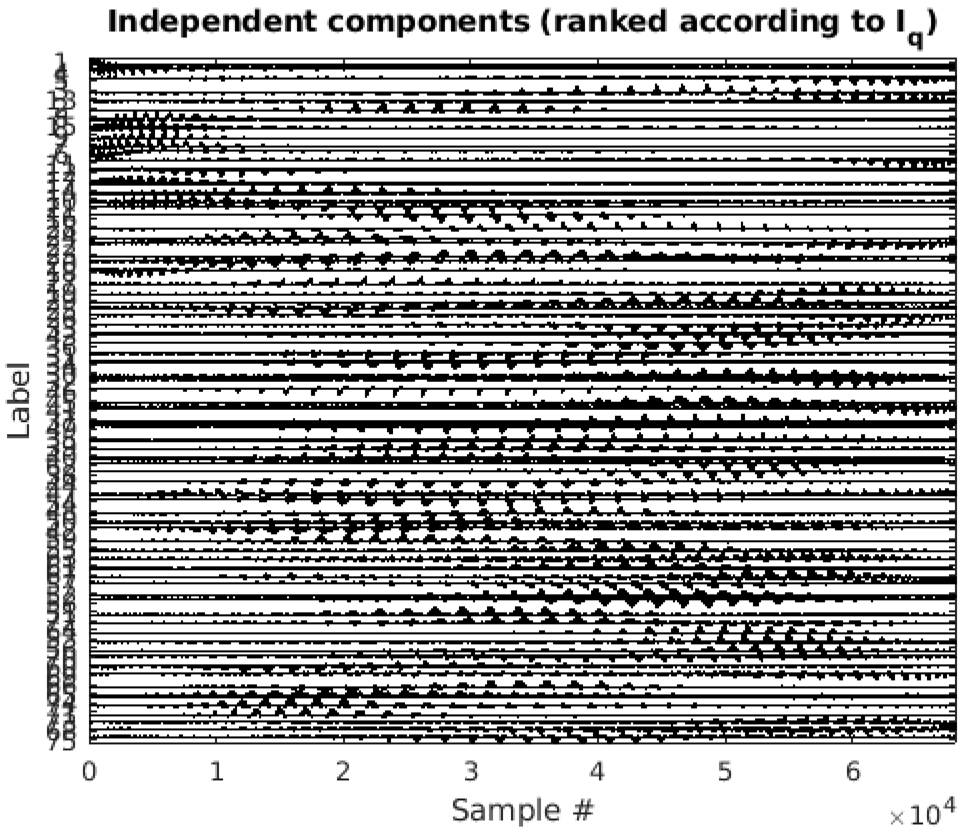
Independent components (ranked according to I$_q$)
Label
Sample #
0
1
2
3
4
5
6
×10^4

Mean Components

Mean across all subjects and sessions is computed for each component

- **a) Timecourse** - Mean timecourse is converted to z-scores.
- **b) Spectra** - Timecourses spectra is computed for each data-set and averaged across sessions. Mean and standard error of mean is shown in the figure.
- **c) Montage** - Axial slices are shown.
- **d) Ortho slices** - Ortho plot is shown for the peak voxel and coordinates are reported.

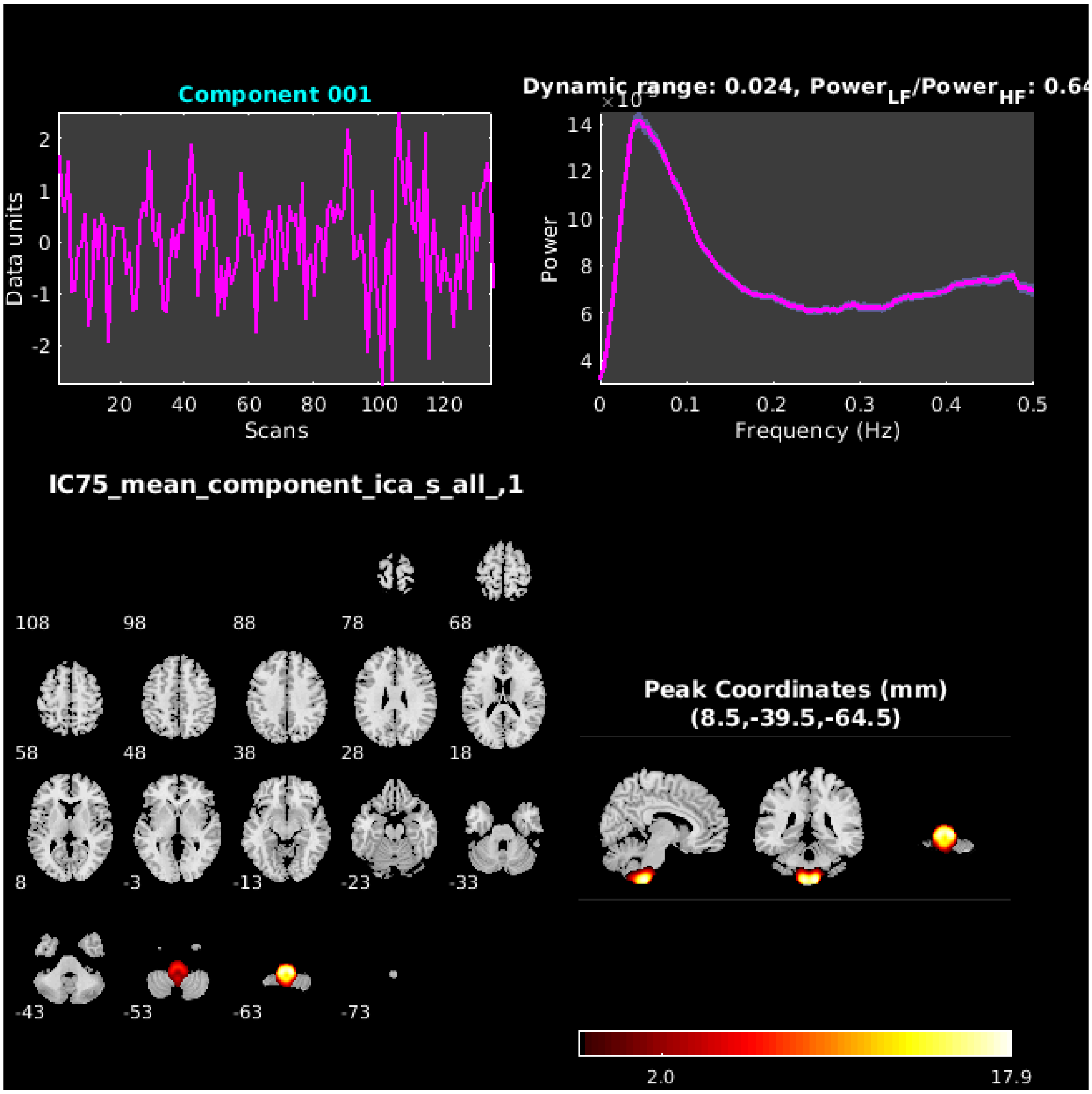

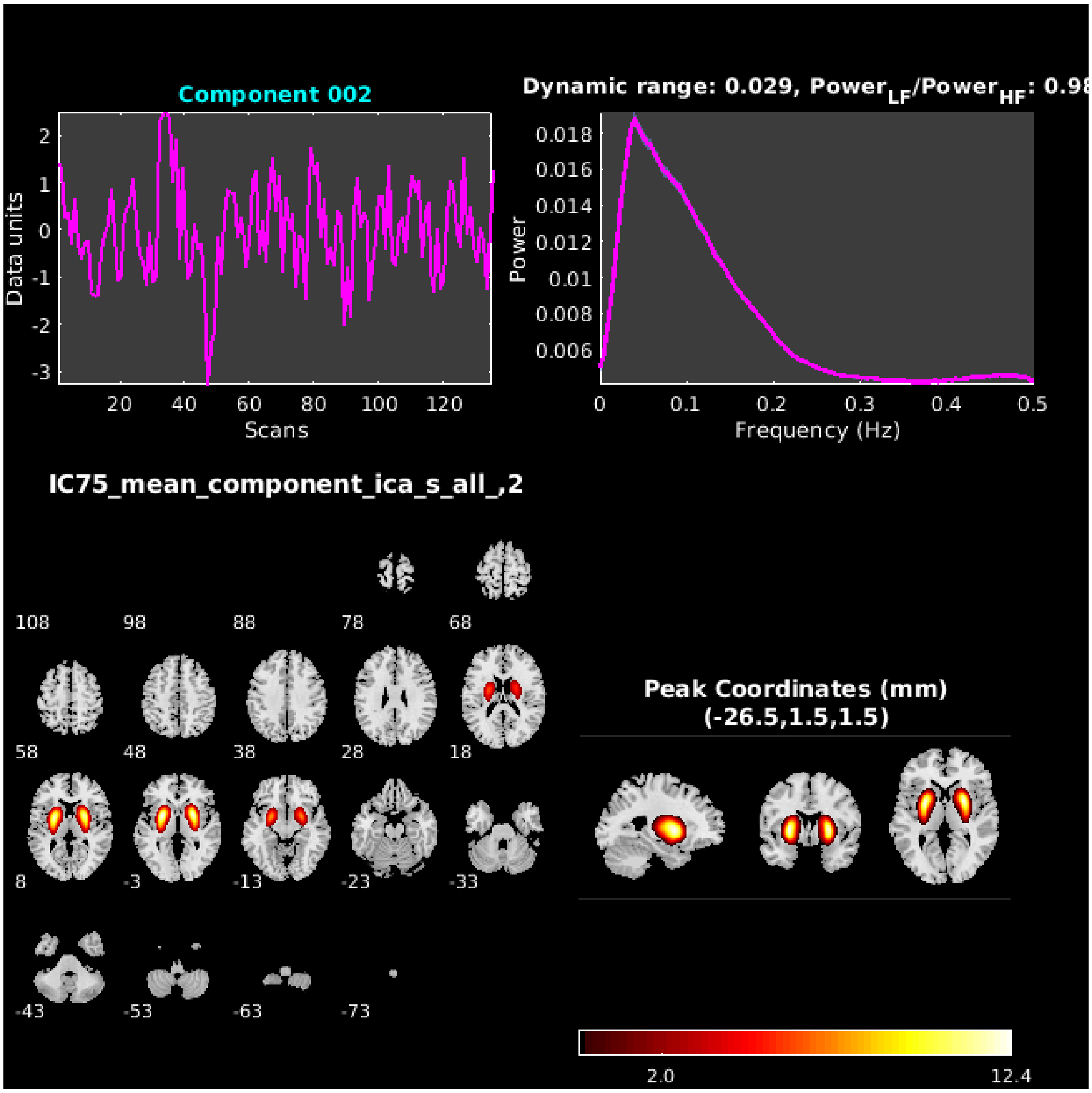
Component 002
Dynamic range: 0.029, Power$_{LF}$/Power$_{HF}$: 0.98
Data units
Scans
Power
Frequency (Hz)
IC75_mean_component_ica_s_all_,2
Peak Coordinates (mm)
(-26.5,1.5,1.5)
2.0
12.4

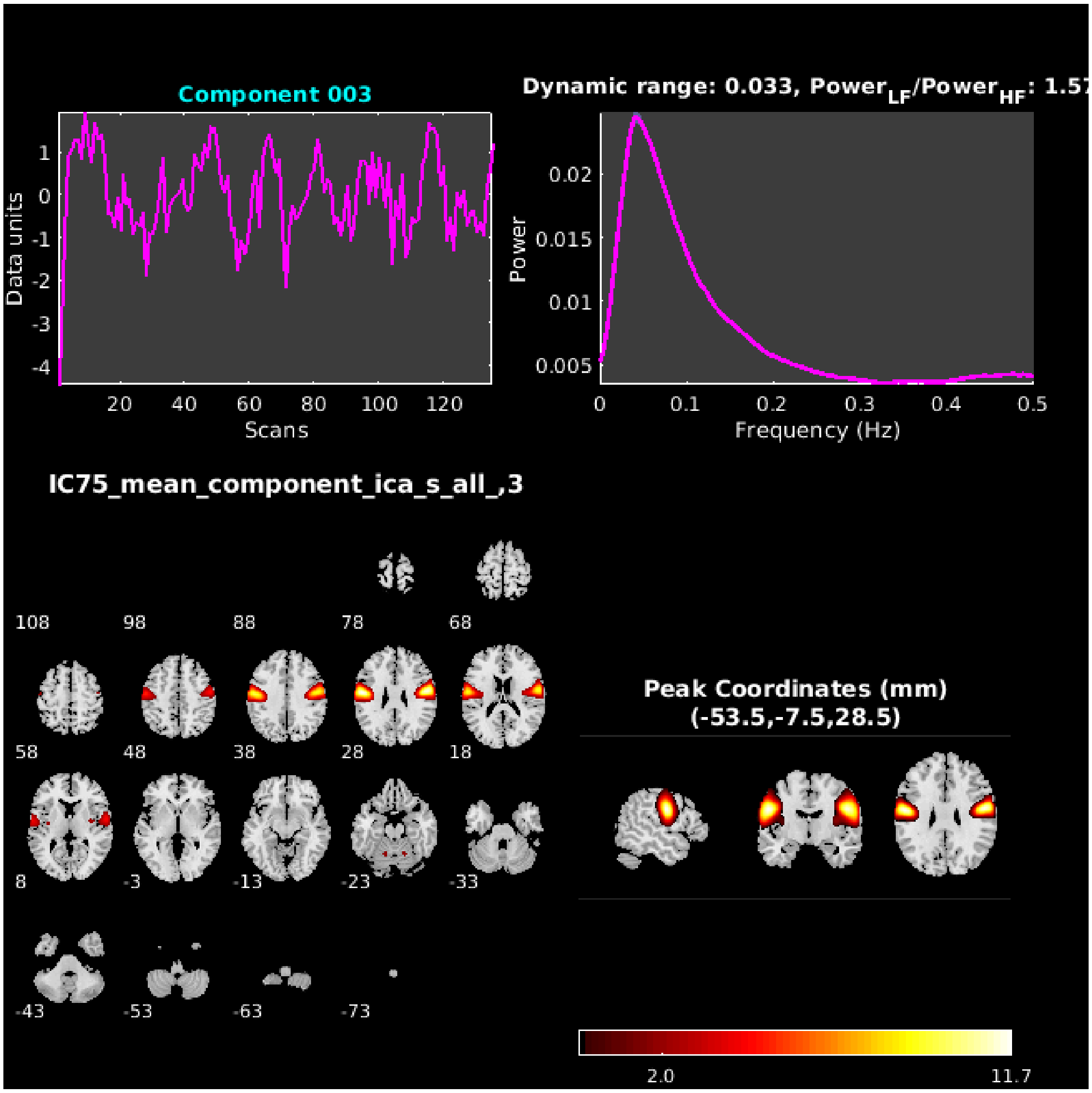

Component 003
Dynamic range: 0.033, Power$_{LF}$/Power$_{HF}$: 1.57
Data units
Scans
Power
Frequency (Hz)
IC75_mean_component_ica_s_all_,3
Peak Coordinates (mm)
(-53.5,-7.5,28.5)
2.0
11.7

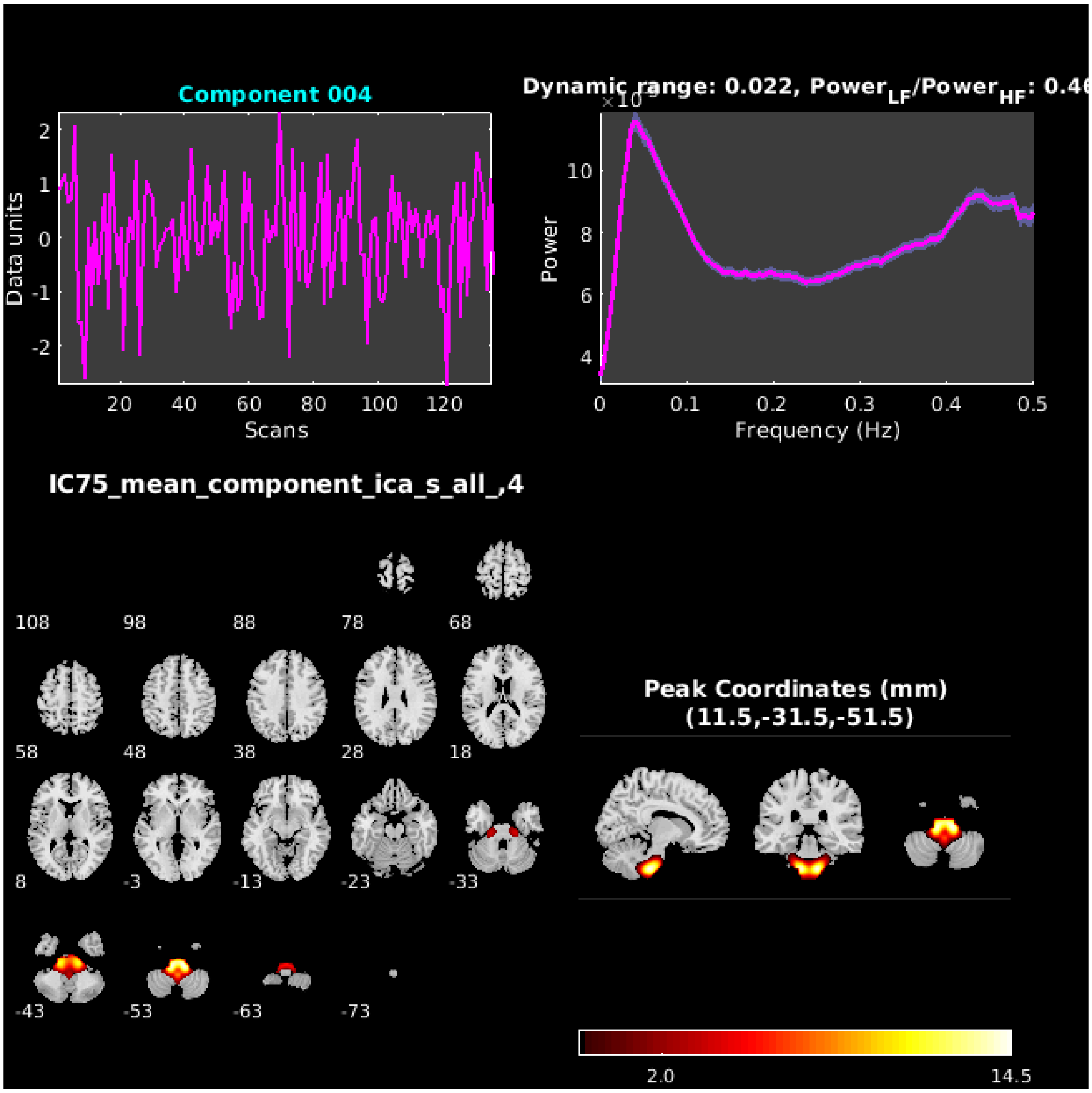
Component 004
Dynamic range: 0.022, Power$_{LF}$/Power$_{HF}$: 0.46
Data units
Scans
Power
Frequency (Hz)
IC75_mean_component_ica_s_all_,4
Peak Coordinates (mm)
(11.5,-31.5,-51.5)
2.0
14.5

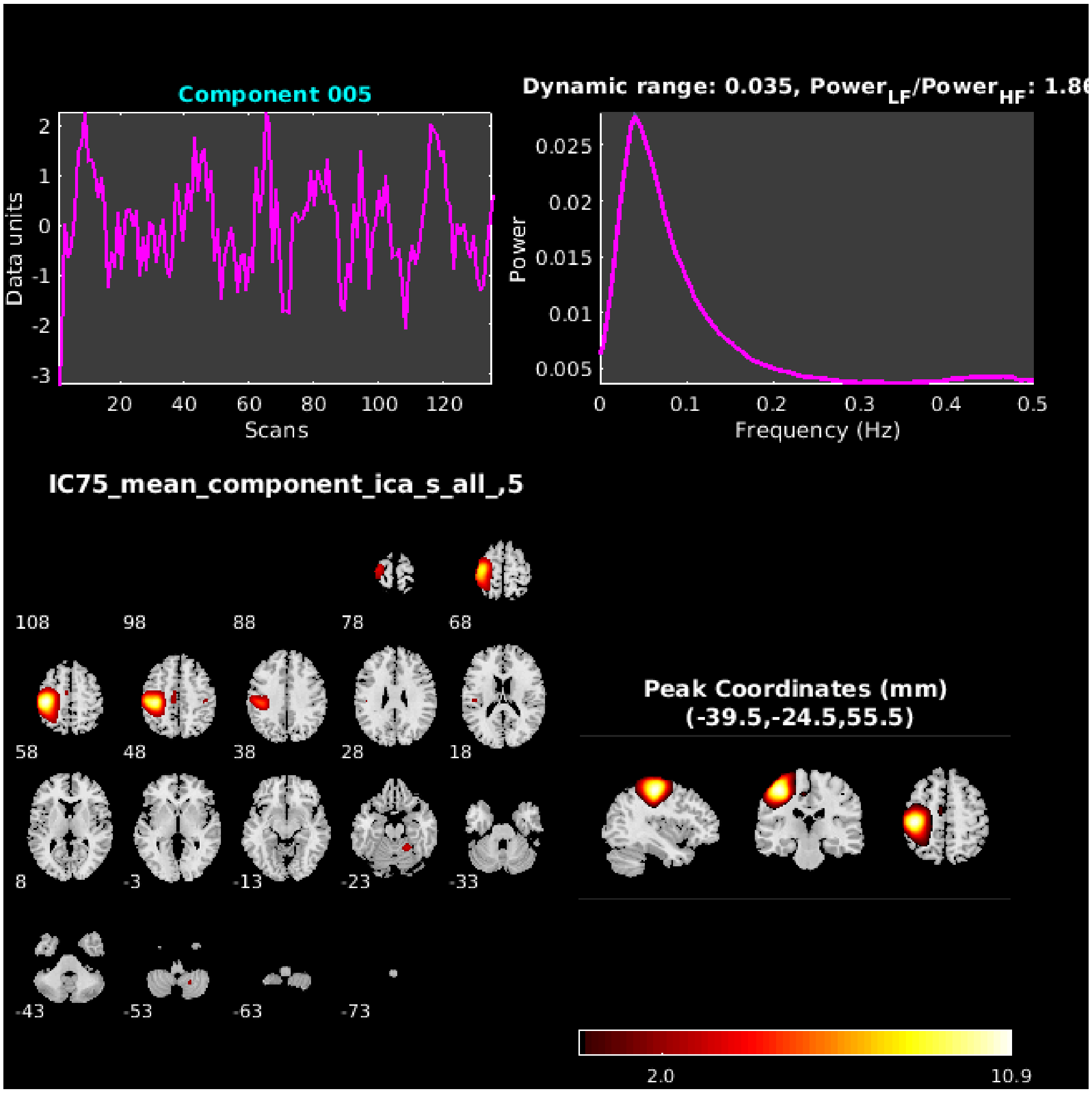

Component 005
Dynamic range: 0.035, Power_LF/Power_HF: 1.86
Data units
Scans
Power
Frequency (Hz)
IC75_mean_component_ica_s_all_,5
108
98
88
78
68
58
48
38
28
18
8
-3
-13
-23
-33
-43
-53
-63
-73
Peak Coordinates (mm)
(-39.5,-24.5,55.5)
2.0
10.9

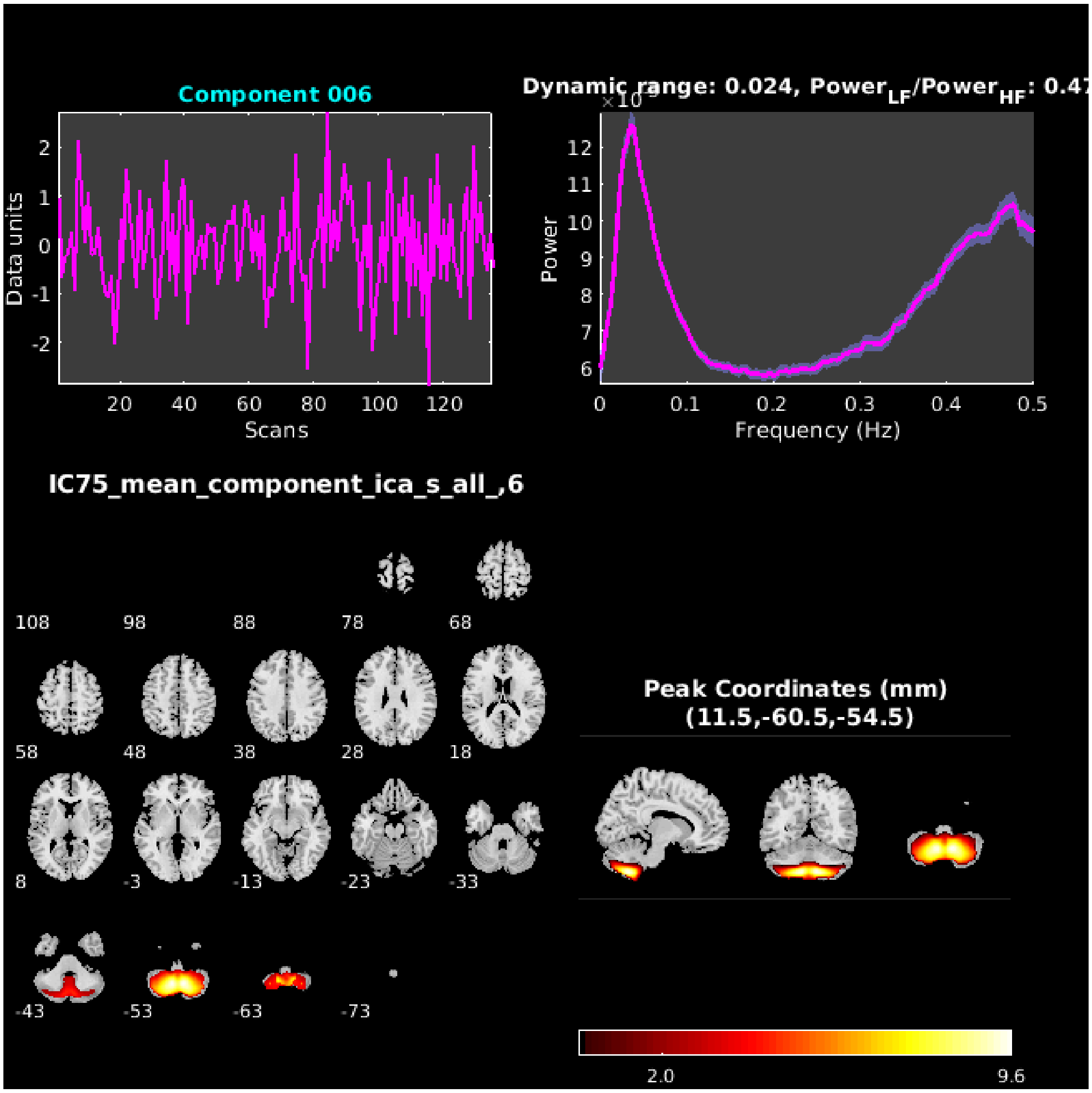
Component 006
Dynamic range: 0.024, Power$_{LF}$/Power$_{HF}$: 0.47
Data units
Scans
Power
Frequency (Hz)
IC75_mean_component_ica_s_all_,6
Peak Coordinates (mm)
(11.5,-60.5,-54.5)
2.0
9.6

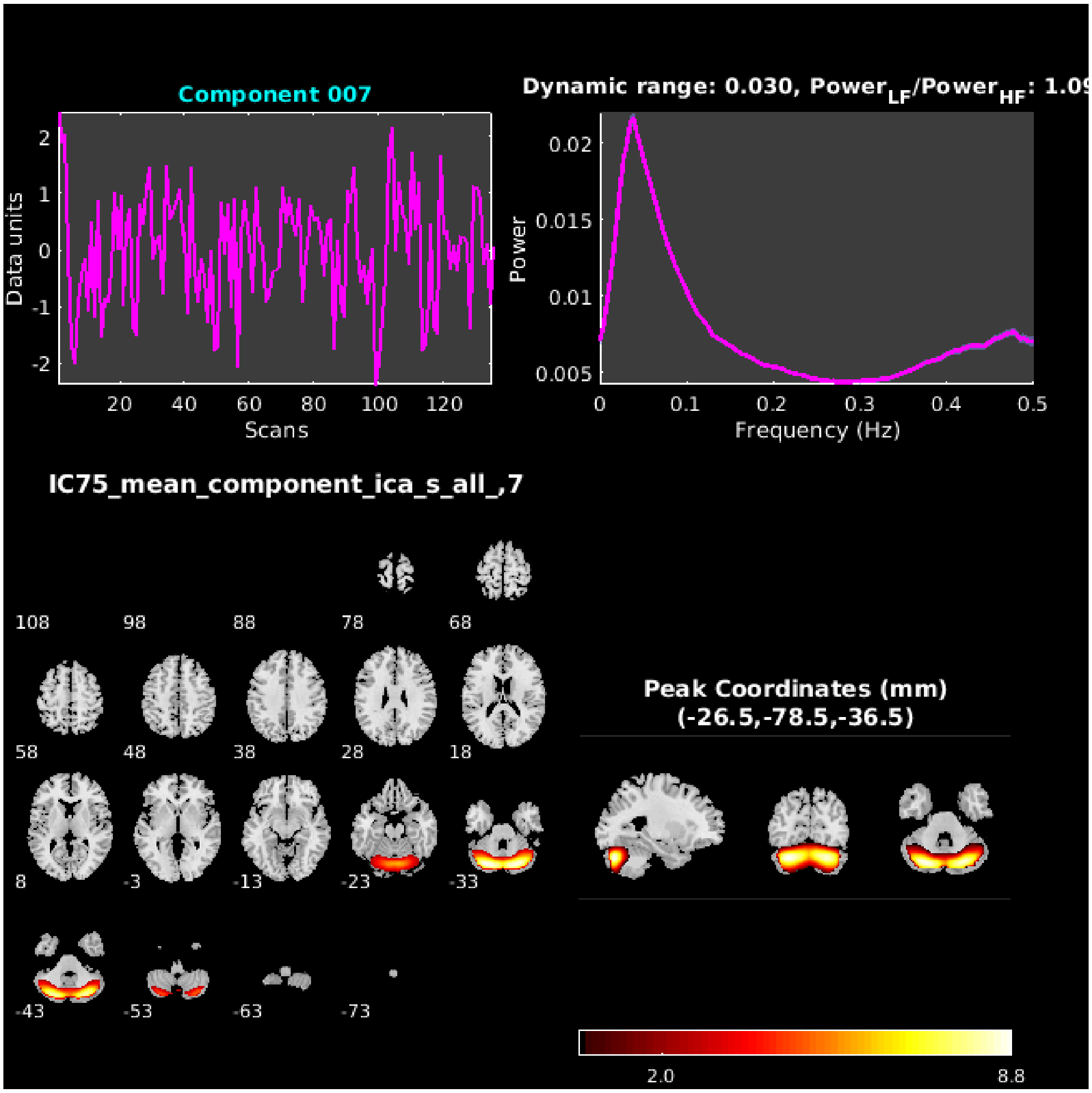

Component 007
Dynamic range: 0.030, Power$_{LF}$/Power$_{HF}$: 1.09
Data units
Scans
Power
Frequency (Hz)
IC75_mean_component_ica_s_all_,7
Peak Coordinates (mm)
(-26.5,-78.5,-36.5)
2.0
8.8

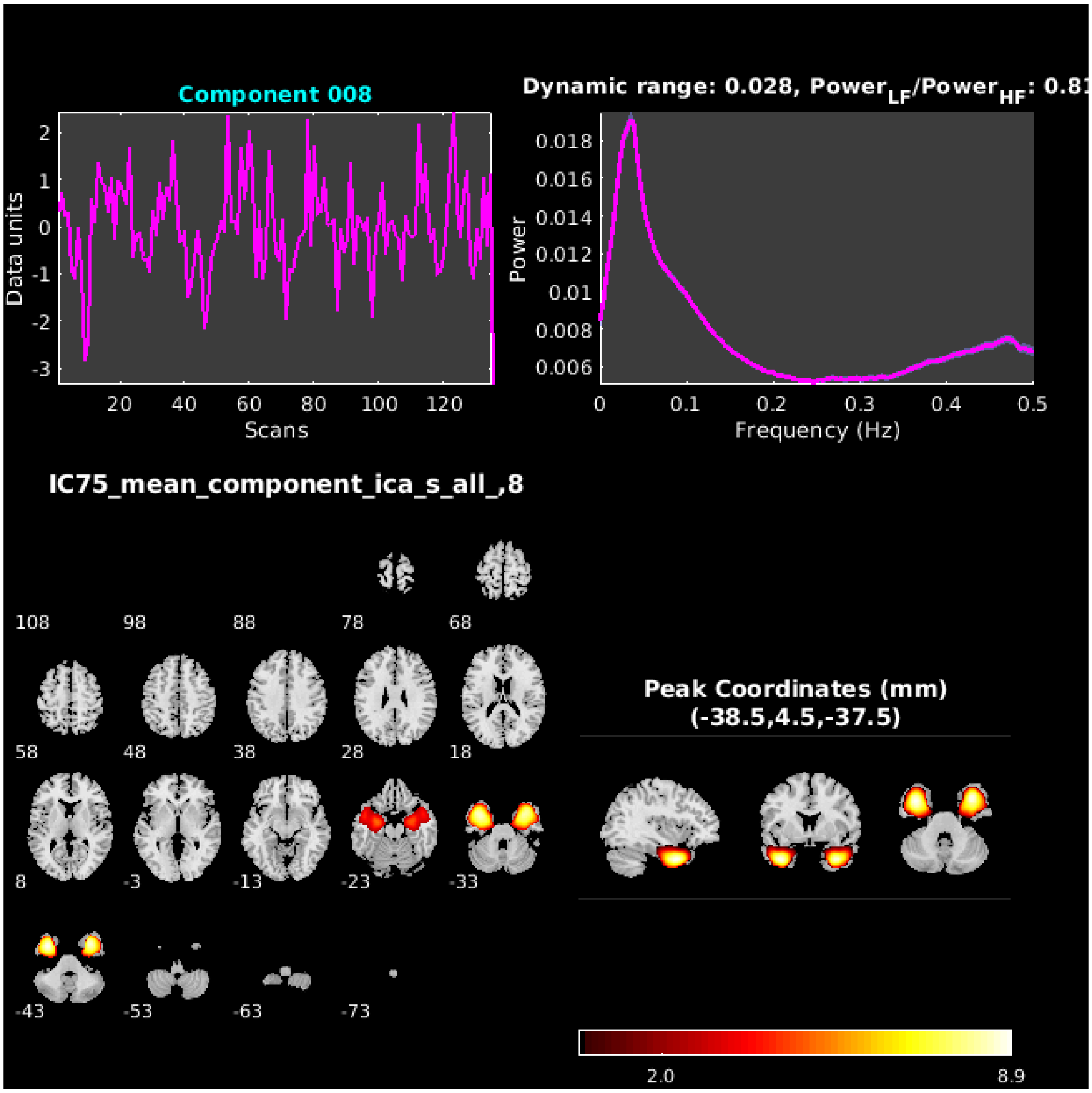
Component 008
Dynamic range: 0.028, Power$_{LF}$/Power$_{HF}$: 0.81
Data units
Scans
Power
Frequency (Hz)
IC75_mean_component_ica_s_all_,8
Peak Coordinates (mm)
(-38.5,4.5,-37.5)
2.0
8.9

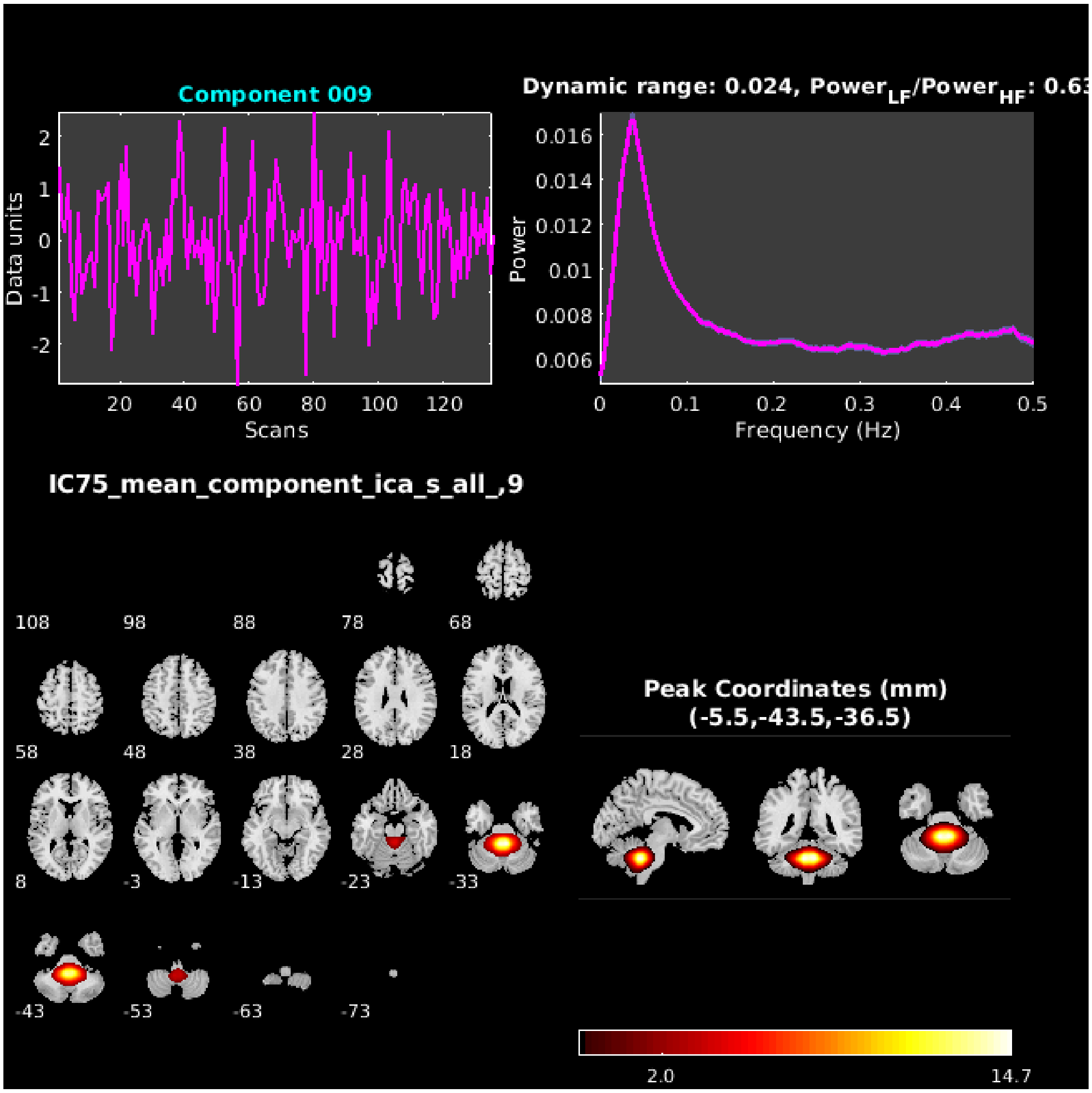
Component 009
Dynamic range: 0.024, Power_LF/Power_HF: 0.6
Data units
Scans
Power
Frequency (Hz)
IC75_mean_component_ica_s_all_,9
108
98
88
78
68
58
48
38
28
18
8
-3
-13
-23
-33
-43
-53
-63
-73
Peak Coordinates (mm)
(-5.5,-43.5,-36.5)
2.0
14.7

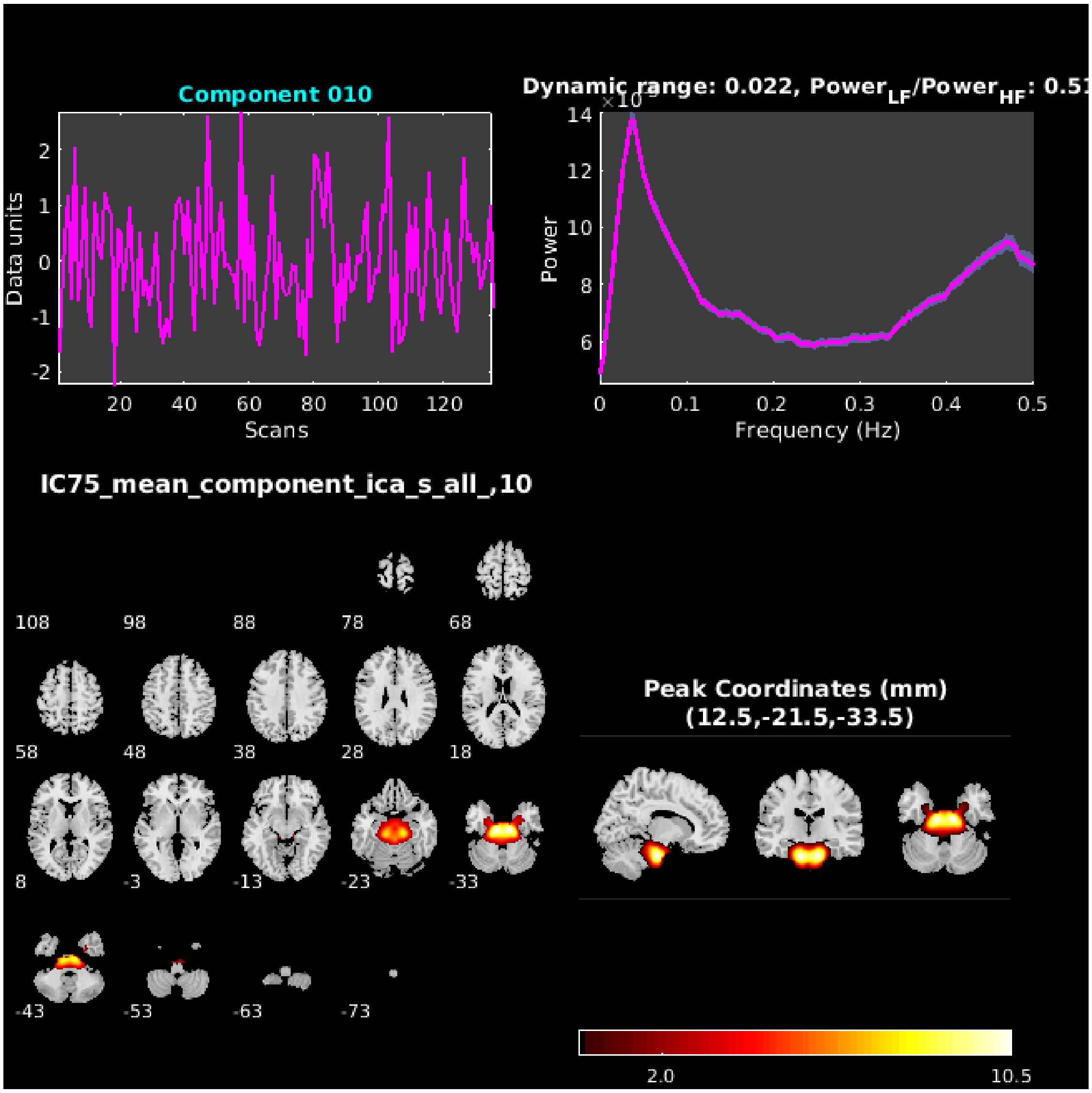

Component 010
Dynamic range: 0.022, Power_LF/Power_HF: 0.51
Data units
Scans
Power
Frequency (Hz)
IC75_mean_component_ica_s_all_,10
108
98
88
78
68
58
48
38
28
18
8
-3
-13
-23
-33
-43
-53
-63
-73
Peak Coordinates (mm)
(12.5,-21.5,-33.5)
2.0
10.5

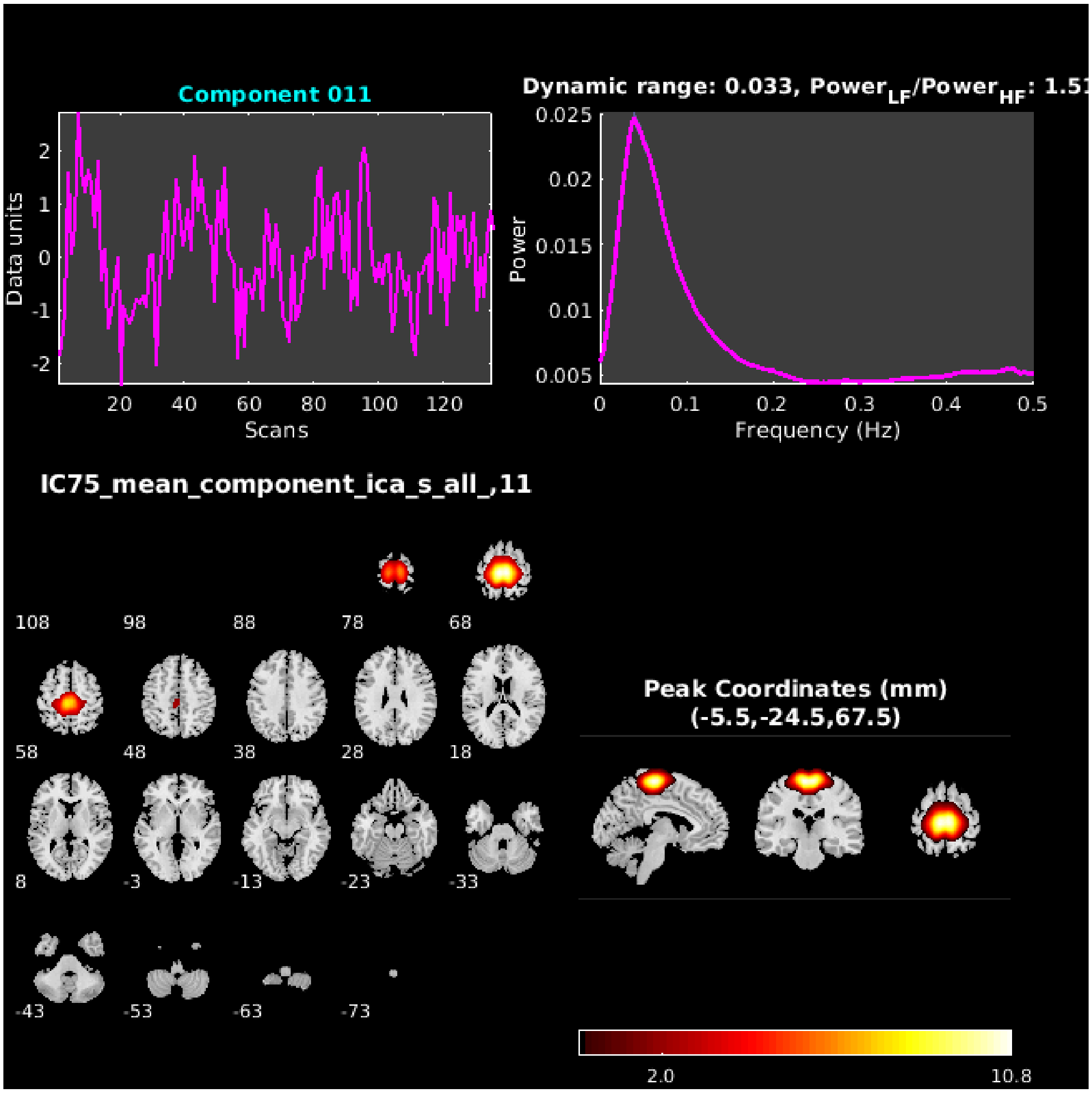

Component 011
Data units
Scans
Dynamic range: 0.033, Power$_{LF}$/Power$_{HF}$: 1.51
Power
Frequency (Hz)
IC75_mean_component_ica_s_all_,11
Peak Coordinates (mm)
(-5.5,-24.5,67.5)
2.0
10.8

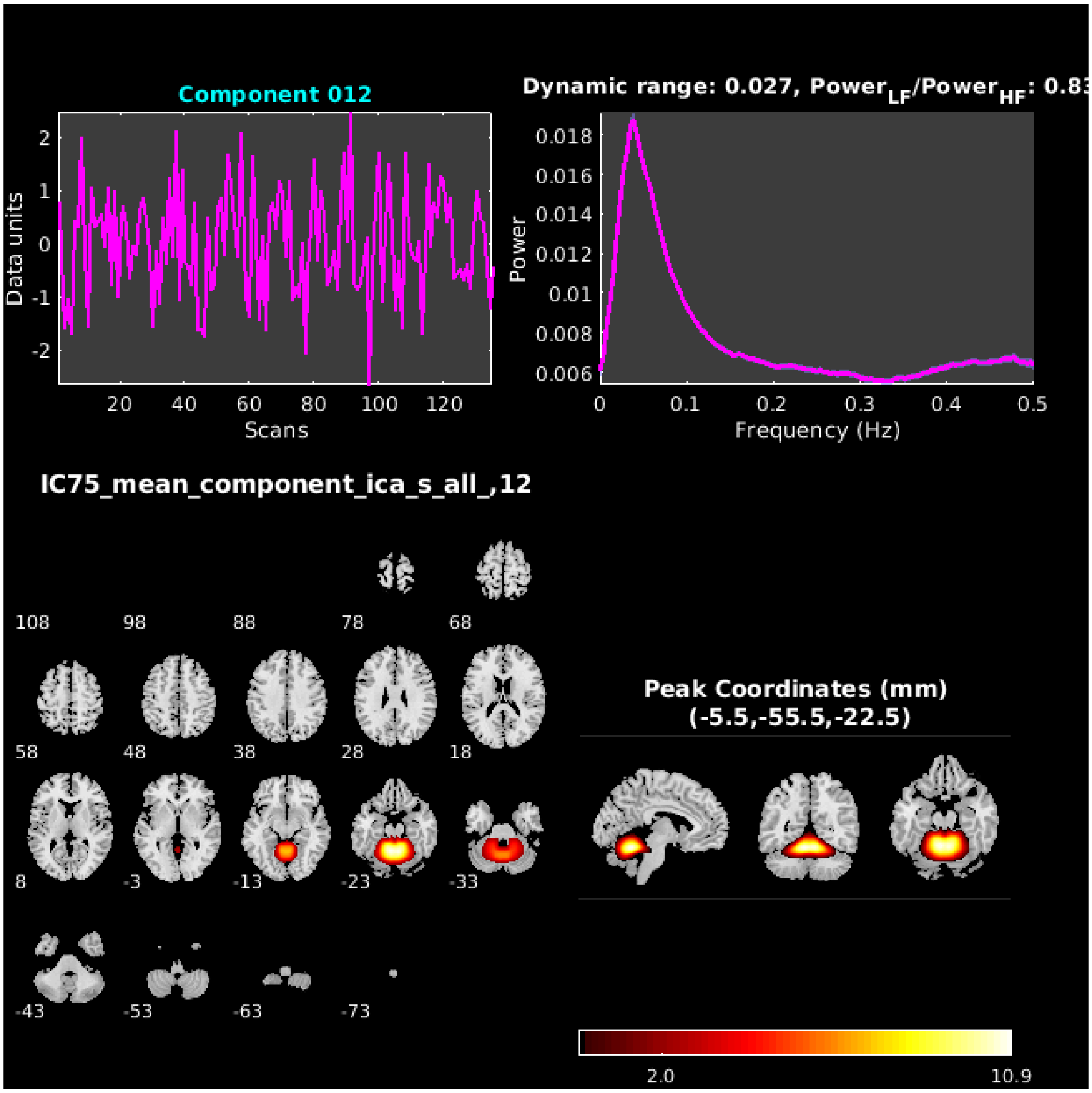

Component 012
Dynamic range: 0.027, Power$_{LF}$/Power$_{HF}$: 0.83
Data units
Scans
Power
Frequency (Hz)
IC75_mean_component_ica_s_all_,12
Peak Coordinates (mm)
(-5.5,-55.5,-22.5)
2.0
10.9

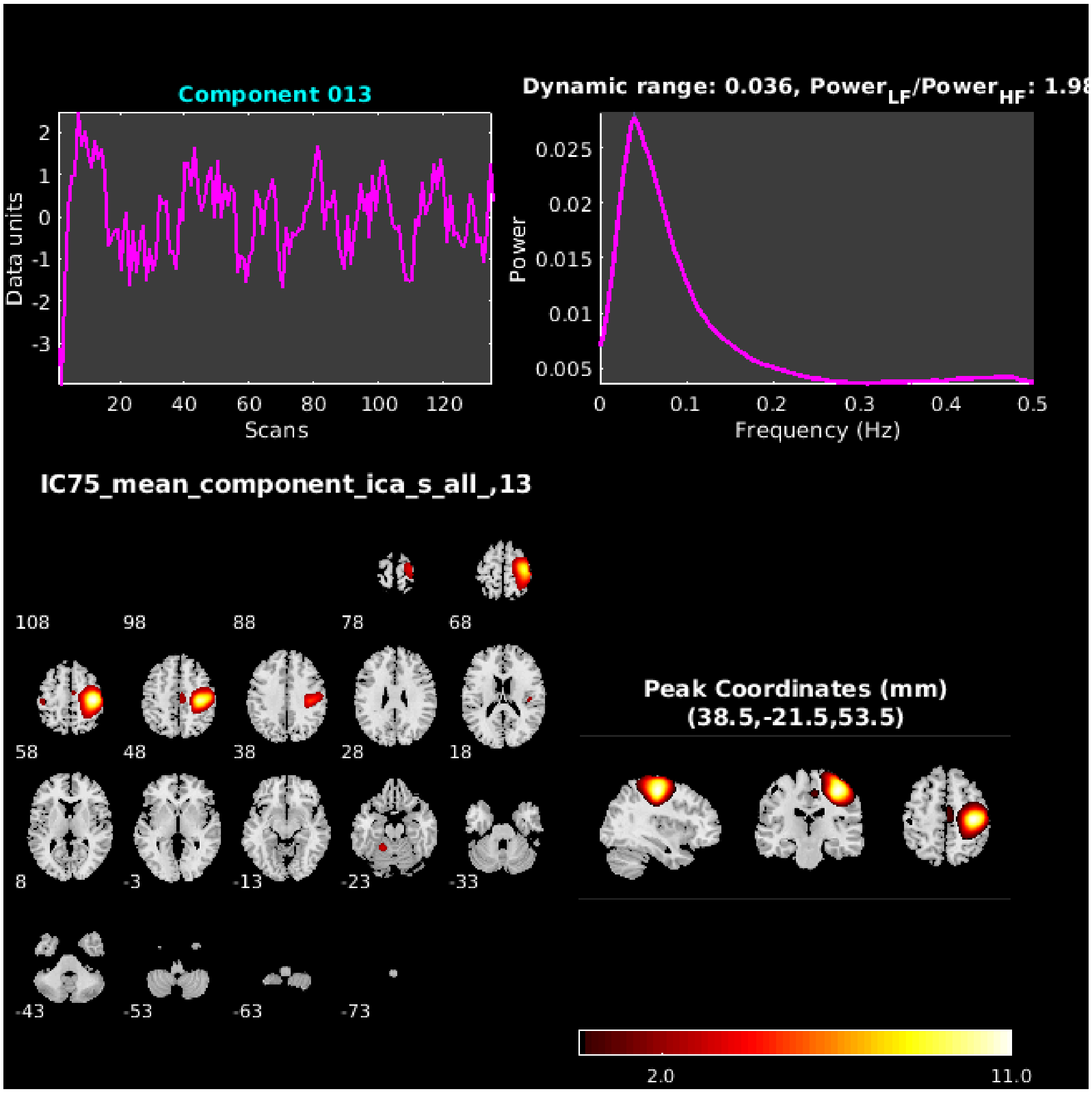

Component 013
Dynamic range: 0.036, Power$_{LF}$/Power$_{HF}$: 1.98
Data units
Scans
Power
Frequency (Hz)
IC75_mean_component_ica_s_all_,13
Peak Coordinates (mm)
(38.5,-21.5,53.5)
2.0
11.0

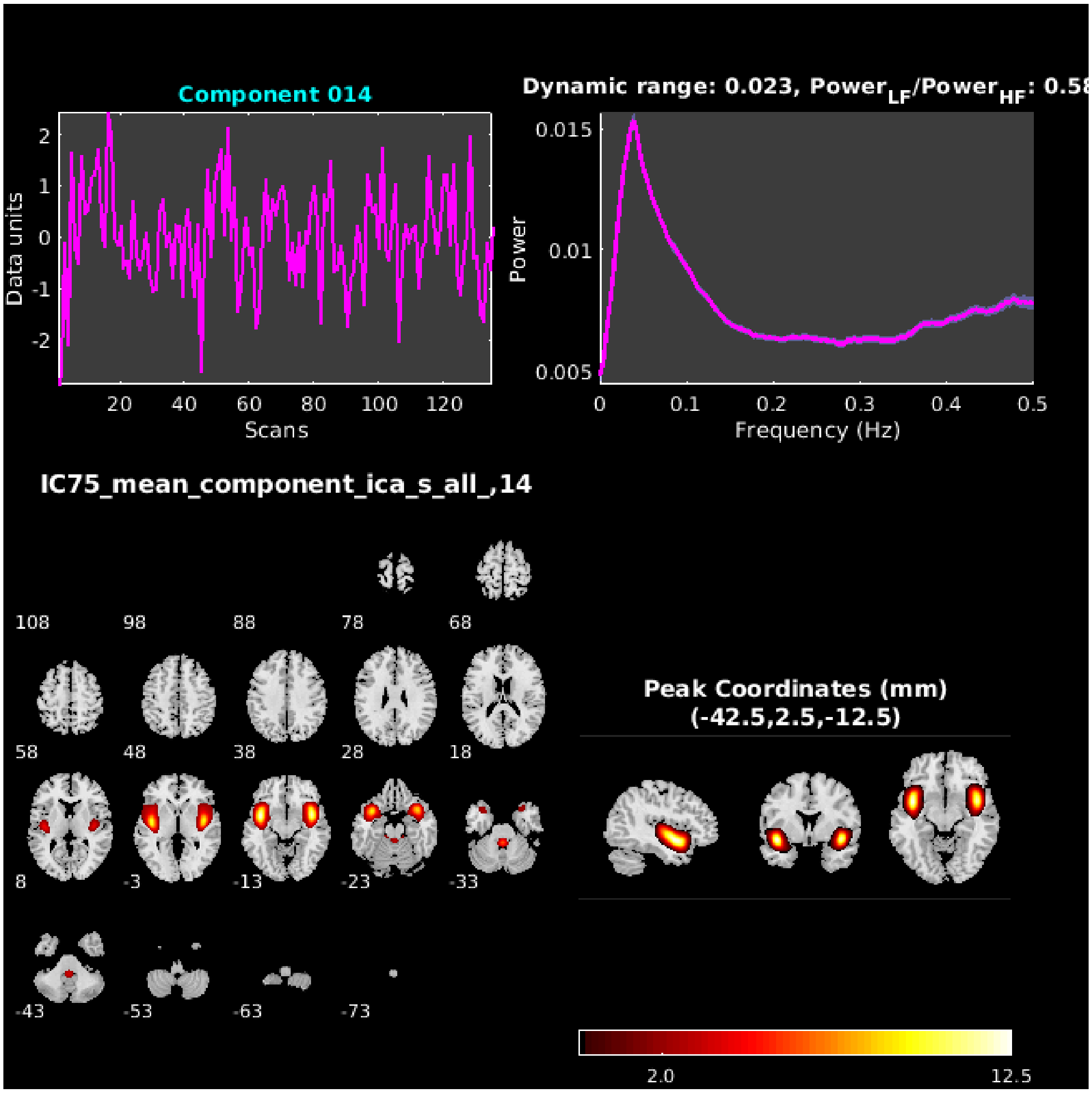

Component 014
Dynamic range: 0.023, Power$_{LF}$/Power$_{HF}$: 0.58
Data units
Scans
Power
Frequency (Hz)
IC75_mean_component_ica_s_all_,14
Peak Coordinates (mm)
(-42.5,2.5,-12.5)
2.0
12.5

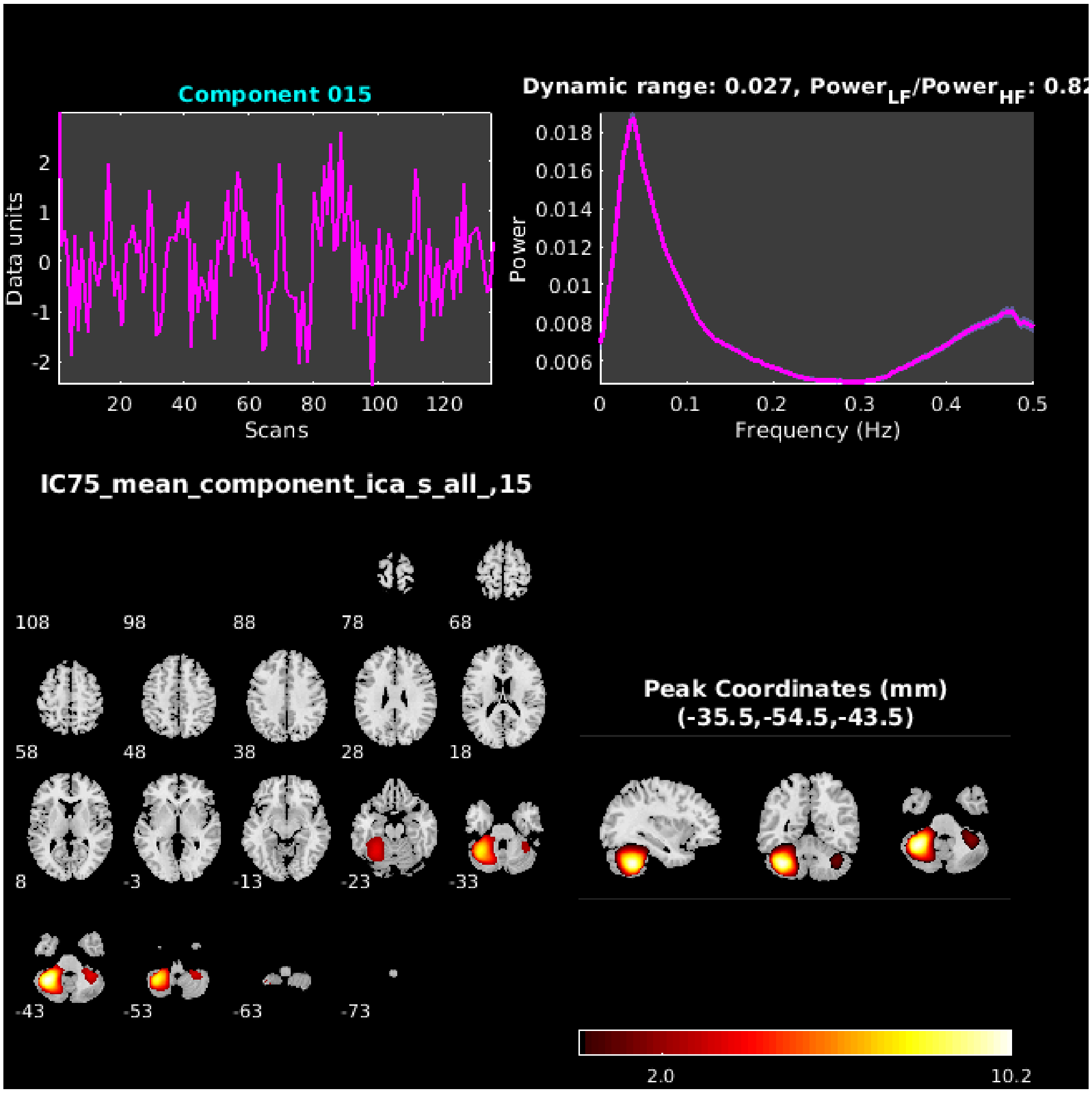
Component 015
Dynamic range: 0.027, Power_LF/Power_HF: 0.82
Data units
Scans
Power
Frequency (Hz)
IC75_mean_component_ica_s_all_,15
Peak Coordinates (mm)
(-35.5,-54.5,-43.5)
2.0
10.2

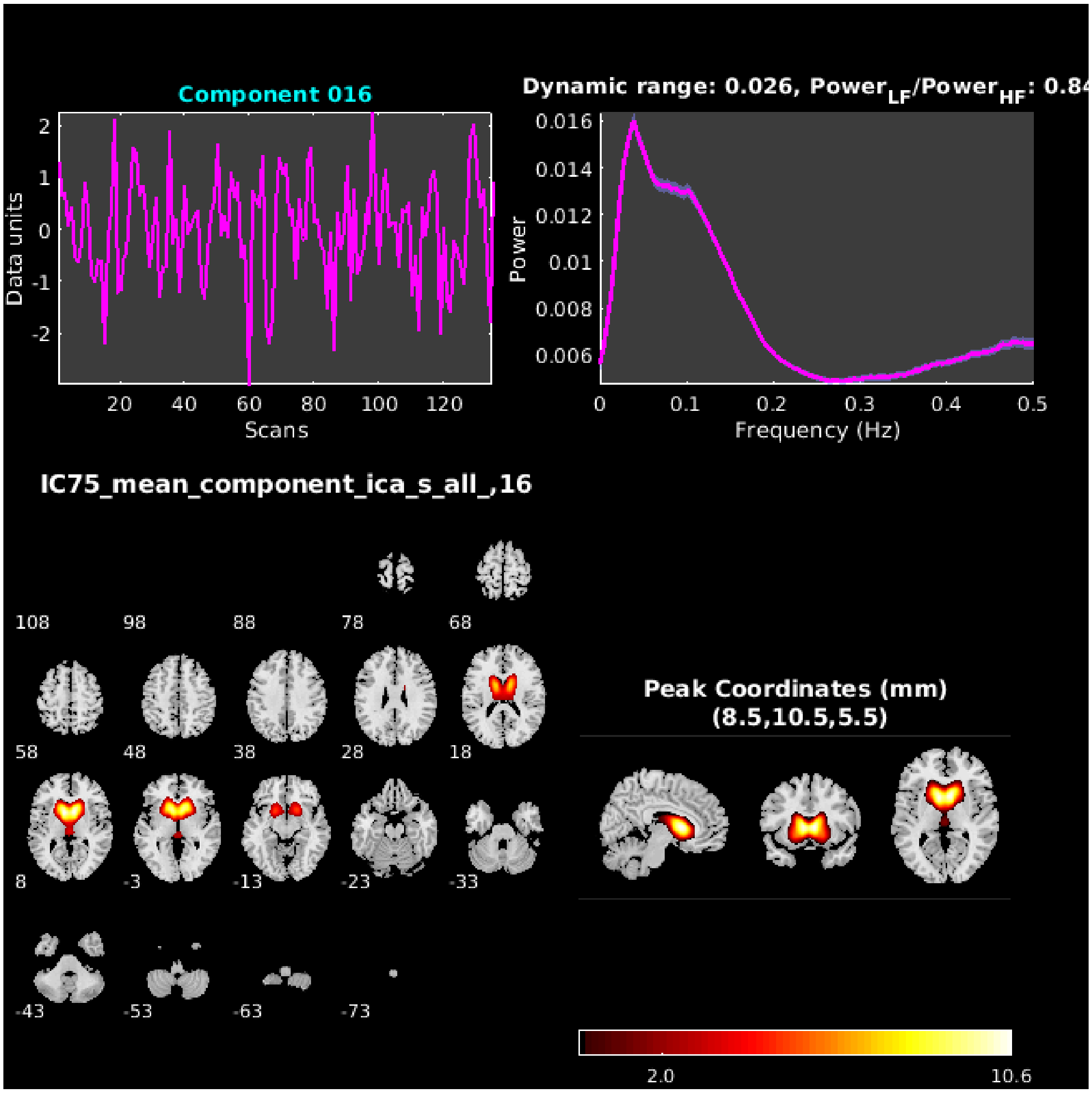
Component 016
Dynamic range: 0.026, Power$_{LF}$/Power$_{HF}$: 0.84
Data units
Scans
Power
Frequency (Hz)
IC75_mean_component_ica_s_all_,16
Peak Coordinates (mm)
(8.5,10.5,5.5)
2.0
10.6

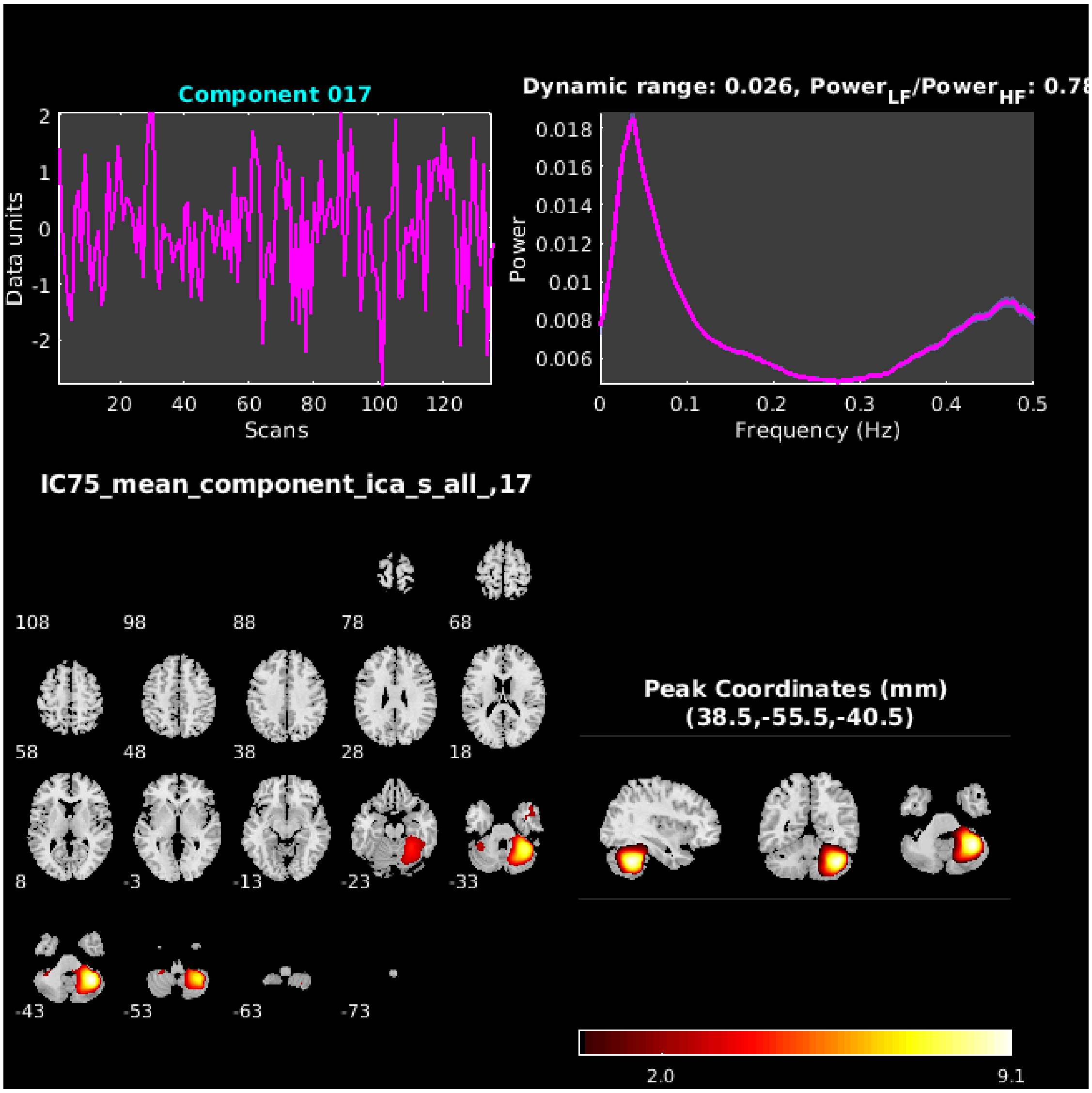
Component 017
Dynamic range: 0.026, Power$_{LF}$/Power$_{HF}$: 0.78
Data units
Scans
Power
Frequency (Hz)
IC75_mean_component_ica_s_all_,17
Peak Coordinates (mm)
(38.5,-55.5,-40.5)

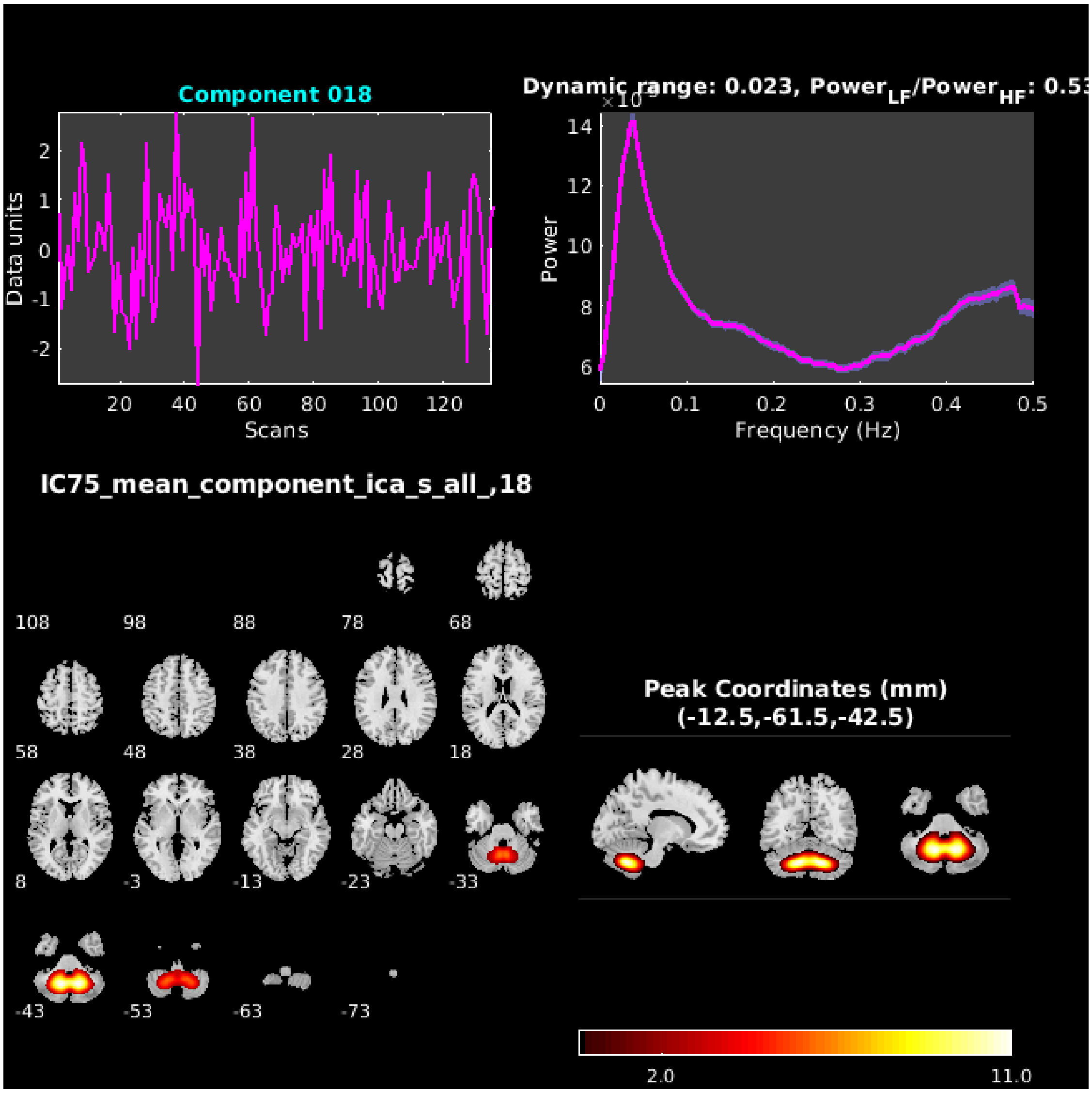
Component 018
Dynamic range: 0.023, Power$_{LF}$/Power$_{HF}$: 0.53
Data units
Scans
Power
Frequency (Hz)
IC75_mean_component_ica_s_all_,18
Peak Coordinates (mm)
(-12.5,-61.5,-42.5)
2.0
11.0

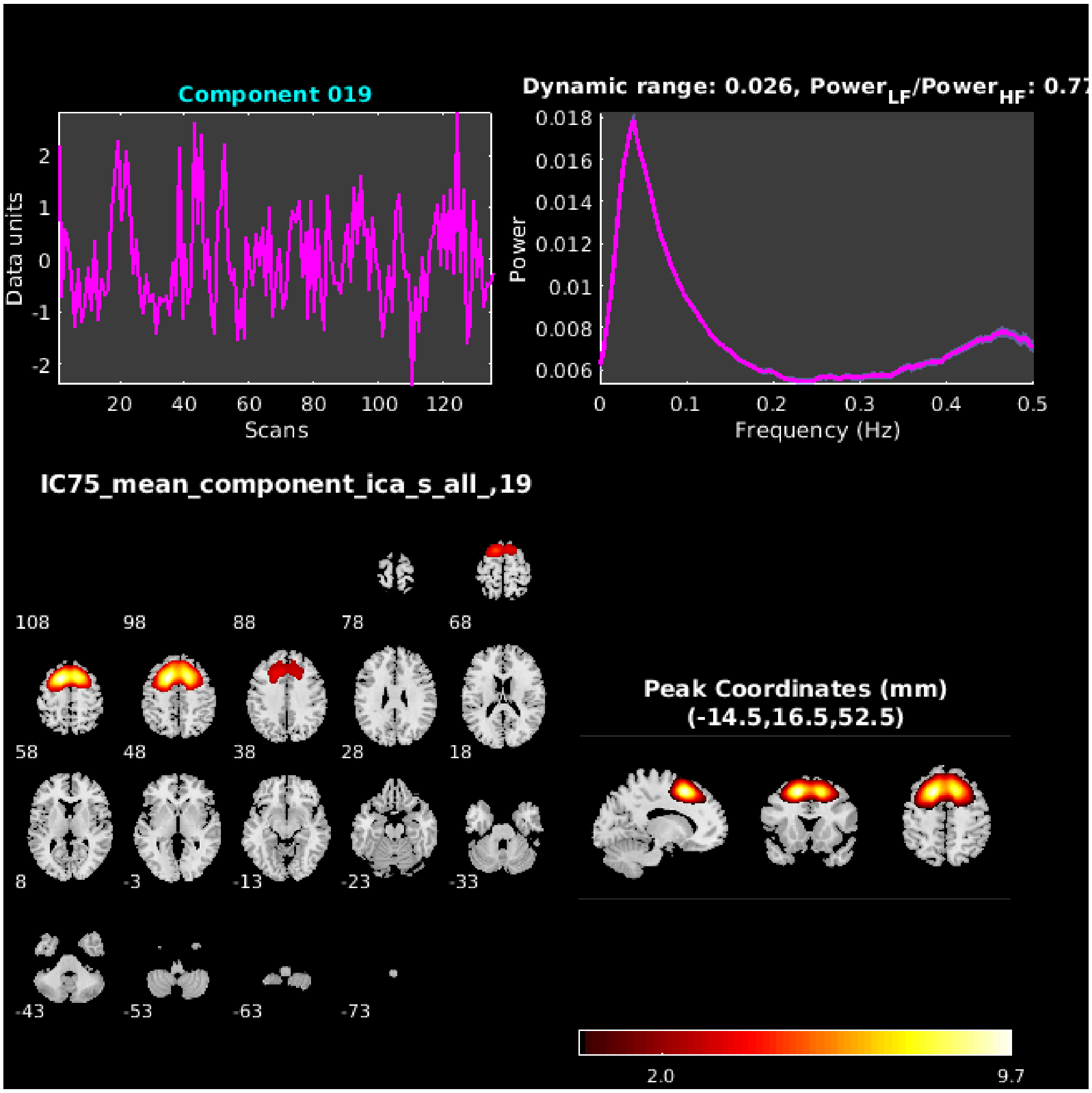

Component 019
Dynamic range: 0.026, Power_LF/Power_HF: 0.77
Data units
Scans
Power
Frequency (Hz)
IC75_mean_component_ica_s_all_,19
108
98
88
78
68
58
48
38
28
18
8
-3
-13
-23
-33
-43
-53
-63
-73
Peak Coordinates (mm)
(-14.5,16.5,52.5)
2.0
9.7

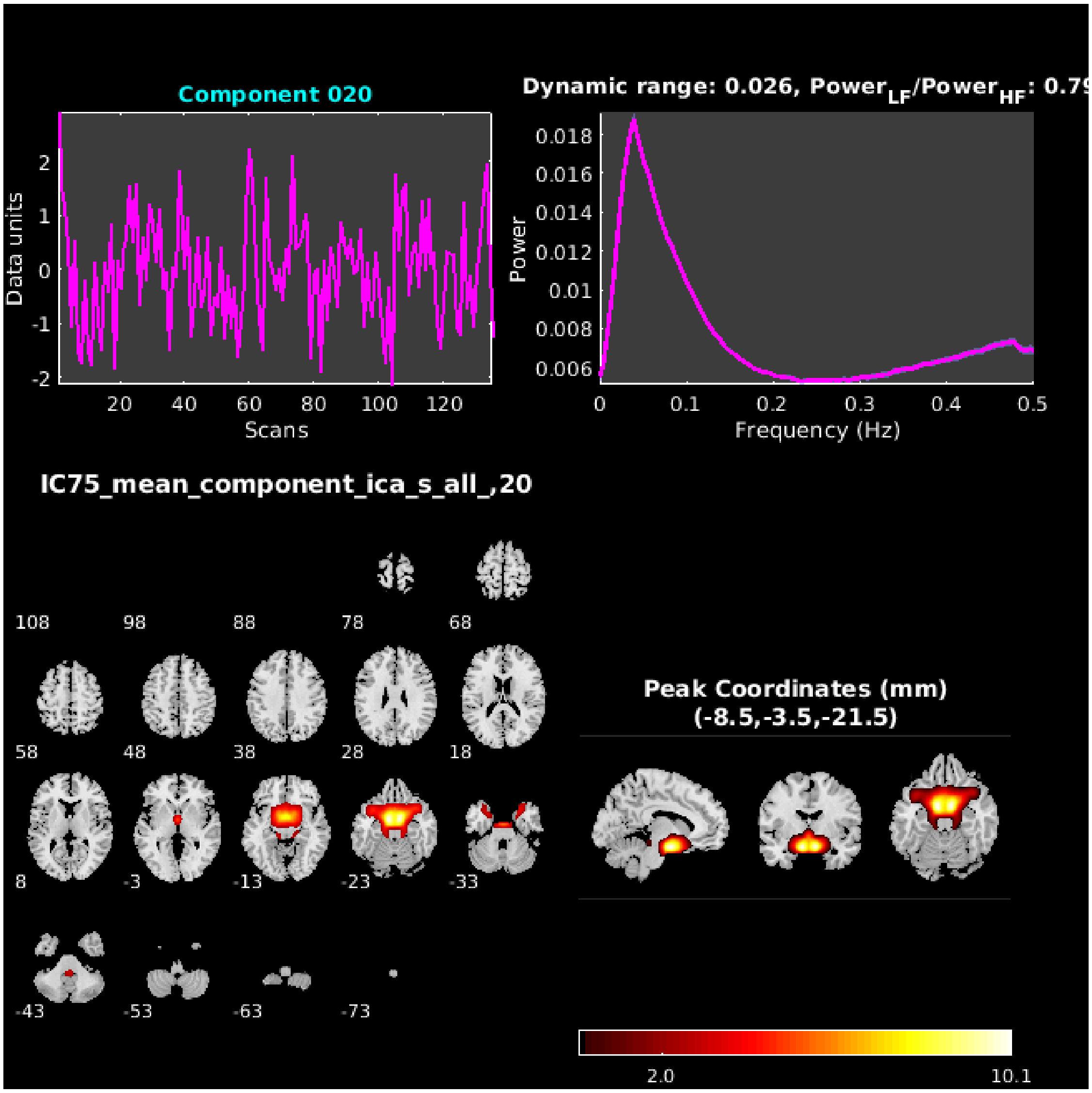
Component 020
Dynamic range: 0.026, Power_LF/Power_HF: 0.79
Data units
Scans
Power
Frequency (Hz)
IC75_mean_component_ica_s_all_,20
Peak Coordinates (mm)
(-8.5,-3.5,-21.5)
2.0
10.1

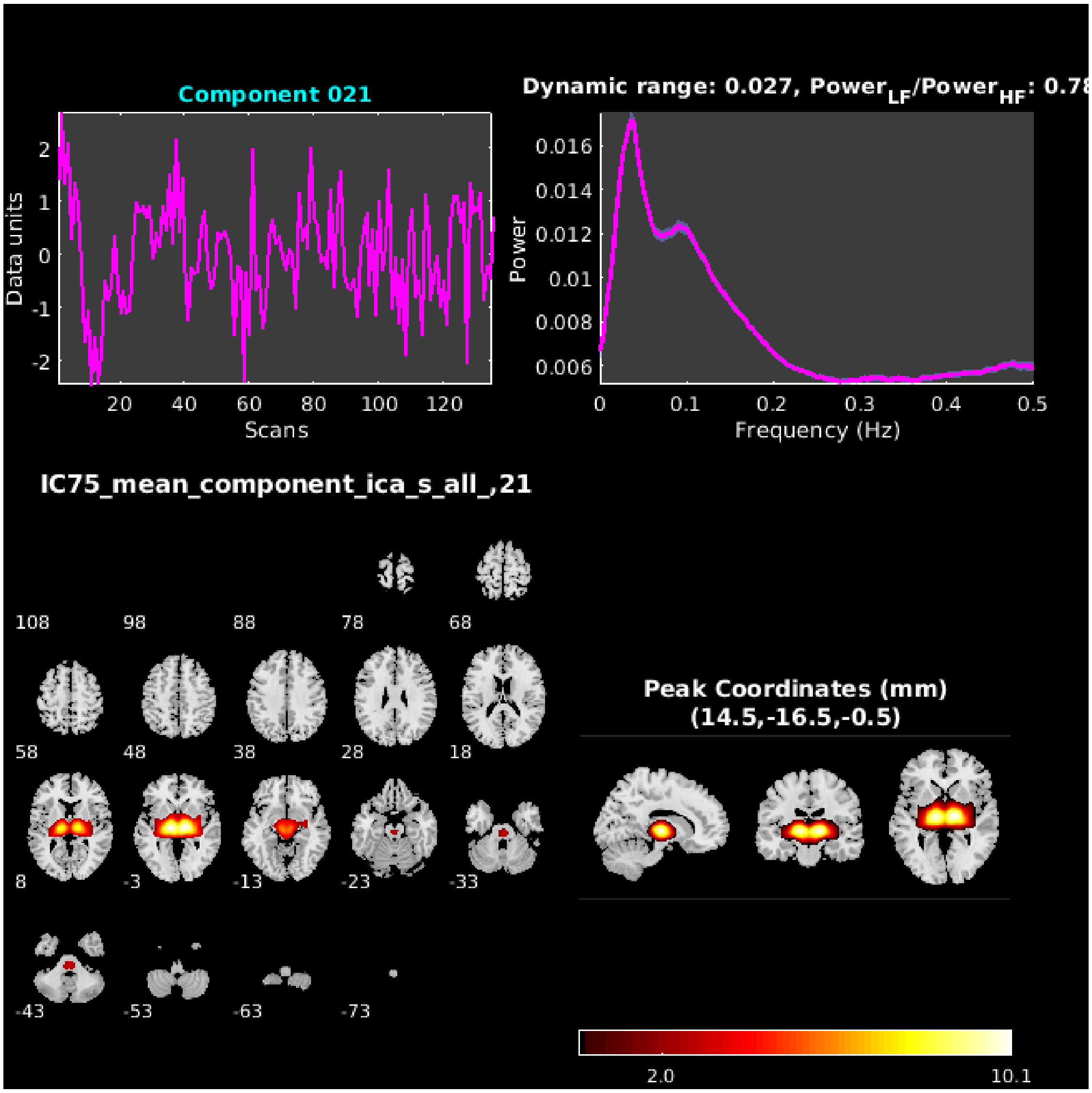

Component 021
Dynamic range: 0.027, Power$_{LF}$/Power$_{HF}$: 0.78
Data units
Scans
Power
Frequency (Hz)
IC75_mean_component_ica_s_all_,21
Peak Coordinates (mm)
(14.5,-16.5,-0.5)
2.0
10.1

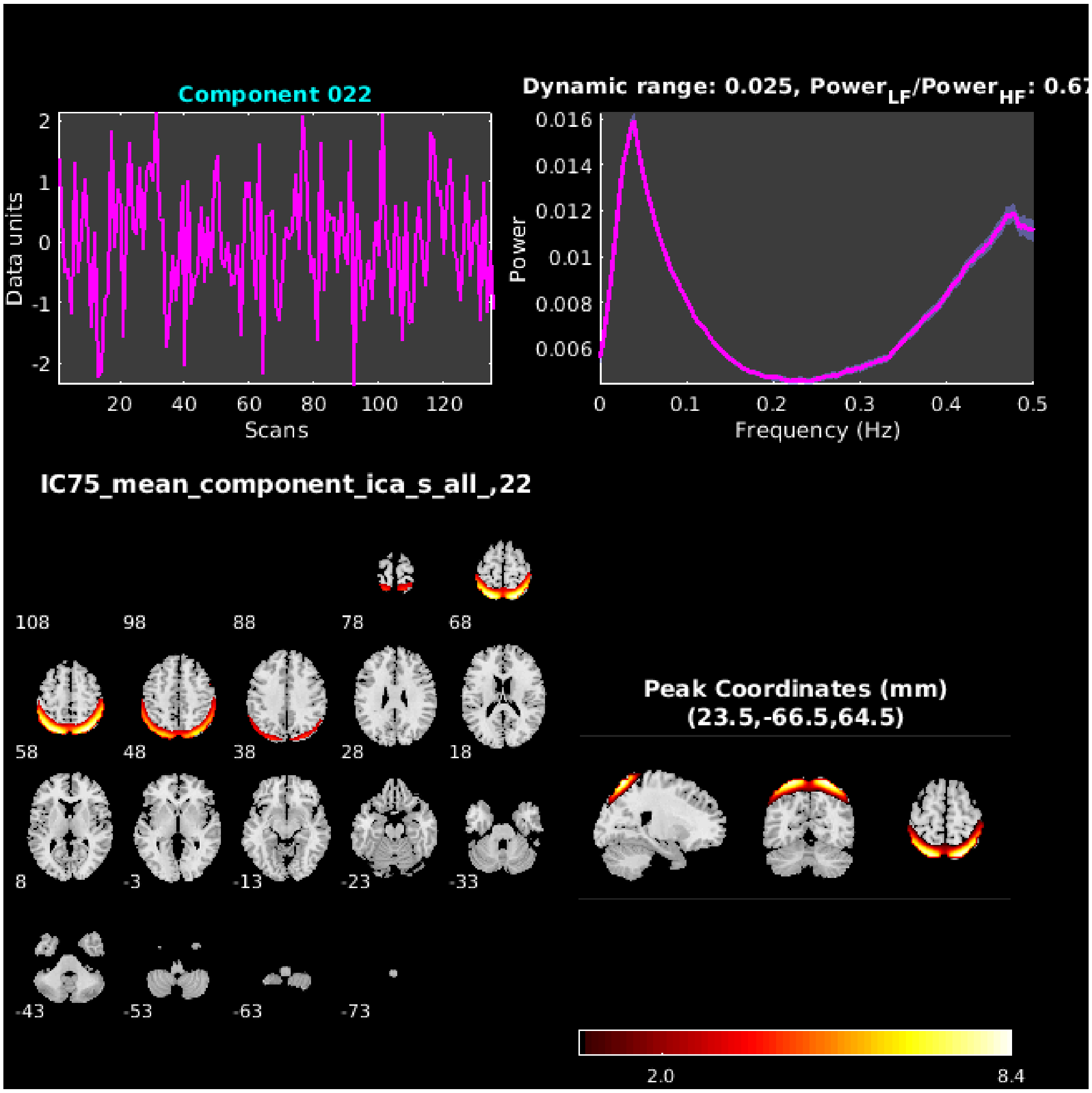

Component 022
Dynamic range: 0.025, Power_LF/Power_HF: 0.67
Data units
Scans
Power
Frequency (Hz)
IC75_mean_component_ica_s_all_,22
Peak Coordinates (mm)
(23.5,-66.5,64.5)
2.0
8.4

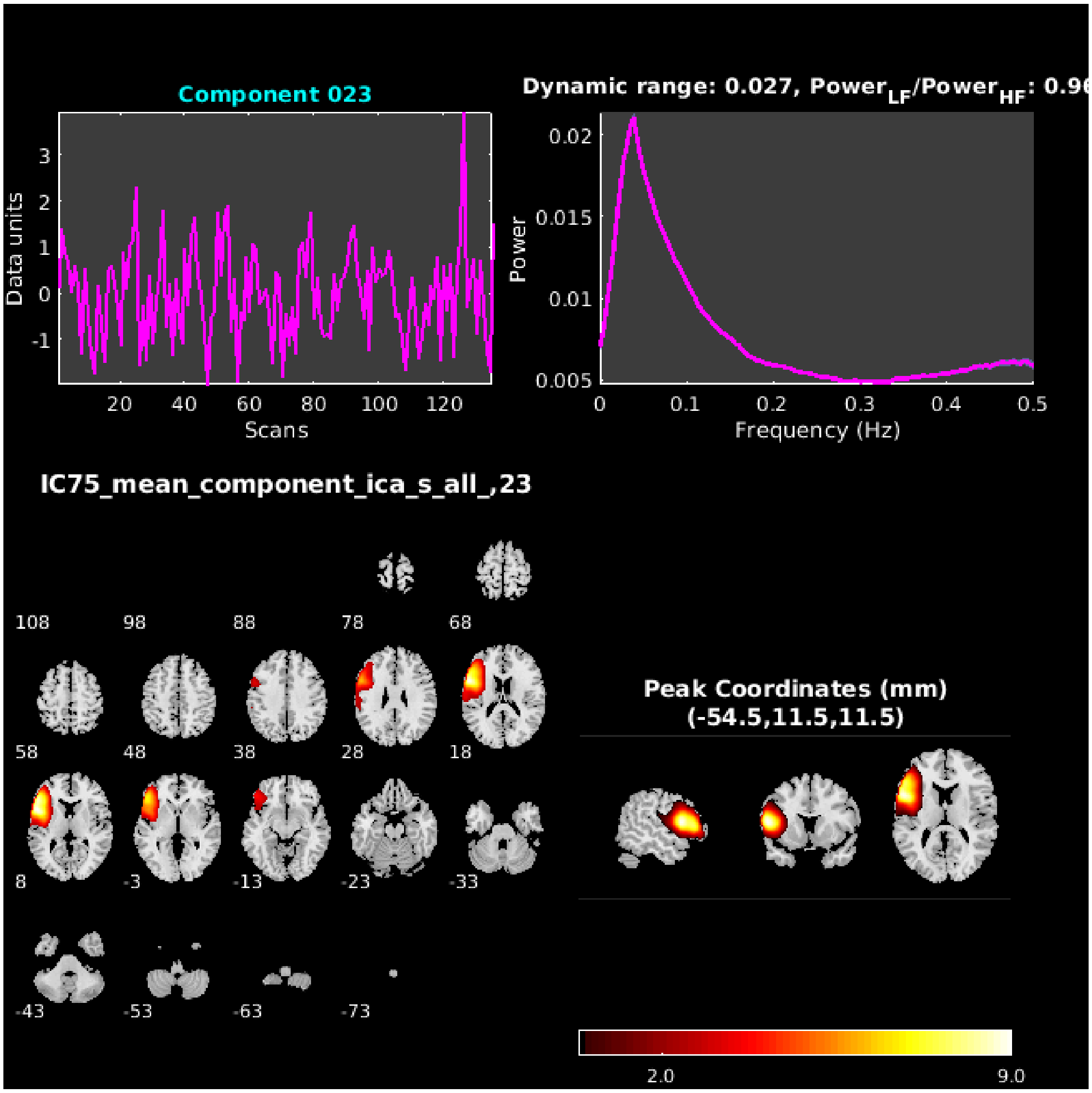
Component 023
Dynamic range: 0.027, Power$_{LF}$/Power$_{HF}$: 0.96
Data units
Scans
Power
Frequency (Hz)
IC75_mean_component_ica_s_all_,23
Peak Coordinates (mm)
(-54.5,11.5,11.5)
2.0
9.0

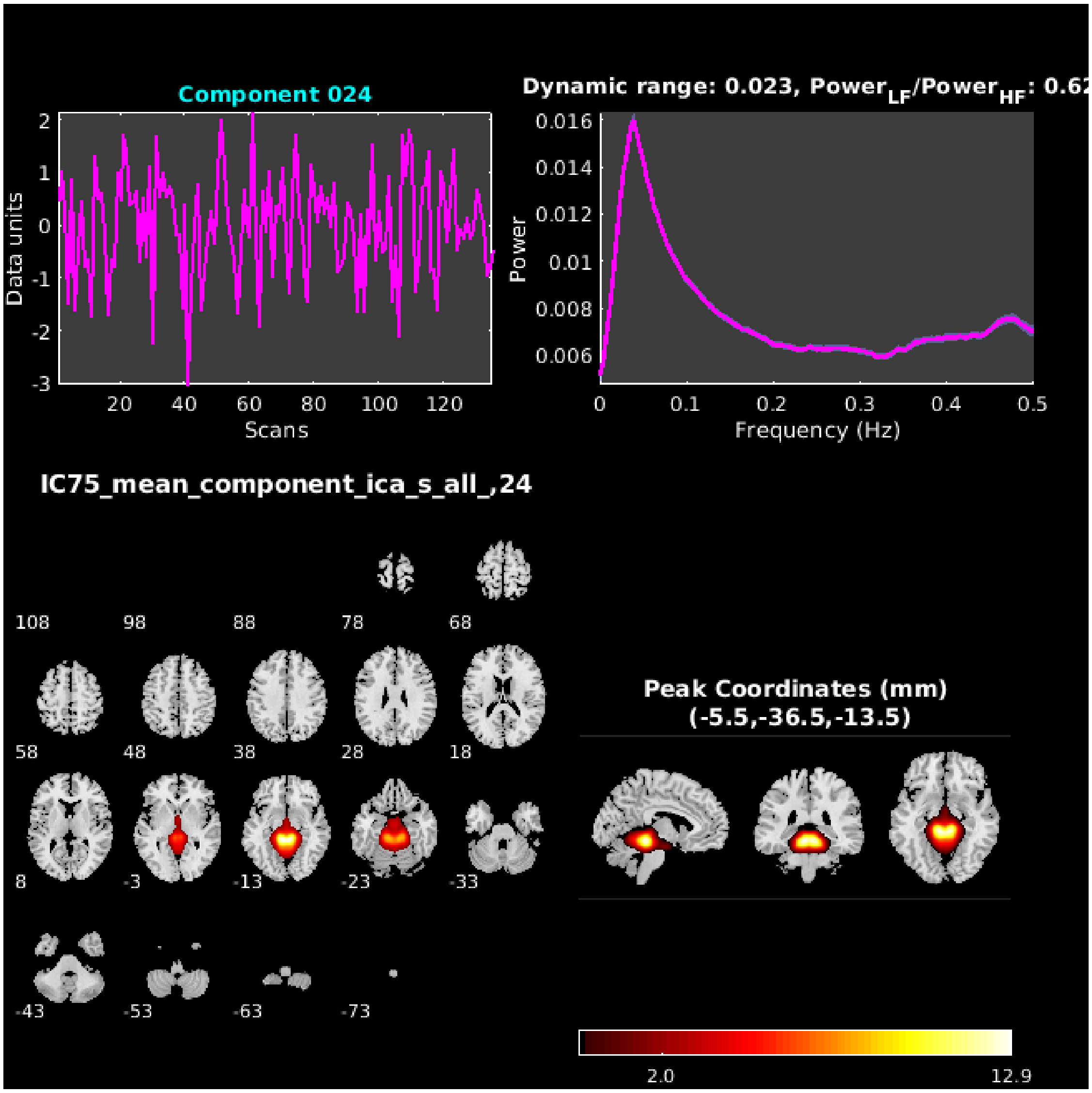

Component 024
Dynamic range: 0.023, Power$_{LF}$/Power$_{HF}$: 0.62
Data units
Scans
Power
Frequency (Hz)
IC75_mean_component_ica_s_all_,24
108
98
88
78
68
58
48
38
28
18
8
-3
-13
-23
-33
-43
-53
-63
-73
Peak Coordinates (mm)
(-5.5,-36.5,-13.5)
2.0
12.9

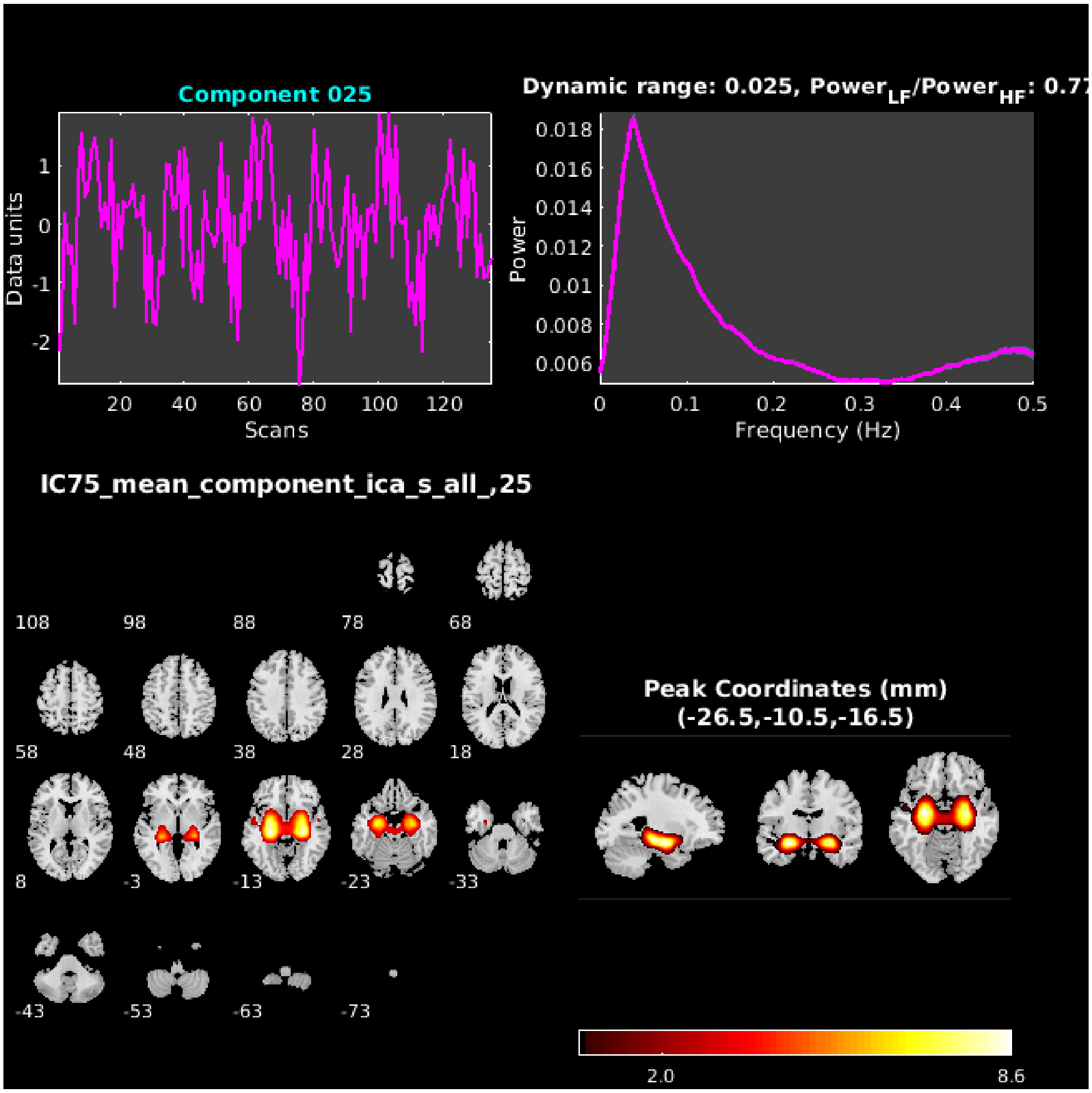
Component 025
Dynamic range: 0.025, Power_LF/Power_HF: 0.77
Data units
Scans
Power
Frequency (Hz)
IC75_mean_component_ica_s_all_,25
Peak Coordinates (mm)
(-26.5,-10.5,-16.5)
2.0
8.6

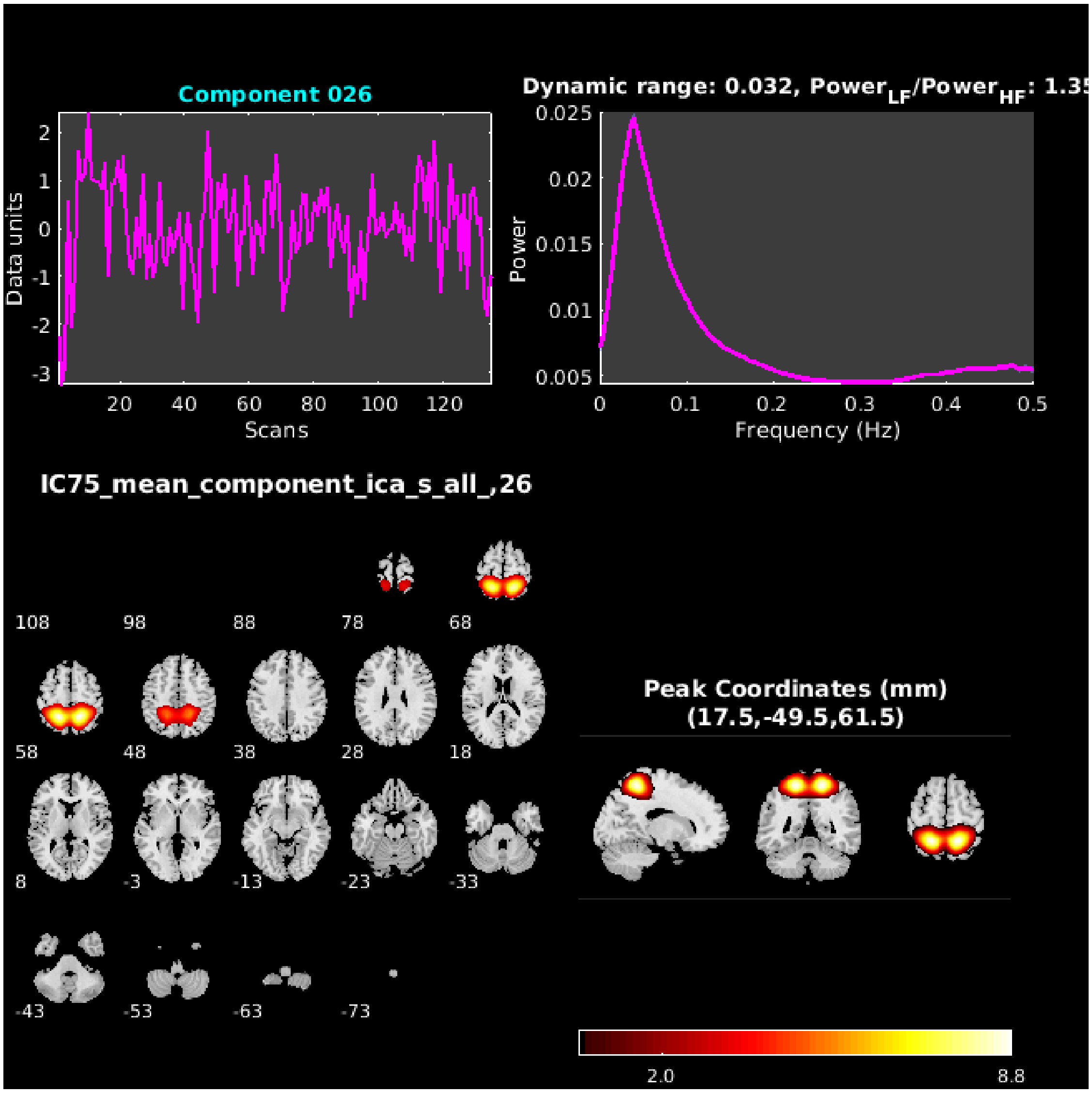
Component 026
Dynamic range: 0.032, Power$_{LF}$/Power$_{HF}$: 1.35
Data units
Scans
Power
Frequency (Hz)
IC75_mean_component_ica_s_all_,26
Peak Coordinates (mm)
(17.5,-49.5,61.5)
2.0
8.8

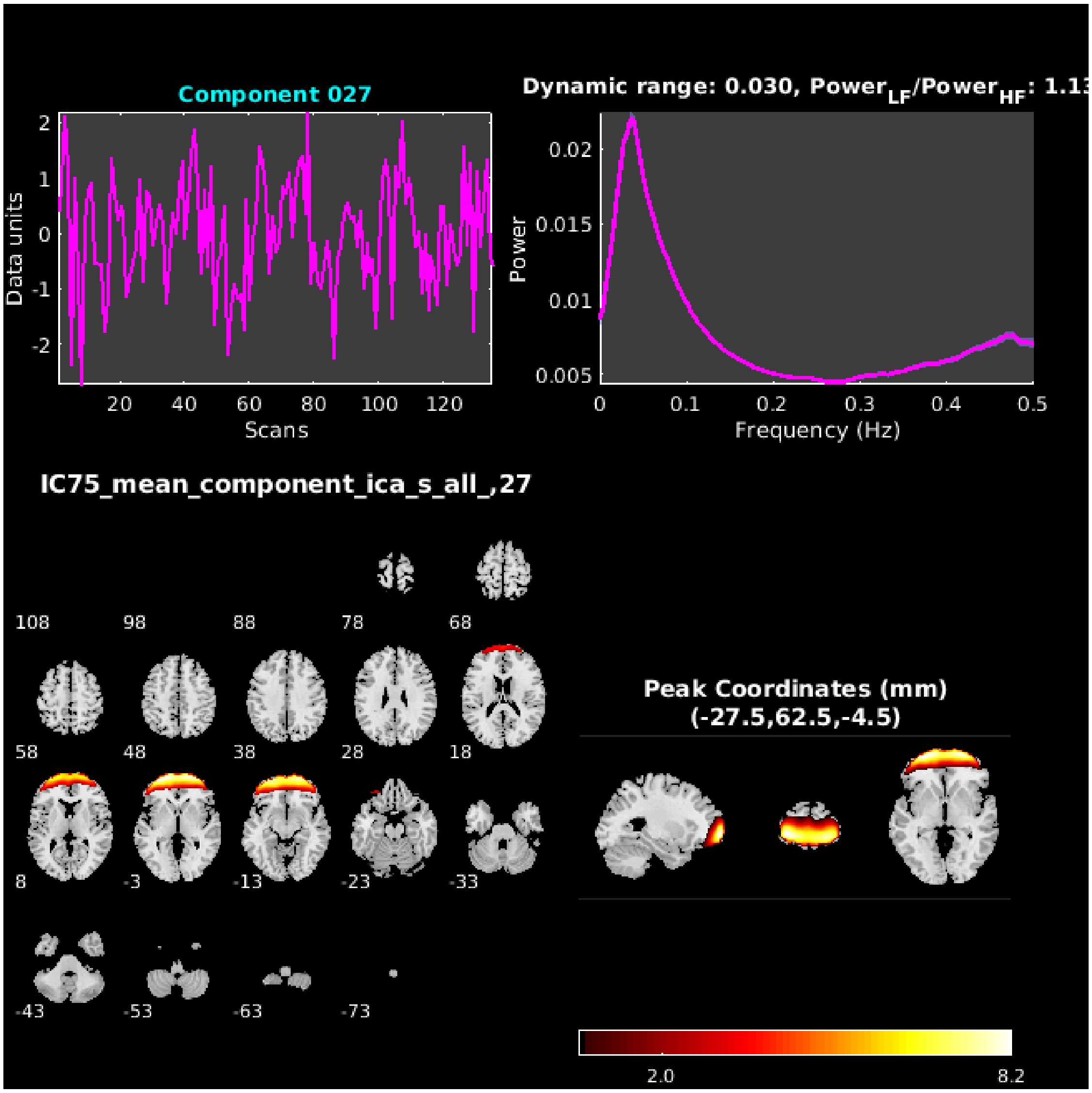

Component 027
Dynamic range: 0.030, Power_LF/Power_HF: 1.13
Data units
Scans
Power
Frequency (Hz)
IC75_mean_component_ica_s_all_,27
Peak Coordinates (mm)
(-27.5,62.5,-4.5)

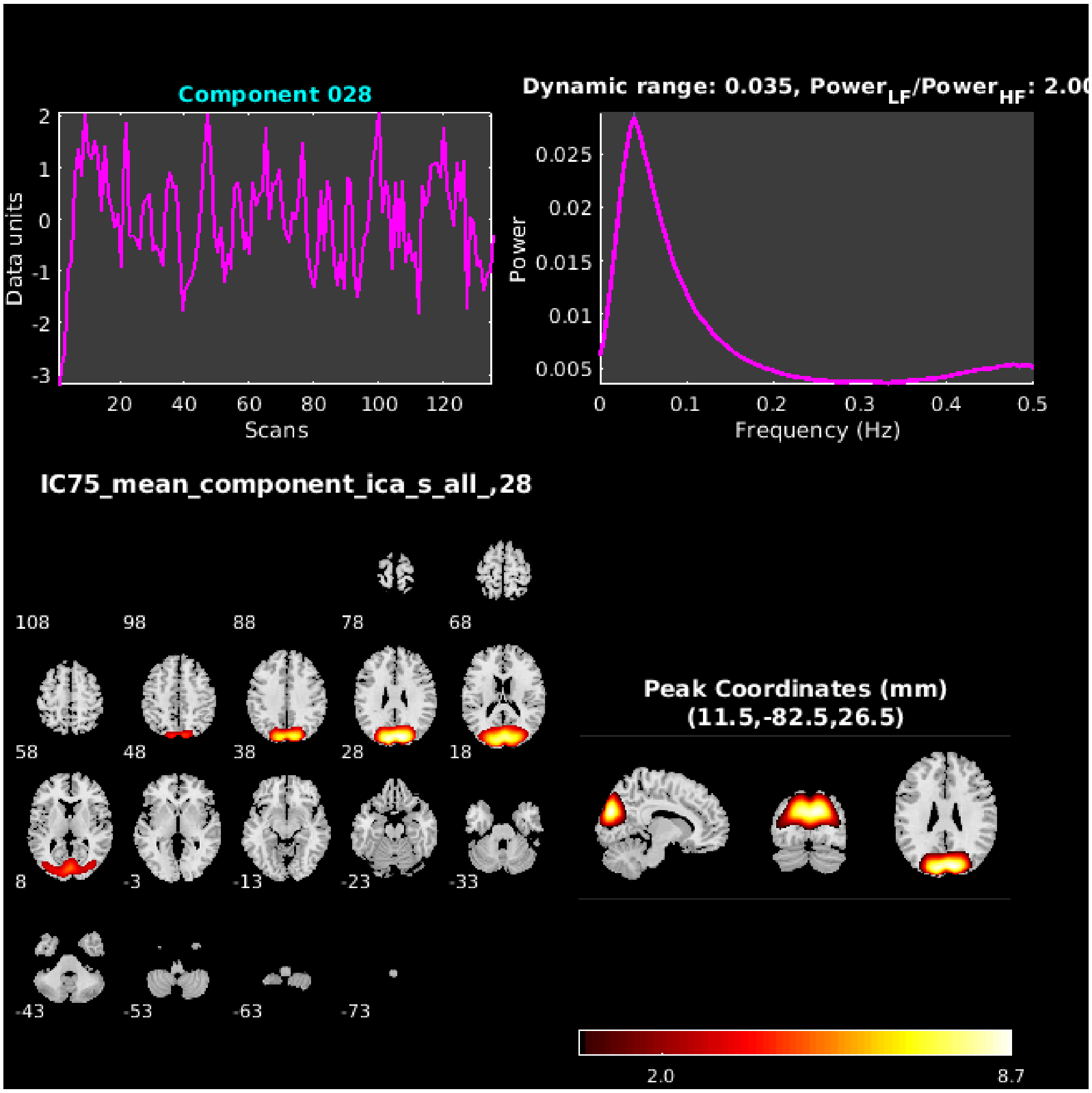
Component 028
Dynamic range: 0.035, Power_LF/Power_HF: 2.0
Data units
Scans
Power
Frequency (Hz)
IC75_mean_component_ica_s_all_,28
108
98
88
78
68
58
48
38
28
18
8
-3
-13
-23
-33
-43
-53
-63
-73
Peak Coordinates (mm)
(11.5,-82.5,26.5)
2.0
8.7

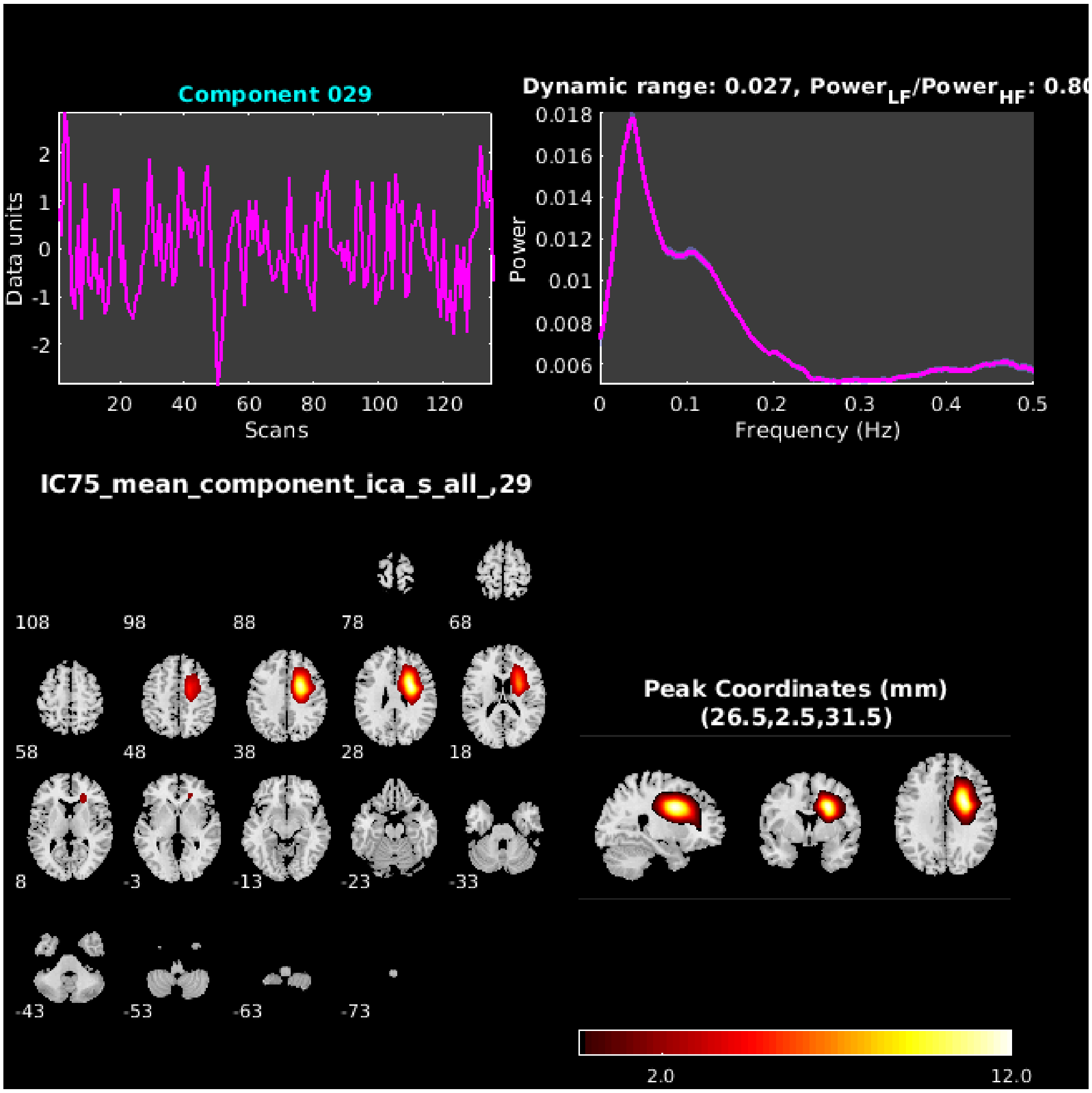

Component 029
Dynamic range: 0.027, Power$_{LF}$/Power$_{HF}$: 0.80
Data units
Scans
Power
Frequency (Hz)
IC75_mean_component_ica_s_all_,29
Peak Coordinates (mm)
(26.5,2.5,31.5)
2.0
12.0

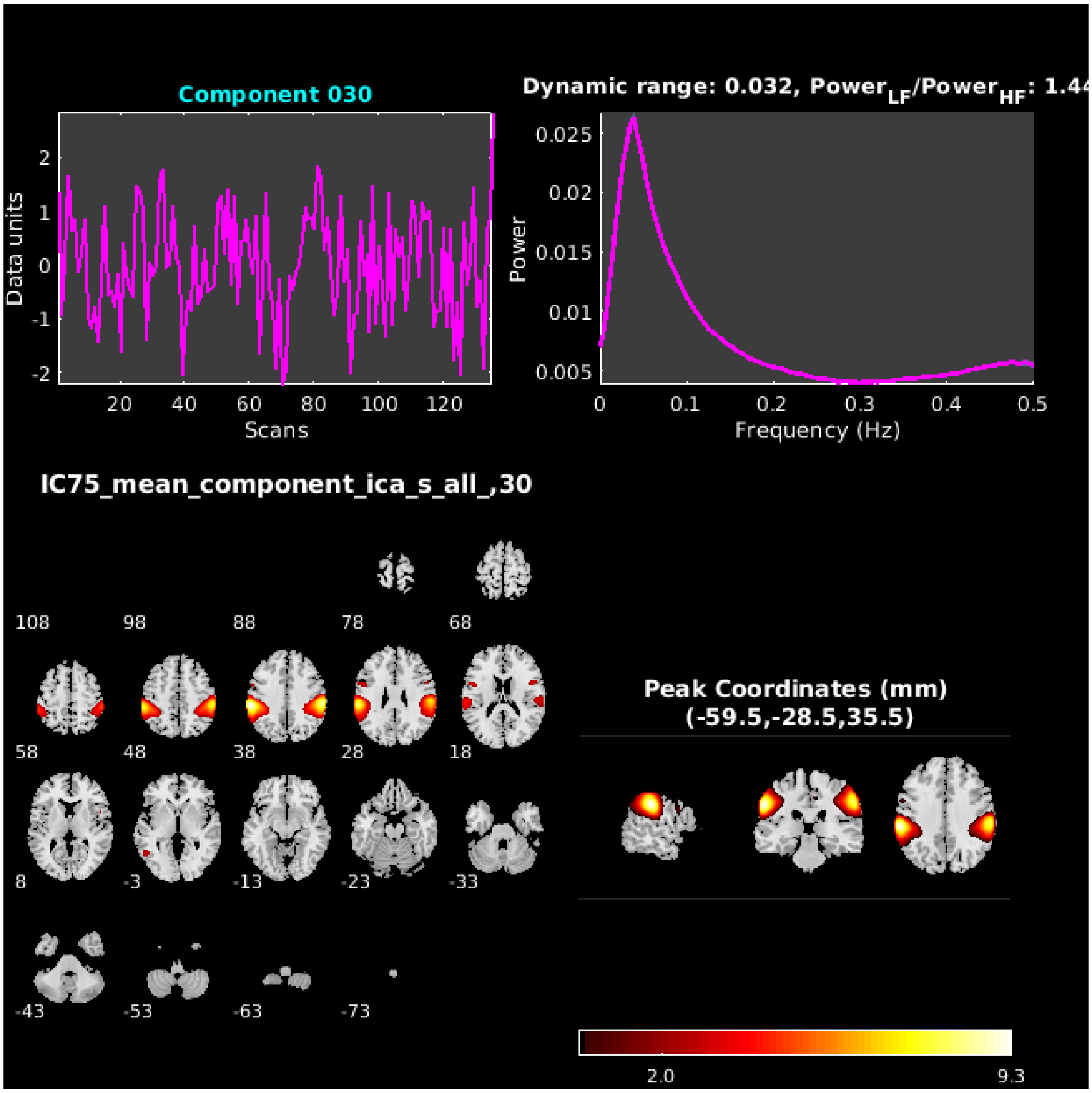

Component 030
Dynamic range: 0.032, Power_LF/Power_HF: 1.44
Data units
Scans
Power
Frequency (Hz)
IC75_mean_component_ica_s_all_,30
Peak Coordinates (mm)
(-59.5,-28.5,35.5)
2.0
9.3

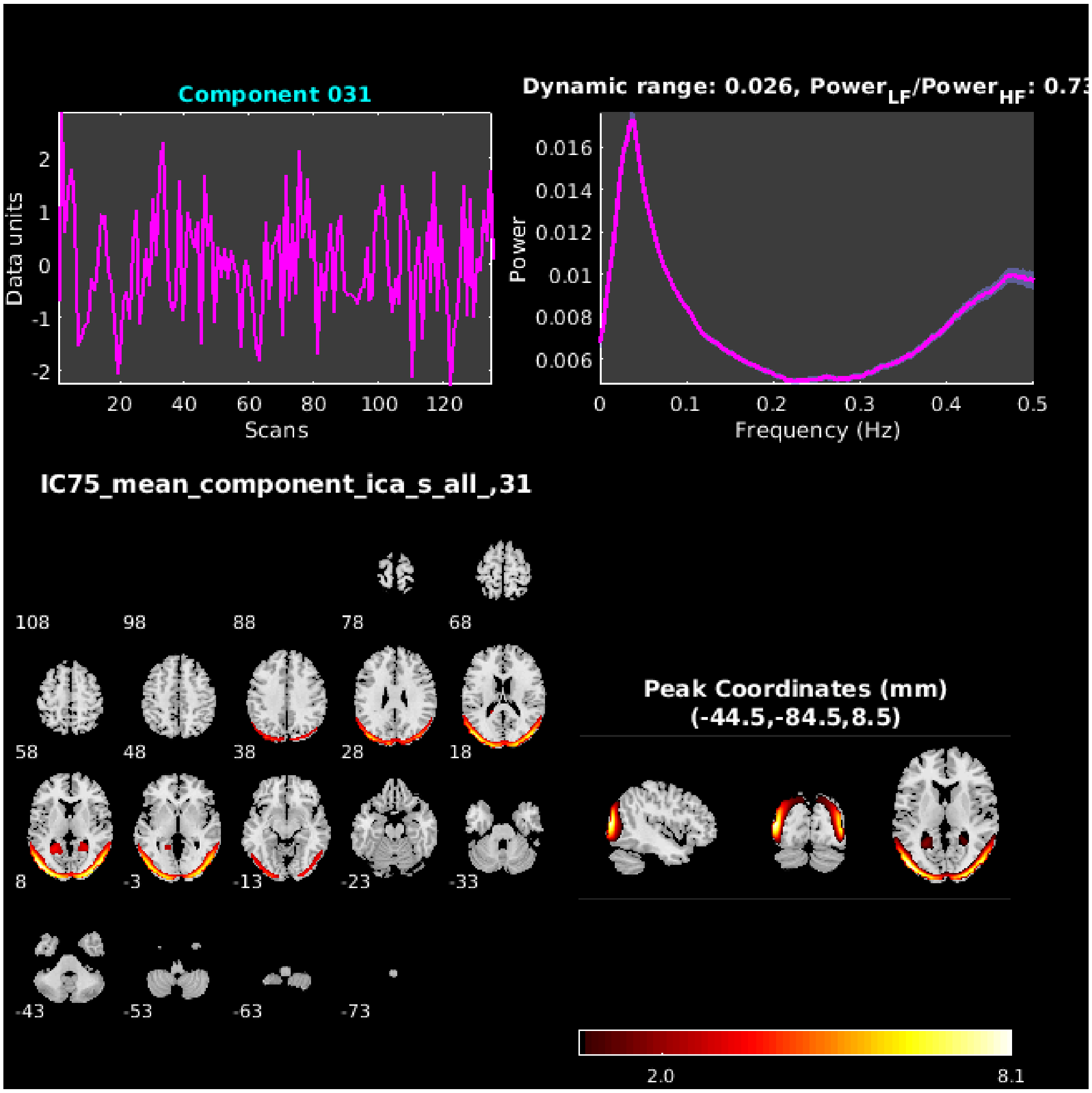
Component 031
Dynamic range: 0.026, Power_LF/Power_HF: 0.73
Data units
Scans
Power
Frequency (Hz)
IC75_mean_component_ica_s_all_,31
Peak Coordinates (mm)
(-44.5,-84.5,8.5)
2.0
8.1

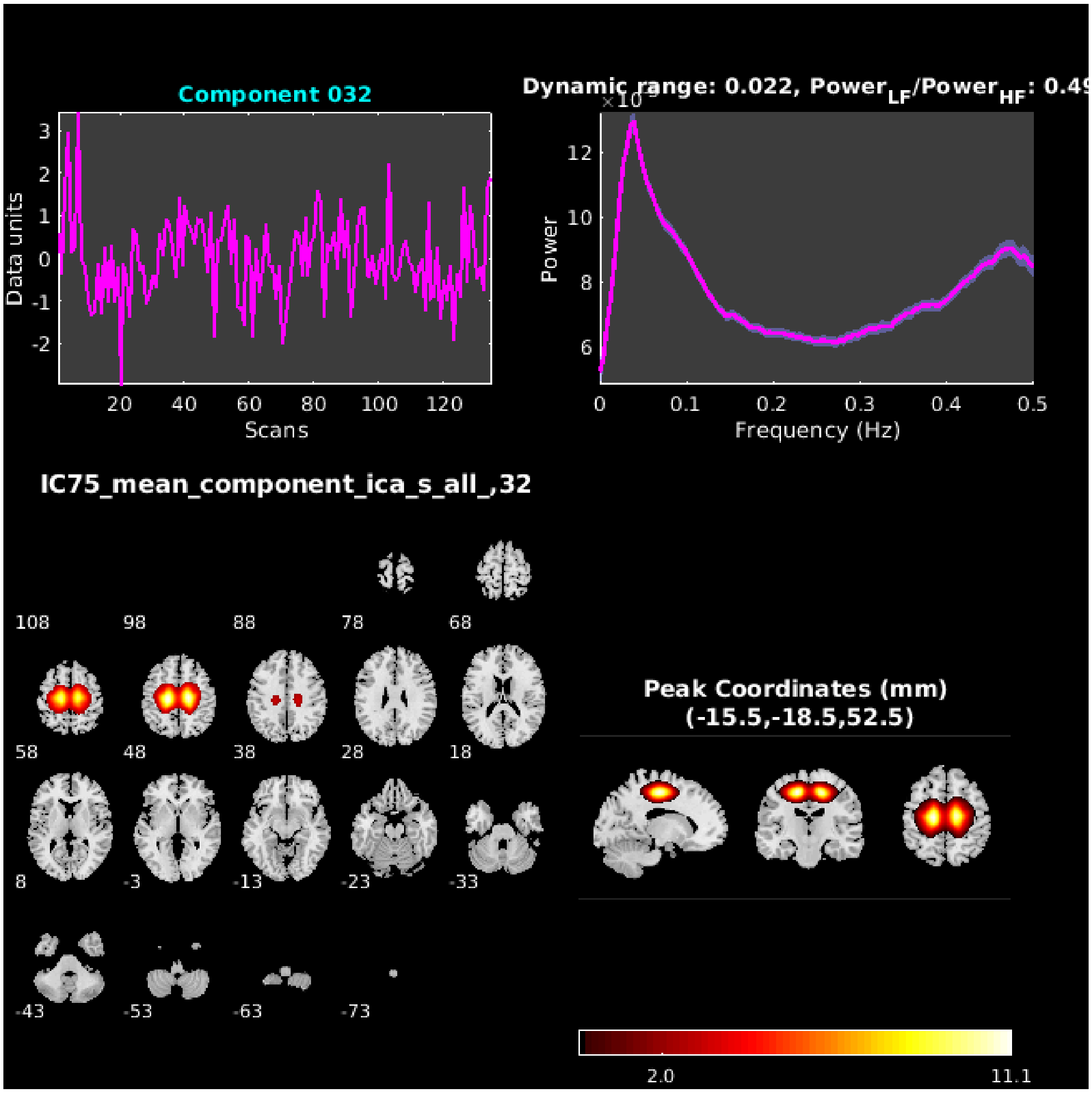
Component 032
Dynamic range: 0.022, Power$_{LF}$/Power$_{HF}$: 0.49
Data units
Scans
Power
Frequency (Hz)
IC75_mean_component_ica_s_all_,32
Peak Coordinates (mm)
(-15.5,-18.5,52.5)
2.0
11.1

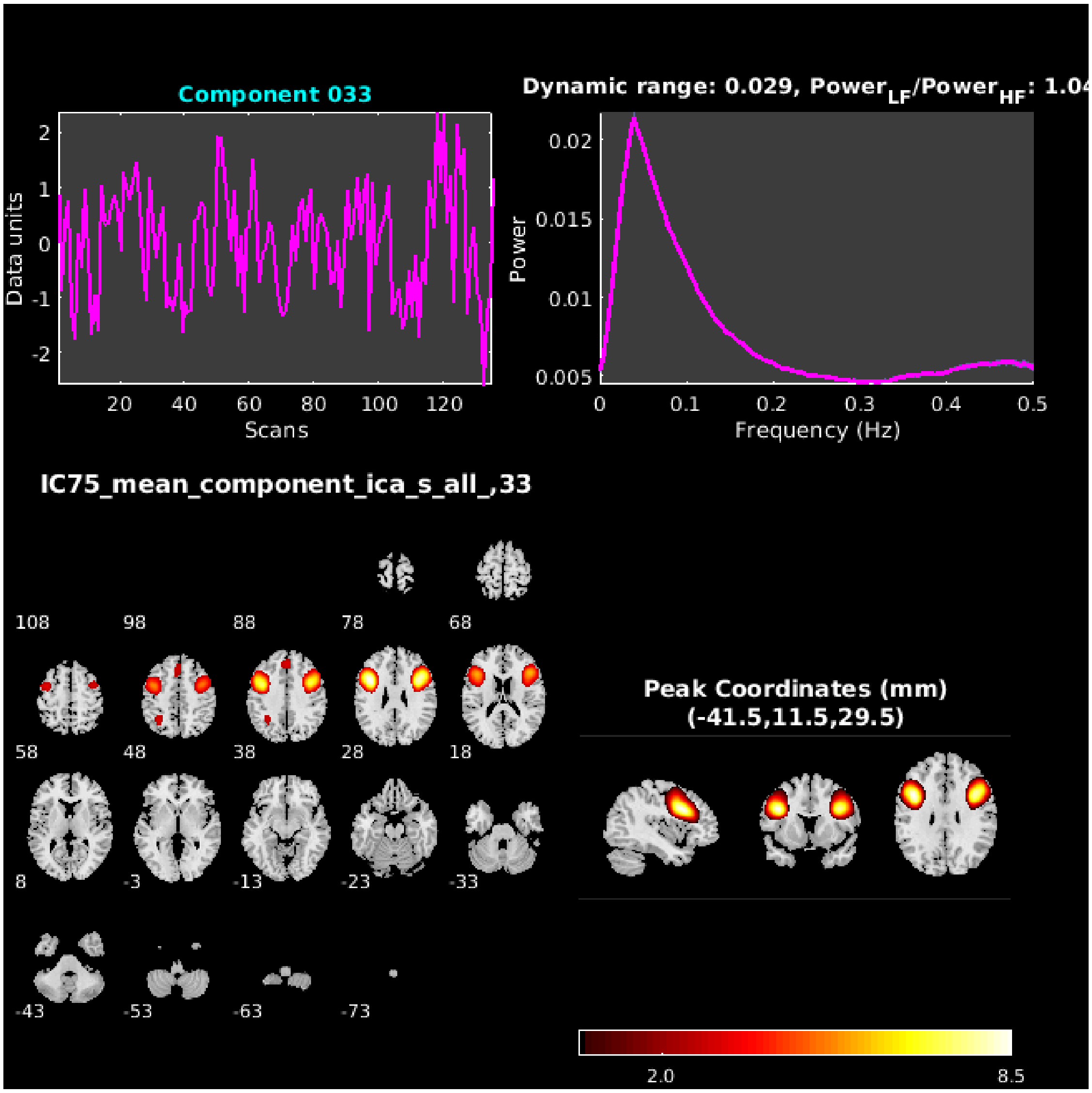
Component 033
Dynamic range: 0.029, Power_LF/Power_HF: 1.04
Data units
Scans
Power
Frequency (Hz)
IC75_mean_component_ica_s_all_,33
Peak Coordinates (mm)
(-41.5,11.5,29.5)
2.0
8.5

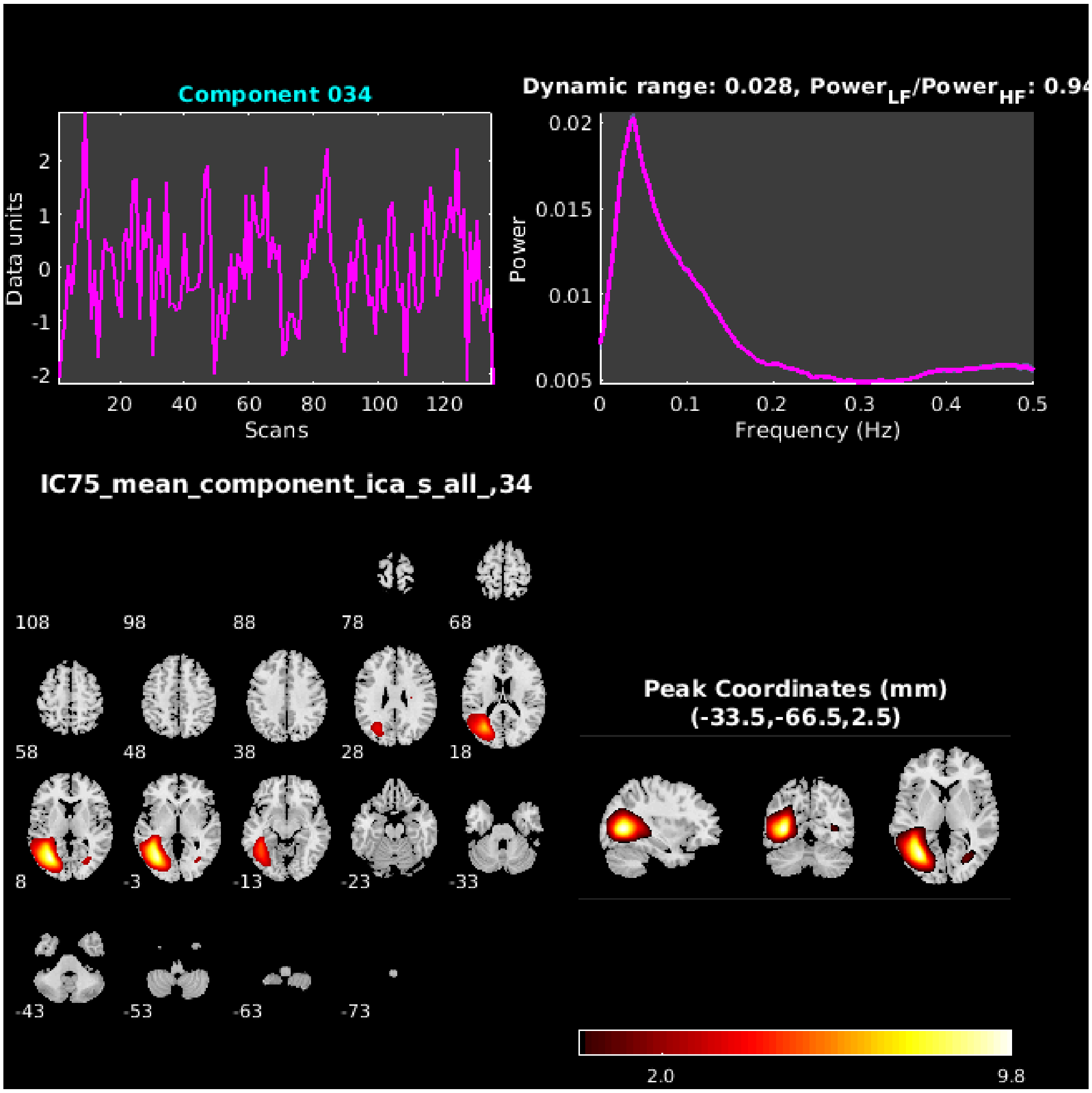
Component 034
Dynamic range: 0.028, Power$_{LF}$/Power$_{HF}$: 0.94
Data units
Scans
Power
Frequency (Hz)
IC75_mean_component_ica_s_all_,34
Peak Coordinates (mm)
(-33.5,-66.5,2.5)
2.0
9.8

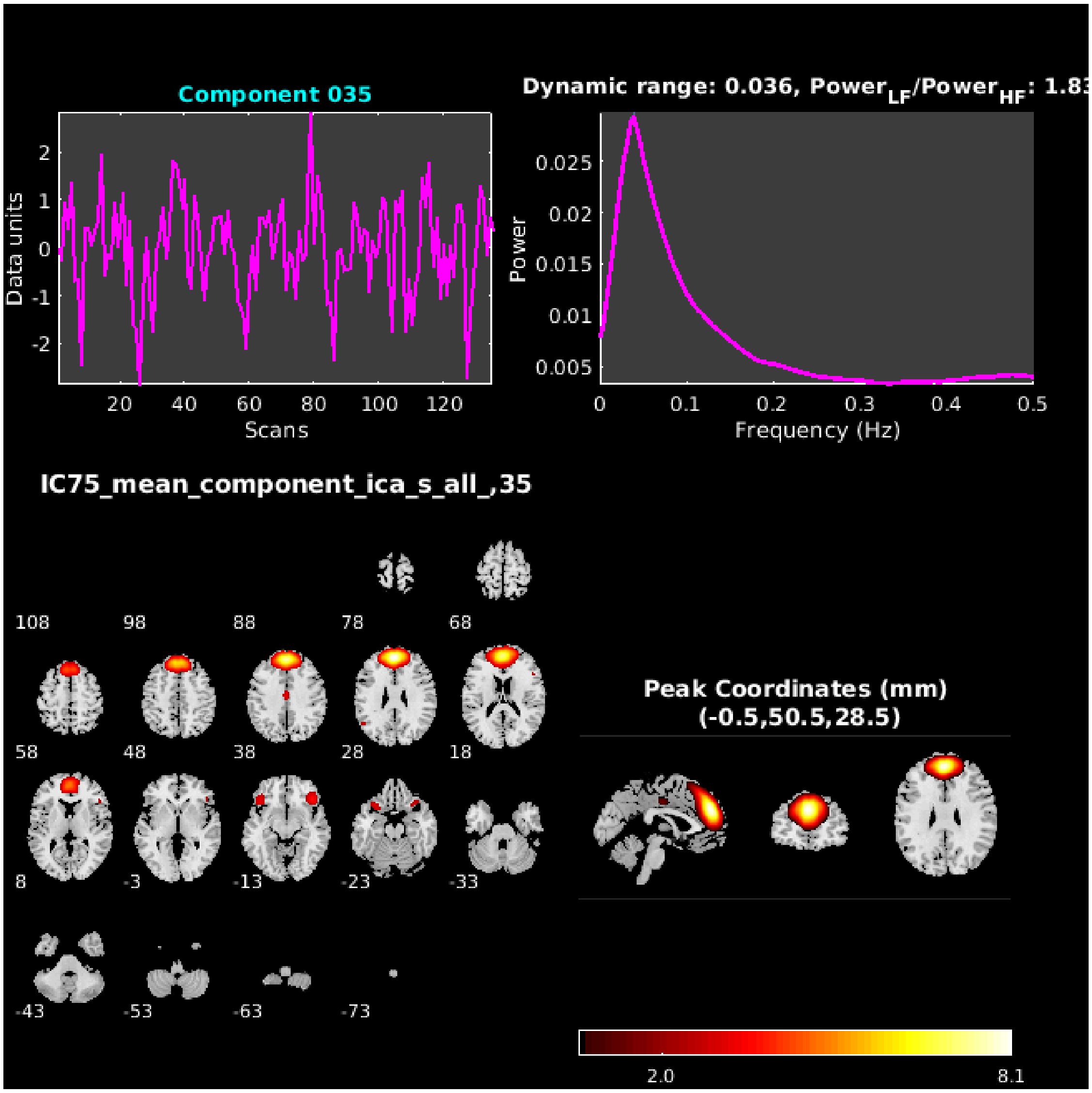
Component 035
Dynamic range: 0.036, Power_LF/Power_HF: 1.83
Data units
Scans
Power
Frequency (Hz)
IC75_mean_component_ica_s_all_,35
Peak Coordinates (mm)
(-0.5,50.5,28.5)
2.0
8.1

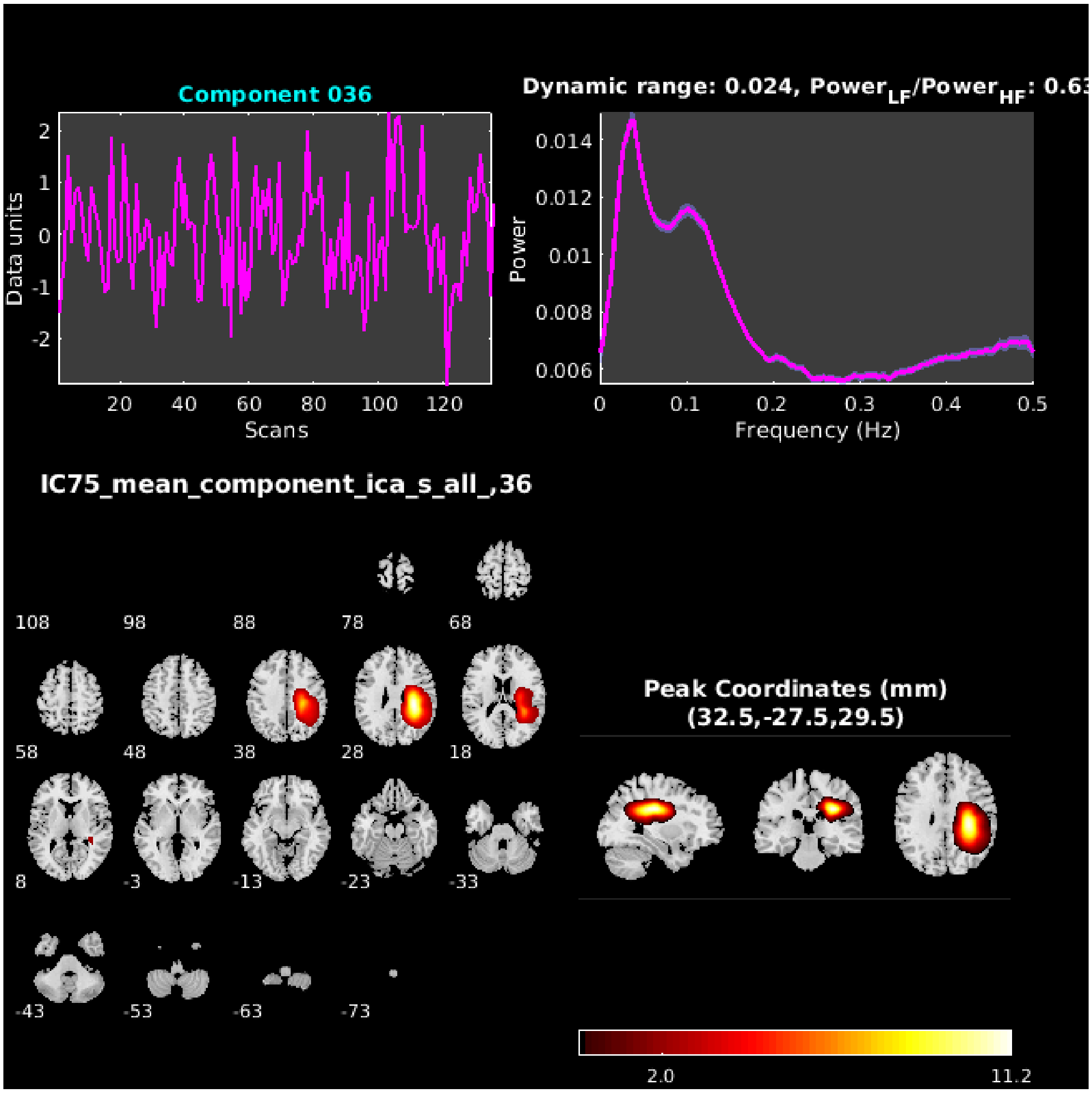

Component 036
Dynamic range: 0.024, Power$_{LF}$/Power$_{HF}$: 0.63
Data units
Scans
Power
Frequency (Hz)
IC75_mean_component_ica_s_all_,36
Peak Coordinates (mm)
(32.5,-27.5,29.5)
2.0
11.2

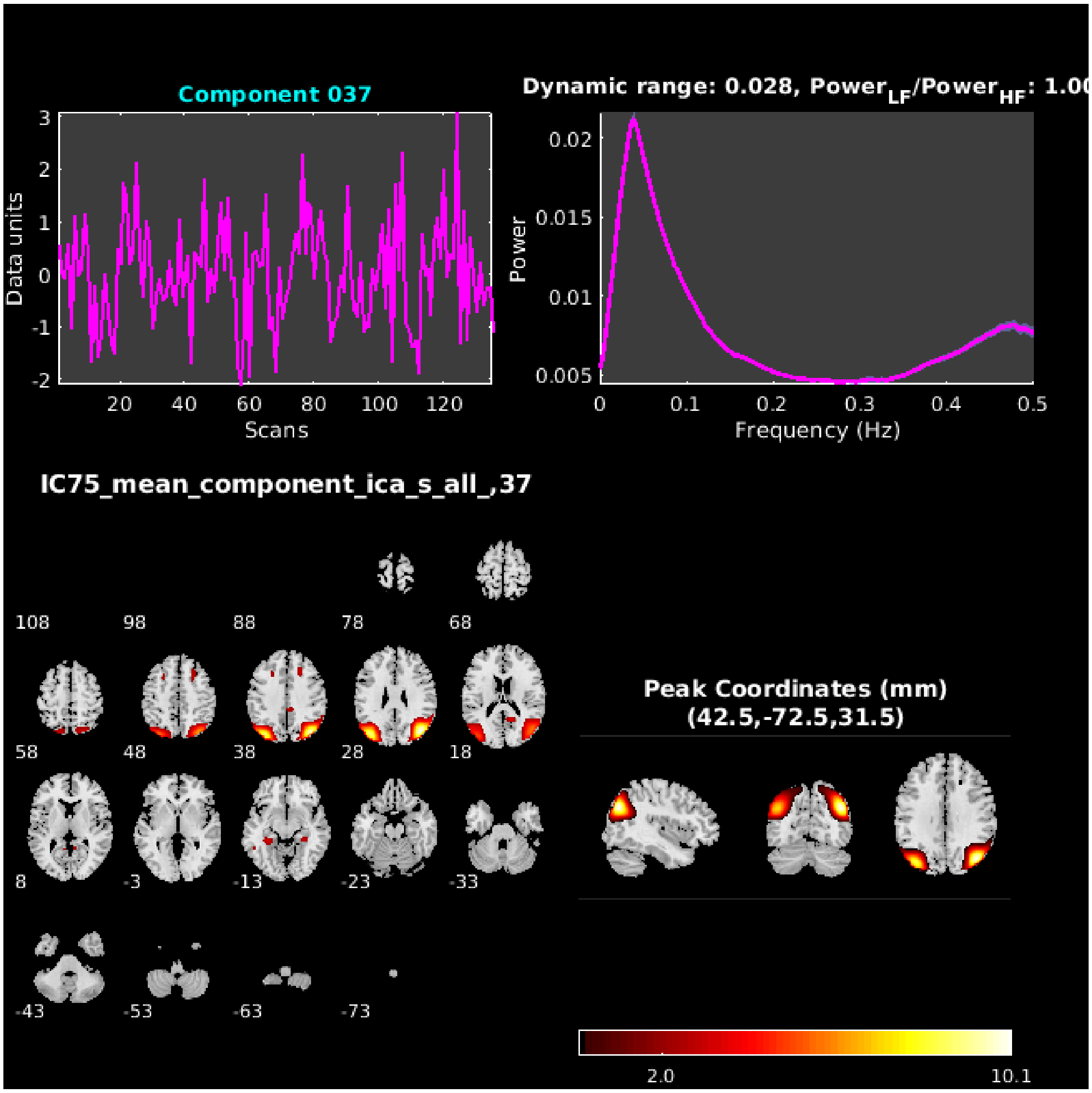

Component 037
Dynamic range: 0.028, Power$_{LF}$/Power$_{HF}$: 1.0
Data units
Scans
Power
Frequency (Hz)
IC75_mean_component_ica_s_all_,37
Peak Coordinates (mm)
(42.5,-72.5,31.5)
2.0
10.1

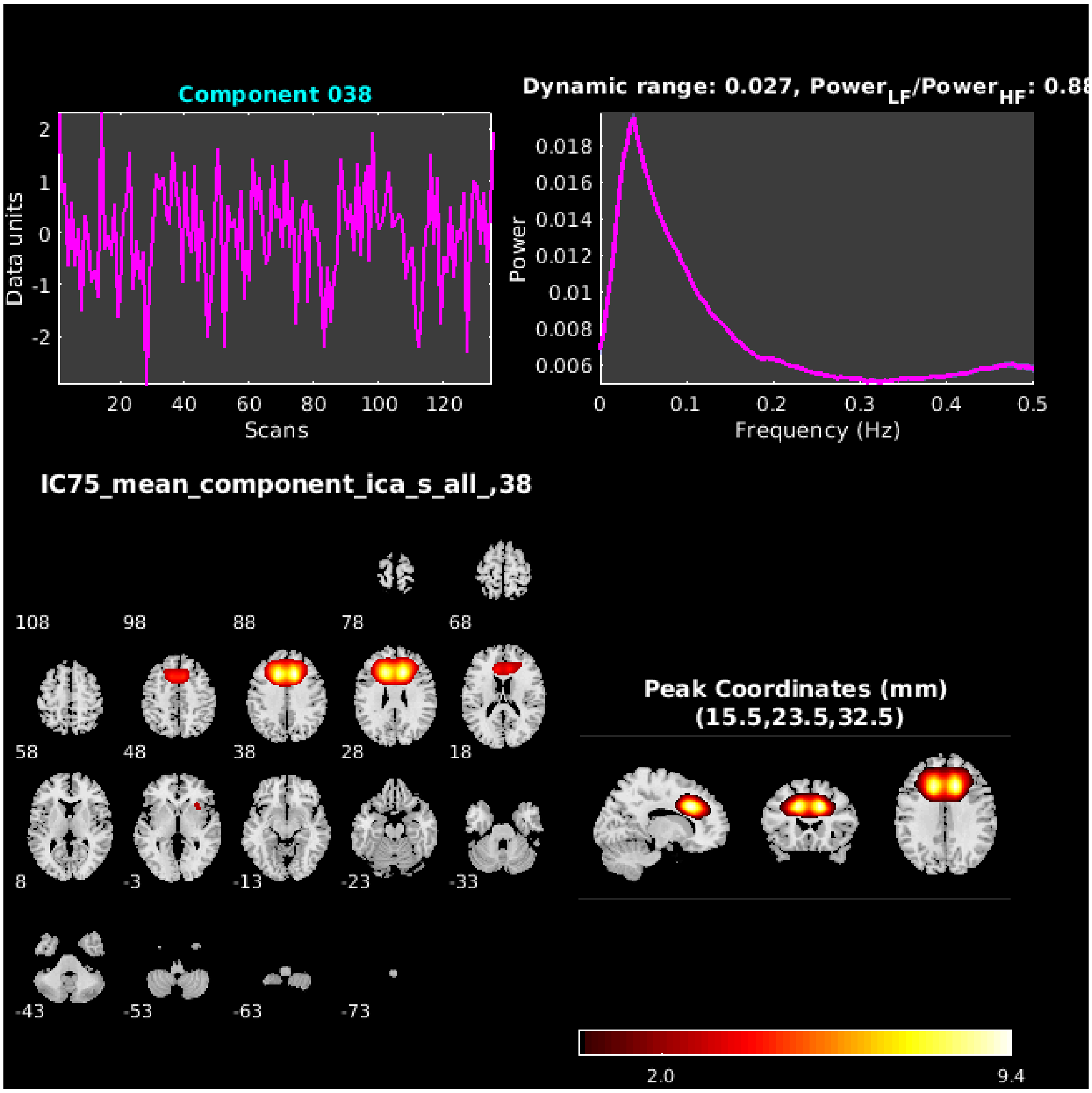
Component 038
Dynamic range: 0.027, Power$_{LF}$/Power$_{HF}$: 0.88
Data units
Scans
Power
Frequency (Hz)
IC75_mean_component_ica_s_all_,38
Peak Coordinates (mm)
(15.5,23.5,32.5)
2.0
9.4

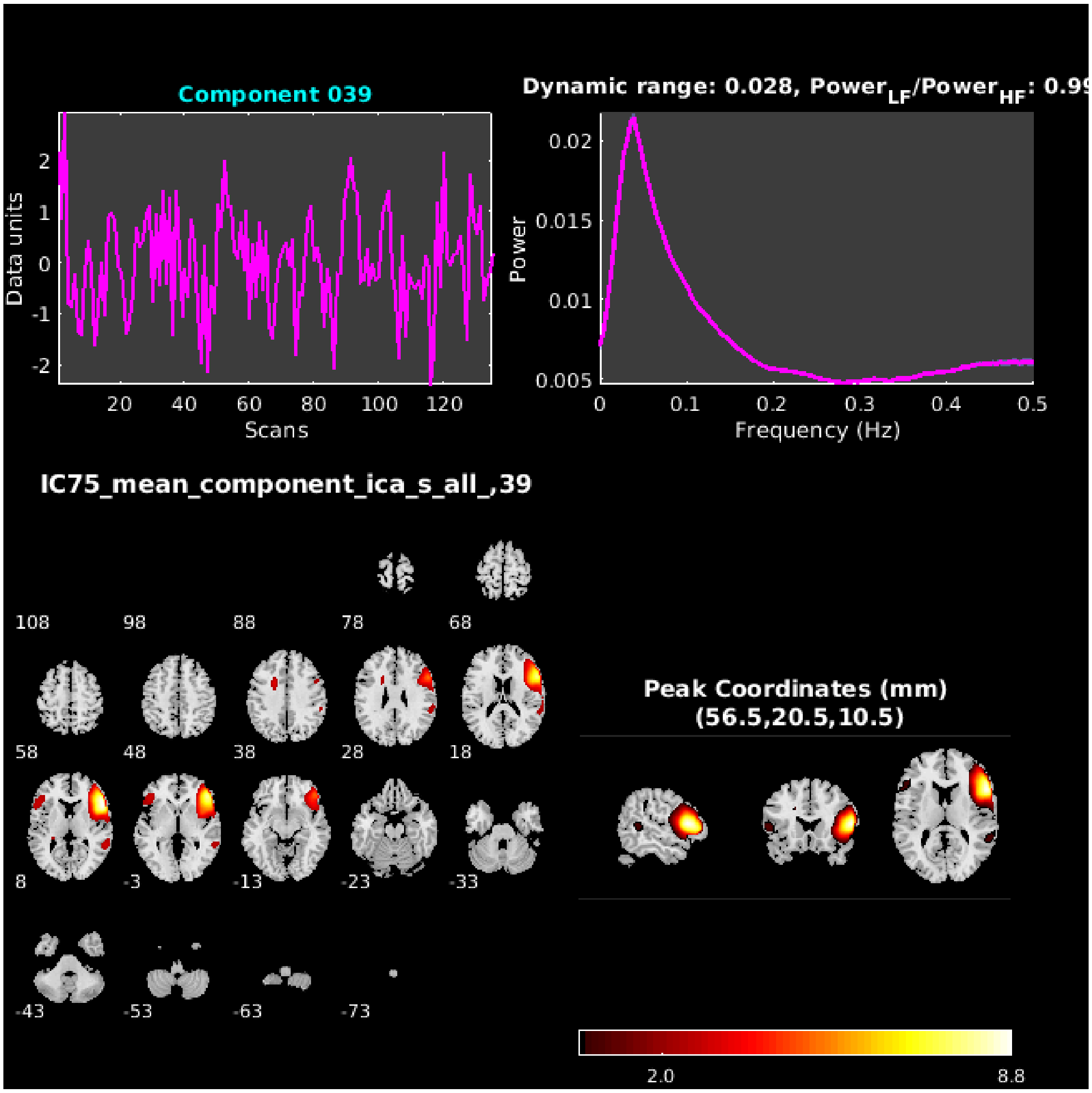

Component 039
Dynamic range: 0.028, Power$_{LF}$/Power$_{HF}$: 0.99
Data units
Scans
Power
Frequency (Hz)
IC75_mean_component_ica_s_all_,39
Peak Coordinates (mm)
(56.5,20.5,10.5)
2.0
8.8

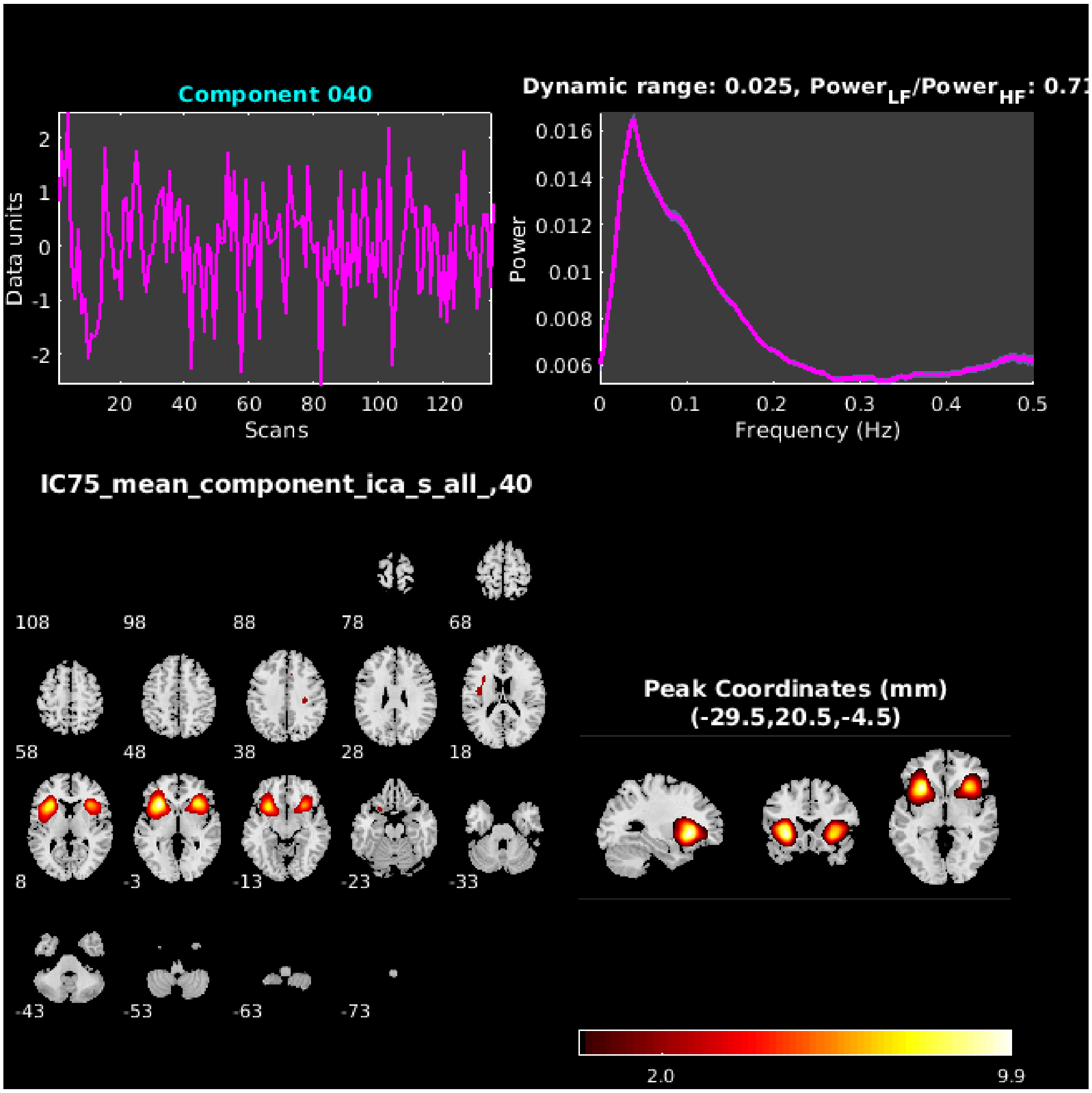

Component 040
Dynamic range: 0.025, Power$_{LF}$/Power$_{HF}$: 0.71
Data units
Scans
Power
Frequency (Hz)
IC75_mean_component_ica_s_all_,40
Peak Coordinates (mm)
(-29.5,20.5,-4.5)

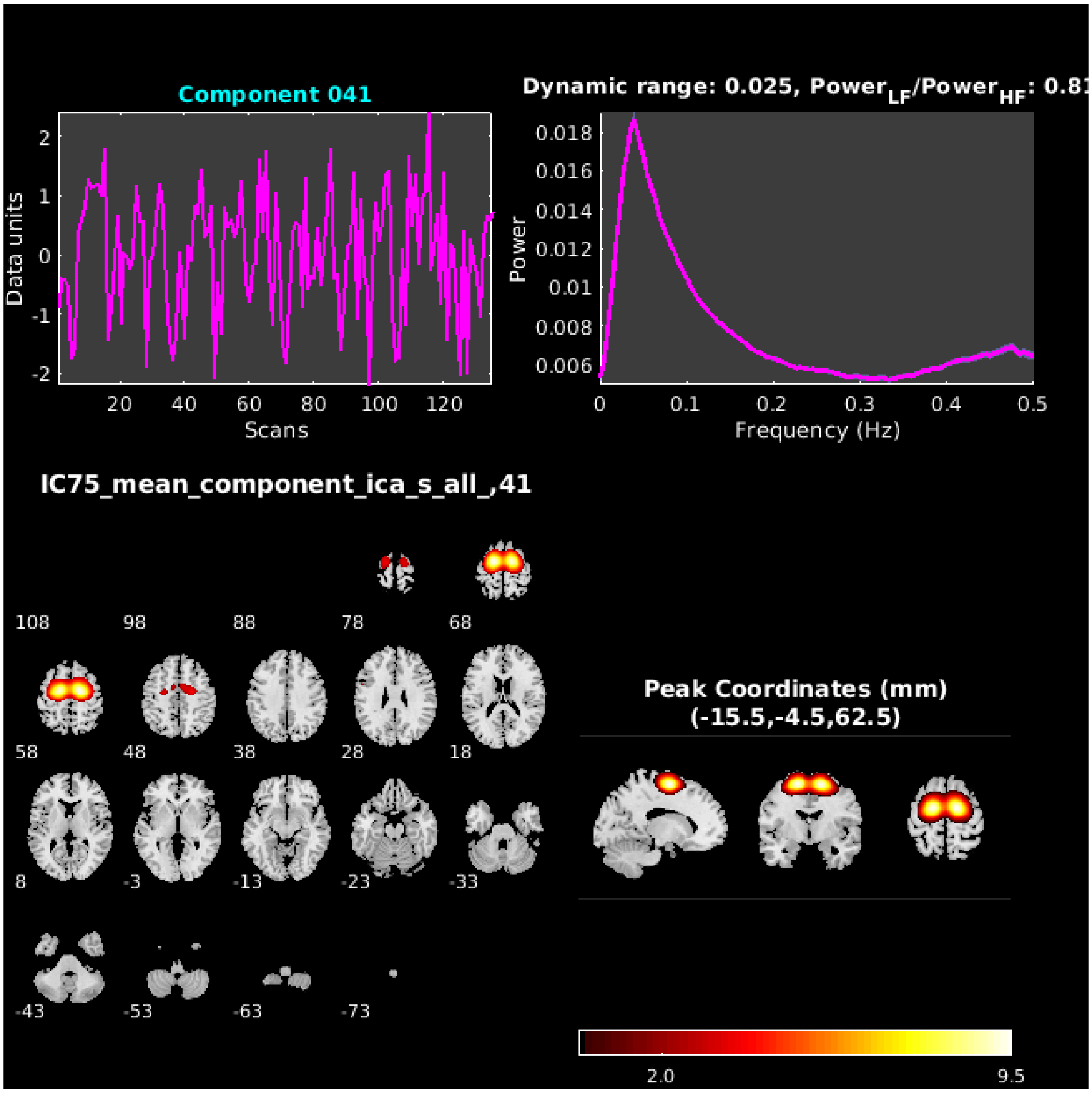
Component 041
Dynamic range: 0.025, Power_LF/Power_HF: 0.81
Data units
Scans
Power
Frequency (Hz)
IC75_mean_component_ica_s_all_,41
108
98
88
78
68
58
48
38
28
18
8
-3
-13
-23
-33
-43
-53
-63
-73
Peak Coordinates (mm)
(-15.5,-4.5,62.5)
2.0
9.5

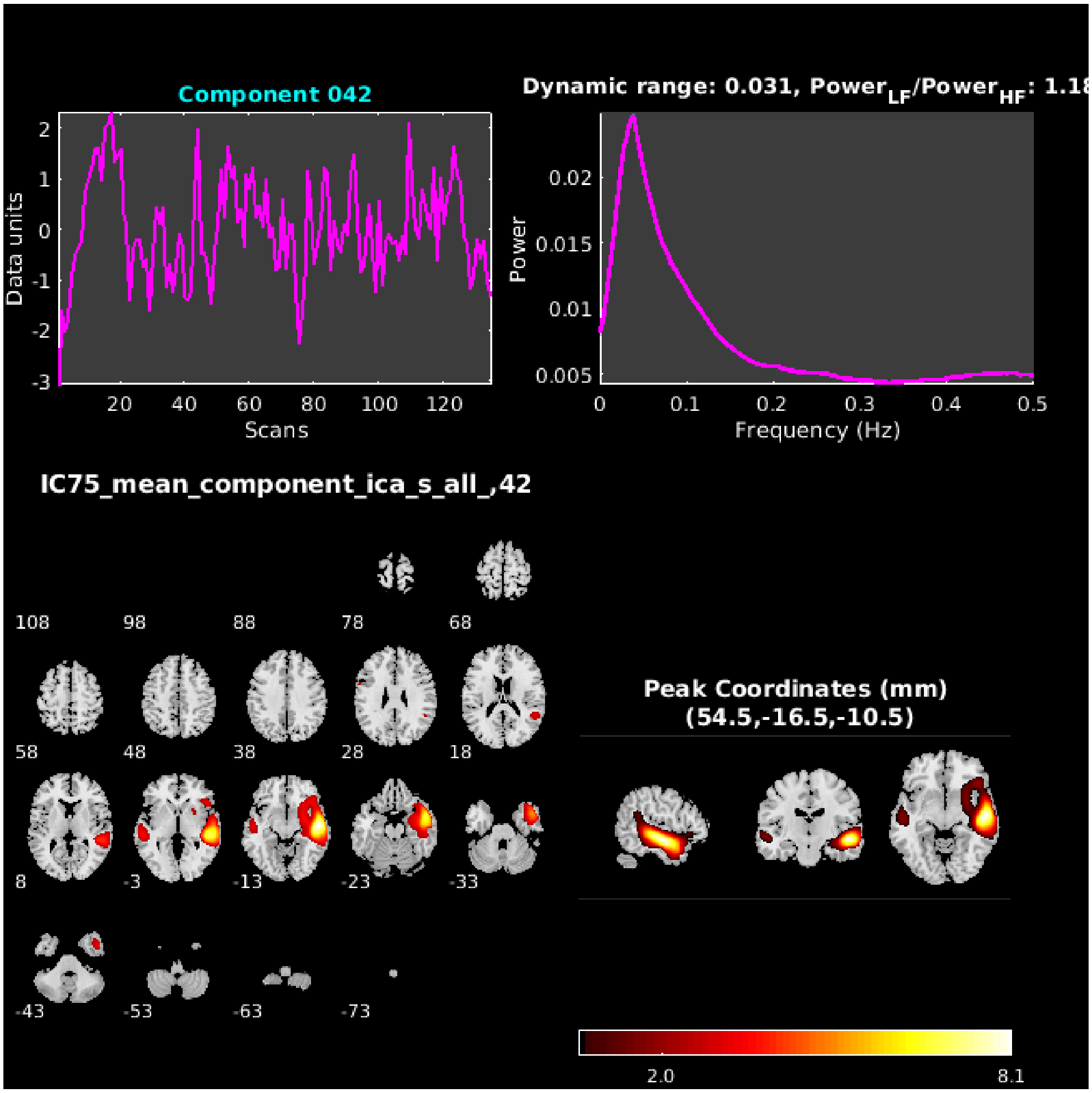
Component 042
Dynamic range: 0.031, Power$_{LF}$/Power$_{HF}$: 1.18
Data units
Scans
Power
Frequency (Hz)
IC75_mean_component_ica_s_all_,42
Peak Coordinates (mm)
(54.5,-16.5,-10.5)
2.0
8.1

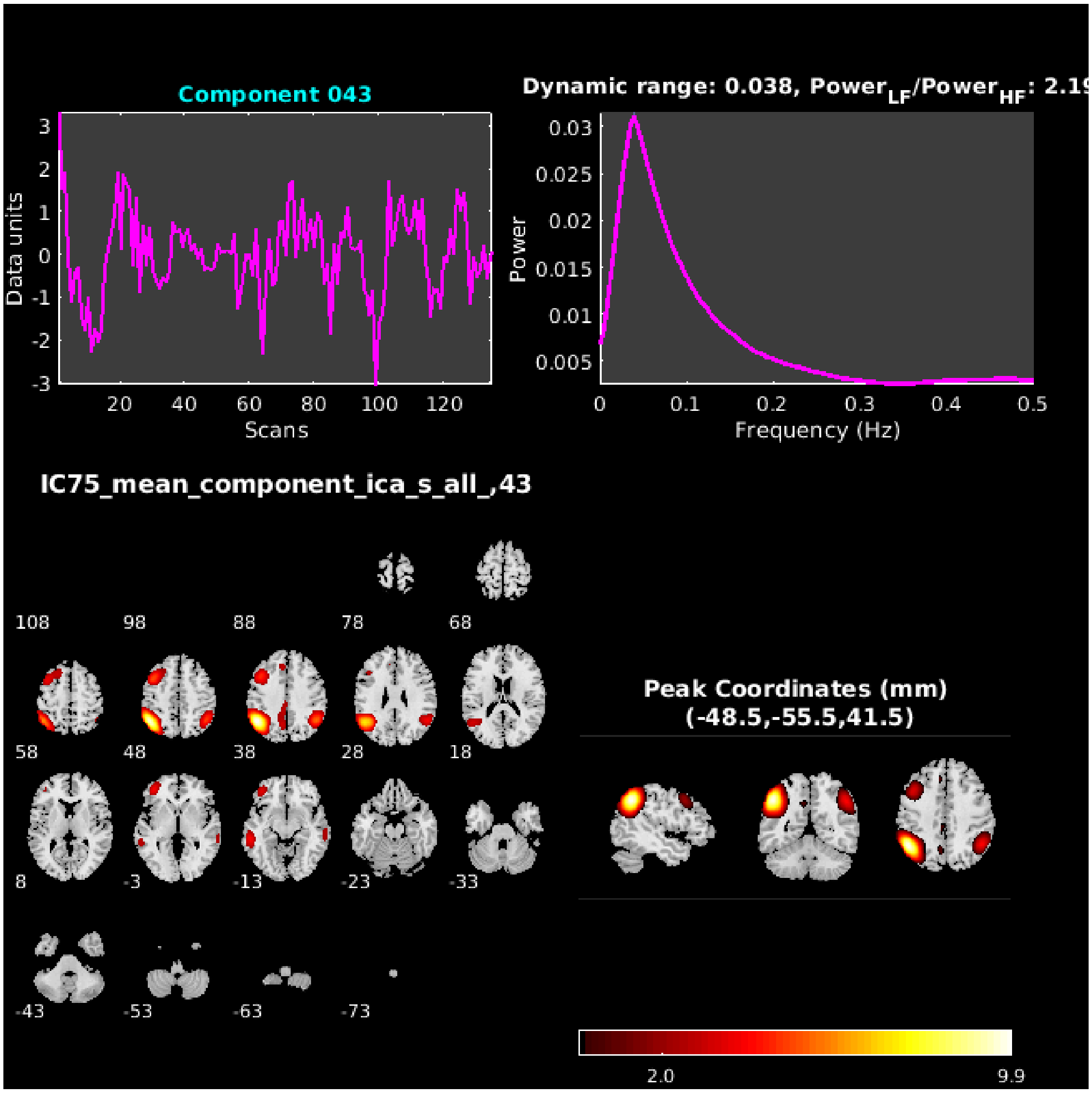

Component 043
Dynamic range: 0.038, Power$_{LF}$/Power$_{HF}$: 2.19
Data units
Scans
Power
Frequency (Hz)
IC75_mean_component_ica_s_all_,43
108
98
88
78
68
58
48
38
28
18
8
-3
-13
-23
-33
-43
-53
-63
-73
Peak Coordinates (mm)
(-48.5,-55.5,41.5)
2.0
9.9

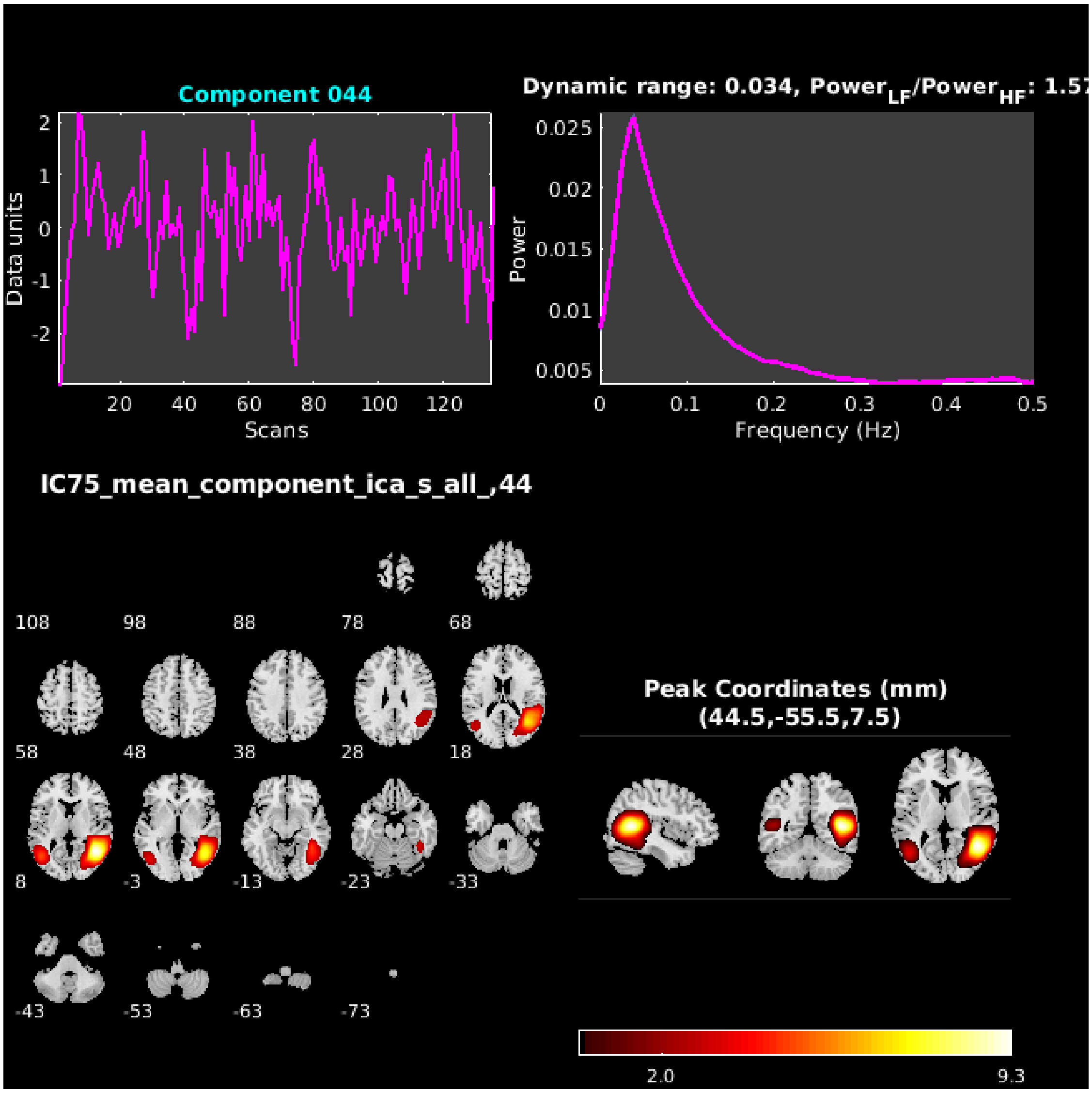

Component 044
Dynamic range: 0.034, Power_LF/Power_HF: 1.57
Data units
Scans
Power
Frequency (Hz)
IC75_mean_component_ica_s_all_,44
108
98
88
78
68
58
48
38
28
18
8
-3
-13
-23
-33
-43
-53
-63
-73
Peak Coordinates (mm)
(44.5,-55.5,7.5)
2.0
9.3

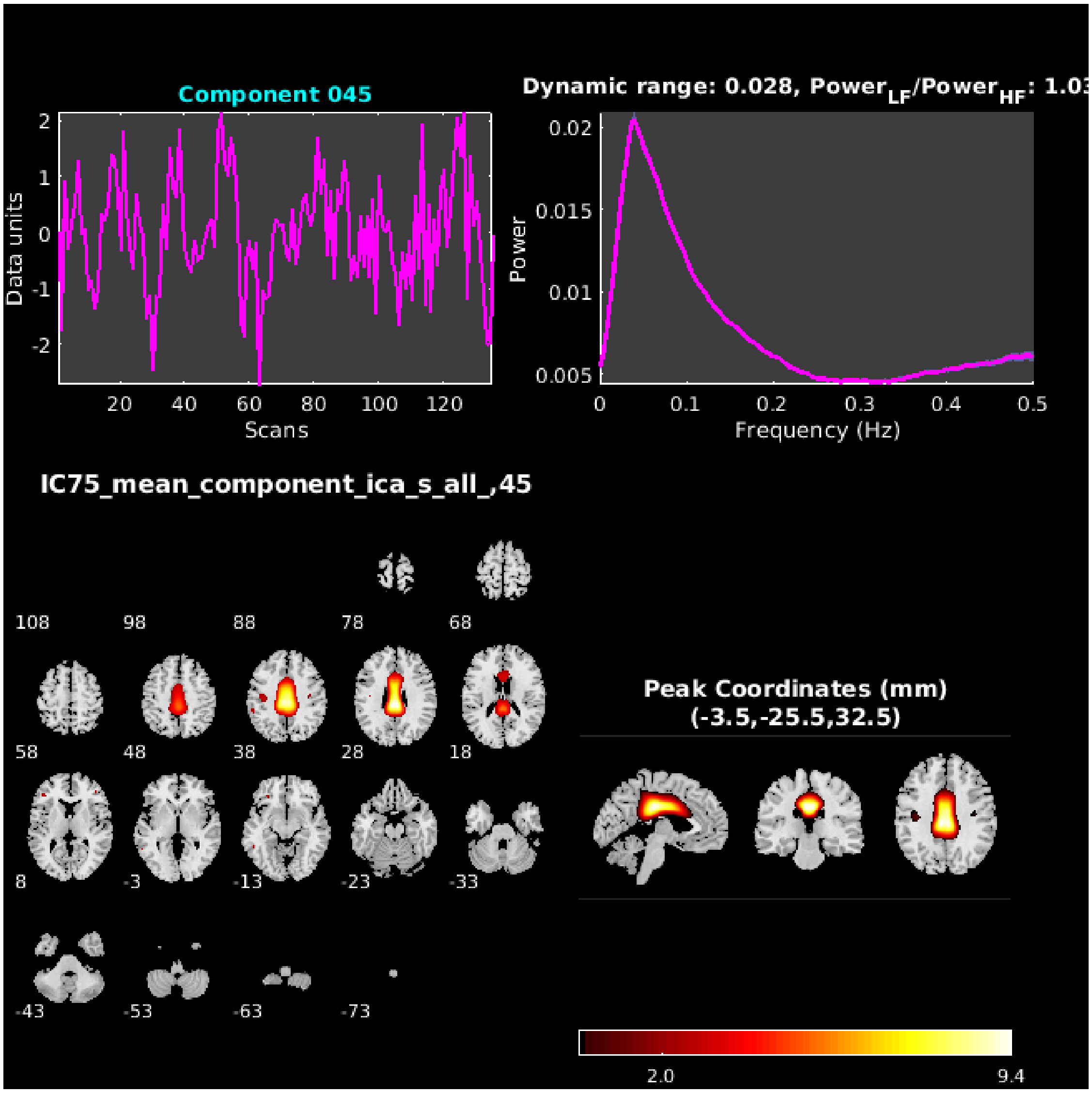

Component 045
Dynamic range: 0.028, Power$_{LF}$/Power$_{HF}$: 1.03
Data units
Scans
Power
Frequency (Hz)
IC75_mean_component_ica_s_all_,45
108
98
88
78
68
58
48
38
28
18
8
-3
-13
-23
-33
-43
-53
-63
-73
Peak Coordinates (mm)
(-3.5,-25.5,32.5)
2.0
9.4

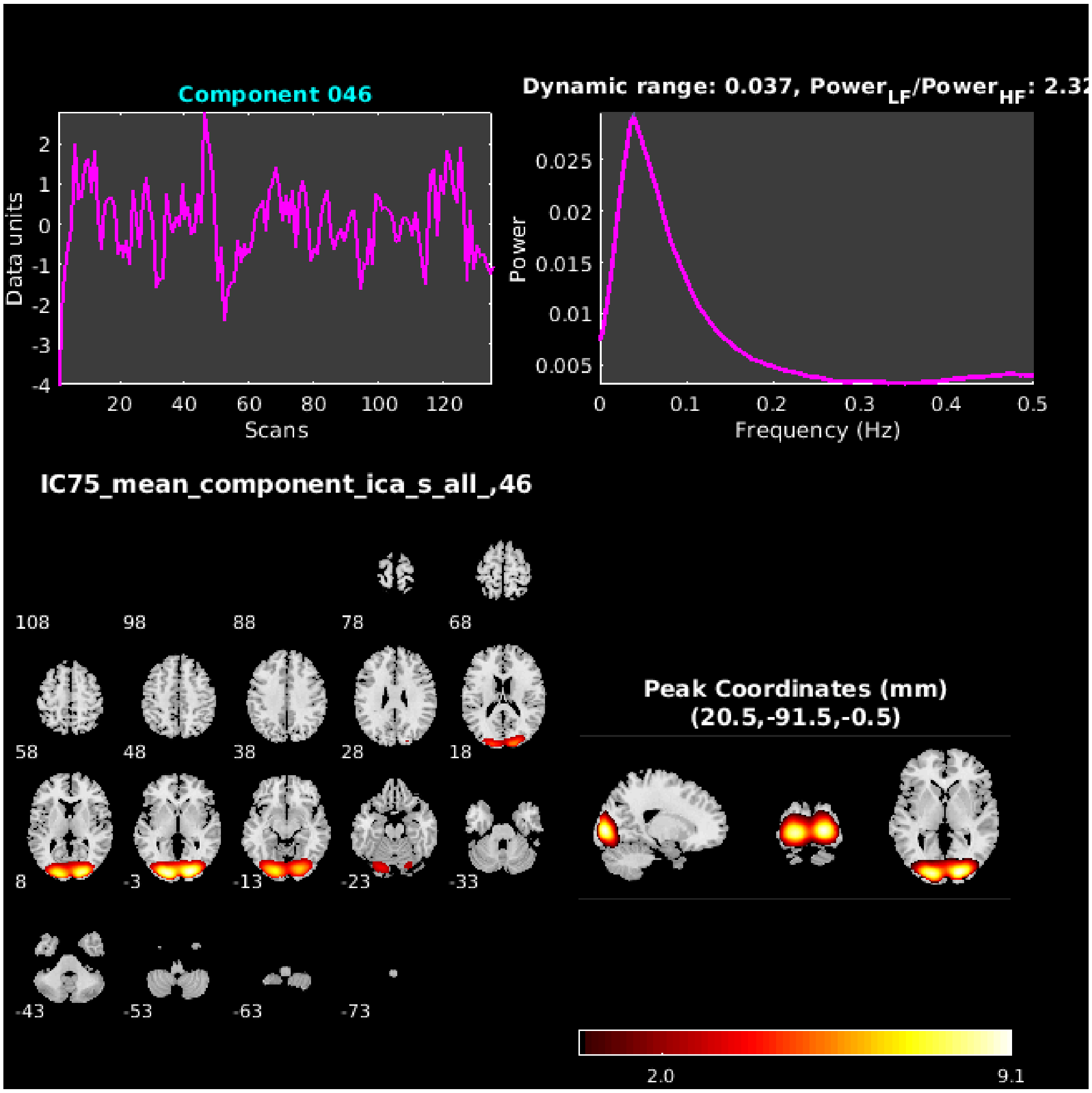

Component 046
Dynamic range: 0.037, Power$_{LF}$/Power$_{HF}$: 2.32
Data units
Scans
Power
Frequency (Hz)
IC75_mean_component_ica_s_all_,46
Peak Coordinates (mm)
(20.5,-91.5,-0.5)
2.0
9.1

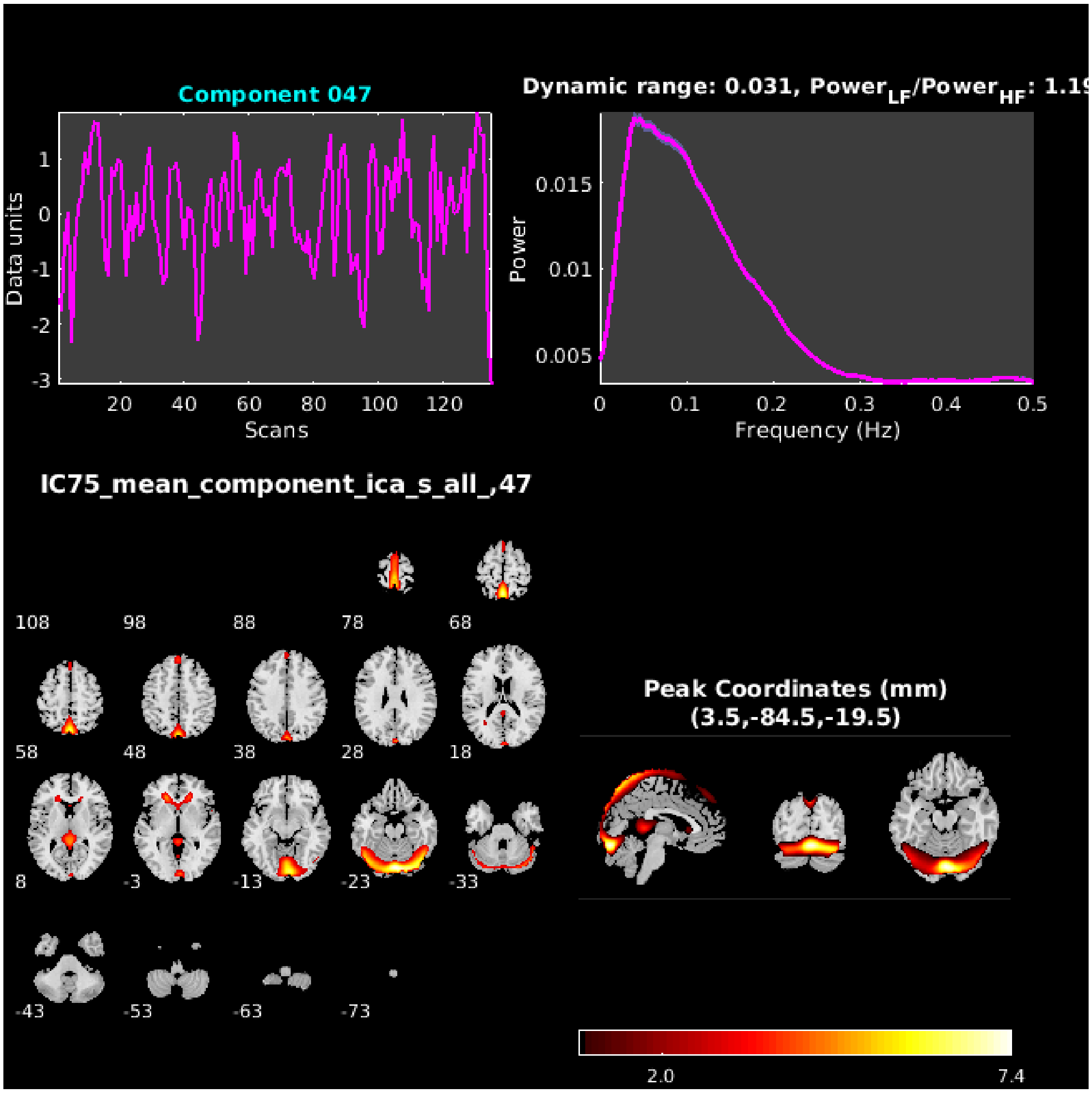

Component 047
Dynamic range: 0.031, Power$_{LF}$/Power$_{HF}$: 1.19
Data units
Scans
Power
Frequency (Hz)
IC75_mean_component_ica_s_all_,47
Peak Coordinates (mm)
(3.5,-84.5,-19.5)
2.0
7.4

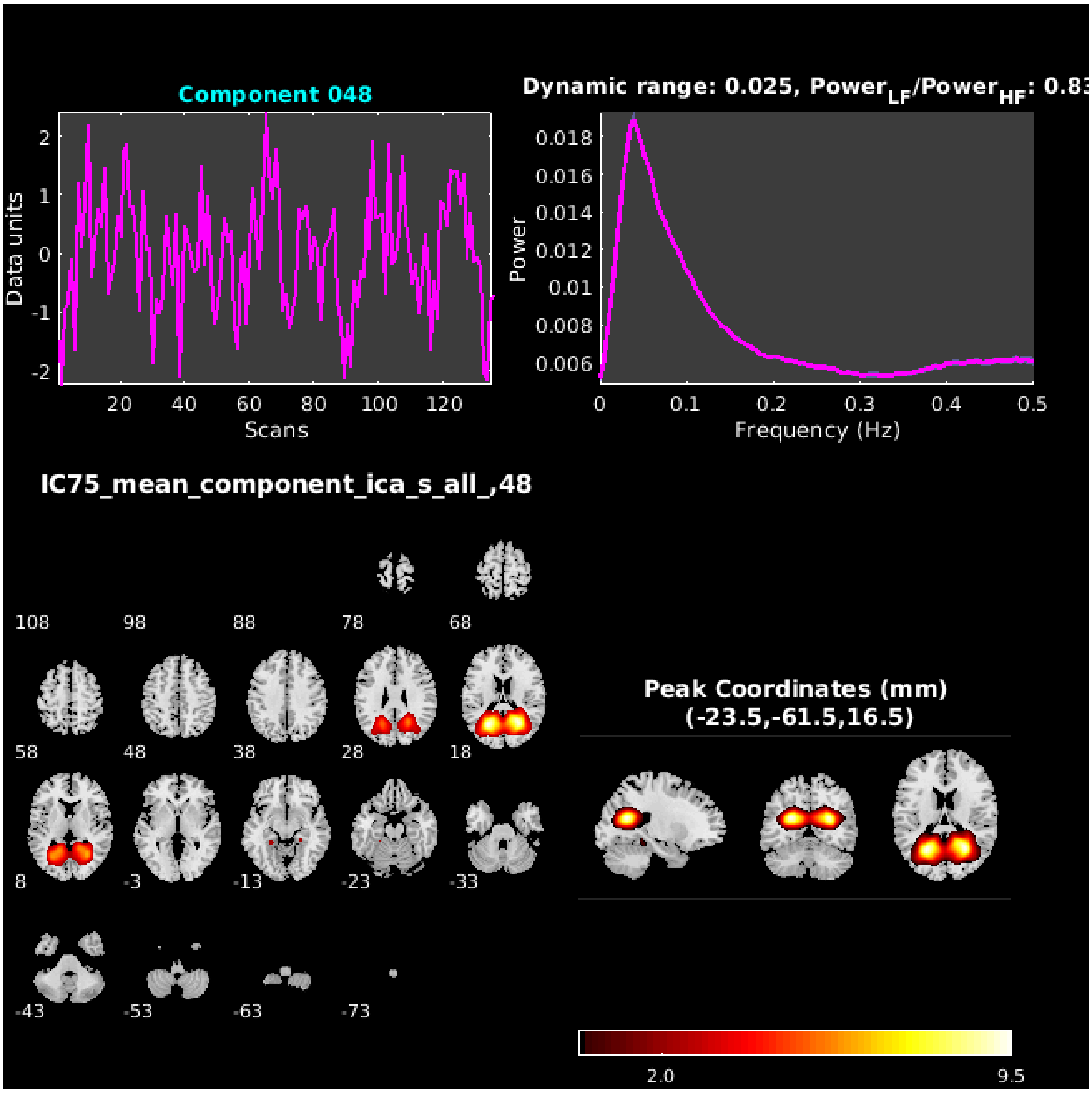
Component 048
Dynamic range: 0.025, Power$_{LF}$/Power$_{HF}$: 0.83
Data units
Scans
Power
Frequency (Hz)
IC75_mean_component_ica_s_all_,48
108
98
88
78
68
58
48
38
28
18
8
-3
-13
-23
-33
-43
-53
-63
-73
Peak Coordinates (mm)
(-23.5,-61.5,16.5)
2.0
9.5

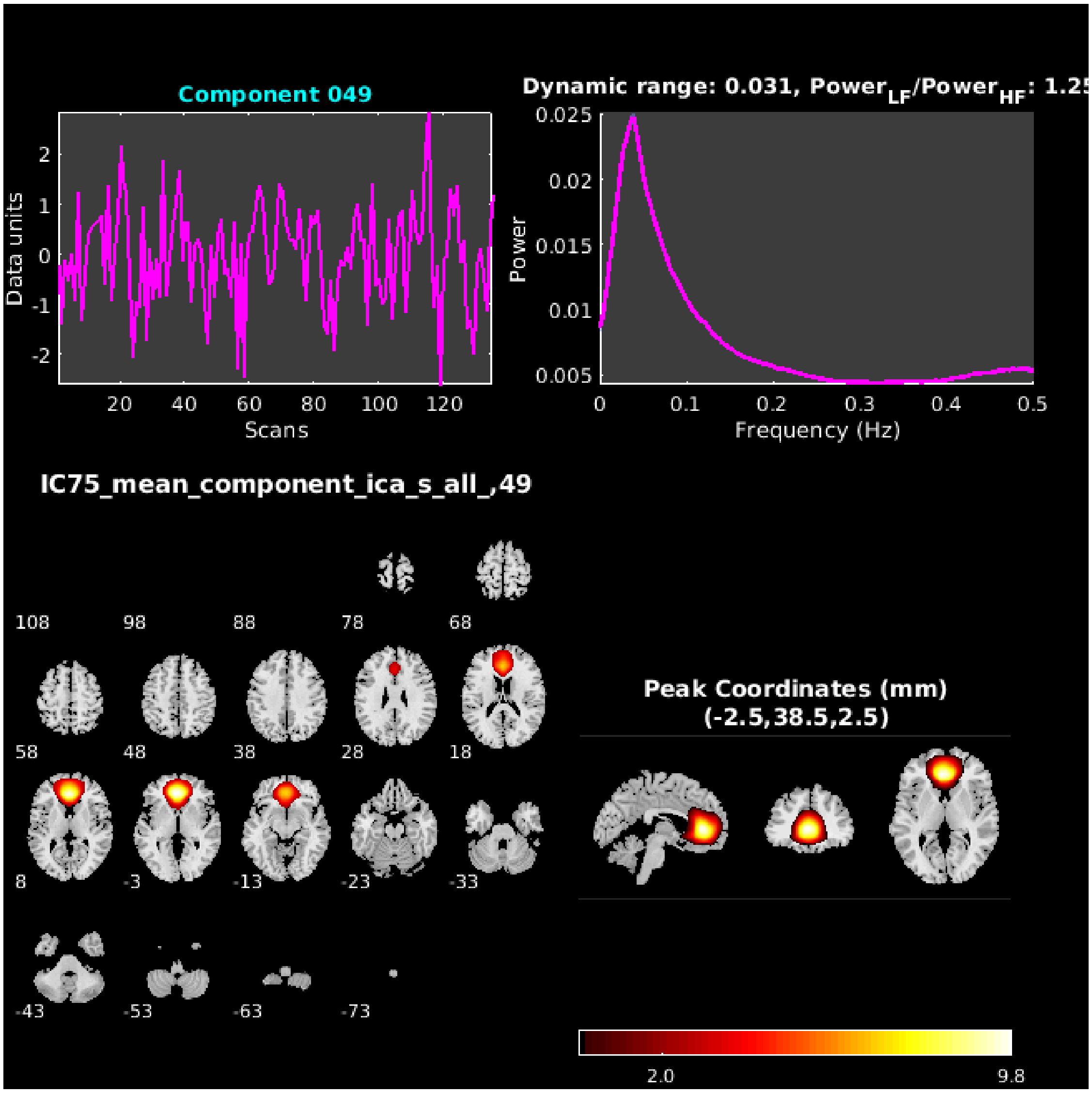

Component 049
Dynamic range: 0.031, Power$_{LF}$/Power$_{HF}$: 1.25
Data units
Scans
Power
Frequency (Hz)
IC75_mean_component_ica_s_all_,49
108
98
88
78
68
58
48
38
28
18
8
-3
-13
-23
-33
-43
-53
-63
-73
Peak Coordinates (mm)
(-2.5,38.5,2.5)
2.0
9.8

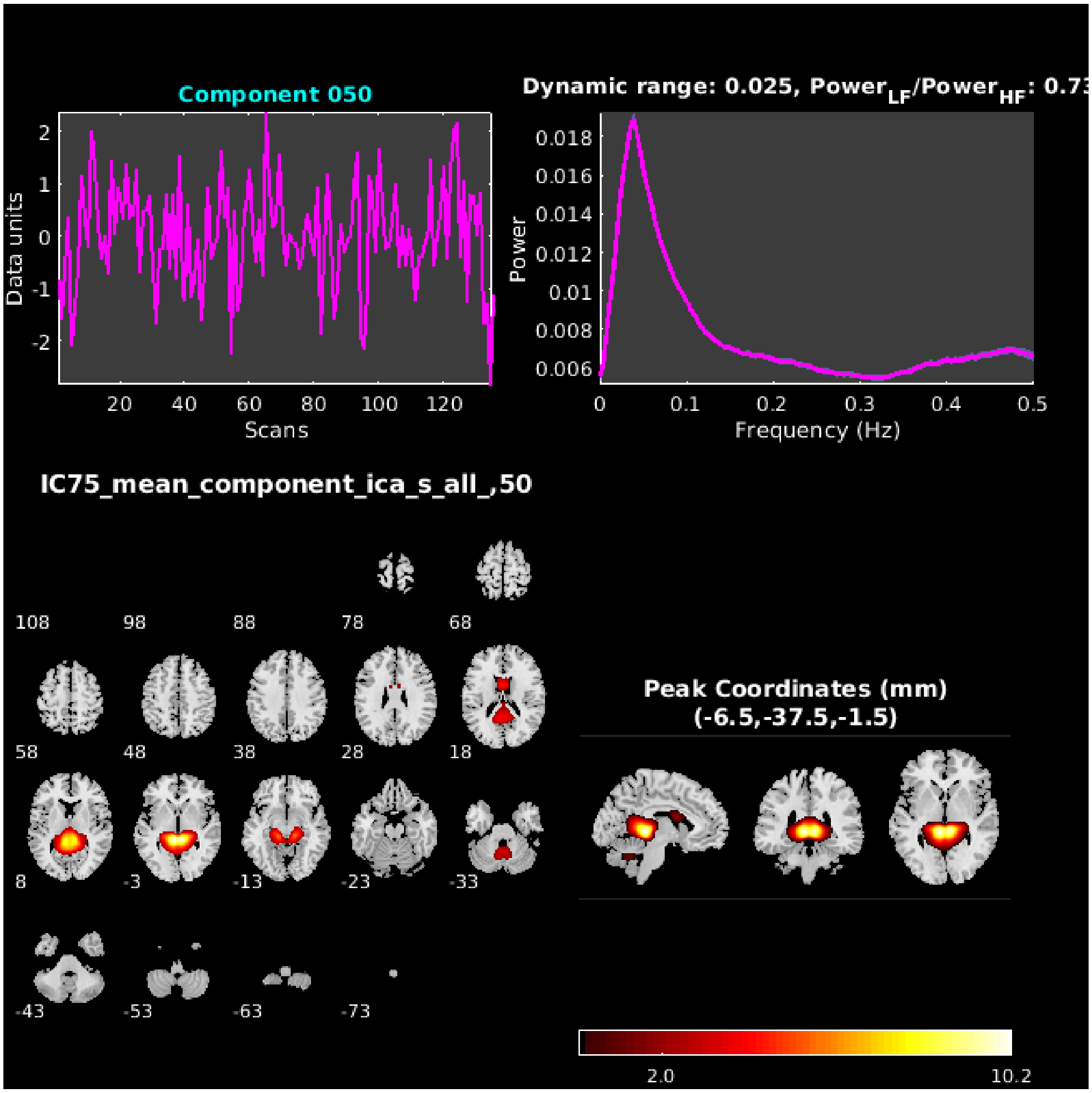
Component 050
Dynamic range: 0.025, Power_LF/Power_HF: 0.73
Data units
Scans
Power
Frequency (Hz)
IC75_mean_component_ica_s_all_,50
Peak Coordinates (mm)
(-6.5,-37.5,-1.5)
2.0
10.2

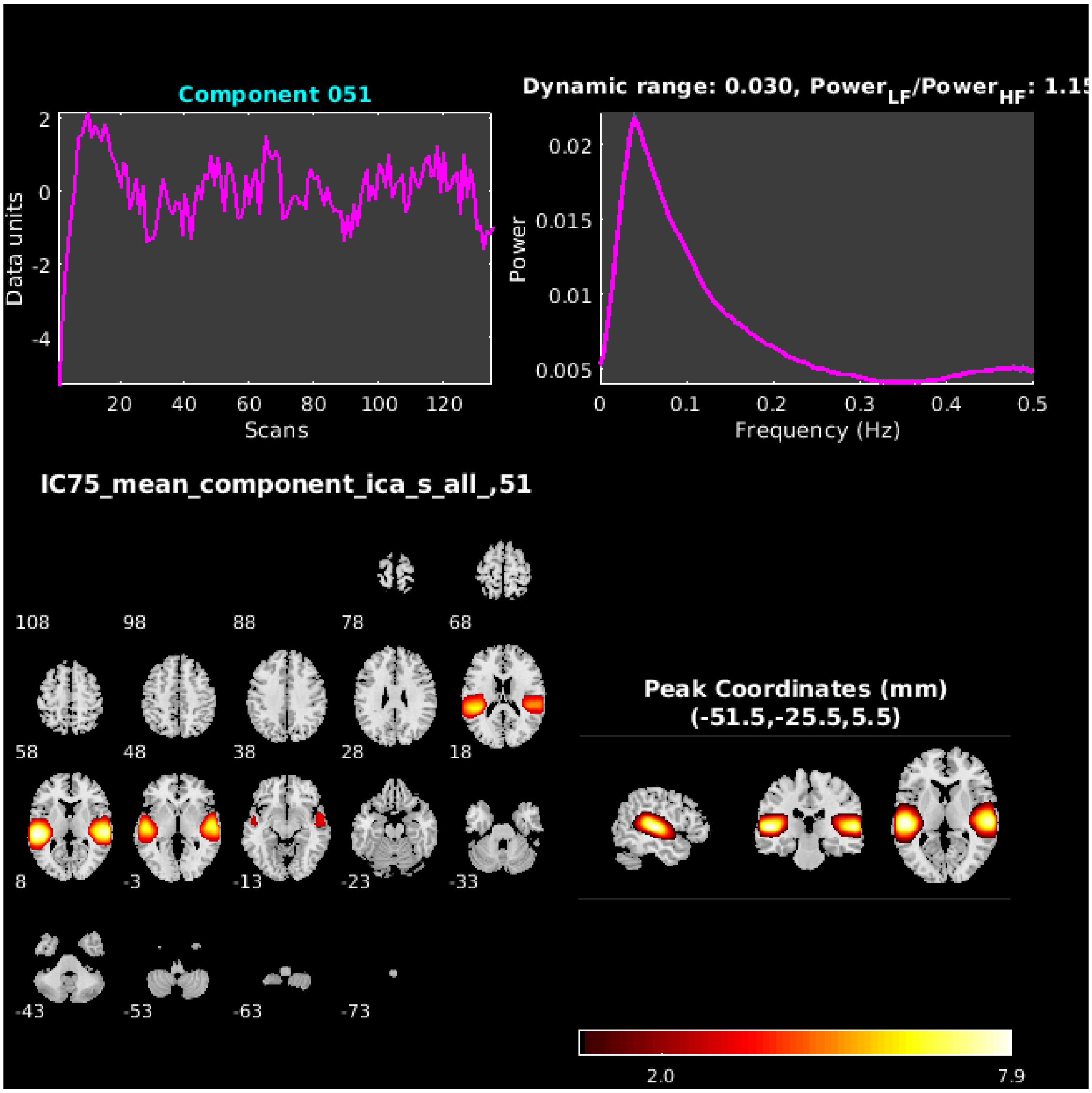

Component 051
Dynamic range: 0.030, Power$_{LF}$/Power$_{HF}$: 1.15
Data units
Scans
Power
Frequency (Hz)
IC75_mean_component_ica_s_all_,51
Peak Coordinates (mm)
(-51.5,-25.5,5.5)
2.0
7.9

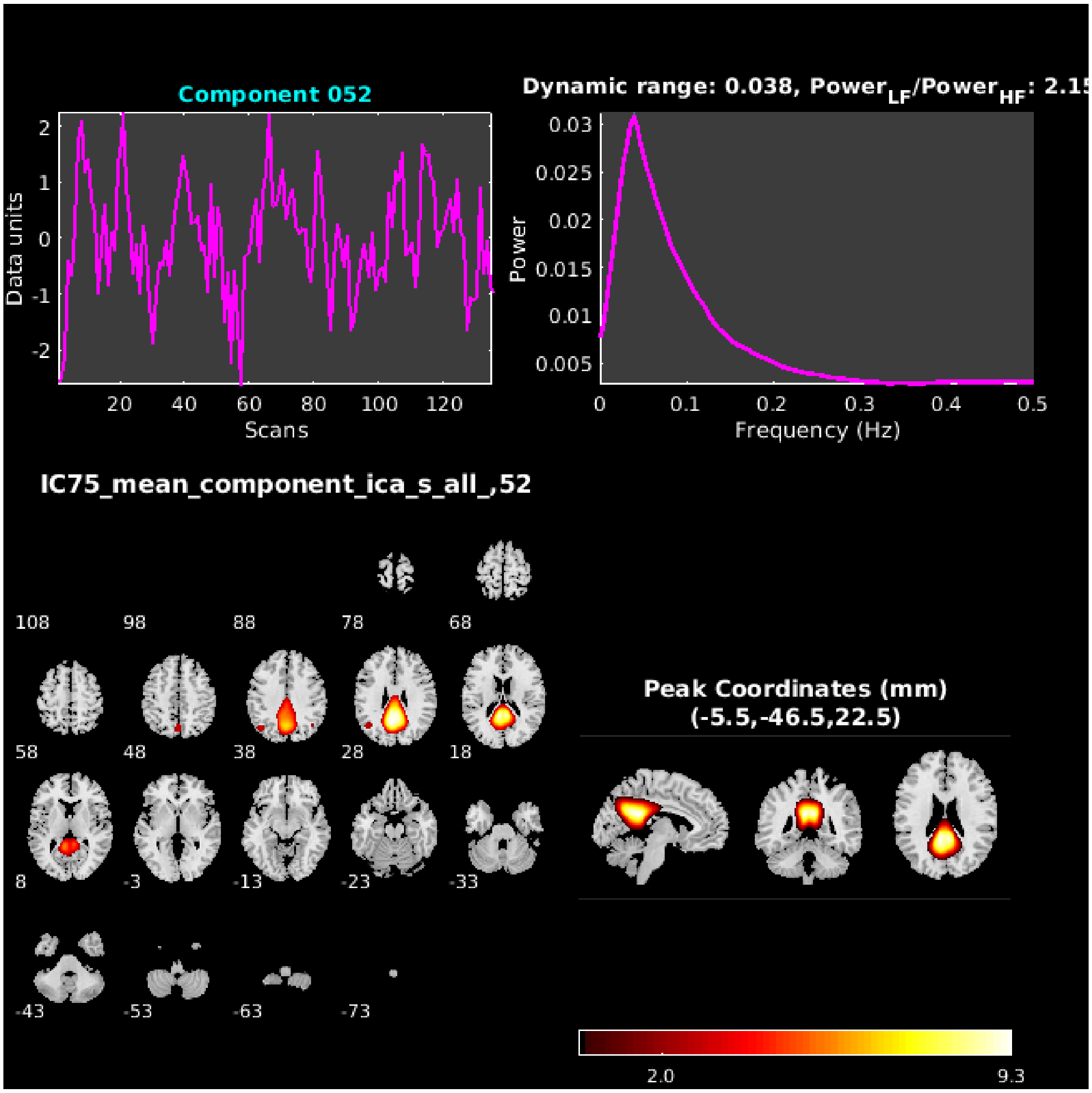
Component 052
Dynamic range: 0.038, Power_LF/Power_HF: 2.15
Data units
Scans
Power
Frequency (Hz)
IC75_mean_component_ica_s_all_,52
Peak Coordinates (mm)
(-5.5,-46.5,22.5)
2.0
9.3

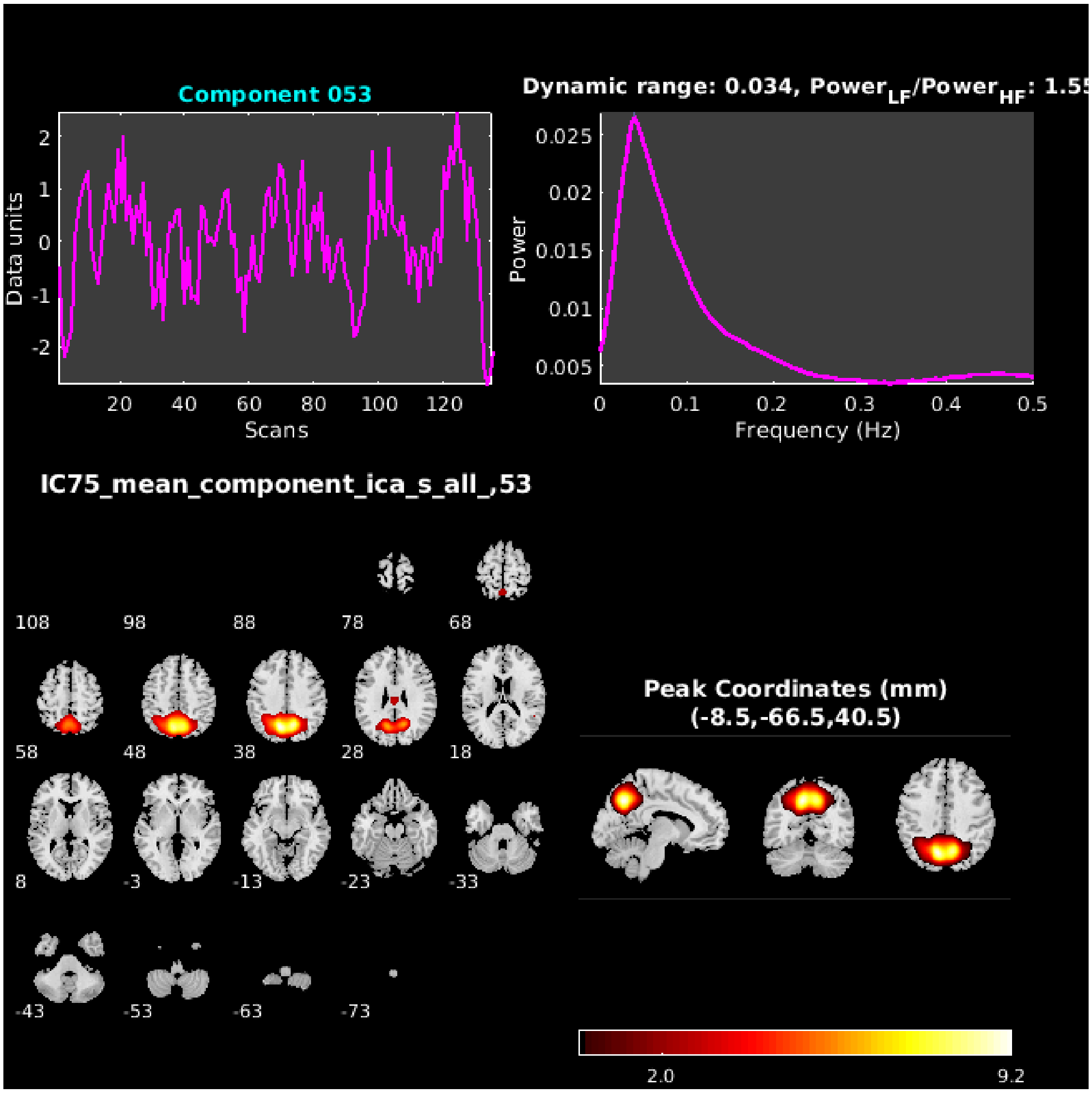
Component 053
Dynamic range: 0.034, Power$_{LF}$/Power$_{HF}$: 1.55
Data units
Scans
Power
Frequency (Hz)
IC75_mean_component_ica_s_all_,53
Peak Coordinates (mm)
(-8.5,-66.5,40.5)
2.0
9.2

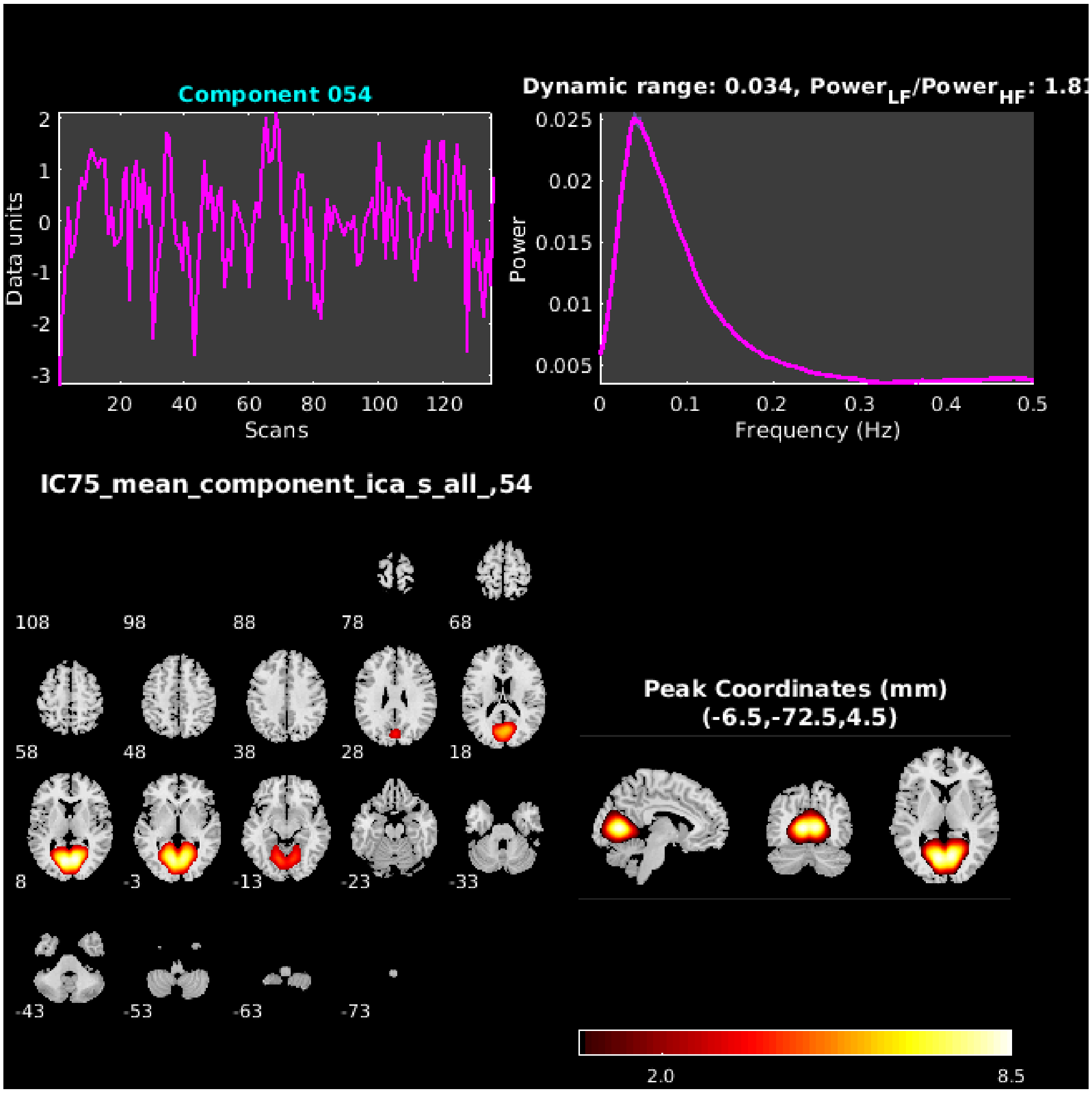

Component 054
Dynamic range: 0.034, Power$_{LF}$/Power$_{HF}$: 1.81
Data units
Scans
Power
Frequency (Hz)
IC75_mean_component_ica_s_all_,54
108
98
88
78
68
58
48
38
28
18
8
-3
-13
-23
-33
-43
-53
-63
-73
Peak Coordinates (mm)
(-6.5,-72.5,4.5)
2.0
8.5

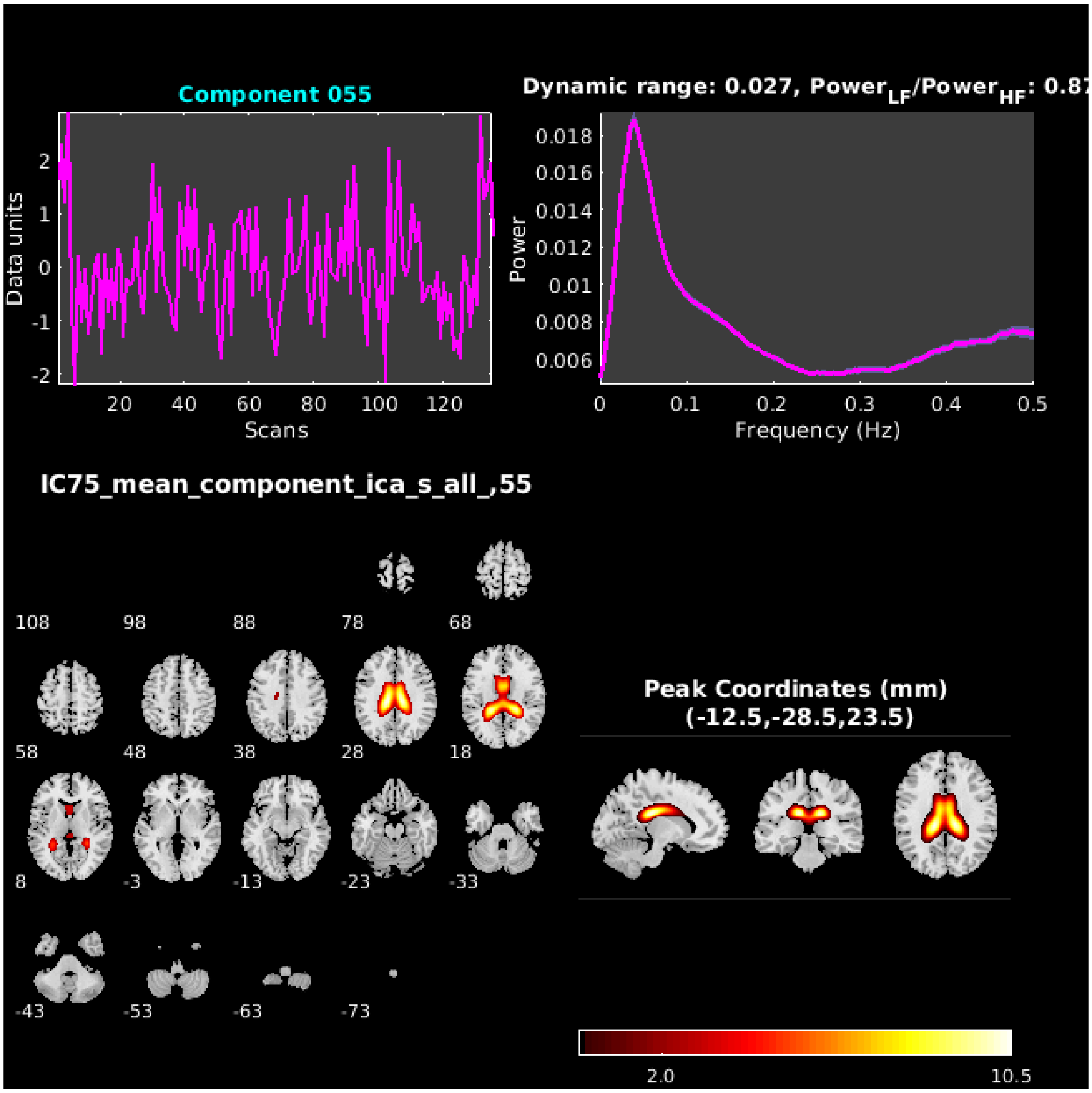
Component 055
Dynamic range: 0.027, Power_LF/Power_HF: 0.87
Data units
Scans
Power
Frequency (Hz)
IC75_mean_component_ica_s_all_,55
108
98
88
78
68
58
48
38
28
18
8
-3
-13
-23
-33
-43
-53
-63
-73
Peak Coordinates (mm)
(-12.5,-28.5,23.5)
2.0
10.5

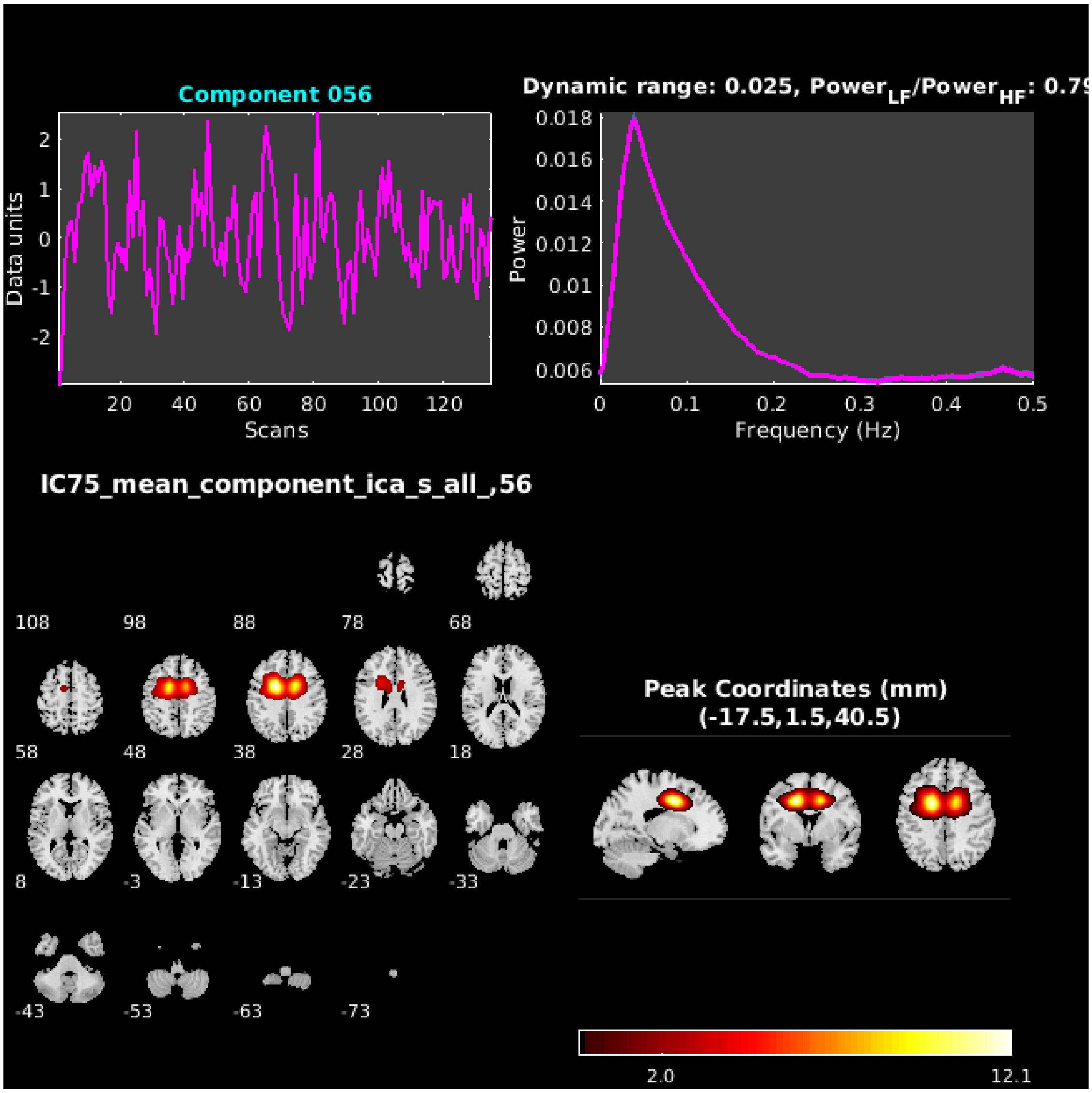

Component 056
Dynamic range: 0.025, Power_LF/Power_HF: 0.79
Data units
Scans
Power
Frequency (Hz)
IC75_mean_component_ica_s_all_,56
Peak Coordinates (mm)
(-17.5,1.5,40.5)
2.0
12.1

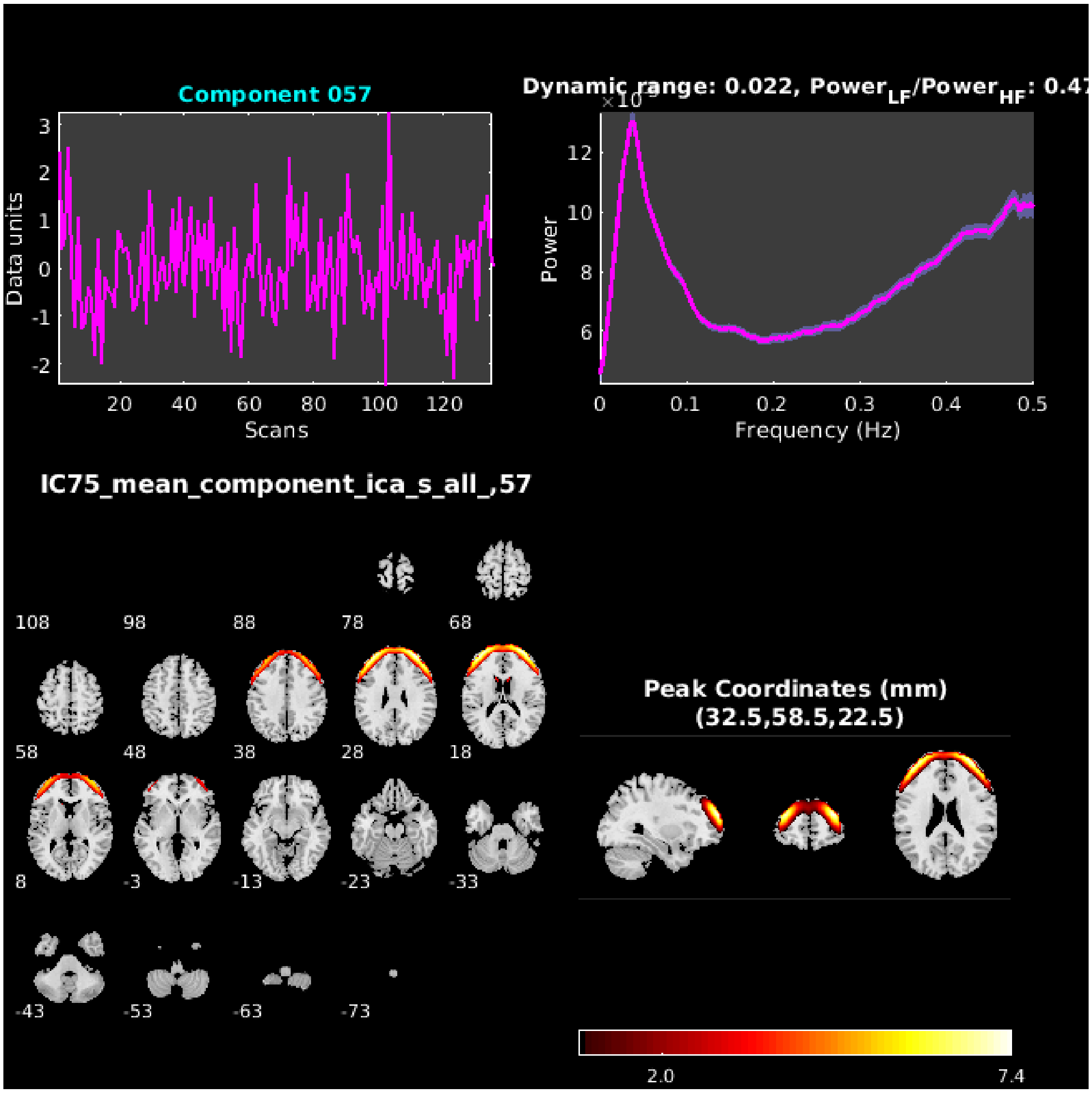
Component 057
Dynamic range: 0.022, Power_LF/Power_HF: 0.47
Data units
Scans
Power
Frequency (Hz)
IC75_mean_component_ica_s_all_,57
Peak Coordinates (mm)
(32.5,58.5,22.5)
2.0
7.4

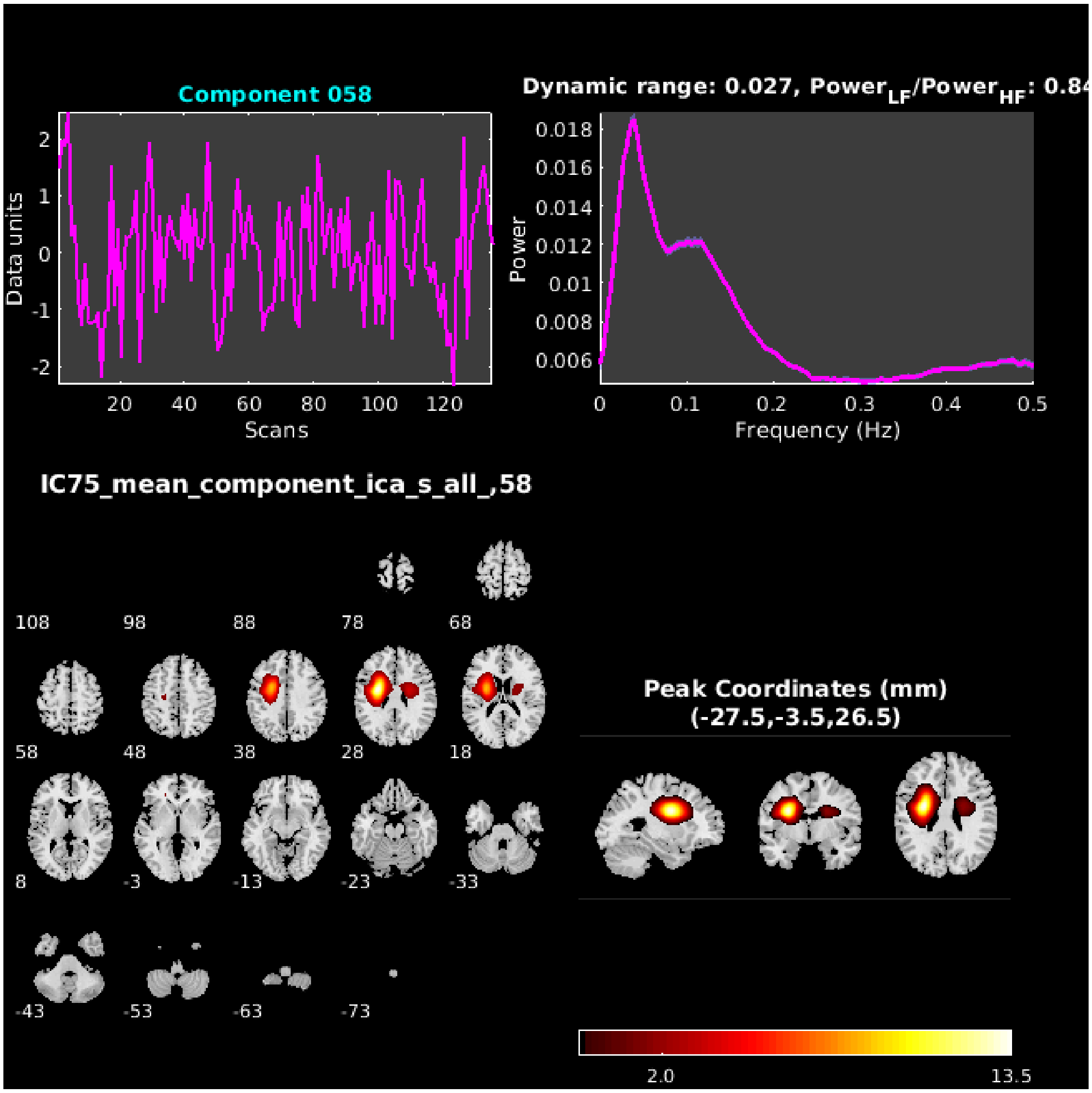
Component 058
Dynamic range: 0.027, Power_LF/Power_HF: 0.84
Data units
Scans
Power
Frequency (Hz)
IC75_mean_component_ica_s_all_,58
108
98
88
78
68
58
48
38
28
18
8
-3
-13
-23
-33
-43
-53
-63
-73
Peak Coordinates (mm)
(-27.5,-3.5,26.5)
2.0
13.5

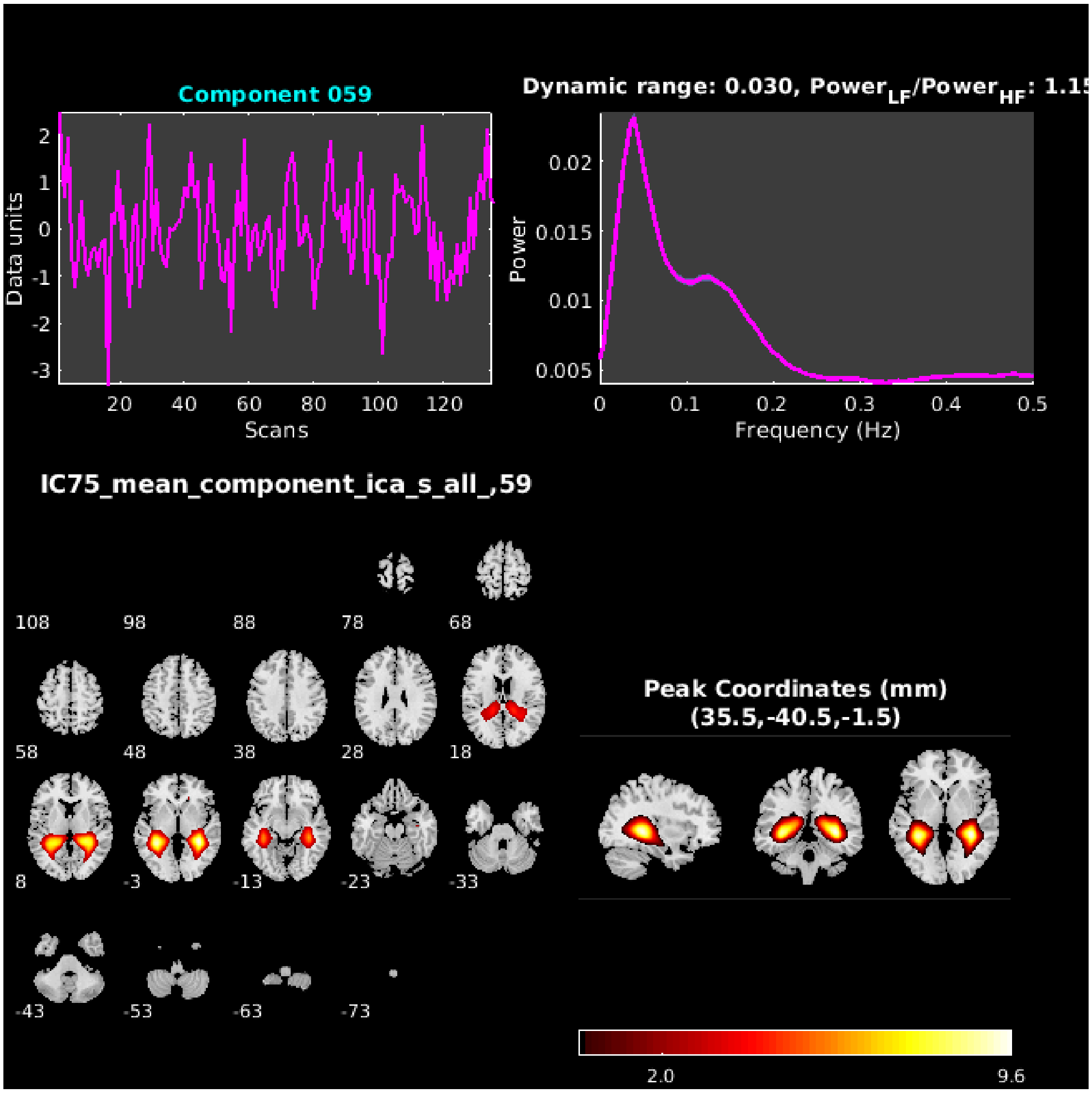

Component 059
Dynamic range: 0.030, Power_LF/Power_HF: 1.15
Data units
Scans
Power
Frequency (Hz)
IC75_mean_component_ica_s_all_,59
108
98
88
78
68
58
48
38
28
18
8
-3
-13
-23
-33
-43
-53
-63
-73
Peak Coordinates (mm)
(35.5,-40.5,-1.5)
2.0
9.6

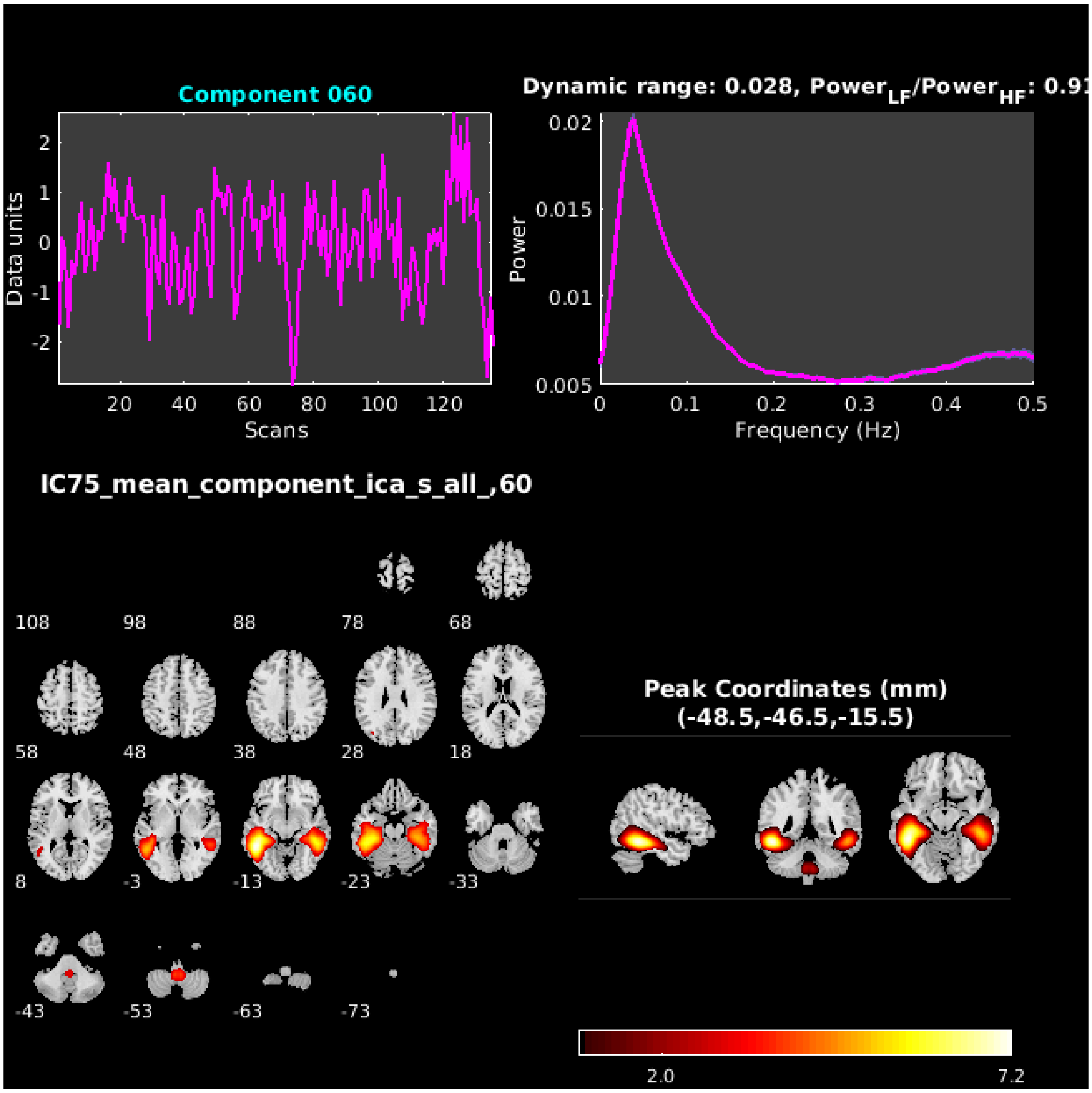
Component 060
Dynamic range: 0.028, Power$_{LF}$/Power$_{HF}$: 0.91
Data units
Scans
Power
Frequency (Hz)
IC75_mean_component_ica_s_all_,60
Peak Coordinates (mm)
(-48.5,-46.5,-15.5)
2.0
7.2

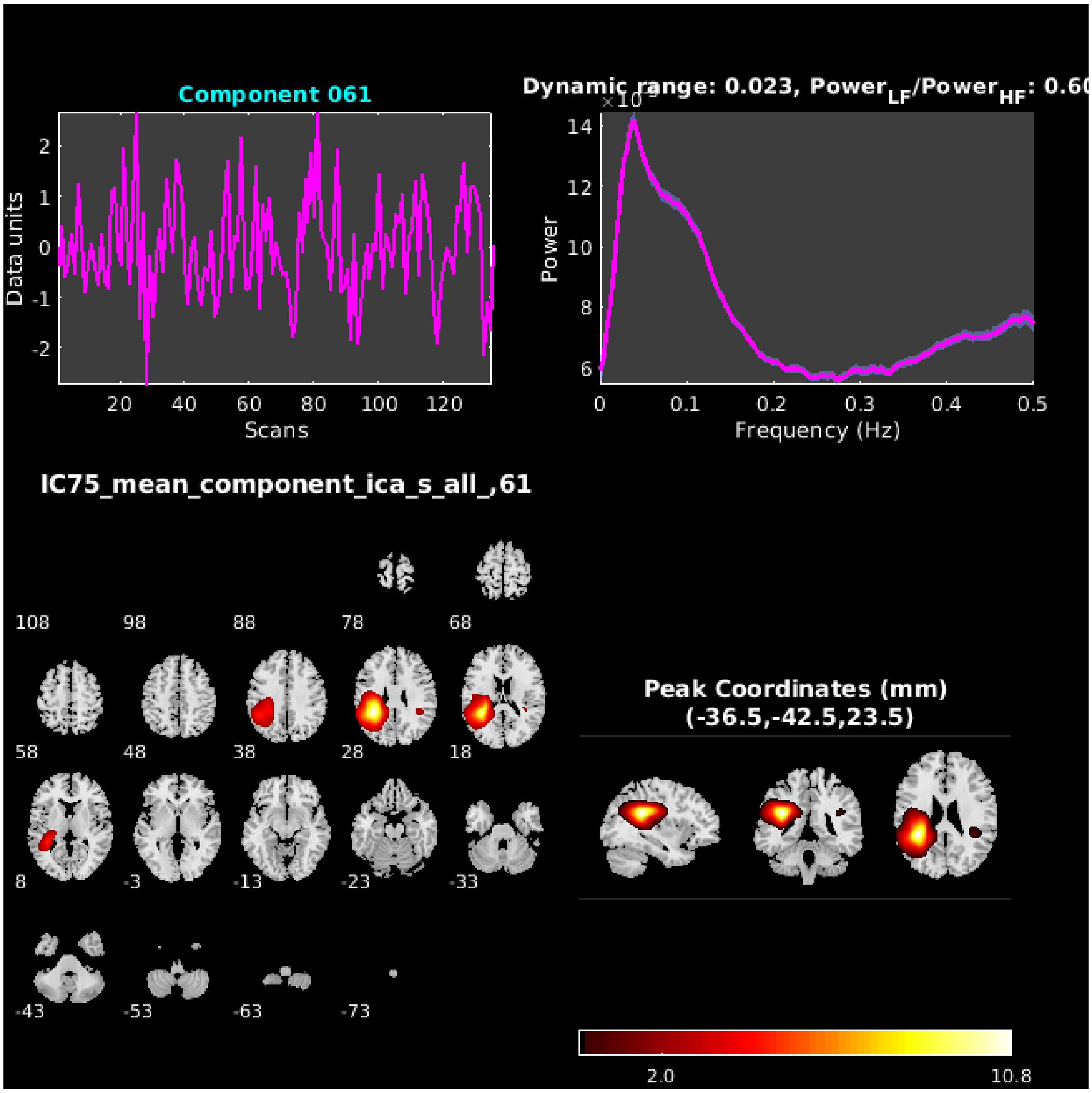
Component 061
Dynamic range: 0.023, Power$_{LF}$/Power$_{HF}$: 0.6
Data units
Scans
Power
Frequency (Hz)
IC75_mean_component_ica_s_all_,61
Peak Coordinates (mm)
(-36.5,-42.5,23.5)
2.0
10.8

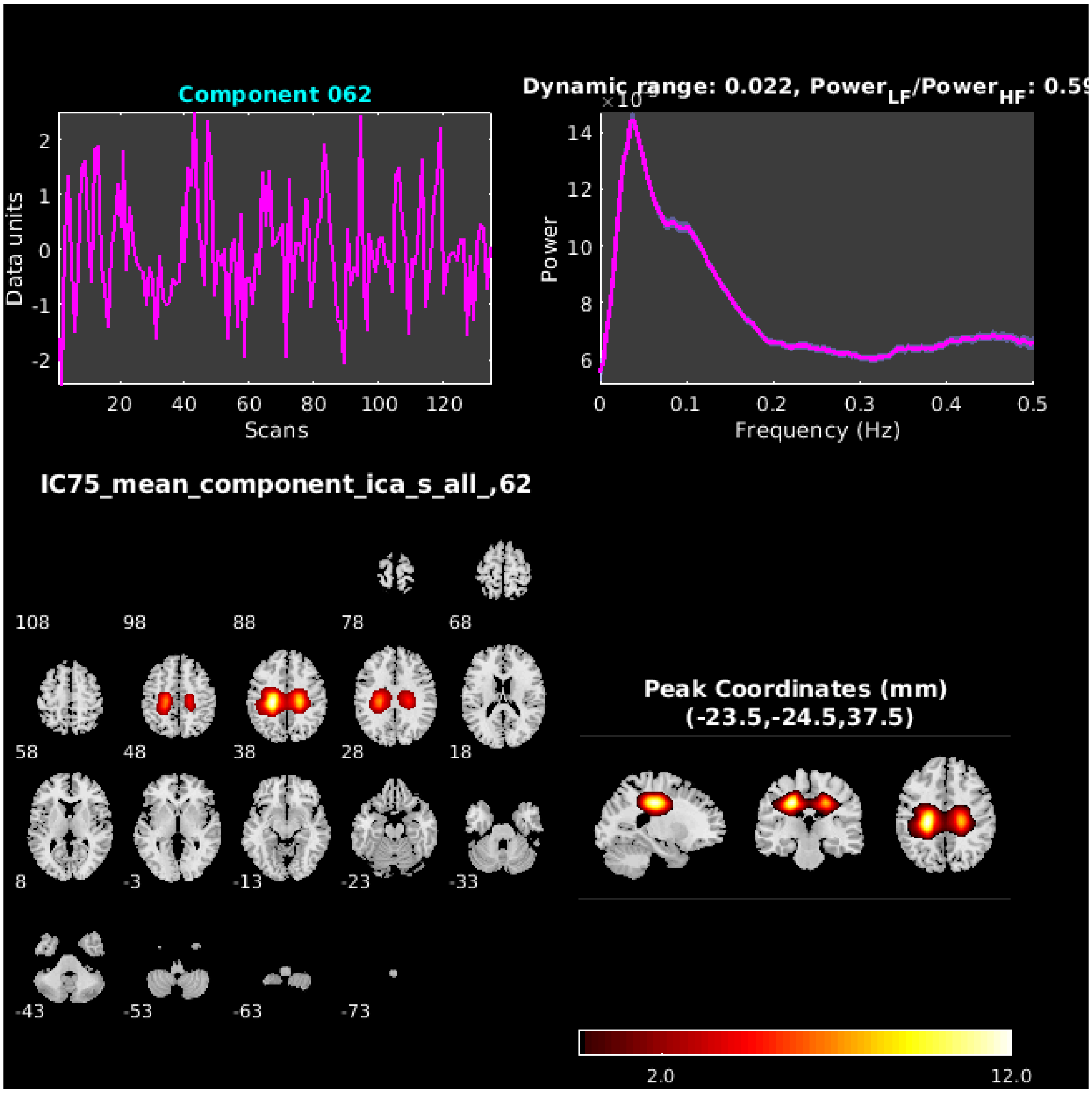

Component 062
Dynamic range: 0.022, Power$_{LF}$/Power$_{HF}$: 0.59
Data units
Scans
Power
Frequency (Hz)
IC75_mean_component_ica_s_all_,62
Peak Coordinates (mm)
(-23.5,-24.5,37.5)
2.0
12.0

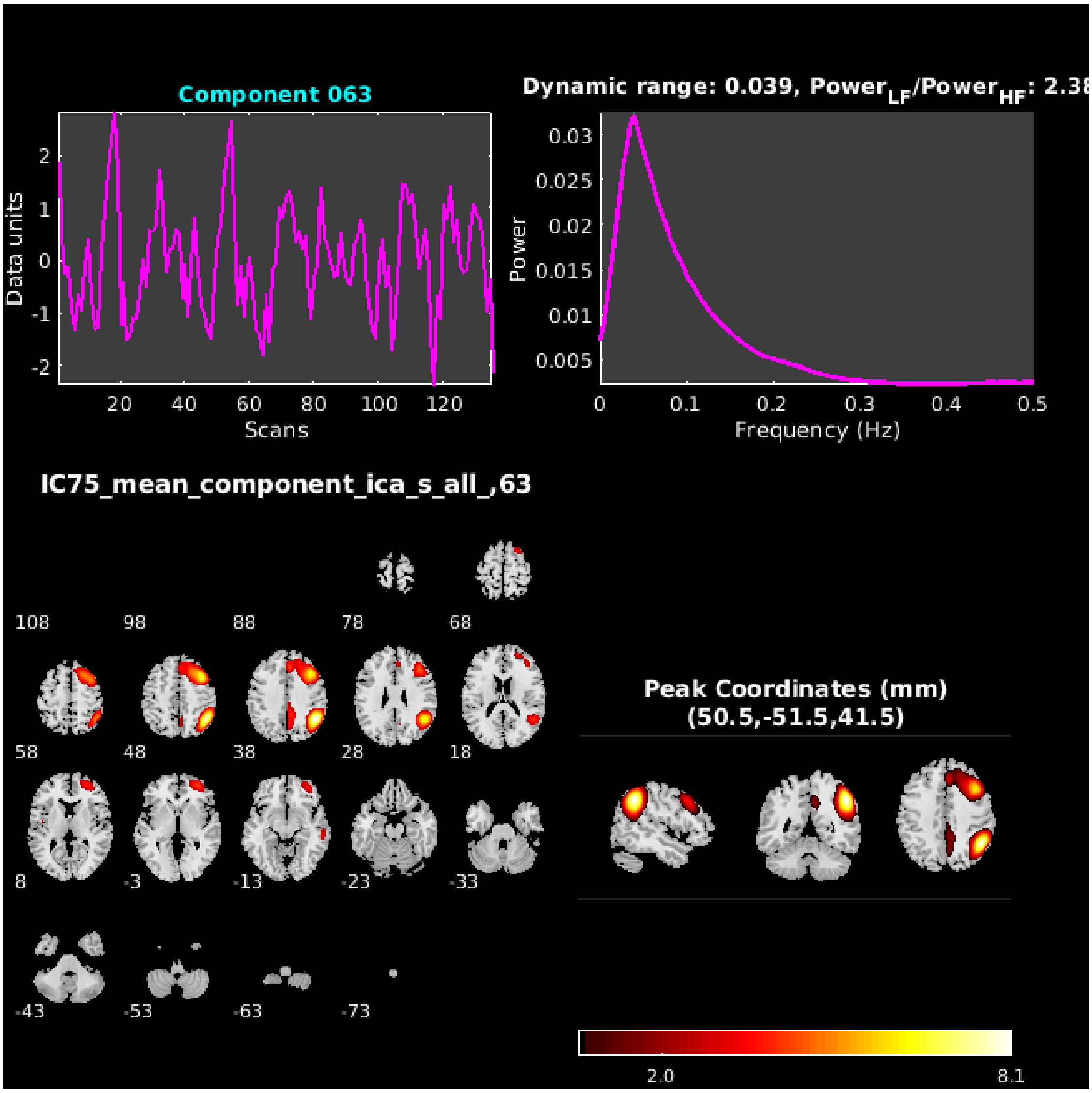

Component 063
Dynamic range: 0.039, Power_LF/Power_HF: 2.38
Data units
Scans
Power
Frequency (Hz)
IC75_mean_component_ica_s_all_,63
108
98
88
78
68
58
48
38
28
18
8
-3
-13
-23
-33
-43
-53
-63
-73
Peak Coordinates (mm)
(50.5,-51.5,41.5)
2.0
8.1

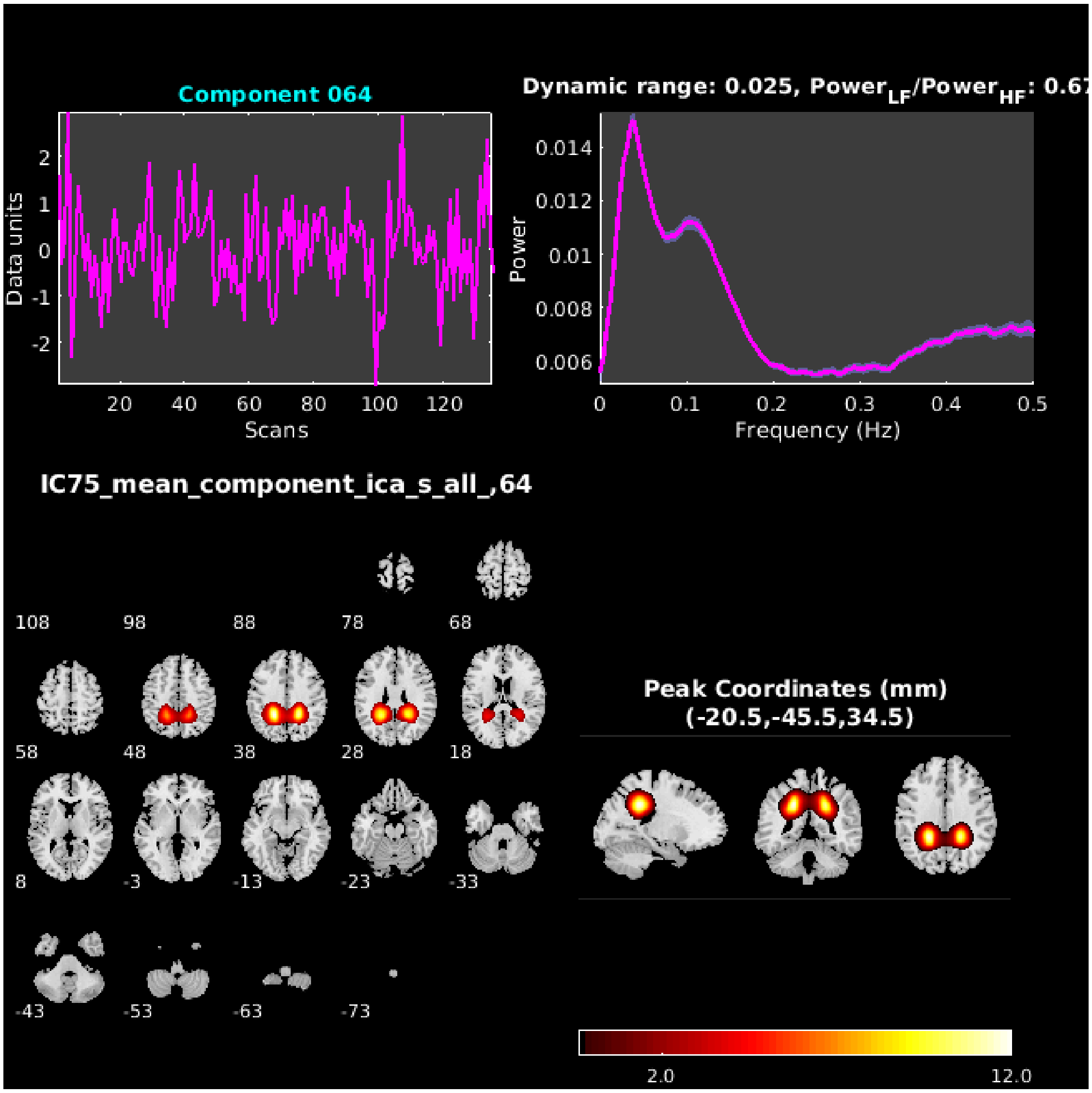
Component 064
Dynamic range: 0.025, Power$_{LF}$/Power$_{HF}$: 0.67
Data units
Scans
Power
Frequency (Hz)
IC75_mean_component_ica_s_all_,64
Peak Coordinates (mm)
(-20.5,-45.5,34.5)
2.0
12.0

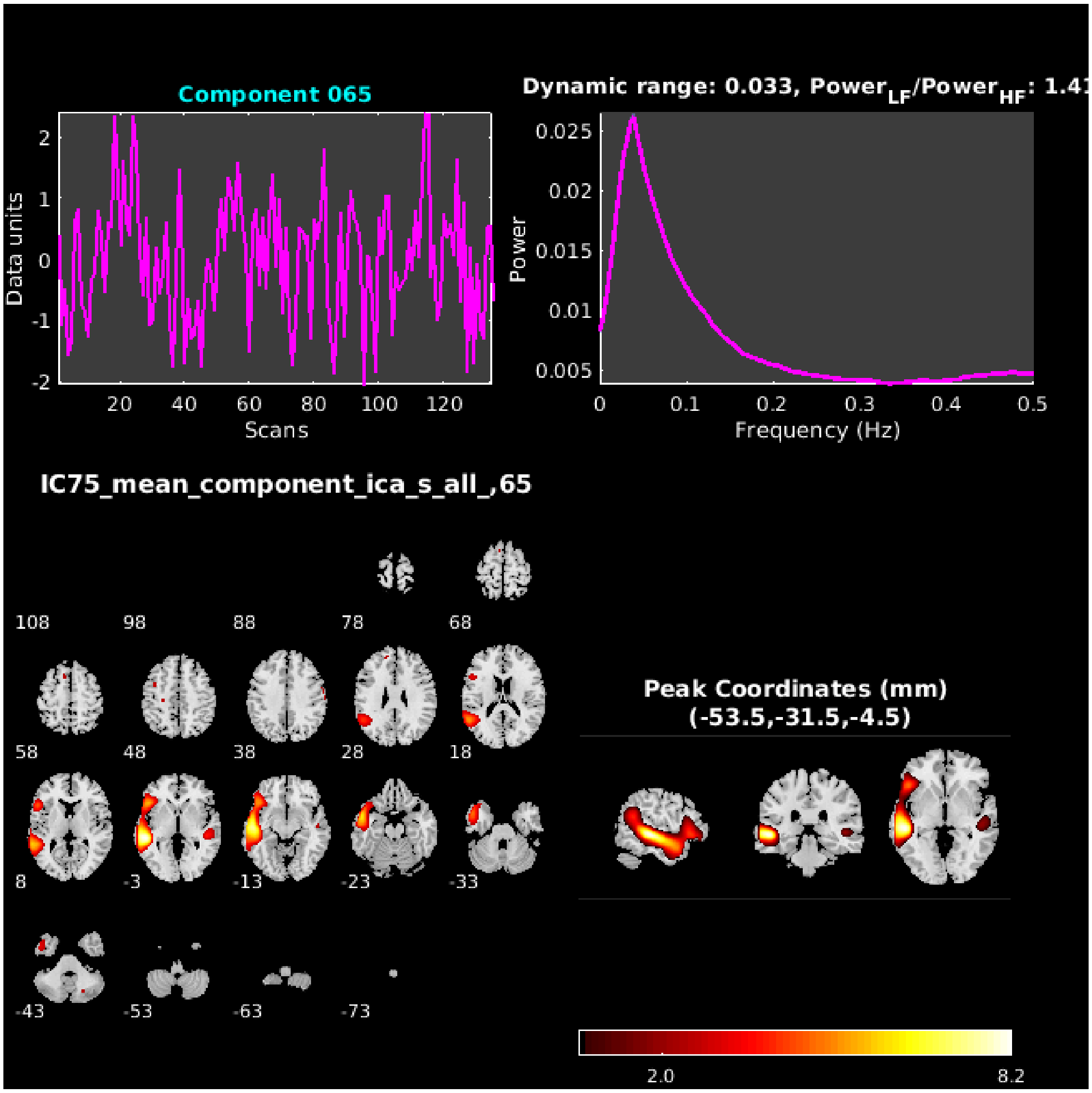

Component 065
Dynamic range: 0.033, Power$_{LF}$/Power$_{HF}$: 1.41
Data units
Scans
Power
Frequency (Hz)
IC75_mean_component_ica_s_all_,65
108
98
88
78
68
58
48
38
28
18
8
-3
-13
-23
-33
-43
-53
-63
-73
Peak Coordinates (mm)
(-53.5,-31.5,-4.5)
2.0
8.2

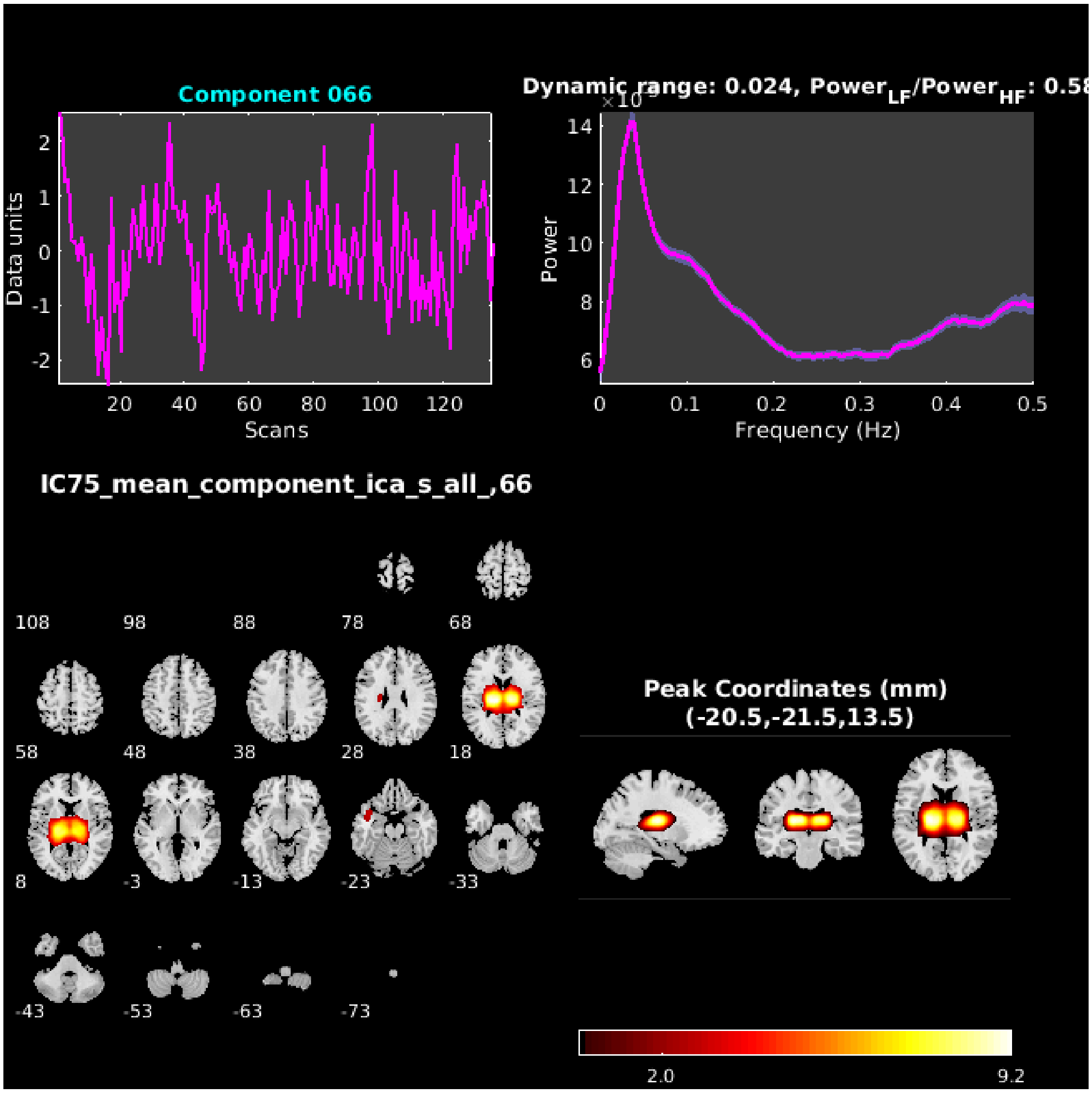

Component 066
Dynamic range: 0.024, Power$_{LF}$/Power$_{HF}$: 0.58
Data units
Scans
Power
Frequency (Hz)
IC75_mean_component_ica_s_all_,66
Peak Coordinates (mm)
(-20.5,-21.5,13.5)
2.0
9.2

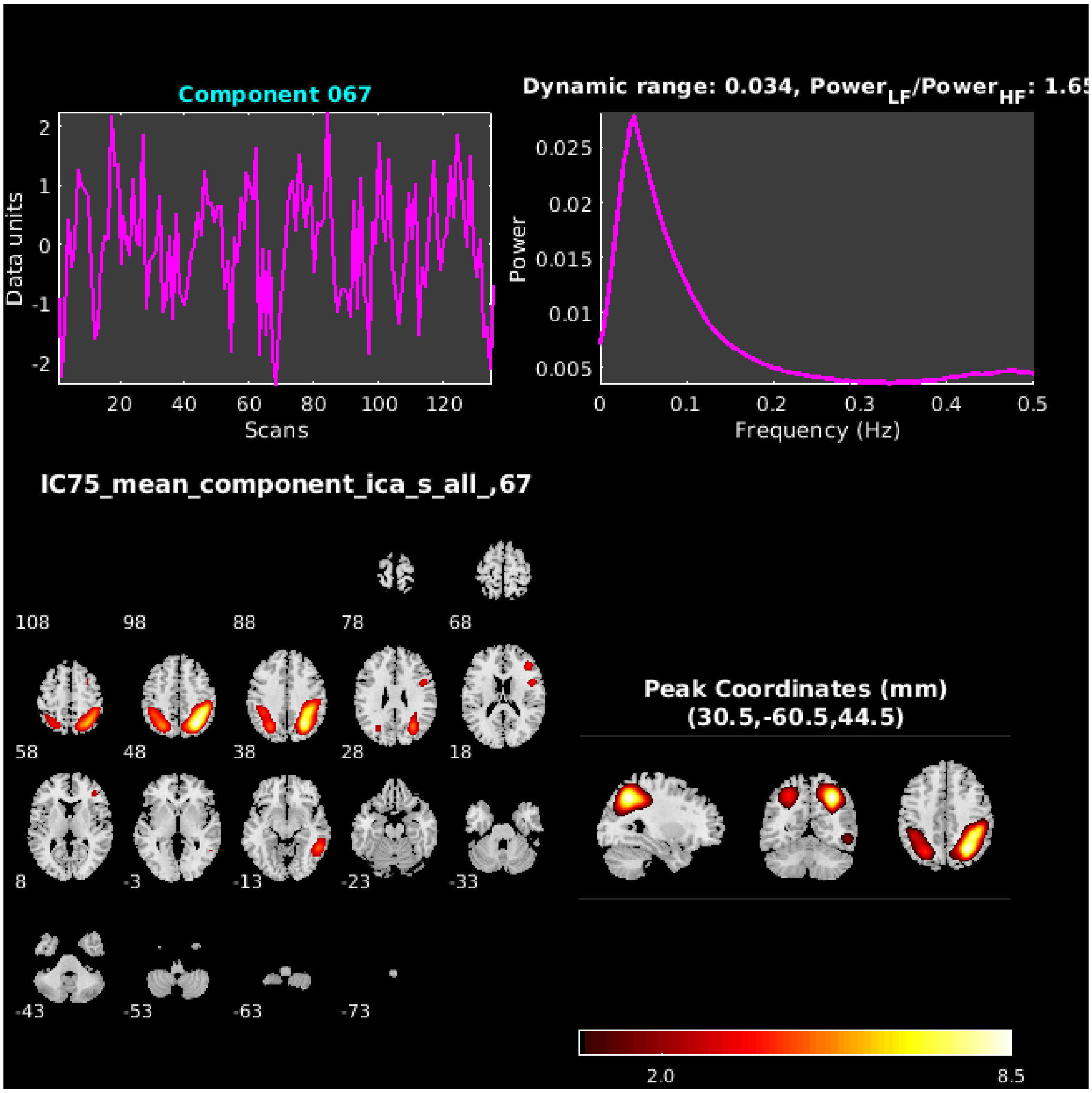
Component 067
Dynamic range: 0.034, Power_LF/Power_HF: 1.65
Data units
Scans
Power
Frequency (Hz)
IC75_mean_component_ica_s_all_,67
108
98
88
78
68
58
48
38
28
18
8
-3
-13
-23
-33
-43
-53
-63
-73
Peak Coordinates (mm)
(30.5,-60.5,44.5)
2.0
8.5

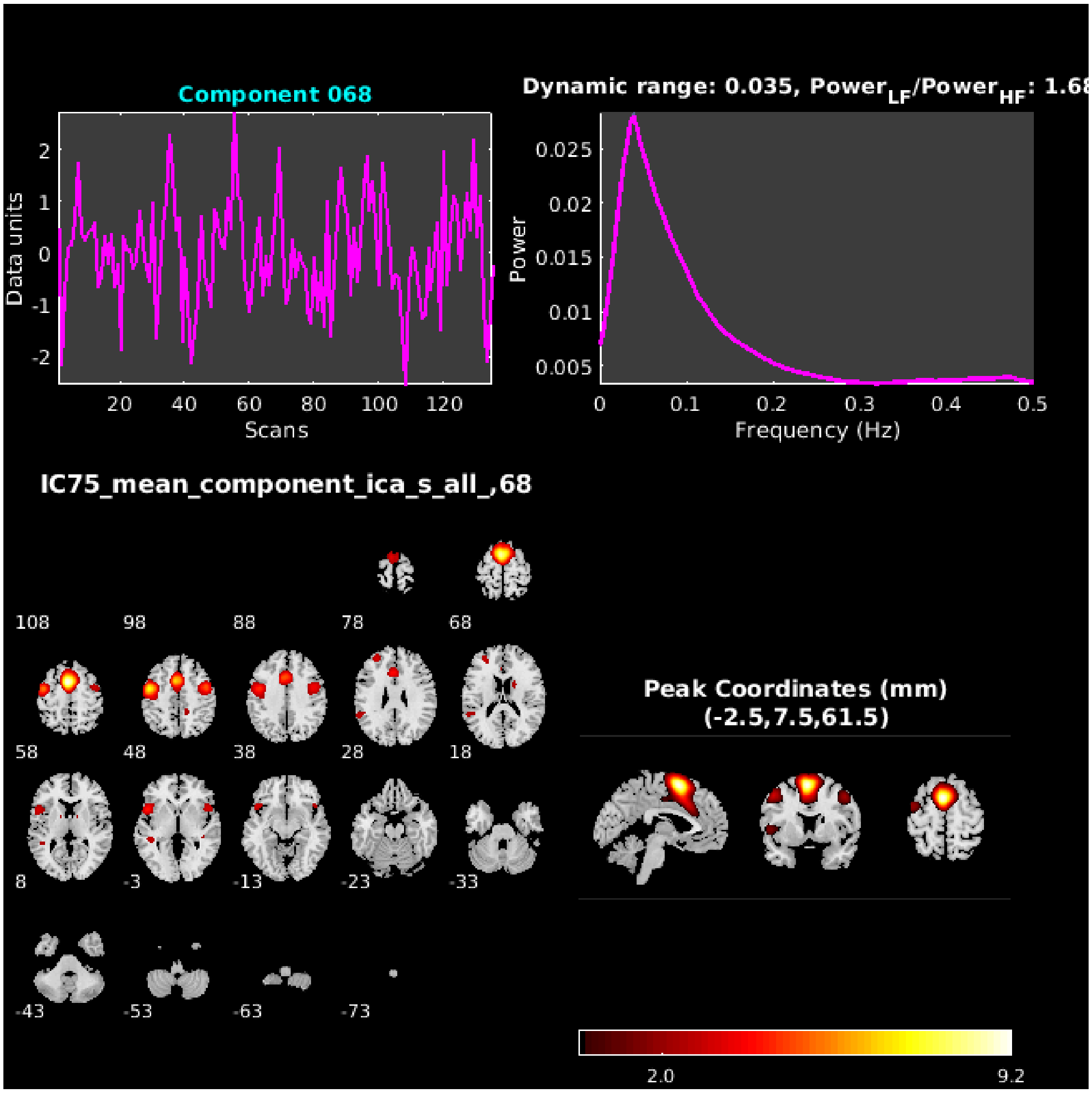
Component 068
Dynamic range: 0.035, Power_LF/Power_HF: 1.68
Data units
Scans
Power
Frequency (Hz)
IC75_mean_component_ica_s_all_,68
Peak Coordinates (mm)
(-2.5,7.5,61.5)
2.0
9.2

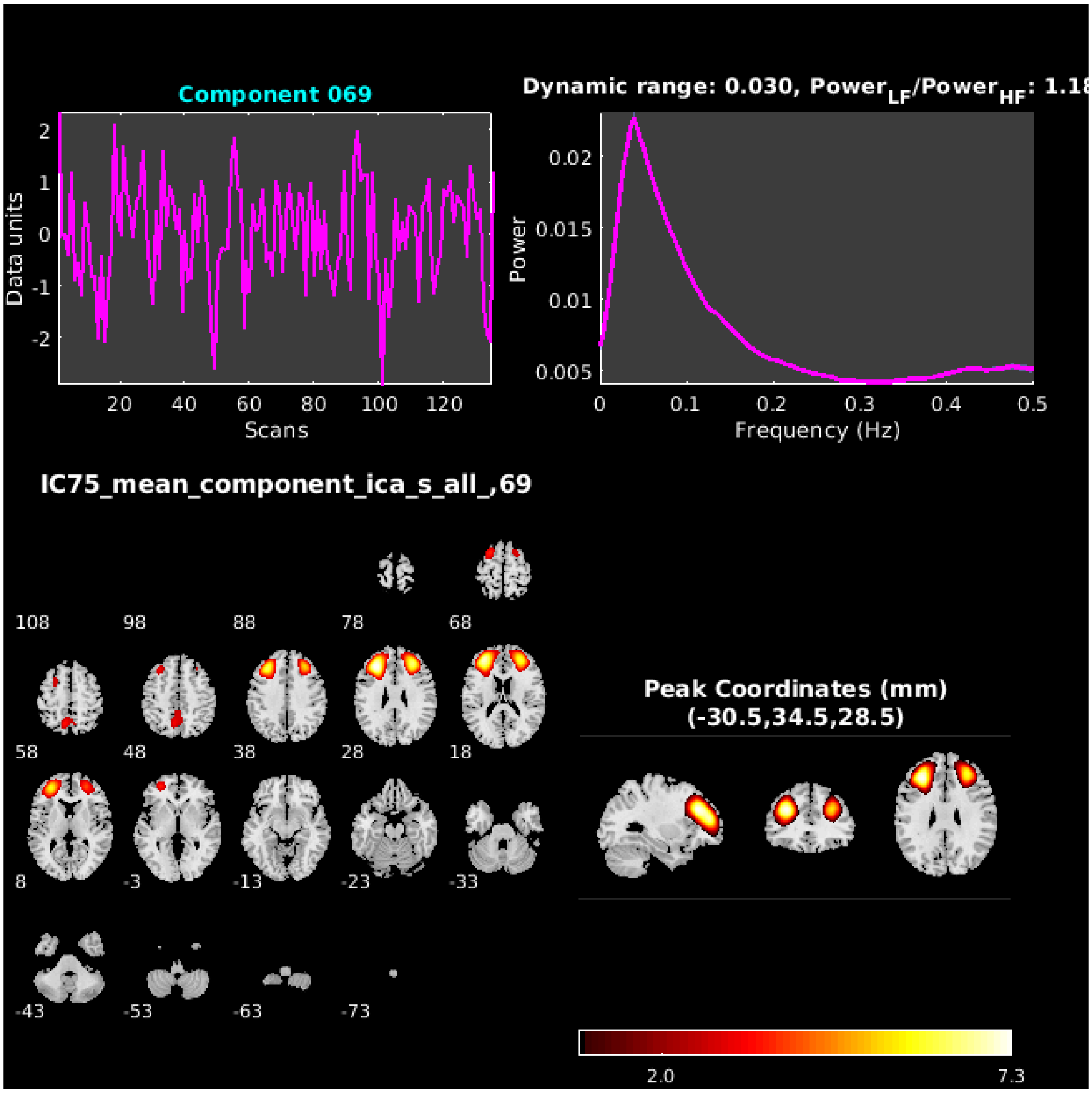
Component 069
Dynamic range: 0.030, Power$_{LF}$/Power$_{HF}$: 1.18
Data units
Scans
Power
Frequency (Hz)
IC75_mean_component_ica_s_all_,69
Peak Coordinates (mm)
(-30.5,34.5,28.5)
2.0
7.3

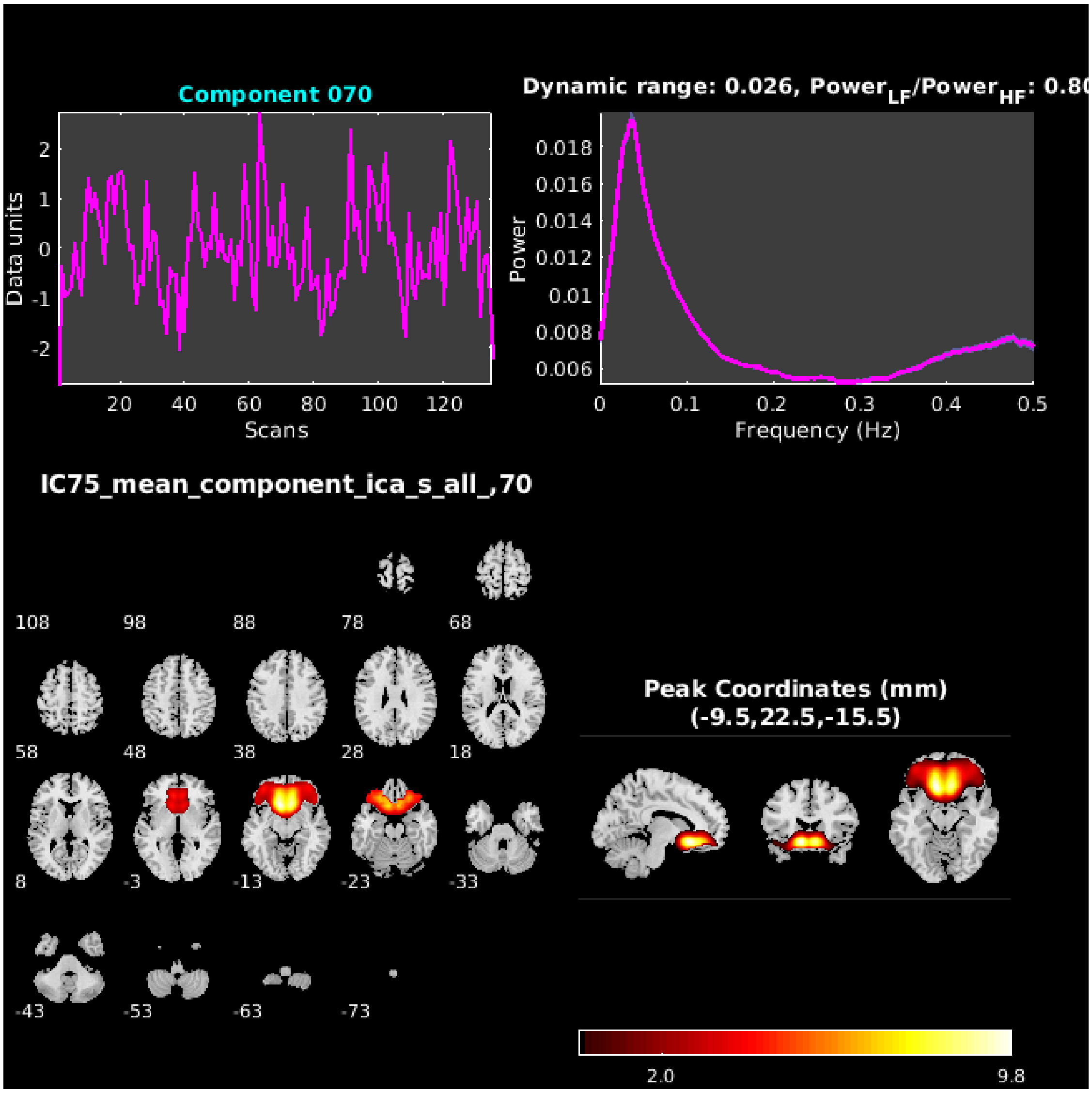
Component 070
Dynamic range: 0.026, Power$_{LF}$/Power$_{HF}$: 0.80
Data units
Scans
Power
Frequency (Hz)
IC75_mean_component_ica_s_all_,70
Peak Coordinates (mm)
(-9.5,22.5,-15.5)
2.0
9.8

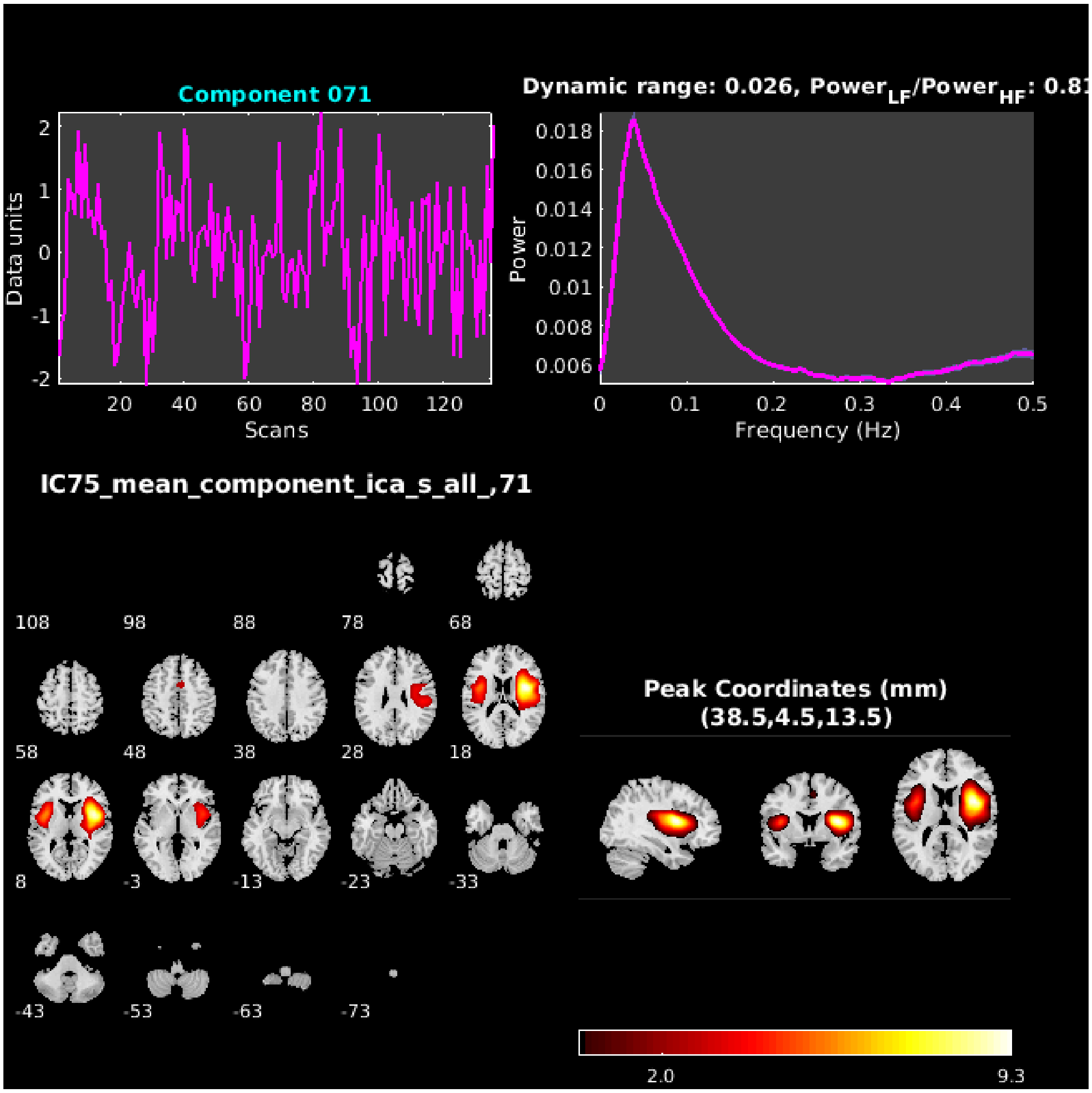
Component 071
Dynamic range: 0.026, Power_LF/Power_HF: 0.81
Data units
Scans
Power
Frequency (Hz)
IC75_mean_component_ica_s_all_,71
Peak Coordinates (mm)
(38.5,4.5,13.5)
2.0
9.3

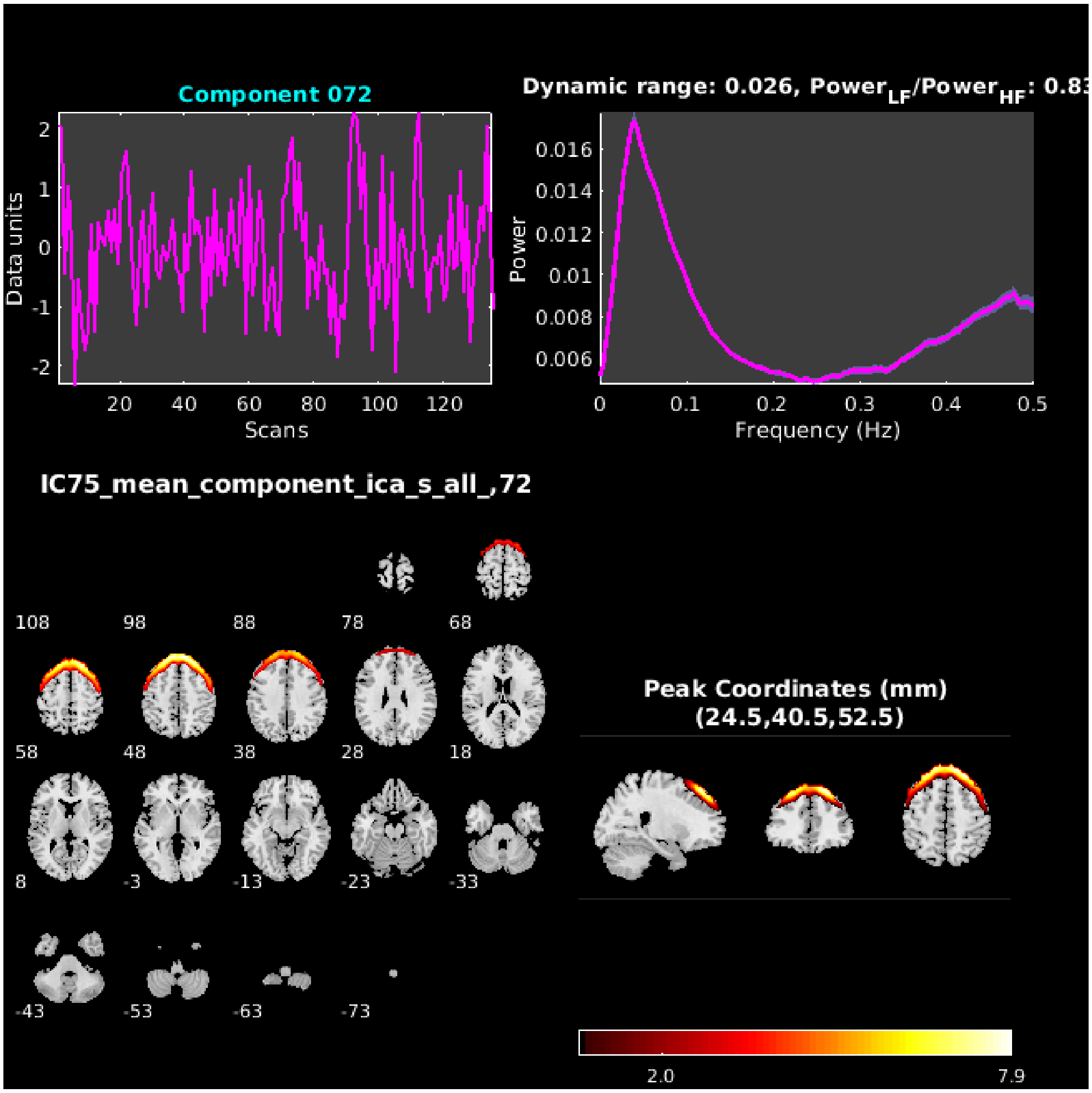
Component 072
Dynamic range: 0.026, Power_LF/Power_HF: 0.83
Data units
Scans
Power
Frequency (Hz)
IC75_mean_component_ica_s_all_,72
Peak Coordinates (mm)
(24.5,40.5,52.5)
2.0
7.9

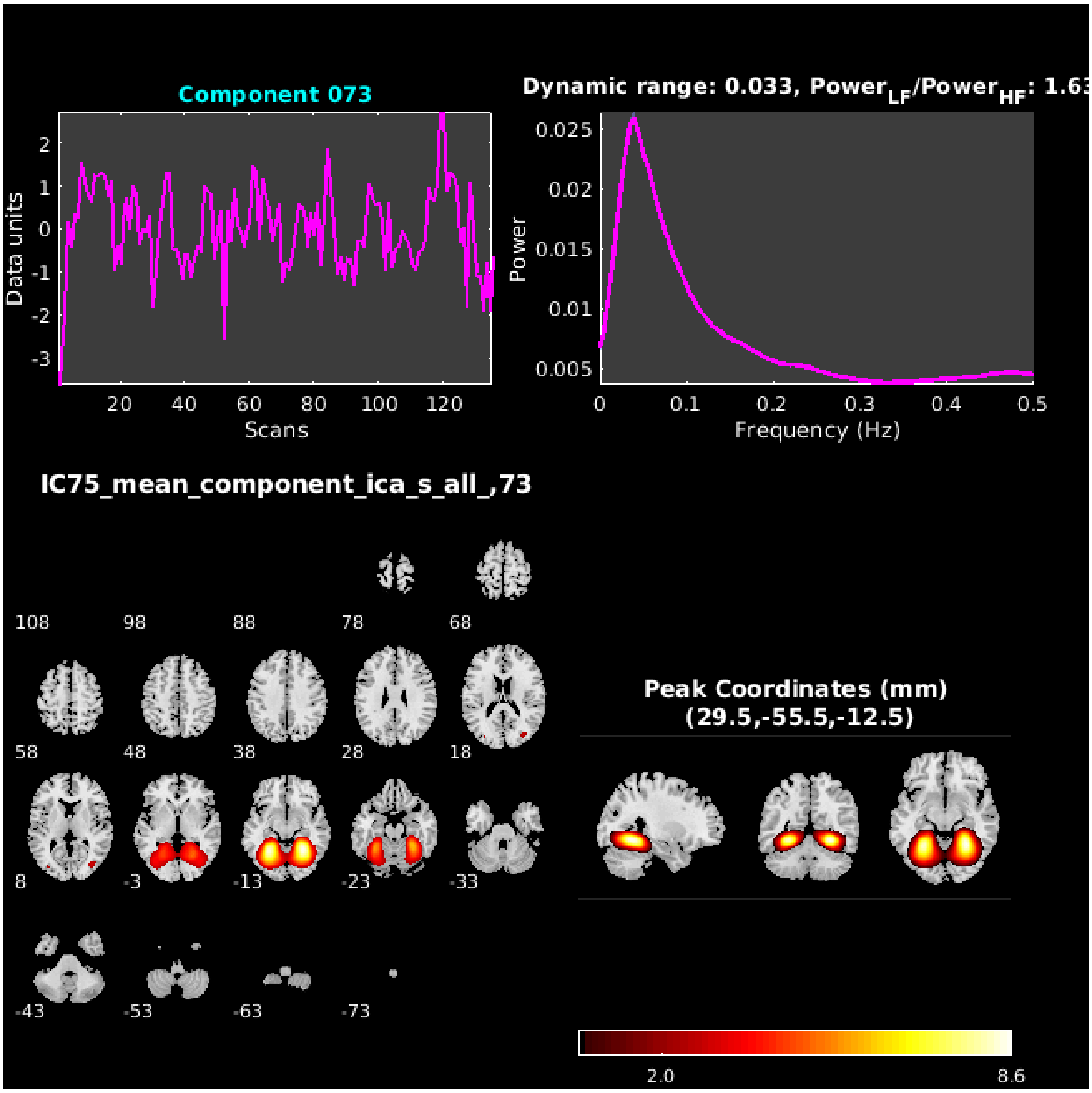
Component 073
Dynamic range: 0.033, Power$_{LF}$/Power$_{HF}$: 1.63
Data units
Scans
Power
Frequency (Hz)
IC75_mean_component_ica_s_all_,73
Peak Coordinates (mm)
(29.5,-55.5,-12.5)
2.0
8.6

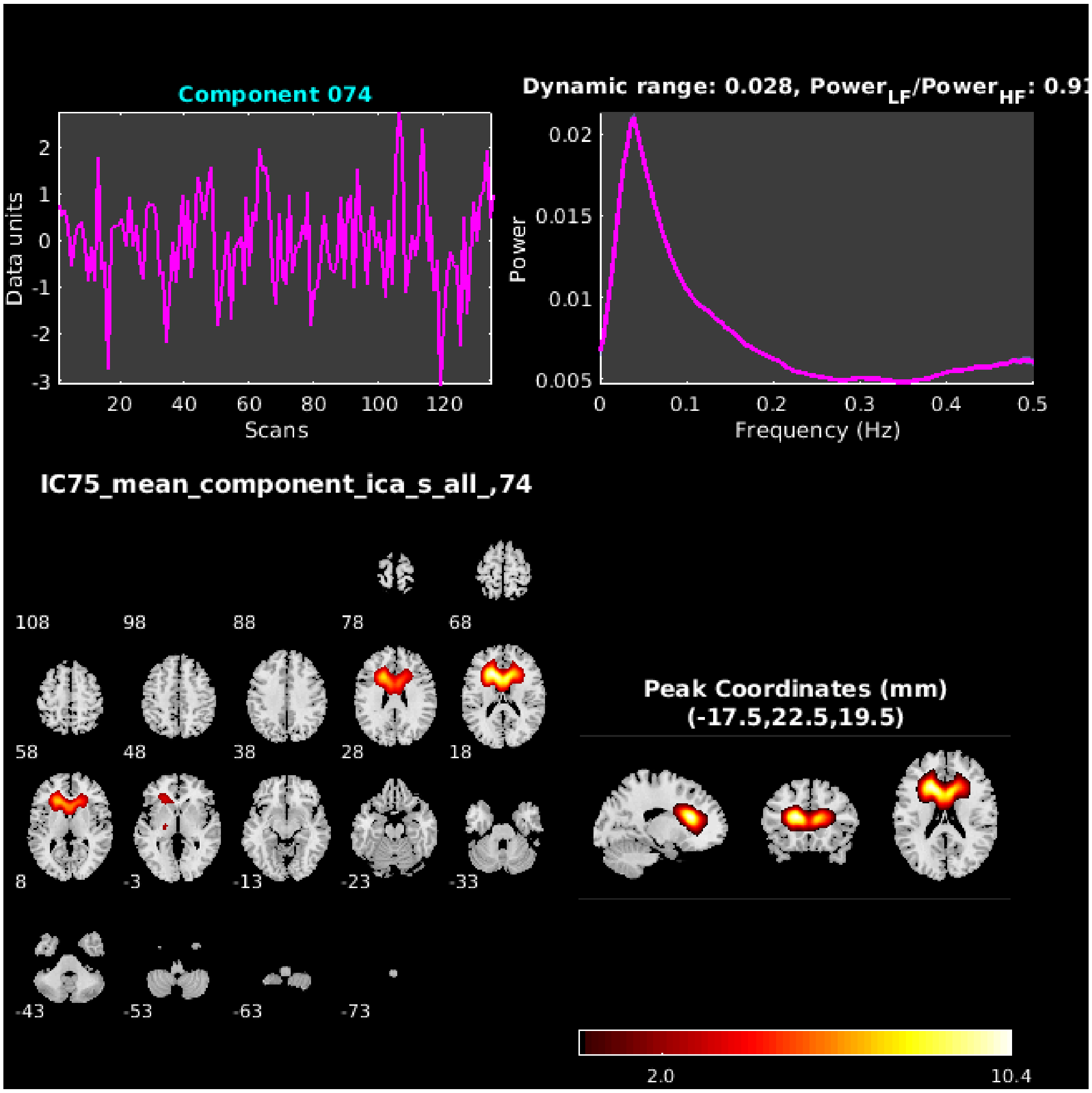
Component 074
Dynamic range: 0.028, PowerLF/PowerHF: 0.9
Data units
Scans
Power
Frequency (Hz)
IC75_mean_component_ica_s_all_,74
Peak Coordinates (mm)
(-17.5,22.5,19.5)
2.0
10.4

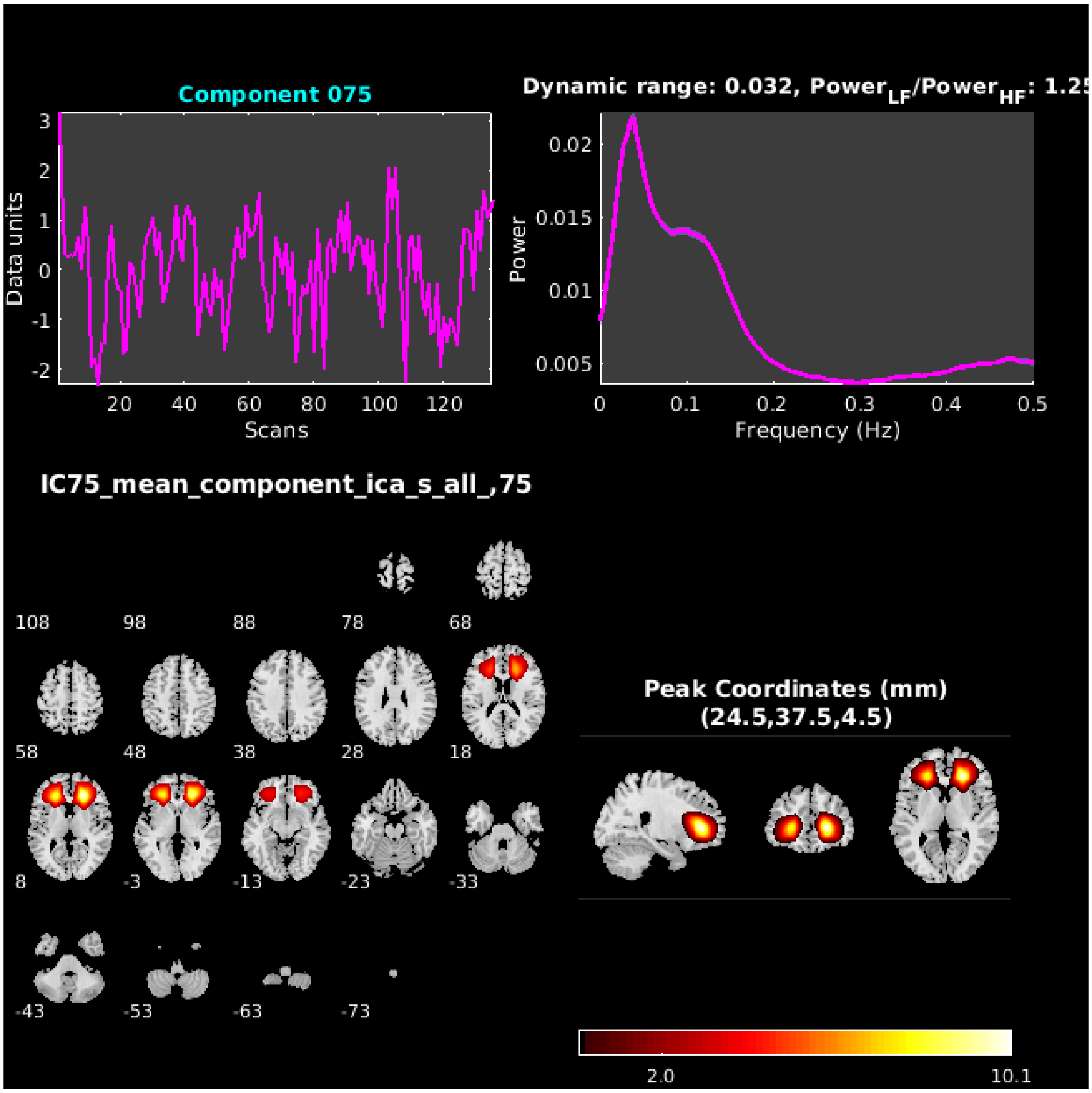

Spectral Summary

- **a) dynamic_range** - Difference between the peak power and minimum power at frequencies to the right of the peak.
- **b) fALFF** - Low frequency to high frequency power ratio.

ComponentNumber	*DynamicRange*	*fALFF*
1	*0.024273*	*0.64118*
2	*0.028927*	*0.98709*
3	*0.032782*	*1.5777*
4	*0.022337*	*0.46275*
5	*0.035277*	*1.8631*
6	*0.024453*	*0.47638*
7	*0.030407*	*1.0957*

8	0.02788	0.81255
9	0.023625	0.63575
10	0.022137	0.51062
11	0.032614	1.5097
12	0.026933	0.8304
13	0.035862	1.9883
14	0.02252	0.58947
15	0.026542	0.8266
16	0.026015	0.84406
17	0.02643	0.78114
18	0.022699	0.53067
19	0.02612	0.77641
20	0.025804	0.79192
21	0.02667	0.78863
22	0.024905	0.6785
23	0.027235	0.9653
24	0.023059	0.62676
25	0.024728	0.77806
26	0.031556	1.3594
27	0.030017	1.1344
28	0.035086	2.0027
29	0.026934	0.80755
30	0.032493	1.4475
31	0.026033	0.73074
32	0.022147	0.49335
33	0.028569	1.0488
34	0.027635	0.94692
35	0.035935	1.831
36	0.023936	0.63906
37	0.02778	1.0064
38	0.026601	0.87989
39	0.027786	0.99086
40	0.024631	0.71684
41	0.025184	0.81307
42	0.030648	1.1879
43	0.037776	2.1903
44	0.034196	1.5715
45	0.028243	1.0361
46	0.037084	2.3198
47	0.03148	1.1907
48	0.025246	0.8331
49	0.03149	1.2594
50	0.025235	0.73522
51	0.02989	1.1583
52	0.037772	2.154
53	0.033808	1.5553
54	0.034138	1.814
55	0.02728	0.87365
56	0.024762	0.7937
57	0.022096	0.47105
58	0.026797	0.84835
59	0.030315	1.1573
60	0.027851	0.91397
61	0.022891	0.60395

62	0.022468	0.59337
63	0.038725	2.3867
64	0.024866	0.67258
65	0.032679	1.4114
66	0.023748	0.57978
67	0.034273	1.6573
68	0.034828	1.6874
69	0.030354	1.1863
70	0.026276	0.80858
71	0.025531	0.81562
72	0.025909	0.83326
73	0.033144	1.6335
74	0.027734	0.91777
75	0.031529	1.2589

Temporal Stats On Beta Weights

Multiple regression is done using the timecourses from SPM design matrix as model and ICA timecourses as observations. R^2 values for each component are shown in bar plot. For each component, one sample t-test results of each session and condition are shown in the bar plots.

Kurtosis of timecourses and spatial maps

Mean across subjects is reported in table. Figure shows mean+/- SEM across subjects

ComponentNumber	Timecourses	SpatialMaps
1	3.6394	12.378
2	4.052	6.628
3	4.2579	6.9551
4	3.6122	8.6491
5	4.0666	5.2046
6	4.9587	5.4083
7	4.4047	4.4972
8	5.1296	4.9858
9	4.1934	6.6734
10	4.2286	5.3182
11	4.323	5.6196
12	4.6284	5.622
13	4.3003	5.1625
14	4.4406	5.9467
15	4.6573	4.5878
16	5.2641	5.5108
17	4.5805	4.34
18	4.8462	5.7136
19	5.3051	4.6589
20	4.4114	4.7234
21	4.2026	4.6654
22	4.4372	4.2273
23	4.8948	4.2613
24	4.2776	6.0599

25	4.7109	4.1804
26	4.8774	4.2949
27	5.4241	4.4668
28	4.3765	4.4545
29	4.1988	5.0573
30	3.8702	4.2526
31	4.8006	3.9306
32	4.6018	4.9394
33	4.0905	4.1592
34	5.2171	4.3123
35	4.7853	4.0433
36	4.4888	4.6067
37	4.3598	4.1552
38	5.2049	4.5459
39	4.617	4.0997
40	5.1839	4.1216
41	4.2562	4.351
42	4.5021	3.9765
43	3.6832	4.4608
44	4.487	4.3185
45	3.7905	4.2087
46	4.5845	5.2048
47	3.7326	3.8583
48	4.5965	4.3631
49	5.8897	4.6662
50	3.8749	4.2283
51	4.2162	4.5346
52	3.3629	4.7036
53	4.4092	4.2384
54	4.0453	4.917
55	4.2284	4.8337
56	4.3643	4.845
57	6.8673	4.0574
58	3.8472	5.1474
59	4.0784	4.5151
60	4.13	3.8264
61	4.924	4.3848
62	3.7706	4.7969
63	3.4505	4.1291
64	4.011	4.9219
65	4.3204	4.0139
66	4.3054	4.3592
67	4.1235	4.06
68	3.5591	3.853
69	4.1544	3.8492
70	5.2692	4.8239
71	5.0015	4.1147
72	5.7315	4.2011
73	4.6017	4.3396
74	4.5022	4.692
75	5.0457	4.5132

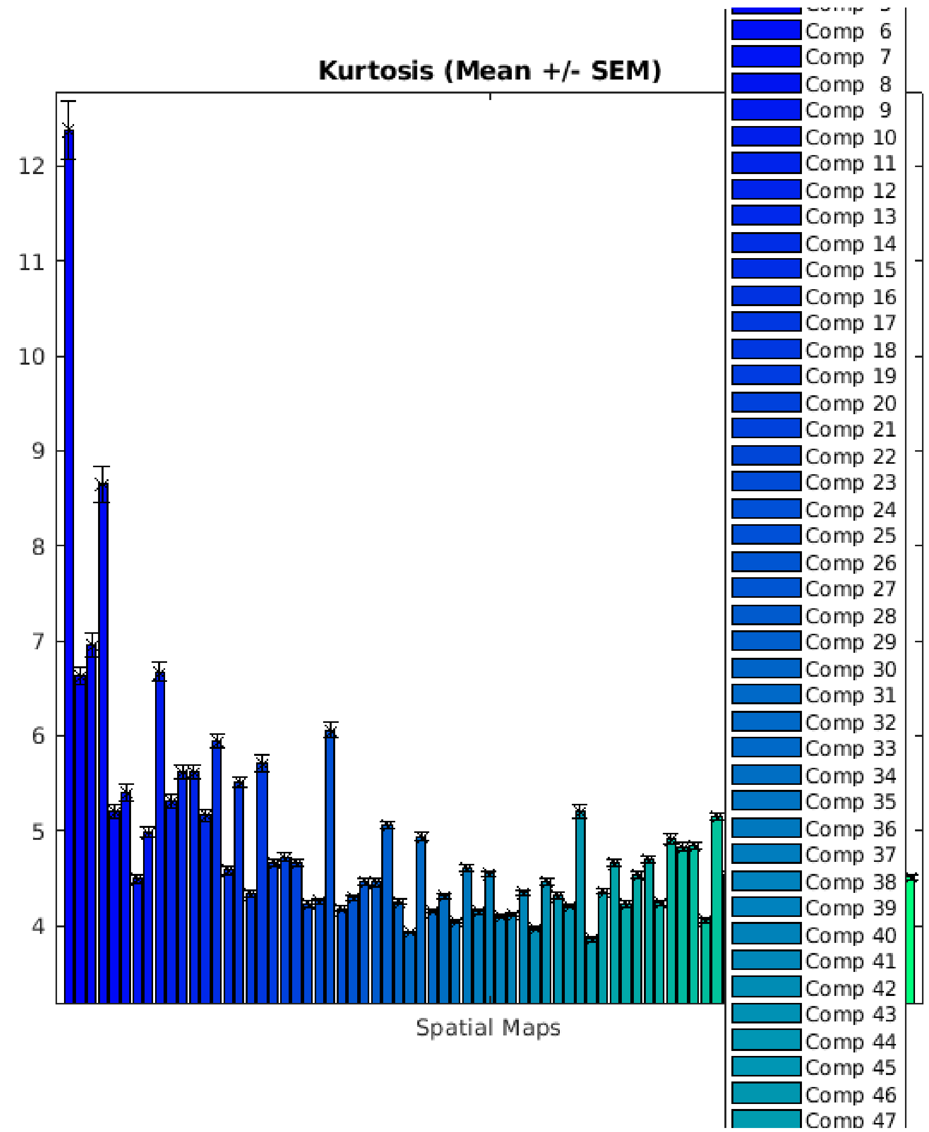

Kurtosis (Mean +/- SEM)
12
11
10
9
8
7
6
5
4
Spatial Maps
Comp 6
Comp 7
Comp 8
Comp 9
Comp 10
Comp 11
Comp 12
Comp 13
Comp 14
Comp 15
Comp 16
Comp 17
Comp 18
Comp 19
Comp 20
Comp 21
Comp 22
Comp 23
Comp 24
Comp 25
Comp 26
Comp 27
Comp 28
Comp 29
Comp 30
Comp 31
Comp 32
Comp 33
Comp 34
Comp 35
Comp 36
Comp 37
Comp 38
Comp 39
Comp 40
Comp 41
Comp 42
Comp 43
Comp 44
Comp 45
Comp 46
Comp 47

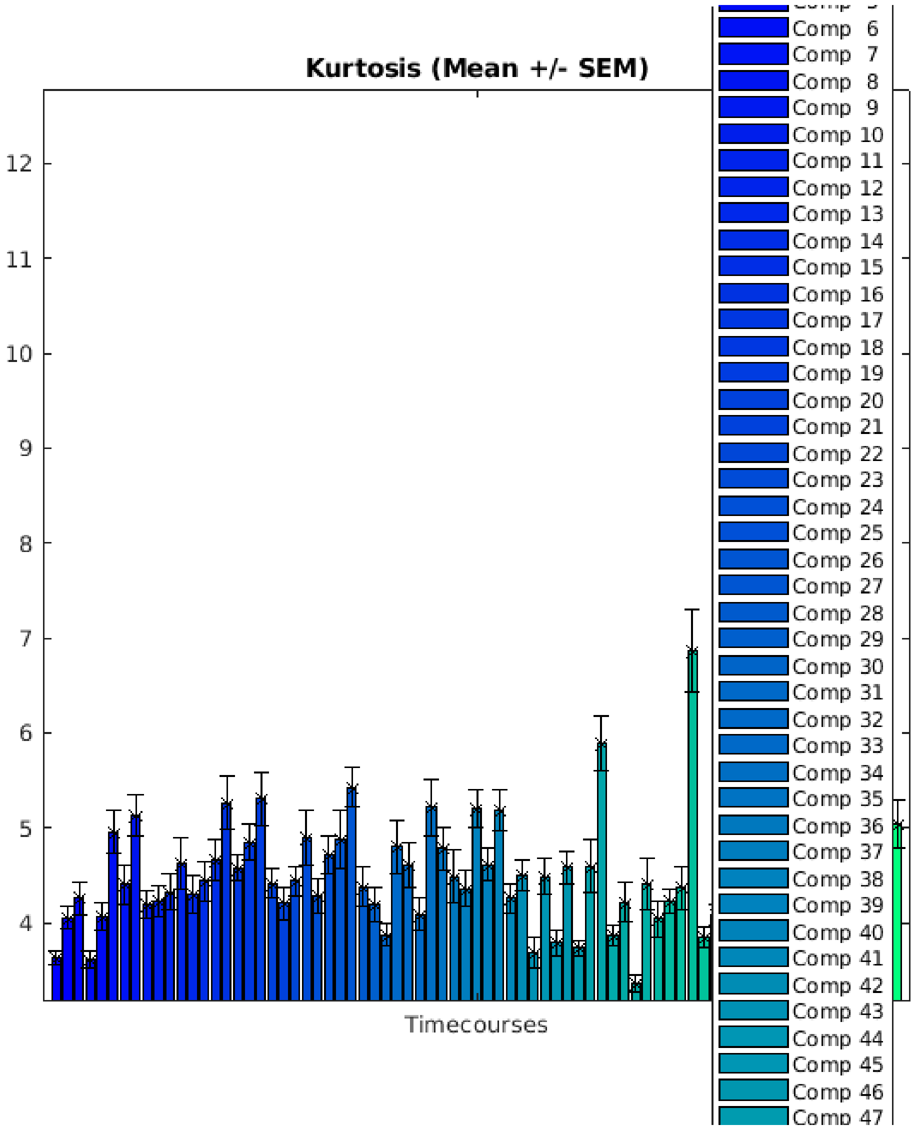

FNC correlations

Functional network connectivity correlations are computed for each data-set and averaged across sessions.

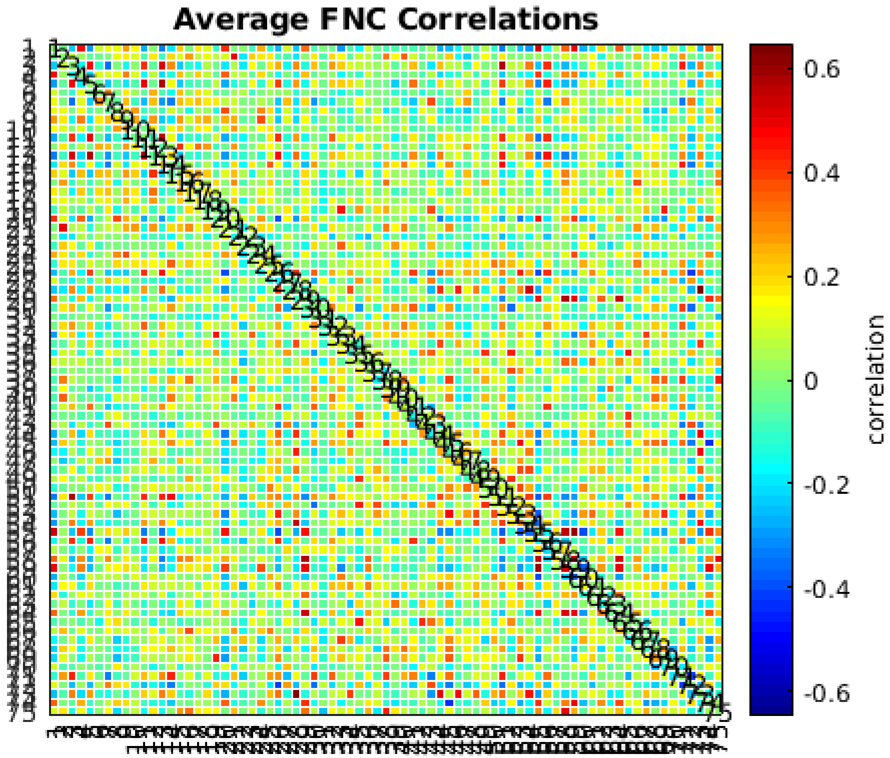

FNC metrics of component spatial maps

Mutual information is computed between components spatially and averaged across data-sets.

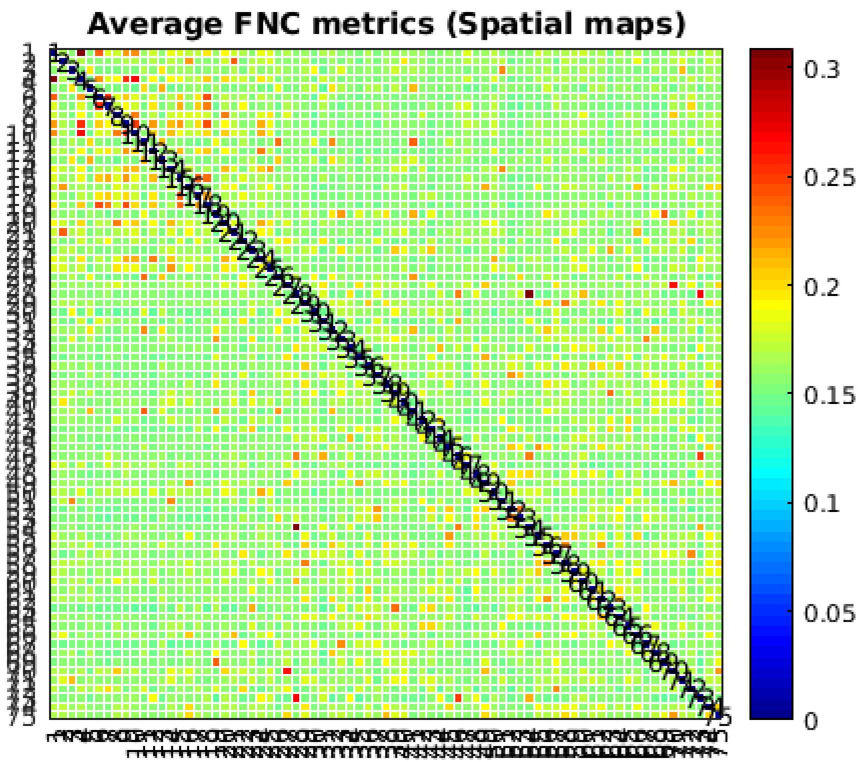

Published with MATLAB® R2019a
