# Supplementary material for: Multi-spatial-scale dynamic interactions between functional sources reveal sex-specific changes in schizophrenia
Source: Netw Neurosci. 2022 Jun 1;6(2):357–81. doi: 10.1162/netn_a_00196 (PMC9208002; doi:10.1162/netn_a_00196)
Supplement: Supplementary file 2 [file netn-06-357-s002.docx]

Supplementary 2. The summary of statistical analysis. The number of significant differences between schizophrenia (sz) group vs control group (CT) in male cohort, Male (SZ vs CT), and female cohort, Female (SZ vs CT), and sex-specific differences, Male (SZ vs CT) vs Female (SZ vs CT). #test indicates the total number of comparisons. For ICN-level analysis, it is equal to total number of within and between FNC pairs (8001). For domain-level analysis, we calculate the average FNC for within and between functional domains and across model-orders which results in 406 features.

| **Analysis** | ***static/dynamic*** | ***Male (SZ vs CT)*** | ***Female (SZ vs CT)*** | ***Male (SZ vs TP)  vs Female (SZ vs TP)*** | ***# tests*** |
| --- | --- | --- | --- | --- | --- |
| **ICN-Level** | static | 1796 | 1214 | 54 | 8001 |
|  | state1 | 318 | 222 | 12 | 8001 |
|  | state2 | 612 | 301 | 26 | 8001 |
|  | state3 | 911 | 334 | 31 | 8001 |
|  | state4 | 480 | 353 | 97 | 8001 |
| **Domain-Level** | static | 178 | 148 | 1 | 406 |
|  | state1 | 34 | 36 | 2 | 406 |
|  | state2 | 80 | 34 | 6 | 406 |
|  | state3 | 97 | 38 | 5 | 406 |
|  | state4 | 23 | 30 | 8 | 406 |
