# Supplementary material for: Multi-spatial-scale dynamic interactions between functional sources reveal sex-specific changes in schizophrenia
Source: Netw Neurosci. 2022 Jun 1;6(2):357–81. doi: 10.1162/netn_a_00196 (PMC9208002; doi:10.1162/netn_a_00196)
Supplement: Supplementary file 3 [file netn-06-357-s003.pdf]

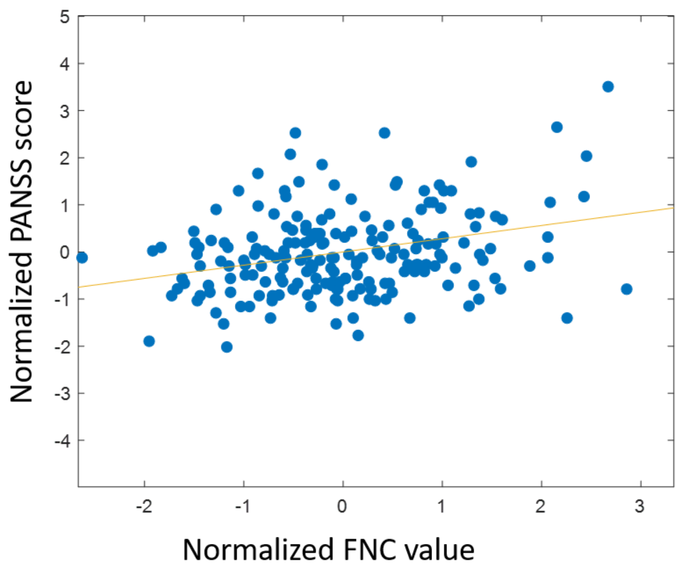

Supplementary 3. Relationship with Symptom Scores. An example of significant correlation between PANSS score and FNC pair (static FNC between SB75-SB100) after correcting for covariates. Values were normalized (zscored).
